# Asymmetric Ring Opening of Oxabicyclic Alkenes: Enhanced Rhodium Catalysis Using Camphor-Derived NHC Ligands Featuring Pyridine Coordination

**DOI:** 10.1021/acs.joc.5c02582

**Published:** 2026-01-05

**Authors:** Daniel Kamzol, Wende Chen, René Wilhelm

**Affiliations:** Institute of Organic Chemistry, 26534 Clausthal University of Technology, Leibnizstrasse 6, 38678 Clausthal-Zellerfeld, Germany

## Abstract

The synthesis, characterization, and reactivity assessment of a chiral camphor-based Rh­(I) catalyst functionalized with a pyridine moiety is presented. The catalytic system was evaluated in the asymmetric ring opening (ARO) of bicyclic alkene substrates, ensuring high product yields and enantioselectivity. While the primary focus was on the use of indoles as nucleophiles in the ARO reaction, a broader scope of bicyclic substrates was also explored. The catalyst demonstrated tolerance toward various functional groups present in the nucleophiles, underscoring its broad synthetic utility in asymmetric synthesis.

## Graphical abstract

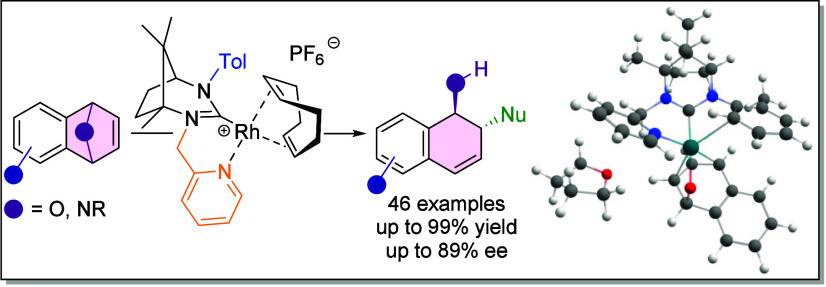

## Introduction

Asymmetric synthesis of enantiopure compounds plays a crucial role in modern synthetic chemistry, particularly for drug-related compounds.^1–3^ In order to achieve high yields, enantioselectivity, economic attractiveness, and broad functional group tolerance in asymmetric synthesis, the application of enantiopure catalysts is considered the most effective strategy.^4^ While the diversity of asymmetric catalytic systems has increased significantly, the development of novel chiral ligands for metal-based catalysts continues to be an area of active research.^5,6^ Among these chiral ligands, growing interest in the development of chiral N-heterocyclic carbenes (NHC) has been observed, owing to their unique ability to enhance the stability of metal complexes and facilitate specific stereochemical interactions.^7^

Recently, our research group has focused on the synthesis of a novel class of chiral bicyclic NHC ligands based on naturally abundant chiral pool compound camphor, using this as an economically attractive source. We evaluated these NHC ligands in ARO reactions, applying them in Ru-catalyzed asymmetric ring-opening metathesis^8^ and in Rh-catalyzed ARO of N-protected azabenzonorbornenes.^9^ Our findings indicate that incorporating additional coordinating atoms within the ligand framework enhances reaction efficiency and results in higher enantioselectivities of the products. Furthermore, we achieved high yields and enantiomeric excesses (ee) with minimal catalyst loading, avoiding the use of auxiliary additives to facilitate stable reaction conditions.^10^ In the context of ARO reactions involving oxabenzonorbornenes,^11–16^ we noted that enantioselectivity was limited by the starting materials, prompting us to seek catalysts that are not only facile to synthesize and inexpensive but also broadly applicable across various ARO substrates.

While extensive research has been conducted in this reaction,^17–20^ the number of publications focusing on transition metal NHC complexes for asymmetric induction remains limited (Scheme 1). Notably, Lautens et al.^21^ pioneered the use of chiral phosphine ligands in ARO reactions in 2000, whereas the first asymmetric reactions with NHC-based complexes were reported by Dorta^22^ in 2020, employing an iridium catalysts. Subsequent studies by Yoshida^11^ demonstrated Rh-NHC-catalyzed reactions. However, these studies typically involve a limited substrate scope, and Rh-based systems often required the addition of NaI to achieve optimal activity. Moreover, Ir catalysts are significantly more expensive than Rh catalysts.

**Scheme 1:**
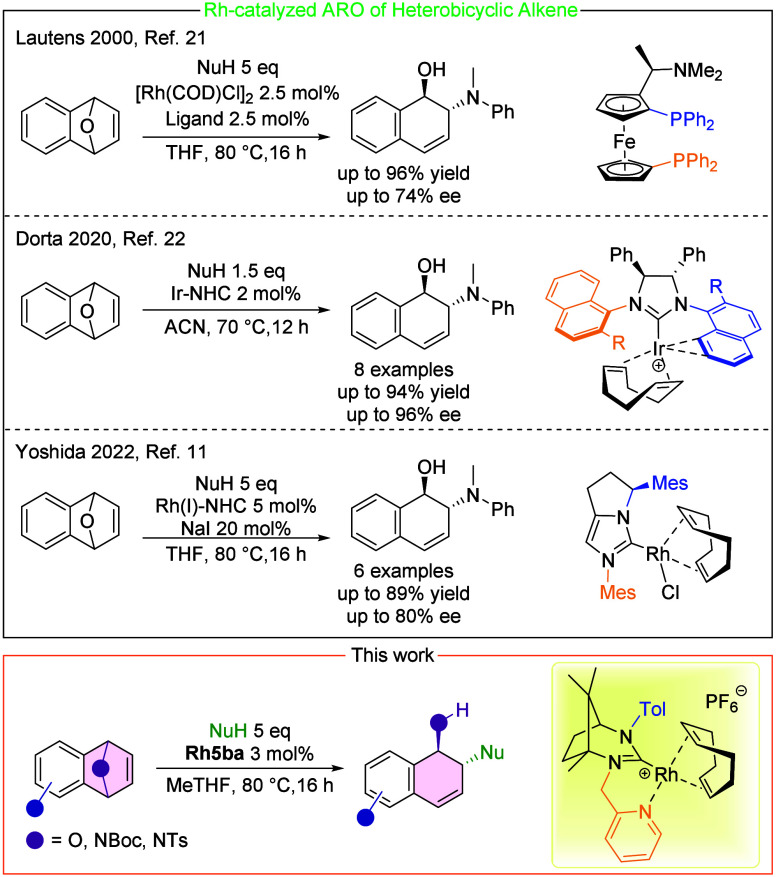
Examples of Catalytic Systems for ARO Reactions

Here, we report the synthesis and characterization of a novel Rh-NHC catalyst featuring a pyridine moiety in an enantiopure ligand, designed to enable efficient and enantioselective catalysis across a broad spectrum of ARO reactions, including those involving anilines and phenols. This work aims to expand the applicability of enantiopure NHC-based rhodium catalysts in asymmetric synthesis, providing a versatile approach to ARO transformations.

## Results and Discussion

The preparation of bicyclic chiral camphor-based ligands, as described herein,^23^ enables the synthesis of a broad spectrum of ligand structures (Scheme 2). In this study, we initiated the synthesis from camphoric diamine (**1**), which can be readily obtained from camphoric acid, a precursor derived from the chiral pool. The first step involved selective modification of one amine group via a Pd-catalyzed Buchwald–Hartwig arylamination reaction. We synthesized and characterized 14 different arylamines (**2**). These arylamines serve as key intermediates that can be subsequently transformed into NHC precursors (**3**) through a series of additional steps. The initial step in this transformation involves cyclization, followed by alkylation. Finally, an anion exchange process was employed primarily to enhance the solubility of the carbene precursors.

**Scheme 2:**
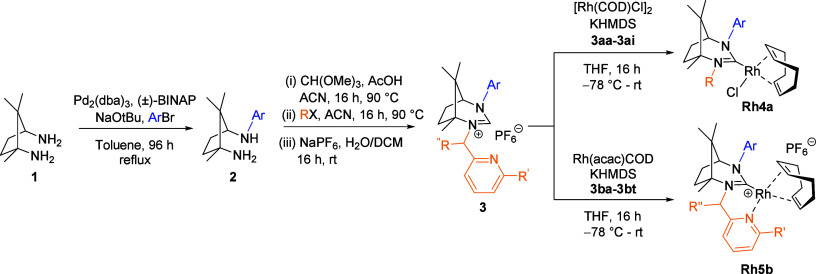
Preparation of Chiral Camphor-Based N-Heterocyclic Carbene Ligands and Mono- and Bidentate Rh-NHC Catalysts

The primary objective of this study was to systematically compare the properties of these ligands, which are depicted in Scheme 3. The synthesized ligand variants encompass both monodentate and bidentate coordination models, with an emphasis on exploring pyridine as a pendant heteroaryl substituent within NHC frameworks. The key factors under investigation included steric hindrance and electronic effects, both of which influence the properties of the ligands and the Rh complexes derived. We initially investigated monodentate ligands (**3aa–3ai**). The primary objective was to evaluate the influence of aromatic substituents on the NHC ligand and assess how steric hindrance impacts the reactivity of the Rh complexes. Specifically, compounds **3aa–3ad**, **3ah**, and **3ai** were synthesized to compare the steric effects of different aryl groups and their consequent effects on reactivity and enantioselectivity. Additionally, compounds **3ae–3ag**, featuring more alkyl substituents, were prepared to examine whether augmenting steric hindrance on this side of the molecule confers any advantages in catalytic performance.

**Scheme 3:**
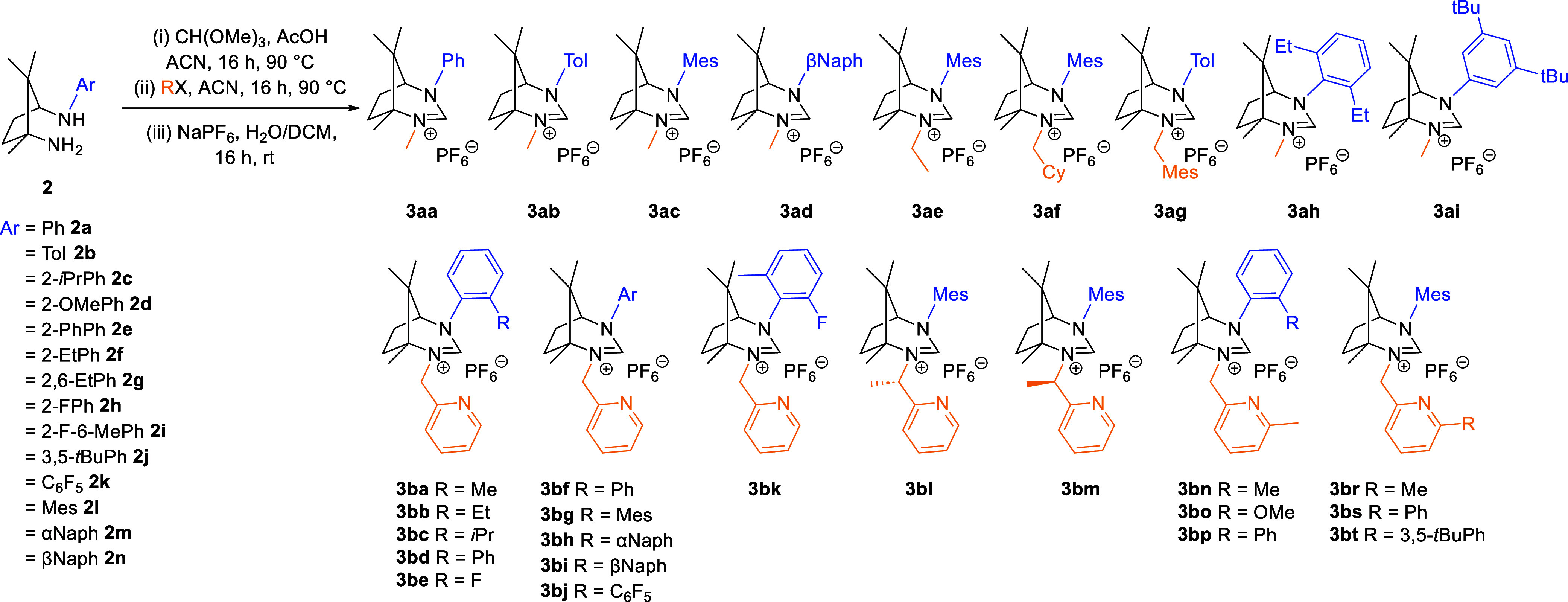
Scope of Prepared NHC Ligands

Furthermore, a series of bidentate ligands (**3ba–3bt**) were synthesized to systematically investigate both the steric properties associated with different substituents on the NHC precursor and the electronic variations arising from pyridine modifications. In this study, the carbene precursors are categorized into three main groups based on their structural features. The first group, comprising compounds **3ba–3bk**, features various aromatic substituents differing in the size and electronic properties of the aryl groups. The second group, including compounds **3bl** and **3bm**, contains additional stereocenters, which are anticipated to enhance the enantioselectivity and increase the bulkiness of the substituents on the alkyl side of the N-heterocyclic carbene. The third group, comprising compounds **3bn–3bt**, incorporates additional steric hindrance at position 6 of pyridine to evaluate its influence on the overall reactivity and selectivity.

In the subsequent step, we synthesized suitable air-stable Rh­(I)-NHC catalysts (Scheme 2) using the prepared ligands. Based on our previous research,^7,9^ we found that the formation of carbenes from camphor-based ligands is most efficient if potassium hexamethyl-disilazide (KHMDS) is used as the base. To prepare the Rh catalysts, two different strategies were employed. For monodentate ligands, the catalysts were generated by reacting [Rh­(COD)­Cl]_2_ with the appropriate carbene precursor and KHMDS in THF at −78 °C and then gradually warming the mixture to room temperature over 16 h. In the case of bidentate Rh­(I)-NHC complexes, a similar approach was taken, where a suitable carbene precursor was reacted with KHMDS in the presence of Rh­(acac)­(COD), resulting in the formation of the corresponding Rh-NHC complexes. These procedures enabled the efficient synthesis of Rh­(I)-NHC catalysts featuring both monodentate (**Rh4a**) and bidentate (**Rh5b**) ligand frameworks.

Upon successful synthesis of a broad range of Rh catalysts, we sought to evaluate their performance in asymmetric reactions to assess the properties of the ligands as well as the Rh complexes. The complexes were investigated in the ring-opening reaction of oxabenzonorbornene. In this context, we explored all prepared catalysts by comparing monodentate and bidentate ligand systems. Our goal was to elucidate the influence of ligand steric and electronic properties on catalytic performance with the aim of optimizing both efficiency and enantioselectivity in the catalytical transformation. As discussed in the Introduction, this reaction has been known for more than two decades; however, few reports have documented its application with chiral NHC complexes.^24–26^ Based on this knowledge, we decided to evaluate our synthesized complexes with the hypothesis that they may offer improved catalytic performance compared to literature-reported Rh complexes.

Our investigation began with monodentate Rh complexes (**Rh4a**). It was assumed that it is necessary to activate the Rh catalyst via the addition of NaI, which facilitates the *in situ* formation of the corresponding Rh–I species during the reaction. Notably, the **Rh4a** complexes exhibited remarkable catalytic performance under these conditions. Most complexes achieved high yields; however, the enantioselectivity remained modest, reaching approximately 99% yield and 12% ee for **Rh4ac (**see Table S1 (see pages S5 and S6)). Subsequently, we evaluated bidentate **Rh5b** complexes in two variants, with and without the addition of NaI. Considering NaI proved to be beneficial in **Rh4a** catalysis, we aimed to assess its influence on the catalytic performance of **Rh5b** species in the ARO reaction (see Table S2 (see pages S6 and S7)). Surprisingly, only **Rh5bd**, **Rh5bg**, **Rh5bl**, and **Rh5bm** exhibited low yields, whereas the remaining complexes delivered moderate to high yields. Furthermore, it was observed that the addition of NaI generally decreased the catalytic efficiency and adversely affected the enantioselectivity adversely. The most promising catalysts among those evaluated were **Rh5ba**, **Rh5bb**, and **Rh5bi** based on ligands **3ba**, **3bb**, and **3bi**, respectively (Scheme 3), which feature simple methylene–pyridine substituents on the alkyl chain and aromatic moieties that introduce slight steric hindrance.

For further optimization, **Rh5ba** was selected, affording the best yield (99%) and ee (72%) in the series. The optimized reaction conditions are summarized in Table S3 (see pages S8 and S9). Additional additives beyond NaI were also evaluated to determine their effects on the reaction. Overall, softer counterions afforded better enantioselectivity, although the addition of salts generally led to decreased yields. The final optimized conditions involved using 3 mol % **Rh5ba** catalyst, 5 equiv of a nucleophile, and 0.5 M MeTHF as the solvent at 80 °C for 16 h. Under these conditions, the addition of salts was deemed unnecessary, providing the best balance of yield and enantioselectivity. Under the optimized conditions, the scope of the catalytic reaction was extended to include a broader range of substrates.

Scheme 4 illustrates the variety of asymmetric products synthesized by using the **Rh5ba** catalyst. The catalyst was evaluated with both anilines and phenols as substrates. The study first focused on the influence of electron-withdrawing groups (EWGs) and electron-donating groups (EDGs) on aniline substrates, as well as the effect of different nucleophile sizes. High tolerance was noted across various substitutions on *N*-methylaniline (compounds **7aa**–**7bc**), affording products in up to 99% yields and ee values between 80% and 85%. Anilines with different steric bulk were also tested (**7ca**–**7f**), resulting in high to excellent yields with enantioselectivities ranging from 76% to 87%. Notably, product **7db** exhibited a significantly lower yield, which is attributed to steric hindrance caused by the isopropyl group, impeding the nucleophilic attack. Despite the reduced yield, the enantioselectivity remained high at 85%. Additionally, products **7e** and **7f** provided good yields of 76% and 65%, respectively. The lower yields in these cases are likely due to decreased nucleophilicity, stemming from the bromine substituent in **7e** and the inherently lower nucleophilicity of diaryl anilines compared to simple *N*-methylaniline in **7f**. Further investigations involved the reaction of indole compounds **7ga** and **7gb**, which delivered good yields with moderate enantioselectivities. Subsequent experiments involved derivatives of oxabenzonorbornenes tested with a series of *N*-methylanilines (**7ha**–**7jc**). Compounds **7ka** and **7kb** were prepared to elucidate the behavior of diaryl anilines; electron-withdrawing substituents in **7ka** led to reduced reaction yields, whereas electron-donating groups in **7kb** improved the yields, highlighting the influence of electronic effects on reactivity.

**Scheme 4:**
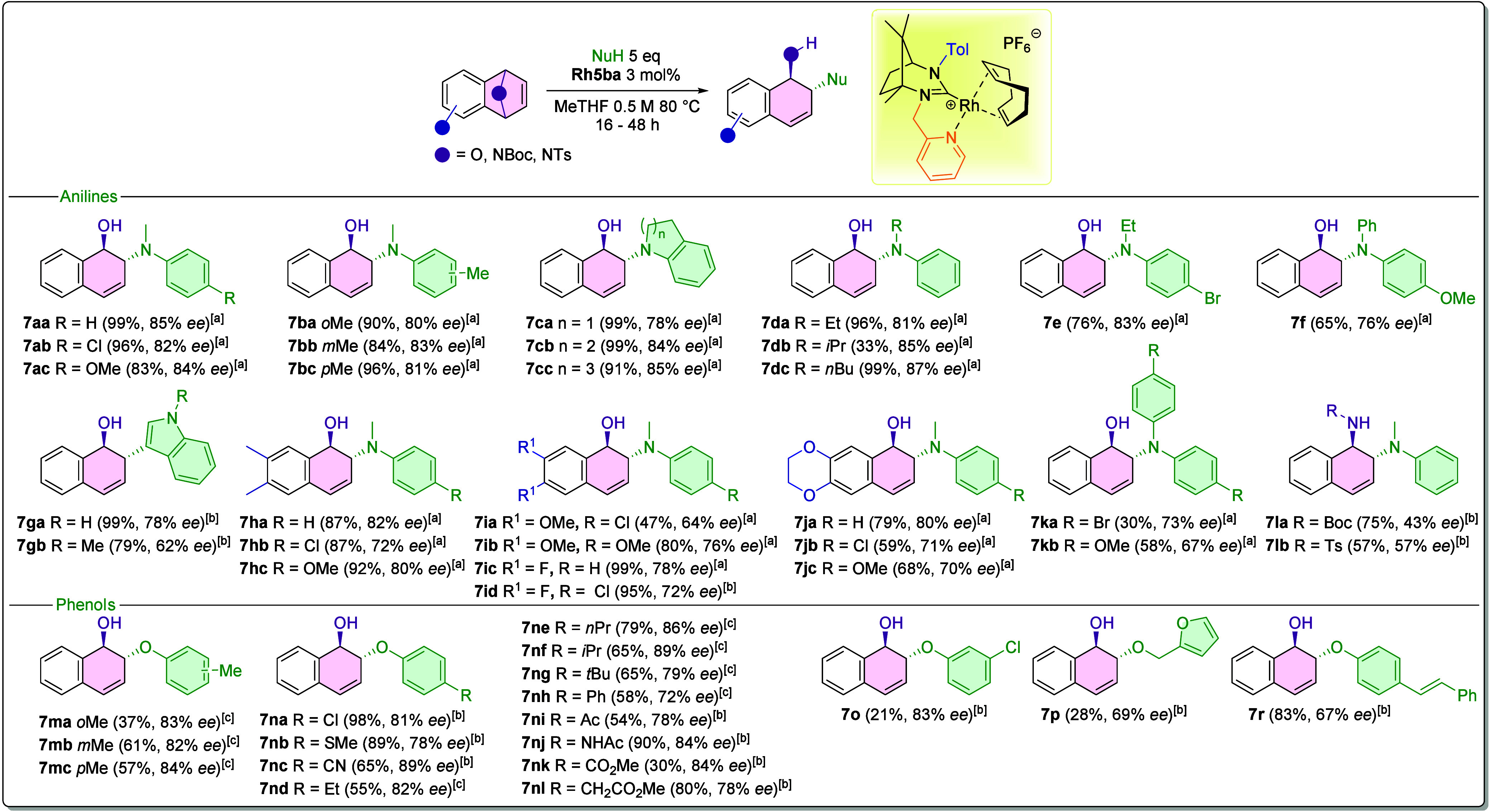
Extended Scope of the Rh-Catalyzed ARO Reaction of Heterocyclic Alkene (isolated yields and ee values determined by chiral HPLC)

a Standard conditions: **6** (0.19 mmol, 1 equiv), NuH (0.96 mmol, 5 equiv), and **Rh5ba** (3 mol %) in MeTHF (0.5 M) at 80 °C for 16 h.
b THF as the solvent.
c ACN as the solvent.

The reaction was also performed on N-protected azabenzonorbornenes **7la** and **7lb**. It was observed that N-protected alkenes afforded moderate yields; however, the enantioselectivity was significantly lower at 43% and 56%, respectively. These results confirm the reactivity of the **Rh5ba** catalyst toward azabenzonorbornene derivatives, although the enantioselectivity achieved with this complex did not yield promising outcomes. Previous studies demonstrated considerably better results with sulfur-functionalized camphor-based Rh-NHC complexes.^9^

Based on the reactions with anilines, the reactivity and selectivity of **Rh5ba** were further evaluated using phenol nucleophiles. Predominantly, reactions with cresoles (**7ma**–**7mc**) were conducted to assess the enantioselectivity and reactivity with different phenol substitutions. Good enantioselectivity was achieved for these products, approximately 83%, together with moderate yields. The yield for *o*-cresol was notably lower, likely due to steric hindrance reducing the nucleophilicity of the substrate. Subsequently, a series of *para*-substituted phenols (**7na**–**7nl** and **7r**) were examined. These reactions demonstrated high and consistent enantioselectivities, ranging from 67% to 89%, alongside favorable overall yields. A key observation is that electron-withdrawing groups in the *para* position tend to decrease the reaction yields. For example, substrate **7nk**, bearing an electron-withdrawing carbonyl ester group resulted in a yield of only 30%, while a high enantioselectivity of 84% was maintained.

Further exploration with chloro-substituted phenols, such as *m*-chlorophenol, afforded **7o** approximately 21% yield with an enantioselectivity of 83%. However, the reaction with *o*-chlorophenol was unsuccessful, indicating that catalytic system **Rh5ba** is sensitive to electron-deficient substrates, which constitutes a primary limitation. The low yield of product **7o** is attributed to side reactions that may occur, as previously described by Lautens et al.^27^ Specifically, catalyst poisoning or participation in cross-coupling reactions can reduce the overall efficiency of the desired transformation. Finally, the reactivity with an alcohol as a nucleophile was investigated. Despite the inherently lower nucleophilicity of alcohols compared to phenols, the reaction produced product **7p** in 28% yield and 69% ee.

Finally, several potential transformations (Scheme 5) are proposed to verify the synthetic utility of the prepared products. The initial step involved the large-scale synthesis of compound **7ab** (>2 mmol) under standard reaction conditions. This approach afforded an 80% yield with a reproducible ee of 83%. From this material, various modifications of the molecule were carried out.

**Scheme 5:**
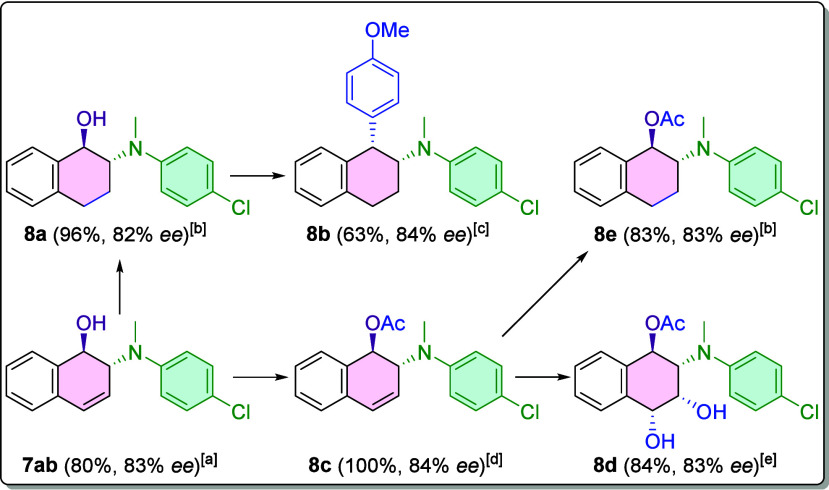
Representative Transformations of Product **7ab**

a Standard conditions: **6** (2.08 mmol, 1 equiv), NuH (10.4 mmol, 5 equiv), and **Rh5ba** (3 mol %) in MeTHF (0.5 M) at 80 °C for 16 h.
b Rh­(PPh_3_)_3_Cl and H_2_ (25 bar) in THF at rt for 16 h.
c AlCl_3_ (2 equiv) and anisole (20 equiv) in DCM at rt for 16 h.
d NaOAc (3 equiv) in Ac_2_O at 90 °C for 24 h.
e OsO_4_ (catalyst) and NMO (5 equiv) in a 7:2:1 *t*BuOH/THF/H_2_O mixture at rt for 16 h.

The first transformation involved the reduction of the double bond to produce compound **8a**, utilizing Wilkinson’s catalyst. Compound **8a** was further modified through a Friedel–Crafts-like reaction, achieved by adding AlCl_3_ as an activator and anisole as the aryl coupling partner, resulting in compound **8b**. Another functionalization pathway involved protecting the alcohol group of **7ab** to afford compound **8c**, which was accomplished via acetylation with Ac_2_O in basic media, yielding the product quantitatively with an ee of 84%. Subsequently, compound **8c** was reduced to **8e** using Wilkinson’s catalyst, following the same methodology employed for **8a**. Oxidation of the double bond was also performed to synthesize **8d**, employing NMO as the oxidant and OsO_4_ as the catalyst.

Based on a proposed mechanism shown in Scheme S2 (page S4), DFT calculations of the proposed diastereomeric intermediates **10** were carried out (for details see page S10). As one can see in Figure 1, **R10** is the energetically more favorable intermediate. Interestingly, the found intermediate also explains the need for a relatively small aryl substituent on the amidinium moiety in order to obtain higher ee values. A strong p-ligand behavior of the tolyl group toward the rhodium center is stabilizing the intermediate, which has not been reported so far.

**Figure 1:**
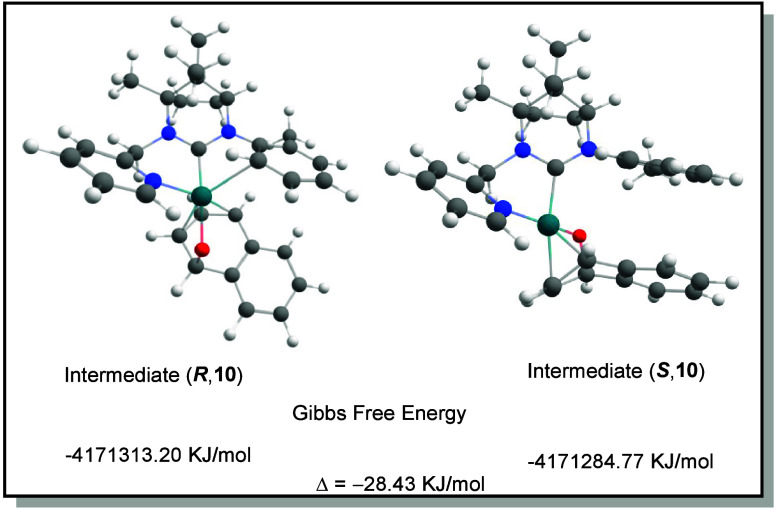
Calculated Gibbs free energies of diastereomeric intermediates **10** (*R* and *S* configurations).

If one THF molecule is added to both diastereomeric intermediates, the p-ligator group was not replaced by a THF molecule in **R10-THF**, while in **S10-THF**, the THF molecule coordinated directly to the rhodium atom in order to realize an octahedral coordination sphere as depicted in Figure 2 (for details see page S12). Again, **R10-THF** was the energetically more favorable diastereomeric intermediate. Larger substituents in the aryl rest would not allow the formation of such a p-ligator behavior, due to simple steric hindrance but also by suppressing the needed rotation of the aryl group in order to coordinate the rhodium atom. Furthermore, these findings could also explain why the enantioselectivity is quite dependent of the solvent. Polar solvents such as THF, MeTHF, ACN, and MeNO_2_ significantly enhance the enantiomeric excess, whereas nonpolar solvents produced lower ee values. This enhancement can be attributed to improved solubilization of the catalyst and reagents in polar solvents. In addition, the DFT calculations show that a coordination of the metal center with a polar solvent molecule is possible. This coordination would have a significant influence on the transition states.

**Figure 2:**
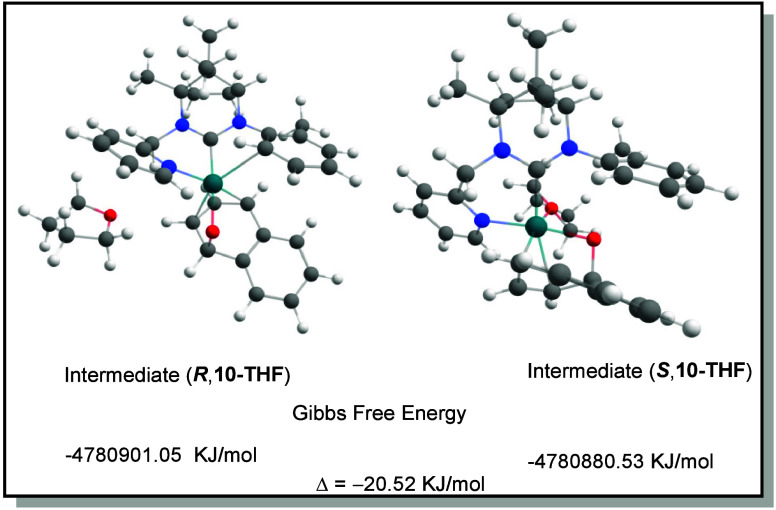
Calculated Gibbs free energies of diastereomeric intermediates **10-THF** (*R* and *S* configurations).

## Conclusions

In conclusion, this study reports the synthesis and characterization of a broad range of mono- and bidentate camphor-based N-heterocyclic carbene ligands. We successfully prepared the corresponding Rh­(I)-NHC complexes from the respective carbene precursors, enabling the evaluation of their reactivity and enantioselectivity in various asymmetric catalytic reactions. The catalytic activity of these complexes was assessed by using multiple substrates, with the best results observed in asymmetric ring-opening reactions. Specifically, we investigated the reactivity and selectivity of all prepared complexes in a reaction involving oxabenzonorbornene as the starting material. Our findings indicate that the **Rh5ba** complex is the most effective catalyst for this transformation. Optimization studies demonstrated that **Rh5ba** can be employed efficiently in the absence of any additives, with low catalyst loading (3 mol %), under mild conditions (80 °C for 16 h).

Cost analysis revealed that our propionate camphor-based ligand **3ba** is approximately 130 times more economical than the (*R*)–(*S*)-BPPFA phosphine ligands traditionally used in similar reactions. Additionally, **Rh5ba** provided higher yields and enantioselectivities compared with previously reported Rh-NHC complexes. The catalyst exhibited an expanded substrate compatibility, with limited reactivity toward nucleophiles bearing significant steric hindrance or electron-withdrawing substituents. These findings demonstrate the potential of camphor-based NHC ligands and their Rh­(I) complexes as economical and highly efficient catalysts for asymmetric transformations, with advantages in selectivity, operational simplicity, and substrate scope.

## Supplementary Material







## Data Availability

The data underlying this study are available in the TU Clausthal library at https://doi.org/10.21268/20251112-0.

## Notes

FAIR data, including the primary NMR FID files, for compounds **2**–**8** (ZIP)
FAIR data, including the primary NMR FID files, for compounds **Rh4**; **Rh5**; Rh­(SIMes); RhPh_2_(SIMes) (ZIP)
Experimental, mechanistic, and DFT calculation details and NMR spectra and data (PDF)
