# Supplementary figures and images for: Asymmetric Ring Opening of Oxabicyclic Alkenes: Enhanced Rhodium Catalysis Using Camphor-Derived NHC Ligands Featuring Pyridine Coordination
Source: J Org Chem. 2026 Jan 5;91(9):3459–65. doi: 10.1021/acs.joc.5c02582 (PMC12973293; doi:10.1021/acs.joc.5c02582)

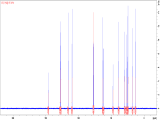

Supplement: Supplementary file 1 [file jo5c02582_si_001.zip › FID for Publication1/2f/13C/pdata/1/thumb.png]

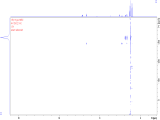

Supplement: Supplementary file 1 [file jo5c02582_si_001.zip › FID for Publication1/2f/15N HSQC/pdata/1/thumb.png]

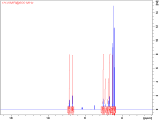

Supplement: Supplementary file 1 [file jo5c02582_si_001.zip › FID for Publication1/2f/1H/pdata/1/thumb.png]

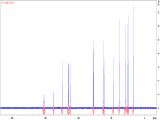

Supplement: Supplementary file 1 [file jo5c02582_si_001.zip › FID for Publication1/2h/13C/pdata/1/thumb.png]

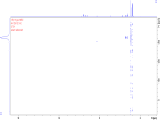

Supplement: Supplementary file 1 [file jo5c02582_si_001.zip › FID for Publication1/2h/15N HSQC/pdata/1/thumb.png]

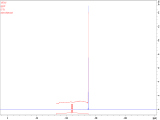

Supplement: Supplementary file 1 [file jo5c02582_si_001.zip › FID for Publication1/2h/19F/pdata/1/thumb.png]

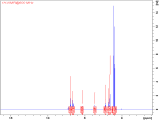

Supplement: Supplementary file 1 [file jo5c02582_si_001.zip › FID for Publication1/2h/1H/pdata/1/thumb.png]

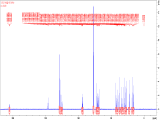

Supplement: Supplementary file 2 [file jo5c02582_si_002.zip › FID for Publication2/Rh4aa/13C/pdata/1/thumb.png]

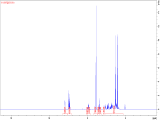

Supplement: Supplementary file 2 [file jo5c02582_si_002.zip › FID for Publication2/Rh4aa/1H/pdata/1/thumb.png]

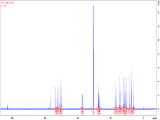

Supplement: Supplementary file 2 [file jo5c02582_si_002.zip › FID for Publication2/Rh4ab/13C/pdata/1/thumb.png]

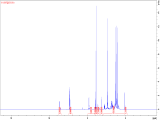

Supplement: Supplementary file 2 [file jo5c02582_si_002.zip › FID for Publication2/Rh4ab/1H/pdata/1/thumb.png]

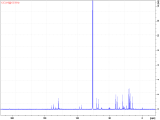

Supplement: Supplementary file 2 [file jo5c02582_si_002.zip › FID for Publication2/Rh4ac/13C/pdata/1/thumb.png]
