# Supplementary material for: Asymmetric Ring Opening of Oxabicyclic Alkenes: Enhanced Rhodium Catalysis Using Camphor-Derived NHC Ligands Featuring Pyridine Coordination
Source: J Org Chem. 2026 Jan 5;91(9):3459–65. doi: 10.1021/acs.joc.5c02582 (PMC12973293; doi:10.1021/acs.joc.5c02582)

Asymmetric Ring Opening of Oxabicyclic Alkenes: Enhanced Rhodium Catalysis Using Camphor-Derived NHC Ligands Featuring Pyridine Coordination

Daniel Kamzol, Wende Chen, René Wilhelm*

Institute of Organic Chemistry, Clausthal University of Technology, Leibnizstr. 6, 38678 Clausthal-Zellerfeld, Germany

Table of content

1. General remarks ... S2

2. Proposed mechanism ... S4

3. Optimization of catalytic reactivity for prepared Rhodium catalysts ... S5

4. DFT Calculation ... S10

5. Experimental Procedures and Characterization Data ... S14

5.1 General procedures ... S14

5.1.1 General procedure for preparation of camphor ligands (**G.P. A**) ... S14

5.1.2 General procedures for preparation of camphor catalysts (**G.P. B**) ... S32

5.1.3 Synthesis of starting materials for Asymmetric Ring Opening reaction (**G. P. C**) ... S46

5.2 General Procedure: Catalytic Asymmetric Ring Opening Reaction (**G.P. E**) ... S49

5.3 Prepared modifications for product **7ab**. ... S77

5.3.1 Scale up for synthesis with **7ab**. ... S77

5.3.2 Reduction of **7ab**. ... S77

5.3.3 Friedel-Crafts reaction of **8a**. ... S78

5.3.4 Acetylation of **7ab**. ... S79

5.3.5 Oxydation of **8c**. ... S79

5.3.6 Reduction of **8c**. ... S80

6. References ... S81

7. Copy of NMR data Analysis ... S83

1. General remarks

Unless otherwise noted, materials were purchased from commercial suppliers and used without further purification. ^1H, ^{13}C, ^{19}F NMR were recorded in $CDCl_3$ solvent on a Bruker AV-400 MHz spectrometer and chemical shifts are reported in ppm. Data are reported in the following way: chemical shift, multiplicity (s = single, d = doublet, t =triplet, q = quartet, br = broad, m = multiplet), coupling constants (Hz) and integration. Assignments are based on HSQC and HMBC spectra. Spectra were calibrated using the corresponding non-deuterated solvent signal. ^{19}F NMR (376 MHz) chemical shifts are given in ppm. Primary NMR data files were processed by MestReNova. Mass spectra were recorded on Bruker Impact2 apparatus with TOF analyzer. The optical rotations were recorded on Digital polarimeter P3000 Series, in a thermostated (20 °C) 1 dm long cell with high-pressure sodium lamp and are reported as follow: $[\alpha]_D{}^T$ [solvent, c (g/100 mL)]. HPLC analysis was performed on a Knauer Azura apparatus using Chiralpak AS-H, Dr. Maisch OD-H and AD-H equivalent columns with isopropyl alcohol and hexane as eluents. Flash-column chromatography was performed over silica gel (200–300 mesh). Thin-layer chromatography (TLC) was performed using silicagel F254 TLC plates and visualization of the developed chromatogram was performed under ultraviolet light (254 nm) and on staining by immersion in aqueous, acidic ceric ammonium molybdate followed by charring at 150 °C. All products were purified by column chromatography using silica gel (Merck 60–120 mesh). The spectra and other data were consistent with the reported values. Unless otherwise stated, all reagents were purchased from commercial suppliers and used as received. All reactions to be performed under an inert atmosphere of argon were achieved using Glovebox. Toluene (puriss. p.a., ACS reagent, ≥ 99.7% (GC)) and THF (99.9% GC) with 2,6-di-tert-butyl-4-methylphenol (250 mg/L) as stabilizer were purified by passage through a column containing activated alumina under nitrogen pressure (Dry Solvent Station GT S100, GlassTechnology, Geneva, CH). (+)-(1*R*,3*S*)-camphoric acid (95-98%), 2-(Bromomethyl)pyridine HBr salt, (*R*)-1-(pyridin-2-yl)ethan-1-ol, (*S*)-1-(pyridin-2-yl)ethan-1-ol, 2-(Bromomethyl)-6-methylpyridine were purchased from BLD, oxabenzonorbornadiene was purchased from TCI Europe, $[Rh(COD)Cl]_2$, Rh(acac)COD from Sigma-Aldrich. All the racemic compounds of the ARO reactions were synthesized in the same manner as the enantioenriched products by replacing the catalysts **Rh5b** with **RhSIMes** and NaI as an additive.

In order to compare the cost of our ligand **3ba** with one of the best performing commercially available ligands, (*R*)-(*S*)-BPPFA, in the AOR, we show here a short price estimation.

Compound	Unitary price	Amount Used	Cost
(+)-camphoric acid	0.53 €/g	15 g	7.95 €
NaN_3	0.40 €/g	14.61 g	5.84 €
	Total for 9 g of **2**		13.79 €
	Unitary cost of **2**		1.53 €
**2**	1.53 €/g	3 g	4.6 €
$Pd_2(dba)_3$	13.20 €/g	0.8 g	10.6 €
(+/-)-BINAP	1.73 €/g	1.2 g	2.08 €
NaO*t*Bu	0.2 €/g	6.1 g	1.22 €
TolBr	0.07 €/g	3.57 g	0.25 €
	Total for 4 g of **2b**		18.75 €
	Unitary cost of **2b**		4.68 €
**2b**	4.68 €/g	1.1 g	5.15 €
$CHC(OMe)_3$	0.12 €/mL	2.6 mL	0.31 €
$BrCH_2PyHBr$	1.54 €/g	2.45 g	3.77 €
$NaPF_6$	0.49 €/g	2 g	0.98 €
	Total for 0.84 g of **3ba**		10.21 €
	Unitary cost of **3ba** /g		12.15 €
	Unitary cost of **3ba** / mol		5823.32 €
	Unitary cost of (*R*)-(*S*)-BPPFA / mol		756855.00 €

All unitary prices for each chemical used in the synthesis of ligand **3ba** were calculated by dividing the listed prices of the largest available unit on Fluorochem (https://dougdiscovery.com) by the amount of each compound required. The unitary price of standard (*R*)-(*S*)-BPPFA is 1210 €/g, (756855 €/mol) while our designed **3ba** proved to be ≈ 130 times cheaper based on €/mol. However, the costs of the solvent, silica gel for column chromatography, H_2SO_4, AcOH and utilities are not included in this esteem since their cost can be considered negligible compared to the cost of applied substrates. Also working hours are not included and hence, this cost comparison is for an academic environment.

2. Proposed mechanism

Scheme S1 shows the proposed mechanism, which is similar to a previously reported Rh-catalyzed[1–3] ring-opening reactions of oxabicyclic alkenes with aniline nucleophiles. Initially, during the reaction, the catalyst interacts with the substrate by binding to the *exo*-face of oxabenzonorbornadiene. Besides, the pyridine moiety coordinates to the metal center, enhancing the stability of the resulting complex intermediate **9**. Subsequently, the rhodium species undergoes oxidative insertion between the C-N bond, leading to the formation of intermediate **10**. The next step involves nucleophilic attack by the indole at the *C3* position via the *endo*-face, resulting in intermediate **11**, which is further transformed into intermediate **12**. Finally, reductive elimination coupled with proton transfer, restoring aromaticity in the indole, yields the final product **7aa** and regenerates the active rhodium catalyst, thus completing the catalytic cycle.

Scheme S1. Proposed mechanism for the **Rh5ba** ring-opening reaction between oxabenzonorbornadiene and *N*-methylaniline.

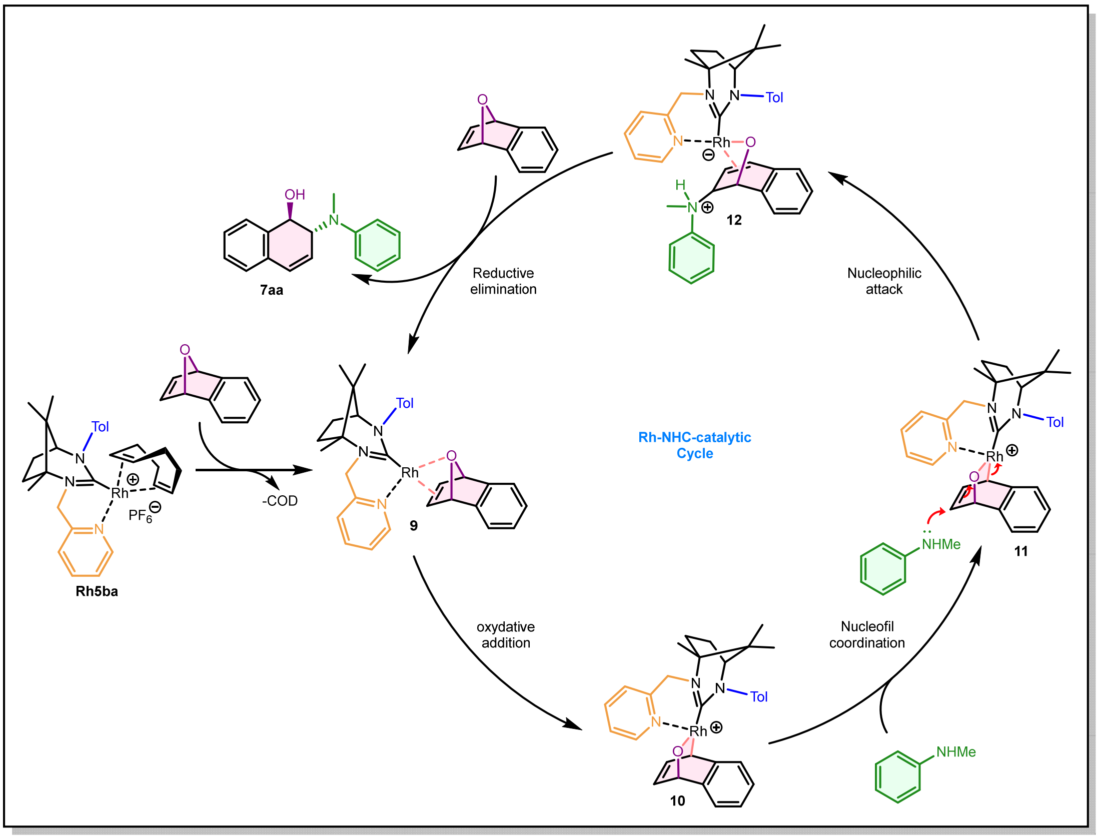

3. Optimization of catalytic reactivity for prepared Rhodium catalysts

We decided to perform catalytic ring-opening reactions for all of our catalysts, including neutral species **Rh4** as well as cationic species **Rh5**. Here, we were able to observe differences in catalytic activity between catalysts. We decided to prepare more in-depth research and screening of the reaction to determine the best results in terms of optimizing the yield and *ee* of the following reaction. The results of our studies on **Rh4** catalysts are listed in **Table S1**, where we highlight the importance of the addition of NaI salt for facilitating a good reaction. Additionally, **Table S2** contains results for **Rh5** catalysts, which also include modifications related to the additives used in the reaction, as well as a screening of the entire table of catalysts that we have prepared in our studies.

General Procedure for Asymmetric ring opening:

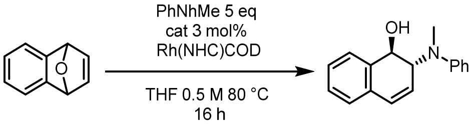

To a flame-dried test vial (5 mL), catalyst (2 mg, 3 mol%) prepared from the stock solution ($CHCl_3$) is transferred. The vial containing the catalyst is then placed in a glovebox after the solvent has evaporated. Next, oxabenzonorbornadiene (14 mg, 1 eq.) is added, followed by *N*-methylaniline (52 µL, 5 eq.), and finally, the solvent (0.2 mL, 0.5 M) is added. The vial is sealed with a Teflon cap and the temperature is set to 80 °C for 16 hours. After the reaction is complete, the vial is removed from the glovebox and subsequent manipulations are performed under ambient air. The crude mixture is transferred to a flask and the solvent is evaporated under reduced pressure. The internal standard (IS) CH_2Br_2 is added to the residue, and a crude NMR is prepared to determine the conversion and yield of the reaction.

Table S1. Catalytic activity for the asymmetric ring-opening with Rhodium complexes **Rh4**.

catalyst	solvent	NaI (20 mol%)	Conv. [%]	Yield [%]	*ee*
**Rh4ae**	-	Yes	100	67	0
**Rh4ae**	THF	-	25	0	-
**Rh4ae**	THF	Yes	99	99	3
**Rh4aa**	THF	Yes	10	7	9
**Rh4ab**	THF	Yes	15	15	13
**Rh4ac**	THF	Yes	100	99	12
**Rh4ad**	THF	Yes	10	10	4
**Rh4ae**	THF	Yes	100	100	3
**Rh4af**	THF	Yes	100	80	1
**Rh4ag**	THF	Yes	100	99	9
**Rh4ah**	THF	Yes	100	99	8
**Rh4ai**	THF	Yes	43	20	4

Conversion and yield determined by ^{1}H NMR. Enantiomeric excess determined by HPLC on OD-H column.

In our investigation of neutral species **Rh4aa** - **Rh4ai**, we initially employed the most active catalyst, **Rh4ae**, based on prior research concerning hydrogen transfer (Table S1). The first three entries suggest that the reaction can be conducted in THF as a solvent or in its absence; however, no reaction was observed in the absence of NaI. The catalytic activities of various aromatic substituents on the **Rh4a** catalysts (**Rh4aa** - **Rh4ad**, **Rh4ah**) were evaluated. Among these, **Rh4ac**, bearing a mesityl group, exhibited the highest activity.

It is hypothesized that increased steric hindrance conferred by the mesityl substituent facilitates more stable insertion of the catalyst into the substrate and minimizes side reactions such as C-H insertion on the aromatic moiety of the camphor-derived NHC ligand. Although **Rh4ai** also possesses significant steric hindrance on the aromatic segment of the ligand, it demonstrated lower catalytic yields, likely due to competitive C-H insertion into the aromatic ring. Modifications to the alkyl chain length of the NHC ligand did not result in a noticeable decrease in catalytic activity **Rh4ae** - **Rh4ag**. Nevertheless, all examined catalytic systems predominantly produced near-racemic mixtures of enantiomers.

In the subsequent step, the catalytic activity of **Rh5b**-type catalysts was evaluated. For this purpose, catalytic reactions were conducted both in the presence and absence of NaI as an additive. The obtained data from these reactions are summarized in **Table S2**.

Table S2. Catalytic activity for the asymmetric ring-opening with Rhodium complexes **Rh5**.

Entry	catalyst	NaI (20 mol%)		*ee*	No NaI		*ee*
		Conv. [%]	Yield [%]		Conv. [%]	Yield [%]	
**1**	**Rh5ba**	100	99	50	99	99	72
**2**	**Rh5bb**	-	-	-	98	95	77
**3**	**Rh5bc**	-	-	-	100	100	53
**4**	**Rh5bd**	-	-	-	18	12	20
**5**	**Rh5be**	0	0	-	100	99	20
**6**	**Rh5bf**	100	99	40	100	99	38
**7**	**Rh5bg**	6	2	11	-	-	-
**8**	**Rh5bh**	-	-	-	100	100	57
**9**	**Rh5bi**	100	99	40	100	99	71
**10**	**Rh5bj**	0	0	-	100	86	27
**11**	**Rh5bk**	2	1	33	100	99	47
**12**	**Rh5bl**	40	38	56	28	27	60
**13**	**Rh5bm**	20	1	25	-	-	-
**14**	**Rh5bn**	30	27	20	100	99	25
**15**	**Rh5bo**	100	97	9	100	99	6
**16**	**Rh5bp**	66	66	5	50	50	7
**17**	**Rh5br**	100	90	62	100	99	60
**18**	**Rh5bs**	98	90	3	-	-	-
**19**	**Rh5bt**	86	72	3	-	-	-
**20**	**$RhPh_2SIMes$**	100	100	33	100	100	26
**21**	**RhSIMes**	100	100	0	100	70	0

Conversion and yield determined by ^1H NMR. Enantiomeric excess determined by HPLC on OD-H column.

In the initial phase of our study, we synthesized a series of catalysts featuring varying degrees of steric hindrance at the aromatic side positions (entries **1 - 11**). These catalysts can be categorized into two groups based on their substituents. Entries **1-5**, substituents involve different sizes of 2-substituted phenyl groups. Entries **6 - 11**, substituents involve various aromatic substituents of differing steric balkiness.

Within the first group, the most active catalysts were those bearing methyl (**Rh5ba**) or ethyl (**Rh5bb**) substituents, also providing the highest enantioselectivity (~75% *ee*). An increase in the size of the substituent generally correlated with decreased catalytic activity and significantly influenced enantioselectivity, as exemplified by **Rh5bd**. Similarly, in the second group, catalysts with larger aromatic substituents, such as

the mesityl group (**Rh5bg**), the most sterically hindered among these, exhibited the lowest yields and enantiomeric excesses.

Furthermore, we synthesized two catalysts (entries **12** and **13**) bearing additional stereocenters on the alkyl side. These catalysts demonstrated lower yields compared to the previous series, indicating a potential negative impact of added stereocenters on catalytic efficiency.

Finally, a series of catalysts with increased steric hindrance at the 6-position of the pyridine ring (entries **14 - 19**) was prepared. Among these, increasing the size of the aromatic substituent in the ligand's aromatic portion (e.g., **Rh5bp**) led to reduced yields. Conversely, modifications at the 6-position of the pyridine ring had a comparatively minor effect on reactivity. The enantioselectivity across this series was generally low, around 30% *ee* or below, with the exception of **Rh5br**, which achieved moderate enantioselectivity (~60% *ee*).

Two additional noteworthy observations were identified. Firstly, our catalysts exhibited higher activity in the absence of the NaI additive. This finding contrasts with literature[4], where the addition of NaI generally functions as an activator, as the exchange of chalcogen ligands on rhodium often results in the formation of rhodium-iodide species that are more reactive than rhodium-chloride complexes. We suggest that, in our system, pyridine coordination to the rhodium center plays a crucial role in enhancing reactivity. The introduction of NaI appears to disrupt this coordination by causing dissociation of the pyridine ligand from the rhodium coordination sphere, thereby reducing steric hindrance and subsequently decreasing both the reaction yield and enantioselectivity.

We conducted NMR measurements to characterize our Rh-catalyst prior to and following the addition of sodium iodide. The comparative spectra are presented in **Scheme S3**.

Scheme S3. Comparison of the ^{1}H NMR spectra of **Rh5ba** prepared in $CDCl_3$, recorded prior to (red) and following (light blue) the addition of NaI.

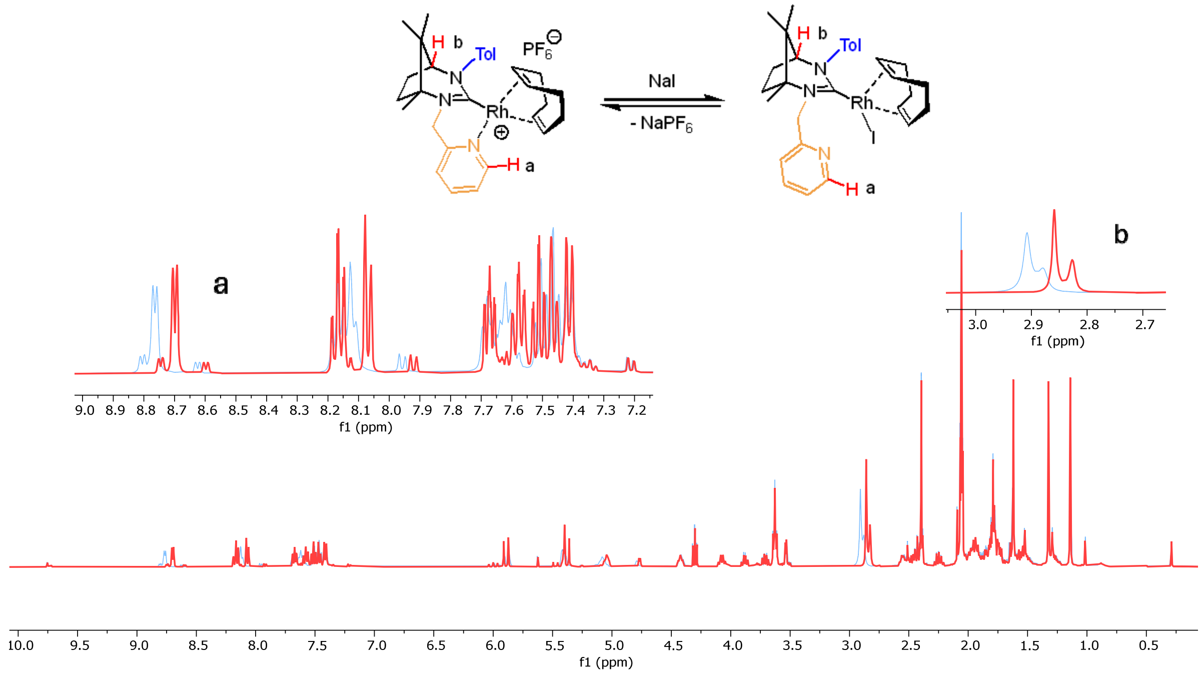

The observed spectral changes were subtle; however, a discernible shift in the NMR spectrum was detected following the addition of NaI. Notably, the most significant alterations were observed in region **A**, corresponding to pyridine protons, and in region **B**, associated with the C-H proton from the camphoric bridge

proton. These findings support the hypothesis that the addition of chalcogen salts disrupts the stability of the active catalytic species **Rh5ba**, thereby indicating a potential deactivation or modification of the catalyst's activity.

Secondly, all rhodium catalysts bearing fluorine substituents in the ligand framework (**Rh5be**, **Rh5bj**, **Rh5bk**) exhibit negligible activity in the presence of NaI, while demonstrating high yields in the absence of NaI. This suggests that the presence of fluorine atoms influences the catalyst's interaction with NaI, further affecting catalytic performance, this phenomenon is a natural consequence of the first observation.

After optimizing the most active catalyst **Rh5ba**, we decided to prepare an optimization of the additive added to the reaction, as well as the temperature, the quantity of aniline, solvents, and catalyst loading. The results of these studies are shown in **Table S3**.

In our previous discussion, we established that NaI as an additive does not promote the reactivity of the **Rh5ba** catalyst. Consequently, we aimed to evaluate the effects of various other additives in conjunction with our catalyst to determine their influence on the reaction outcome. **Table S3** summarizes these effects and presents the optimized reaction conditions.

Initially, different salts were employed to investigate the influence of counterions on catalytic performance entries **1 - 10**. Notably, salts such as $NaPF_6$ and $NaSbF_6$ yielded promising results by increasing *ee* from approximately 50% to around 80%, which is advantageous. However, these additives also led to a significant decrease in overall yield. Subsequently, we examined the effect of varying the amount of aniline added to the reaction entries **11** and **12**. The data indicated that reducing the amount of aniline resulted in a marked decrease in yield. Based on these findings, we selected 5 eq. of aniline as the optimal amount for subsequent experiments. Further experiments involved conducting the reactions in the absence of NaI and with 5 equivalents of aniline. Next, we explored the influence of temperature on the reaction entries **13 - 15**. The results showed that temperature had a negligible effect on overall reactivity but influenced enantioselectivity; therefore, subsequent reactions were performed at 80 °C. Finally, we investigated the effect of different solvents on the reaction entries **16 - 26**. Among the solvents tested, MeTHF provided the highest yield and enantioselectivity, indicating its suitability for this catalytic system.

The conclusion of this research indicates that our catalyst **Rh5ba** exhibits optimal activity in the absence of any additives. This is supported by the data presented in **Table S2**, where we evaluated the activity of the catalyst both with and without the addition of NaI. Following screening for temperature and the loading of aniline **2**, we determined that maintaining standard conditions of 80 °C and 5 eq. of aniline is optimal. Furthermore, solvent screening revealed that the reaction is most effective in polar solvents. However, each solvent can yield significantly different enantiomeric excess values. Ultimately, MeTHF emerged as the most suitable solvent for this reaction.

Only one diastereomer of the product was observed through comparison of the crude NMR spectra with previous literature.[5] Additionally, HPLC characterization was employed to analyze the product, which, in conjunction with spectroscopic data and optical rotation, provided insight into the absolute configuration of the product.

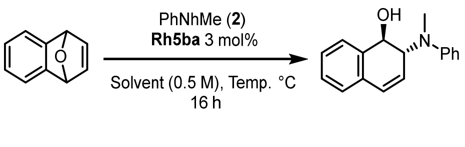

Table S3. *Optimization of reaction conditions for the ARO reaction with* ***Rh5ba****.*

**Entry**	Temp.	**2** eq	solvent	Additive (20 mol%)	Conv.[%][a] (yield)	*ee*
**1**	80 °C	5	THF	NaI	100 (99)	50
**2**	80 °C	5	THF	AgOTf	-	-
**3**	80 °C	5	THF	SnI_2	-	-
**4**	80 °C	5	THF	$NaPF_6$	99 (20)	82
**5**	80 °C	5	THF	$NaSbF_6$	30 (12)	80
**6**	80 °C	5	THF	NaBArF	100 (99)	60
**7**	80 °C	5	THF	NaOTf	100 (98)	60
**8**	80 °C	5	THF	NaBr	-	-
**9**	80 °C	5	THF	NaCl	-	-
**10**	80 °C	5	THF	$AgSbF_6$	-	-
**11**	80 °C	3	THF	NaI	82 (67)	55
**12**	80 °C	1.5	THF	NaI	43 (40)	40
**13**	80 °C	5	THF	-	99 (99)	72
**14**	60 °C	5	THF	-	98 (95)	55
**15**	100 °C	5	THF	-	100 (94)	63
**16**	80 °C	5	THF	-	100 (99)	70
**17**	80 °C	5	Dioxane	-	100 (99)	24
**18**	80 °C	5	Toluene	-	100 (99)	27
**19**	80 °C	5	ACN	-	100 (85)	72
**20**	80 °C	5	$CHCl_3$	-	90 (14)	40
**21**	80 °C	5	$MeNO_2$	-	85 (63)	78
**22**	80 °C	5	THP	-	100 (80)	27
**23**	80 °C	5	DME	-	100 (99)	64
**24**	**80 °C**	**5**	**MeTHF**	**-**	**100 (99)**	**82**
**25**[b]	80 °C	5	MeTHF	-	100 (98)	80
**26**	80 °C	5	$n\text{Bu}_2\text{O}$	-	100 (73)	80

[a] Conversion and yield determined by ^{1}H NMR. Ee determined by HPLC on OD-H column. [b] use 6 mol% of catalyst.

4. DFT Calculation

The DFT computations allowed us to investigate the enantioselectivity of the product and to elucidate the behavior of the pyridine arm within our catalyst system.

DFT calculations were performed for the first postulated intermediate **10**. The calculation of the diastereomeric intermediates give similar insights as the diastereomeric transition-states but are easier to optimize and faster to calculate, hence reducing a possible error range. Each diastereomeric intermediate was initially optimized with XTB.[6,7] After that, we used the Conformer-Rotamer Ensemble Sampling Tool (CREST), which was developed by Grimme et al.[7,8] Afterwards, the conformer set was further processed using CENSO, developed by the same group,[9,10] to predict the most important conformer according to the Boltzmann distribution. In all calculations, THF was incorporated as a solvent. The conformer with the highest Boltzmann weight was further calculated with high-level DFT with ORCA.[11,12] The structure was optimized using the functional PBE0 with a dispersion correction[13,14] and the def2-TZVP basis set[15] with a PCM solvent model for THF. Frequency calculations were free from negative values.

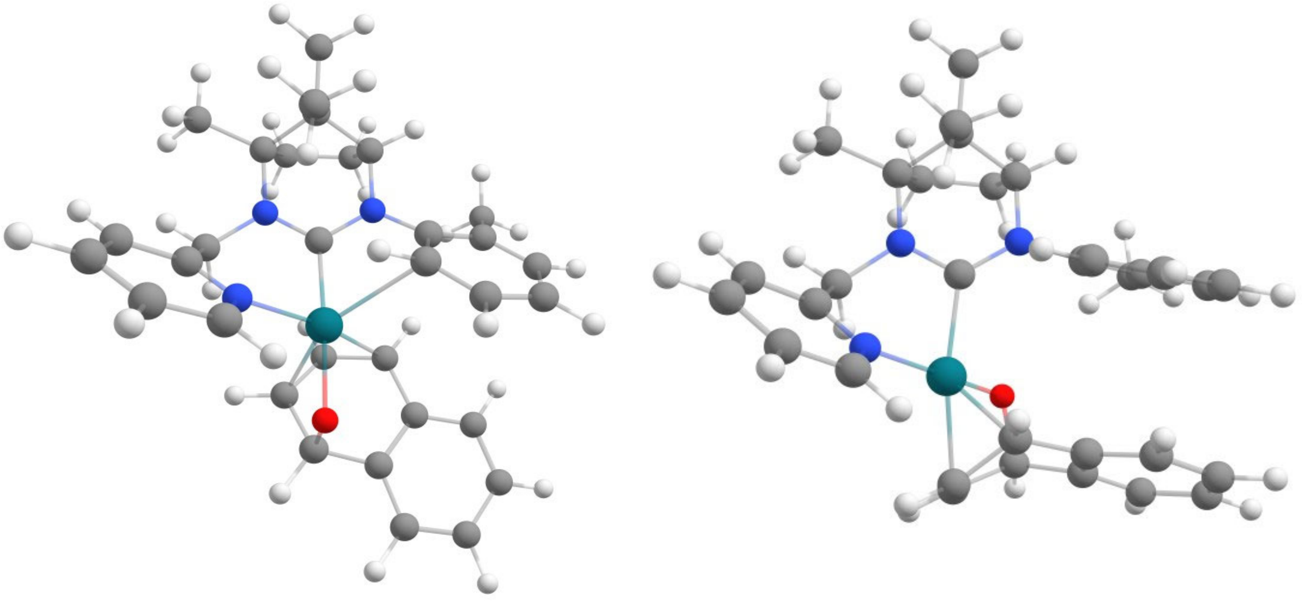

Intermediate (***R***,**10**) Intermediate (***S***,**10**)

Gibbs Free Energy

-4171313.20 KJ/mol -4171284.77 KJ/mol

Δ = –28.43 KJ/mol

X,Y,Z Coordinates for **S,10**

6	-2.725947000	-1.506186000	-0.459924000	1	-0.414351000	-3.017456000	1.383316000
6	-3.342736000	-1.760335000	0.924973000	1	-1.555268000	-3.985005000	0.447319000
6	-3.999215000	-0.425200000	1.333071000	6	4.308503000	-2.750364000	-0.636470000
6	-3.574920000	0.544912000	0.231078000	6	3.671692000	-1.548253000	-0.926012000
6	-3.552636000	-0.346583000	-1.033200000	6	4.322018000	-3.236753000	0.660506000
7	-2.144022000	0.903217000	0.515256000	1	3.673087000	-1.158763000	-1.937037000
7	-1.327253000	-1.056027000	-0.332881000	1	4.814599000	-4.176557000	0.882633000
6	-2.960853000	0.332189000	-2.258025000	6	3.049749000	-0.835278000	0.087309000
6	-4.937882000	-0.870932000	-1.404210000	6	3.700508000	-2.519765000	1.682294000
6	-1.100625000	0.134360000	0.203245000	1	3.704871000	-2.899683000	2.698833000
6	-1.810963000	2.169074000	1.168838000	6	3.068048000	-1.326030000	1.398776000
6	-1.041876000	3.049043000	0.224815000	1	4.793394000	-3.306469000	-1.430525000
45	0.671792000	0.856320000	0.870102000	6	2.272543000	-0.531933000	2.394541000
6	-0.332252000	-1.764459000	-1.070888000	6	2.483575000	0.505601000	-0.123449000

6	0.014011000	-3.068927000	-0.710516000	1	2.490311000	0.859231000	-1.149737000
6	0.881021000	-3.761044000	-1.551551000	6	2.745190000	1.460275000	0.940161000
6	1.392186000	-3.186636000	-2.704312000	1	2.924761000	2.512030000	0.753850000
6	1.064962000	-1.879047000	-3.027191000	6	2.505071000	0.971968000	2.198500000
6	0.203251000	-1.171266000	-2.206786000	1	2.485067000	1.629636000	3.060437000
6	-0.502212000	-3.704839000	0.539296000	8	0.910555000	-0.699786000	2.111501000
7	0.165661000	2.587230000	-0.139993000	1	2.514409000	-0.839696000	3.418917000
1	-4.076427000	-2.564744000	0.867755000	1	-2.726656000	-2.383722000	-1.104004000
1	-2.575765000	-2.071144000	1.632072000	6	-4.448753000	1.771801000	0.105976000
1	-3.664775000	-0.071730000	2.309361000	1	-5.430505000	1.459808000	-0.249342000
1	-5.087086000	-0.500045000	1.369622000	1	-4.606120000	2.268761000	1.063992000
1	-2.759098000	-0.403782000	-3.039181000	1	-4.044822000	2.493539000	-0.606396000
1	-2.035289000	0.868709000	-2.049469000	1	-0.079481000	-0.155600000	-2.452235000
1	-3.674368000	1.054904000	-2.659581000	1	2.062975000	-3.754793000	-3.338400000
1	-5.580253000	-0.064370000	-1.760785000	6	-1.548727000	4.231893000	-0.277669000
1	-4.832656000	-1.591317000	-2.218960000	1	-2.523071000	4.574474000	0.047514000
1	-5.445179000	-1.372577000	-0.580959000	6	-0.800706000	4.954272000	-1.193961000
1	-1.227700000	1.980552000	2.079956000	1	-1.178866000	5.883813000	-1.601766000
1	-2.716174000	2.671249000	1.491958000	6	0.880872000	3.274135000	-1.032691000
1	1.167442000	-4.772003000	-1.282112000	1	1.841231000	2.864407000	-1.314958000
1	1.472659000	-1.410680000	-3.915370000	6	0.432204000	4.459972000	-1.582559000
1	0.062641000	-4.609601000	0.764955000	1	1.050630000	4.979543000	-2.302636000

X,Y,Z Coordinates for **R,10**

6	-2.359915000	-2.419721000	0.028581000	1	0.119647000	-3.812538000	1.189559000
6	-2.661299000	-2.798756000	1.481472000	1	-0.727770000	-4.734400000	-0.046041000
6	-3.469027000	-1.608853000	2.042654000	6	5.562646000	-1.079370000	0.477264000
6	-3.538952000	-0.599275000	0.889972000	6	4.221636000	-1.386067000	0.671500000
6	-3.526389000	-1.504278000	-0.369879000	6	5.986022000	0.242590000	0.502545000
7	-2.239161000	0.155232000	0.887575000	1	3.889955000	-2.419175000	0.676891000
7	-1.124151000	-1.625681000	-0.043030000	1	7.034457000	0.479350000	0.361212000
6	-3.332177000	-0.761545000	-1.682182000	6	3.300336000	-0.366960000	0.886321000
6	-4.796384000	-2.344602000	-0.485049000	6	5.062072000	1.265905000	0.694714000
6	-1.119359000	-0.364479000	0.410191000	1	5.384055000	2.302494000	0.684516000
6	-2.169803000	1.567700000	1.250055000	6	3.722604000	0.968615000	0.871321000
6	-1.903750000	2.439791000	0.054722000	1	6.280054000	-1.876178000	0.317172000
45	0.517930000	0.712416000	0.055736000	6	2.636467000	2.001479000	0.828163000
6	-0.050372000	-1.978200000	-0.898039000	6	1.905348000	-0.640315000	1.223449000
6	0.399756000	-0.999530000	-1.792601000	1	1.609375000	-1.681548000	1.273370000
6	1.522397000	-1.214927000	-2.583545000	6	1.195664000	0.286937000	2.009878000
6	2.168941000	-2.433572000	-2.511417000	1	0.410561000	-0.017274000	2.692368000
6	1.700304000	-3.411719000	-1.641981000	6	1.435393000	1.643919000	1.711497000
6	0.603221000	-3.211597000	-0.808975000	1	0.900355000	2.431421000	2.229368000
6	0.222769000	-4.252688000	0.194884000	8	2.082366000	1.937987000	-0.460453000
7	-0.774016000	2.207323000	-0.630485000	1	3.030026000	2.997883000	1.067203000
1	-3.238692000	-3.722842000	1.517668000	1	-2.249979000	-3.268746000	-0.642887000
1	-1.740114000	-2.970514000	2.035740000	6	-4.727110000	0.330585000	0.971269000
1	-3.000990000	-1.155991000	2.917235000	1	-4.789191000	0.829964000	1.939065000
1	-4.475879000	-1.910568000	2.334361000	1	-4.713478000	1.084436000	0.182081000
1	-3.159066000	-1.471940000	-2.493792000	1	-5.635137000	-0.260900000	0.860289000
1	-2.499383000	-0.058983000	-1.667661000	1	3.040548000	-2.629435000	-3.124141000
1	-4.232572000	-0.193853000	-1.925601000	1	-0.224556000	-0.121209000	-1.979300000
1	-5.060686000	-2.858840000	0.438382000	6	-2.770625000	3.450934000	-0.321147000
1	-5.643485000	-1.725060000	-0.782688000	1	-3.674379000	3.617287000	0.251738000
1	-4.653943000	-3.100881000	-1.260801000	6	-2.467028000	4.232928000	-1.424350000
1	-1.367725000	1.707442000	1.977488000	6	-0.473174000	2.960222000	-1.690851000
1	-3.094107000	1.872599000	1.726481000	1	0.462916000	2.734034000	-2.183776000
1	1.865319000	-0.437031000	-3.254424000	6	-1.298332000	3.981334000	-2.122464000
1	2.228417000	-4.356813000	-1.573755000	1	-3.135828000	5.027372000	-1.733154000
1	0.986828000	-5.028172000	0.240023000	1	-1.018617000	4.564981000	-2.989700000

DFT calculations were performed for the first postulated intermediate **10-THF** in the same why as for **10**. However, the initial starting structure with the additional THF molecule, was obtained with the newly implemented SOLVATOR tool with the DOCKER algorithm in ORCA 6.1[16] with the optimized structures of **10**.

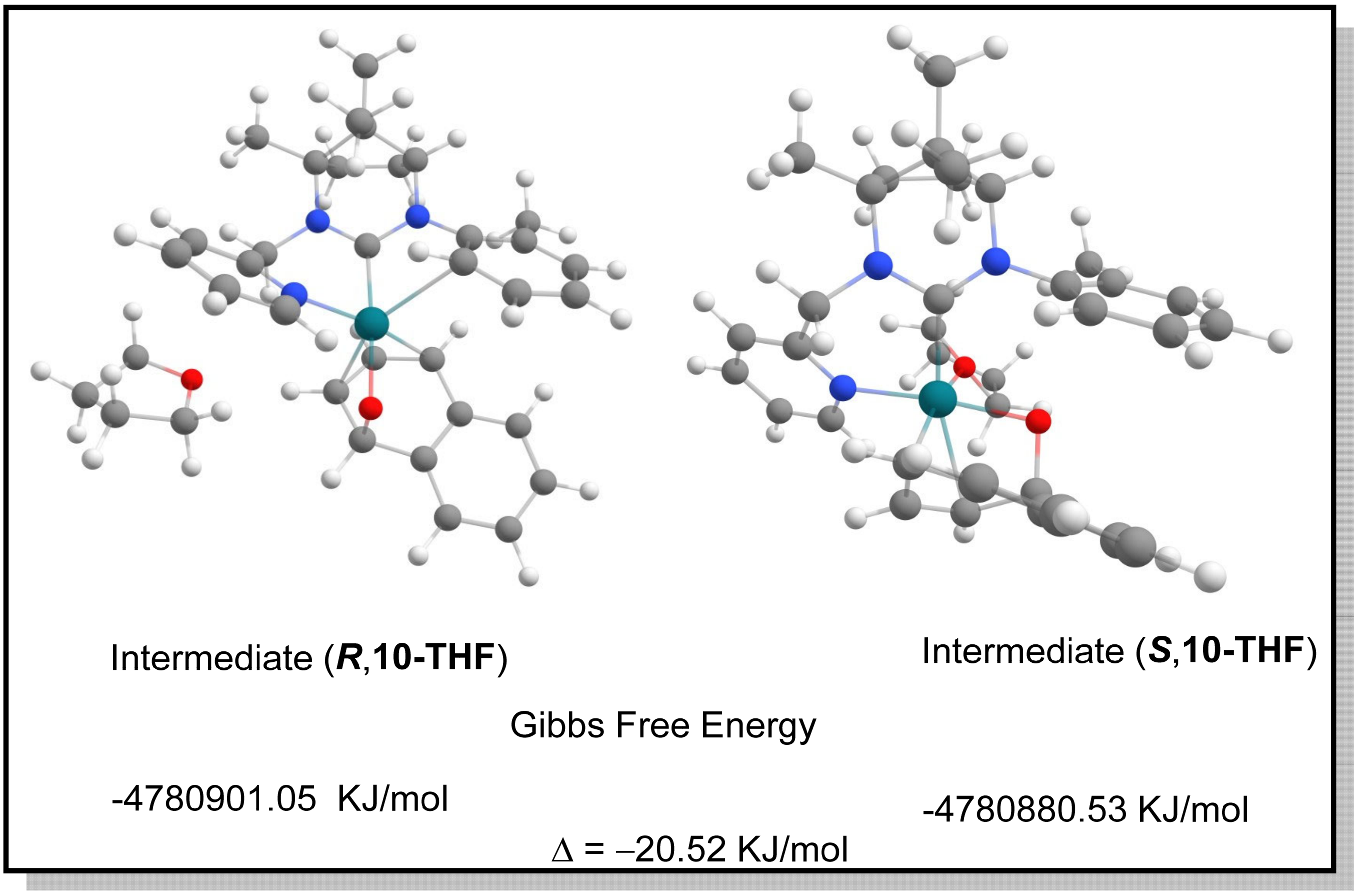

X,Y,Z Coordinates for **S,10-THF**

6	-0.981377000	-3.644428000	-0.303608000	6	-2.753857000	2.162121000	-0.806995000
6	-0.898816000	-3.959682000	-1.799162000	6	-2.582722000	4.523736000	-0.360034000
6	0.594163000	-3.796827000	-2.155191000	1	-1.972320000	5.403031000	-0.179524000
6	1.271546000	-3.367631000	-0.846778000	6	-1.972097000	3.300751000	-0.573069000
6	0.397568000	-4.046071000	0.240291000	1	-5.828715000	3.557752000	-0.525764000
7	1.056433000	-1.887272000	-0.704965000	6	-0.508596000	3.060498000	-0.353924000
7	-1.139806000	-2.196453000	-0.099425000	6	-2.069185000	0.925078000	-1.174372000
6	0.678493000	-3.584148000	1.661009000	1	-2.693527000	0.067251000	-1.393758000
6	0.520209000	-5.568087000	0.205378000	6	-0.807020000	1.000170000	-1.792767000
6	-0.103360000	-1.375944000	-0.323498000	1	-0.472521000	0.259868000	-2.510317000
6	2.161224000	-0.935352000	-0.792265000	6	0.065672000	1.980678000	-1.278460000
6	2.494561000	-0.351985000	0.551222000	1	1.079870000	2.072690000	-1.648672000
45	-0.323447000	0.530562000	0.208311000	8	-0.391710000	2.459940000	0.910861000
6	-2.195076000	-1.656646000	0.677600000	1	0.059422000	3.997080000	-0.427004000
6	-1.838782000	-0.805707000	1.730923000	1	-1.794662000	-4.145784000	0.217268000
6	-2.807507000	-0.132573000	2.466088000	6	2.735966000	-3.733506000	-0.782341000
6	-4.140519000	-0.349017000	2.174541000	1	3.286198000	-3.364281000	-1.648908000
6	-4.488615000	-1.218856000	1.147987000	1	3.214600000	-3.364385000	0.126388000
6	-3.541313000	-1.877293000	0.368817000	1	2.819063000	-4.819714000	-0.791539000
6	-3.975201000	-2.698083000	-0.803624000	1	-4.914932000	0.157523000	2.737721000
7	1.522120000	0.331222000	1.172163000	1	-0.800249000	-0.787372000	2.071297000
1	-1.243033000	-4.976747000	-1.988444000	6	3.742513000	-0.500754000	1.130775000
1	-1.537399000	-3.289564000	-2.372166000	1	4.508956000	-1.058207000	0.607242000
1	0.763833000	-3.051865000	-2.933155000	6	3.990285000	0.068189000	2.369243000
1	1.024717000	-4.735389000	-2.506935000	6	1.755985000	0.888215000	2.362751000

1	-0.069945000	-3.994453000	2.342725000	1	0.935267000	1.444063000	2.796421000
1	0.677548000	-2.500892000	1.777281000	6	2.978826000	0.776185000	2.996209000
1	1.656493000	-3.946052000	1.985032000	1	4.961843000	-0.038577000	2.836663000
1	0.423101000	-5.988551000	-0.795067000	1	3.125695000	1.240370000	3.962563000
1	1.480309000	-5.885914000	0.614229000	6	4.789385000	3.207349000	0.123931000
1	-0.263802000	-6.003428000	0.829572000	6	5.586219000	2.811961000	-1.113275000
1	1.885245000	-0.123376000	-1.466032000	6	4.687377000	1.756383000	-1.731373000
1	3.035170000	-1.422038000	-1.209011000	8	3.351037000	2.130126000	-1.400777000
1	-2.509535000	0.536232000	3.264032000	6	3.364583000	3.146010000	-0.394950000
1	-5.536883000	-1.365787000	0.910571000	1	4.936451000	2.475553000	0.922075000
1	-5.046639000	-2.583715000	-0.965279000	1	5.048274000	4.193662000	0.510256000
1	-3.455905000	-2.381209000	-1.711660000	1	5.689578000	3.666506000	-1.787208000
1	-3.769054000	-3.762115000	-0.667871000	1	6.582182000	2.430271000	-0.885962000
6	-4.747209000	3.485184000	-0.543122000	1	4.898209000	0.764989000	-1.310624000
6	-4.140784000	2.256841000	-0.774312000	1	4.774367000	1.695114000	-2.818744000
6	-3.971309000	4.619971000	-0.349351000	1	3.066612000	4.099619000	-0.847239000
1	-4.743383000	1.372100000	-0.951554000	1	2.632776000	2.886512000	0.373260000
1	-4.446267000	5.579501000	-0.179106000				

X,Y,Z Coordinates for **R,10-THF**

6	0.074941000	2.907939000	1.445461000	6	-2.295617000	-1.181458000	-2.127552000
6	1.476249000	2.892195000	2.053703000	6	-4.272130000	-2.056703000	-1.059425000
6	2.421351000	3.028620000	0.845282000	1	-4.729515000	-2.681980000	-0.298953000
6	1.488393000	3.152838000	-0.369211000	6	-2.894679000	-1.992495000	-1.155299000
6	0.215259000	3.807525000	0.215040000	1	-5.093217000	0.137218000	-3.510851000
7	1.095064000	1.757644000	-0.773700000	6	-1.944151000	-2.570220000	-0.142844000
7	-0.312952000	1.547219000	1.014800000	6	-0.830495000	-1.252447000	-2.247260000
6	-0.971653000	3.801070000	-0.733201000	1	-0.414099000	-0.718751000	-3.095415000
6	0.455981000	5.246358000	0.667316000	6	-0.241185000	-2.540910000	-1.944879000
6	0.271482000	0.984232000	-0.051659000	1	0.618761000	-2.909392000	-2.492136000
6	1.580729000	1.225744000	-2.037979000	6	-0.701148000	-3.159107000	-0.814074000
6	2.554073000	0.112205000	-1.869913000	1	-0.251542000	-4.073514000	-0.445087000
45	0.185925000	-1.045672000	-0.411779000	8	-1.483753000	-1.490509000	0.610713000
6	-1.578027000	1.104358000	1.522812000	1	-2.444570000	-3.314798000	0.489152000
6	-1.731521000	0.779753000	2.871113000	1	-0.694179000	3.252279000	2.134780000
6	-3.013592000	0.475570000	3.323260000	6	2.116326000	3.941390000	-1.495378000
6	-4.109884000	0.494459000	2.474922000	1	2.313103000	4.947777000	-1.127075000
6	-3.936295000	0.811691000	1.136206000	1	3.076458000	3.523596000	-1.803926000
6	-2.670875000	1.115271000	0.668576000	1	1.465014000	4.023772000	-2.367629000
6	-0.574806000	0.731900000	3.816475000	1	-2.520646000	1.350045000	-0.376586000
7	2.076740000	-0.972379000	-1.256040000	1	-5.095176000	0.255567000	2.859138000
1	1.591275000	3.727483000	2.745211000	6	3.861701000	0.173823000	-2.322218000
1	1.644598000	1.980814000	2.621442000	1	4.213644000	1.074502000	-2.809520000
1	3.085364000	2.171466000	0.727946000	6	4.692156000	-0.918694000	-2.142006000
1	3.049880000	3.916524000	0.926794000	1	5.718573000	-0.892984000	-2.487659000
1	-1.876124000	4.122011000	-0.211859000	6	2.873858000	-2.032669000	-1.084604000
1	-1.161645000	2.822555000	-1.171144000	1	2.436258000	-2.886743000	-0.582300000
1	-0.794968000	4.501832000	-1.552323000	6	4.185208000	-2.045521000	-1.513207000
1	1.362810000	5.368101000	1.259222000	1	4.791664000	-2.927034000	-1.352070000
1	0.521131000	5.914485000	-0.192722000	6	3.195001000	-1.955848000	2.731844000
1	-0.389060000	5.575897000	1.276954000	6	2.003500000	-2.645140000	3.385255000
1	2.037073000	2.019302000	-2.614508000	6	0.970359000	-2.596889000	2.280434000
1	0.727755000	0.860292000	-2.609475000	8	1.245929000	-1.379148000	1.558951000
1	-3.145488000	0.212506000	4.367720000	6	2.518048000	-0.842508000	1.963716000
1	-4.777216000	0.812607000	0.453276000	1	3.923558000	-1.575070000	3.447689000
1	0.261855000	0.186802000	3.377855000	1	3.703816000	-2.638448000	2.045865000
1	-0.216358000	1.732989000	4.071545000	1	2.213748000	-3.666714000	3.702187000
1	-0.862875000	0.237073000	4.744391000	1	1.663300000	-2.074819000	4.253335000
6	-4.475292000	-0.462946000	-2.852376000	1	-0.062334000	-2.553958000	2.621762000
6	-3.089662000	-0.408878000	-2.964211000	1	1.081927000	-3.437993000	1.589412000
6	-5.066604000	-1.294111000	-1.911543000	1	3.061837000	-0.515789000	1.078694000
1	-2.625915000	0.222249000	-3.715911000	1	2.339454000	0.025490000	2.603544000
1	-6.146785000	-1.338800000	-1.829550000				

5. Experimental Procedures and Characterization Data

5.1 General procedures

5.1.1 General procedure for preparation of camphor ligands (**G.P. A**)

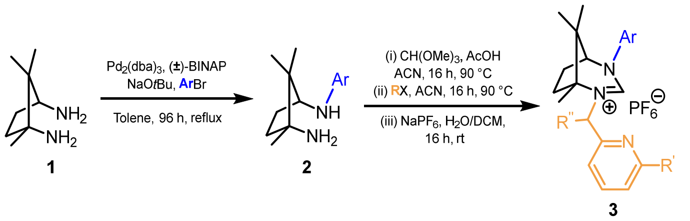

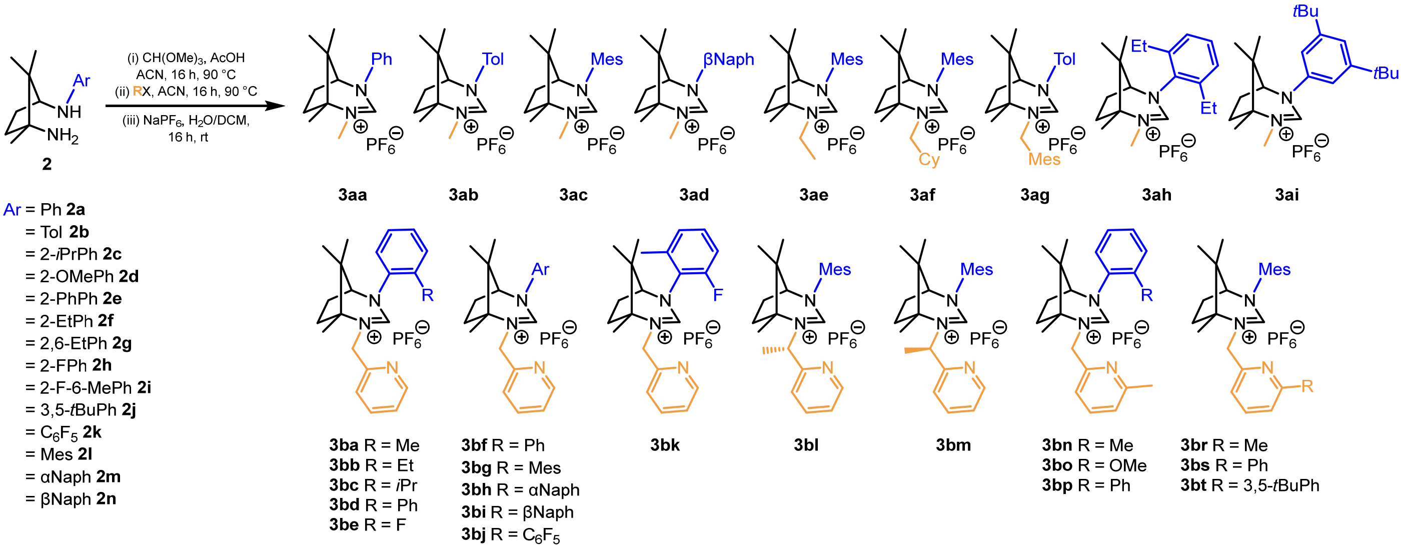

General procedure A-1 Preparation of camphor diamine. To a vigorously stirred mixture of (1*R*,3*S*)-camphoric acid (15.00 g, 75 mmol), in concentrated H_2SO_4 (75 mL) and ethanol-free chloroform (300 mL), at 60 °C (oil bath), was added sodium azide* (14.61 g, 0.23 mol) in small amounts over a period of 2 h. The mixture was then stirred for a further 24 h at 60 °C. The mixture was cooled, poured into H_2O (500 mL), and the aqueous phase extracted 3 times with $CHCl_3$. Aqueous phase made strongly basic with 12 M NaOH. The amine was extracted into $CHCl_3$ (6 × 200 mL), the organic extracts dried with anhydrous $MgSO_4$ and the chloroform removed in vacuo to give a clear oil. The oil was dissolved in diethyl ether (100 mL), the solution filtered, and the solvent removed in vacuo to give **1** as a white solid (9 g, 84 %).

*Sodium azide is a highly toxic and potentially explosive compound. Handle with extreme care in a well-ventilated fume hood, wearing appropriate personal protective equipment. Avoid inhalation, ingestion, and contact with skin and eyes. Store away from heat, acids, and incompatible materials. Proper disposal procedures must be followed to prevent environmental contamination and accidental detonation.

^{1}H NMR (400 MHz, $CDCl_3$) δ 2.95 (dd, J = 8.5, 6.7 Hz, 1H), 2.05 – 1.92 (m, 1H), 1.73 – 1.54 (m, 2H), 1.53 (s, 4H), 1.27 (ddt, J = 13.2, 10.8, 6.7 Hz, 1H), 0.98 (s, 3H), 0.77 (s, 3H), 0.75 (s, 3H).

^{13}C{^{1}H} NMR (101 MHz, $CDCl_3$) δ 60.9, 60.7, 46.1, 38.3, 30.2, 25.9, 22.2, 16.3.

The spectral data were consistent with the literature.[17]

General procedure A-2 Preparation of Cross Coupling Reaction Buchwald-Hartwig amination. In a Glovebox 100 mL vial charged with $Pd_2(dba)_3$ (5 mol%), (±)-BINAP (10 mol%) and NaO*t*Bu (3 equiv.) were dissolved in

toluene and stirred for 20 min. Diamine **1** (1 eq.) and a bromoaryl (1.1 eq.) were added and the solution was stirred for 72 h at 125°C (heating mantle). The solution was filtered through a plug of silica and the plug was eluted with additional toluene. Thereafter, the plug was eluted with DCM/MeOH (9:1 v/v) until all the product was removed from the silica plug. The solvent was evaporated under reduced pressure and the crude product was dissolved in DCM. The organic phase was extracted with 12 M HCl. The aqueous phase was separated and washed three times with DCM. Thereafter, the aqueous phase was cooled to 0 °C and 12 M NaOH was slowly added until the solution reached a pH of 14. The basic solution was extracted five times with DCM and the combined organic phases were dried over $MgSO_4$, filtered and the solvent was removed. The crude product was dissolved in pentane and the solution was filtered to remove insoluble impurities. The solvent was removed and the product **2** was further purified via chromatography if necessary. Diamines **2a**, **2b**, **2c**, **2d**, **2e**, **2g**, **2l**, **2m** where prepared according to literature procedures.[17]

General Procedure A-3: Preparation of Camphor NHC Ligands. Camphor ligands were prepared through a three-step reaction from amines **2**, involving cyclization, alkylation, and finally, anion exchange. Based on our experience, only the second step has an impact on the reaction yields; cyclization and anion exchange are usually quantitative.

In the first reaction, the corresponding amine **2** was charged into a 50 mL Schlenk flask and dissolved in 10 mL of dry ACN. To the reaction solution, trimethyl orthoformate (5 eq.) and acetic acid (5 eq.) were added. The reaction flask, equipped with a condenser and an open Schlenk stub pipe (for the slow evaporation of the produced methanol), was set to 90 °C (oil bath) for 16 hours. The crude reaction mixture was concentrated under reduced pressure to remove unreacted orthoformate and ACN. The mixture was then dissolved in DCM and extracted with a 30% KOH solution. After separating the fractions, the aqueous phase was washed two more times with DCM. The combined organic layers were dried with $MgSO_4$, filtered, and the solvent was evaporated to obtain the cyclic product in quantitative yield. The product was used in the next step without further purification.

The cyclic product was charged into a 50 mL Schlenk flask, dissolved in dry ACN (10 mL), and alkyl bromide or MeI (2.5 - 10 eq) was added. The reaction mixture was stirred at 90 °C (oil bath) for 16 hours under a nitrogen atmosphere. The crude reaction mixture was then concentrated under reduced pressure to remove solvent. In this step, a controlled TLC was typically prepared to detect the formation of organic salt. The crude mixture was used in the next step without further purification.

For the ion exchange step, the previously prepared crude mixture was dissolved in DCM (10 mL), and H_2O (10 mL), followed by addition of $NaPF_6$ (2.5 eq). The mixture was vigorously stirred overnight at room temperature. After completion of the reaction, the mixture was extracted three times with dichloromethane. The combined organic fractions were dried with $MgSO_4$, filtered through a cotton plug, and concentrated under reduced pressure. The crude reaction mixture was purified via column chromatography to obtain the final ligand **3** as the product. Ligands **3aa**, **3ab**, **3ac**, **3ae**, **3ag**, **3ah** where prepared according to literature procedures.[17–19]

(1*S*,3*R*)-*N*[1]-(2-ethylphenyl)-2,2,3-trimethylcyclopentane-1,3-diamine (2f)

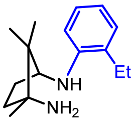

$C_{16}H_{26}N_2$
246,40 g/mol

Following **G.P. A-2**, $Pd_2(dba)_3$ (322 mg, 0.352 mmol, 5 mol%), (±)-BINAP (440 mg, 0.703 mmol, 10 mol%), and NaO*t*Bu (2.03 g, 21.09 mmol, 3 eq.) were added to anhydrous toluene (100 mL), and the reaction mixture was stirred at room temperature for 20 minutes. After this, **1** (1 g, 7.03 mmol 1 eq.) and 1-bromo-2-ethylbenzene (1.07 mL, 7.734 mmol, 1.1 eq.) were added, and the solution was stirred at 125 °C for 72 hours. After the reaction was complete, the mixture was first filtered through SiO_2 with DCM (150 mL), and the cake was washed with an additional DCM/MeOH (200 mL). The crude reaction mixture was concentrated under reduced pressure and redissolved in pure DCM for acid-base extraction. After extraction, the crude product was dried with $MgSO_4$, filtered through a cotton plug, and concentrated under reduced pressure to yield product **2f** (1.15 g, 66%) as a light yellow syrup.

Rf 0.63 (SiO_2, DCM/MeOH 9:1, v/v).

^{1}H NMR (600 MHz, $CDCl_3$) δ 7.16 (td, *J* = 7.9, 1.6 Hz, 1H), 7.12 (dd, *J* = 7.3, 1.2 Hz, 1H), 6.72 – 6.65 (m, 2H), 5.43 (s, 1H), 3.74 (s, 1H), 2.57 (qd, *J* = 7.5, 3.7 Hz, 2H), 2.34 – 2.24 (m, 1H), 1.94 – 1.85 (m, 1H), 1.73 – 1.63 (m, 2H), 1.32 (t, *J* = 7.5 Hz, 3H), 1.23 (s, 3H), 1.09 (s, 3H), 1.04 (s, 3H).

$^{13}C\{^1H\}$ NMR (151 MHz, $CDCl_3$) δ 146.0, 128.1, 128.0, 126.9, 115.8, 109.8, 62.7, 61.5, 47.3, 38.5, 29.4, 26.9, 25.1, 24.5, 17.3, 13.1.

^{15}N NMR (61 MHz, $CDCl_3$) δ -294.2, -322.1.

$[\alpha]^{25}_D$ = +156 (c = 1.00, ACN).

IR (ATR neat) ṽ =: 3348, 3318, 3066, 1577, 1508, 1450, 1146, 742 cm^{-1}.

HRMS (ESI): *m/z* calculated for $C_{16}H_{27}N_2^+$ [M + H$^+$] 247.2169; found 247.2176.

(1*S*,3*R*)-*N*[1]-(2-fluorophenyl)-2,2,3-trimethylcyclopentane-1,3-diamine (2h)

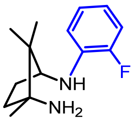

$C_{14}H_{21}FN_2$
236,33 g/mol

Following **G.P. A-2**, $Pd_2(dba)_3$ (322 mg, 0.352 mmol, 5 mol%), (±)-BINAP (440 mg, 0.703 mmol, 10 mol%), and NaO*t*Bu (2.03 g, 21.09 mmol, 3 eq.) were added to anhydrous toluene (100 mL), and the reaction mixture was stirred at room temperature for 20 minutes. After this, **1** (1 g, 7.03 mmol 1 eq.) and 1-bromo-2-fluorobenzene (0.82 mL, 7.734 mmol, 1.1 eq.) were added, and the solution was stirred at 125 °C for 72 hours. After the reaction was complete, the mixture was first filtered through SiO_2 with DCM (150 mL), and the cake was washed with an additional DCM/MeOH (200 mL). The crude reaction mixture was concentrated under reduced pressure and redissolved in pure DCM for acid-base extraction. After extraction, the crude product was dried with $MgSO_4$, filtered through a cotton plug, and concentrated under reduced pressure to yield product **2h** (1.18 g, 71%) as a light yellow syrup.

^1H NMR (600 MHz, $CDCl_3$) δ 6.98 – 6.90 (m, 2H), 6.70 (td, *J* = 8.4, 1.2 Hz, 1H), 6.53 (dddd, *J* = 9.0, 7.9, 4.8, 1.6 Hz, 1H), 5.32 (d, *J* = 7.5 Hz, 1H), 3.68 – 3.61 (m, 1H), 2.27 – 2.17 (m, 1H), 1.82 – 1.73 (m, 1H), 1.66 – 1.55 (m, 2H), 1.14 (s, 3H), 0.98 (s, 3H), 0.95 (s, 3H).

$^{13}C\{^1H\}$ NMR (151 MHz, $CDCl_3$) δ 152.7, 151.1, 137.3, 137.2, 124.5, 124.5, 115.4, 115.3, 114.5, 114.4, 112.2, 112.2, 62.5, 61.4, 47.4, 38.3, 29.3, 26.6, 24.6, 17.1.

^{15}N NMR (61 MHz, $CDCl_3$) δ -304.1, -324.8.

^{19}F NMR (377 MHz, $CDCl_3$) δ -136.8.

$[\alpha]^{25}_D$ = +158 (c = 1.00, ACN).

IR (ATR neat) ṽ =: 3302, 2961, 2868, 1615, 1509, 1452, 1334, 1254, 1225 cm^{-1}.

HRMS (ESI): *m/z* calculated for $C_{14}H_{22}FN_2^+$ [M + H$^+$] 237.1762; found 237.1776.

(1*S*,3*R*)-*N*[1]-(2-fluoro-6-methylphenyl)-2,2,3-trimethylcyclopentane-1,3-diamine (2i)

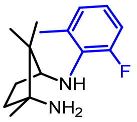

$C_{15}H_{23}FN_2$
250,36 g/mol

Following **G.P. A-2**, $Pd_2(dba)_3$ (480 mg, 0.527 mmol, 5 mol%), (±)-BINAP (660 mg, 1.055 mmol, 10 mol%), and NaO*t*Bu (3.04 g, 31.64 mmol, 3 eq.) were added to anhydrous toluene (100 mL), and the reaction mixture was stirred at room temperature for 20 minutes. After this, **1** (1.5 g, 10.55 mmol 1 eq.) and 2-bromo-1-fluoro-3-methylbenzene (1.32 mL, 10.55 mmol, 1.0 eq.) were added, and the solution was stirred at 125 °C for 72 hours. After the reaction was complete, the mixture was first filtered through SiO_2 with DCM (150 mL), and the cake was washed with an additional DCM/MeOH (200 mL). The crude reaction mixture was concentrated under reduced pressure and redissolved in pure DCM for acid-base extraction. After extraction, the crude product was dried with $MgSO_4$, filtered through a cotton plug, and concentrated under reduced pressure. Column chromatography (SiO_2, DCM/MeOH 95:5, v/v) of the crude mixture yielded **2i** (1.88 g, 71%) as a light deep brown oil.

Rf 0.33 (SiO_2, DCM/MeOH 9:1, v/v).

^1H NMR (600 MHz, $CDCl_3$) δ 6.86 – 6.79 (m, 2H), 6.61 (td, *J* = 7.8, 5.0 Hz, 1H), 4.81 (s, 1H), 3.79 (dq, *J* = 6.4, 2.3, 1.7 Hz, 1H), 2.23 (s, 3H), 2.12 – 2.02 (m, 1H), 1.78 – 1.69 (m, 1H), 1.66 – 1.57 (m, 2H), 1.12 (s, 3H), 1.03 (s, 3H), 0.91 (s, 3H).

$^{13}C\{^1H\}$ NMR (151 MHz, $CDCl_3$) δ 154.4, 152.8, 135.5, 135.4, 128.7, 128.6, 126.1, 126.0, 118.4, 118.3, 114.1, 113.9, 66.0, 65.9, 61.6, 47.3, 38.2, 29.7, 26.8, 24.3, 18.8, 18.7, 17.1.

^{15}N NMR (61 MHz, $CDCl_3$) δ -226.1, -324.0.

^{19}F NMR (377 MHz, $CDCl_3$) δ -128.8.

[α]$^{25}_{D}$ = +61 (c = 1.00, $CHCl_3$).

IR (ATR neat) ṽ =: 3351, 2963, 2868, 1614, 1579, 1478, 1387, 1309, 1271, 1214 cm^{-1}.

HRMS (ESI): *m*/*z* calculated for $C_{15}H_{24}FN_2^+$ [M + H$^+$] 251.1918; found 251.1923.

(1*S*,3*R*)-*N*[1]-(3,5-di-*tert*-butylphenyl)-2,2,3-trimethylcyclopentane-1,3-diamine (2j)

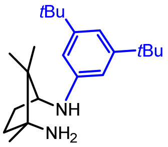

$C_{22}H_{38}N_2$
330,56 g/mol

Following **G.P. A-2**, $Pd_2(dba)_3$ (170 mg, 0.186 mmol, 5 mol%), (±)-BINAP (173 mg, 0.371 mmol, 10 mol%), and NaO*t*Bu (1.07 g, 11.14 mmol, 3 eq.) were added to anhydrous toluene (50 mL), and the reaction mixture was stirred at room temperature for 20 minutes. After this, **1** (530 mg, 3.71 mmol 1 eq.) and 1-bromo-3,5-di-*tert*-butylbenzene (1 g, 3.71 mmol, 1.0 eq.) were added, and the solution was stirred at 125 °C for 72 hours. After the reaction was complete, the mixture was first filtered through SiO_2 with DCM (150 mL), and the cake was washed with an additional DCM/MeOH (200 mL). The crude reaction mixture was concentrated under reduced pressure and redissolved in pure DCM for acid-base extraction. After extraction, the crude product was dried with $MgSO_4$, filtered through a cotton plug, and concentrated under reduced pressure to yield product **2j** (810 mg, 67%) as a white crystals syrup.

^{1}H NMR (600 MHz, $CDCl_3$) δ 6.75 (t, *J* = 1.6 Hz, 1H), 6.48 (d, *J* = 1.7 Hz, 2H), 4.60 (s, 1H), 3.66 (dd, *J* = 8.2, 5.1 Hz, 1H), 2.23 (dddd, *J* = 13.6, 10.7, 8.2, 4.4 Hz, 1H), 1.79 (ddd, *J* = 13.1, 10.5, 6.1 Hz, 1H), 1.66 (td, *J* = 13.2, 12.0, 4.2 Hz, 1H), 1.63 – 1.55 (m, 1H), 1.32 (s, 18H), 1.17 (s, 3H), 0.99 (s, 3H), 0.99 (s, 3H).

^{13}C{^{1}H} NMR (151 MHz, $CDCl_3$) δ 151.5, 147.9, 111.4, 107.9, 62.7, 61.5, 47.1, 38.3, 34.9, 31.6, 31.6, 29.4, 26.3, 24.5, 17.4.

^{15}N NMR (61 MHz, $CDCl_3$) δ -294.2, -325.9.

[α]$^{25}_{D}$ = +54 (c = 1.00, $CHCl_3$).

IR (ATR neat) ṽ =: 3348, 3280, 2958, 2864, 1593, 1511, 1435, 1335, 1222 cm^{-1}.

HRMS (ESI): *m*/*z* calculated for $C_{22}H_{39}N_2^+$ [M + H$^+$] 331.3108; found 331.3115.

(1*R*,3*S*)-1,2,2-trimethyl-*N*[3]-(perfluorophenyl)cyclopentane-1,3-diamine (2k)

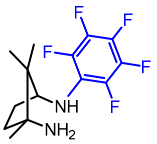

$C_{14}H_{17}F_5N_2$
308,30 g/mol

Compound **2k** was synthesized using a modified procedure from the literature.[2] Diamine **1** (1 g, 7.03 mmol, 1 equiv.) and K_2CO_3 (7.8 g, 56.27 mmol, 8 equiv.) were suspended in 30 mL of ACN and stirred at 90 °C for 1 hour. Once the mixture cooled to room temperature hexafluorobenzene (4.1 mL, 35.15 mmol, 5 equiv.) was added under a nitrogen atmosphere. The resulting suspension was then heated at 90 °C for 72 hours. Notably, the color of the suspension changed to brown just 30 min. after adding hexafluoro-benzene. After cooling, the reaction mixture was diluted with 40 mL of water and extracted with DCM (4 x 100 mL). The combined organic layers were dried over anhydrous $MgSO_4$, filtered, and the solvent was removed under reduced pressure. The crude product was purified through column chromatography (SiO_2, using petroleum ether/EtOAc 8:2, followed by 1:1, v/v), yielding **2k** (965 mg, 44%) as a light yellow liquid.

Rf 0.40 (SiO_2, DCM/MeOH 9:1, v/v).

^{1}H NMR (600 MHz, $CDCl_3$) δ 5.98 (s, 1H), 3.76 (t, *J* = 8.2 Hz, 1H), 2.20 – 2.10 (m, 1H), 1.85 – 1.75 (m, 1H), 1.69 – 1.57 (m, 2H), 1.13 (s, 3H), 0.98 (s, 3H), 0.89 (s, 3H).

^{13}C{^{1}H} NMR (151 MHz, $CDCl_3$) δ 139.2, 138.9, 137.6, 137.5, 137.3, 133.5, 131.9, 124.9, 66.3, 62.0, 48.0, 38.2, 30.1, 26.7, 25.2, 17.0.

^{15}N NMR (61 MHz, $CDCl_3$) δ -308.8, -321.7.

^{19}F NMR (377 MHz, $CDCl_3$) δ -159.5, -159.6, -165.8, -165.9, -165.9, -165.9, -174.9.

[α]$^{25}_{D}$ = +57 (c = 1.00, $CHCl_3$).

IR (ATR neat) ṽ =: 3252, 3243, 2968, 2872, 1656, 1582, 1511, 1487, 1372, 1229 cm^{-1}.

HRMS (ESI): *m*/*z* calculated for $C_{14}H_{18}F_5N_2^+$ [M + H$^+$] 309.1385; found 309.1393.

(1*R*,3*S*)-1,2,2-trimethyl-*N*[3]-(naphthalen-2-yl)cyclopentane-1,3-diamine (2n)

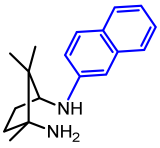

$C_{18}H_{24}N_2$
268,40 g/mol

Following **G.P. A-2**, $Pd_2(dba)_3$ (195 mg, 0.212 mmol, 5 mol%), (±)-BINAP (280 mg, 0.422 mmol, 10 mol%), and NaO*t*Bu (1.22 g, 12.65 mmol, 3 eq.) were added to anhydrous toluene (100 mL), and the reaction mixture was stirred at room temperature for 20 minutes. After this, **1** (600 mg, 4.22 mmol 1 eq.) and 2-bromonaphthalene (1.05 g, 5.06 mmol, 1.2 eq.) were added, and the solution was stirred at 125 °C for 72 hours. After the reaction was complete, the mixture was first filtered through SiO_2 with DCM (150 mL), and the cake was washed with an additional DCM/MeOH (200 mL). The crude reaction mixture was concentrated under reduced pressure and redissolved in pure DCM for acid-base extraction. After extraction, the crude product was dried with $MgSO_4$, filtered through a cotton plug, and concentrated under reduced pressure to yield product **2n** (965 mg, 85%) as a light yellow syrup.

Rf 0.41 (SiO_2, DCM/MeOH 9:1, v/v).

^{1}H NMR (600 MHz, $CDCl_3$) δ 7.64 (d, *J* = 7.9 Hz, 1H), 7.58 (t, *J* = 8.8 Hz, 2H), 7.33 (ddd, *J* = 8.2, 6.8, 1.3 Hz, 1H), 7.15 (ddd, *J* = 8.1, 6.9, 1.2 Hz, 1H), 6.88 (dd, *J* = 8.7, 2.4 Hz, 1H), 6.78 (d, *J* = 2.3 Hz, 1H), 5.34 (s, 1H), 3.78 (q, *J* = 7.2, 6.6 Hz, 1H), 2.33 – 2.23 (m, 1H), 1.88 – 1.79 (m, 1H), 1.70 – 1.59 (m, 2H), 1.17 (s, 3H), 1.00 (d, *J* = 2.2 Hz, 6H).

^{13}C{^{1}H} NMR (151 MHz, $CDCl_3$) δ 146.5, 135.6, 128.9, 127.7, 127.2, 126.2, 125.8, 121.4, 118.8, 104.2, 62.9, 61.6, 47.5, 38.4, 29.2, 26.8, 25.0, 17.4.

^{15}N NMR (61 MHz, $CDCl_3$) δ -290.4, -325.9.

$[\alpha]^{25}_D$ = +107 (c = 1.00, $CHCl_3$).

IR (ATR neat) ṽ =: 3290, 3049, 2958, 1737, 1625, 1601, 1516, 1483, 1397, 1264 cm^{-1}.

HRMS (ESI): *m*/*z* calculated for $C_{18}H_{25}N_2^+$ [M + H$^+$] 269.2012; found 269.2021.

(1*R*,5*S*)-1,2,8,8-tetramethyl-4-(naphthalen-2-yl)-2,4-diazabicyclo[3.2.1]oct-2-en-2-ium Hexafluorophosphate (3ad)

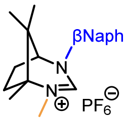

$C_{20}H_{25}F_6N_2P$
438,40 g/mol

Following **G.P. A-3**, a 50 mL Schlenk flask was charged with **2n** (500 mg, 1.86 mmol, 1 eq.), methyl orthoformate (1.02 mL, 9.3 mmol, 5 eq.), and acetic acid (0.53 mL, 9.3 mmol, 5 eq.) in 10 mL of dry ACN. The reaction mixture was stirred for 16 hours at 90 °C. After an acid-base work-up, the crude product was used in the next step without further purification. In the second step, the crude product was dissolved in 10 mL of dry ACN, followed by the addition of Iodomethane (1.16 mL, 18.6 mmol, 10 eq.). The reaction mixture was stirred for 16 hours at 90 °C under nitrogen. After the work-up, the crude product was redissolved in DCM (10 mL) and water (10 mL), followed by the addition of $NaPF_6$ (780 mg, 4.65 mmol, 2.5 eq.). The reaction mixture was stirred for 16 hours at room temperature. Column chromatography (SiO_2, DCM/petroleum ether/EtOAc 7:2:1, v/v) of the crude mixture yielded **3ad** as a light yellow solid (775 mg, 86% yield).

mp 245 °C

Rf 0.65 (SiO_2, DCM/MeOH 9:1, v/v).

^{1}H NMR (600 MHz, $CDCl_3$) δ 8.75 (s, 1H), 8.22 (d, *J* = 2.2 Hz, 1H), 7.96 – 7.91 (m, 1H), 7.79 (d, *J* = 8.8 Hz, 1H), 7.75 – 7.69 (m, 1H), 7.49 (dd, *J* = 8.8, 2.4 Hz, 1H), 7.45 – 7.38 (m, 2H), 3.86 (d, *J* = 5.2 Hz, 1H), 3.35 (s, 3H), 2.73 (ddd, *J* = 14.2, 9.5, 4.5 Hz, 1H), 2.44 (ddd, *J* = 14.4, 9.5, 4.8 Hz, 1H), 2.21 (ddt, *J* = 14.7, 12.0, 4.9 Hz, 1H), 1.90 (ddd, *J* = 14.4, 12.1, 4.8 Hz, 1H), 1.26 (s, 3H), 1.15 (s, 6H).

^{13}C{^{1}H} NMR (151 MHz, $CDCl_3$) δ 152.2, 137.3, 133.2, 132.2, 130.3, 128.3, 127.5, 127.3, 126.9, 120.9, 120.0, 71.1, 70.0, 41.4, 38.7, 38.7, 31.4, 21.8, 17.1, 14.1.

^{15}N NMR (61 MHz, $CDCl_3$) δ -241.7.

^{19}F NMR (377 MHz, $CDCl_3$) δ -71.9, -73.8.

^{31}P NMR (162 MHz, $CDCl_3$) δ -131.3, -135.7, -140.1, -144.5, -148.9, -153.3, -157.7.

$[\alpha]^{25}_D$ = +10 (c = 1.00, $CHCl_3$).

IR (ATR neat) ṽ =: 3052, 2969, 2918, 2877, 1647, 758, 566 cm^{-1}.

HRMS (ESI): m/z calculated for $C_{20}H_{25}N_2^+$ [M - PF_6^-] 293.2012; found 293.2020.

(1*R*,5*S*)-2-(cyclohexylmethyl)-4-mesityl-1,8,8-trimethyl-2,4-diazabicyclo[3.2.1]oct-2-en-2-ium Hexafluorophosphate (3af)

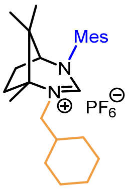

$C_{25}H_{39}F_6N_2P$
512,57 g/mol

Following **G.P. A-3**, a 50 mL Schlenk flask was charged with **2l** (500 mg, 1.92 mmol, 1 eq.), methyl orthoformate (1.05 mL, 9.61 mmol, 5 eq.), and acetic acid (0.55 mL, 9.61 mmol, 5 eq.) in 10 mL of dry ACN. The reaction mixture was stirred for 16 hours at 90 °C. After an acid-base work-up, the crude product was used in the next step without further purification. In the second step, the crude product was dissolved in 10 mL of dry ACN, followed by the addition of Cyclohexylmethyl bromide (2.68 mL, 19.22 mmol, 10 eq.). The reaction mixture was stirred for 16 hours at 90 °C under nitrogen. After the work-up, the crude product was redissolved in DCM (10 mL) and water (10 mL), followed by the addition of $NaPF_6$ (810 mg, 4.8 mmol, 2.5 eq.). The reaction mixture was stirred for 16 hours at room temperature. Column chromatography (SiO_2, Et_2O, followed by DCM) of the crude mixture yielded **3af** (570 mg, 58%) as a light yellow solid.

mp 98 °C

Rf 0.67 (SiO_2, DCM/MeOH 9:1, v/v).

^{1}H NMR (600 MHz, $CDCl_3$) δ 7.54 (s, 1H), 6.95 (s, 1H), 6.93 (s, 1H), 3.50 (d, J = 4.0 Hz, 1H), 3.41 (d, J = 6.9 Hz, 2H), 2.52 (ddd, J = 13.5, 9.4, 4.8 Hz, 1H), 2.34 (s, 3H), 2.27 (d, J = 4.8 Hz, 6H), 2.25 – 2.17 (m, 4H), 1.84 (d, J = 12.2 Hz, 1H), 1.81 – 1.72 (m, 2H), 1.70 – 1.62 (m, 2H), 1.62 – 1.54 (m, 1H), 1.47 (s, 3H), 1.34 (s, 3H), 1.31 – 1.20 (m, 3H), 1.21 (s, 3H), 1.03 – 0.99 (m, 1H).

^{13}C{^{1}H} NMR (151 MHz, $CDCl_3$) δ 155.0, 139.9, 134.9, 134.6, 134.2, 131.4, 130.6, 70.6, 70.5, 57.8, 42.2, 40.5, 38.1, 31.9, 31.8, 30.4, 30.0, 26.2, 25.9, 25.7, 25.6, 22.0, 20.9, 20.6, 19.2, 19.1, 18.7, 17.9, 14.8.

^{15}N NMR (61 MHz, $CDCl_3$) δ -235.8.

^{19}F NMR (377 MHz, $CDCl_3$) δ -71.9, -73.8.

^{31}P NMR (162 MHz, $CDCl_3$) δ -131.3, -135.7, -140.1, -144.5, -148.9, -153.3, -157.7.

$[\alpha]^{25}_D$ = -116 (c = 1.00, $CHCl_3$).

IR (ATR neat) ṽ =: 3335, 2925, 2854, 1686, 1642, 1479, 1449, 1402, 738, 555 cm^{-1}.

HRMS (ESI): m/z calculated for $C_{25}H_{39}N_2^+$ [M - PF_6^-] 367.3108; found 367.3113.

(1*R*,5*S*)-4-(3,5-di-tert-butylphenyl)-1,2,8,8-tetramethyl-2,4-diazabicyclo[3.2.1]oct-2-en-2-ium Hexafluorophosphate (3ai)

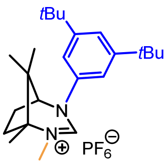

$C_{24}H_{39}F_6N_2P$
500,55 g/mol

Following **G.P. A-3**, a 50 mL Schlenk flask was charged with **2j** (670 mg, 2.03 mmol, 1 eq.), methyl orthoformate (1.1 mL, 10.13 mmol, 5 eq.), and acetic acid (0.6 mL, 10.13 mmol, 5 eq.) in 10 mL of dry ACN. The reaction mixture was stirred for 16 hours at 90 °C. After an acid-base work-up, the crude product was used in the next step without further purification. In the second step, the crude product was dissolved in 10 mL of dry ACN, followed by the addition of Iodomethane (1.15 mL, 20.03 mmol, 10 eq.). The reaction mixture was stirred for 16 hours at 90 °C under nitrogen. After the work-up, the crude product was redissolved in DCM (10 mL) and water (10 mL), followed by the addition of $NaPF_6$ (780 mg, 4.62 mmol, 2.5 eq.). The reaction mixture was stirred for 16 hours at room temperature. Column chromatography (SiO_2, DCM/MeOH 9:1, v/v) of the crude mixture yielded **3ai** (916 mg, 94%) as a light yellow solid.

mp 224 °C

Rf 0.72 (SiO_2, DCM/MeOH 9:1, v/v).

^{1}H NMR (600 MHz, $CDCl_3$) δ 7.90 (s, 1H), 7.43 (t, J = 1.5 Hz, 1H), 7.05 (d, J = 1.6 Hz, 2H), 3.73 (d, J = 5.1 Hz, 1H), 3.29 (s, 3H), 2.73 (ddd, J = 14.1, 9.4, 4.5 Hz, 1H), 2.45 (ddd, J = 14.3, 9.4, 4.8 Hz, 1H), 2.29 (ddt, J = 19.0, 11.8, 3.4 Hz, 1H), 2.05 (ddd, J = 14.7, 12.1, 4.8 Hz, 1H), 1.40 (s, 3H), 1.31 (s, 18H), 1.23 (d, J = 1.7 Hz, 6H).

^{13}C{^{1}H} NMR (151 MHz, $CDCl_3$) δ 153.8, 152.5, 140.3, 123.2, 116.8, 71.1, 70.9, 41.1, 39.1, 37.7, 35.2, 31.9, 31.3, 31.0, 21.7, 17.1, 14.1.

^{15}**N NMR** (61 MHz, $CDCl_3$) δ -237.7, -228.8.

^{19}**F NMR** (377 MHz, $CDCl_3$) δ -71.9, -73.8.

^{31}**P NMR** (162 MHz, $CDCl_3$) δ -131.3, -135.7, -140.1, -144.5, -148.9, -153.3, -157.7.

[α]$^{25}_{D}$ = +34 (c = 1.00, $CHCl_3$).

IR (ATR neat) ṽ =: 2955, 2903, 2868, 1655, 1591, 1436, 1363, 708, 556 cm^{-1}.

HRMS (ESI): *m*/*z* calculated for $C_{24}H_{39}N_2^+$ [M - PF_6^-] 355.3108; found 355.3121.

(1*R*,5*S*)-1,8,8-trimethyl-2-(pyridin-2-ylmethyl)-4-(o-tolyl)-2,4-diazabicyclo[3.2.1]oct-2-en-2-ium Hexafluorophosphate (3ba)

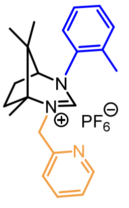

$C_{22}H_{28}F_6N_3P$
479,45 g/mol

Following **G.P. A-3**, a 50 mL Schlenk flask was charged with **2b** (1.1 g, 4.73 mmol, 1 eq.), methyl orthoformate (2.6 mL, 23.67 mmol, 5 eq.), and acetic acid (1.35 mL, 23.67 mmol, 5 eq.) in 10 mL of dry ACN. The reaction mixture was stirred for 16 hours at 90 °C. After an acid-base work-up, the crude product was used in the next step without further purification. In the second step, the crude product was dissolved in 10 mL of dry ACN, followed by the addition of 2-(Bromomethyl)pyridine (2.45 g, 14.20 mmol, 3 eq.). The reaction mixture was stirred for 16 hours at 90 °C under nitrogen. 2-(Bromomethyl)pyridine is used as an HBr salt and needs to be extracted with $NaHCO_3$ to obtain pure bromide. The yield of extraction is usually 80%. The resulting solution of pure 2-(bromomethyl)pyridine is lacrimator. After the work-up, the crude product was redissolved in DCM (10 mL) and water (10 mL), followed by the addition of $NaPF_6$ (2 g, 11.85 mmol, 2.5 eq.). The reaction mixture was stirred for 16 hours at room temperature. Column chromatography (SiO_2, DCM/MeOH 9:1, v/v) of the crude mixture yielded **3ba** (840 mg, 37%) as a light yellow solid.

mp 145 °C

Rf 0.57 (SiO_2, DCM/MeOH 9:1, v/v).

^{1}**H NMR** (600 MHz, $CDCl_3$) δ 8.59 (d, *J* = 4.5 Hz, 1H), 7.87 (s, 1H), 7.73 (t, *J* = 7.6 Hz, 1H), 7.35 (d, *J* = 7.7 Hz, 1H), 7.33 – 7.27 (m, 5H), 4.87 – 4.77 (m, 2H), 3.59 (d, *J* = 5.0 Hz, 1H), 2.53 – 2.38 (m, 5H), 2.18 – 2.09 (m, 1H), 1.89 (td, *J* = 13.8, 11.9, 3.7 Hz, 1H), 1.35 (s, 3H), 1.29 (s, 3H), 1.15 (s, 3H).

^{13}**C{^{1}H} NMR** (151 MHz, $CDCl_3$) δ 155.6, 153.6, 149.9, 139.3, 137.8, 133.5, 132.5, 129.9, 128.2, 128.1, 127.3, 123.9, 122.7, 72.1, 71.3, 54.4, 42.0, 39.7, 31.7, 21.9, 18.3, 17.7, 14.6.

^{15}**N NMR** (^{15}N NMR (61 MHz, $CDCl_3$) δ -234.7, -70.0.

^{19}**F NMR** (377 MHz, $CDCl_3$) δ -71.9, -73.8.

^{31}**P NMR** (162 MHz, $CDCl_3$) δ -131.3, -135.7, -140.1, -144.5, -148.9, -153.3, -157.7.

[α]$^{25}_{D}$ = -60 (c = 1.00, $CHCl_3$).

IR (ATR neat) ṽ =: 3359, 2953, 1728, 1647, 1437, 1374, 872, 831, 766, 554 cm^{-1}.

HRMS (ESI): *m*/*z* calculated for $C_{22}H_{28}N_3^+$ [M - PF_6^-] 334.2278; found 334.2284.

(1*R*,5*S*)-4-(2-ethylphenyl)-1,8,8-trimethyl-2-(pyridin-2-ylmethyl)-2,4-diazabicyclo[3.2.1]oct-2-en-2-ium Hexafluorophosphate (3bb)

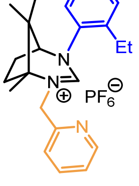

$C_{23}H_{30}F_6N_3P$
493,48 g/mol

Following **G.P. A-3**, a 50 mL Schlenk flask was charged with **2f** (500 mg, 2.03 mmol, 1 eq.), methyl orthoformate (1.1 mL, 10.15 mmol, 5 eq.), and acetic acid (0.6 mL, 10.15 mmol, 5 eq.) in 10 mL of dry ACN. The reaction mixture was stirred for 16 hours at 90 °C. After an acid-base work-up, the crude product was used in the next step without further purification. In the second step, the crude product was dissolved in 10 mL of dry ACN, followed by the addition of 2-(Bromomethyl)pyridine (1.7 g, 10.15 mmol, 5 eq.). The reaction mixture was stirred for 16 hours at 90 °C under nitrogen. 2-(Bromomethyl)pyridine is used as an HBr salt and needs to be extracted with $NaHCO_3$ to obtain pure bromide. The yield of extraction is usually 80%. The resulting solution of pure 2-(bromomethyl)pyridine is lacrimator. After the work-up, the crude product was redissolved in DCM (10 mL) and water (10 mL), followed by the addition of $NaPF_6$ (850 mg, 5.07 mmol, 2.5 eq.). The reaction mixture was stirred for 16 hours at room temperature. Column chromatography (SiO_2,

DCM/petroleum ether/EtOAc 7:1:2, v/v) of the crude mixture yielded **3bb** (680 mg, 68%) as a light yellow solid.

mp 137 °C

Rf 0.55 (SiO_2, DCM/MeOH 9:1, v/v).

^{1}H NMR (600 MHz, $CDCl_3$) δ 8.60 – 8.56 (m, 1H), 7.86 (s, 1H), 7.74 (td, *J* = 7.7, 1.8 Hz, 1H), 7.43 – 7.32 (m, 5H), 7.31 – 7.28 (m, 1H), 4.89 (d, *J* = 16.6 Hz, 1H), 4.84 (d, *J* = 16.6 Hz, 1H), 3.57 (d, *J* = 4.9 Hz, 1H), 2.78 (q, *J* = 7.2 Hz, 2H), 2.64 (ddd, *J* = 25.3, 12.8, 7.3 Hz, 1H), 2.47 (ddt, *J* = 18.5, 13.4, 6.6 Hz, 1H), 2.18 – 2.08 (m, 1H), 1.91 (ddd, *J* = 14.5, 12.2, 4.4 Hz, 1H), 1.37 (s, 3H), 1.34 – 1.28 (m, 6H), 1.17 (s, 3H).

^{13}C{^{1}H} NMR (151 MHz, $CDCl_3$) δ 155.9, 153.8, 149.8, 139.6, 137.8, 130.5, 130.4, 128.1, 123.9, 122.8, 72.1, 54.4, 42.1, 39.7, 31.6, 24.0, 22.2, 17.7, 15.4, 14.7.

^{15}N NMR (61 MHz, $CDCl_3$) δ -234.6, -68.6.

^{19}F NMR (377 MHz, $CDCl_3$) δ -71.9, -73.8.

^{31}P NMR (162 MHz, $CDCl_3$) δ -131.3, -135.7, -140.1, -144.5, -148.9, -153.3, -157.7.

$[\alpha]^{25}_D$ = -43 (c = 1.00, $CHCl_3$).

IR (ATR neat) ṽ =: 2955, 2922, 2852, 1731, 1643, 1593, 1492, 762, 555 cm^{-1}.

HRMS (ESI): *m/z* calculated for $C_{23}H_{30}N_3^+$ [M - PF_6^-] 348.2434; found 348.2446.

(1*R*,5*S*)-4-(2-isopropylphenyl)-1,8,8-trimethyl-2-(pyridin-2-ylmethyl)-2,4-diazabicyclo[3.2.1]oct-2-en-2-ium Hexafluorophosphate (3bc)

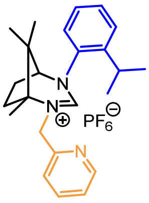

$C_{24}H_{32}F_6N_3P$
507,51 g/mol

Following **G.P. A-3**, a 50 mL Schlenk flask was charged with **2c** (500 mg, 1.92 mmol, 1 eq.), methyl orthoformate (1.05 mL, 9.6 mmol, 5 eq.), and acetic acid (0.55 mL, 9.6 mmol, 5 eq.) in 10 mL of dry ACN. The reaction mixture was stirred for 16 hours at 90 °C. After an acid-base work-up, the crude product was used in the next step without further purification. In the second step, the crude product was dissolved in 10 mL of dry ACN, followed by the addition of 2-(Bromomethyl)pyridine (1.64 g, 9.6 mmol, 5 eq.). The reaction mixture was stirred for 16 hours at 90 °C under nitrogen. 2-(Bromomethyl)pyridine is used as an HBr salt and needs to be extracted with $NaHCO_3$ to obtain pure bromide. The yield of extraction is usually 80%. The resulting solution of pure 2-(bromomethyl)pyridine is lacrimator. After the work-up, the crude product was redissolved in DCM (10 mL) and water (10 mL), followed by the addition of $NaPF_6$ (810 mg, 4.8 mmol, 2.5 eq.). The reaction mixture was stirred for 16 hours at room temperature. Column chromatography (SiO_2, DCM/petroleum ether/EtOAc 7:1:2, v/v) of the crude mixture yielded **3bc** (500 mg, 52%) as a light yellow solid.

mp 220 °C

Rf 0.56 (SiO_2, DCM/MeOH 9:1, v/v).

^{1}H NMR (600 MHz, TCE) δ 8.66 (d, *J* = 4.8 Hz, 1H), 7.86 (d, *J* = 9.5 Hz, 2H), 7.55 – 7.47 (m, 2H), 7.43 (dd, *J* = 14.4, 6.3 Hz, 2H), 7.39 (ddd, *J* = 8.8, 6.3, 2.4 Hz, 1H), 7.30 (d, *J* = 7.1 Hz, 1H), 4.86 (d, *J* = 4.0 Hz, 2H), 3.62 (d, *J* = 4.8 Hz, 1H), 3.33 – 3.25 (m, 1H), 2.60 – 2.51 (m, 1H), 2.48 (d, *J* = 11.1 Hz, 1H), 2.20 (ddt, *J* = 14.7, 12.3, 4.8 Hz, 1H), 1.98 (ddd, *J* = 14.4, 12.3, 4.2 Hz, 1H), 1.44 (s, 3H), 1.37 (d, *J* = 6.8 Hz, 3H), 1.36 (s, 3H), 1.35 (d, *J* = 6.7 Hz, 3H), 1.22 (s, 3H).

^{13}C{^{1}H} NMR (151 MHz, TCE) δ 158.8, 156.1, 152.5, 147.7, 141.9, 140.9, 133.9, 131.2, 131.1, 130.3, 127.5, 126.2, 75.7, 75.5, 57.3, 45.1, 42.9, 34.7, 31.0, 27.8, 26.8, 25.0, 20.7, 20.5, 17.7.

^{15}N NMR (61 MHz, TCE) δ -70.5, -237.7.

^{19}F NMR (377 MHz, TCE) δ -71.9, -73.8.

^{31}P NMR (162 MHz, TCE) δ -131.3, -135.7, -140.1, -144.5, -148.9, -153.3, -157.7.

$[\alpha]^{25}_D$ = -25 (c = 1.00, $CHCl_3$).

IR (ATR neat) ṽ =: 2965, 2878, 1643, 1593, 1436, 1371, 1081, 832, 771, 555 cm^{-1}.

HRMS (ESI): *m/z* calculated for $C_{24}H_{32}N_3^+$ [M - PF_6^-] 362.2591; found 362.2603.

(1*R*,5*S*)-4-([1,1'-biphenyl]-2-yl)-1,8,8-trimethyl-2-(pyridin-2-ylmethyl)-2,4-diazabicyclo[3.2.1]oct-2-en-2-ium Hexafluorophosphate (3bd)

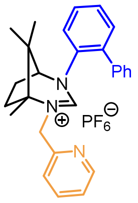

$C_{27}H_{30}F_6N_3P$
541,52 g/mol

Following **G.P. A-3**, a 50 mL Schlenk flask was charged with **2e** (250 mg, 0.82 mmol, 1 eq.), methyl orthoformate (0.45 mL, 4.1 mmol, 5 eq.), and acetic acid (0.235 mL, 4.1 mmol, 5 eq.) in 10 mL of dry ACN. The reaction mixture was stirred for 16 hours at 90 °C. After an acid-base work-up, the crude product was used in the next step without further purification. In the second step, the crude product was dissolved in 10 mL of dry ACN, followed by the addition of 2-(Bromomethyl)pyridine (560 mg, 3.29 mmol, 4 eq.). The reaction mixture was stirred for 16 hours at 90 °C under nitrogen. 2-(Bromomethyl)pyridine is used as an HBr salt and needs to be extracted with $NaHCO_3$ to obtain pure bromide. The yield of extraction is usually 80%. The resulting solution of pure 2-(bromomethyl)pyridine is lacrimator. After the work-up, the crude product was redissolved in DCM (10 mL) and water (10 mL), followed by the addition of $NaPF_6$ (350 mg, 2.05 mmol, 2.5 eq.). The reaction mixture was stirred for 16 hours at room temperature. Column chromatography (SiO_2, DCM/petroleum ether/EtOAc 7:2:1, followed by 7:1:2, v/v) of the crude mixture yielded **3bd** (355 mg, 80%) as a light yellow solid.

mp 209 °C

Rf 0.63 (SiO_2, DCM/MeOH 9:1, v/v).

^{1}H NMR (600 MHz, CD_2Cl_2) δ 8.74 (ddd, *J* = 4.8, 1.7, 0.9 Hz, 1H), 8.14 (s, 1H), 7.82 (td, *J* = 7.7, 1.8 Hz, 1H), 7.64 – 7.57 (m, 2H), 7.56 – 7.51 (m, 3H), 7.51 – 7.48 (m, 1H), 7.48 – 7.45 (m, 1H), 7.44 – 7.39 (m, 3H), 7.34 (d, *J* = 7.7 Hz, 1H), 4.82 (d, *J* = 16.2 Hz, 1H), 4.75 (d, *J* = 16.2 Hz, 1H), 3.34 (d, *J* = 4.5 Hz, 1H), 2.21 (d, *J* = 9.9 Hz, 1H), 1.90 – 1.84 (m, 1H), 1.84 – 1.71 (m, 2H), 1.31 (s, 3H), 0.95 (s, 3H), 0.78 (s, 3H).

$^{13}C\{^1H\}$ NMR (151 MHz, CD_2Cl_2) δ 155.5, 153.2, 150.0, 138.7, 138.2, 137.8, 137.7, 132.5, 129.8, 129.6, 129.5, 128.9, 128.5, 127.7, 123.9, 122.5, 72.0, 71.6, 54.3, 41.8, 39.4, 30.4, 21.4, 16.8, 14.4.

^{15}N NMR (61 MHz, CD_2Cl_2) δ -234.3, -69.33.

^{19}F NMR (377 MHz, CD_2Cl_2) δ -71.9, -73.8.

^{31}P NMR (162 MHz, CD_2Cl_2) δ -131.3, -135.7, -140.1, -144.5, -148.9, -153.3, -157.7.

$[\alpha]^{25}_D$ = -12 (c = 1.00, $CHCl_3$).

IR (ATR neat) ṽ =: 3069, 2983, 2929, 2922, 1643, 1436, 1317, 1207, 877, 753, 556 cm^{-1}.

HRMS (ESI): *m*/*z* calculated for $C_{27}H_{30}N_3^+$ [M - PF_6^-] 396.2434; found 396.2446.

(1*R*,5*S*)-4-(2-fluorophenyl)-1,8,8-trimethyl-2-(pyridin-2-ylmethyl)-2,4-diazabicyclo[3.2.1]oct-2-en-2-ium Hexafluorophosphate (3be)

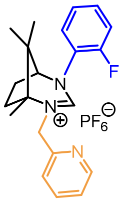

$C_{21}H_{25}F_7N_3P$
483,41 g/mol

Following **G.P. A-3**, a 50 mL Schlenk flask was charged with **2h** (500 mg, 2.12 mmol, 1 eq.), methyl orthoformate (1.15 mL, 10.58 mmol, 5 eq.), and acetic acid (0.59 mL, 10.58 mmol, 5 eq.) in 10 mL of dry ACN. The reaction mixture was stirred for 16 hours at 90 °C. After an acid-base work-up, the crude product was used in the next step without further purification. In the second step, the crude product was dissolved in 10 mL of dry ACN, followed by the addition of 2-(Bromomethyl)pyridine (1.8 g, 10.58 mmol, 5 eq.). The reaction mixture was stirred for 16 hours at 90 °C under nitrogen. 2-(Bromomethyl)pyridine is used as an HBr salt and needs to be extracted with $NaHCO_3$ to obtain pure bromide. The yield of extraction is usually 80%. The resulting solution of pure 2-(bromomethyl)pyridine is lacrimator. After the work-up, the crude product was redissolved in DCM (10 mL) and water (10 mL), followed by the addition of $NaPF_6$ (890 mg, 5.29 mmol, 2.5 eq.). The reaction mixture was stirred for 16 hours at room temperature. Column chromatography (SiO_2, DCM/petroleum ether/EtOAc 7:2:1, followed by 7:1:2, then DCM/EtOAc 7:3, v/v) of the crude mixture yielded **3be** (655 mg, 64%) as a light yellow solid.

mp 155 °C

Rf 0.55 (SiO_2, DCM/MeOH 9:1, v/v).

^{1}H NMR (600 MHz, CD_3CN) δ 8.63 (d, *J* = 4.1 Hz, 1H), 8.18 (s, 1H), 7.82 (td, *J* = 7.6, 1.7 Hz, 1H), 7.55 – 7.46 (m, 2H), 7.44 (d, *J* = 7.7 Hz, 1H), 7.40 – 7.33 (m, 3H), 4.85 (d, *J* = 16.3 Hz, 1H), 4.79 (d, *J* = 16.3 Hz,

1H), 3.81 (d, J = 5.2 Hz, 1H), 2.41 – 2.32 (m, 2H), 2.25 – 2.16 (m, 1H), 2.00 – 1.91 (m, 1H), 1.31 (s, 3H), 1.20 (s, 3H), 1.13 (s, 3H).

^{13}C{^{1}H} NMR (151 MHz, CD_3CN) δ 157.5, 156.4, 155.8, 154.5, 150.4, 138.2, 131.9, 131.8, 128.9, 128.8, 127.6, 126.4, 126.4, 124.3, 123.2, 117.9, 117.8, 73.3, 71.9, 55.0, 42.2, 40.2, 32.1, 21.3, 16.8, 14.4, 1.3, 1.2, 1.0, 0.9, 0.8, 0.6, 0.5.

^{15}N NMR (61 MHz, CD_3CN) δ -245.8, -231.1, -70.2

^{19}F NMR (377 MHz, CD_3CN) δ -70.6, -72.4, -122.1.

^{31}P NMR (162 MHz, CD_3CN) δ -131.3, -135.7, -140.1, -144.5, -148.9, -153.3, -157.7.

[α]$^{25}_{D}$ = +6 (c = 1.00, $CHCl_3$).

IR (ATR neat) ṽ =: 3075, 2997, 2978, 1647, 1503, 1449, 1379, 1209, 828, 750, 670 cm^{-1}.

HRMS (ESI): *m/z* calculated for $C_{21}H_{25}FN_3^+$ [M - PF_6^-] 338.2027; found 338.2037.

(1*R*,5*S*)-1,8,8-trimethyl-4-phenyl-2-(pyridin-2-ylmethyl)-2,4-diazabicyclo[3.2.1]oct-2-en-2-ium Hexafluorophosphate (3bf)

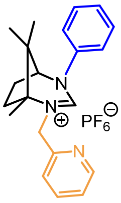

$C_{21}H_{26}F_6N_3P$
465,42 g/mol

Following **G.P. A-3**, a 50 mL Schlenk flask was charged with **2a** (800 mg, 3.66 mmol, 1 eq.), methyl orthoformate (2 mL, 18.32 mmol, 5 eq.), and acetic acid (1.05 mL, 18.32 mmol, 5 eq.) in 10 mL of dry ACN. The reaction mixture was stirred for 16 hours at 90 °C. After an acid-base work-up, the crude product was used in the next step without further purification. In the second step, the crude product was dissolved in 10 mL of dry ACN, followed by the addition of 2-(Bromomethyl)pyridine (1.89 g, 10.99 mmol, 3 eq.). The reaction mixture was stirred for 16 hours at 90 °C under nitrogen. 2-(Bromomethyl)pyridine is used as an HBr salt and needs to be extracted with $NaHCO_3$ to obtain pure bromide. The yield of extraction is usually 80%. The resulting solution of pure 2-(bromomethyl)pyridine is lacrimator. After the work-up, the crude product was redissolved in DCM (10 mL) and water (10 mL), followed by the addition of $NaPF_6$ (1.54 g, 9.16 mmol, 2.5 eq.). The reaction mixture was stirred for 16 hours at room temperature. Column chromatography (SiO_2, DCM/petroleum ether/EtOAc 7:2:1, followed by DCM/Et_2O 7:3, v/v) of the crude mixture yielded **3bf** (1.44 g, 84%) as a light yellow solid.

mp 162 °C

Rf 0.20 (SiO_2, DCM/petroleum ether/EtOAc 7:2:1, v/v).

^{1}H NMR (600 MHz, $CDCl_3$) δ 8.59 (d, J = 4.1 Hz, 1H), 8.11 (s, 1H), 7.75 (td, J = 7.7, 1.7 Hz, 1H), 7.52 – 7.44 (m, 2H), 7.43 – 7.39 (m, 3H), 7.39 – 7.36 (m, 1H), 7.31 (dd, J = 7.2, 5.1 Hz, 1H), 4.91 (d, J = 16.4 Hz, 1H), 4.84 (d, J = 16.4 Hz, 1H), 3.88 (d, J = 5.1 Hz, 1H), 2.48 (ddd, J = 13.8, 9.4, 4.3 Hz, 1H), 2.39 (ddd, J = 20.4, 12.6, 3.6 Hz, 1H), 2.27 (ddt, J = 12.1, 9.7, 4.8 Hz, 1H), 1.93 (ddd, J = 14.0, 12.1, 4.7 Hz, 1H), 1.35 (s, 3H), 1.18 (s, 3H), 1.18 (s, 3H).

^{13}C{^{1}H} NMR (151 MHz, $CDCl_3$) δ 153.8, 153.6, 149.9, 140.6, 137.7, 130.6, 130.5, 128.8, 123.8, 122.7, 122.5, 122.2, 72.5, 70.9, 54.5, 41.5, 40.0, 32.0, 21.7, 16.9, 14.4.

^{15}N NMR (61 MHz, $CDCl_3$) δ -230.6, -69.7.

^{19}F NMR (377 MHz, $CDCl_3$) δ -71.9, -73.8.

^{31}P NMR (162 MHz, $CDCl_3$) δ -131.3, -135.7, -140.1, -144.5, -148.9, -153.3, -157.7.

[α]$^{25}_{D}$ = +30 (c = 1.00, $CHCl_3$).

IR (ATR neat) ṽ =: 3078, 2991, 1646, 1592, 1572, 1495, 1434, 764, 554 cm^{-1}.

HRMS (ESI): *m/z* calculated for $C_{21}H_{26}N_3^+$ [M - PF_6^-] 320.2121; found 320.2128.

(1*R*,5*S*)-4-mesityl-1,8,8-trimethyl-2-(pyridin-2-ylmethyl)-2,4-diazabicyclo[3.2.1]oct-2-en-2-ium Hexafluorophosphate (3bg)

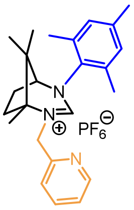

$C_{24}H_{32}F_6N_3P$
507,51 g/mol

Following **G.P. A-3**, a 50 mL Schlenk flask was charged with **2l** (500 mg, 1.85 mmol, 1 eq.), methyl orthoformate (1.01 mL, 9.25 mmol, 5 eq.), and acetic acid (0.51 mL, 9.25 mmol, 5 eq.) in 10 mL of dry ACN. The reaction mixture was stirred for 16 hours at 90 °C. After an acid-base work-up, the crude product was used in the next step without further purification. In the second step, the crude product was dissolved in 10 mL of dry ACN, followed by the addition of 2-(Bromomethyl)pyridine (1.58 g, 9.25 mmol, 5 eq.). The reaction mixture was stirred for 16 hours at 90 °C under nitrogen. 2-(Bromomethyl)pyridine is used as an HBr salt and needs to be extracted with $NaHCO_3$ to obtain pure bromide. The yield of extraction is usually 80%. The resulting solution of pure 2-(bromomethyl)pyridine is lacrimator. After the work-up, the crude product was redissolved in DCM (10 mL) and water (10 mL), followed by the addition of $NaPF_6$ (780 mg, 4.62 mmol, 2.5 eq.). The reaction mixture was stirred for 16 hours at room temperature. Column chromatography (SiO_2, DCM/petroleum ether/EtOAc 7:2:1, v/v) of the crude mixture yielded **3bg** (530 mg, 56%) as a light yellow solid.

mp 205 °C

Rf 0.51 (SiO_2, DCM/MeOH 9:1, v/v).

^{1}H NMR (600 MHz, $CDCl_3$) δ 8.57 (ddd, *J* = 4.7, 1.6, 0.8 Hz, 1H), 7.79 (s, 1H), 7.74 (td, *J* = 7.6, 1.8 Hz, 1H), 7.37 (d, *J* = 7.7 Hz, 1H), 7.32 – 7.27 (m, 1H), 6.98 (s, 1H), 6.94 (s, 1H), 4.89 (d, *J* = 16.5 Hz, 1H), 4.77 (d, *J* = 16.5 Hz, 1H), 3.51 (d, *J* = 4.3 Hz, 1H), 2.60 (ddd, *J* = 15.2, 9.5, 6.0 Hz, 1H), 2.51 (s, 3H), 2.36 – 2.26 (m, 7H), 2.08 – 1.99 (m, 1H), 1.89 (ddd, *J* = 15.2, 12.2, 3.4 Hz, 1H), 1.42 (s, 3H), 1.38 (s, 3H), 1.16 (s, 3H).

^{13}C{^{1}H} NMR (151 MHz, $CDCl_3$) δ 156.5, 153.8, 149.8, 139.9, 137.8, 135.1, 135.0, 134.7, 131.2, 130.7, 123.9, 122.9, 71.1, 55.1, 42.5, 39.4, 31.7, 22.1, 21.0, 19.2, 18.4, 18.0, 15.1.

^{15}N NMR (61 MHz, $CDCl_3$) δ -67.0, -235.3.

^{19}F NMR (377 MHz, $CDCl_3$) δ -71.9, -73.8.

^{31}P NMR (162 MHz, $CDCl_3$) δ -131.3, -135.7, -140.1, -144.5, -148.9, -153.3, -157.7.

$[\alpha]^{25}_D$ = -243 (c = 1.00, DCM).

IR (ATR neat) ṽ =: 2961, 1644, 1592, 1448, 1402, 1367, 1317, 1212, 739, 555 cm^{-1}.

HRMS (ESI): *m*/*z* calculated for $C_{24}H_{32}N_3^+$ [M - PF_6^-] 362.2591; found 362.2593.

(1*R*,5*S*)-1,8,8-trimethyl-4-(naphthalen-1-yl)-2-(pyridin-2-ylmethyl)-2,4-diazabicyclo[3.2.1]oct-2-en-2-ium Hexafluorophosphate (3bh)

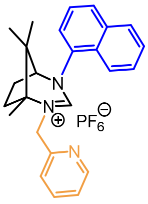

$C_{25}H_{28}F_6N_3P$
515,48 g/mol

Following **G.P. A-3**, a 50 mL Schlenk flask was charged with **2m** (400 mg, 1.49 mmol, 1 eq.), methyl orthoformate (0.82 mL, 7.45 mmol, 5 eq.), and acetic acid (0.43 mL, 7.45 mmol, 5 eq.) in 10 mL of dry ACN. The reaction mixture was stirred for 16 hours at 90 °C. After an acid-base work-up, the crude product was used in the next step without further purification. In the second step, the crude product was dissolved in 10 mL of dry ACN, followed by the addition of 2-(Bromomethyl)pyridine (1.27 g, 7.45 mmol, 5 eq.). The reaction mixture was stirred for 16 hours at 90 °C under nitrogen. 2-(Bromomethyl)pyridine is used as an HBr salt and needs to be extracted with $NaHCO_3$ to obtain pure bromide. The yield of extraction is usually 80%. The resulting solution of pure 2-(bromomethyl)pyridine is lacrimator. After the work-up, the crude product was redissolved in DCM (10 mL) and water (10 mL), followed by the addition of $NaPF_6$ (630 mg, 3.73 mmol, 2.5 eq.). The reaction mixture was stirred for 16 hours at room temperature. Column chromatography (SiO_2, DCM/petroleum ether/EtOAc 7:2:1, followed by 7:1:2, v/v) of the crude mixture yielded **3bh** (550 mg, 71%) as a light yellow solid.

mp 125 °C

Rf 0.62 (SiO_2, DCM/MeOH 9:1, v/v).

^{1}H NMR (600 MHz, DMSO) δ 8.85 – 8.80 (m, 2H), 8.29 (s, 1H), 8.15 (d, *J* = 8.0 Hz, 2H), 7.94 (td, *J* = 7.6, 1.7 Hz, 1H), 7.88 (t, *J* = 7.5 Hz, 1H), 7.77 – 7.69 (m, 3H), 7.62 (d, *J* = 7.7 Hz, 1H), 7.49 (dd, *J* = 6.9, 4.9 Hz, 1H), 5.09 – 4.93 (m, 2H), 4.05 (d, *J* = 4.8 Hz, 1H), 2.51 – 2.47 (m, 1H), 2.44 (td, *J* = 11.2, 9.1, 3.4 Hz, 1H), 2.22

(ddt, J = 14.4, 12.3, 4.8 Hz, 1H), 2.03 (ddd, J = 13.7, 12.2, 4.2 Hz, 1H), 1.45 (s, 3H), 1.42 (s, 3H), 1.22 (s, 3H).

^{13}C{^{1}H} NMR (151 MHz, DMSO) δ 156.5, 154.1, 149.2, 137.2, 133.9, 129.4, 128.4, 127.7, 126.7, 125.3, 123.2, 122.2, 121.1, 71.5, 53.5, 39.0, 31.2, 20.6, 16.9, 13.7.

^{15}N NMR (61 MHz, DMSO) δ -129.5, -243.9.

^{19}F NMR (377 MHz, DMSO) δ -70.9, -72.8.

^{31}P NMR (162 MHz, DMSO) δ -130.3, -134.7, -139.1, -143.5, -147.9, -152.3, -156.7.

$[\alpha]^{25}_{D}$ = -98 (c = 1.00, $CHCl_3$).

IR (ATR neat) ṽ =: 3065, 2984, 1643, 1593, 1471, 1438, 1397, 1374, 1321, 772, 556 cm^{-1}.

HRMS (ESI): *m*/*z* calculated for $C_{25}H_{28}N_3^+$ [M - PF_6^-] 370.2278; found 370.2277.

(1*R*,5*S*)-1,8,8-trimethyl-4-(naphthalen-2-yl)-2-(pyridin-2-ylmethyl)-2,4-diazabicyclo[3.2.1]oct-2-en-2-ium Hexafluorophosphate (3bi)

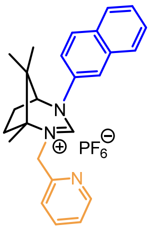

$C_{25}H_{28}F_6N_3P$
515,48 g/mol

Following **G.P. A-3**, a 50 mL Schlenk flask was charged with **2n** (500 mg, 1.86 mmol, 1 eq.), methyl orthoformate (1.02 mL, 9.31 mmol, 5 eq.), and acetic acid (0.53 mL, 9.31 mmol, 5 eq.) in 10 mL of dry ACN. The reaction mixture was stirred for 16 hours at 90 °C. After an acid-base work-up, the crude product was used in the next step without further purification. In the second step, the crude product was dissolved in 10 mL of dry ACN, followed by the addition of 2-(Bromomethyl)pyridine (1.6 g, 9.31 mmol, 5 eq.). The reaction mixture was stirred for 16 hours at 90 °C under nitrogen. 2-(Bromomethyl)pyridine is used as an HBr salt and needs to be extracted with $NaHCO_3$ to obtain pure bromide. The yield of extraction is usually 80%. The resulting solution of pure 2-(bromomethyl)pyridine is lacrimator. After the work-up, the crude product was redissolved in DCM (10 mL) and water (10 mL), followed by the addition of $NaPF_6$ (790 mg, 4,66 mmol, 2.5 eq.). The reaction mixture was stirred for 16 hours at room temperature. Column chromatography (SiO_2, DCM/petroleum ether/EtOAc 7:2:1, followed by 7:1:2, v/v) of the crude mixture yielded **3bi** (400 mg, 43%) as a light yellow solid.

mp 120 °C

Rf 0.5 (SiO_2, DCM/MeOH 9:1, v/v).

^{1}H NMR (600 MHz, $CDCl_3$) δ 9.16 (s, 1H), 8.49 (ddd, J = 4.8, 1.7, 0.8 Hz, 1H), 8.31 (d, J = 2.3 Hz, 1H), 7.98 – 7.93 (m, 1H), 7.87 (d, J = 8.8 Hz, 1H), 7.79 – 7.75 (m, 1H), 7.68 – 7.62 (m, 2H), 7.46 (qdp, J = 5.0, 5.0, 3.9, 2.0, 1.9, 1.9, 1.8 Hz, 2H), 7.42 (d, J = 7.7 Hz, 1H), 7.18 (ddd, J = 7.5, 4.8, 0.9 Hz, 1H), 5.49 (d, J = 16.4 Hz, 1H), 5.17 (d, J = 16.4 Hz, 1H), 3.91 (d, J = 5.2 Hz, 1H), 2.66 (ddd, J = 14.0, 9.5, 4.4 Hz, 1H), 2.51 (ddd, J = 14.3, 9.5, 4.8 Hz, 1H), 2.28 – 2.21 (m, 1H), 1.91 – 1.84 (m, 1H), 1.32 (s, 3H), 1.24 (s, 3H), 1.15 (s, 3H).

^{13}C{^{1}H} NMR (151 MHz, $CDCl_3$) δ 154.8, 154.6, 149.6, 137.8, 137.3, 133.4, 132.4, 130.4, 128.5, 127.6, 127.4, 127.0, 123.4, 122.8, 121.1, 120.0, 72.0, 70.5, 55.0, 41.8, 40.0, 32.0, 22.0, 17.4, 14.8.

^{15}N NMR (61 MHz, $CDCl_3$) δ -230.4, -65.5.

^{19}F NMR (377 MHz, $CDCl_3$) δ -71.9, -73.8.

^{31}P NMR (162 MHz, $CDCl_3$) δ -131.3, -135.7, -140.1, -144.5, -148.9, -153.3, -157.7.

$[\alpha]^{25}_{D}$ = +10 (c = 1.00, $CHCl_3$).

IR (ATR neat) ṽ =: 3043, 2951, 2874, 1641, 1625, 1593, 1570, 1469, 723 cm^{-1}.

HRMS (ESI): *m*/*z* calculated for $C_{25}H_{28}N_3^+$ [M - PF_6^-] 370.2278; found 370.2283.

(1*R*,5*S*)-1,8,8-trimethyl-4-(perfluorophenyl)-2-(pyridin-2-ylmethyl)-2,4-diazabicyclo[3.2.1]oct-2-en-2-ium bromide (3bj)

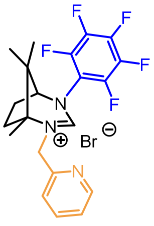

$C_{21}H_{21}BrF_5N_3$
490,3160 g/mol

Following **G.P. A-3**, a 50 mL Schlenk flask was charged with **2k** (400 g, 1.29 mmol, 1 eq.), methyl orthoformate (0.71 mL, 6.49 mmol, 5 eq.), and acetic acid (0.37 mL, 6.49 mmol, 5 eq.) in 10 mL of dry ACN. The reaction mixture was stirred for 16 hours at 90 °C. After an acid-base work-up, the crude product was used in the next step without further purification. In the second step, the crude product was dissolved in 10 mL of dry ACN, followed by the addition of 2-(Bromomethyl)pyridine (1.12 g, 6.49 mmol, 5 eq.). The reaction mixture was stirred for 16 hours at 90 °C under nitrogen. 2-(Bromomethyl)pyridine is used as an HBr salt and needs to be extracted with $NaHCO_3$ to obtain pure bromide. The yield of extraction is usually 80%. The resulting solution of pure 2-(bromomethyl)pyridine is lacrimator. The reaction mixture was stirred for 16 hours at room temperature. Column chromatography (SiO_2, DCM/MeOH 95:5, v/v) of the crude mixture yielded **3bj** (150 mg, 24%) as a light yellow oil.

mp 54 °C

Rf 0.38 (SiO_2, DCM/MeOH 9:1, v/v).

^{1}H NMR (600 MHz, $CDCl_3$) δ 10.11 (s, 1H), 8.77 (ddd, *J* = 4.8, 1.7, 0.8 Hz, 1H), 7.94 (td, *J* = 7.7, 1.8 Hz, 1H), 7.79 (d, *J* = 7.7 Hz, 1H), 7.49 (ddd, *J* = 7.5, 4.8, 0.9 Hz, 1H), 5.55 (d, *J* = 15.7 Hz, 1H), 5.19 (d, *J* = 15.7 Hz, 1H), 3.82 (d, *J* = 4.8 Hz, 1H), 2.60 (pd, *J* = 9.5, 3.4 Hz, 2H), 2.46 – 2.37 (m, 1H), 2.16 – 2.08 (m, 1H), 1.63 (s, 3H), 1.48 (s, 3H), 1.37 (s, 3H).

^{13}C{^{1}H} NMR (151 MHz, $CDCl_3$) δ 157.6, 153.3, 149.5, 144.1 (dd, *J* = 11.6, 3.1 Hz), 142.7 (t, *J* = 12.2 Hz), 142.4 (dd, *J* = 11.6, 2.4 Hz), 141.0 (td, *J* = 12.6, 3.9 Hz), 138.8 (td, *J* = 12.8, 11.2, 3.0 Hz), 137.4, 137.1 (td, *J* = 12.4, 4.4 Hz), 123.5, 122.7, 114.9 (td, *J* = 14.4, 14.0, 4.2 Hz), 72.6, 71.8, 54.9, 42.0, 39.8, 31.7, 21.7, 16.8, 14.5.

^{15}N NMR (61 MHz, $CDCl_3$) δ -66.4, -225.1, -260.3.

^{19}F NMR (377 MHz, $CDCl_3$) δ -145.52 – -145.68 (m), -151.54 (t, *J* = 21.7 Hz), -159.58 – -159.80 (m).

$[\alpha]^{25}_D$ = -35 (c = 1.00, $CHCl_3$).

IR (ATR neat) ṽ =: 2952, 1641, 1516, 1383, 1240, 1038, 993, 764, 558 cm^{-1}.

HRMS (ESI): *m*/*z* calculated for $C_{21}H_{21}N_3F_5^+$ [M - Br$^-$] 410.1650; found 410.1653.

(1*R*,5*S*)-4-(2-fluoro-6-methylphenyl)-1,8,8-trimethyl-2-(pyridin-2-ylmethyl)-2,4-diazabicyclo[3.2.1]oct-2-en-2-ium Hexafluorophosphate (3bk)

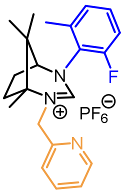

$C_{22}H_{27}F_7N_3P$
497,44 g/mol

Following **G.P. A-3**, a 50 mL Schlenk flask was charged with **2i** (500 mg, 1.997 mmol, 1 eq.), methyl orthoformate (1.1 mL, 9.99 mmol, 5 eq.), and acetic acid (0.57 mL, 9.99 mmol, 5 eq.) in 10 mL of dry ACN. The reaction mixture was stirred for 16 hours at 90 °C. After an acid-base work-up, the crude product was used in the next step without further purification. In the second step, the crude product was dissolved in 10 mL of dry ACN, followed by the addition of 2-(Bromomethyl)pyridine (1.7 g, 9.99 mmol, 5 eq.). The reaction mixture was stirred for 16 hours at 90 °C under nitrogen. 2-(Bromomethyl)pyridine is used as an HBr salt and needs to be extracted with $NaHCO_3$ to obtain pure bromide. The yield of extraction is usually 80%. The resulting solution of pure 2-(bromomethyl)pyridine is lacrimator. After the work-up, the crude product was redissolved in DCM (10 mL) and water (10 mL), followed by the addition of $NaPF_6$ (840 mg, 4.99 mmol, 2.5 eq.). The reaction mixture was stirred for 16 hours at room temperature. Column chromatography (SiO_2, DCM/petroleum ether/EtOAc 7:2:1, followed by 7:1:2, v/v) of the crude mixture yielded **3bk** (500 mg, 50%) as a light yellow solid. Mixture of rotamers ca. 6:4, not assigned.

mp 197 °C

Rf 0.55 (SiO_2, DCM/MeOH 9:1, v/v).

^{1}H NMR (600 MHz, CD_2Cl_2) δ 8.68 (d, *J* = 3.5 Hz, 1H), 7.98 (d, *J* = 10.9 Hz, 1H), 7.85 (dq, 1H), 7.47 – 7.37 (m, 3H), 7.32 – 7.08 (m, 2H), 4.97 – 4.73 (m, 2H), 3.75 – 3.53 (m, 1H), 2.69 (s, 2H), 2.60 – 2.43 (m, 2H), 2.43 – 2.32 (m, 1H), 2.17 (dtdd, *J* = 21.6, 14.4, 8.1, 4.7 Hz, 1H), 2.05 – 1.89 (m, 1H), 1.51 – 1.45 (m, 3H), 1.41 – 1.34 (m, 3H), 1.22 (s, 3H).

$^{13}C\{^1H\}$ NMR (151 MHz, CD_2Cl_2) δ 158.2, 157.1, 156.5, 155.9, 153.1, 152.9, 150.0, 149.9, 137.9, 137.8, 137.3, 137.0, 131.3, 131.3, 131.1, 131.0, 127.8, 127.6, 127.2, 127.1, 126.2, 124.1, 124.0, 122.7, 114.7, 114.6, 114.5, 114.4, 72.4, 72.1, 71.9, 71.0, 55.0, 54.9, 42.3, 42.0, 40.1, 39.5, 31.9, 31.6, 21.8, 21.5, 17.9, 17.7, 17.6, 16.8, 16.7, 14.8, 14.5.

^{15}N NMR (61 MHz, CD_2Cl_2) δ -246.6, -232.9, -69.3.

^{19}F NMR (377 MHz, CD_2Cl_2) δ -71.9, -73.8, -118.8, -123.9.

^{31}P NMR (162 MHz, CD_2Cl_2) δ -131.3, -135.7, -140.1, -144.5, -148.9, -153.3, -157.7.

$[\alpha]^{25}_D$ = -173 (c = 1.00, ACN).

IR (ATR neat) ṽ =: 2981, 1648, 1594, 1475, 1440, 1403, 1376, 1319, 1297, 740, 556 cm^{-1}.

HRMS (ESI): *m*/*z* calculated for $C_{22}H_{27}FN_3^+$ [M - PF_6^-] 352.2184; found 352.2193.

(1*R*,5*S*)-4-mesityl-1,8,8-trimethyl-2-((*S*)-1-(pyridin-2-yl)ethyl)-2,4-diazabicyclo[3.2.1]oct-2-en-2-ium Hexafluorophosphate (3bl)

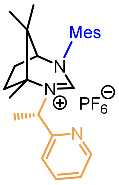

$C_{25}H_{34}F_6N_3P$
521,53 g/mol

Following **G.P. A-3**, a 50 mL Schlenk flask was charged with **2l** (700 mg, 2.59 mmol, 1 eq.), methyl orthoformate (1.42 mL, 12.95 mmol, 5 eq.), and acetic acid (0.74 mL, 12.95 mmol, 5 eq.) in 10 mL of dry ACN. The reaction mixture was stirred for 16 hours at 90 °C. After an acid-base work-up, the crude product was used in the next step without further purification. In a parallel flask, (*R*)-1-(pyridin-2-yl)ethyl methanesulfonate was synthesized by stirring (*R*)-1-(pyridin-2-yl)ethan-1-ol (960 mg, 7.77 mmol, 3 eq.) with triethylamine (0.66 mL, 8.55 mmol, 3.3 eq.) and methanesulfonyl chloride (1.2 mL, 8.55 mmol, 3.3 eq.) in 20 mL of dry DCM for 2 hours at room temperature. Upon completion of the reaction, the mixture was subjected to extraction DCM/water. The combined organic fractions were then dried over anhydrous $MgSO_4$, passed through a cotton plug for filtration, and concentrated under reduced pressure to yield the final product. The resulting (*R*)-1-(pyridin-2-yl)ethyl methanesulfonate was utilized in the subsequent step without further purification. In the second step, the crude cyclic product **2l** was dissolved in 10 mL of dry ACN, followed by the addition of (*R*)-1-(pyridin-2-yl)ethyl methanesulfonate**.** The reaction mixture was stirred for 16 hours at 90 °C under nitrogen. After the work-up, the crude product was redissolved in DCM (10 mL) and water (10 mL), followed by the addition of $NaPF_6$ (1.09 g, 6.47 mmol, 2.5 eq.). The reaction mixture was stirred for 16 hours at room temperature. Column chromatography (SiO_2, DCM/petroleum ether/EtOAc 7:2:1, followed by 7:1:2, v/v) of the crude mixture yielded **3bl** (960 mg, 71%) as a light yellow solid.

mp 106 °C

Rf 0.22 (SiO_2, DCM/petroleum ether/EtOAc 7:2:1, v/v).

^1H NMR (600 MHz, $CDCl_3$) δ 8.50 – 8.44 (m, 1H), 8.03 (s, 1H), 7.73 (td, *J* = 7.7, 1.8 Hz, 1H), 7.41 (d, *J* = 7.8 Hz, 1H), 7.25 (ddd, *J* = 7.6, 4.9, 1.1 Hz, 1H), 7.05 (s, 1H), 7.01 (s, 1H), 5.35 (q, *J* = 6.9 Hz, 1H), 3.55 (d, *J* = 4.2 Hz, 1H), 2.73 (ddd, *J* = 15.0, 9.4, 5.7 Hz, 1H), 2.38 (s, 3H), 2.32 (s, 3H), 2.30 (s, 3H), 2.23 – 2.18 (m, 2H), 2.15 – 2.08 (m, 1H), 1.73 (d, *J* = 7.0 Hz, 3H), 1.49 (s, 3H), 1.12 (s, 3H), 0.88 (s, 3H).

$^{13}C\{^1H\}$ NMR (151 MHz, $CDCl_3$) δ 156.9, 154.1, 149.9, 140.2, 138.1, 135.4, 134.5, 134.4, 131.5, 130.8, 129.9, 123.8, 122.5, 73.2, 70.5, 57.0, 42.4, 41.0, 31.8, 24.3, 22.3, 20.9, 19.2, 19.1, 18.5, 17.7, 14.3.

^{15}N NMR (61 MHz, $CDCl_3$) δ -237.6, -220.9.

^{19}F NMR (377 MHz, $CDCl_3$) δ -71.9, -73.8.

^{31}P NMR (162 MHz, $CDCl_3$) δ -131.3, -135.7, -140.1, -144.5, -148.9, -153.3, -157.7.

$[\alpha]^{25}_D$ = -181 (c = 1.00, ACN).

IR (ATR neat) ṽ =: 2978, 1641, 1473, 1454, 1402, 1384, 1366, 753, 555 cm^{-1}.

HRMS (ESI): *m*/*z* calculated for $C_{25}H_{34}N_3^+$ [M - PF_6^-] 376.2747; found 376.2739.

(1*R*,5*S*)-4-mesityl-1,8,8-trimethyl-2-((*R*)-1-(pyridin-2-yl)ethyl)-2,4-diazabicyclo[3.2.1]oct-2-en-2-ium Hexafluorophosphate (3bm)

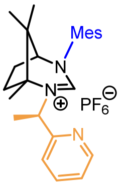

$C_{25}H_{34}F_6N_3P$
521,53 g/mol

Following **G.P. A-3**, a 50 mL Schlenk flask was charged with **2l** (700 mg, 2.59 mmol, 1 eq.), methyl orthoformate (1.42 mL, 12.95 mmol, 5 eq.), and acetic acid (0.74 mL, 12.95 mmol, 5 eq.) in 10 mL of dry ACN. The reaction mixture was stirred for 16 hours at 90 °C. After an acid-base work-up, the crude product was used in the next step without further purification. In a parallel flask, (*S*)-1-(pyridin-2-yl)ethyl methanesulfonate was synthesized by stirring (*S*)-1-(pyridin-2-yl)ethan-1-ol (960 mg, 7.77 mmol, 3 eq.) with triethylamine (0.66 mL, 8.55 mmol, 3.3 eq.) and methanesulfonyl chloride (1.2 mL, 8.55 mmol, 3.3 equiv.) in 20 mL of dry DCM for 2 hours at room temperature. Upon completion of the reaction, the mixture was subjected to extraction DCM/water. The combined organic fractions were then dried over anhydrous $MgSO_4$, passed through a cotton plug for filtration, and concentrated under reduced pressure to yield the final product. The resulting (*S*)-1-(pyridin-2-yl)ethyl methanesulfonate was utilized in the subsequent step without further purification. In the second step, the crude cyclic product **2l** was dissolved in 10 mL of dry ACN, followed by the addition of (*S*)-1-(pyridin-2-yl)ethyl methanesulfonate**.** The reaction mixture was stirred for 16 hours at 90 °C under nitrogen. After the work-up, the crude product was redissolved in DCM (10 mL) and water (10 mL), followed by the addition of $NaPF_6$ (1.09 g, 6.47 mmol, 2.5 eq.). The reaction mixture was stirred for 16 hours at room temperature. Column chromatography (SiO_2, DCM/petroleum ether/EtOAc 7:2:1, followed by 7:1:2, v/v) of the crude mixture yielded **3bm** (790 mg, 58%) as a light yellow solid.

mp 175 °C

Rf 0.65 (SiO_2, DCM/MeOH 9:1, v/v).

^{1}H NMR (600 MHz, $CDCl_3$) δ 8.57 (ddd, *J* = 3.9, 1.6, 1.1 Hz, 1H), 7.86 (s, 1H), 7.80 (td, *J* = 7.7, 1.7 Hz, 1H), 7.53 (d, *J* = 7.7 Hz, 1H), 7.35 (dd, *J* = 6.8, 4.8 Hz, 1H), 7.00 (s, 1H), 6.96 (s, 1H), 5.21 (q, *J* = 7.0 Hz, 1H), 3.56 (d, *J* = 4.1 Hz, 1H), 2.35 (s, 3H), 2.32 (s, 3H), 2.28 (d, *J* = 3.1 Hz, 3H), 2.25 – 2.15 (m, 2H), 2.04 (ddt, *J* = 17.9, 8.2, 5.0 Hz, 1H), 1.88 (s, 1H), 1.63 (d, *J* = 7.0 Hz, 3H), 1.56 (s, 3H), 1.39 (s, 3H), 1.20 (s, 3H).

^{13}C{^{1}H} NMR (151 MHz, $CDCl_3$) δ 157.2, 153.6, 153.5, 149.7, 140.1, 138.6, 135.3, 134.9, 134.5, 134.4, 131.3, 131.3, 130.8, 130.8, 124.2, 122.7, 72.4, 71.0, 57.7, 42.2, 39.2, 31.8, 22.2, 22.0, 20.9, 20.8, 19.2, 18.3, 17.8, 15.9, 15.1.

^{15}N NMR (61 MHz, $CDCl_3$) δ -236.7, -222.5.

^{19}F NMR (377 MHz, $CDCl_3$) δ -72.4, -74.3.

^{31}P NMR (162 MHz, $CDCl_3$) δ -131.3, -135.7, -140.1, -144.5, -148.9, -153.3, -157.7.

$[\alpha]^{25}_D$ = -224 (c = 1.00, ACN).

IR (ATR neat) ṽ =: 2983, 1637, 1474, 1454, 1318, 830, 754, 739, 584, 555 cm^{-1}.

HRMS (ESI): *m*/*z* calculated for $C_{25}H_{34}N_3^+$ [M - PF_6^-] 376.2747; found 376.2760.

(1*R*,5*S*)-1,8,8-trimethyl-2-((6-methylpyridin-2-yl)methyl)-4-(o-tolyl)-2,4-diazabicyclo[3.2.1]oct-2-en-2-ium Hexafluorophosphate (3bn)

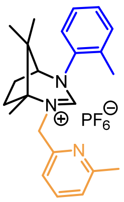

$C_{23}H_{30}F_6N_3P$
493,48 g/mol

Following **G.P. A-3**, a 50 mL Schlenk flask was charged with **2b** (350 mg, 1.44 mmol, 1 eq.), methyl orthoformate (0.79 mL, 7.2 mmol, 5 eq.), and acetic acid (0.41 mL, 7.2 mmol, 5 eq.) in 10 mL of dry ACN. The reaction mixture was stirred for 16 hours at 90 °C. After an acid-base work-up, the crude product was used in the next step without further purification. In the second step, the crude product was dissolved in 10 mL of dry ACN, followed by the addition of 2-(Bromomethyl)-6-methylpyridine (1.1 g, 5.78 mmol, 4 eq.). The reaction mixture was stirred for 16 hours at 90 °C under nitrogen. After the work-up, the crude product was redissolved in DCM (10 mL) and water (10 mL), followed by the addition of $NaPF_6$ (610 mg, 3.61 mmol, 2.5 eq.). The reaction mixture was stirred for 16 hours at room temperature. Column chromatography (SiO_2, DCM/petroleum ether/EtOAc 7:2:1, followed by 7:1:2, v/v) of the crude mixture yielded **3bn** (470 mg, 66%) as a light yellow solid.

mp 216 °C

Rf 0.10 (SiO_2, DCM/pentane/Et_2O 3:6:1, v/v).

^{1}H NMR (600 MHz, CD_2Cl_2) δ 7.92 (s, 1H), 7.69 (t, *J* = 7.7 Hz, 1H), 7.45 – 7.37 (m, 3H), 7.33 (d, *J* = 7.6 Hz, 1H), 7.22 (d, *J* = 7.7 Hz, 1H), 7.18 (d, *J* = 7.6 Hz, 1H), 4.75 (s, 2H), 3.66 (d, *J* = 5.0 Hz, 1H), 2.58 (s, 3H), 2.52 (s, 3H), 2.48 – 2.37 (m, 2H), 2.22 – 2.13 (m, 1H), 1.97 – 1.89 (m, 1H), 1.39 (s, 3H), 1.33 (s, 3H), 1.19 (s, 3H).

^{13}C{^{1}H} NMR (151 MHz, CD_2Cl_2) δ 159.2, 155.4, 152.2, 139.3, 138.1, 133.6, 132.5, 129.9, 127.9, 126.9, 123.6, 119.6, 72.1, 71.5, 54.5, 41.8, 39.7, 31.7, 24.1, 21.6, 18.3, 17.4, 14.5.

^{15}N NMR (61 MHz, CD_2Cl_2) δ -234.9, -72.3.

^{19}F NMR (377 MHz, CD_2Cl_2) δ -71.9, -73.8.

^{31}P NMR (162 MHz, CD_2Cl_2) δ -131.3, -135.7, -140.1, -144.5, -148.9, -153.3, -157.7.

$[\alpha]^{25}_D$ = -81 (c = 1.00, ACN).

IR (ATR neat) ṽ =: 3079, 2992, 1644, 1577, 1453, 1400, 1373, 872, 755 cm^{-1}.

HRMS (ESI): *m/z* calculated for $C_{23}H_{30}N_3^+$ [M - PF_6^-] 348.2440; found 348.2444.

(1*R*,5*S*)-4-(2-methoxyphenyl)-1,8,8-trimethyl-2-((6-methylpyridin-2-yl)methyl)-2,4-diazabicyclo[3.2.1]oct-2-en-2-ium Hexafluorophosphate (3bo)

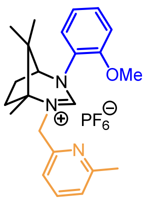

$C_{23}H_{30}F_6N_3OP$
509,48 g/mol

Following **G.P. A-3**, a 50 mL Schlenk flask was charged with **2d** (350 mg, 1.36 mmol, 1 eq.), methyl orthoformate (0.74 mL, 6.78 mmol, 5 eq.), and acetic acid (0.39 mL, 6.78 mmol, 5 eq.) in 10 mL of dry ACN. The reaction mixture was stirred for 16 hours at 90 °C. After an acid-base work-up, the crude product was used in the next step without further purification. In the second step, the crude product was dissolved in 10 mL of dry ACN, followed by the addition of 2-(Bromomethyl)-6-methylpyridine (1.0 g, 5.42 mmol, 4 eq.). The reaction mixture was stirred for 16 hours at 90 °C under nitrogen. After the work-up, the crude product was redissolved in DCM (10 mL) and water (10 mL), followed by the addition of $NaPF_6$ (570 mg, 3.39 mmol, 2.5 eq.). The reaction mixture was stirred for 16 hours at room temperature. Column chromatography (SiO_2, DCM/petroleum ether/EtOAc 7:2:1, followed by 7:1:2, v/v) of the crude mixture yielded **3bo** (600 mg, 87%) as a light yellow solid.

mp 165 °C

Rf 0.65 (SiO_2, DCM/MeOH 9:1, v/v).

^{1}H NMR (600 MHz, $CDCl_3$) δ 7.91 (s, 1H), 7.64 (t, *J* = 7.7 Hz, 1H), 7.42 (t, *J* = 7.9 Hz, 1H), 7.38 (d, *J* = 7.6 Hz, 1H), 7.20 – 7.14 (m, 2H), 7.07 (d, *J* = 8.0 Hz, 2H), 4.82 (dd, *J* = 16.7, 15.3 Hz, 2H), 3.94 (s, 3H), 3.68 (d, *J* = 5.2 Hz, 1H), 2.55 (s, 3H), 2.53 – 2.42 (m, 2H), 2.23 – 2.13 (m, 1H), 1.92 (td, *J* = 12.8, 11.3, 3.0 Hz, 1H), 1.36 (s, 3H), 1.26 (s, 3H), 1.17 (s, 3H).

^{13}C{^{1}H} NMR (151 MHz, $CDCl_3$) δ 158.7, 155.2, 153.3, 153.0, 137.9, 130.9, 128.9, 126.6, 123.2, 121.7, 119.6, 112.7, 72.3, 71.0, 56.0, 54.6, 41.7, 39.8, 31.8, 24.4, 21.9, 17.2, 14.6.

^{15}N NMR (61 MHz, $CDCl_3$) δ -234.8, -68.5.

^{19}F NMR (377 MHz, $CDCl_3$) δ -71.9, -73.8.

^{31}P NMR (162 MHz, $CDCl_3$) δ -131.3, -135.7, -140.1, -144.5, -148.9, -153.3, -157.7.

$[\alpha]^{25}_D$ = -14 (c = 1.00, ACN).

IR (ATR neat) ṽ =: 3112, 2988, 1650, 1502, 1448, 1379, 742, 555 cm^{-1}.

HRMS (ESI): *m/z* calculated for $C_{23}H_{30}N_3O^+$ [M - PF_6^-] 364.2383; found 364.2389.

(1*R*,5*S*)-4-([1,1'-biphenyl]-2-yl)-1,8,8-trimethyl-2-((6-methylpyridin-2-yl)methyl)-2,4-diazabicyclo[3.2.1]oct-2-en-2-ium Hexafluorophosphate (3bp)

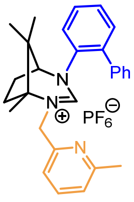

$C_{28}H_{32}F_6N_3P$
555,55 g/mol

Following **G.P. A-3**, a 50 mL Schlenk flask was charged with **2e** (350 mg, 1.15 mmol, 1 eq.), methyl orthoformate (0.63 mL, 5.75 mmol, 5 eq.), and acetic acid (0.33 mL, 5.75 mmol, 5 eq.) in 10 mL of dry ACN. The reaction mixture was stirred for 16 hours at 90 °C. After an acid-base work-up, the crude product was used in the next step without further purification. In the second step, the crude product was dissolved in 10 mL of dry ACN, followed by the addition of 2-(Bromomethyl)-6-methylpyridine (890 mg, 4.60 mmol, 4 eq.). The reaction mixture was stirred for 16 hours at 90 °C under nitrogen. After the work-up, the crude product was redissolved in DCM (10 mL) and water (10 mL), followed by the addition of $NaPF_6$ (480 mg, 2.87 mmol, 2.5 eq.). The reaction mixture was stirred for 16 hours at room temperature. Column chromatography (SiO_2, DCM/petroleum ether/EtOAc 7:2:1, followed by 7:1:2, v/v) of the crude mixture yielded **3bp** (500 mg, 78%) as a light yellow solid.

mp 140 °C

Rf 0.60 (SiO_2, DCM/MeOH 9:1, v/v).

^{1}H NMR (600 MHz, $CDCl_3$) δ 8.11 (s, 1H), 7.60 (t, *J* = 7.6 Hz, 1H), 7.55 (dd, *J* = 7.7, 1.5 Hz, 1H), 7.56 – 7.42 (m, 4H), 7.40 (dd, *J* = 7.4, 2.6 Hz, 1H), 7.37 – 7.31 (m, 3H), 7.15 (d, *J* = 7.7 Hz, 1H), 7.06 (d, *J* = 7.5 Hz, 1H), 4.81 (d, *J* = 16.4 Hz, 1H), 4.72 (d, *J* = 16.3 Hz, 1H), 3.20 (d, *J* = 4.1 Hz, 1H), 2.60 (s, 3H), 2.36 – 2.28 (m, 1H), 1.88 (q, *J* = 17.9, 14.9 Hz, 1H), 1.75 – 1.65 (m, 2H), 1.21 (s, 3H), 0.85 (s, 3H), 0.66 (s, 3H).

^{13}C{^{1}H} NMR (151 MHz, $CDCl_3$) δ 158.6, 155.7, 152.7, 138.6, 138.3, 138.0, 137.9, 132.4, 129.8, 129.7, 129.6, 129.0, 128.5, 128.2, 123.4, 119.6, 72.0, 71.4, 54.4, 41.9, 39.4, 30.4, 24.5, 21.8, 17.0, 14.5.

^{15}N NMR (61 MHz, $CDCl_3$) δ -231.9, -69.1.

^{19}F NMR (377 MHz, $CDCl_3$) δ -71.9, -73.8.

^{31}P NMR (162 MHz, $CDCl_3$) δ -131.3, -135.7, -140.1, -144.5, -148.9, -153.3, -157.7.

$[\alpha]^{25}_D$ = -45 (c = 1.00, ACN).

IR (ATR neat) ṽ =: 3071, 2986, 1641, 1575, 1480, 1452, 1372, 1209, 875, 754, 555 cm^{-1}.

HRMS (ESI): *m*/*z* calculated for $C_{28}H_{32}N_3^+$ [M - PF_6^-] 410.2591; found 410.2597.

(1*R*,5*S*)-4-mesityl-1,8,8-trimethyl-2-((6-methylpyridin-2-yl)methyl)-2,4-diazabicyclo[3.2.1]oct-2-en-2-ium Hexafluorophosphate (3br)

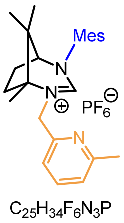

$C_{25}H_{34}F_6N_3P$
521,53 g/mol

Following **G.P. A-3**, a 50 mL Schlenk flask was charged with **2l** (500 mg, 1.85 mmol, 1 eq.), methyl orthoformate (1.01 mL, 9.25 mmol, 5 eq.), and acetic acid (0.53 mL, 9.25 mmol, 5 eq.) in 10 mL of dry ACN. The reaction mixture was stirred for 16 hours at 90 °C. After an acid-base work-up, the crude product was used in the next step without further purification. In the second step, the crude product was dissolved in 10 mL of dry ACN, followed by the addition of 2-(Bromomethyl)-6-methylpyridine (1.72 g, 9.24 mmol, 5 eq.). The reaction mixture was stirred for 16 hours at 90 °C under nitrogen. After the work-up, the crude product was redissolved in DCM (10 mL) and water (10 mL), followed by the addition of $NaPF_6$ (780 mg, 4.62 mmol, 2.5 eq.). The reaction mixture was stirred for 16 hours at room temperature. Column chromatography (SiO_2, petroleum ether/EtOAc 1:1, followed by DCM/petroleum ether/EtOAc 7:1:2, v/v) of the crude mixture yielded **3br** (650 mg, 67%) as a light yellow solid.

mp 227 °C

Rf 0.65 (SiO_2, DCM/MeOH 9:1, v/v).

^{1}H NMR (600 MHz, $CDCl_3$) δ 7.74 (s, 1H), 7.60 (t, *J* = 7.7 Hz, 1H), 7.13 (dd, *J* = 7.6, 4.6 Hz, 2H), 6.97 (s, 1H), 6.93 (s, 1H), 4.79 (d, *J* = 16.3 Hz, 1H), 4.70 (d, *J* = 16.3 Hz, 1H), 3.51 (d, *J* = 4.2 Hz, 1H), 2.62 (ddd, *J* = 15.2, 9.5, 6.0 Hz, 1H), 2.53 (s, 3H), 2.50 (s, 3H), 2.36 – 2.28 (m, 4H), 2.27 (s, 3H), 2.08 – 2.00 (m, 1H), 1.88 (ddd, *J* = 15.1, 11.7, 3.4 Hz, 1H), 1.39 (s, 3H), 1.35 (s, 3H), 1.15 (s, 3H).

^{13}C{^{1}H} NMR (151 MHz, $CDCl_3$) δ 158.9, 156.2, 152.9, 139.8, 137.9, 135.1, 134.8, 134.6, 131.2, 130.6, 123.4, 119.8, 71.0, 71.0, 55.5, 42.4, 39.4, 31.6, 24.2, 21.9, 20.9, 19.0, 18.8, 17.8, 14.9.

^{15}N NMR (61 MHz, $CDCl_3$) δ -235.3, -66.8.

^{19}F NMR (377 MHz, $CDCl_3$) δ -71.9, -73.8.

^{31}P NMR (162 MHz, $CDCl_3$) δ -131.3, -135.7, -140.1, -144.5, -148.9, -153.3, -157.7.

$[\alpha]^{25}_D$ = -213 (c = 1.00, ACN).

IR (ATR neat) ṽ =: 2980, 1644, 1594, 1575, 1453, 1402, 1370, 1317, 740, 556 cm^{-1}.

HRMS (ESI): *m*/*z* calculated for $C_{25}H_{34}N_3^+$ [M - PF_6^-] 376.2747; found 376.2751.

(1*R*,5*S*)-4-mesityl-1,8,8-trimethyl-2-((6-phenylpyridin-2-yl)methyl)-2,4-diazabicyclo[3.2.1]oct-2-en-2-ium Hexafluorophosphate (3bs)

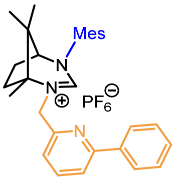

$C_{30}H_{36}F_6N_3P$
583,60 g/mol

Following **G.P. A-3**, a 50 mL Schlenk flask was charged with **2l** (300 mg, 1.11 mmol, 1 eq.), methyl orthoformate (0.61 mL, 5.55 mmol, 5 eq.), and acetic acid (0.32 mL, 5.55 mmol, 5 eq.) in 10 mL of dry ACN. The reaction mixture was stirred for 16 hours at 90 °C. After an acid-base work-up, the crude product was used in the next step without further purification. In the second step, the crude product was dissolved in 10 mL of dry ACN, followed by the addition of 2-(bromomethyl)-6-phenylpyridine[20] (690 mg, 2.77 mmol, 2.5 eq.). The reaction mixture was stirred for 16 hours at 90 °C under nitrogen. After the work-up, the crude product was redissolved in DCM (10 mL) and water (10 mL), followed by the addition of $NaPF_6$ (470 mg, 2.77 mmol, 2.5 eq.). The reaction mixture was stirred for 16 hours at room temperature. Column chromatography (SiO_2, petroleum ether/EtOAc 1:1, followed by DCM/EtOAc 7:3, v/v) of the crude mixture yielded **3bs** (350 mg, 54%) as a light yellow solid.

mp 194 °C

Rf 0.53 (SiO_2, DCM/MeOH 9:1, v/v).

^{1}H NMR (600 MHz, $CDCl_3$) δ 7.91 – 7.85 (m, 3H), 7.80 (t, *J* = 7.7 Hz, 1H), 7.70 (d, *J* = 7.4 Hz, 1H), 7.44 (qd, *J* = 4.4, 1.7 Hz, 3H), 7.32 (d, *J* = 7.3 Hz, 1H), 6.93 (s, 1H), 6.92 (s, 1H), 4.97 (d, *J* = 16.2 Hz, 1H), 4.82 (d, *J* = 16.2 Hz, 1H), 3.49 (d, *J* = 4.0 Hz, 1H), 2.71 (ddd, *J* = 14.8, 9.4, 5.1 Hz, 1H), 2.31 – 2.23 (m, 10H), 2.06 – 1.94 (m, 2H), 1.48 (s, 3H), 1.28 (s, 3H), 1.14 (s, 3H).

^{13}C{^{1}H} NMR (151 MHz, $CDCl_3$) δ 157.8, 156.1, 153.9, 139.9, 138.7, 135.0, 134.7, 134.5, 131.2, 130.6, 129.5, 128.8, 127.1, 121.6, 120.8, 71.5, 71.0, 55.4, 42.3, 39.9, 31.7, 22.1, 20.9, 19.0, 18.5, 17.9, 14.9.

^{15}N NMR (^{15}N NMR (61 MHz, $CDCl_3$) δ -234.2, -72.8.

^{19}F NMR (377 MHz, $CDCl_3$) δ -72.2, -74.1.

^{31}P NMR (162 MHz, $CDCl_3$) δ -131.3, -135.7, -140.1, -144.5, -148.9, -153.3, -157.7.

$[\alpha]^{25}_D$ = -253 (c = 1.00, ACN).

IR (ATR neat) ṽ =: 2982, 2963, 2926, 1730, 1643, 1578, 1448, 765, 555 cm^{-1}.

HRMS (ESI): *m*/*z* calculated for $C_{30}H_{36}N_3^+$ [M - PF_6^-] 438.2904; found 438.2913.

(1*R*,5*S*)-2-((6-(3,5-di-*tert*-butylphenyl)pyridin-2-yl)methyl)-4-mesityl-1,8,8-trimethyl-2,4-diazabicyclo[3.2.1]oct-2-en-2-ium Hexafluorophosphate (3bt)

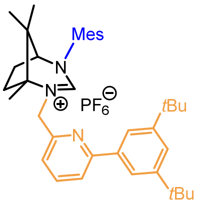

$C_{38}H_{52}F_6N_3P$
695,82 g/mol

Following **G.P. A-3**, a 50 mL Schlenk flask was charged with **2l** (270 mg, 1.0 mmol, 1 eq.), methyl orthoformate (0.55 mL, 5.0 mmol, 5 eq.), and acetic acid (0.29 mL, 5.0 mmol, 5 eq.) in 10 mL of dry ACN. The reaction mixture was stirred for 16 hours at 90 °C. After an acid-base work-up, the crude product was used in the next step without further purification. In the second step, the crude product was dissolved in 10 mL of dry ACN, followed by the addition of 2-(bromomethyl)-6-(3,5-di-*tert*-butylphenyl)pyridine[20] (470 mg, 1.3 mmol, 1.3 eq.). The reaction mixture was stirred for 16 hours at 90 °C under nitrogen. After the work-up, the crude product was redissolved in DCM (10 mL) and water (10 mL), followed by the addition of $NaPF_6$ (420 mg, 2.5 mmol, 2.5 eq.). The reaction mixture was stirred for 16 hours at room temperature. Column chromatography (SiO_2, DCM) of the crude mixture yielded **3bt** (290 mg, 42%) as a light yellow solid.

mp 171 °C

Rf 0.75 (SiO_2, DCM/EtOAc 9:1, v/v).

^{1}H NMR (600 MHz, $CDCl_3$) δ 7.84 – 7.77 (m, 2H), 7.72 – 7.67 (m, 3H), 7.54 (t, *J* = 1.7 Hz, 1H), 7.37 (d, *J* = 7.4 Hz, 1H), 6.89 (s, 1H), 6.82 (s, 1H), 4.99 (d, *J* = 15.9 Hz, 1H), 4.87 (d, *J* = 15.9 Hz, 1H), 3.52 (d, *J* = 2.1 Hz, 1H), 2.87 – 2.81 (m, 1H), 2.26 – 2.22 (m, 7H), 2.08 (dd, *J* = 9.1, 3.2 Hz, 2H), 2.03 (s, 3H), 1.60 (s, 3H), 1.36 (s, 18H), 1.33 (s, 3H), 1.20 (s, 3H).

^{13}C{^{1}H} NMR (151 MHz, $CDCl_3$) δ 159.4, 156.0, 154.0, 151.3, 139.7, 138.5, 138.4, 135.0, 134.5, 131.2, 130.5, 123.6, 121.5, 121.4, 121.3, 71.5, 70.9, 54.9, 42.4, 39.9, 35.1, 31.6, 31.6, 22.1, 20.8, 19.0, 18.1, 17.9, 15.3.

^{15}N NMR (61 MHz, $CDCl_3$) δ -234.2, -71.3.

^{19}F NMR (377 MHz, $CDCl_3$) δ -72.2, -74.1.

^{31}P NMR (162 MHz, $CDCl_3$) δ -131.3, -135.7, -140.1, -144.5, -148.9, -153.3, -157.7.

$[\alpha]^{25}_{D}$ = -190 (c = 1.00, ACN).

IR (ATR neat) ṽ =: 2956, 1641, 1586, 1463, 1364, 833, 768, 739, 556 cm^{-1}.

HRMS (ESI): *m/z* calculated for $C_{38}H_{52}N_3^{+}$ [M - PF_6^-] 550.4156; found 550.4158.

5.1.2 General procedures for preparation of camphor catalysts (**G.P. B**)

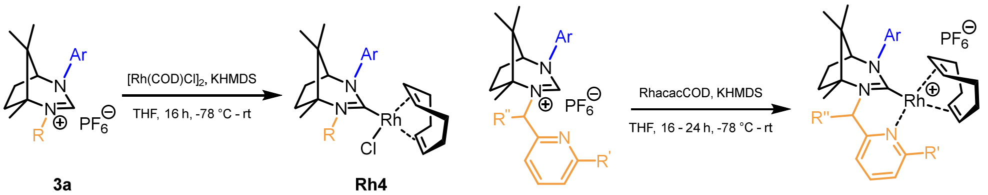

General procedure B-1: In the glovebox, a 10 mL vial was charged with $[Rh(COD)Cl]_2$ (1 eq.), camphor ligand **3a** (2.2 eq), and KHMDS (2.2 eq). The vial was then sealed with a Teflon cap and taken out of the glovebox. The reaction flask was cooled to -78 °C, and dry THF (3 - 5 mL) was slowly added. After the addition of the solvent, the reaction mixture was maintained at -78 °C for an additional hour, and then slowly warmed to room temperature. The reaction mixture was immediately subjected to column chromatography. After the separation of fractions, the collected product **Rh4** was transferred to a vial and stored under nitrogen in the refrigerator.

General procedure B-2: In the glovebox, a 10 mL vial was charged with Rh(acac)COD (1 eq.), camphor ligand **3b** (1.2 eq), and KHMDS (1.2 eq). The vial was then sealed with a Teflon cap and taken out of the glovebox. The reaction flask was cooled to -78 °C, and dry THF (3 - 5 mL) was slowly added. After the addition of the solvent, the reaction mixture was maintained at -78 °C for an additional hour, and then slowly warmed to room temperature. The reaction mixture was immediately subjected to column chromatography. After the separation of fractions, the collected product **Rh5** was transferred to a vial and stored under nitrogen in the refrigerator. IR measurements for **Rh5** were not reported, as all samples exhibited prominent absorption bands characteristic of PF_6^- groups, typically observed around 758 cm^{-1} and 555 cm^{-1}.

Complex Rh4aa

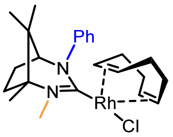

$C_{24}H_{34}ClN_2Rh$
488,91 g/mol

Following **G.P. B-1**, $[Rh(COD)Cl]_2$ (20 mg, 41 µmol, 1 eq.), **3aa** (31.2 mg, 81 µmol, 2.2 eq.), and KHMDS (18 mg, 90.5 µmol, 2.2 eq.) were dissolved in anhydrous THF (5 mL). The reaction mixture was stirred at -78 °C for 1 hour and then slowly warmed to room temperature over 16 hours. Column chromatography (SiO_2, DCM/MeOH 95:5, v/v) of the crude mixture yielded **Rh4aa** as a yellow solid (46 mg, 99%). Mixture of rotamers ca. 2:1, not assigned.

Rf 0.73 (SiO_2, DCM, v/v).

^{1}H NMR (600 MHz, $CDCl_3$) δ 8.00 – 7.88 (m, 2H), 7.44 – 7.35 (m, 2H), 7.34 – 7.22 (m, 1H), 4.96 – 4.77 (m, 2H), 3.91 (s, 1H), 3.80 (s, 2H), 3.54 – 3.26 (m, 2H), 2.80 – 2.75 (m, 1H), 2.60 – 2.49 (m, 1H), 2.41 – 2.27 (m, 1H), 2.27 – 2.17 (m, 1H), 2.05 – 1.88 (m, 1H), 1.88 – 1.79 (m, 1H), 1.79 – 1.69 (m, 2H), 1.69 – 1.58 (m, 1H), 1.51 – 1.37 (m, 2H), 1.31 – 1.21 (m, 4H), 1.21 – 1.08 (m, 2H), 1.06 (s, 2H), 1.01 (s, 2H), 0.96 (s, 1H).

^{13}C{^{1}H} NMR (151 MHz, $CDCl_3$) δ 205.5, 205.4, 205.2, 205.1, 147.6, 146.2, 128.8, 128.6, 128.5, 128.5, 126.8, 126.0, 124.8, 94.5, 94.5, 94.4, 94.3, 94.0, 94.0, 93.4, 93.4, 72.6, 71.4, 71.3, 70.9, 70.7, 70.4, 70.3, 70.0, 69.0, 68.9, 68.7, 42.9, 42.6, 41.9, 40.6, 38.8, 37.7, 34.9, 34.7, 32.7, 30.7, 30.6, 30.5, 29.8, 28.6, 28.5, 28.1, 27.2, 27.1, 22.8, 22.3, 18.3, 18.1, 16.1, 15.7.

IR (ATR neat) ṽ =: 3056, 2912, 2871, 2826, 2212, 1734, 1652, 1593, 1483, 1463, 1431, 755, 567 cm^{-1}.

HRMS (ESI): *m/z* calculated for $C_{24}H_{34}N_2Rh^+$ [M – Cl^-] 453.1772; found 453.1768.

Complex Rh4ab

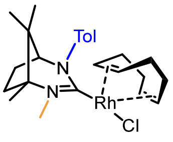

$C_{25}H_{36}ClN_2Rh$
502,93 g/mol

Following **G.P. B-1**, $[Rh(COD)Cl]_2$ (20 mg, 41 µmol, 1 eq.), **3ab** (31.2 mg, 81 µmol, 2.2 eq.), and KHMDS (18 mg, 90.5 µmol, 2.2 eq.) were dissolved in anhydrous THF (5 mL). The reaction mixture was stirred at -78 °C for 1 hour and then slowly warmed to room temperature over 16 hours. Column chromatography (SiO_2, DCM/MeOH 95:5, v/v) of the crude mixture yielded **Rh4ab** as a yellow solid (53 mg, 99%). Mixture of rotamers ca. 8:2, not assigned.

Rf 0.65 (SiO_2, DCM, v/v).

^{1}H NMR (600 MHz, $CDCl_3$) δ 8.73 – 8.47 (m, 1H), 7.36 – 7.27 (m, 3H), 4.84 – 4.61 (m, 2H), 3.89 (d, *J* = 29.0 Hz, 3H), 3.52 (dt, *J* = 125.3, 7.4 Hz, 1H), 3.18 (d, *J* = 4.3 Hz, 1H), 2.83 – 2.70 (m, 1H), 2.51 – 2.17 (m, 5H), 1.97 – 1.87 (m, 1H), 1.88 – 1.73 (m, 3H), 1.74 – 1.60 (m, 1H), 1.52 – 1.34 (m, 2H), 1.36 – 1.22 (m, 6H), 1.20 (s, 3H), 1.02 (m, 3H).

^{13}C{^{1}H} NMR (151 MHz, $CDCl_3$) δ 207.0, 206.7, 205.8, 205.5, 144.8, 142.6, 142.1, 133.4, 133.2, 131.6, 131.3, 130.2, 130.1, 130.1, 130.0, 130.0, 127.7, 126.5, 126.5, 125.9, 125.2, 125.1, 124.9, 95.1, 93.9, 93.8, 93.5, 93.5, 92.8, 92.8, 92.2, 92.1, 78.6, 78.5, 69.7, 68.8, 68.7, 68.6, 68.5, 68.5, 68.3, 68.2, 68.2, 67.6, 67.5, 66.1, 66.1, 66.0, 63.0, 62.9, 43.1, 41.7, 41.2, 40.5, 40.3, 40.2, 37.0, 36.6, 36.1, 34.5, 34.2, 31.2, 30.9, 30.2, 29.9, 29.7, 29.1, 29.0, 28.7, 28.5, 28.3, 27.2, 27.1, 27.0, 25.8, 25.6, 21.7, 21.7, 21.6, 21.5, 19.8, 18.0, 17.9, 17.6, 17.6, 17.4, 15.1, 15.0, 14.4, 13.1.

IR (ATR neat) ṽ =: 2955, 2922, 2871, 2826, 2213, 1733, 1684, 1550, 1482, 1463, 755, 573 cm^{-1}.

HRMS (ESI): *m/z* calculated for $C_{25}H_{36}N_2Rh^+$ [M – Cl^-] 467.1928; found 467.1936.

Complex Rh4ac

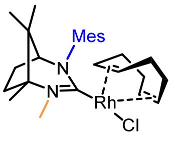

$C_{27}H_{40}ClN_2Rh$
530,99 g/mol

Following **G.P. B-1**, $[Rh(COD)Cl]_2$ (20 mg, 41 µmol, 1 eq.), **3ac** (35 mg, 81 µmol, 2.2 eq.), and KHMDS (18 mg, 90.5 µmol, 2.2 eq.) were dissolved in anhydrous THF (5 mL). The reaction mixture was stirred at -78 °C for 1 hour and then slowly warmed to room temperature over 16 hours. Column chromatography (SiO_2, DCM/MeOH 98:2, v/v) of the crude mixture yielded **Rh4ac** as a yellow solid (31 mg, 72%). The product was designated as a single rotamer.

Rf 0.15 (SiO_2, DCM, v/v).

^{1}H NMR (600 MHz, $CDCl_3$) δ 7.07 (s, 1H), 6.99 (s, 1H), 4.47 (s, 2H), 3.84 (s, 3H), 3.73 (s, 1H), 3.17 (d, *J* = 3.5 Hz, 1H), 2.82 (s, 1H), 2.44 (s, 3H), 2.37 (s, 3H), 2.34 (s, 3H), 2.31 – 2.16 (m, 3H), 1.94 (s, 2H), 1.90 – 1.77 (m, 3H), 1.74 (dd, *J* = 14.4, 7.7 Hz, 1H), 1.52 – 1.36 (m, 2H), 1.34 (s, 4H), 1.06 (s, 3H), 1.00 (s, 3H).

^{13}C{^{1}H} NMR (151 MHz, $CDCl_3$) δ 204.3, 204.0, 139.2, 136.4, 136.2, 133.2, 132.8, 132.4, 131.5, 129.3, 129.1, 128.7, 127.8, 127.6, 124.9, 96.5, 96.4, 93.3, 93.3, 69.9, 68.8, 68.7, 67.3, 62.3, 62.2, 51.4, 50.8, 50.0, 49.7, 40.8, 40.3, 37.9, 35.4, 29.7, 29.4, 28.7, 27.4, 24.6, 21.5, 19.8, 19.0, 17.6, 15.3.

IR (ATR neat) ṽ =: 2957, 2920, 2874, 2829, 1655, 1609, 1560, 1480, 727, 557 cm^{-1}.

HRMS (ESI): *m/z* calculated for $C_{27}H_{40}N_2Rh^+$ [M – Cl^-] 495.2241; found 495.2248.

Complex Rh4ad

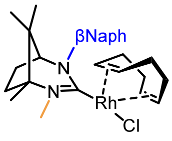

$C_{28}H_{36}ClN_2Rh$
538,97 g/mol

Following **G.P. B-1**, $[Rh(COD)Cl]_2$ (20 mg, 41 µmol, 1 eq.), **3ad** (34 mg, 81 µmol, 2.2 eq.), and KHMDS (18 mg, 90.5 µmol, 2.2 eq.) were dissolved in anhydrous THF (5 mL). The reaction mixture was stirred at -78 °C for 1 hour and then slowly warmed to room temperature over 16 hours. Column chromatography (SiO_2, DCM/MeOH 97:3, v/v) of the crude mixture yielded **Rh4ad** as a yellow solid (27 mg, 62%). Mixture of rotamers ca. 2:1, not assigned.

Rf 0.70 (SiO_2, DCM, v/v).

^{1}H NMR (600 MHz, $CDCl_3$) δ 8.97 (s, 1H), 7.99 (t, J = 8.3 Hz, 1H), 7.90 – 7.81 (m, 2H), 7.76 – 7.58 (m, 1H), 7.55 – 7.44 (m, 2H), 4.89 – 4.65 (m, 2H), 3.94 (s, 1H), 3.84 (s, 2H), 3.58 – 3.28 (m, 2H), 2.63 (ddd, J = 13.8, 10.9, 6.0 Hz, 1H), 2.42 – 2.21 (m, 3H), 1.88 (ddt, J = 14.1, 9.9, 4.8 Hz, 2H), 1.83 – 1.66 (m, 2H), 1.37 – 1.19 (m, 8H), 1.13 (s, 2H), 1.06 (s, 2H), 1.00 (s, 1H), 0.78 – 0.68 (m, 1H).

^{13}C{^{1}H} NMR (151 MHz, $CDCl_3$) δ 205.7, 205.6, 205.4, 205.4, 144.5, 143.0, 133.3, 133.2, 132.6, 131.9, 131.6, 131.0, 128.9, 128.2, 128.1, 128.0, 127.9, 127.5, 127.5, 127.3, 126.8, 126.7, 126.4, 126.0, 125.6, 122.5, 122.2, 94.9, 94.9, 94.8, 94.7, 94.1, 94.1, 93.6, 93.5, 72.0, 71.1, 71.1, 70.7, 70.6, 70.3, 70.2, 70.1, 68.8, 68.7, 68.6, 68.3, 42.9, 42.7, 42.1, 40.7, 38.9, 37.8, 35.2, 35.0, 32.7, 30.6, 30.2, 30.0, 29.8, 28.3, 28.2, 27.0, 27.0, 22.9, 22.4, 18.4, 18.2, 16.2, 15.8.

IR (ATR neat) ṽ =: 2978, 2920, 2871, 2585, 2829, 1731, 1631, 1599, 1507, 1485 cm^{-1}.

HRMS (ESI): *m*/*z* calculated for $C_{28}H_{36}N_2Rh^+$ [M – Cl^-] 503.1928; found 503.1916.

Complex Rh4ae

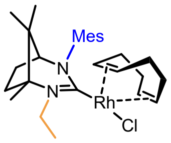

$C_{28}H_{42}ClN_2Rh$
545,01 g/mol

Following **G.P. B-1**, $[Rh(COD)Cl]_2$ (20 mg, 41 µmol, 1 eq.), **3ae** (35 mg, 81 µmol, 2.2 eq.), and KHMDS (18 mg, 90.5 µmol, 2.2 eq.) were dissolved in anhydrous THF (5 mL). The reaction mixture was stirred at -78 °C for 1 hour and then slowly warmed to room temperature over 16 hours. Column chromatography (SiO_2, DCM/MeOH 98:2, v/v) of the crude mixture yielded **Rh4ae** as a yellow solid (31 mg, 72%). The product was designated as a single rotamer.

Rf 0.20 (SiO_2, DCM, v/v).

^{1}H NMR (600 MHz, $CDCl_3$) δ 7.04 (s, 1H), 6.96 (s, 1H), 5.13 (s, 1H), 4.53 (t, J = 7.2 Hz, 1H), 4.37 (q, J = 7.5 Hz, 1H), 4.17 (s, 1H), 3.54 (s, 1H), 3.16 (d, J = 3.4 Hz, 1H), 2.85 (s, 1H), 2.55 – 2.42 (m, 4H), 2.42 – 2.32 (m, 8H), 2.29 – 2.22 (m, 1H), 1.96 (s, 1H), 1.85 (t, J = 11.6 Hz, 2H), 1.76 (dtd, J = 36.5, 12.4, 12.0, 7.0 Hz, 2H), 1.61 (d, J = 25.3 Hz, 1H), 1.54 (t, J = 6.9 Hz, 3H), 1.50 – 1.43 (m, 2H), 1.40 (s, 3H), 1.03 (s, 3H), 0.98 (s, 3H).

^{13}C{^{1}H} NMR (151 MHz, $CDCl_3$) δ 208.5, 208.2, 140.8, 138.6, 137.0, 133.5, 130.6, 129.7, 128.8, 97.2, 97.2, 94.1, 94.1, 70.7, 69.7, 69.6, 67.8, 63.4, 63.3, 50.1, 41.8, 40.3, 36.4, 31.1, 30.6, 28.8, 25.8, 23.3, 23.0, 20.9, 20.1, 18.8, 18.4, 16.2.

IR (ATR neat) ṽ =: 2978, 2920, 2871, 2585, 2829, 1731, 1631, 1599, 1507 cm^{-1}.

HRMS (ESI): *m*/*z* calculated for $C_{28}H_{42}N_2Rh^+$ [M – Cl^-] 509.2398; found 509.2404.

Complex Rh4af

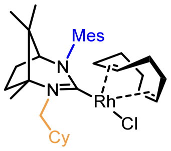

$C_{33}H_{50}ClN_2Rh$
613,13 g/mol

Following **G.P. B-1**, $[Rh(COD)Cl]_2$ (30 mg, 61 µmol, 1 eq.), **3af** (69 mg, 134 µmol, 2.2 eq.), and KHMDS (27 mg, 134 µmol, 2.2 eq.) were dissolved in anhydrous THF (5 mL). The reaction mixture was stirred at -78 °C for 1 hour and then slowly warmed to room temperature over 16 hours. Column chromatography (SiO_2, DCM/MeOH 95:5, v/v) of the crude mixture yielded **Rh4af** as a yellow solid (28 mg, 64%). Mixture of rotamers ca. 85:15, not assigned.

Rf 0.80 (SiO_2, DCM/MeOH v/v).

^{1}H NMR (600 MHz, $CDCl_3$) δ 6.95 (d, J = 5.3 Hz, 1H), 6.81 (d, J = 40.5 Hz, 1H), 4.39 (dt, J = 15.5, 6.5 Hz, 2H), 3.41 – 3.30 (m, 1H), 3.04 (d, J = 3.1 Hz, 1H), 2.89 (s, 1H), 2.77 (d, J = 4.0 Hz, 0H), 2.60 (s, 0H), 2.47 (s, 3H), 2.43 – 2.28 (m, 3H), 2.23 (t, J = 7.3 Hz, 6H), 2.20 – 2.14 (m, 1H), 1.94 – 1.80 (m, 3H), 1.80 – 1.63 (m, 6H), 1.60 (d, J = 12.6 Hz, 1H), 1.55 – 1.41 (m, 5H), 1.40 – 1.23 (m, 5H), 1.13 – 1.07 (m, 2H), 1.07 – 1.00 (m, 1H), 0.97 (s, 3H), 0.85 (d, J = 25.2 Hz, 3H), 0.81 – 0.71 (m, 1H).

^{13}C{^{1}H} NMR (151 MHz, $CDCl_3$) δ 211.1, 210.7, 137.0, 133.6, 130.8, 130.7, 129.6, 128.9, 96.7, 96.6, 93.6, 93.6, 72.3, 71.2, 69.2, 69.1, 68.9, 43.0, 42.2, 39.9, 39.5, 39.4, 39.0, 35.4, 32.2, 32.1, 31.2, 31.1, 30.7, 29.8, 29.8, 29.8, 29.3, 27.3, 27.0, 26.8, 26.7, 26.7, 26.5, 24.4, 23.6, 23.6, 21.3, 20.9, 20.8, 20.3, 20.3, 19.2, 17.6.

IR (ATR neat) ṽ =: 2919, 2850, 1724, 1686, 1641, 1609, 1541, 1447 cm^{-1}.

HRMS (ESI): *m/z* calculated for $C_{33}H_{50}N_2Rh^+$ [M – Cl^-] 577.3024; found 577.3037.

Complex Rh4ag

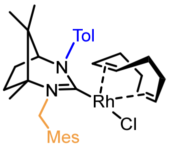

$C_{34}H_{46}ClN_2Rh$
621,11 g/mol

Following **G.P. B-1**, $[Rh(COD)Cl]_2$ (20 mg, 41 µmol, 1 eq.), **3ag** (33 mg, 81 µmol, 2.2 eq.), and KHMDS (18 mg, 90.5 µmol, 2.2 eq.) were dissolved in anhydrous THF (5 mL). The reaction mixture was stirred at -78 °C for 1 hour and then slowly warmed to room temperature over 16 hours. Column chromatography (SiO_2, DCM/MeOH 95:5, v/v) of the crude mixture yielded **Rh4ag** as a yellow solid (48 mg, 97%). Mixture of rotamers ca. 6:4, not assigned.

Rf 0.46 (SiO_2, DCM, v/v).

^{1}H NMR (600 MHz, $CDCl_3$) δ 8.78 (dd, *J* = 80.7, 7.4 Hz, 1H), 7.46 – 7.37 (m, 1H), 7.35 – 7.23 (m, 1H), 6.93 – 6.73 (m, 3H), 5.31 (dd, *J* = 91.4, 16.0 Hz, 1H), 4.81 – 4.56 (m, 2H), 4.27 – 3.67 (m, 2H), 3.11 (dd, *J* = 34.3, 4.6 Hz, 1H), 3.00 (ddd, *J* = 14.1, 9.5, 5.1 Hz, 1H), 2.68 (t, *J* = 7.6 Hz, 1H), 2.55 – 2.37 (m, 8H), 2.35 – 2.22 (m, 7H), 2.21 – 2.10 (m, 3H), 2.08 – 1.99 (m, 1H), 1.74 (ddt, *J* = 17.1, 9.2, 4.7 Hz, 2H), 1.69 – 1.63 (m, 2H), 1.60 – 1.48 (m, 2H), 1.47 – 1.36 (m, 2H), 1.30 (d, *J* = 12.7 Hz, 3H), 1.17 (s, 1H), 0.87 (d, *J* = 14.5 Hz, 3H), 0.68 (s, 2H).

^{13}C{^{1}H} NMR (151 MHz, $CDCl_3$) δ 214.0, 213.7, 208.7, 208.4, 147.2, 144.6, 138.0, 137.9, 137.4, 137.1, 136.8, 136.6, 136.5, 134.7, 134.1, 132.9, 132.5, 132.3, 130.9, 130.9, 130.8, 130.4, 130.2, 130.0, 129.9, 129.8, 129.7, 127.7, 127.2, 127.0, 126.3, 126.2, 95.2, 95.1, 95.1, 95.1, 95.0, 94.9, 94.3, 94.3, 78.8, 78.7, 72.7, 71.2, 70.9, 69.9, 68.7, 68.6, 67.4, 67.3, 66.1, 66.0, 65.5, 65.4, 58.9, 57.7, 43.3, 43.0, 37.9, 36.8, 33.2, 32.7, 32.4, 31.1, 31.0, 30.9, 30.7, 28.1, 28.1, 27.9, 27.8, 23.5, 22.7, 21.7, 21.3, 21.1, 20.9, 20.9, 20.9, 20.8, 20.7, 20.2, 19.4, 18.7, 18.1, 14.1.

IR (ATR neat) ṽ =: 2955, 2917, 2874, 2828, 2217, 1660, 1607, 1493, 1422 cm^{-1}.

HRMS (ESI): *m/z* calculated for $C_{34}H_{46}N_2Rh^+$ [M – Cl^-] 585.2711; found 585.2715.

Complex Rh4ah

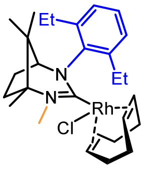

$C_{28}H_{42}ClN_2Rh$
545,01 g/mol

Following **G.P. B-1**, $[Rh(COD)Cl]_2$ (15 mg, 30 µmol, 1 eq.), **3ah** (27 mg, 59 µmol, 2.2 eq.), and KHMDS (14 mg, 68 µmol, 2.2 eq.) were dissolved in anhydrous THF (5 mL). The reaction mixture was stirred at -78 °C for 1 hour and then slowly warmed to room temperature over 16 hours. Column chromatography (SiO_2, DCM/MeOH 95:5, v/v) of the crude mixture yielded **Rh4ah** as a yellow solid (23 mg, 72%). The product was designated as a single rotamer.

Rf 0.13 (SiO_2, DCM, v/v).

^{1}H NMR (600 MHz, $CDCl_3$) δ 7.39 – 7.31 (m, 2H), 7.20 (dd, *J* = 7.2, 1.6 Hz, 1H), 4.51 (t, *J* = 7.6 Hz, 1H), 4.40 (q, *J* = 8.0 Hz, 1H), 4.22 (s, 1H), 4.00 (s, 3H), 3.61 (t, *J* = 6.6 Hz, 1H), 3.22 (dq, *J* = 16.2, 8.5, 8.0 Hz, 1H), 3.18 (s, 1H), 2.75 – 2.59 (m, 3H), 2.59 – 2.55 (m, 1H), 2.51 – 2.40 (m, 3H), 2.20 (t, *J* = 10.2 Hz, 1H), 1.94 (dd, *J* = 14.4, 5.7 Hz, 1H), 1.85 – 1.78 (m, 2H), 1.73 (ddt, *J* = 22.4, 14.9, 7.0 Hz, 2H), 1.66 (s, 1H), 1.46 (tdt, *J* = 14.8, 10.2, 5.5 Hz, 1H), 1.32 (s, 3H), 1.28 (t, *J* = 7.6 Hz, 6H), 1.10 (s, 3H), 0.98 (s, 3H).

^{13}C{^{1}H} NMR (151 MHz, $CDCl_3$) δ 207.4, 207.1, 144.5, 142.1, 139.5, 128.0, 127.8, 126.6, 97.3, 97.3, 94.5, 94.5, 78.9, 78.8, 70.9, 69.2, 69.1, 68.1, 63.1, 63.0, 42.3, 41.4, 39.1, 36.5, 31.0, 30.9, 30.2, 29.8, 28.6, 27.4, 25.7, 24.8, 22.9, 19.0, 17.4, 16.6, 14.8.

IR (ATR neat) ṽ =: 3402, 2985, 2958, 2923, 2868, 2819, 1726, 1686, 1548 cm^{-1}.

HRMS (ESI): *m/z* calculated for $C_{28}H_{42}N_2Rh^+$ [M – Cl^-] 509.2398; found 509.2407.

Complex Rh4ai

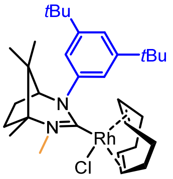

$C_{32}H_{50}ClN_2Rh$
601,12 g/mol

Following **G.P. B-1**, $[Rh(COD)Cl]_2$ (20 mg, 41 μmol, 1 eq.), **3ai** (41 mg, 81 μmol, 2.2 eq.), and KHMDS (18 mg, 90.5 μmol, 2.2 eq.) were dissolved in anhydrous THF (5 mL). The reaction mixture was stirred at -78 °C for 1 hour and then slowly warmed to room temperature over 16 hours. Column chromatography (SiO_2, DCM/MeOH 97:3, v/v) of the crude mixture yielded **Rh4ai** as a yellow solid (44 mg, 61%). Mixture of rotamers ca. 2:1, not assigned.

Rf 0.43 (SiO_2, DCM, v/v).

^{1}H NMR (600 MHz, CD_2Cl_2) δ 7.40 (dt, *J* = 37.3, 1.7 Hz, 1H), 4.60 – 4.45 (m, 1H), 4.44 (td, *J* = 8.0, 3.8 Hz, 1H), 4.02 (s, 1H), 3.91 (s, 2H), 3.37 – 3.11 (m, 2H), 2.63 (dq, *J* = 101.6, 7.6, 4.2 Hz, 1H), 2.41 (ddd, *J* = 13.6, 9.6, 4.0 Hz, 1H), 2.37 – 2.27 (m, 1H), 2.26 – 1.97 (m, 3H), 1.89 – 1.73 (m, 3H), 1.69 – 1.45 (m, 3H), 1.38 (d, *J* = 5.7 Hz, 18H), 1.29 (d, *J* = 7.1 Hz, 3H), 1.21 – 1.12 (m, 2H), 1.05 (s, 2H), 1.02 (s, 2H), 0.96 (s, 1H).

^{13}C{^{1}H} NMR (151 MHz, CD_2Cl_2) δ 206.0, 205.7, 205.3, 205.0, 151.7, 151.6, 147.9, 146.2, 121.0, 120.4, 95.7, 95.6, 95.1, 95.1, 95.1, 95.0, 94.9, 72.8, 71.3, 69.7, 69.3, 69.2, 68.6, 67.8, 67.7, 66.8, 66.7, 41.5, 41.2, 40.9, 40.5, 39.0, 38.4, 35.4, 34.1, 33.5, 33.0, 31.6, 30.9, 30.6, 29.0, 28.8, 28.5, 28.1, 22.4, 22.4, 18.2, 17.8, 16.0, 15.9.

IR (ATR neat) ṽ =: 2952, 2873, 2829, 1590, 1485, 1446, 1434, 1403, 1382, 1373, 732 cm^{-1}.

HRMS (ESI): *m*/*z* calculated for $C_{32}H_{50}N_2Rh^+$ [M – Cl^-] 565.3024; found 565.3039.

Complex Rh5ba

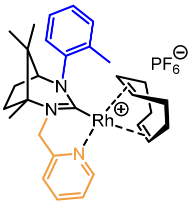

$C_{30}H_{39}F_6N_3PRh$
689,53 g/mol

Following **G.P. B-2**, Rh(acac)COD (30 mg, 97 μmol, 1 eq.), **3ba** (54 mg, 145 μmol, 1.5 eq.), and KHMDS (29 mg, 145 μmol, 1.5 eq.) were dissolved in anhydrous THF (5 mL). The reaction mixture was stirred at -78 °C for 1 hour and then slowly warmed to room temperature over 16 hours. Column chromatography (SiO_2, petroleum ether/EtOAc 1:1, followed by DCM/EtOAc 95:5, v/v) of the crude mixture yielded **Rh5ba** as a yellow solid (60 mg, 86%). Mixture of rotamers ca. 95:15, not assigned.

Rf 0.56 (SiO_2, DCM/MeOH 9:1, v/v).

^{1}H NMR (600 MHz, CD_2Cl_2) δ 8.38 (d, *J* = 5.4 Hz, 1H), 8.02 (td, *J* = 7.7, 1.6 Hz, 1H), 7.74 (d, *J* = 7.6 Hz, 1H), 7.56 (ddd, *J* = 7.2, 5.4, 1.2 Hz, 1H), 7.54 – 7.43 (m, 2H), 7.42 – 7.33 (m, 1H), 7.18 (dd, *J* = 7.4, 1.4 Hz, 1H), 5.78 (d, *J* = 15.2 Hz, 1H), 5.09 (d, *J* = 15.2 Hz, 1H), 4.87 – 4.75 (m, 1H), 4.29 (dt, *J* = 63.1, 7.2 Hz, 1H), 4.10 – 3.89 (m, 1H), 3.30 (dd, *J* = 21.1, 4.7 Hz, 1H), 2.60 – 2.49 (m, 1H), 2.46 – 2.33 (m, 1H), 2.31 (s, 3H), 2.07 (dddt, *J* = 25.5, 17.8, 10.1, 3.2 Hz, 1H), 2.01 – 1.86 (m, 3H), 1.78 – 1.64 (m, 2H), 1.60 (dq, *J* = 14.6, 8.2 Hz, 1H), 1.54 (s, 3H), 1.52 – 1.36 (m, 2H), 1.30 – 1.22 (m, 5H), 1.07 (s, 3H).

^{13}C{^{1}H} NMR (151 MHz, CD_2Cl_2) δ 210.2, 209.9, 157.5, 151.4, 151.2, 150.9, 143.5, 139.8, 139.7, 133.3, 132.7, 132.6, 129.3, 128.4, 128.0, 127.4, 126.3, 125.9, 125.2, 124.8, 124.6, 97.8, 97.8, 97.7, 78.6, 78.6, 73.4, 70.9, 70.4, 70.2, 70.1, 69.8, 56.3, 43.3, 40.8, 40.4, 40.1, 39.4, 34.7, 33.1, 31.3, 30.7, 30.4, 30.0, 29.9, 29.0, 27.3, 22.6, 22.3, 22.2, 20.4, 19.9, 18.6, 18.5, 18.3, 17.7, 16.9, 16.6, 15.5.

^{15}N NMR (61 MHz, CD_2Cl_2) δ -124.8, -229.7.

^{19}F NMR (377 MHz, CD_2Cl_2) δ -72.1, -74.0.

^{31}P NMR (162 MHz, CD_2Cl_2) δ -131.4, -135.8, -140.2, -144.5, -148.9, -153.3, -157.7.

$[\alpha]^{25}_D$ = +28 (c = 1.00, $CHCl_3$).

IR (ATR neat) ṽ =: 2945, 2878, 2832, 1732, 1604, 1477, 1462 cm^{-1}.

UV-Vis: 440 nm, 331 nm (c = 4*10^{-6} mol/dm^3, $CHCl_3$)

HRMS (ESI): *m*/*z* calculated for $C_{30}H_{39}N_3Rh^+$ [M - PF_6^-] 544.2194; found 544.2190.

Complex Rh5bb

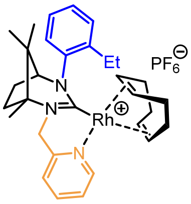

$C_{31}H_{41}F_6N_3PRh$
703,56 g/mol

Following **G.P. B-2**, Rh(acac)COD (20 mg, 65 µmol, 1 eq.), **3bb** (40 mg, 77 µmol, 1.2 eq.), and KHMDS (18 mg, 90 µmol, 1.4 eq.) were dissolved in anhydrous THF (5 mL). The reaction mixture was stirred at -78 °C for 1 hour and then slowly warmed to room temperature over 16 hours. Column chromatography (SiO_2, petroleum ether/EtOAc 1:1, followed by DCM/EtOAc 95:5, v/v) of the crude mixture yielded **Rh5bb** as a yellow solid (36 mg, 79%). Mixture of rotamers ca. 90:10, not assigned.

Rf 0.68 (SiO_2, DCM/MeOH 9:1, v/v).

^{1}H NMR (600 MHz, $CDCl_3$) δ 8.45 (d, *J* = 5.3 Hz, 1H), 8.02 (td, *J* = 7.6, 1.5 Hz, 1H), 7.87 (d, *J* = 7.5 Hz, 1H), 7.62 (ddd, *J* = 8.1, 4.7, 2.0 Hz, 1H), 7.57 (td, *J* = 7.4, 1.6 Hz, 1H), 7.53 (td, *J* = 7.5, 1.5 Hz, 1H), 7.47 (dd, *J* = 7.6, 1.3 Hz, 1H), 7.18 (dd, *J* = 7.4, 1.3 Hz, 1H), 5.78 (d, *J* = 15.2 Hz, 1H), 5.20 (d, *J* = 15.2 Hz, 1H), 4.90 – 4.84 (m, 1H), 4.32 (t, *J* = 7.1 Hz, 1H), 3.96 (dq, *J* = 32.0, 7.5 Hz, 1H), 3.29 (d, *J* = 2.4 Hz, 1H), 2.65 (qd, *J* = 7.6, 4.0 Hz, 2H), 2.52 (q, *J* = 7.7 Hz, 1H), 2.40 (dtd, *J* = 15.6, 9.3, 6.6 Hz, 1H), 2.10 – 1.94 (m, 3H), 1.94 – 1.85 (m, 2H), 1.79 – 1.67 (m, 3H), 1.58 (s, 3H), 1.51 – 1.40 (m, 2H), 1.34 (dt, *J* = 17.3, 7.5 Hz, 3H), 1.29 – 1.23 (m, 4H), 1.08 (s, 3H).

^{13}C{^{1}H} NMR (151 MHz, $CDCl_3$) δ 210.1, 157.1, 151.0, 142.7, 139.6, 138.2, 129.8, 129.2, 128.3, 127.2, 126.2, 124.6, 97.7, 97.7, 97.3, 77.9, 77.8, 70.6, 70.2, 69.5, 69.4, 56.1, 43.0, 39.1, 34.4, 29.7, 29.7, 28.8, 27.0, 23.2, 22.7, 18.3, 16.3, 14.0.

^{15}N NMR (61 MHz, $CDCl_3$) δ -122.6, -226.5.

^{19}F NMR (377 MHz, $CDCl_3$) δ -72.5, -74.4.

^{31}P NMR (162 MHz, $CDCl_3$) δ -131.2, -135.5, -139.9, -144.3, -148.7, -153.1, -157.5.

HRMS (ESI): *m*/*z* calculated for $C_{31}H_{41}N_3Rh^+$ [M - PF_6^-] 558.2350; found 558.2355.

Complex Rh5bc

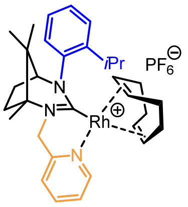

$C_{32}H_{43}F_6N_3PRh$
717,59 g/mol

Following **G.P. B-2**, Rh(acac)COD (20 mg, 65 µmol, 1 eq.), **3bc** (40 mg, 77 µmol, 1.2 eq.), and KHMDS (18 mg, 90 µmol, 1.4 eq.) were dissolved in anhydrous THF (5 mL). The reaction mixture was stirred at -78 °C for 1 hour and then slowly warmed to room temperature over 16 hours. Column chromatography (SiO_2, petroleum ether/EtOAc 1:1, followed by DCM/EtOAc 95:5, v/v) of the crude mixture yielded **Rh5bc** as a yellow solid (42 mg, 91%). Mixture of rotamers ca. 93:7, not assigned.

Rf 0.65 (SiO_2, DCM/MeOH 9:1, v/v).

^{1}H NMR (600 MHz, $CDCl_3$) δ 8.42 (d, *J* = 5.2 Hz, 1H), 7.98 (td, *J* = 7.7, 1.5 Hz, 1H), 7.84 (d, *J* = 7.6 Hz, 1H), 7.58 (ddd, *J* = 7.0, 5.5, 1.2 Hz, 1H), 7.54 – 7.48 (m, 2H), 7.47 – 7.41 (m, 1H), 7.15 (dd, *J* = 5.6, 3.4 Hz, 1H), 5.76 (d, *J* = 15.2 Hz, 1H), 5.18 (d, *J* = 15.2 Hz, 1H), 4.87 – 4.81 (m, 1H), 4.26 (t, *J* = 7.1 Hz, 1H), 3.96 (q, *J* = 7.6 Hz, 1H), 3.28 (s, 1H), 3.01 (hept, *J* = 6.8 Hz, 1H), 2.56 (q, *J* = 7.9 Hz, 1H), 2.36 (dtd, *J* = 16.5, 9.7, 6.9 Hz, 1H), 2.06 – 1.80 (m, 4H), 1.75 – 1.62 (m, 3H), 1.55 (s, 4H), 1.51 – 1.37 (m, 2H), 1.31 – 1.18 (m, 10H), 1.03 (s, 3H).

^{13}C{^{1}H} NMR (151 MHz, $CDCl_3$) δ 210.4, 210.1, 157.2, 151.0, 143.2, 142.1, 139.6, 129.5, 128.2, 127.9, 127.1, 126.2, 124.7, 97.5, 97.5, 97.4, 97.3, 77.7, 77.6, 71.0, 70.8, 69.5, 69.4, 68.1, 56.2, 42.9, 39.1, 34.3, 29.8, 29.7, 29.6, 28.7, 27.6, 27.1, 25.7, 24.9, 23.8, 22.8, 18.3, 16.3.

^{15}N NMR (61 MHz, $CDCl_3$) δ -122.3, -225.6.

^{19}F NMR (377 MHz, $CDCl_3$) δ -72.6, -74.5.

^{31}P NMR (162 MHz, $CDCl_3$) δ -131.2, -135.5, -139.9, -144.3, -148.7, -153.1, -157.5.

HRMS (ESI): *m*/*z* calculated for $C_{32}H_{43}N_3Rh^+$ [M - PF_6^-] 620.2507; found 620.2516.

Complex Rh5bd

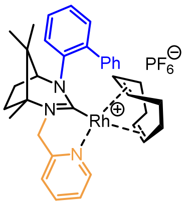

$C_{35}H_{41}F_6N_3PRh$
751,60 g/mol

Following **G.P. B-2**, Rh(acac)COD (20 mg, 65 µmol, 1 eq.), **3bd** (42 mg, 77 µmol, 1.2 eq.), and KHMDS (18 mg, 90 µmol, 1.4 eq.) were dissolved in anhydrous THF (5 mL). The reaction mixture was stirred at -78 °C for 1 hour and then slowly warmed to room temperature over 16 hours. Column chromatography (SiO_2, petroleum ether/EtOAc 1:1, followed by DCM/EtOAc 95:5, v/v) of the crude mixture yielded **Rh5bd** as a yellow solid (36 mg, 75%). Mixture of rotamers ca. 80:20, not assigned.

Rf 0.65 (SiO_2, DCM/MeOH 9:1, v/v).

^{1}H NMR (600 MHz, $CDCl_3$) δ 8.61 (dd, *J* = 21.3, 5.2 Hz, 1H), 7.94 (dtd, *J* = 28.3, 7.7, 1.5 Hz, 1H), 7.82 – 7.76 (m, 2H), 7.72 – 7.56 (m, 2H), 7.56 – 7.49 (m, 1H), 7.45 – 7.37 (m, 4H), 7.36 – 7.27 (m, 1H), 7.25 – 7.19 (m, 2H), 5.93 (dd, *J* = 30.1, 15.0 Hz, 1H), 5.21 (dd, *J* = 49.2, 15.0 Hz, 1H), 4.91 (dt, *J* = 7.0, 3.8 Hz, 1H), 4.40 (dt, *J* = 15.1, 7.2 Hz, 1H), 4.10 – 3.91 (m, 1H), 3.03 (dt, *J* = 12.9, 6.6 Hz, 1H), 2.98 (d, *J* = 4.5 Hz, 1H), 2.47 (dddd, *J* = 32.8, 23.6, 15.1, 6.6 Hz, 1H), 2.28 (dtd, *J* = 16.1, 9.3, 7.2 Hz, 1H), 2.17 – 2.08 (m, 1H), 2.08 – 1.96 (m, 2H), 1.70 – 1.45 (m, 5H), 1.45 – 1.40 (m, 3H), 1.20 (ddt, *J* = 12.2, 9.3, 4.5 Hz, 1H), 0.62 (s, 3H), 0.60 (s, 3H).

^{13}C{^{1}H} NMR (151 MHz, $CDCl_3$) δ 209.0, 208.7, 156.8, 156.1, 151.3, 151.2, 145.1, 142.4, 139.9, 139.5, 139.2, 138.7, 136.5, 136.4, 132.5, 132.2, 129.4, 128.9, 128.8, 128.7, 128.6, 128.1, 127.7, 127.3, 126.0, 125.5, 124.8, 124.4, 97.2, 97.2, 96.8, 96.4, 96.3, 73.4, 72.2, 72.0, 71.6, 71.5, 70.4, 69.7, 68.0, 56.7, 56.5, 42.1, 40.7, 39.3, 39.2, 34.1, 34.1, 30.5, 30.4, 29.9, 29.7, 29.6, 29.3, 29.1, 27.3, 27.1, 25.6, 23.8, 22.3, 21.9, 18.2, 17.5, 17.3, 16.5, 15.8, 15.0.

^{15}N NMR (61 MHz, $CDCl_3$) δ -121.7, -225.0.

^{19}F NMR (377 MHz, $CDCl_3$) δ -72.6, -74.5.

^{31}P NMR (162 MHz, $CDCl_3$) δ -131.1, -135.5, -139.9, -144.3, -148.7, -153.1, -157.5.

HRMS (ESI): *m*/*z* calculated for $C_{35}H_{41}N_3Rh^+$ [M - PF_6^-] 606.2350; found 606.2360.

Complex Rh5be

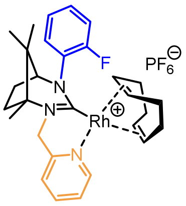

$C_{29}H_{36}F_7N_3PRh$
693,50 g/mol

Following **G.P. B-2**, Rh(acac)COD (20 mg, 65 µmol, 1 eq.), **3be** (38 mg, 77 µmol, 1.2 eq.), and KHMDS (18 mg, 90 µmol, 1.4 eq.) were dissolved in anhydrous THF (5 mL). The reaction mixture was stirred at -78 °C for 1 hour and then slowly warmed to room temperature over 16 hours. Column chromatography (SiO_2, petroleum ether/EtOAc 1:1, followed by DCM/EtOAc 95:5, v/v) of the crude mixture yielded **Rh5be** as a yellow solid (40 mg, 90%). Mixture of rotamers ca. 80:20, not assigned.

Rf 0.58 (SiO_2, DCM/MeOH 9:1, v/v).

^{1}H NMR (600 MHz, $CDCl_3$) δ 8.55 – 8.13 (m, 1H), 7.94 (dtd, *J* = 20.4, 7.6, 1.5 Hz, 1H), 7.80 (dd, *J* = 18.3, 7.7 Hz, 1H), 7.64 – 7.54 (m, 2H), 7.54 – 7.38 (m, 2H), 7.18 (d, *J* = 24.1 Hz, 1H), 5.82 (dd, *J* = 83.9, 15.0 Hz, 1H), 5.22 (dd, *J* = 57.9, 15.0 Hz, 1H), 5.08 – 4.73 (m, 1H), 4.26 (d, *J* = 62.3 Hz, 1H), 4.07 – 3.96 (m, 1H), 3.33 (d, *J* = 48.4 Hz, 1H), 2.58 – 2.33 (m, 2H), 2.30 – 2.20 (m, 1H), 2.12 – 1.95 (m, 3H), 1.91 (s, 1H), 1.81 – 1.68 (m, 2H), 1.66 – 1.56 (m, 2H), 1.56 – 1.43 (m, 5H), 1.16 (d, *J* = 23.1 Hz, 3H), 0.98 (d, *J* = 72.4 Hz, 3H).

^{13}C{^{1}H} NMR (151 MHz, $CDCl_3$) δ 208.7, 208.5, 156.6, 156.0, 155.7, 152.5, 152.4, 151.4, 139.6, 139.4, 136.7, 132.9, 131.6, 130.1, 129.0, 126.2, 125.9, 125.5, 125.2, 124.7, 124.6, 124.5, 123.0, 122.5, 116.8, 116.6, 110.5, 98.8, 97.8, 79.2, 72.7, 72.0, 71.3, 70.9, 56.5, 55.9, 53.4, 52.9, 43.6, 43.5, 42.7, 40.6, 40.1, 39.2, 38.2, 37.2, 34.2, 34.0, 31.6, 31.1, 30.3, 29.7, 29.2, 28.9, 27.0, 26.9, 24.3, 22.4, 22.0, 17.8, 17.6, 17.4, 17.1, 16.2, 16.2, 16.0, 15.4.

^{15}N NMR (61 MHz, $CDCl_3$) δ -125.8, -227.1.

^{19}F NMR (377 MHz, $CDCl_3$) δ -72.4, -74.3, -120.6, -122.3.

^{31}P NMR (162 MHz, $CDCl_3$) δ -131.1, -135.5, -139.9, -144.3, -148.7, -153.1, -157.5.

HRMS (ESI): *m*/*z* calculated for $C_{29}H_{36}FN_3Rh^+$ [M - PF_6^-] 548.1948; found 548.1950.

Complex Rh5bf

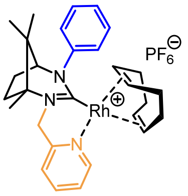

$C_{29}H_{37}F_6N_3PRh$
675,51 g/mol

Following **G.P. B-2**, Rh(acac)COD (20 mg, 65 µmol, 1 eq.), **3bf** (36 mg, 77 µmol, 1.2 eq.), and KHMDS (18 mg, 90 µmol, 1.4 eq.) were dissolved in anhydrous THF (5 mL). The reaction mixture was stirred at -78 °C for 1 hour and then slowly warmed to room temperature over 16 hours. Column chromatography (SiO_2, petroleum ether/EtOAc 1:1, followed by DCM/EtOAc 95:5, v/v) of the crude mixture yielded **Rh5bf** as a yellow solid (28 mg, 64%). Mixture of rotamers ca. 75:25, not assigned.

Rf 0.23 (SiO_2, DCM/MeOH 9:1, v/v).

^{1}H NMR (600 MHz, $CDCl_3$) δ 8.47 (d, *J* = 4.5 Hz, 1H), 7.95 (dtd, *J* = 24.3, 7.7, 1.5 Hz, 1H), 7.76 (dd, *J* = 70.7, 7.6 Hz, 1H), 7.66 – 7.53 (m, 3H), 7.50 – 7.36 (m, 3H), 5.83 (dd, *J* = 95.3, 15.1 Hz, 1H), 5.22 (dd, *J* = 58.5, 15.0 Hz, 1H), 4.98 (dt, *J* = 7.5, 4.0 Hz, 1H), 4.22 (dt, *J* = 48.8, 7.1 Hz, 1H), 4.03 (dt, *J* = 13.7, 8.0 Hz, 1H), 3.53 (t, *J* = 5.6 Hz, 1H), 2.54 – 2.22 (m, 4H), 2.21 – 2.09 (m, 1H), 2.02 (dtd, *J* = 28.1, 9.9, 8.0, 4.7 Hz, 2H), 1.93 – 1.87 (m, 1H), 1.83 – 1.71 (m, 1H), 1.64 – 1.49 (m, 5H), 1.48 – 1.40 (m, 2H), 1.21 (s, 1H), 1.13 (s, 2H), 1.07 (s, 2H), 0.93 (s, 1H).

^{13}C{^{1}H} NMR (151 MHz, $CDCl_3$) δ 207.2, 206.9, 157.0, 156.3, 156.2, 152.7, 152.1, 151.1, 147.3, 145.6, 139.7, 139.5, 139.3, 136.7, 129.8, 129.5, 128.1, 127.4, 126.1, 125.6, 125.2, 124.8, 124.8, 124.7, 122.9, 122.4, 110.4, 97.6, 97.6, 97.3, 80.1, 78.9, 78.8, 72.4, 72.3, 71.6, 71.3, 71.1, 71.1, 70.6, 62.8, 56.5, 55.9, 53.1, 43.7, 43.4, 42.4, 41.3, 40.2, 39.6, 37.4, 34.4, 34.2, 33.2, 31.3, 30.6, 29.8, 29.2, 28.9, 27.2, 27.0, 24.4, 22.5, 22.1, 17.9, 17.8, 17.2, 16.4, 16.3, 15.6.

^{15}N NMR (61 MHz, $CDCl_3$) δ -123.6, -228.1.

^{19}F NMR (377 MHz, $CDCl_3$) δ -72.4, -74.3, -120.6, -122.3.

^{31}P NMR (162 MHz, $CDCl_3$) δ -131.1, -135.5, -139.9, -144.3, -148.7, -153.1, -157.5.

HRMS (ESI): *m*/*z* calculated for $C_{29}H_{37}N_3Rh^+$ [M - PF_6^-] 548.1948; found 548.1950.

Complex Rh5bg

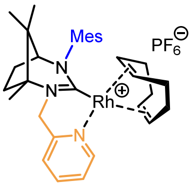

$C_{32}H_{43}F_6N_3PRh$
717,59 g/mol

Following **G.P. B-2**, $[Rh(COD)Cl]_2$ (30 mg, 97 µmol, 1 eq.), **3bg** (100 mg, 190 µmol, 2 eq.), and KHMDS (48 mg, 240 µmol, 2.5 eq.) were dissolved in anhydrous THF (5 mL). The reaction mixture was stirred at -78 °C for 1 hour and then slowly warmed to room temperature over 16 hours. Column chromatography (SiO_2, petroleum ether/EtOAc 1:1, followed by DCM/EtOAc 95:5, v/v) of the crude mixture yielded **Rh5bg** as a yellow solid (67 mg, 97%).

Rf 0.71 (SiO_2, DCM/MeOH 9:1, v/v).

^{1}H NMR (600 MHz, $CDCl_3$) δ 8.21 (d, *J* = 5.4 Hz, 1H), 7.96 (td, *J* = 7.7, 1.5 Hz, 1H), 7.79 (d, *J* = 7.5 Hz, 1H), 7.50 (ddd, *J* = 7.2, 5.5, 1.2 Hz, 1H), 7.15 (s, 1H), 6.92 (s, 1H), 5.88 (d, *J* = 15.2 Hz, 1H), 5.16 (d, *J* = 15.2 Hz, 1H), 4.73 – 4.67 (m, 1H), 4.44 (t, *J* = 7.1 Hz, 1H), 3.89 (q, *J* = 7.8 Hz, 1H), 3.19 (d, *J* = 4.0 Hz, 1H), 2.73 (q, *J* = 7.6 Hz, 1H), 2.42 – 2.24 (m, 6H), 2.20 (s, 3H), 2.14 – 2.03 (m, 4H), 2.04 – 1.85 (m, 4H), 1.83 – 1.64 (m, 4H), 1.55 (s, 4H), 1.45 (tt, *J* = 14.8, 7.3 Hz, 1H), 1.42 – 1.28 (m, 3H), 1.25 (s, 4H), 1.03 (s, 3H).

^{13}C{^{1}H} NMR (151 MHz, $CDCl_3$) δ 206.7, 206.4, 156.6, 150.6, 140.2, 139.7, 138.1, 136.1, 134.0, 130.4, 130.2, 125.8, 124.5, 97.7, 97.6, 96.2, 96.1, 79.5, 79.4, 71.8, 70.0, 56.0, 42.3, 39.7, 34.4, 30.5, 30.3, 29.3, 27.0, 22.7, 20.9, 20.3, 19.9, 19.0, 17.2.

^{15}N NMR (61 MHz, $CDCl_3$) δ -124.4, -234.3.

^{19}F NMR (377 MHz, $CDCl_3$) δ -72.5, -74.4.

^{31}P NMR (162 MHz, $CDCl_3$) δ -131.1, -135.5, -139.9, -144.3, -148.7, -153.1, -157.5.

HRMS (ESI): *m*/*z* calculated for $C_{32}H_{43}N_3Rh^+$ [M - PF_6^-] 572.2507; found 572.2514.

Complex Rh5bh

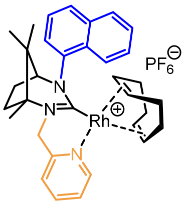
$C_{33}H_{39}F_6N_3PRh$
725,57 g/mol

Following **G.P. B-2**, $[Rh(COD)Cl]_2$ (20 mg, 65 μmol, 1 eq.), **3bh** (40 mg, 77 μmol, 1.2 eq.), and KHMDS (18 mg, 90 μmol, 1.4 eq.) were dissolved in anhydrous THF (5 mL). The reaction mixture was stirred at -78 °C for 1 hour and then slowly warmed to room temperature over 16 hours. Column chromatography (SiO_2, petroleum ether/EtOAc 1:1, followed by DCM/EtOAc 95:5, v/v) of the crude mixture yielded **Rh5bh** as a yellow solid (20 mg, 43%). Mixture of rotamers ca. 60:40, not assigned.

Rf 0.56 (SiO_2, DCM/MeOH 9:1, v/v).

^{1}H NMR (600 MHz, $CDCl_3$) δ 8.32 (d, *J* = 5.2 Hz, 1H), 8.13 – 7.91 (m, 4H), 7.89 – 7.76 (m, 2H), 7.69 – 7.54 (m, 3H), 7.30 – 7.22 (m, 1H), 5.71 (dd, *J* = 52.1, 15.1 Hz, 1H), 5.24 (dd, *J* = 25.4, 15.2 Hz, 1H), 4.30 (dt, *J* = 13.9, 6.9 Hz, 1H), 3.94 (dt, *J* = 27.6, 7.2 Hz, 1H), 3.78 – 3.55 (m, 2H), 2.43 – 2.20 (m, 2H), 2.01 – 1.79 (m, 4H), 1.78 – 1.66 (m, 3H), 1.65 – 1.54 (m, 4H), 1.53 – 1.44 (m, 2H), 1.17 – 1.01 (m, 4H), 0.94 – 0.78 (m, 3H).

^{13}C{^{1}H} NMR (151 MHz, $CDCl_3$) δ 210.4, 210.0, 207.2, 206.9, 157.4, 157.1, 151.3, 150.4, 143.4, 140.0, 139.9, 139.7, 135.0, 134.5, 132.8, 129.7, 129.4, 129.1, 128.6, 128.0, 127.9, 127.7, 127.3, 126.8, 126.3, 126.1, 125.9, 125.7, 125.7, 124.9, 124.7, 122.7, 122.3, 120.2, 98.4, 98.3, 98.0, 98.0, 97.9, 97.8, 97.5, 78.9, 78.8, 78.4, 78.3, 74.4, 71.1, 70.8, 70.2, 70.2, 68.3, 68.2, 56.3, 56.1, 43.3, 41.7, 40.2, 39.0, 36.3, 34.6, 31.8, 30.4, 29.8, 29.6, 28.6, 28.6, 27.0, 25.8, 22.8, 22.5, 18.5, 18.3, 16.7, 16.4.

^{15}N NMR (61 MHz, $CDCl_3$) δ -123.7, -225.7.

^{19}F NMR (377 MHz, $CDCl_3$) δ -72.5, -74.4.

^{31}P NMR (162 MHz, $CDCl_3$) δ -131.1, -135.5, -139.9, -144.3, -148.7, -153.1, -157.5.

HRMS (ESI): *m/z* calculated for $C_{33}H_{39}N_3Rh^+$ [M - PF_6^-] 580.2194; found 580.2202.

Complex Rh5bi

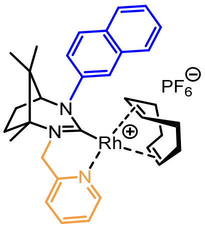
$C_{33}H_{39}F_6N_3PRh$
725,57 g/mol

Following **G.P. B-2**, $[Rh(COD)Cl]_2$ (30 mg, 97 μmol, 1 eq.), **3bi** (60 mg, 116 μmol, 1.2 eq.), and KHMDS (27 mg, 135 μmol, 1.4 eq.) were dissolved in anhydrous THF (5 mL). The reaction mixture was stirred at -78 °C for 1 hour and then slowly warmed to room temperature over 16 hours. Column chromatography (SiO_2, petroleum ether/EtOAc 1:1, followed by DCM/EtOAc 95:5, v/v) of the crude mixture yielded **Rh5bi** as a yellow solid (63 mg, 90%). Mixture of rotamers ca. 50:50, not assigned. In the spectra we can find every proton from both atropoisomers.

Rf 0.68 (SiO_2, DCM/MeOH 9:1, v/v).

^{1}H NMR (600 MHz, $CDCl_3$) δ 8.47 (d, *J* = 5.2 Hz, 1H), 8.17 (d, *J* = 5.2 Hz, 1H), 8.11 (d, *J* = 8.2 Hz, 1H), 8.07 (td, *J* = 7.6, 1.5 Hz, 1H), 8.04 (d, *J* = 8.4 Hz, 1H), 8.03 – 7.98 (m, 2H), 7.96 (d, *J* = 8.2 Hz, 1H), 7.94 (d, *J* = 7.6 Hz, 1H), 7.86 (d, *J* = 7.6 Hz, 1H), 7.82 – 7.77 (m, 3H), 7.65 (td, *J* = 6.8, 5.8, 1.2 Hz, 1H), 7.63 – 7.59 (m, 3H), 7.60 – 7.55 (m, 2H), 7.46 (dd, *J* = 6.5, 0.9 Hz, 1H), 7.28 (d, *J* = 8.4 Hz, 1H), 7.25 (dd, *J* = 8.4, 1.0 Hz, 1H), 5.76 (d, *J* = 15.1 Hz, 1H), 5.67 (d, *J* = 15.2 Hz, 1H), 5.26 (d, *J* = 15.2 Hz, 1H), 5.22 (d, *J* = 15.2 Hz, 1H), 4.88 – 4.83 (m, 1H), 4.30 (dt, *J* = 13.8, 6.9 Hz, 2H), 3.97 (q, *J* = 7.4 Hz, 1H), 3.91 (t, *J* = 7.2 Hz, 1H), 3.74 (d, *J* = 4.4 Hz, 1H), 3.70 (dd, *J* = 13.7, 5.7 Hz, 1H), 3.57 (d, *J* = 4.6 Hz, 1H), 2.40 – 2.27 (m, 3H), 2.24 (q, *J* = 7.6 Hz, 2H), 2.02 – 1.89 (m, 5H), 1.88 – 1.81 (m, 3H), 1.79 – 1.72 (m, 4H), 1.71 – 1.65 (m, 3H), 1.65 – 1.54 (m, 10H), 1.53 – 1.44 (m, 5H), 1.13 (s, 4H), 1.10 – 1.04 (m, 4H), 0.56 (dtd, *J* = 13.6, 9.3, 3.9 Hz, 1H).

^{13}C{^{1}H} NMR (151 MHz, $CDCl_3$) δ 210.7, 210.2, 207.3, 206.9, 157.3, 157.0, 151.2, 150.3, 143.3, 139.9, 139.8, 139.6, 134.9, 134.5, 132.7, 129.6, 129.3, 129.0, 128.5, 127.9, 127.8, 127.6, 127.2, 126.8, 126.2, 126.0, 125.8, 125.6, 125.6, 124.9, 124.6, 122.6, 122.2, 120.1, 98.3, 98.2, 97.9, 97.8, 97.7, 97.4, 78.8, 78.7, 78.4, 78.3, 74.3, 71.0, 70.7, 70.1, 68.2, 68.2, 56.2, 56.1, 43.2, 41.6, 40.2, 38.9, 36.2, 34.5, 31.7, 30.4, 29.7, 29.5, 28.5, 28.5, 26.9, 25.7, 22.7, 22.4, 18.5, 18.2, 16.6, 16.3.

^{15}N NMR (61 MHz, $CDCl_3$) δ -124.7, -229.3.

^{19}F NMR (377 MHz, $CDCl_3$) δ -72.5, -74.4.

^{31}P NMR (162 MHz, $CDCl_3$) δ -131.1, -135.5, -139.9, -144.3, -148.7, -153.1, -157.5.

HRMS (ESI): *m/z* calculated for $C_{33}H_{39}N_3Rh^+$ [M - PF_6^-] 580.2194; found 580.2170.

Complex Rh5bj

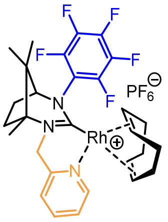

$C_{29}H_{32}F_{11}N_3PRh$
765,46 g/mol

Following **G.P. B-2**, $[Rh(COD)Cl]_2$ (15 mg, 48 µmol, 1 eq.), **3bj** (32 mg, 58 µmol, 1.2 eq.), and KHMDS (14 mg, 68 µmol, 1.4 eq.) were dissolved in anhydrous THF (5 mL). The reaction mixture was stirred at -78 °C for 1 hour and then slowly warmed to room temperature over 16 hours. Column chromatography (SiO_2, petroleum ether/EtOAc 1:1, followed by DCM/EtOAc 95:5, v/v) of the crude mixture yielded **Rh5bj** as a yellow solid (23 mg, 62%). Mixture of rotamers ca. 85:15, not assigned.

Rf 0.63 (SiO_2, DCM/MeOH 9:1, v/v).

^{1}H NMR (600 MHz, $CDCl_3$) δ 8.12 (d, *J* = 5.3 Hz, 1H), 8.01 (td, *J* = 7.6, 1.4 Hz, 1H), 7.93 (d, *J* = 7.6 Hz, 1H), 7.48 (td, *J* = 7.2, 5.3, 1.4 Hz, 1H), 5.82 (d, *J* = 15.4 Hz, 1H), 5.30 (d, *J* = 15.5 Hz, 1H), 4.93 (t, *J* = 9.2 Hz, 1H), 4.49 (t, *J* = 7.0 Hz, 1H), 4.14 (q, *J* = 7.4 Hz, 1H), 3.22 (s, 1H), 2.65 (q, *J* = 7.2 Hz, 1H), 2.57 – 2.42 (m, 1H), 2.25 (dp, *J* = 9.5, 5.6, 5.2 Hz, 1H), 2.19 – 1.97 (m, 4H), 1.88 – 1.75 (m, 3H), 1.68 (ddp, *J* = 24.8, 14.4, 8.1 Hz, 1H), 1.60 – 1.49 (m, 5H), 1.13 (s, 3H), 1.05 (s, 3H).

^{13}C{^{1}H} NMR (151 MHz, $CDCl_3$) δ 200.9, 156.3, 150.1, 140.2, 125.7, 125.4, 100.4, 99.0, 81.6, 81.5, 72.7, 72.0, 71.0, 70.9, 56.3, 42.9, 39.5, 34.0, 31.1, 30.5, 30.1, 27.0, 22.4, 17.5, 16.1.

^{15}N NMR (61 MHz, $CDCl_3$) δ -125.7, -218.7

^{19}F NMR (377 MHz, $CDCl_3$) δ -72.6, -74.5, -145.2, -145.3, -145.5, -145.5, -145.5, -145.5, -145.6, -145.6, -153.3, -153.3, -153.4, -160.4, -160.4, -160.4, -160.5, -160.5, -160.5, -160.6, -160.6, -160.7, -160.7, -160.7.

^{31}P NMR (162 MHz, $CDCl_3$) δ -131.8, -135.7, -140.1, -144.5, -148.9, -153.3, -158.8.

HRMS (ESI): *m/z* calculated for $C_{29}H_{32}F_5N_3Rh^+$ [M - PF_6^-] 620.1571; found 620.1590.

Complex Rh5bk

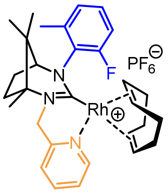

$C_{30}H_{38}F_7N_3PRh$
707,52 g/mol

Following **G.P. B-2**, $[Rh(COD)Cl]_2$ (30 mg, 97 µmol, 1 eq.), **3bk** (48 mg, 97 µmol, 1 eq.), and KHMDS (21 mg, 106 µmol, 1.1 eq.) were dissolved in anhydrous THF (5 mL). The reaction mixture was stirred at -78 °C for 1 hour and then slowly warmed to room temperature over 16 hours. Column chromatography (SiO_2, petroleum ether/EtOAc 1:1, followed by DCM/EtOAc 95:5, v/v) of the crude mixture yielded **Rh5bk** as a yellow solid (47 mg, 70%). Mixture of rotamers ca. 75:25, not assigned.

Rf 0.63 (SiO_2, DCM/MeOH 9:1, v/v).

^{1}H NMR (600 MHz, CD_2Cl_2) δ 8.26 – 8.20 (m, 1H), 8.07 – 7.99 (m, 1H), 7.83 – 7.71 (m, 1H), 7.61 – 7.50 (m, 1H), 7.47 – 7.39 (m, 1H), 7.36 (d, *J* = 7.8 Hz, 1H), 7.32 – 7.16 (m, 1H), 7.14 – 7.07 (m, 1H), 5.96 – 5.84 (m, 1H), 5.18 – 5.10 (m, 1H), 4.97 – 4.77 (m, 1H), 4.64 – 4.42 (m, 1H), 4.07 – 3.96 (m, 1H), 3.34 – 3.20 (m, 1H), 2.81 – 2.71 (m, 1H), 2.54 – 2.37 (m, 1H), 2.37 – 2.31 (m, 3H), 2.20 – 2.04 (m, 3H), 2.02 – 1.94 (m, 1H), 1.92 – 1.81 (m, 1H), 1.80 – 1.70 (m, 2H), 1.70 – 1.61 (m, 1H), 1.60 – 1.53 (m, 5H), 1.22 (d, *J* = 2.9 Hz, 3H), 1.08 (s, 3H).

^{13}C{^{1}H} NMR (151 MHz, CD_2Cl_2) δ 207.0, 206.6, 158.9, 157.2, 156.6, 156.5, 150.8, 150.6, 139.7, 139.2, 138.5, 136.5, 132.8, 132.7, 129.6, 129.5, 129.4, 129.3, 129.0, 128.2, 127.4, 126.9, 125.9, 125.3, 124.3, 124.0, 114.3, 114.2, 114.0, 113.8, 99.2, 99.1, 98.9, 98.8, 97.0, 97.0, 81.1, 81.0, 79.3, 79.2, 73.2, 71.3, 71.3, 70.8, 70.6, 60.3, 56.0, 55.8, 42.8, 42.2, 39.8, 39.3, 34.6, 34.3, 30.8, 30.4, 29.9, 29.7, 29.1, 29.1, 26.9, 26.7, 22.3, 22.2, 20.8, 19.4, 19.3, 18.3, 18.2, 17.5, 17.4, 16.7, 16.5, 14.0.

^{15}N NMR (61 MHz, CD_2Cl_2) δ -123.8, -229.9.

^{19}F NMR (377 MHz, CD_2Cl_2) δ -72.1, -72.1, -72.1, -72.1, -72.1, -72.1, -74.0, -74.0, -74.0, -74.0, -116.6, -116.6, -117.8, -120.2, -120.3.

^{31}P NMR (162 MHz, CD_2Cl_2) δ -131.4, -135.8, -140.2, -144.6, -148.9, -153.3, -157.7.

HRMS (ESI): *m/z* calculated for $C_{30}H_{38}FN_3Rh^+$ [M - PF_6^-] 562.2099; found 562.2095.

Complex Rh5bl

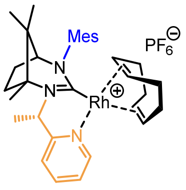

$C_{33}H_{45}F_6N_3PRh$
731,61 g/mol

Following **G.P. B-2**, $[Rh(COD)Cl]_2$ (30 mg, 97 µmol, 1 eq.), **3bl** (100 mg, 193 µmol, 2 eq.), and KHMDS (58 mg, 290 µmol, 3 eq.) were dissolved in anhydrous THF (5 mL). The reaction mixture was stirred at -78 °C for 1 hour and then slowly warmed to room temperature over 16 hours. Column chromatography (SiO_2, petroleum ether/EtOAc 1:1, followed by DCM/pentane/Et_2O 7:1:2, v/v) of the crude mixture yielded **Rh5bl** as a yellow solid (35 mg, 50%). The product was designated as a single rotamer.

Rf 0.63 (SiO_2, DCM/MeOH 9:1, v/v).

^{1}H NMR (600 MHz, CD_2Cl_2) δ 8.30 (d, *J* = 5.6 Hz, 1H), 7.94 (td, *J* = 7.7, 1.6 Hz, 1H), 7.56 (d, *J* = 7.7 Hz, 1H), 7.41 (ddd, *J* = 7.2, 5.6, 1.4 Hz, 1H), 7.10 (s, 1H), 6.94 (s, 1H), 5.42 (q, *J* = 7.2 Hz, 1H), 4.50 (t, *J* = 6.4 Hz, 1H), 4.38 (t, *J* = 7.4 Hz, 1H), 3.76 (q, *J* = 8.1 Hz, 1H), 3.10 (d, *J* = 5.2 Hz, 1H), 2.99 (d, *J* = 7.2 Hz, 3H), 2.77 (q, *J* = 7.0 Hz, 1H), 2.43 (s, 3H), 2.41 – 2.34 (m, 3H), 2.33 (s, 3H), 2.32 – 2.25 (m, 2H), 2.24 (s, 3H), 2.23 – 2.16 (m, 1H), 2.05 – 1.99 (m, 3H), 1.98 – 1.92 (m, 1H), 1.51 – 1.43 (m, 4H), 1.44 – 1.32 (m, 1H), 0.92 (s, 3H), 0.48 (s, 3H).

^{13}C{^{1}H} NMR (151 MHz, CD_2Cl_2) δ 204.2, 203.9, 159.5, 150.7, 143.5, 139.7, 138.6, 137.4, 134.8, 134.4, 131.1, 130.7, 130.2, 125.3, 124.5, 96.9, 96.8, 92.3, 92.2, 81.2, 81.1, 79.0, 79.0, 74.6, 72.4, 60.6, 40.4, 40.3, 33.4, 33.2, 30.1, 29.3, 27.1, 26.3, 23.2, 21.9, 21.3, 20.5, 20.4, 18.9, 16.9.

^{15}N NMR (61 MHz, CD_2Cl_2) δ -130.8, -218.7.

^{19}F NMR (377 MHz, CD_2Cl_2) δ -72.2, -72.2, -74.1, -74.1.

^{31}P NMR (162 MHz, CD_2Cl_2) δ -131.4, -135.8, -140.2, -144.6, -149.0, -153.4, -157.7.

HRMS (ESI): *m*/*z* calculated for $C_{33}H_{45}N_3Rh^+$ [M - PF_6^-] 586.2669; found 586.2665.

Complex Rh5bm

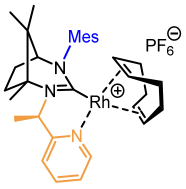

$C_{33}H_{45}F_6N_3PRh$
731,61 g/mol

Following **G.P. B-2**, $[Rh(COD)Cl]_2$ (30 mg, 97 µmol, 1 eq.), **3bm** (50 mg, 97 µmol, 1 eq.), and KHMDS (21 mg, 106 µmol, 1.1 eq.) were dissolved in anhydrous THF (5 mL). The reaction mixture was stirred at -78 °C for 1 hour and then slowly warmed to room temperature over 16 hours. Column chromatography (SiO_2, petroleum ether/EtOAc 1:1, followed by Et_2O/DCM 7:3, v/v) of the crude mixture yielded **Rh5bm** as a yellow solid (44 mg, 62%). The product was designated as a single rotamer.

Rf 0.25 (SiO_2, Et_2O/DCM 7:3, v/v).

^{1}H NMR (600 MHz, CD_2Cl_2) δ 8.16 (d, *J* = 5.5 Hz, 1H), 7.99 (td, *J* = 7.7, 1.6 Hz, 1H), 7.69 (d, *J* = 7.4 Hz, 1H), 7.47 (ddd, *J* = 7.5, 5.6, 1.4 Hz, 1H), 7.12 (s, 1H), 6.99 (s, 1H), 5.32 (q, 1H), 4.62 (td, *J* = 8.0, 3.6 Hz, 1H), 4.46 (t, *J* = 7.5 Hz, 1H), 3.90 (q, *J* = 8.0 Hz, 1H), 3.22 (d, *J* = 2.8 Hz, 1H), 3.09 (q, *J* = 7.4 Hz, 1H), 2.70 (d, *J* = 7.2 Hz, 3H), 2.36 (s, 3H), 2.34 – 2.27 (m, 2H), 2.25 (s, 3H), 2.08 (dddd, *J* = 14.3, 10.2, 8.0, 4.3 Hz, 1H), 1.97 (dddd, *J* = 29.4, 14.6, 9.3, 3.6 Hz, 2H), 1.90 – 1.83 (m, 5H), 1.78 – 1.66 (m, 4H), 1.63 – 1.54 (m, 4H), 1.22 (s, 3H), 1.07 (s, 3H).

^{13}C{^{1}H} NMR (151 MHz, CD_2Cl_2) δ 206.6, 206.3, 159.8, 150.4, 141.4, 140.0, 137.8, 135.6, 133.9, 130.5, 130.4, 125.7, 124.7, 98.1, 98.1, 92.9, 92.9, 80.4, 80.3, 75.8, 75.7, 72.4, 71.2, 61.2, 42.3, 38.9, 33.1, 29.7, 29.3, 29.2, 27.3, 24.9, 22.8, 20.5, 20.1, 20.0, 18.6, 16.1, -3.4.

^{15}N NMR (61 MHz, CD_2Cl_2) δ -130.8, -222.2.

^{19}F NMR (377 MHz, CD_2Cl_2) δ -72.1, -74.0.

^{31}P NMR (162 MHz, CD_2Cl_2) δ -131.4, -135.8, -140.2, -140.2, -144.6, -148.9, -153.3, -157.7.

HRMS (ESI): *m*/*z* calculated for $C_{33}H_{45}N_3Rh^+$ [M - PF_6^-] 586.2669; found 586.2652.

Complex Rh5bn

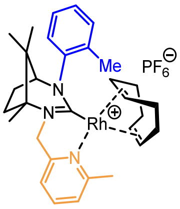

$C_{31}H_{41}F_6N_3PRh$
703,56 g/mol

Following **G.P. B-2**, $[Rh(COD)Cl]_2$ (15 mg, 48.5 µmol, 1 eq.), **3bn** (29 mg, 58 µmol, 1.2 eq.), and KHMDS (14 mg, 67.7 µmol, 1.4 eq.) were dissolved in anhydrous THF (5 mL). The reaction mixture was stirred at -78 °C for 1 hour and then slowly warmed to room temperature over 16 hours. Column chromatography (SiO_2, petroleum ether/EtOAc 1:1, followed by DCM/EtOAc 95:5, v/v) of the crude mixture yielded **Rh5bn** as a yellow solid (32 mg, 95%). Mixture of rotamers ca. 85:15, not assigned.

Rf 0.66 (SiO_2, DCM/MeOH 9:1, v/v).

^{1}H NMR (600 MHz, $CDCl_3$) δ 7.91 – 7.78 (m, 1H), 7.71 – 7.56 (m, 1H), 7.50 – 7.27 (m, 4H), 7.07 – 6.98 (m, 1H), 6.50 – 6.21 (m, 1H), 5.42 – 5.13 (m, 1H), 4.81 – 4.57 (m, 1H), 4.39 – 4.20 (m, 1H), 3.98 – 3.76 (m, 1H), 3.32 – 3.21 (m, 1H), 2.86 – 2.75 (m, 3H), 2.72 – 2.60 (m, 1H), 2.37 – 2.19 (m, 4H), 2.19 – 2.10 (m, 1H), 2.00 – 1.75 (m, 3H), 1.74 – 1.55 (m, 4H), 1.54 (s, 3H), 1.52 – 1.38 (m, 2H), 1.34 – 1.28 (m, 1H), 1.24 (s, 3H), 1.04 (s, 3H).

^{13}C{^{1}H} NMR (151 MHz, $CDCl_3$) δ 210.0, 209.7, 160.5, 160.3, 157.6, 157.0, 146.2, 143.5, 139.8, 139.5, 133.1, 132.5, 132.4, 129.1, 128.9, 128.1, 126.9, 126.5, 126.0, 125.8, 125.1, 122.6, 122.4, 98.6, 98.6, 95.3, 95.2, 94.4, 74.8, 74.7, 73.4, 71.6, 71.5, 70.6, 70.0, 69.7, 56.1, 42.9, 40.8, 40.0, 39.3, 32.9, 32.8, 32.4, 30.3, 30.0, 30.0, 29.7, 29.6, 27.5, 27.0, 26.5, 26.4, 22.7, 22.2, 20.3, 18.4, 18.2, 17.7, 16.4, 14.9.

^{15}N NMR (61 MHz, $CDCl_3$) δ -120.8, -225.0.

^{19}F NMR (377 MHz, $CDCl_3$) δ -72.7, -74.6.

^{31}P NMR (162 MHz, $CDCl_3$) δ -131.2, -135.6, -140.0, -144.4, -148.8, -153.2, -157.6.

HRMS (ESI): *m/z* calculated for $C_{31}H_{41}N_3Rh^+$ [M - PF_6^-] 558.2356; found 558.2368.

Complex Rh5bo

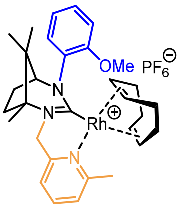

$C_{31}H_{41}F_6N_3OPRh$
719,56 g/mol

Following **G.P. B-2**, $[Rh(COD)Cl]_2$ (15 mg, 48.5 µmol, 1 eq.), **3bo** (29.5 mg, 58 µmol, 1.2 eq.), and KHMDS (14 mg, 67.7 µmol, 1.4 eq.) were dissolved in anhydrous THF (5 mL). The reaction mixture was stirred at -78 °C for 1 hour and then slowly warmed to room temperature over 16 hours. Column chromatography (SiO_2, petroleum ether/EtOAc 1:1, followed by DCM/EtOAc 95:5, v/v) of the crude mixture yielded **Rh5bo** as a yellow solid (32.5 mg, 93%). Mixture of rotamers ca. 75:25, not assigned.

Rf 0.64 (SiO_2, DCM/MeOH 9:1, v/v).

^{1}H NMR (600 MHz, $CDCl_3$) δ 7.89 – 7.80 (m, 1H), 7.68 – 7.53 (m, 1H), 7.52 – 7.41 (m, 1H), 7.41 – 7.31 (m, 1H), 7.18 – 7.11 (m, 2H), 7.10 – 7.01 (m, 1H), 6.38 – 6.17 (m, 1H), 5.30 – 5.10 (m, 1H), 4.83 – 4.67 (m, 1H), 4.42 – 4.19 (m, 1H), 3.99 – 3.79 (m, 4H), 3.44 – 3.17 (m, 1H), 2.82 – 2.72 (m, 3H), 2.70 (t, *J* = 6.8 Hz, 1H), 2.43 – 2.24 (m, 2H), 2.24 – 2.06 (m, 1H), 2.01 – 1.85 (m, 2H), 1.83 – 1.74 (m, 1H), 1.73 – 1.64 (m, 2H), 1.62 – 1.53 (m, 2H), 1.52 – 1.41 (m, 4H), 1.40 – 1.34 (m, 1H), 1.13 (s, 3H), 1.01 (s, 3H).

^{13}C{^{1}H} NMR (151 MHz, $CDCl_3$) δ 207.9, 207.6, 205.0, 160.3, 160.2, 157.8, 157.1, 153.8, 153.6, 139.8, 139.5, 135.8, 133.4, 130.1, 129.1, 128.9, 125.7, 125.6, 125.1, 122.4, 122.3, 120.7, 120.4, 112.4, 112.2, 98.3, 97.9, 97.9, 96.1, 96.0, 95.0, 75.5, 75.4, 72.9, 71.3, 70.9, 70.8, 70.4, 69.7, 56.2, 55.9, 55.8, 55.5, 42.7, 40.5, 40.2, 39.3, 32.9, 32.4, 30.5, 30.2, 29.9, 29.7, 29.5, 28.1, 27.5, 27.0, 26.5, 26.4, 22.7, 22.1, 22.1, 17.6, 17.3, 16.3, 15.3.

^{15}N NMR (61 MHz, $CDCl_3$) δ -120.8, -228.0.

^{19}F NMR (377 MHz, $CDCl_3$) δ -72.8, -74.7.

^{31}P NMR (162 MHz, $CDCl_3$) δ -131.2, -135.6, -140.0, -144.4, -148.8, -153.2, -157.6.

HRMS (ESI): *m/z* calculated for $C_{31}H_{41}N_3ORh^+$ [M - PF_6^-] 574.2305; found 574.2317.

Complex Rh5bp

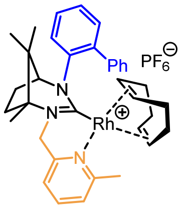

$C_{36}H_{43}F_6N_3PRh$
765,63 g/mol

Following **G.P. B-2**, $[Rh(COD)Cl]_2$ (15 mg, 48.5 µmol, 1 eq.), **3bp** (32 mg, 58 µmol, 1.2 eq.), and KHMDS (14 mg, 67.7 µmol, 1.4 eq.) were dissolved in anhydrous THF (5 mL). The reaction mixture was stirred at -78 °C for 1 hour and then slowly warmed to room temperature over 16 hours. Column chromatography (SiO_2, petroleum ether/EtOAc 1:1, followed by DCM/EtOAc 95:5, v/v) of the crude mixture yielded **Rh5bp** as a yellow solid (21 mg, 57%). Mixture of rotamers ca. 7:3, not assigned.

Rf 0.58 (SiO_2, DCM/MeOH 9:1, v/v).

^{1}H NMR (600 MHz, $CDCl_3$) δ 7.84 (dt, *J* = 32.5, 7.7 Hz, 1H), 7.64 (d, *J* = 7.4 Hz, 1H), 7.61 (td, *J* = 7.6, 1.6 Hz, 1H), 7.59 – 7.52 (m, 1H), 7.49 – 7.36 (m, 5H), 7.34 (dd, *J* = 7.5, 1.6 Hz, 1H), 7.27 – 7.20 (m, 2H), 6.52 (t, *J* = 15.4 Hz, 1H), 5.28 (dd, *J* = 51.8, 15.3 Hz, 1H), 4.67 – 4.49 (m, 1H), 4.45 – 4.22 (m, 1H), 4.15 – 3.97 (m, 1H), 3.20 (t, *J* = 7.0 Hz, 1H), 3.01 (d, *J* = 11.4 Hz, 4H), 2.48 – 2.29 (m, 1H), 2.31 – 2.14 (m, 1H), 2.14 – 2.04 (m, 1H), 2.00 (ddt, *J* = 17.2, 9.0, 3.6 Hz, 1H), 1.95 – 1.83 (m, 1H), 1.83 – 1.64 (m, 2H), 1.66 – 1.45 (m, 3H), 1.43 (s, 3H), 1.19 – 1.10 (m, 1H), 0.90 – 0.81 (m, 1H), 0.63 (s, 3H), 0.60 (s, 3H).

^{13}C{^{1}H} NMR (151 MHz, $CDCl_3$) δ 208.7, 208.4, 160.4, 160.2, 157.4, 157.0, 145.2, 142.7, 139.8, 139.4, 138.6, 137.1, 136.8, 132.8, 132.5, 129.4, 128.9, 128.8, 128.5, 128.4, 128.3, 128.3, 128.0, 127.8, 127.5, 125.8, 125.6, 125.1, 122.7, 122.4, 99.6, 99.6, 99.3, 99.3, 92.7, 92.7, 76.1, 76.1, 74.9, 74.9, 74.2, 74.1, 74.0, 73.7, 72.0, 70.7, 70.0, 56.5, 56.4, 42.1, 40.9, 39.5, 39.4, 33.2, 32.6, 30.6, 30.5, 30.3, 30.2, 29.9, 29.8, 27.7, 27.1, 26.7, 26.6, 22.4, 22.1, 18.1, 17.4, 16.5, 14.8.

^{15}N NMR (61 MHz, $CDCl_3$) δ -121.7, -223.1.

^{19}F NMR (377 MHz, $CDCl_3$) δ -72.8, -74.7.

^{31}P NMR (162 MHz, $CDCl_3$) δ -131.2, -135.6, -140.0, -144.4, -148.8, -153.2, -157.6.

HRMS (ESI): *m/z* calculated for $C_{36}H_{43}N_3Rh^+$ [M - PF_6^-] 620.2507; found 620.2520.

Complex Rh5br

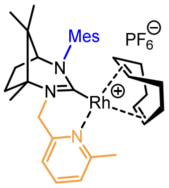

$C_{33}H_{45}F_6N_3PRh$
731,2310 g/mol

Following **G.P. B-2**, $[Rh(COD)Cl]_2$ (20 mg, 64.5 µmol, 1 eq.), **3br** (34 mg, 64.5 µmol, 1 eq.), and KHMDS (15 mg, 71 µmol, 1.2 eq.) were dissolved in anhydrous THF (5 mL). The reaction mixture was stirred at -78 °C for 1 hour and then slowly warmed to room temperature over 16 hours. Column chromatography (SiO_2, petroleum ether/EtOAc 1:1, followed by DCM/EtOAc 95:5, v/v) of the crude mixture yielded **Rh5br** as a yellow solid (25 mg, 53%).

Rf 0.53 (SiO_2, DCM/MeOH 9:1, v/v).

^{1}H NMR (600 MHz, $CDCl_3$) δ 7.84 (t, *J* = 7.7 Hz, 1H), 7.62 (d, *J* = 7.3 Hz, 1H), 7.33 (d, *J* = 7.3 Hz, 1H), 7.07 (s, 1H), 6.93 (s, 1H), 6.49 (d, *J* = 15.5 Hz, 1H), 5.24 (d, *J* = 15.5 Hz, 1H), 4.41 (s, 2H), 4.05 – 4.00 (m, 1H), 3.17 (d, *J* = 4.0 Hz, 1H), 3.03 (t, *J* = 7.2 Hz, 1H), 2.82 (s, 3H), 2.38 – 2.31 (m, 4H), 2.23 – 2.16 (m, 4H), 1.97 (s, 3H), 1.96 – 1.85 (m, 3H), 1.82 – 1.74 (m, 3H), 1.74 – 1.64 (m, 2H), 1.62 – 1.55 (m, 4H), 1.46 – 1.38 (m, 1H), 1.30 (s, 3H), 1.04 (s, 3H).

^{13}C{^{1}H} NMR (151 MHz, $CDCl_3$) δ 205.3, 205.0, 160.1, 156.8, 140.4, 139.7, 138.1, 135.8, 134.4, 130.5, 130.4, 125.7, 122.2, 98.6, 98.5, 92.5, 92.5, 75.3, 75.2, 75.2, 75.1, 72.5, 70.2, 55.3, 42.2, 40.0, 33.4, 30.9, 30.1, 29.9, 27.1, 27.0, 22.9, 20.9, 19.8, 19.5, 19.0, 17.4.

^{15}N NMR (61 MHz, $CDCl_3$) δ -119.1, -227.5.

^{19}F NMR (377 MHz, $CDCl_3$) δ -72.7, -74.6.

^{31}P NMR (162 MHz, $CDCl_3$) δ -131.2, -135.6, -140.0, -144.4, -148.8, -153.2, -157.6.

HRMS (ESI): *m/z* calculated for $C_{33}H_{45}N_3Rh^+$ [M - PF_6^-] 586.2663; found 586.2669.

Complex Rh5bs

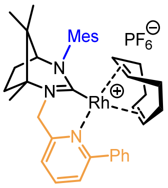

$C_{38}H_{47}F_6N_3PRh$
793,68 g/mol

Following **G.P. B-2**, $[Rh(COD)Cl]_2$ (20 mg, 64.5 µmol, 1 eq.), **3bs** (38 mg, 64.5 µmol, 1 eq.), and KHMDS (15 mg, 71 µmol, 1.2 eq.) were dissolved in anhydrous THF (5 mL). The reaction mixture was stirred at -78 °C for 1 hour and then slowly warmed to room temperature over 16 hours. Column chromatography (SiO_2, petroleum ether/EtOAc 1:1, followed by DCM/EtOAc 95:5, v/v) of the crude mixture yielded **Rh5bs** as a yellow solid (35 mg, 67%).

Rf 0.57 (SiO_2, DCM/MeOH 9:1, v/v).

^{1}H NMR (600 MHz, $CDCl_3$) δ 8.50 (d, *J* = 7.5 Hz, 2H), 8.08 (t, *J* = 7.8 Hz, 1H), 7.89 (dd, *J* = 7.5, 1.0 Hz, 1H), 7.78 (dd, *J* = 8.0, 1.0 Hz, 1H), 7.61 (dt, *J* = 7.8, 1.6 Hz, 1H), 7.49 – 7.42 (m, 2H), 7.01 (s, 1H), 6.97 (s, 1H), 6.53 (d, *J* = 15.2 Hz, 1H), 5.51 (d, *J* = 15.2 Hz, 1H), 4.29 (t, *J* = 7.6 Hz, 1H), 4.09 (q, *J* = 6.7 Hz, 1H), 3.67 (t, *J* = 7.2 Hz, 1H), 3.17 (d, *J* = 3.7 Hz, 1H), 2.47 (dtd, *J* = 15.1, 9.0, 8.3, 6.3 Hz, 1H), 2.43 (s, 3H), 2.39 (q, *J* = 7.3 Hz, 1H), 2.23 (s, 3H), 2.20 – 2.11 (m, 1H), 2.02 (dtd, *J* = 16.4, 9.7, 6.6 Hz, 1H), 1.95 – 1.82 (m, 4H), 1.74 – 1.70 (m, 3H), 1.66 (ddd, *J* = 19.1, 9.5, 4.8 Hz, 1H), 1.59 (s, 4H), 1.56 – 1.50 (m, 1H), 1.41 (s, 3H), 1.27 – 1.21 (m, 1H), 1.17 (h, *J* = 8.8 Hz, 1H), 1.10 (s, 3H).

^{13}C{^{1}H} NMR (151 MHz, $CDCl_3$) δ 205.1, 204.8, 159.4, 156.7, 141.2, 140.4, 138.5, 137.1, 136.6, 135.3, 131.2, 130.6, 130.6, 129.5, 128.3, 124.8, 123.5, 99.3, 99.3, 90.0, 89.9, 75.3, 75.2, 72.7, 70.9, 56.0, 42.8, 39.8, 34.7, 31.1, 30.4, 29.3, 25.8, 23.3, 21.1, 19.4, 19.4, 18.9, 17.6.

^{15}N NMR (61 MHz, $CDCl_3$) δ -229.5, -132.8.

^{19}F NMR (377 MHz, $CDCl_3$) δ -72.7, -74.6.

^{31}P NMR (162 MHz, $CDCl_3$) δ -131.2, -135.6, -140.0, -144.4, -148.8, -153.2, -157.6.

HRMS (ESI): *m*/*z* calculated for $C_{38}H_{47}N_3Rh^+$ [M - PF_6^-] 648.2820; found 648.2832.

Complex Rh 5bt

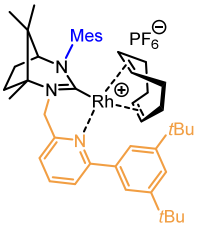

$C_{46}H_{63}F_6N_3PRh$
905,90 g/mol

Following **G.P. B-2**, $[Rh(COD)Cl]_2$ (20 mg, 64.5 µmol, 1 eq.), **3bt** (45 mg, 64.5 µmol, 1 eq.), and KHMDS (15 mg, 71 µmol, 1.1 eq.) were dissolved in anhydrous THF (5 mL). The reaction mixture was stirred at -78 °C for 1 hour and then slowly warmed to room temperature over 16 hours. Column chromatography (SiO_2, petroleum ether/EtOAc 1:1, followed by DCM/EtOAc 95:5, v/v) of the crude mixture yielded **Rh5bt** as a yellow solid (12 mg, 21%).

Rf 0.63 (SiO_2, DCM/MeOH 9:1, v/v).

^{1}H NMR (600 MHz, $CDCl_3$) δ 8.05 (t, *J* = 7.7 Hz, 1H), 7.91 (dd, *J* = 7.6, 1.2 Hz, 1H), 7.56 (t, *J* = 1.7 Hz, 1H), 7.44 (dd, *J* = 7.8, 1.0 Hz, 1H), 7.34 (d, *J* = 1.7 Hz, 2H), 7.05 (s, 1H), 6.99 (s, 1H), 6.54 (d, *J* = 15.2 Hz, 1H), 5.47 (d, *J* = 15.2 Hz, 1H), 4.33 (t, *J* = 6.4 Hz, 1H), 4.18 (d, *J* = 6.8 Hz, 1H), 3.38 (s, 1H), 3.06 (d, *J* = 3.8 Hz, 1H), 2.58 (q, *J* = 7.3 Hz, 1H), 2.37 (s, 3H), 2.35 – 2.26 (m, 2H), 2.23 (s, 3H), 2.20 – 2.12 (m, 2H), 1.88 (s, 3H), 1.85 – 1.73 (m, 5H), 1.73 – 1.66 (m, 4H), 1.64 – 1.58 (m, 2H), 1.32 (d, *J* = 11.1 Hz, 21H), 1.06 (s, 3H).

^{13}C{^{1}H} NMR (151 MHz, $CDCl_3$) δ 206.6, 206.3, 163.1, 156.5, 151.1, 140.7, 140.1, 138.5, 137.6, 135.5, 134.2, 131.5, 130.9, 127.1, 124.0, 123.8, 98.1, 93.9, 75.9, 75.8, 73.2, 72.1, 72.0, 70.6, 55.9, 42.6, 39.7, 35.2, 33.3, 32.1, 31.5, 30.5, 30.3, 30.1, 29.8, 29.8, 29.5, 26.8, 23.4, 22.8, 21.0, 19.8, 19.6, 18.6, 17.4, 14.3.

^{15}N NMR (61 MHz, $CDCl_3$) δ -123.8, -226.7.

^{19}F NMR (377 MHz, $CDCl_3$) δ -72.7, -74.6.

^{31}P NMR (162 MHz, $CDCl_3$) δ -135.6, -140.0, -144.4, -148.8, -153.2.

HRMS (ESI): *m*/*z* calculated for $C_{46}H_{63}N_3Rh^+$ [M - PF_6^-] 760.4072; found 760.4081.

Complex RhPh$_2$(SIMes)

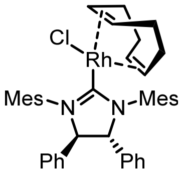

$C_{41}H_{46}ClN_2Rh$
705,19 g/mol

Following **G.P. C**, $[Rh(COD)Cl]_2$ (20 mg, 41 µmol, 1 eq.), **Ph$_2$SIMes BF$_4$** (53 mg, 97 µmol, 2.4 eq.), and KHMDS (19 mg, 97 µmol, 2.4 eq.) were dissolved in anhydrous THF (3 mL). The reaction mixture was stirred at -78 °C for 1 hour and then slowly warmed to room temperature over 16 hours. Column chromatography (SiO_2, petroleum ether/EtOAc 1:1, v/v) of the crude mixture yielded **RhPh$_2$(SIMes)** as a yellow solid (58 mg, 99%). The product was designated as a single rotamer. The ligand was prepared in the standard way described in the literature.[21]

Rf 0.12 (SiO_2, DCM/MeOH 9:1, v/v).

^{1}H NMR (600 MHz, $CDCl_3$) δ 7.38 – 7.33 (m, 2H), 7.33 – 7.23 (m, 8H), 7.01 (s, 1H), 6.95 (s, 1H), 6.85 (s, 1H), 6.78 (s, 1H), 5.65 (d, *J* = 11.0 Hz, 1H), 5.57 (d, *J* = 11.0 Hz, 1H), 4.56 (td, *J* = 7.6, 3.4 Hz, 1H), 4.44 (q, *J* = 7.6, 6.8 Hz, 1H), 3.51 (s, 1H), 3.28 (t, *J* = 6.8 Hz, 1H), 2.90 (s, 3H), 2.57 (s, 3H), 2.36 (s, 3H), 2.31 (s, 3H), 2.30 (s, 3H), 1.95 (s, 3H), 1.94 – 1.88 (m, 2H), 1.70 – 1.61 (m, 4H), 1.58 – 1.48 (m, 2H).

^{13}C{^{1}H} NMR (151 MHz, $CDCl_3$) δ 212.0, 211.7, 139.3, 138.2, 137.5, 137.5, 136.8, 136.7, 135.6, 135.5, 134.4, 134.3, 130.3, 130.1, 129.1, 129.1, 128.9, 128.6, 128.5, 128.5, 128.3, 98.0, 98.0, 96.5, 96.4, 73.6, 73.4, 70.3, 70.2, 65.4, 65.3, 34.2, 31.2, 29.4, 26.8, 22.3, 21.0, 21.0, 20.9, 20.0, 19.7.

^{15}N NMR (61 MHz, $CDCl_3$) δ -227.5.

IR (ATR neat) ṽ =: 2915, 2875, 2830, 1604, 1479, 1455, 1403, 1377, 1342, 1306 cm^{-1}.

HRMS (ESI): *m*/*z* calculated for $C_{41}H_{46}N_2Rh^+$ [M – Cl^-] 669.2711; found 669.2716.

Complex Rh(SIMes)

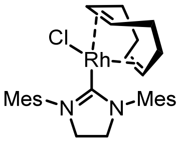

$C_{29}H_{38}ClN_2Rh$
552,99 g/mol

Following **G.P. C**, $[Rh(COD)Cl]_2$ (38 mg, 77 µmol, 1 eq.), **SIMesBr** (66 mg, 170 µmol, 2.2 eq.), and KHMDS (36 mg, 170 µmol, 2.4 eq.) were dissolved in anhydrous THF (3 mL). The reaction mixture was stirred at -78 °C for 1 hour and then slowly warmed to room temperature over 16 hours. Column chromatography (SiO_2, petroleum ether/EtOAc 1:1, v/v) of the crude mixture yielded **Rh(SIMes)** as a yellow solid (86 mg, 99%). Mixture of atropoisomers ca. 6:4, not assigned. The ligand was prepared in the standard way described in the literature.[22]

^{1}H NMR (400 MHz, $CDCl_3$) δ 6.97 – 6.82 (m, 4H), 4.55 – 4.33 (m, 2H), 3.86 – 3.68 (m, 4H), 3.49 – 3.19 (m, 2H), 2.52 (s, 6H), 2.25 (s, 12H), 1.75 – 1.63 (m, 4H), 1.51 – 1.33 (m, 4H).

^{13}C{^{1}H} NMR (101 MHz, $CDCl_3$) δ 213.0, 212.8, 212.5, 212.3, 138.5, 138.3, 137.9, 137.9, 136.4, 136.4, 135.3, 135.3, 130.1, 130.0, 128.5, 128.5, 97.3, 97.2, 96.8, 96.7, 68.7, 68.6, 67.7, 67.6, 51.6, 51.5, 32.7, 32.6, 31.6, 29.8, 28.5, 28.2, 22.7, 21.1, 20.8, 20.0, 18.5, 18.4, 14.2.

The spectral data were consistent with the literature.[22]

5.1.3 Synthesis of starting materials for Asymmetric Ring Opening reaction (**G. P. C**)

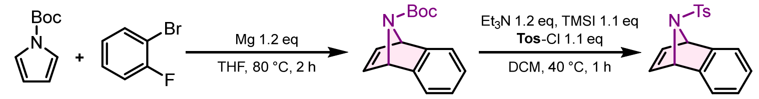

6a 6b

***tert*-butyl (1*R*,4*S*)-1,4-dihydro-1,4-epiminonaphthalene-9-carboxylate (6a)**

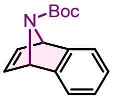

$C_{15}H_{17}NO_2$
243,31 g/mol

A 250 mL three-neck round-bottom flask equipped with a condenser, magnetic stir bar, and dropping funnel was charged with activated magnesium turnings (861 mg, 35.88 mmol, 1.2 eq.). The flask was flame-dried under vacuum, flushed with argon, and allowed to cool. *N*-Boc-pyrrole (5 g, 29.90 mmol, 1 eq.) in 100 mL of dry THF was then added to the flask and heated to a gentle reflux. A solution of *o*-Fluorobromobenzene (3.92 mL, 32.90 mmol, 1.1 eq.) in 50 mL of dry THF was introduced dropwise under an argon atmosphere over 30 minutes, followed by refluxing for additional 2 hours. The initiation of the reaction was indicated by the solution becoming turbid, then turning yellow. After cooling and evaporating THF, the mixture was poured into a flask containing 500 mL of an aqueous solution

of ammonium chloride (300 g) and concentrated ammonium hydroxide (10 mL, 25.0% w/w NH_3). The aqueous layer was extracted with petroleum ether (3 × 50 mL), and the combined organic layers were dried over anhydrous magnesium sulfate, yielding a dark oil. This crude product was purified by column chromatography (SiO_2 petroleum ether/EtOAc 95:5 v/v) producing compound **6a** as a yellow crystalline solid (3.75 g, 52% yield).

Rf 0.53 (SiO_2, Petroleum ether/EtOAc 9:1, v/v).

^{1}H NMR (400 MHz, $CDCl_3$) δ 7.25 (s, 2H), 6.95 (dd, *J* = 5.1, 3.0 Hz, 4H), 5.48 (s, 2H), 1.37 (s, 9H).

^{13}C{^{1}H} NMR (101 MHz, $CDCl_3$) δ 155.2, 148.3, 125.0, 80.6, 28.2.

The spectral data were consistent with the literature.[23]

General Procedure for the Synthesis of *N*-Ts Compound 6b

In a round-bottom flask **6a** (1 eq.) was dissolved in 100 mL of DCM. To this solution, Et_3N (1.2 eq.) was added, and the reaction mixture was heated to reflux. TMSI (1.1 eq.) was added dropwise over 10 minutes, followed by an additional 15 minutes of heating under reflux. The progress of the reaction was monitored by TLC, confirming complete consumption of the *N*-Boc starting material. After reflux, the reaction mixture was cooled to 0 °C using an ice bath. Methanol (1 mL) was added dropwise, and the mixture was stirred at 0 °C for 10 minutes. Sulfonyl chloride (1 eq.) was then added, and the reaction mixture was allowed to gradually warm to room temperature. The reaction was stirred for an additional hour at room temperature. After the reaction time, 50 mL of water was added to the mixture, along with sufficient DCM to dissolve any precipitates formed. The organic and aqueous layers were separated, and the aqueous phase was extracted twice with DCM. The combined organic layers were dried over anhydrous $MgSO_4$, filtered, and concentrated under reduced pressure. The crude product was purified by column chromatography, resulting in the *N*-protected compound as a crystalline solid.

(*1R,4S*)-9-tosyl-1,4-dihydro-1,4-epiminonaphthalene (6b)

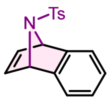

$C_{17}H_{15}NO_2S$
297,37 g/mol

Following above procedure, **6b** (1.2 g, 4.93 mmol, 1 eq.) was dissolved in 100 mL of DCM, followed by the addition of Et_3N (0.820 mL, 5.92 mmol, 1.2 eq.). The mixture was heated to reflux, and TMSI (0.770 mL, 5.42 mmol, 1.1 eq.) was added dropwise over 10 minutes. The reaction was then refluxed for an additional 15 minutes. The reaction mixture was cooled to 0 °C, and MeOH (1 mL) was added dropwise. After stirring at 0 °C for 10 minutes, 4-Toluenesulfonyl chloride (940 mg, 4.93 mmol, 1 eq.) was added, and the reaction was allowed to warm to room temperature, stirring for an additional hour. Water (50 mL) and DCM were added to dissolve any precipitates, and the organic and aqueous layers were separated. The aqueous layer was extracted twice with DCM, and the combined organic layers were dried over $MgSO_4$ and concentrated. Column chromatography (SiO_2, petroleum ether/EtOAc 6:4, v/v) gave **6b** as dark brown solid (845 mg, 58%).

Rf 0.34 (SiO_2, Petroleum ether/EtOAc 1:1, v/v).

^{1}H NMR (400 MHz, $CDCl_3$) δ 7.46 (d, *J* = 7.9 Hz, 2H), 7.09 (d, *J* = 7.9 Hz, 2H), 7.02 (dd, *J* = 5.2, 3.1 Hz, 2H), 6.82 – 6.71 (m, 4H), 5.44 (t, *J* = 1.5 Hz, 2H), 2.33 (s, 3H).

^{13}C{^{1}H} NMR (101 MHz, $CDCl_3$) δ 155.2, 148.3, 143.6, 142.4, 125.0, 121.1, 80.6, 66.2, 28.2.

The spectral data were consistent with the literature.[24]

General Procedure for the Synthesis of bicyclic alkene G. P. C

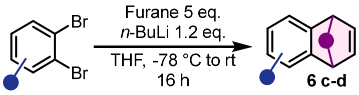

A 100 mL flame-dried RBF, fitted with a magnetic stir bar and containing dibromoarene (1 eq.), was placed under vacuum for 10 minutes and then purged with argon three times. Next, freshly distilled furan (5 eq.) dissolved in THF (100 mL) was added, and the mixture was cooled to -78 °C. *n*-BuLi (2.5 M in hexanes, 1.2 eq) was then added dropwise while keeping the reaction temperature stable. The mixture was stirred for 2 hours at -78 °C before being allowed to warm up to room temperature. After 16 hours, the reaction was quenched with a saturated NH_4Cl solution. The organic layer was separated, and the aqueous layer was extracted three times with DCM. The combined organic layers were washed with brine, dried over anhydrous $MgSO_4$, concentrated, and then purified via column chromatography on silica gel to obtain the desired starting material.

6,7-dimethyl-1,4-dihydro-1,4-epoxynaphthalene (6c)

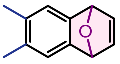

$C_{12}H_{12}O$
172,23 g/mol

Following **G. P. C**, 1,2-dibromo-4,5-dimethylbenzene (4.0 g, 15.2 mmol, 1 eq.) was dissolved in 100 mL of THF and cooled down to -78 °C. To the reaction mixture, furan (5.5 mL, 75.8 mmol, 5 eq.) was added, and it was stirred for an additional 10 minutes. Then, *n*-BuLi (7.2 mL, 18.2 mmol, 1.2 eq.) was added to the reaction mixture. The reaction mixture was stirred for 2 hours at -78 °C before being allowed to warm up to room temperature. A saturated solution of NH_4Cl (2 mL) was added, followed by the work-up of the reaction mixture. Column chromatography of the crude product (SiO_2, petroleum ether/EtOAc 9:1, v/v) yielded **6c** as a light-yellow solid (760 mg, 30%).

Rf 0.43 (SiO_2, Petroleum ether/EtOAc 9:1, v/v).

^{1}H NMR (400 MHz, $CDCl_3$) δ 7.09 (s, 2H), 7.03 (s, 2H), 5.70 (s, 2H), 2.24 (s, 6H).

^{13}C{^{1}H} NMR (101 MHz, $CDCl_3$) δ 146.7, 143.2, 132.6, 122.2, 82.3, 19.9.

The spectral data were consistent with the literature.[25]

6,7-dimethoxy-1,4-dihydro-1,4-epoxynaphthalene (6d)

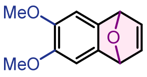

$C_{12}H_{12}O_3$
204,23 g/mol

Following **G. P. C**, 1,2-dibromo-4,5-dimethoxybenzene (4.0 g, 13.5 mmol, 1 eq.) was dissolved in 100 mL of THF and cooled down to -78 °C. To the reaction mixture, furan (4.9 mL, 67.6 mmol, 5 eq.) was added, and it was stirred for an additional 10 minutes. Then, *n*-BuLi (6.5 mL, 16.2 mmol, 1.2 eq.) was added to the reaction mixture. The reaction mixture was stirred for 2 hours at -78 °C before being allowed to warm up to room temperature. A saturated solution of NH_4Cl (2 mL) was added, followed by the work-up of the reaction mixture. Column chromatography of the crude product (SiO_2, petroleum ether/EtOAc 9:1, v/v) yielded **6d** as a brown solid (630 mg, 23%).

Rf 0.25 (SiO_2, Petroleum ether/EtOAc 9:1, v/v).

^{1}H NMR (400 MHz, $CDCl_3$) δ 7.04 (t, J = 1.0 Hz, 1H), 6.97 (s, 1H), 5.68 (t, J = 1.0 Hz, 1H), 3.85 (s, 2H).

^{13}C{^{1}H} NMR (101 MHz, $CDCl_3$) δ 146.0, 143.5, 141.9, 106.9, 82.7, 56.6.

The spectral data were consistent with the literature.[25]

2,3,6,9-tetrahydro-6,9-epoxynaphtho[2,3-*b*][1,4]dioxine (6e)

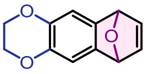

$C_{12}H_{10}O_3$
202,21 g/mol

Following **G. P. C**, 6,7-dibromo-2,3-dihydrobenzo[*b*][1,4]dioxine (2.0 g, 6.8 mmol, 1 eq.) was dissolved in 70 mL of THF and cooled down to -78 °C. To the reaction mixture, furan (2.5 mL, 34.0 mmol, 5 eq.) was added, and it was stirred for an additional 10 minutes. Then, *n*-BuLi (3.3 mL, 8.2 mmol, 1.2 eq.) was added to the reaction mixture. The reaction mixture was stirred for 2 hours at -78 °C before being allowed to warm up to room temperature. A saturated solution of

NH_4Cl (2 mL) was added, followed by the work-up of the reaction mixture. Column chromatography of the crude product (SiO_2, petroleum ether/EtOAc 9:1, v/v) yielded **6e** as a brown solid (680 mg, 50%).

Rf 0.27 (SiO_2, Petroleum ether/EtOAc 9:1, v/v).

^{1}H NMR (400 MHz, $CDCl_3$) δ 6.98 (t, *J* = 1.0 Hz, 2H), 6.81 (s, 2H), 5.62 (s, 2H), 4.19 (s, 4H).

^{13}C{^{1}H} NMR (101 MHz, $CDCl_3$) δ 142.9, 141.5, 139.8, 111.3, 82.2, 64.4.

IR (ATR neat) ṽ =: 3039, 2997, 2927, 1605, 1459, 1392, 1342 cm^{-1}.

HRMS (ESI): *m*/*z* calculated for $C_{12}H_{10}NaO_3^+$ [M + Na^+] 225.0522; found 225.0525.

6,7-difluoro-1,4-dihydro-1,4-epoxynaphthalene (6f)

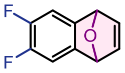

$C_{10}H_6F_2O$
180,15 g/mol

Following **G. P. C**, 1,2-dibromo-4,5-difluorobenzene (2.0 g, 7.36 mmol, 1 eq.) was dissolved in 30 mL of Et_2O and cooled down to -78 °C. To the reaction mixture, furan (5.1 mL, 70.36 mmol, 10 eq.) was added, and it was stirred for an additional 10 minutes. Then, *n*-BuLi (3.83 mL, 9.56 mmol, 1.2 eq.) was added to the reaction mixture. The reaction mixture was stirred for 2 hours at -78 °C before being allowed to warm up to room temperature. A saturated solution of NH_4Cl (2 mL) was added, followed by the work-up of the reaction mixture. Column chromatography of the crude product (SiO_2, petroleum ether/EtOAc 9:1, v/v) yielded **6f** as a light-yellow solid (900 mg, 68%).

Rf 0.22 (SiO_2, Petroleum ether/EtOAc 95:5, v/v).

^{1}H NMR (400 MHz, $CDCl_3$) δ 7.07 (t, *J* = 7.7 Hz, 2H), 7.03 (s, 2H), 5.69 (s, 2H).

^{13}C{^{1}H} NMR (101 MHz, $CDCl_3$) δ 148.55 (d, *J* = 14.9 Hz), 146.09 (d, *J* = 14.9 Hz), 145.15 (t, *J* = 4.7 Hz), 143.13, 110.94 (t, *J* = 7.4 Hz), 110.71 (t, *J* = 7.4 Hz), 82.13.

The spectral data were consistent with the literature.[26]

5.2 General Procedure: Catalytic Asymmetric Ring Opening Reaction (**G.P. E**).

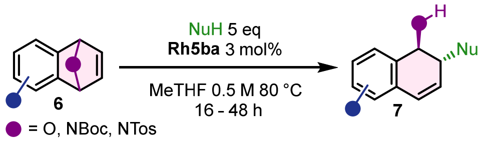

General Procedure E: To a flame-dried test vial (5 mL), catalyst (2 - 4 mg) prepared from the stock solution ($CHCl_3$) is transferred. The vial containing the catalyst is then placed in a glovebox after the solvent has evaporated. Next, oxabenzonorbornadiene (1 eq.) is added, followed by corresponding aniline or phenol (5 eq), and finally, the solvent (0.5 M) is added. The vial is sealed with a Teflon cap and the temperature is set to 80 °C (heating mantle) for 16 to 24 hours. After the reaction is complete, the vial is removed from the glovebox and subsequent manipulations are performed under ambient air. The crude mixture is transferred to a flask and the solvent is evaporated under reduced pressure. The internal standard (IS) CH_2Br_2 is added to the residue, and a crude NMR is prepared to determine the conversion and yield of the reaction. Subsequently, column chromatography is carried out to purify the product and determine the isolated yield.

(1*R*,2*R*)-2-(methyl(phenyl)amino)-1,2-dihydronaphthalen-1-ol (7aa)

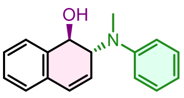

$C_{17}H_{17}NO$
251,33 g/mol

Following **G. P. E** vial containing **Rh5ba** (4 mg, 3 mol%) was charged to glovebox and into reaction vial added oxabenzonorbornadiene (28 mg, 0.19 mmol, 1 eq.) and *N*-methylaniline (105 µL, 0.97 mmol, 5 eq.) followed by dry MeTHF (0.39 mL) reaction mixture was stirred at 80 °C for 16 h. After reaction was completed vial was taken from glovebox and concentrated under reduced pressure, from the crude reaction mixture prepared NMR sample. Column chromatography (SiO_2, Pentane/Et_2O 15:1, followed by Et_2O, v/v) of the crude mixture gave **7aa** as a light yellow oil (49 mg, 99%). The *ee* was determined to be 85 % using chiral HPLC (OD-H, *i*-propanol/*n*-hexane = 10/90, flow rate = 0.5 mL/min, l = 254 nm) t_R = 20.8 (major), 22.8 (minor).

Rf 0.18 (SiO_2, pentane/Et_2O 9:1, v/v).

^{1}H NMR (400 MHz, $CDCl_3$) δ 7.59 – 7.49 (m, 1H), 7.32 – 7.21 (m, 4H), 7.16 – 7.07 (m, 1H), 6.96 (d, *J* = 8.2 Hz, 2H), 6.80 (t, *J* = 7.3 Hz, 1H), 6.58 (dd, *J* = 9.8, 2.5 Hz, 1H), 5.92 (dd, *J* = 9.8, 3.0 Hz, 1H), 5.09 (d, *J* = 9.8 Hz, 1H), 4.73 (dt, *J* = 9.7, 2.7 Hz, 1H), 2.83 (s, 3H), 2.40 (s, 1H).

^{13}C{^{1}H} NMR (101 MHz, $CDCl_3$) δ 150.4, 136.6, 132.1, 129.8, 129.4, 128.2, 128.0, 127.9, 126.6, 125.7, 118.2, 114.8, 70.2, 63.6, 33.5.

$[\alpha]^{25}_D$ = +30 (c = 1.00, $CHCl_3$).

The spectral data were consistent with the literature.[5]

***ee* determination:**

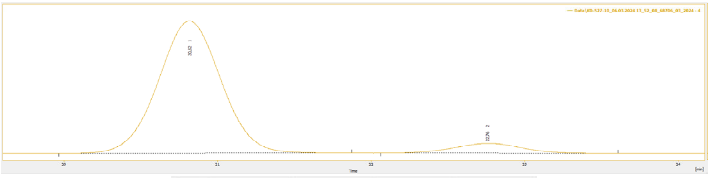

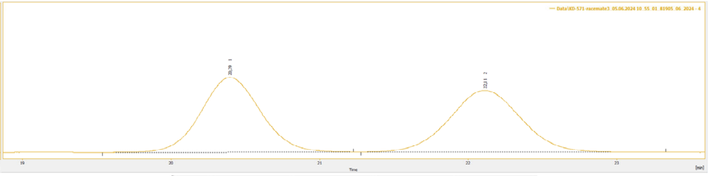

	Reten. Time [min]	Area [mAU.s]	Height [mAU]	Area [%]
1	20,817	26851,094	940,270	92,6
2	22,758	2144,857	69,858	7,4
	Total	28995,951	1010,128	100,0

	Reten. Time [min]	Area [mAU.s]	Height [mAU]	Area [%]
1	20,394	25965,371	932,866	50,1
2	22,111	25881,438	771,345	49,9
	Total	51846,809	1704,211	100,0

(1*R*,2*R*)-2-((4-chlorophenyl)(methyl)amino)-1,2-dihydronaphthalen-1-ol (7ab)

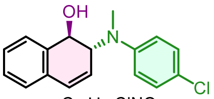

$C_{17}H_{16}ClNO$
285,77 g/mol

Following **G. P. E** vial containing **Rh5ba** (4 mg, 3 mol%) was charged to glovebox and into reaction vial added oxabenzonorbornadiene (28 mg, 0.19 mmol, 1 eq.) and 4-Chloro-*N*-methylaniline (117 µL, 0.97 mmol, 5 eq.) followed by dry MeTHF (0.39 mL) reaction mixture was stirred at 80 °C for 16 h. After reaction was completed vial was taken from glovebox and concentrated under reduced pressure, from the crude reaction mixture prepared NMR sample. Column chromatography (SiO_2, Hex/Et_2O 9:1, followed by Hex/Et_2O 3:1, v/v) of the crude mixture gave **7ab** as a light yellow oil (53 mg, 96%). The *ee* was determined to be 82 % using chiral HPLC (AD-H, *i*-propanol/*n*-hexane = 5/95, flow rate = 0.5 mL/min, l = 254 nm) t_R = 30.9 (major), 35.6 (minor).

Rf 0.25 (SiO_2, Hex/Et_2O 3:1, v/v).

^{1}H NMR (400 MHz, $CDCl_3$) δ 7.56 – 7.49 (m, 1H), 7.32 – 7.26 (m, 2H), 7.23 – 7.16 (m, 2H), 7.16 – 7.11 (m, 1H), 6.90 – 6.84 (m, 2H), 6.61 (dd, *J* = 9.8, 2.5 Hz, 1H), 5.90 (dd, *J* = 9.8, 3.1 Hz, 1H), 5.07 (d, *J* = 9.4 Hz, 1H), 4.67 (dt, *J* = 9.5, 2.8 Hz, 1H), 2.81 (s, 3H), 2.39 (s, 1H).

^{13}C{^{1}H} NMR (101 MHz, $CDCl_3$) δ 148.8, 136.4, 132.0, 130.1, 129.1, 128.3, 128.2, 127.3, 126.7, 125.8, 123.0, 115.8, 70.2, 63.7, 33.7.

$[\alpha]^{25}_D$ = +40 (c = 0.10, $CHCl_3$).

The spectral data were consistent with the literature.[27]

***ee* determination:**

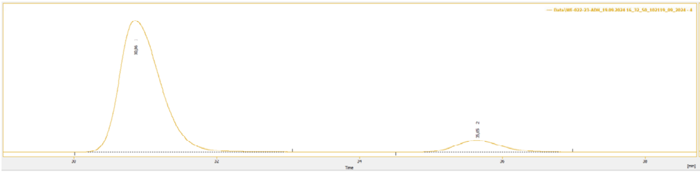

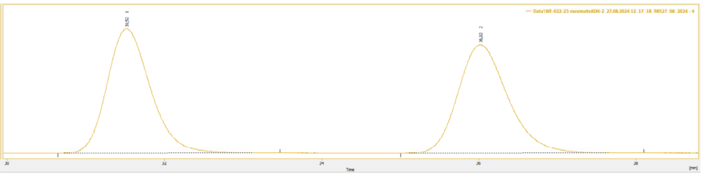

	Reten. Time [min]	Area [mAU.s]	Height [mAU]	Area [%]
1	30,864	48328,951	1214,483	91,1
2	35,647	4748,740	108,484	8,9
	Total	53077,691	1322,967	100,0

	Reten. Time [min]	Area [mAU.s]	Height [mAU]	Area [%]
1	31,518	23108,408	601,634	50,0
2	36,023	23130,727	524,982	50,0
	Total	46239,135	1126,616	100,0

(1*R*,2*R*)-2-((4-methoxyphenyl)(methyl)amino)-1,2-dihydronaphthalen-1-ol (7ac)

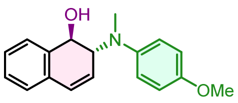

$C_{18}H_{19}NO_2$
281,3550 g/mol

Following **G. P. E** vial containing **Rh5ba** (4 mg, 3 mol%) was charged to glovebox and into reaction vial added oxabenzonorbornadiene (28 mg, 0.19 mmol, 1 eq.) and 4-Methoxy-*N*-methylanilin (133 mg, 0.97 mmol, 5 eq.) followed by dry MeTHF (0.39 mL) reaction mixture was stirred at 80 °C for 16 h. After reaction was completed vial was taken from glovebox and concentrated under reduced pressure, from the crude reaction mixture prepared NMR sample. Column chromatography (SiO_2, Pentane/Et_2O 9:1, followed by Pentane/Et_2O 4:1, followed by Pentane/Et_2O 1:4, v/v) of the crude mixture gave **7ac** as a light yellow oil (45 mg, 83%). The *ee* was determined to be 84 % using chiral HPLC (OD-H, *i*-propanol/*n*-hexane = 10/90, flow rate = 0.5 mL/min, l = 254 nm) t_R = 23.8 (minor), 28.8 (major).

Rf 0.07 (SiO_2, Pentane/Et_2O 9:1, v/v).

^{1}H NMR (400 MHz, $CDCl_3$) δ 7.60 – 7.52 (m, 1H), 7.30 – 7.22 (m, 2H), 7.09 (dd, *J* = 6.8, 2.0 Hz, 1H), 6.96 – 6.91 (m, 2H), 6.86 – 6.81 (m, 2H), 6.55 (dd, *J* = 9.8, 2.5 Hz, 1H), 5.94 (dd, *J* = 9.8, 2.8 Hz, 1H), 5.08 (d, *J* = 10.7 Hz, 1H), 4.52 (dt, *J* = 10.4, 2.7 Hz, 1H), 3.76 (s, 3H), 2.78 (s, 3H), 2.61 (s, 1H).

^{13}C{^{1}H} NMR (101 MHz, $CDCl_3$) δ 153.1, 144.9, 136.8, 132.1, 129.7, 128.1, 127.9, 127.6, 126.5, 125.4, 117.6, 114.8, 69.8, 65.5, 55.8, 34.1.

$[\alpha]^{25}_D$ = +35 (c = 1.00, $CHCl_3$).

The spectral data were consistent with the literature.[5]

***ee* determination:**

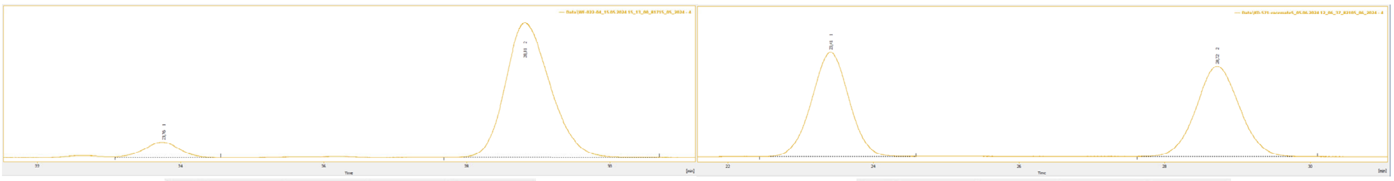

	Reten. Time [min]	Area [mAU.s]	Height [mAU]	Area [%]
1	23,762	4245,784	122,636	8,2
2	28,813	47464,146	1099,420	91,8
	Total	51709,930	1222,055	100,0

	Reten. Time [min]	Area [mAU.s]	Height [mAU]	Area [%]
1	23,413	12806,479	359,787	49,8
2	28,721	12904,206	311,828	50,2
	Total	25710,685	671,615	100,0

(1*R*,2*R*)-2-(methyl(*o*-tolyl)amino)-1,2-dihydronaphthalen-1-ol (7ba)

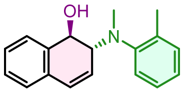

$C_{18}H_{19}NO$
265,36 g/mol

Following **G. P. E** vial containing **Rh5ba** (4 mg, 3 mol%) was charged to glovebox and into reaction vial added oxabenzonorbornadiene (28 mg, 0.19 mmol, 1 eq.) and *N*-methyl-2-methylaniline (120 µL, 0.97 mmol, 5 eq.) followed by dry MeTHF (0.39 mL) reaction mixture was stirred at 80 °C for 16 h. After reaction was completed vial was taken from glovebox and concentrated under reduced pressure, from the crude reaction mixture prepared NMR sample. Column chromatography (SiO_2, Hex/Et_2O 7:1, followed by Hex/Et_2O 4:1, v/v) of the crude mixture gave **7ba** as a light yellow oil (46 mg, 90%). The *ee* was determined to be 80 % using chiral HPLC (OD-H, *i*-propanol/*n*-hexane = 10/90, flow rate = 0.5 mL/min, l = 254 nm) t_R = 12.8 (major), 15.2 (minor).

Rf 0.42 (SiO_2, Hex/Et_2O 3:1, v/v).

^{1}H NMR (400 MHz, $CDCl_3$) δ 7.50 – 7.43 (m, 1H), 7.23 – 7.12 (m, 2H), 7.11 – 7.04 (m, 3H), 7.03 – 6.97 (m, 1H), 6.95 – 6.85 (m, 1H), 6.51 (dd, *J* = 9.8, 2.3 Hz, 1H), 6.04 (dd, *J* = 9.8, 2.8 Hz, 1H), 5.01 (d, *J* = 10.3 Hz, 1H), 3.91 (d, *J* = 10.2 Hz, 1H), 2.64 (s, 3H), 2.26 (s, 3H).

^{13}C{^{1}H} NMR (101 MHz, $CDCl_3$) δ 137.1, 132.7, 132.0, 131.8, 129.8, 128.1, 127.8, 127.3, 126.6, 126.4, 125.3, 123.6, 121.7, 108.6, 69.7, 65.4, 35.6, 19.0.

$[\alpha]^{25}_D$ = -100 (c = 0.10, $CHCl_3$).

IR (ATR neat) ṽ =: 3373, 3031, 2925, 2853, 2798, 1596, 1577, 1489, 1452 cm^{-1}.

HRMS (ESI): *m/z* calculated for $C_{18}H_{19}NNaO^+$ [M + Na^+] 288.1359; found 288.1360.

***ee* determination:**

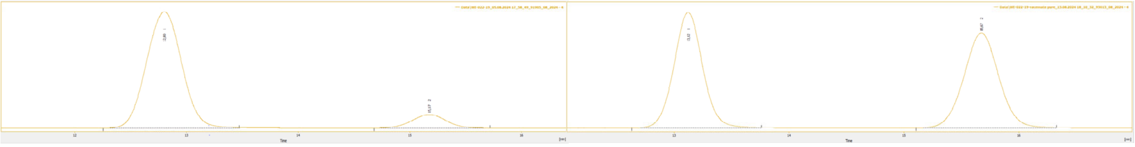

	Reten. Time [min]	Area [mAU.s]	Height [mAU]	Area [%]
1	12,800	27846,797	1261,413	89,9
2	15,172	3140,543	145,115	10,1
	Total	30987,340	1406,529	100,0

	Reten. Time [min]	Area [mAU.s]	Height [mAU]	Area [%]
1	13,123	12830,733	736,601	50,0
2	15,672	12809,168	604,528	50,0
	Total	25639,901	1341,129	100,0

(1*R*,2*R*)-2-(methyl(*m*-tolyl)amino)-1,2-dihydronaphthalen-1-ol (7bb)

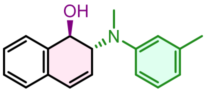

$C_{18}H_{19}NO$
265,36 g/mol

Following **G. P. E** vial containing **Rh5ba** (4 mg, 3 mol%) was charged to glovebox and into reaction vial added oxabenzonorbornadiene (28 mg, 0.19 mmol, 1 eq.) and *N*-methyl-3-methylaniline (121 µL, 0.97 mmol, 5 eq.) followed by dry MeTHF (0.39 mL) reaction mixture was stirred at 80 °C for 16 h. After reaction was completed vial was taken from glovebox and concentrated under reduced pressure, from the crude reaction mixture prepared NMR sample. Column chromatography (SiO_2, Hex/Et_2O 9:1, followed by Hex/Et_2O 3:1, v/v) of the crude mixture gave **7bb** as a light yellow oil (43 mg, 84%). The *ee* was determined to be 83 % using chiral HPLC (OD-H, *i*-propanol/*n*-hexane = 5/95, flow rate = 0.5 mL/min, l = 254 nm) t_R = 25.4 (major), 27.1 (minor).

Rf 0.31 (SiO_2, Hex/Et_2O 3:1, v/v).

^{1}H NMR (400 MHz, $CDCl_3$) δ 7.65 – 7.49 (m, 1H), 7.36 – 7.27 (m, 2H), 7.22 – 7.10 (m, 2H), 6.85 – 6.78 (m, 2H), 6.67 (d, *J* = 7.4 Hz, 1H), 6.61 (dd, *J* = 9.8, 2.5 Hz, 1H), 5.95 (dd, *J* = 9.8, 2.9 Hz, 1H), 5.14 (d, *J* = 9.9 Hz, 1H), 4.76 (dt, *J* = 9.9, 2.7 Hz, 1H), 2.87 (s, 3H), 2.52 (s, 1H), 2.35 (s, 3H).

^{13}C{^{1}H} NMR (101 MHz, $CDCl_3$) δ 150.4, 139.2, 136.7, 132.1, 129.8, 129.2, 128.2, 128.0, 127.8, 126.6, 125.7, 119.4, 115.7, 112.1, 70.2, 63.9, 33.5, 22.0.

$[\alpha]^{25}_D$ = +310 (c = 0.10, $CHCl_3$).

The spectral data were consistent with the literature.[4]

***ee* determination:**

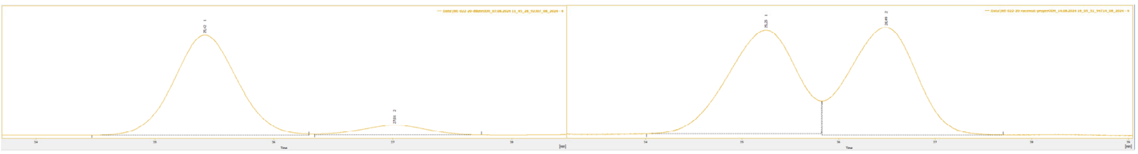

	Reten. Time [min]	Area [mAU.s]	Height [mAU]	Area [%]
1	25,418	15832,037	427,235	91,3
2	27,013	1500,772	39,025	8,7
	Total	17332,809	466,260	100,0

	Reten. Time [min]	Area [mAU.s]	Height [mAU]	Area [%]
1	25,247	4715,988	93,787	49,8
2	26,491	4745,434	97,420	50,2
	Total	9461,422	191,207	100,0

(1*R*,2*R*)-2-(methyl(*p*-tolyl)amino)-1,2-dihydronaphthalen-1-ol (7bc)

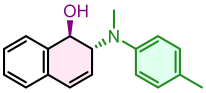

$C_{18}H_{19}NO$
265,36 g/mol

Following **G. P. E** vial containing **Rh5ba** (4 mg, 3 mol%) was charged to glovebox and into reaction vial added oxabenzonorbornadiene (28 mg, 0.19 mmol, 1 eq.) and *N*-methyl-4-methylaniline (122 µL, 0.97 mmol, 5 eq.) followed by dry MeTHF (0.39 mL) reaction mixture was stirred at 80 °C for 16 h. After reaction was completed vial was taken from glovebox and concentrated under reduced pressure, from the crude reaction mixture prepared NMR sample. Column chromatography (SiO_2, Hex/Et_2O 17:1, followed by Hex/Et_2O 9:1, followed by Hex/Et_2O 4:1, v/v) of the crude mixture gave **7bc** as a light yellow oil (49 mg, 96%). The *ee* was determined to be 81 % using chiral HPLC (OD-H, *i*-propanol/*n*-hexane = 5/95, flow rate = 0.5 mL/min, l = 254 nm) t_R = 25.1 (minor), 28.3 (major).

Rf 0.31 (SiO_2, Hex/Et_2O 3:1, v/v).

^{1}H NMR (400 MHz, $CDCl_3$) δ 7.61 – 7.55 (m, 1H), 7.33 – 7.27 (m, 2H), 7.17 – 7.08 (m, 3H), 6.95 – 6.89 (m, 2H), 6.60 (dd, *J* = 9.8, 2.5 Hz, 1H), 5.96 (dd, *J* = 9.8, 2.9 Hz, 1H), 5.13 (d, *J* = 10.0 Hz, 1H), 4.69 (dt, *J* = 10.1, 2.7 Hz, 1H), 2.84 (s, 3H), 2.61 (s, 1H), 2.31 (s, 3H).

^{13}C{^{1}H} NMR (101 MHz, $CDCl_3$) δ 148.2, 136.7, 132.1, 129.9, 129.8, 128.2, 128.0, 127.9, 127.7, 126.5, 125.6, 115.5, 70.0, 64.4, 33.7, 20.4.

$[\alpha]^{25}_D$ = +30 (c = 0.10, $CHCl_3$).

The spectral data were consistent with the literature.[5]

***ee* determination:**

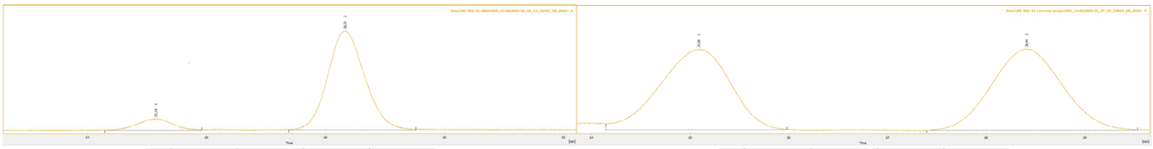

	Reten. Time [min]	Area [mAU.s]	Height [mAU]	Area [%]
1	25,142	383,470	9,705	9,6
2	28,320	3603,997	83,921	90,4
	Total	3987,467	93,626	100,0

	Reten. Time [min]	Area [mAU.s]	Height [mAU]	Area [%]
1	25,084	3967,192	79,742	49,6
2	28,407	4032,076	80,813	50,4
	Total	7999,268	160,555	100,0

(1*R*,2*R*)-2-(indolin-1-yl)-1,2-dihydronaphthalen-1-ol (7ca)

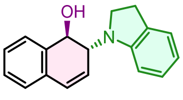

$C_{18}H_{17}NO$
263,34 g/mol

Following **G. P. E** vial containing **Rh5ba** (4 mg, 3 mol%) was charged to glovebox and into reaction vial added oxabenzonorbornadiene (28 mg, 0.19 mmol, 1 eq.) and Indoline (108 µL, 0.97 mmol, 5 eq.) followed by dry MeTHF (0.39 mL) reaction mixture was stirred at 80 °C for 16 h. After reaction was completed vial was taken from glovebox and concentrated under reduced pressure, from the crude reaction mixture prepared NMR sample. Column chromatography (SiO_2, Pentane/Et_2O 7:3, v/v) of the crude mixture gave **7ca** as a light yellow oil (55 mg, 99%). The *ee* was determined to be 78 % using chiral HPLC (OD-H, *i*-propanol/*n*-hexane = 10/90, flow rate = 0.5 mL/min, l = 254 nm) t_R = 21.4 (minor), 23.9 (major).

Rf 0.52 (SiO_2, Pentane/Et_2O 1:1, v/v).

^{1}H NMR (400 MHz, $CDCl_3$) δ 7.65 – 7.59 (m, 1H), 7.36 – 7.28 (m, 2H), 7.17 – 7.07 (m, 3H), 6.75 (td, *J* = 7.4, 1.0 Hz, 1H), 6.66 (dd, *J* = 9.8, 2.4 Hz, 1H), 6.61 (d, *J* = 7.8 Hz, 1H), 5.90 (dd, *J* = 9.8, 2.9 Hz, 1H), 5.11 (d, *J* = 10.3 Hz, 1H), 4.50 (dt, *J* = 10.3, 2.7 Hz, 1H), 3.44 – 3.32 (m, 2H), 3.08 – 2.95 (m, 2H), 2.80 (s, 1H).

^{13}C{^{1}H} NMR (101 MHz, $CDCl_3$) δ 151.0, 136.5, 132.1, 130.8, 130.5, 128.2, 128.0, 127.5, 126.6, 126.6, 125.5, 124.9, 118.6, 108.1, 69.7, 59.7, 48.7, 28.6.

$[\alpha]^{25}_D$ = -68 (c = 0.25, $CHCl_3$).

IR (ATR neat) ṽ =: 3374, 3025, 2921, 2851, 1713, 1604, 1485, 1456, cm^{-1}.

HRMS (ESI): *m*/*z* calculated for $C_{18}H_{17}NNaO^+$ [M + Na^+] 286.1202; found 286.1203.

***ee* determination:**

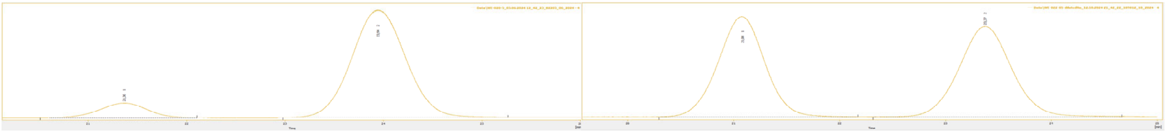

	Reten. Time [min]	Area [mAU.s]	Height [mAU]	Area [%]
1	21,363	5414,294	155,110	11,0
2	23,942	43974,410	1143,647	89,0
	Total	49388,705	1298,757	100,0

	Reten. Time [min]	Area [mAU.s]	Height [mAU]	Area [%]
1	21,082	5951,614	192,922	49,5
2	23,372	6079,717	174,382	50,5
	Total	12031,331	367,304	100,0

(1*R*,2*R*)-2-(3,4-dihydroquinolin-1(2*H*)-yl)-1,2-dihydronaphthalen-1-ol (7cb)

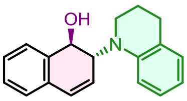

$C_{19}H_{19}NO$
277,37 g/mol

Following **G. P. E** vial containing **Rh5ba** (4 mg, 3 mol%) was charged to glovebox and into reaction vial added oxabenzonorbornadiene (28 mg, 0.19 mmol, 1 eq.) and 1,2,3,4-Tetrahydroquinolin (121 µL, 0.97 mmol, 5 eq.) followed by dry MeTHF (0.39 mL) reaction mixture was stirred at 80 °C for 16 h. After reaction was completed vial was taken from glovebox and concentrated under reduced pressure, from the crude reaction mixture prepared NMR sample. Column chromatography (SiO_2, Hex/Et_2O 9:1, v/v) of the crude mixture gave **7cb** as a light yellow oil (55 mg, 99%). The *ee* was determined to be 84 % using chiral HPLC (OD-H, *i*-propanol/*n*-hexane = 5/95, flow rate = 0.5 mL/min, l = 254 nm) t_R = 26.1 (major), 27.6 (minor).

Rf 0.24 (SiO_2, Hex/Et_2O 3:1, v/v).

^{1}H NMR (400 MHz, $CDCl_3$) δ 7.58 – 7.53 (m, 1H), 7.34 – 7.28 (m, 2H), 7.19 – 7.13 (m, 1H), 7.12 – 7.06 (m, 1H), 7.06 – 7.02 (m, 1H), 6.96 (d, *J* = 8.3 Hz, 1H), 6.73 – 6.63 (m, 2H), 5.96 (dd, *J* = 9.8, 3.3 Hz, 1H), 5.16 (d, *J* = 8.9 Hz, 1H), 4.79 (dt, *J* = 8.9, 2.9 Hz, 1H), 3.30 (dt, *J* = 11.2, 5.6 Hz, 1H), 3.14 (dt, *J* = 11.6, 5.9 Hz, 1H), 2.90 – 2.73 (m, 2H), 2.42 (s, 1H), 1.99 – 1.89 (m, 2H).

$^{13}C\{^1H\}$ NMR (101 MHz, $CDCl_3$) δ 145.2, 136.7, 132.1, 130.0, 129.7, 128.2, 128.2, 127.9, 127.2, 126.7, 126.1, 124.3, 117.2, 112.6, 69.7, 61.4, 44.4, 28.3, 22.6.

$[\alpha]^{25}_D$ = -30 (c = 0.10, $CHCl_3$).

The spectral data were consistent with the literature.[28]

***ee* determination:**

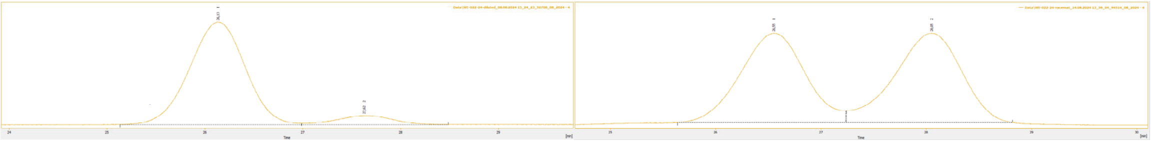

	Reten. Time [min]	Area [mAU.s]	Height [mAU]	Area [%]
1	26,130	4276,621	108,016	91,9
2	27,624	376,662	9,413	8,1
	Total	4653,284	117,429	100,0

	Reten. Time [min]	Area [mAU.s]	Height [mAU]	Area [%]
1	26,548	6447,531	149,897	49,2
2	28,048	6657,675	149,781	50,8
	Total	13105,206	299,678	100,0

(1*R*,2*R*)-2-(2,3,4,5-tetrahydro-1*H*-benzo[*b*]azepin-1-yl)-1,2-dihydronaphthalen-1-ol (7cc)

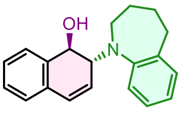

$C_{20}H_{21}NO$
291,39 g/mol

Following **G. P. E** vial containing **Rh5ba** (4 mg, 3 mol%) was charged to glovebox and into reaction vial added oxabenzonorbornadiene (28 mg, 0.19 mmol, 1 eq.) and 2,3,4,5-Tetrahydro-1*H*-1-benzazepine (138 µL, 0.97 mmol, 5 eq.) followed by dry MeTHF (0.39 mL) reaction mixture was stirred at 80 °C for 16 h. After reaction was completed vial was taken from glovebox and concentrated under reduced pressure, from the crude reaction mixture prepared NMR sample. Column chromatography (SiO_2, Hex/Et_2O 9:1, v/v) of the crude mixture gave **7cc** as a light yellow oil (51 mg, 91%). The *ee* was determined to be 85 % using chiral HPLC (OD-H, *i*-propanol/*n*-hexane = 2/98, flow rate = 0.5 mL/min, l = 254 nm) t_R = 16.0 (major), 18.9 (minor).

Rf 0.34 (SiO_2, Hex/Et_2O 3:1, v/v).

^1H NMR (400 MHz, $CDCl_3$) δ 7.61 (d, *J* = 6.8 Hz, 1H), 7.31 – 7.21 (m, 2H), 7.19 – 7.08 (m, 3H), 7.00 – 6.89 (m, 2H), 6.62 (dd, *J* = 9.9, 2.7 Hz, 1H), 6.23 (dd, *J* = 9.9, 2.1 Hz, 1H), 5.05 (d, *J* = 12.2 Hz, 1H), 4.38 (dt, *J* = 12.2, 2.5 Hz, 1H), 3.62 (dt, *J* = 14.2, 4.0 Hz, 1H), 3.25 (s, 1H), 2.90 – 2.82 (m, 2H), 2.71 (t, *J* = 12.4 Hz, 1H), 1.98 – 1.84 (m, 2H), 1.73 – 1.62 (m, 1H), 1.45 – 1.34 (m, 1H).

$^{13}C\{^1H\}$ NMR (101 MHz, $CDCl_3$) δ 152.8, 137.1, 136.9, 131.9, 130.7, 130.4, 129.0, 128.1, 127.5, 127.0, 126.2, 124.7, 122.3, 119.0, 70.4, 66.6, 50.4, 36.2, 31.8, 26.3.

$[\alpha]^{25}_D$ = -230 (c = 0.10, $CHCl_3$).

IR (ATR neat) ṽ =: 3504, 3060, 3030, 2922, 2851, 1737, 1595, 1492, 1451, 1399, 1352 cm^{-1}.

HRMS (ESI): *m*/*z* calculated for $C_{20}H_{21}NNaO^+$ [M + Na^+] 314.1515; found 314.1518.

***ee* determination:**

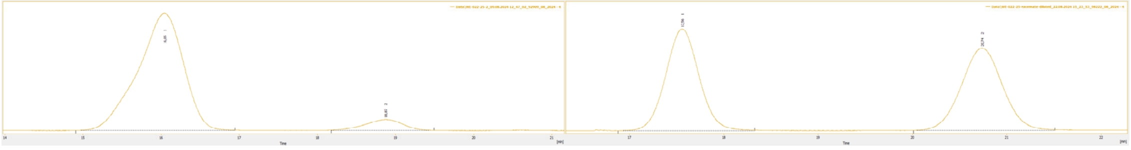

	Reten. Time [min]	Area [mAU.s]	Height [mAU]	Area [%]
1	16,046	31927,560	786,306	92,7
2	18,873	2504,923	74,208	7,3
	Total	34432,483	860,514	100,0

	Reten. Time [min]	Area [mAU.s]	Height [mAU]	Area [%]
1	17,559	4197,415	169,624	50,1
2	20,738	4184,086	137,584	49,9
	Total	8381,502	307,209	100,0

(1*R*,2*R*)-2-(ethyl(phenyl)amino)-1,2-dihydronaphthalen-1-ol (7da)

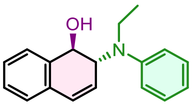

$C_{18}H_{19}NO$
265,3560 g/mol

Following **G. P. E** vial containing **Rh5ba** (4 mg, 3 mol%) was charged to glovebox and into reaction vial added oxabenzonorbornadiene (28 mg, 0.19 mmol, 1 eq.) and *N*-Ethylaniline (122 µL, 0.97 mmol, 5 eq.) followed by dry MeTHF (0.39 mL) reaction mixture was stirred at 80 °C for 16 h. After reaction was completed vial was taken from glovebox and concentrated under reduced pressure, from the crude reaction mixture prepared NMR sample. Column chromatography (SiO_2, Hex/Et_2O 9:1, v/v) of the crude mixture gave **7da** as a light yellow oil (49 mg, 96%). The *ee* was determined to be 81 % using chiral HPLC (OD-H, *i*-propanol/*n*-hexane = 10/90, flow rate = 0.5 mL/min, l = 254 nm) t_R = 14.9 (major), 16.1 (minor).

Rf 0.30 (SiO_2, Hex/Et_2O 3:1, v/v).

^{1}H NMR (400 MHz, $CDCl_3$) δ 7.54 – 7.47 (m, 1H), 7.29 – 7.21 (m, 4H), 7.13 – 7.07 (m, 1H), 6.95 (d, *J* = 8.2 Hz, 2H), 6.77 (t, *J* = 7.3 Hz, 1H), 6.59 (dd, *J* = 9.8, 2.5 Hz, 1H), 5.94 (dd, *J* = 9.8, 3.2 Hz, 1H), 5.09 (d, *J* = 9.2 Hz, 1H), 4.65 (dt, *J* = 9.2, 2.9 Hz, 1H), 3.34 (q, *J* = 7.0 Hz, 2H), 2.44 (s, 1H), 1.14 (t, *J* = 7.0 Hz, 3H).

^{13}C{^{1}H} NMR (101 MHz, $CDCl_3$) δ 148.3, 136.5, 132.1, 129.6, 129.4, 128.7, 128.2, 128.2, 126.7, 126.2, 117.8, 115.0, 70.4, 63.4, 41.7, 14.5.

$[\alpha]^{25}_D$ = -10 (c = 0.10, $CHCl_3$).

The spectral data were consistent with the literature.[4]

***ee* determination:**

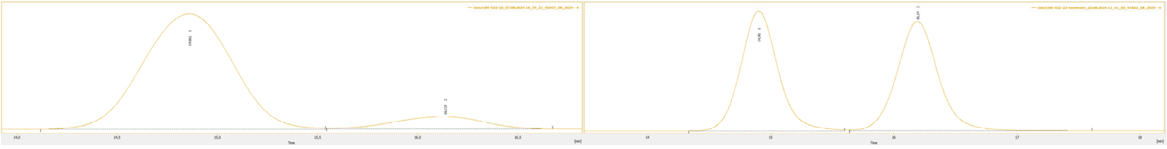

	Reten. Time [min]	Area [mAU.s]	Height [mAU]	Area [%]
1	14,862	5463,407	175,095	90,6
2	16,137	568,965	18,782	9,4
	Total	6032,372	193,877	100,0

	Reten. Time [min]	Area [mAU.s]	Height [mAU]	Area [%]
1	14,903	18366,828	871,608	49,8
2	16,192	18533,110	794,958	50,2
	Total	36899,939	1666,566	100,0

(1*R*,2*R*)-2-(isopropyl(phenyl)amino)-1,2-dihydronaphthalen-1-ol (7db)

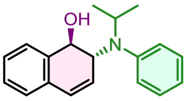

$C_{19}H_{21}NO$
279,38 g/mol

Following **G. P. E** vial containing **Rh5ba** (4 mg, 3 mol%) was charged to glovebox and into reaction vial added oxabenzonorbornadiene (28 mg, 0.19 mmol, 1 eq.) and *N*-Isopropylaniline (140 µL, 0.97 mmol, 5 eq.) followed by dry MeTHF (0.39 mL) reaction mixture was stirred at 80 °C for 16 h. After reaction was completed vial was taken from glovebox and concentrated under reduced pressure, from the crude reaction mixture prepared NMR sample. Column chromatography (SiO_2, Pentane/Et_2O 5:1, v/v) of the crude mixture gave **7db** as a light yellow oil (18 mg, 33%). The *ee* was determined to be 85 % using chiral HPLC (OD-H, *i*-propanol/*n*-hexane = 5/95, flow rate = 0.5 mL/min, l = 254 nm) t_R = 12.0 (major), 13.4 (minor).

Rf 0.46 (SiO_2, Pentane/Et_2O 3:1, v/v).

^{1}H NMR (400 MHz, $CDCl_3$) δ 7.61 – 7.56 (m, 1H), 7.27 – 7.18 (m, 4H), 7.10 (dt, *J* = 8.0, 1.2 Hz, 3H), 6.80 (tt, *J* = 7.2, 1.1 Hz, 1H), 6.49 (dd, *J* = 9.8, 2.9 Hz, 1H), 6.14 (dd, *J* = 9.8, 2.4 Hz, 1H), 5.38 (d, *J* = 11.8 Hz, 1H), 4.41 (dt, *J* = 12.1, 2.7 Hz, 1H), 4.08 (hept, *J* = 6.7 Hz, 1H), 2.52 (s, 1H), 1.34 (d, *J* = 6.7 Hz, 3H), 1.21 (d, *J* = 6.7 Hz, 3H).

^{13}C{^{1}H} NMR (101 MHz, $CDCl_3$) δ 147.8, 137.6, 133.0, 132.3, 128.9, 128.1, 128.0, 127.7, 126.3, 125.2, 119.6, 119.0, 68.6, 60.2, 51.4, 29.8, 22.8, 21.7.

$[\alpha]^{25}_D$ = -24 (c = 0.25, $CHCl_3$).

The spectral data were consistent with the literature.[5]

***ee* determination:**

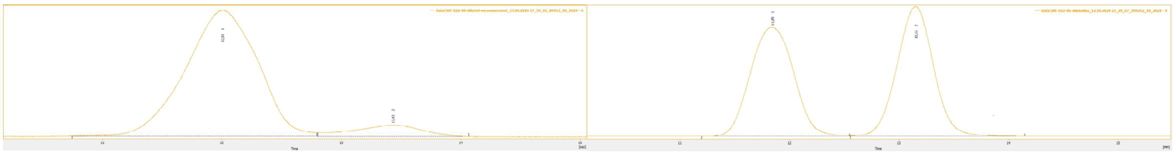

	Reten. Time [min]	Area [mAU.s]	Height [mAU]	Area [%]
1	12,005	10115,836	240,980	92,4
2	13,431	828,150	21,470	7,6
	Total	10943,987	262,451	100,0

	Reten. Time [min]	Area [mAU.s]	Height [mAU]	Area [%]
1	11,845	20773,080	754,107	50,0
2	13,156	20775,669	895,242	50,0
	Total	41548,749	1649,349	100,0

(1*R*,2*R*)-2-(butyl(phenyl)amino)-1,2-dihydronaphthalen-1-ol (7dc)

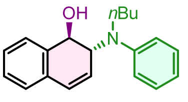

$C_{20}H_{23}NO$
293,41 g/mol

Following **G. P. E** vial containing **Rh5ba** (4 mg, 3 mol%) was charged to glovebox and into reaction vial added oxabenzonorbornadiene (28 mg, 0.19 mmol, 1 eq.) and *N*-Butylaniline (155 µL, 0.97 mmol, 5 eq.) followed by dry MeTHF (0.39 mL) reaction mixture was stirred at 80 °C for 16 h. After reaction was completed vial was taken from glovebox and concentrated

under reduced pressure, from the crude reaction mixture prepared NMR sample. Column chromatography (SiO_2, Pentane/Et_2O 7:1, followed by Pentane/Et_2O 4:1, v/v) of the crude mixture gave **7dc** as a light yellow oil (58 mg, 99%). The *ee* was determined to be 87 % using chiral HPLC (OD-H, *i*-propanol/*n*-hexane = 2/98, flow rate = 0.5 mL/min, l = 254 nm) t_R = 25.6 (major), 26.8 (minor).

Rf 0.34 (SiO_2, Pentane/Et_2O 4:1, v/v).

^{1}H NMR (400 MHz, $CDCl_3$) δ 7.56 – 7.50 (m, 1H), 7.29 – 7.21 (m, 4H), 7.13 – 7.08 (m, 1H), 6.94 (d, *J* = 8.0 Hz, 2H), 6.77 (t, *J* = 7.3 Hz, 1H), 6.57 (dd, *J* = 9.8, 2.5 Hz, 1H), 5.95 (dd, *J* = 9.7, 3.1 Hz, 1H), 5.12 (d, *J* = 9.6 Hz, 1H), 4.65 (dt, *J* = 9.6, 2.8 Hz, 1H), 3.32 – 3.16 (m, 2H), 2.67 (s, 1H), 1.71 – 1.39 (m, 2H), 1.27 (t, *J* = 3.7 Hz, 2H), 0.87 (t, *J* = 7.3 Hz, 3H).

^{13}C{^{1}H} NMR (101 MHz, $CDCl_3$) δ 148.7, 136.5, 132.1, 129.5, 129.4, 128.8, 128.2, 128.1, 126.6, 126.0, 117.9, 115.3, 70.4, 64.0, 47.5, 31.0, 20.4, 14.0.

$[\alpha]^{25}_D$ = -20 (c = 0.25, $CHCl_3$).

IR (ATR neat) ṽ =: 3402, 3059, 3035, 2956, 2926, 2856, 1719 cm^{-1}.

HRMS (ESI): *m*/*z* calculated for $C_{20}H_{23}NNaO^+$ [M + Na^+] 316.1672; found 316.1668.

***ee* determination:**

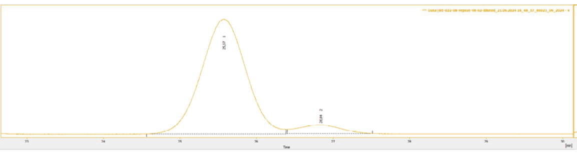

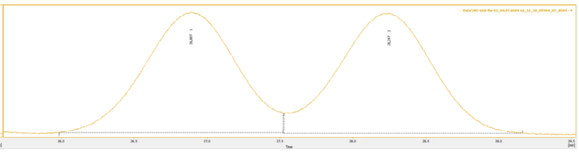

	Reten. Time [min]	Area [mAU.s]	Height [mAU]	Area [%]
1	25,573	6956,065	170,581	93,3
2	26,837	499,724	13,041	6,7
	Total	7455,788	183,622	100,0

	Reten. Time [min]	Area [mAU.s]	Height [mAU]	Area [%]
1	26,887	2112,594	48,191	49,5
2	28,247	2158,330	48,000	50,5
	Total	4270,924	96,192	100,0

(1*R*,2*R*)-2-((4-bromophenyl)(ethyl)amino)-1,2-dihydronaphthalen-1-ol (7e)

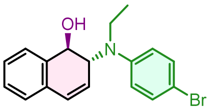

$C_{18}H_{18}BrNO$
344,25 g/mol

Following **G. P. E** vial containing **Rh5ba** (4 mg, 3 mol%) was charged to glovebox and into reaction vial added oxabenzonorbornadiene (28 mg, 0.19 mmol, 1 eq.) and 4-bromo-*N*-ethylaniline (121 µL, 0.97 mmol, 5 eq.) followed by dry MeTHF (0.39 mL) reaction mixture was stirred at 80 °C for 16 h. After reaction was completed vial was taken from glovebox and concentrated under reduced pressure, from the crude reaction mixture prepared NMR sample. Column chromatography (SiO_2, Pentane/Et_2O 4:1, v/v) of the crude mixture gave **7e** as a light yellow oil (51 mg, 76%). The *ee* was determined to be 83 % using chiral HPLC (AS-H, *i*-propanol/*n*-hexane = 10/90, flow rate = 0.5 mL/min, l = 254 nm) t_R = 15.5 (major), 18.0 (minor).

Rf 0.52 (SiO_2, Pentane/Et_2O 1:1, v/v).

^{1}H NMR (400 MHz, $CDCl_3$) δ 7.53 – 7.49 (m, 1H), 7.35 – 7.27 (m, 4H), 7.17 – 7.11 (m, 1H), 6.84 – 6.77 (m, 2H), 6.62 (dd, *J* = 9.7, 2.4 Hz, 1H), 5.92 (dd, *J* = 9.7, 3.3 Hz, 1H), 5.06 (d, *J* = 8.8 Hz, 1H), 4.61 (dt, *J* = 8.8, 2.9 Hz, 1H), 3.31 (q, *J* = 7.1 Hz, 2H), 2.29 (s, 1H), 1.11 (t, *J* = 7.0 Hz, 3H).

^{13}C{^{1}H} NMR (101 MHz, $CDCl_3$) δ 147.3, 136.3, 132.1, 132.0, 129.9, 128.4, 128.4, 128.1, 126.8, 126.3, 116.3, 109.5, 70.4, 63.2, 41.7, 14.3.

$[\alpha]^{25}_D$ = +16 (c = 0.25, $CHCl_3$).

IR (ATR neat) ṽ =: 3403, 2966, 2924, 2853, 1720, 1587, 1492, 1452 cm^{-1}.

HRMS (ESI): *m*/*z* calculated for $C_{18}H_{18}BrNNaO^+$ [M + Na^+] 366.0469; found 366.0469.

***ee* determination:**

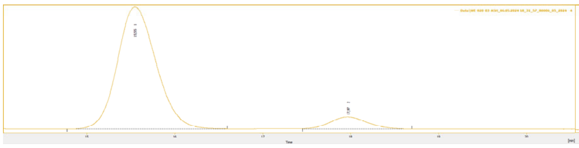

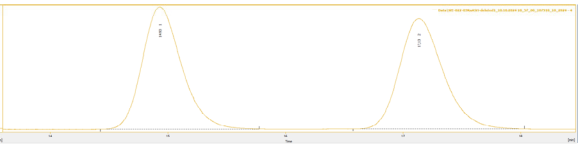

	Reten. Time [min]	Area [mAU.s]	Height [mAU]	Area [%]
1	15,545	17738,878	578,820	91,5
2	17,970	1657,636	56,183	8,5
	Total	19396,513	635,003	100,0

	Reten. Time [min]	Area [mAU.s]	Height [mAU]	Area [%]
1	14,934	15812,596	678,608	50,1
2	17,134	15746,977	613,236	49,9
	Total	31559,573	1291,844	100,0

(1*R*,2*R*)-2-((4-methoxyphenyl)(phenyl)amino)-1,2-dihydronaphthalen-1-ol (7f)

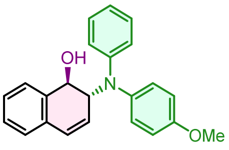

$C_{23}H_{21}NO_2$
343,43 g/mol

Following **G. P. E** vial containing **Rh5ba** (4 mg, 3 mol%) was charged to glovebox and into reaction vial added oxabenzonorbornadiene (28 mg, 0.19 mmol, 1 eq.) and 4-methoxy-*N*-phenylaniline (193 mg, 0.97 mmol, 5 eq.) followed by dry MeTHF (0.39 mL) reaction mixture was stirred at 80 °C for 16 h. After reaction was completed vial was taken from glovebox and concentrated under reduced pressure, from the crude reaction mixture prepared NMR sample. Column chromatography (SiO_2, Pentane/Et_2O 3:1, v/v) of the crude mixture gave **7f** as a light yellow oil (43 mg, 65%). The *ee* was determined to be 76 % using chiral HPLC (OD-H, *i*-propanol/*n*-hexane = 10/90, flow rate = 0.5 mL/min, l = 254 nm) t_R = 18.5 (minor), 19.7 (major).

Rf 0.18 (SiO_2, Pentane/Et_2O 3:1, v/v).

^{1}H NMR (400 MHz, $CDCl_3$) δ 7.57 – 7.52 (m, 1H), 7.31 – 7.19 (m, 4H), 7.15 – 7.09 (m, 2H), 7.07 – 7.03 (m, 1H), 6.91 – 6.83 (m, 5H), 6.50 (dd, *J* = 9.8, 2.5 Hz, 1H), 6.15 (dd, *J* = 9.8, 3.0 Hz, 1H), 5.19 (d, *J* = 10.0 Hz, 1H), 5.04 (dt, *J* = 10.1, 2.8 Hz, 1H), 3.81 (s, 3H), 2.46 (s, 1H).

^{13}C{^{1}H} NMR (101 MHz, $CDCl_3$) δ 157.4, 148.9, 137.8, 136.8, 132.2, 130.1, 129.2, 129.1, 128.9, 128.1, 128.0, 126.5, 125.8, 119.0, 117.4, 114.8, 69.8, 62.9, 55.5.

$[\alpha]^{25}_D$ = +210 (c = 0.10, $CHCl_3$).

IR (ATR neat) ṽ =: 3389, 3050, 2932, 2834, 1704, 1630, 1596, 1579 cm^{-1}.

HRMS (ESI): *m*/*z* calculated for $C_{23}H_{21}NNaO_2^+$ [M + Na^+] 366.1465; found 366.1463.

***ee* determination:**

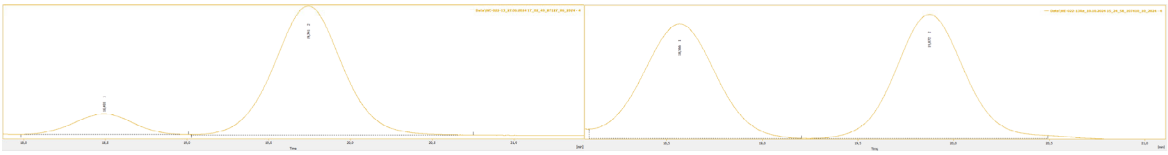

	Reten. Time [min]	Area [mAU.s]	Height [mAU]	Area [%]
1	18,493	1658,265	65,591	12,2
2	19,741	11979,847	408,481	87,8
	Total	13638,112	474,072	100,0

	Reten. Time [min]	Area [mAU.s]	Height [mAU]	Area [%]
1	18,566	4862,552	179,997	49,6
2	19,872	4940,683	194,941	50,4
	Total	9803,234	374,938	100,0

(1*S*,2*S*)-2-(1*H*-indol-3-yl)-1,2-dihydronaphthalen-1-ol (7ga)

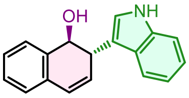

$C_{18}H_{15}NO$
261,32 g/mol

Following **G. P. E** vial containing **Rh5ba** (2 mg, 3 mol%) was charged to glovebox and into reaction vial added oxabenzonorbornadiene (14 mg, 0.095 mmol, 1 eq.) and indole (57 mg, 0.49 mmol, 5 eq.) followed by dry THF (0.19 mL) reaction mixture was stirred at 80 °C for 16 h. After reaction was completed vial was taken from glovebox and concentrated under reduced pressure, from the crude reaction mixture prepared NMR sample. Column chromatography (SiO_2, petroleum ether/EtOAc 9:1, v/v) of the crude mixture gave **7ga** as an brown oil (25 mg, 99%). The *ee* was determined to be 78 % using chiral HPLC (OD-H, *i*-propanol/*n*-hexane = 10/90, flow rate = 0.5 mL/min, l = 254 nm) t_R = 54.0 (major), 58.5 (minor).

Rf 0.36 (SiO_2, petroleum ether/EtOAc 7:3, v/v).

^{1}H NMR (400 MHz, $CDCl_3$) δ 7.92 (s, 1H), 7.67 (d, *J* = 8.0 Hz, 1H), 7.35 – 7.28 (m, 1H), 7.25 (dt, *J* = 8.2, 0.8 Hz, 1H), 7.20 (td, *J* = 7.4, 1.6 Hz, 1H), 7.17 – 7.12 (m, 2H), 7.12 – 7.07 (m, 1H), 7.05 (ddd, *J* = 8.0, 7.1, 1.1 Hz, 1H), 6.83 (d, *J* = 2.4 Hz, 1H), 6.57 (dd, *J* = 9.5, 2.1 Hz, 1H), 6.08 (dd, *J* = 9.6, 3.9 Hz, 1H), 4.93 (d, *J* = 8.0 Hz, 1H), 3.99 (ddd, *J* = 8.0, 3.7, 2.0 Hz, 1H), 1.96 (s, 1H).

^{13}C{^{1}H} NMR (101 MHz, $CDCl_3$) δ 136.8, 136.2, 132.7, 130.3, 128.2, 128.0, 127.2, 126.7, 126.7, 126.5, 122.7, 122.4, 119.7, 119.6, 114.5, 111.5, 73.0, 41.4.

$[\alpha]^{25}_D$ = -8 (c = 1.00, $CHCl_3$).

The spectral data were consistent with the literature.[28]

***ee* determination:**

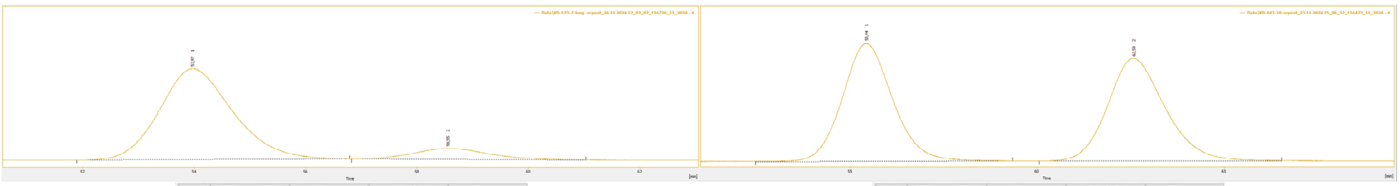

	Reten. Time [min]	Area [mAU.s]	Height [mAU]	Area [%]
1	53,966	27837,339	304,126	89,1
2	58,547	3412,756	36,603	10,9
	Total	31250,095	340,729	100,0

	Reten. Time [min]	Area [mAU.s]	Height [mAU]	Area [%]
1	55,441	31429,697	335,829	50,2
2	62,588	31118,818	291,951	49,8
	Total	62548,515	627,780	100,0

(1*S*,2*S*)-2-(1-methyl-1*H*-indol-3-yl)-1,2-dihydronaphthalen-1-ol (7gb)

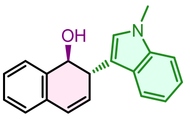

$C_{19}H_{17}NO$
275,35 g/mol

Following **G. P. E** vial containing **Rh5ba** (4 mg, 3 mol%) was charged to glovebox and into reaction vial added oxabenzonorbornadiene (28 mg, 0.19 mmol, 1 eq.) and 1-methylindole (120 µL, 0.97 mmol, 5 eq.) followed by dry THF (0.39 mL) reaction mixture was stirred at 80 °C for 16 h. After reaction was completed vial was taken from glovebox and concentrated under reduced pressure, from the crude reaction mixture prepared NMR sample. Column chromatography (SiO_2, petroleum ether/EtOAc 9:1, v/v) of the crude mixture gave **7gb** as a light yellow oil (43 mg, 79%). The *ee* was determined to be 62 % using chiral HPLC (AS-H, *i*-propanol/*n*-hexane = 10/90, flow rate = 0.5 mL/min, l = 254 nm) t_R = 24.0 (major), 27.4 (minor).

Rf 0.46 (SiO_2, petroleum ether/EtOAc 7:3, v/v).

^{1}H NMR (400 MHz, $CDCl_3$) δ 7.73 (d, *J* = 7.9 Hz, 1H), 7.43 – 7.36 (m, 1H), 7.31 – 7.20 (m, 4H), 7.16 (dd, *J* = 7.2, 1.4 Hz, 1H), 7.11 (ddd, *J* = 7.9, 6.9, 1.1 Hz, 1H), 6.80 (s, 1H), 6.63 (dd, *J* = 9.6, 2.0 Hz, 1H), 6.15 (dd, *J* = 9.6, 3.8 Hz, 1H), 4.99 (d, *J* = 8.2 Hz, 1H), 4.05 (ddd, *J* = 7.9, 3.7, 2.2 Hz, 1H), 3.67 (s, 3H), 2.08 (s, 1H).

^{13}C{^{1}H} NMR (101 MHz, $CDCl_3$) δ 137.6, 136.3, 132.7, 130.5, 128.1, 127.9, 127.3, 127.2, 127.1, 126.6, 126.4, 122.0, 119.7, 119.2, 113.0, 109.6, 73.1, 41.3, 32.8.

$[\alpha]^{25}_D$ = -77 (c = 1.00, $CHCl_3$).

IR (ATR neat) ṽ =: 3538, 3388, 3029, 2929, 1730, 1613, 1544, 1482 cm^{-1}.

HRMS (ESI): *m*/*z* calculated for $C_{19}H_{17}NO^+$ [M + Na^+] 298.1202; found 298.1204.

***ee* determination:**

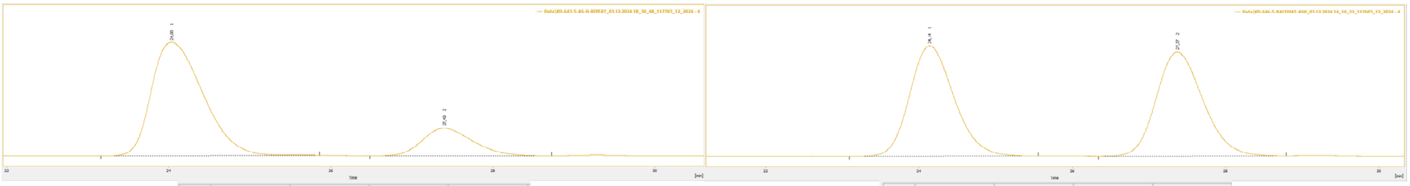

	Reten. Time [min]	Area [mAU.s]	Height [mAU]	Area [%]
1	24,031	55560,306	1269,791	81,0
2	27,398	13030,668	306,688	19,0
	Total	68590,974	1576,480	100,0

	Reten. Time [min]	Area [mAU.s]	Height [mAU]	Area [%]
1	24,135	29653,413	742,524	50,2
2	27,368	29426,363	699,420	49,8
	Total	59079,776	1441,944	100,0

(1*R*,2*R*)-6,7-dimethyl-2-(methyl(phenyl)amino)-1,2-dihydronaphthalen-1-ol (7ha)

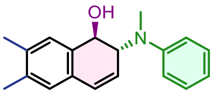

$C_{19}H_{21}NO$
279,38 g/mol

Following **G. P. E** vial containing **Rh5ba** (4 mg, 3 mol%) was charged to glovebox and into reaction vial added **6c** (33 mg, 0.19 mmol, 1 eq.) and *N*-Methylaniline (105 µL, 0.97 mmol, 5 eq.) followed by dry MeTHF (0.39 mL) reaction mixture was stirred at 80 °C for 16 h. After reaction was completed vial was taken from glovebox and concentrated under reduced pressure, from the crude reaction mixture prepared NMR sample. Column chromatography (SiO_2, Hex/Et_2O 9:1, v/v) of the crude mixture gave **7ha** as a light yellow oil (47 mg, 87%). The *ee* was determined to be 82 % using chiral HPLC (OD-H, *i*-propanol/*n*-hexane = 10/90, flow rate = 0.5 mL/min, l = 254 nm) t_R = 17.4 (major), 19.7 (minor).

Rf 0.27 (SiO_2, Hex/Et_2O 3:1, v/v).

^{1}H NMR (600 MHz, $CDCl_3$) δ 7.32 (s, 1H), 7.31 – 7.27 (m, 2H), 7.00 – 6.97 (m, 2H), 6.94 (s, 1H), 6.82 (tt, *J* = 7.2, 1.0 Hz, 1H), 6.58 (dd, *J* = 9.7, 2.4 Hz, 1H), 5.88 (dd, *J* = 9.7, 3.2 Hz, 1H), 5.04 (d, *J* = 8.9 Hz, 1H), 4.72 (dt, *J* = 9.0, 2.8 Hz, 1H), 2.83 (s, 3H), 2.30 (d, *J* = 12.2 Hz, 6H).

$^{13}C\{^1H\}$ NMR (151 MHz, $CDCl_3$) δ 150.3, 136.7, 136.2, 133.9, 129.7, 129.7, 129.4, 128.1, 127.4, 126.4, 118.0, 114.6, 70.1, 63.4, 33.3, 19.8, 19.5.

$[\alpha]^{25}_D$ = +60 (c = 0.10, $CHCl_3$).

The spectral data were consistent with the literature.[5]

***ee* determination:**

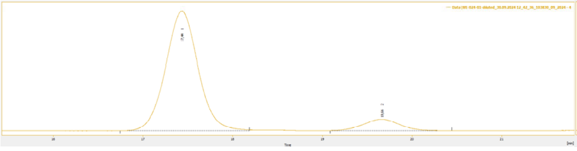

	Reten. Time [min]	Area [mAU.s]	Height [mAU]	Area [%]
1	17,436	13693,471	544,246	90,3
2	19,665	1474,338	51,697	9,7
	Total	15167,808	595,942	100,0

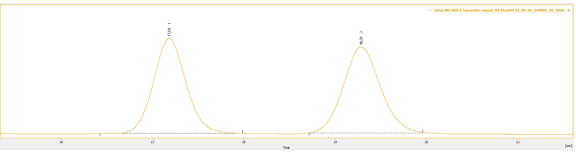

	Reten. Time [min]	Area [mAU.s]	Height [mAU]	Area [%]
1	17,177	29498,777	1188,177	49,2
2	19,280	30420,901	1077,089	50,8
	Total	59919,678	2265,266	100,0

(1*R*,2*R*)-2-((4-chlorophenyl)(methyl)amino)-6,7-dimethyl-1,2-dihydronaphthalen-1-ol (7hb)

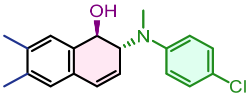

$C_{19}H_{20}ClNO$
313,83 g/mol

Following **G. P. E** vial containing **Rh5ba** (4 mg, 3 mol%) was charged to glovebox and into reaction vial added **6c** (33 mg, 0.19 mmol, 1 eq.) and 4-Chloro-*N*-methylaniline (117 µL, 0.97 mmol, 5 eq.) followed by dry MeTHF (0.39 mL) reaction mixture was stirred at 80 °C for 16 h. After reaction was completed vial was taken from glovebox and concentrated under reduced pressure, from the crude reaction mixture prepared NMR sample. Column chromatography (SiO_2, Hex/Et_2O 9:1, v/v) of the crude mixture gave **7hb** as a light yellow oil (53 mg, 87%). The *ee* was determined to be 72 % using chiral HPLC (AS-H, *i*-propanol/*n*-hexane = 5/95, flow rate = 0.5 mL/min, l = 254 nm) t_R = 21.9 (major), 25.8 (minor).

Rf 0.19 (SiO_2, Hex/Et_2O 3:1, v/v).

^1H NMR (600 MHz, $CDCl_3$) δ 7.28 (s, 1H), 7.22 – 7.17 (m, 2H), 6.93 (s, 1H), 6.88 – 6.84 (m, 2H), 6.58 (dd, *J* = 9.7, 2.3 Hz, 1H), 5.83 (dd, *J* = 9.7, 3.3 Hz, 1H), 4.98 (d, *J* = 8.5 Hz, 1H), 4.62 (dt, *J* = 8.7, 2.9 Hz, 1H), 2.77 (s, 3H), 2.28 (d, *J* = 9.3 Hz, 6H), 2.24 (s, 1H).

$^{13}C\{^1H\}$ NMR (151 MHz, $CDCl_3$) δ 148.9, 136.8, 136.4, 133.7, 129.9, 129.6, 129.1, 128.2, 127.5, 125.9, 122.7, 115.6, 70.1, 63.4, 33.5, 19.8, 19.5.

$[\alpha]^{25}_D$ = +30 (c = 0.10, $CHCl_3$).

IR (ATR neat) ṽ =: 3386, 2920, 2855, 1594, 1563, 1494, 1452 cm^{-1}.

HRMS (ESI): *m*/*z* calculated for $C_{19}H_{20}ClNNaO^+$ [M + Na^+] 336.1126; found 336.1131.

***ee* determination:** AS-H column, 95/5 Hex/*i*PrOH, 35 °C, 0.5 ml/min.

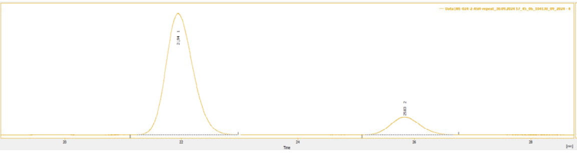

	Reten. Time [min]	Area [mAU.s]	Height [mAU]	Area [%]
1	21,943	34653,795	1056,157	86,1
2	25,834	5611,697	151,635	13,9
	Total	40265,492	1207,792	100,0

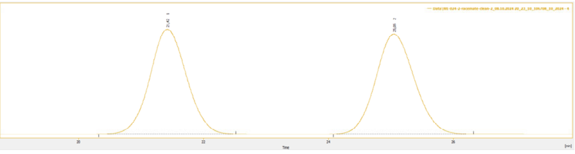

	Reten. Time [min]	Area [mAU.s]	Height [mAU]	Area [%]
1	21,424	60140,836	1465,414	49,9
2	25,050	60388,686	1397,386	50,1
	Total	120529,522	2862,800	100,0

(1*R*,2*R*)-2-((4-methoxyphenyl)(methyl)amino)-6,7-dimethyl-1,2-dihydronaphthalen-1-ol (7hc)

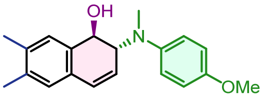

$C_{20}H_{23}NO_2$
309,41 g/mol

Following **G. P. E** vial containing **Rh5ba** (4 mg, 3 mol%) was charged to glovebox and into reaction vial added **6c** (33 mg, 0.19 mmol, 1 eq.) and 4-Methoxy-*N*-methylaniline (133 mg, 0.97 mmol, 5 eq.) followed by dry MeTHF (0.39 mL) reaction mixture was stirred at 80 °C for 16 h. After reaction was completed vial was taken from glovebox and concentrated under reduced pressure, from the crude reaction mixture prepared NMR sample. Column chromatography (SiO_2, Hex/Et_2O 9:1, v/v) of the crude mixture gave **7hc** as a light yellow oil (55 mg, 92%). The *ee* was determined to be 80 % using chiral HPLC (OD-H, *i*-propanol/*n*-hexane = 10/90, flow rate = 0.5 mL/min, l = 254 nm) t_R = 23.1 (minor), 26.2 (major).

Rf 0.14 (SiO_2, Hex/Et_2O 3:1, v/v).

^{1}H NMR (600 MHz, $CDCl_3$) δ 7.34 (s, 1H), 6.98 – 6.94 (m, 2H), 6.91 (s, 1H), 6.88 – 6.84 (m, 2H), 6.54 (dd, *J* = 9.8, 2.4 Hz, 1H), 5.89 (dd, *J* = 9.7, 3.0 Hz, 1H), 5.02 (d, *J* = 9.6 Hz, 1H), 4.51 (dt, *J* = 9.7, 2.8 Hz, 1H), 3.78 (s, 3H), 2.77 (s, 3H), 2.55 (s, 1H), 2.29 (d, *J* = 16.8 Hz, 6H).

^{13}C{^{1}H} NMR (151 MHz, $CDCl_3$) δ 152.9, 144.9, 136.5, 136.0, 134.1, 129.7, 129.5, 128.0, 127.0, 126.2, 117.4, 114.7, 69.7, 65.2, 55.8, 33.9, 19.8, 19.5.

$[\alpha]^{25}_D$ = +100 (c = 0.10, $CHCl_3$).

IR (ATR neat) ṽ =: 3507, 3064, 3032, 3007, 2922, 2854, 1738, 1575, 1509, 1399, 1376 cm^{-1}.

HRMS (ESI): *m*/*z* calculated for $C_{20}H_{23}NNaO_2^+$ [M + Na^+] 332.1621; found 332.1625.

***ee* determination:**

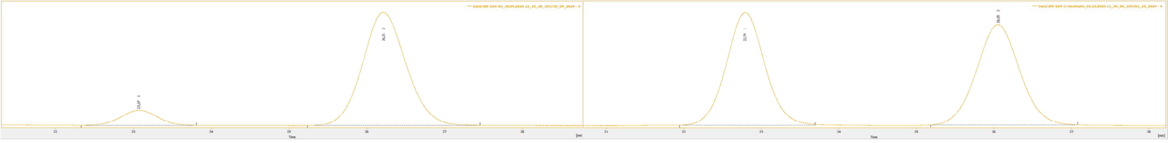

	Reten. Time [min]	Area [mAU.s]	Height [mAU]	Area [%]
1	23,065	2573,975	78,491	9,9
2	26,211	23413,458	600,586	90,1
	Total	25987,433	679,077	100,0

	Reten. Time [min]	Area [mAU.s]	Height [mAU]	Area [%]
1	22,785	14569,571	421,865	50,1
2	26,052	14498,812	374,929	49,9
	Total	29068,383	796,794	100,0

(1*R*,2*R*)-2-((4-chlorophenyl)(methyl)amino)-6,7-dimethoxy-1,2-dihydronaphthalen-1-ol (7ia)

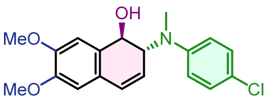

$C_{19}H_{20}ClNO_3$
345,82 g/mol

Following **G. P. E** vial containing **Rh5ba** (4 mg, 3 mol%) was charged to glovebox and into reaction vial added **6d** (40 mg, 0.19 mmol, 1 eq.) and 4-Chloro-*N*-methylaniline (117 µL, 0.97 mmol, 5 eq.) followed by dry MeTHF (0.39 mL) reaction mixture was stirred at 80 °C for 16 h. After reaction was completed vial was taken from glovebox and concentrated under reduced pressure, from the crude reaction mixture prepared NMR sample. Column chromatography (SiO_2, Hex/Et_2O 1:1, v/v) of the crude mixture gave **7ia** as a light yellow oil (31 mg, 47%). The *ee* was determined to be 64 % using chiral HPLC (AS-H, *i*-propanol/*n*-hexane = 10/90, flow rate = 0.5 mL/min, l = 254 nm) t_R = 39.2 (major), 42.5 (minor).

Rf 0.07 (SiO_2, Hex/Et_2O 1:1, v/v).

^{1}H NMR (400 MHz, $CDCl_3$) δ 7.22 – 7.17 (m, 2H), 7.09 (s, 1H), 6.89 – 6.85 (m, 2H), 6.68 (s, 1H), 6.53 (dd, *J* = 9.7, 2.4 Hz, 1H), 5.80 (dd, *J* = 9.7, 3.3 Hz, 1H), 4.99 (d, *J* = 9.1 Hz, 1H), 4.63 (dt, *J* = 9.1, 2.8 Hz, 1H), 3.91 (d, *J* = 9.7 Hz, 6H), 2.79 (s, 3H), 2.19 (s, 1H).

^{13}C{^{1}H} NMR (101 MHz, $CDCl_3$) δ 148.9, 148.9, 148.7, 129.7, 129.2, 129.1, 125.2, 124.9, 122.9, 115.7, 110.3, 109.7, 70.2, 63.7, 56.2, 33.6.

$[\alpha]^{25}_D$ = +30 (c = 0.10, $CHCl_3$).

IR (ATR neat) ṽ =: 3430, 2924, 2853, 1723, 1594, 1462, 1403, 1376, 1327 cm^{-1}.

HRMS (ESI): *m*/*z* calculated for $C_{19}H_{20}ClNNaO_3^+$ [M + Na^+] 368.1024; found 368.1028.

***ee* determination:**

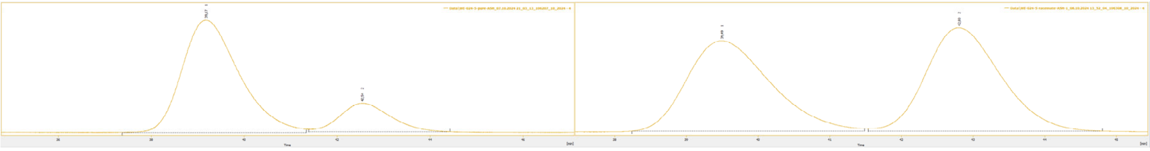

	Reten. Time [min]	Area [mAU.s]	Height [mAU]	Area [%]
1	39,174	9484,359	111,077	81,8
2	42,536	2108,598	27,851	18,2
	Total	11592,957	138,929	100,0

	Reten. Time [min]	Area [mAU.s]	Height [mAU]	Area [%]
1	39,494	20124,028	248,786	49,5
2	42,803	20555,376	284,970	50,5
	Total	40679,404	533,756	100,0

(1*R*,2*R*)-6,7-dimethoxy-2-((4-methoxyphenyl)(methyl)amino)-1,2-dihydronaphthalen-1-ol (7ib)

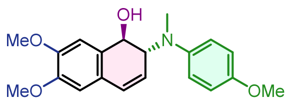

$C_{20}H_{23}NO_4$
341,41 g/mol

Following **G. P. E** vial containing **Rh5ba** (4 mg, 3 mol%) was charged to glovebox and into reaction vial added **6d** (40 mg, 0.19 mmol, 1 eq.) and 4-Methoxy-*N*-methylaniline (133 mg, 0.97 mmol, 5 eq.) followed by dry MeTHF (0.39 mL) reaction mixture was stirred at 80 °C for 16 h. After reaction was completed vial was taken from glovebox and concentrated under reduced pressure, from the crude reaction mixture prepared NMR sample. Column chromatography (SiO_2, Hex/Et_2O 1:1, v/v) of the crude mixture gave **7ib** as a light yellow oil (53 mg, 80%). The *ee* was determined to be 76 % using chiral HPLC (OD-H, *i*-propanol/*n*-hexane = 10/90, flow rate = 0.5 mL/min, l = 254 nm) t_R = 56.9 (minor), 60.2 (major).

Rf 0.08 (SiO_2, Hex/Et_2O 1:1, v/v).

^{1}H NMR (400 MHz, $CDCl_3$) δ 7.13 (s, 1H), 6.97 – 6.92 (m, 2H), 6.87 – 6.81 (m, 2H), 6.65 (s, 1H), 6.48 (dd, *J* = 9.8, 2.5 Hz, 1H), 5.85 (dd, *J* = 9.8, 2.9 Hz, 1H), 5.01 (d, *J* = 10.2 Hz, 1H), 4.49 (dt, *J* = 10.1, 2.7 Hz, 1H), 3.92 (s, 3H), 3.88 (s, 3H), 3.76 (s, 3H), 2.77 (d, *J* = 1.1 Hz, 3H), 2.60 (s, 1H).

^{13}C{^{1}H} NMR (101 MHz, $CDCl_3$) δ 153.1, 148.8, 148.3, 144.9, 129.6, 129.2, 125.4, 125.0, 117.5, 114.8, 110.2, 109.3, 69.8, 65.5, 56.1, 55.8, 34.1.

$[\alpha]^{25}_D$ = +30 (c = 0.10, $CHCl_3$).

IR (ATR neat) ṽ =: 3485, 2995, 2933, 2833, 1603, 1574, 1506, 1462 cm^{-1}.

HRMS (ESI): *m*/*z* calculated for $C_{20}H_{23}NNaO_4^+$ [M + Na^+] 364.1519; found 364.1522.

***ee* determination:**

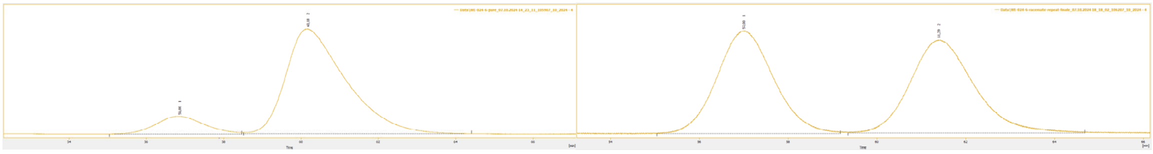

	Reten. Time [min]	Area [mAU.s]	Height [mAU]	Area [%]
1	56,858	8732,446	100,024	11,9
2	60,176	64638,944	598,944	88,1
	Total	73371,390	698,968	100,0

	Reten. Time [min]	Area [mAU.s]	Height [mAU]	Area [%]
1	57,001	4546,978	51,047	49,6
2	61,393	4622,237	46,273	50,4
	Total	9169,214	97,320	100,0

(1*R*,2*R*)-6,7-difluoro-2-(methyl(phenyl)amino)-1,2-dihydronaphthalen-1-ol (7ic)

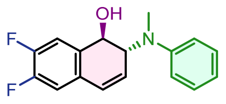

$C_{17}H_{15}F_2NO$
287,31 g/mol

Following **G. P. E** vial containing **Rh5ba** (4 mg, 3 mol%) was charged to glovebox and into reaction vial added **6f** (34 mg, 0.19 mmol, 1 eq.) and *N*-Methylaniline (105 µL, 0.97 mmol, 5 eq.) followed by dry MeTHF (0.39 mL) reaction mixture was stirred at 80 °C for 16 h. After reaction was completed vial was taken from glovebox and concentrated under reduced pressure, from the crude reaction mixture prepared NMR sample. Column chromatography (SiO_2, Hex/Et_2O 9:1, v/v) of the crude mixture gave **7ic** as a light yellow oil (54 mg, 99%). The *ee* was determined to be 78 % using chiral HPLC (OD-H, *i*-propanol/*n*-hexane = 10/90, flow rate = 0.5 mL/min, l = 254 nm) t_R = 22.6 (minor), 26.2 (major).

Rf 0.19 (SiO_2, Hex/Et_2O 4:1, v/v).

^{1}H NMR (400 MHz, $CDCl_3$) δ 7.41 (dd, *J* = 10.9, 7.9 Hz, 1H), 7.30 – 7.26 (m, 2H), 6.96 (d, *J* = 8.2 Hz, 2H), 6.91 (dd, *J* = 10.5, 7.7 Hz, 1H), 6.82 (t, *J* = 7.3 Hz, 1H), 6.45 (dd, *J* = 9.8, 2.7 Hz, 1H), 5.96 (dd, *J* = 9.8, 2.5 Hz, 1H), 5.08 (d, *J* = 11.1 Hz, 1H), 4.71 (dt, *J* = 11.3, 2.6 Hz, 1H), 2.90 (s, 3H), 2.46 (s, 1H).

^{13}C{^{1}H} NMR (^{13}C NMR (101 MHz, $CDCl_3$) δ 150.98 (d, *J* = 13.1 Hz), 150.29, 133.98 – 133.56 (m), 129.39, 129.09 (d, *J* = 2.9 Hz), 127.97 (t, *J* = 1.9 Hz), 69.49, 64.09, 33.70.

^{19}F NMR (377 MHz, $CDCl_3$) δ -138.56 (d, *J* = 20.6 Hz), -140.72 (d, *J* = 21.0 Hz).

$[\alpha]^{25}_D$ = +50 (c = 0.10, $CHCl_3$).

The spectral data were consistent with the literature.[29]

***ee* determination:**

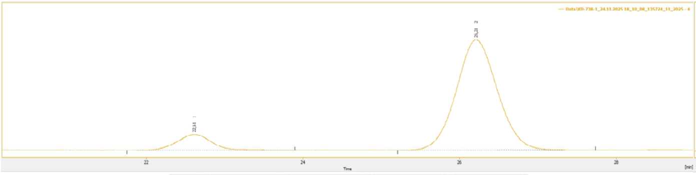

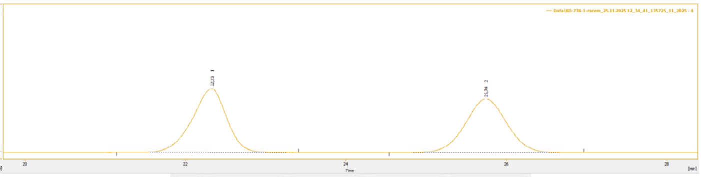

	Reten. Time [min]	Area [mAU.s]	Height [mAU]	Area [%]
1	22,606	2003,633	63,414	11,1
2	26,207	16127,812	440,011	88,9
	Total	18131,445	503,425	100,0

	Reten. Time [min]	Area [mAU.s]	Height [mAU]	Area [%]
1	22,329	17839,498	594,109	49,8
2	25,742	17962,193	499,051	50,2
	Total	35801,691	1093,160	100,0

(1*R*,2*R*)-2-((4-chlorophenyl)(methyl)amino)-6,7-difluoro-1,2-dihydronaphthalen-1-ol (7id)

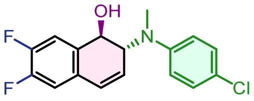

$C_{17}H_{14}ClF_2NO$
321,75 g/mol

Following **G. P. E** vial containing **Rh5ba** (4 mg, 3 mol%) was charged to glovebox and into reaction vial added **6f** (34 mg, 0.19 mmol, 1 eq.) and 4-Chloro-*N*-methylaniline (117 µL, 0.97 mmol, 5 eq.) followed by dry THF (0.39 mL) reaction mixture was stirred at 80 °C for 16 h. After reaction was completed vial was taken from glovebox and concentrated under reduced pressure, from the crude reaction mixture prepared NMR sample. Column chromatography (SiO_2, Hex/Et_2O 9:1, v/v) of the crude mixture gave **7id** as a light yellow oil (58 mg, 95%). The *ee* was determined to be 72 % using chiral HPLC (AS-H, *i*-propanol/*n*-hexane = 5/95, flow rate = 0.5 mL/min, l = 254 nm) t_R = 28.5 (minor), 31.3 (major).

Rf 0.11 (SiO_2, Hex/Et_2O 4:1, v/v).

^{1}H NMR (400 MHz, $CDCl_3$) δ 7.40 (dd, *J* = 10.8, 7.9 Hz, 1H), 7.24 – 7.15 (m, 2H), 6.92 (dd, *J* = 10.6, 7.6 Hz, 1H), 6.89 – 6.83 (m, 2H), 6.46 (dd, *J* = 9.8, 2.7 Hz, 1H), 5.92 (dd, *J* = 9.8, 2.6 Hz, 1H), 5.05 (d, *J* = 11.0 Hz, 1H), 4.64 (dt, *J* = 11.0, 2.6 Hz, 1H), 2.87 (s, 3H), 2.39 (s, 1H).

^{13}C{^{1}H} NMR (101 MHz, $CDCl_3$) δ 151.02 (dd, *J* = 12.9, 2.6 Hz), 148.84, 148.56 (dd, *J* = 8.6, 4.5 Hz), 133.56 (dd, *J* = 4.0, 1.6 Hz), 129.15, 128.79 (t, *J* = 3.9 Hz), 128.63 (d, *J* = 2.5 Hz), 128.20 (t, *J* = 2.2 Hz), 123.43, 69.50, 64.18, 33.83.

^{19}F NMR (377 MHz, $CDCl_3$) δ -138.28 (d, *J* = 20.6 Hz), -140.41 (d, *J* = 21.0 Hz).

$[\alpha]^{25}_D$ = +40 (c = 0.10, $CHCl_3$).

IR (ATR neat) ṽ =: 3419, 2923, 1592, 1494, 1305, 1208, 1167, 1100, 867, 809 cm^{-1}.

HRMS (ESI): *m*/*z* calculated for $C_{17}H_{14}ClF_2NNaO^+$ [M + Na^+] 344.0624; found 344.0627.

***ee* determination:**

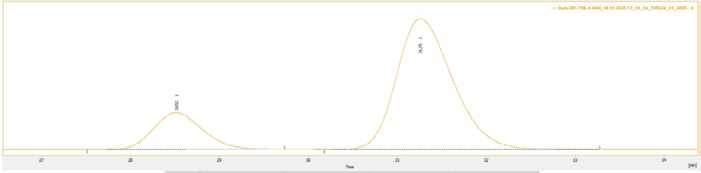

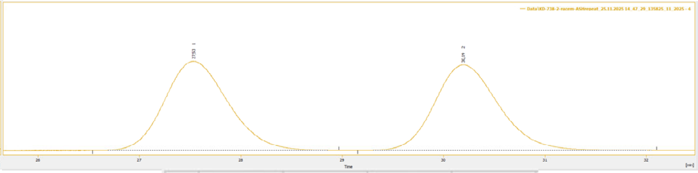

	Reten. Time [min]	Area [mAU.s]	Height [mAU]	Area [%]
1	28,516	5209,121	168,470	13,9
2	31,255	32349,125	735,911	86,1
	Total	37558,246	904,381	100,0

	Reten. Time [min]	Area [mAU.s]	Height [mAU]	Area [%]
1	27,532	31134,725	713,607	50,9
2	30,192	30083,936	685,460	49,1
	Total	61218,661	1399,067	100,0

(6*R*,7*R*)-7-(methyl(phenyl)amino)-2,3,6,7-tetrahydronaphtho[2,3-*b*][1,4]dioxin-6-ol (7ja)

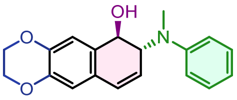

$C_{19}H_{19}NO_3$
309,37 g/mol

Following **G. P. E** vial containing **Rh5ba** (4 mg, 3 mol%) was charged to glovebox and into reaction vial added **6e** (39 mg, 0.19 mmol, 1 eq.) and *N*-Methylaniline (105 µL, 0.97 mmol, 5 eq.) followed by dry MeTHF (0.39 mL) reaction mixture was stirred at 80 °C for 16 h. After reaction was completed vial was taken from glovebox and concentrated under reduced pressure, from the crude reaction mixture prepared NMR sample. Column chromatography (SiO_2, Hex/Et_2O 3:1, v/v) of the crude mixture gave **7ja** as a light yellow oil (47 mg, 79%). The *ee* was determined to be 80 % using chiral HPLC (OD-H, *i*-propanol/*n*-hexane = 10/90, flow rate = 0.5 mL/min, l = 254 nm) t_R = 55.0 (minor), 64.8 (major).

Rf 0.06 (SiO_2, Hex/Et_2O 3:1, v/v).

^{1}H NMR (400 MHz, $CDCl_3$) δ 7.29 – 7.23 (m, 2H), 7.04 (s, 1H), 6.96 (d, *J* = 7.8 Hz, 2H), 6.79 (tt, *J* = 7.3, 1.0 Hz, 1H), 6.66 (s, 1H), 6.48 (dd, *J* = 9.7, 2.4 Hz, 1H), 5.81 (dd, *J* = 9.7, 3.2 Hz, 1H), 4.96 (d, *J* = 8.9 Hz, 1H), 4.66 (dt, *J* = 9.0, 2.8 Hz, 1H), 4.26 (s, 4H), 2.81 (s, 3H), 2.25 (s, 1H).

^{13}C{^{1}H} NMR (101 MHz, $CDCl_3$) δ 150.2, 143.4, 143.1, 130.3, 129.4, 129.2, 125.9, 125.8, 118.2, 115.7, 115.6, 114.7, 69.8, 64.6, 64.5, 63.3, 33.5.

$[\alpha]^{25}_D$ = +10 (c = 0.10, $CHCl_3$).

The spectral data were consistent with the literature.[30]

***ee* determination:**

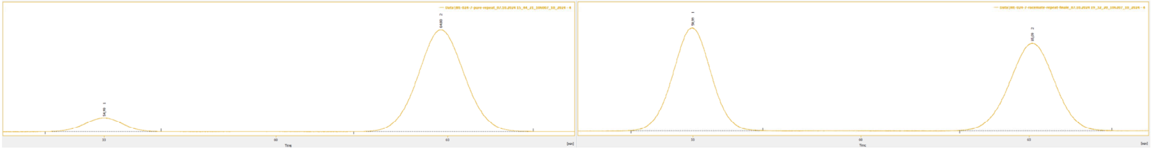

	Reten. Time [min]	Area [mAU.s]	Height [mAU]	Area [%]
1	54,993	3075,016	39,469	9,8
2	64,802	28178,924	296,892	90,2
	Total	31253,940	336,361	100,0

	Reten. Time [min]	Area [mAU.s]	Height [mAU]	Area [%]
1	54,992	13418,589	168,898	49,9
2	65,093	13486,124	143,499	50,1
	Total	26904,713	312,397	100,0

(6*R*,7*R*)-7-((4-chlorophenyl)(methyl)amino)-2,3,6,7-tetrahydronaphtho[2,3-*b*][1,4]dioxin-6-ol (7jb)

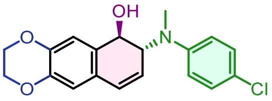

$C_{19}H_{18}ClNO_3$
343,81 g/mol

Following **G. P. E** vial containing **Rh5ba** (4 mg, 3 mol%) was charged to glovebox and into reaction vial added **6e** (39 mg, 0.19 mmol, 1 eq.) and 4-Chloro-*N*-methylaniline (117 µL, 0.97 mmol, 5 eq.) followed by dry MeTHF (0.39 mL) reaction mixture was stirred at 80 °C for 16 h. After reaction was completed vial was taken from glovebox and concentrated under reduced pressure, from the crude reaction mixture prepared NMR sample. Column chromatography (SiO_2, Hex/Et_2O 6:1, v/v) of the crude mixture gave **7jb** as a light yellow oil (39 mg, 59%). The *ee* was determined to be 71 % using chiral HPLC (AS-H, *i*-propanol/*n*-hexane = 10/90, flow rate = 1.0 mL/min, l = 254 nm) t_R = 30.3 (minor), 41.5 (major).

Rf 0.06 (SiO_2, Hex/Et_2O 3:1, v/v).

^{1}H NMR (400 MHz, $CDCl_3$) δ 7.21 – 7.16 (m, 2H), 7.02 (s, 1H), 6.88 – 6.82 (m, 2H), 6.66 (s, 1H), 6.50 (dd, *J* = 9.7, 2.3 Hz, 1H), 5.78 (dd, *J* = 9.7, 3.3 Hz, 1H), 4.91 (d, *J* = 8.6 Hz, 1H), 4.59 (dt, *J* = 8.6, 2.8 Hz, 1H), 4.26 (s, 4H), 2.77 (s, 3H), 2.17 (s, 1H).

^{13}C{^{1}H} NMR (101 MHz, $CDCl_3$) δ 148.8, 143.4, 143.2, 130.1, 129.4, 129.1, 125.8, 125.3, 122.8, 115.8, 115.7, 112.6, 69.8, 64.6, 64.5, 63.2, 33.6.

$[\alpha]^{25}_D$ = +20 (c = 0.10, $CHCl_3$).

IR (ATR neat) ṽ =: 3431, 3037, 2927, 2873, 1737, 1594, 1574, 1494, 1460 cm^{-1}.

HRMS (ESI): *m*/*z* calculated for $C_{19}H_{18}ClNNaO_3^+$ [M + Na^+] 366.0867; found 366.0867.

***ee* determination:**

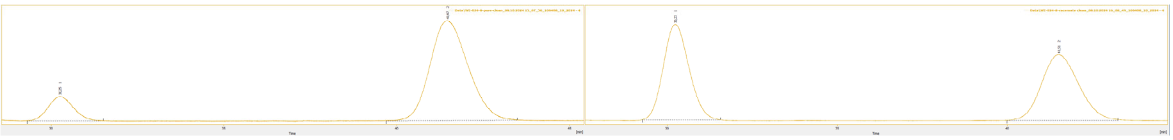

	Reten. Time [min]	Area [mAU.s]	Height [mAU]	Area [%]
1	30,253	495,732	9,563	14,3
2	41,468	2961,139	38,621	85,7
	Total	3456,871	48,184	100,0

	Reten. Time [min]	Area [mAU.s]	Height [mAU]	Area [%]
1	30,233	10804,606	205,690	50,0
2	41,513	10808,224	142,361	50,0
	Total	21612,830	348,051	100,0

(6*R*,7*R*)-7-((4-methoxyphenyl)(methyl)amino)-2,3,6,7-tetrahydronaphtho[2,3-*b*][1,4]dioxin-6-ol (7jc)

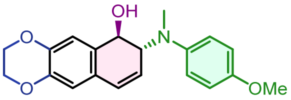

$C_{20}H_{21}NO_4$
339,39 g/mol

Following **G. P. E** vial containing **Rh5ba** (4 mg, 3 mol%) was charged to glovebox and into reaction vial added **6e** (39 mg, 0.19 mmol, 1 eq.) and 4-Methoxy-*N*-methylaniline (133 mg, 0.97 mmol, 5 eq.) followed by dry MeTHF (0.39 mL) reaction mixture was stirred at 80 °C for 16 h. After reaction was completed vial was taken from glovebox and concentrated under reduced pressure, from the crude reaction mixture prepared NMR sample. Column chromatography (SiO_2, Hex/Et_2O 3:1, followed by Hex/Et_2O 1:1, v/v)

of the crude mixture gave **7jc** as a light yellow oil (45 mg, 68%). The *ee* was determinated to be 70 % using chiral HPLC, retention times were 45.2 min. (major) and 50.2 min. The *ee* was determined to be 70 % using chiral HPLC (AS-H, *i*-propanol/*n*-hexane = 10/90, flow rate = 1.0 mL/min, l = 254 nm) t_R = 45.2 (major), 50.2 (minor).

Rf 0.14 (SiO_2, Hex/Et_2O 1:1, v/v).

^{1}H NMR (400 MHz, $CDCl_3$) δ 7.06 (s, 1H), 6.96 – 6.91 (m, 2H), 6.87 – 6.81 (m, 2H), 6.64 (s, 1H), 6.45 (dd, *J* = 9.8, 2.5 Hz, 1H), 5.83 (dd, *J* = 9.8, 3.0 Hz, 1H), 4.94 (d, *J* = 10.3 Hz, 1H), 4.46 (dt, *J* = 9.6, 2.8 Hz, 1H), 4.25 (s, 4H), 3.77 (s, 3H), 2.75 (s, 3H), 2.43 (s, 1H).

^{13}C{^{1}H} NMR (101 MHz, $CDCl_3$) δ 153.0, 144.9, 143.3, 142.9, 130.5, 129.0, 126.0, 125.7, 117.4, 115.6, 115.2, 114.8, 69.5, 65.1, 64.6, 64.5, 55.8, 34.0.

$[\alpha]^{25}_D$ = +30 (c = 0.10, $CHCl_3$).

IR (ATR neat) ṽ =: 3465, 3030, 2832, 1738, 1573, 1460, 1401 cm^{-1}.

HRMS (ESI): *m*/*z* calculated for $C_{20}H_{21}NNaO_4^+$ [M + Na^+] 362.1363; found 362.1369.

***ee* determination:**

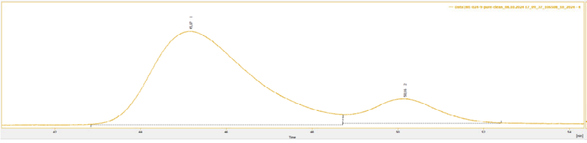

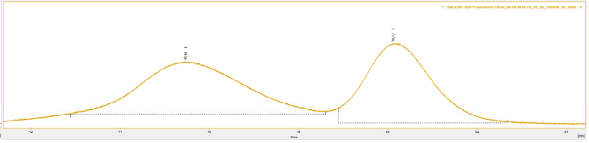

	Reten. Time [min]	Area [mAU.s]	Height [mAU]	Area [%]
1	45,170	3452,151	21,266	84,9
2	50,160	615,318	5,593	15,1
	Total	4067,469	26,859	100,0

	Reten. Time [min]	Area [mAU.s]	Height [mAU]	Area [%]
1	45,460	4084,398	25,510	50,5
2	50,117	4002,686	38,829	49,5
	Total	8087,084	64,339	100,0

(1*R*,2*R*)-2-(bis(4-bromophenyl)amino)-1,2-dihydronaphthalen-1-ol (7ka)

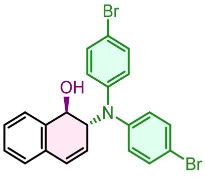

$C_{22}H_{17}Br_2NO$
471,19 g/mol

Following **G. P. E** vial containing **Rh5ba** (4 mg, 3 mol%) was charged to glovebox and into reaction vial added oxabenzonorbornadiene (28 mg, 0.19 mmol, 1 eq.) and Bis(4-bromophenyl)amine (316 mg, 0.97 mmol, 5 eq.) followed by dry MeTHF (0.39 mL) reaction mixture was stirred at 80 °C for 16 h. After reaction was completed vial was taken from glovebox and concentrated under reduced pressure, from the crude reaction mixture prepared NMR sample. Column chromatography (SiO_2, Pentane/Et_2O 19:1, v/v) of the crude mixture gave **7ka** as a light yellow oil (27 mg, 30%). The *ee* was determined to be 73 % using chiral HPLC (OD-H, *i*-propanol/*n*-hexane = 10/90, flow rate = 0.5 mL/min, l = 254 nm) t_R = 20.9 (major), 23.5 (minor).

Rf 0.19 (SiO_2, Pentane/Et_2O 9:1, v/v).

^{1}H NMR (400 MHz, $CDCl_3$) δ 7.53 – 7.46 (m, 1H), 7.38 – 7.29 (m, 4H), 7.30 – 7.22 (m, 2H), 7.08 – 7.01 (m, 1H), 6.93 – 6.84 (m, 4H), 6.49 (dd, *J* = 9.7, 2.4 Hz, 1H), 6.04 (dd, *J* = 9.7, 3.3 Hz, 1H), 5.18 (d, *J* = 9.3 Hz, 1H), 4.94 (dt, *J* = 9.3, 2.9 Hz, 1H), 2.06 (s, 1H).

^{13}C{^{1}H} NMR (101 MHz, $CDCl_3$) δ 145.6, 136.4, 132.4, 132.0, 129.5, 128.9, 128.4, 127.9, 126.8, 126.2, 124.9, 115.5, 69.5, 63.4.

$[\alpha]^{25}_D$ = +10 (c = 0.10, $CHCl_3$).

IR (ATR neat) ṽ =: 3397, 3053, 2955, 2924, 2854, 1708, 1631, 1583, 1484 cm^{-1}.

HRMS (ESI): *m*/*z* calculated for $C_{22}H_{17}Br_2NNaO^+$ [M + Na^+] 491.9569; found 491.9567.

***ee* determination:**

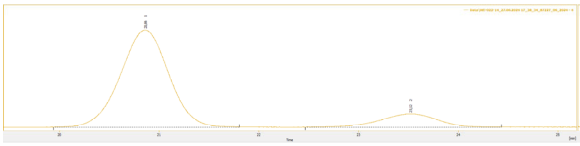

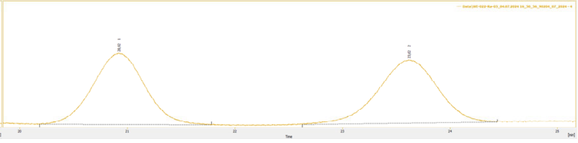

	Reten. Time [min]	Area [mAU.s]	Height [mAU]	Area [%]
1	20,861	5796,756	172,269	86,3
2	23,520	919,283	23,159	13,7
	Total	6716,038	195,428	100,0

	Reten. Time [min]	Area [mAU.s]	Height [mAU]	Area [%]
1	20,920	1674,118	49,664	49,7
2	23,619	1694,752	44,020	50,3
	Total	3368,870	93,684	100,0

(1*R*,2*R*)-2-(bis(4-methoxyphenyl)amino)-1,2-dihydronaphthalen-1-ol (7kb)

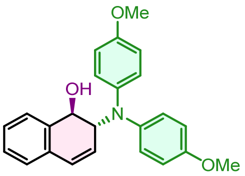

$C_{24}H_{23}NO_3$
373,45 g/mol

Following **G. P. E** vial containing **Rh5ba** (4 mg, 3 mol%) was charged to glovebox and into reaction vial added oxabenzonorbornadiene (28 mg, 0.19 mmol, 1 eq.) and Bis(4-methoxybenzyl)amine (222 mg, 0.97 mmol, 5 eq.) followed by dry MeTHF (0.39 mL) reaction mixture was stirred at 80 °C for 16 h. After reaction was completed vial was taken from glovebox and concentrated under reduced pressure, from the crude reaction mixture prepared NMR sample. Column chromatography (SiO_2, Pentane/Et_2O 3:1, v/v) of the crude mixture gave **7kb** as a light yellow oil (42 mg, 58%). The *ee* was determined to be 67 % using chiral HPLC (OD-H, *i*-propanol/*n*-hexane = 10/90, flow rate = 0.5 mL/min, l = 254 nm) t_R = 28.0 (minor), 31.4 (major).

Rf 0.22 (SiO_2, Pentane/Et_2O 3:1, v/v).

^{1}H NMR (400 MHz, $CDCl_3$) δ 7.58 (d, *J* = 7.6 Hz, 1H), 7.31 – 7.22 (m, 2H), 7.08 – 7.04 (m, 1H), 7.02 – 6.96 (m, 4H), 6.87 – 6.81 (m, 4H), 6.49 (dd, *J* = 9.8, 2.6 Hz, 1H), 6.18 (dd, *J* = 9.8, 2.8 Hz, 1H), 5.18 (d, *J* = 10.7 Hz, 1H), 4.96 (dt, *J* = 10.7, 2.7 Hz, 1H), 3.80 (s, 6H), 2.62 (s, 1H).

^{13}C{^{1}H} NMR (101 MHz, $CDCl_3$) δ 155.1, 140.9, 137.0, 132.1, 129.2, 128.9, 128.0, 127.8, 126.4, 125.5, 124.6, 114.7, 69.8, 63.9, 55.7.

$[\alpha]^{25}_D$ = +40 (c = 0.10, $CHCl_3$).

IR (ATR neat) ṽ =: 3380, 3049, 3011, 2956, 2928, 2838, 1714, 1595, 1579, 1504 cm^{-1}.

HRMS (ESI): *m*/*z* calculated for $C_{24}H_{23}NNaO_3^+$ [M + Na^+] 396.1570; found 396.1570.

***ee* determination:**

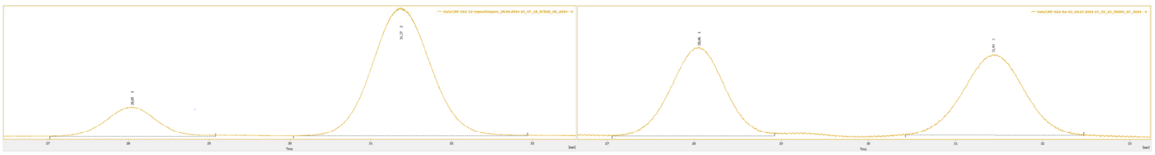

	Reten. Time [min]	Area [mAU.s]	Height [mAU]	Area [%]
1	28,045	9207,449	209,865	16,5
2	31,374	46677,057	921,744	83,5
	Total	55884,506	1131,609	100,0

	Reten. Time [min]	Area [mAU.s]	Height [mAU]	Area [%]
1	28,055	943,016	21,960	49,2
2	31,431	974,346	19,988	50,8
	Total	1917,362	41,948	100,0

tert-butyl ((1*R*,2*R*)-2-(methyl(phenyl)amino)-1,2-dihydronaphthalen-1-yl)carbamate (7la)

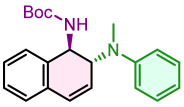

$C_{22}H_{26}N_2O_2$
350,46 g/mol

Following **G. P. E** vial containing **Rh5ba** (4 mg, 3 mol%) was charged to glovebox and into reaction vial added **6a** (45 mg, 0.19 mmol, 1 eq.) and *N*-methylaniline (105 µL, 0.97 mmol, 5 eq.) followed by dry THF (0.39 mL) reaction mixture was stirred at 80 °C for 16 h. After reaction was completed vial was taken from glovebox and concentrated under reduced pressure, from the crude reaction mixture prepared NMR sample. Column chromatography (SiO_2, petroleum ether/Et_2O 9:1, v/v) of the crude mixture gave **7la** as a light yellow oil (50 mg, 75%). The *ee* was determined to be 43 % using chiral HPLC (AS-H, *i*-propanol/*n*-hexane = 10/90, flow rate = 0.5 mL/min, l = 254 nm) t_R = 10.9 (major), 12.1 (minor).

Rf 0.38 (SiO_2, petroleum ether/Et_2O 9:1, v/v).

^{1}H NMR (600 MHz, $CDCl_3$) δ 7.35 (d, *J* = 7.4 Hz, 1H), 7.29 – 7.18 (m, 4H), 7.11 (d, *J* = 6.8 Hz, 1H), 6.87 (d, *J* = 8.3 Hz, 2H), 6.74 (t, *J* = 7.2 Hz, 1H), 6.62 (d, *J* = 9.7 Hz, 1H), 5.94 (dd, *J* = 9.8, 2.5 Hz, 1H), 5.22 (t, *J* = 10.0 Hz, 1H), 4.80 (d, *J* = 10.4 Hz, 1H), 4.52 (d, *J* = 9.1 Hz, 1H), 2.85 (s, 3H), 1.36 (s, 9H).

^{13}C{^{1}H} NMR (151 MHz, $CDCl_3$) δ 155.5, 135.9, 132.9, 130.2, 129.9, 129.5, 129.3, 128.1, 128.0, 126.8, 125.9, 117.3, 113.8, 79.6, 60.9, 52.5, 33.2, 28.5, 28.4.

$[\alpha]^{25}_D$ = +16 (c = 1.00, $CHCl_3$).

IR (ATR neat) ṽ =: 3378, 3062, 3022, 2974, 2927, 2901, 2817, 1685, 1594, 1520, 1501 cm^{-1}.

HRMS (ESI): *m*/*z* calculated for $C_{22}H_{26}N_2NaO_2^+$ [M + Na^+] 373.1886; found 373.1892.

***ee* determination:**

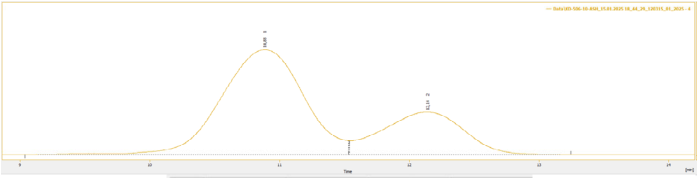

	Reten. Time [min]	Area [mAU.s]	Height [mAU]	Area [%]
1	10,883	28430,687	647,124	71,3
2	12,141	11457,237	261,055	28,7
	Total	39887,923	908,180	100,0

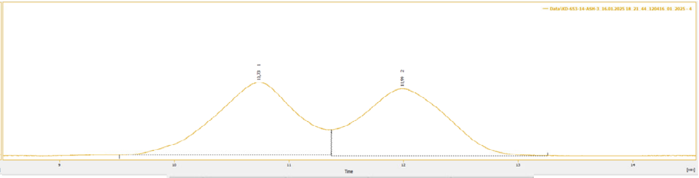

	Reten. Time [min]	Area [mAU.s]	Height [mAU]	Area [%]
1	10,733	3734,674	74,276	50,5
2	11,987	3659,965	69,914	49,5
	Total	7394,639	144,190	100,0

4-methyl-*N*-((1*R*,2*R*)-2-(methyl(phenyl)amino)-1,2-dihydronaphthalen-1-yl)benzenesulfonamide (7lb)

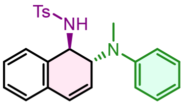

$C_{24}H_{24}N_2O_2S$
404,53 g/mol

Following **G. P. E** vial containing **Rh5ba** (4 mg, 3 mol%) was charged to glovebox and into reaction vial added **6b** (36 mg, 0.12 mmol, 1 eq.) and *N*-methylaniline (66 µL, 0.61 mmol, 5 eq.) followed by dry THF (0.24 mL) reaction mixture was stirred at 80 °C for 16 h. After reaction was completed vial was taken from glovebox and concentrated under reduced pressure, from the crude reaction mixture prepared NMR sample. Column chromatography (SiO_2, petroleum ether/EtOAc 9:1, v/v) of the crude mixture gave **7lb** as a light yellow oil (28 mg, 57%). The *ee* was determined to be 57 % using chiral HPLC (OD-H, *i*-propanol/*n*-hexane = 10/90, flow rate = 0.5 mL/min, l = 254 nm) t_R = 27.2 (major), 29.7 (minor).

Rf 0.25 (SiO_2, petroleum ether/EtOAc 6:1, v/v).

^{1}H NMR (400 MHz, $CDCl_3$) δ 7.66 – 7.60 (m, 2H), 7.29 – 7.19 (m, 5H), 7.15 – 7.09 (m, 2H), 6.92 (d, *J* = 7.5 Hz, 1H), 6.80 – 6.74 (m, 3H), 6.71 (dd, *J* = 9.7, 1.4 Hz, 1H), 5.87 (dd, *J* = 9.7, 4.7 Hz, 1H), 4.71 (td, *J* = 4.9, 1.4 Hz, 1H), 4.64 – 4.53 (m, 2H), 2.43 (s, 3H), 2.35 (s, 3H).

^{13}C{^{1}H} NMR (101 MHz, $CDCl_3$) δ 149.1, 143.5, 137.7, 133.9, 132.4, 130.5, 129.7, 129.3, 128.9, 128.6, 127.9, 127.4, 127.2, 126.2, 117.8, 114.0, 59.1, 54.7, 32.5, 21.7.

$[\alpha]^{25}_D$ = -81 (c = 1.00, $CHCl_3$).

IR (ATR neat) ṽ =: 3267, 3058, 3033, 2920, 2813, 1595, 1502, 1450, 1423 cm^{-1}.

HRMS (ESI): *m*/*z* calculated for $C_{24}H_{24}N_2NaO_2S^+$ [M + Na^+] 427.1451; found 427.1458.

***ee* determination:**

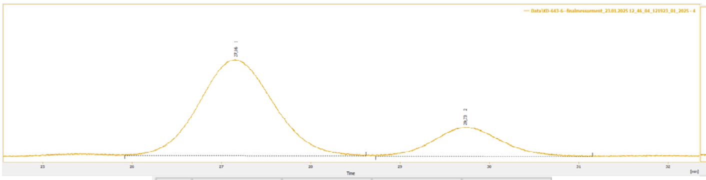

	Reten. Time [min]	Area [mAU.s]	Height [mAU]	Area [%]
1	27,157	4456,985	77,696	78,3
2	29,727	1232,961	23,390	21,7
	Total	5689,946	101,086	100,0

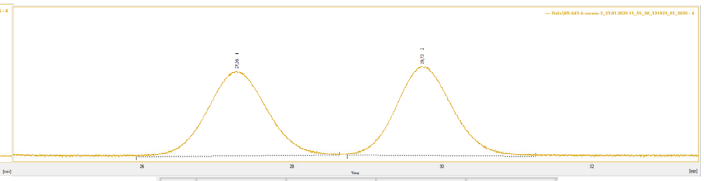

	Reten. Time [min]	Area [mAU.s]	Height [mAU]	Area [%]
1	27,258	1680,269	30,319	50,2
2	29,733	1668,260	31,859	49,8
	Total	3348,530	62,178	100,0

(1*R*,2*R*)-2-(*o*-tolyloxy)-1,2-dihydronaphthalen-1-ol (7ma)

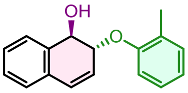

$C_{17}H_{16}O_2$
252,31 g/mol

Following **G. P. E** vial containing **Rh5ba** (4 mg, 3 mol%) was charged to glovebox and into reaction vial added oxabenzonorbornadiene (28 mg, 0.19 mmol, 1 eq.) and 2-methlphenole (105 mg, 0.97 mmol, 5 eq.) followed by dry ACN (0.39 mL) reaction mixture was stirred at 80 °C for 24 h. After reaction was completed vial was taken from glovebox and concentrated under reduced pressure, from the crude reaction mixture prepared NMR sample. Column chromatography (SiO_2, petroleum ether/Et_2O 93:7, v/v) of the crude mixture gave **7ma** as a white solid (18 mg, 37%). The *ee* was determined to be 83 % using chiral HPLC (AD-H, *i*-propanol/*n*-hexane = 15/85, flow rate = 0.5 mL/min, l = 254 nm) t_R = 14.4 (minor), 15.4 (major).

Rf 0.48 (SiO_2, petroleum ether/Et_2O 8:2, v/v).

^{1}H NMR (400 MHz, $CDCl_3$) δ 7.66 (d, *J* = 7.0 Hz, 1H), 7.35 – 7.27 (m, 2H), 7.20 (d, *J* = 7.3 Hz, 1H), 7.18 – 7.12 (m, 2H), 6.92 (t, *J* = 7.4 Hz, 1H), 6.88 (d, *J* = 8.2 Hz, 1H), 6.52 (dd, *J* = 9.9, 1.9 Hz, 1H), 6.03 (dd, *J* = 9.9, 2.1 Hz, 1H), 5.24 (d, *J* = 10.2 Hz, 1H), 5.13 (dt, *J* = 10.2, 2.0 Hz, 1H), 2.61 (s, 1H), 2.28 (s, 3H).

$^{13}C\{^{1}H\}$ NMR (101 MHz, $CDCl_3$) δ 155.6, 135.7, 132.1, 131.3, 129.0, 128.3, 128.1, 128.0, 127.0, 126.6, 126.6, 125.3, 121.4, 113.1, 79.5, 72.7, 16.6.

$[\alpha]^{25}_{D}$ = -155 (c = 1.00, $CHCl_3$).

The spectral data were consistent with the literature.[31]

***ee* determination:**

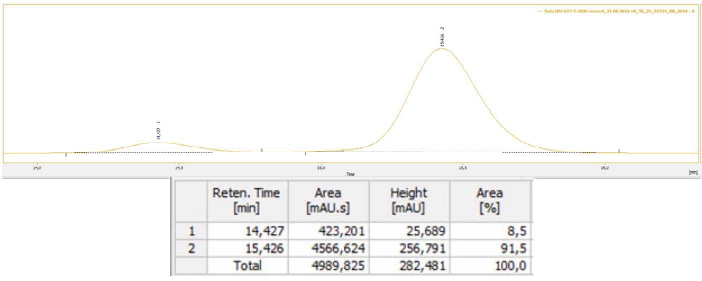

	Reten. Time [min]	Area [mAU.s]	Height [mAU]	Area [%]
1	14,427	423,201	25,689	8,5
2	15,426	4566,624	256,791	91,5
	Total	4989,825	282,481	100,0

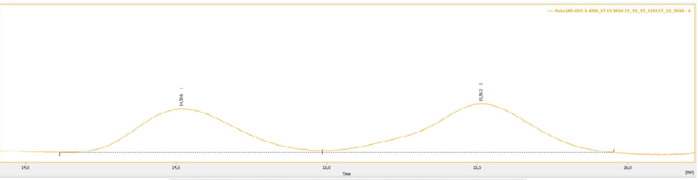

	Reten. Time [min]	Area [mAU.s]	Height [mAU]	Area [%]
1	14,516	1506,164	69,189	50,2
2	15,512	1492,083	70,253	49,8
	Total	2998,248	139,442	100,0

(1*R*,2*R*)-2-(*m*-tolyloxy)-1,2-dihydronaphthalen-1-ol (7mb)

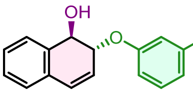

$C_{17}H_{16}O_2$
252,31 g/mol

Following **G. P. E** vial containing **Rh5ba** (4 mg, 3 mol%) was charged to glovebox and into reaction vial added oxabenzonorbornadiene (28 mg, 0.19 mmol, 1 eq.) and 3-methlphenole (101 µL, 0.97 mmol, 5 eq.) followed by dry ACN (0.39 mL) reaction mixture was stirred at 80 °C for 24 h. After reaction was completed vial was taken from glovebox and concentrated under reduced pressure, from the crude reaction mixture prepared NMR sample. Column chromatography (SiO_2, Hex/Et_2O 95:5, v/v) of the crude mixture gave **7mb** as a white solid (30 mg, 61%). The *ee* was determined to be 82 % using chiral HPLC (AD-H, *i*-propanol/*n*-hexane = 10/90, flow rate = 0.5 mL/min, l = 254 nm) t_R = 15.7 (minor), 22.4 (major).

Rf 0.20 (SiO_2, Hex/Et_2O 9:1, v/v).

^{1}H NMR (400 MHz, $CDCl_3$) δ 7.65 (d, *J* = 6.3 Hz, 1H), 7.35 – 7.24 (m, 2H), 7.19 (t, *J* = 7.7 Hz, 1H), 7.15 (s, 1H), 6.89 – 6.71 (m, 3H), 6.52 (dd, *J* = 9.8, 1.8 Hz, 1H), 6.04 (dd, *J* = 9.9, 2.1 Hz, 1H), 5.19 (d, *J* = 10.2 Hz, 1H), 5.12 (dt, *J* = 10.2, 2.0 Hz, 1H), 2.50 (s, 1H), 2.34 (s, 3H).

$^{13}C\{^{1}H\}$ NMR (101 MHz, $CDCl_3$) δ 157.5, 139.9, 135.7, 132.1, 129.6, 129.0, 128.3, 128.1, 126.5, 126.4, 125.3, 122.4, 116.8, 112.8, 79.1, 72.5, 21.6.

$[\alpha]^{25}_{D}$ = -140 (c = 1.00, $CHCl_3$).

The spectral data were consistent with the literature.[31]

***ee* determination:**

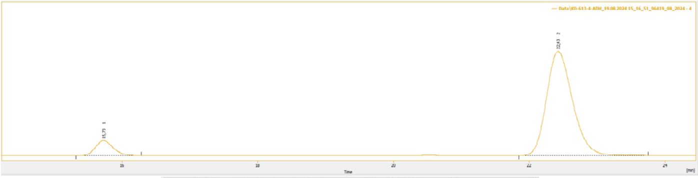

	Reten. Time [min]	Area [mAU.s]	Height [mAU]	Area [%]
1	15,728	1293,462	71,791	8,9
2	22,425	13274,548	500,247	91,1
	Total	14568,011	572,038	100,0

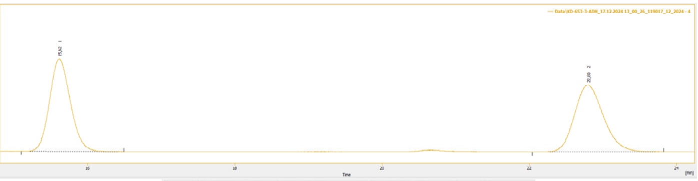

	Reten. Time [min]	Area [mAU.s]	Height [mAU]	Area [%]
1	15,618	6432,689	327,571	49,9
2	22,802	6450,206	236,742	50,1
	Total	12882,895	564,314	100,0

(1*R*,2*R*)-2-(*p*-tolyloxy)-1,2-dihydronaphthalen-1-ol (7mc)

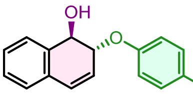

$C_{17}H_{16}O_2$
252,31 g/mol

Following **G. P. E** vial containing **Rh5ba** (4 mg, 3 mol%) was charged to glovebox and into reaction vial added oxabenzonorbornadiene (28 mg, 0.19 mmol, 1 eq.) and 4-methlphenole (105 mg, 0.97 mmol, 5 eq.) followed by dry ACN (0.39 mL) reaction mixture was stirred at 80 °C for 24 h. After reaction was completed vial was taken from glovebox and concentrated under reduced pressure, from the crude reaction mixture prepared NMR sample. Column chromatography (SiO_2, Hex/Et_2O 95:5, followed by Hexane/Et_2O 85:15 v/v) of the crude mixture gave **7mc** as a white solid (28 mg, 57%). The *ee* was determined to be 84 % using chiral HPLC (AD-H, *i*-propanol/*n*-hexane = 10/90, flow rate = 0.5 mL/min, l = 254 nm) t_R = 20.2 (minor), 28.0 (major).

Rf 0.17 (SiO_2, Hex/Et_2O 9:1, v/v).

^{1}H NMR (400 MHz, $CDCl_3$) δ 7.69 – 7.62 (m, 1H), 7.35 – 7.24 (m, 2H), 7.19 – 7.07 (m, 3H), 6.92 – 6.82 (m, 2H), 6.51 (dd, *J* = 9.8, 2.0 Hz, 1H), 6.04 (dd, *J* = 9.9, 2.2 Hz, 1H), 5.19 (d, *J* = 10.1 Hz, 1H), 5.08 (dt, *J* = 10.1, 2.1 Hz, 1H), 2.72 (s, 1H), 2.32 (s, 3H).

^{13}C{^{1}H} NMR (101 MHz, $CDCl_3$) δ 155.3, 135.7, 132.1, 131.0, 130.3, 129.0, 128.3, 128.1, 126.6, 126.5, 125.3, 116.0, 79.4, 72.5, 20.6.

[α]$^{25}_D$ = -116 (c = 1.00, $CHCl_3$).

The spectral data were consistent with the literature.[32]

***ee* determination:**

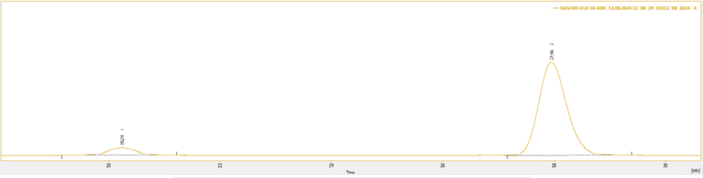

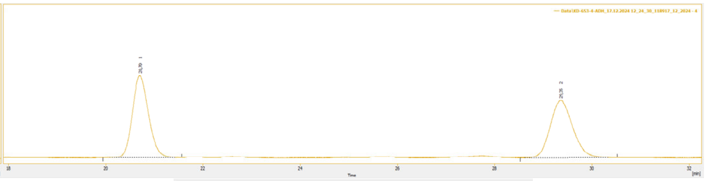

	Reten. Time [min]	Area [mAU.s]	Height [mAU]	Area [%]
1	20,236	725,265	20,056	8,1
2	27,956	8218,990	235,056	91,9
	Total	8944,254	255,112	100,0

	Reten. Time [min]	Area [mAU.s]	Height [mAU]	Area [%]
1	20,702	5471,316	238,434	50,6
2	29,354	5346,126	167,024	49,4
	Total	10817,442	405,458	100,0

(1*R*,2*R*)-2-(4-chlorophenoxy)-1,2-dihydronaphthalen-1-ol (7na)

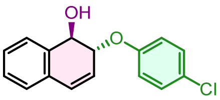

$C_{16}H_{13}ClO_2$
272,73 g/mol

Following **G. P. E** vial containing **Rh5ba** (4 mg, 3 mol%) was charged to glovebox and into reaction vial added oxabenzonorbornadiene (28 mg, 0.19 mmol, 1 eq.) and 4-chlorophenole (125 mg, 0.97 mmol, 5 eq.) followed by dry THF (0.39 mL) reaction mixture was stirred at 80 °C for 16 h. After reaction was completed vial was taken from glovebox and concentrated under reduced pressure, from the crude reaction mixture prepared NMR sample. Column chromatography (SiO_2, petroleum ether/Et_2O 9:1, v/v) of the crude mixture gave **7na** as a white solid (51 mg, 98%). The *ee* was determined to be 81 % using chiral HPLC (AD-H, *i*-propanol/*n*-hexane = 10/90, flow rate = 1.0 mL/min, l = 254 nm) t_R = 13.1 (minor), 16.5 (major).

Rf 0.44 (SiO_2, petroleum ether/EtOAc 9:1, v/v).

^{1}H NMR (400 MHz, $CDCl_3$) δ 7.67 – 7.58 (m, 1H), 7.34 – 7.20 (m, 4H), 7.16 – 7.05 (m, 1H), 6.92 – 6.84 (m, 2H), 6.52 (dd, *J* = 9.8, 1.9 Hz, 1H), 5.97 (dd, *J* = 9.9, 2.2 Hz, 1H), 5.16 (d, *J* = 10.0 Hz, 1H), 5.05 (dt, *J* = 10.0, 2.1 Hz, 1H), 2.62 (s, 1H).

^{13}C{^{1}H} NMR (101 MHz, $CDCl_3$) δ 156.1, 135.5, 131.9, 129.7, 129.5, 128.5, 128.3, 126.7, 126.5, 125.6, 125.4, 117.3, 79.6, 72.4.

[α]$^{25}_D$ = -69 (c = 1.00, $CHCl_3$).

The spectral data were consistent with the literature.[32]

***ee* determination:**

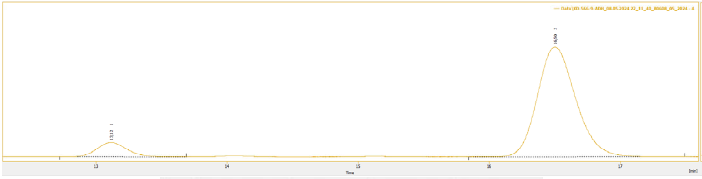

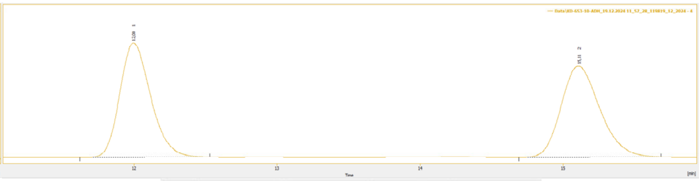

	Reten. Time [min]	Area [mAU.s]	Height [mAU]	Area [%]
1	13,115	856,233	54,404	9,3
2	16,501	8384,117	414,126	90,7
	Total	9240,350	468,530	100,0

	Reten. Time [min]	Area [mAU.s]	Height [mAU]	Area [%]
1	11,996	16551,792	1097,612	50,0
2	15,109	16539,416	876,224	50,0
	Total	33091,208	1973,837	100,0

(1*R*,2*R*)-2-(4-(methylthio)phenoxy)-1,2-dihydronaphthalen-1-ol (7nb)

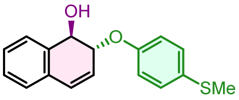

$C_{17}H_{16}O_2S$
284,37 g/mol

Following **G. P. E** vial containing **Rh5ba** (4 mg, 3 mol%) was charged to glovebox and into reaction vial added oxabenzonorbornadiene (28 mg, 0.19 mmol, 1 eq.) and 4-hydroxythioanisole (135 mg, 0.97 mmol, 5 eq.) followed by dry THF (0.39 mL) reaction mixture was stirred at 80 °C for 16 h. After reaction was completed vial was taken from glovebox and concentrated under reduced pressure, from the crude reaction mixture prepared NMR sample. Column chromatography (SiO_2, petroleum ether/Et_2O 9:1, v/v) of the crude mixture gave **7nb** as a white solid (49 mg, 89%). The *ee* was determined to be 78 % using chiral HPLC (AD-H, *i*-propanol/*n*-hexane = 10/90, flow rate = 0.5 mL/min, l = 254 nm) t_R = 30.8 (minor), 42.7 (major).

Rf 0.25 (SiO_2, petroleum ether/EtOAc 9:1, v/v).

^{1}H NMR (400 MHz, $CDCl_3$) δ 7.63 (d, *J* = 6.3 Hz, 1H), 7.35 – 7.23 (m, 4H), 7.15 – 7.07 (m, 1H), 6.95 – 6.86 (m, 2H), 6.52 (dd, *J* = 9.8, 1.8 Hz, 1H), 5.99 (dd, *J* = 9.8, 2.2 Hz, 1H), 5.17 (dd, *J* = 10.0, 3.2 Hz, 1H), 5.07 (dt, *J* = 10.1, 2.0 Hz, 1H), 2.60 (d, *J* = 3.8 Hz, 1H), 2.45 (s, 3H).

^{13}C{^{1}H} NMR (101 MHz, $CDCl_3$) δ 156.0, 135.6, 132.0, 130.1, 129.3, 128.4, 128.2, 126.7, 126.0, 125.4, 116.7, 79.5, 72.5, 17.9.

$[\alpha]^{25}_D$ = -97 (c = 1.00, $CHCl_3$).

IR (ATR neat) ṽ =: 3301, 3036, 2962, 2918, 2583, 1594, 11570, 1490, 1258 cm^{-1}.

HRMS (ESI): *m*/*z* calculated for $C_{17}H_{16}NaO_2S^+$ [M + Na^+] 307.0763; found 307.0765.

***ee* determination:**

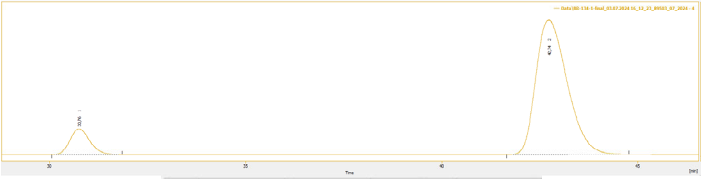

	Reten. Time [min]	Area [mAU.s]	Height [mAU]	Area [%]
1	30,757	12143,402	344,794	10,8
2	42,736	99917,446	1808,715	89,2
	Total	112060,848	2153,509	100,0

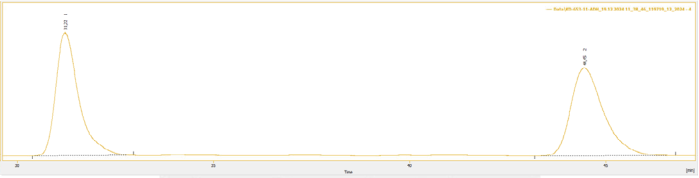

	Reten. Time [min]	Area [mAU.s]	Height [mAU]	Area [%]
1	31,222	62202,771	1541,952	49,7
2	44,454	62899,848	1100,140	50,3
	Total	125102,619	2642,092	100,0

4-(((1*R*,2*R*)-1-hydroxy-1,2-dihydronaphthalen-2-yl)oxy)benzonitrile (7nc)

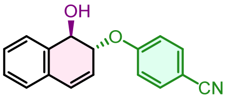

$C_{17}H_{13}NO_2$
263,30 g/mol

Following **G. P. E** vial containing **Rh5ba** (4 mg, 3 mol%) was charged to glovebox and into reaction vial added oxabenzonorbornadiene (28 mg, 0.19 mmol, 1 eq.) and 4-cyanophenol (115 mg, 0.97 mmol, 5 eq.) followed by dry THF (0.39 mL) reaction mixture was stirred at 80 °C for 48 h. After reaction was completed vial was taken from glovebox and concentrated under reduced pressure, from the crude reaction mixture prepared NMR sample. Column chromatography (SiO_2, petroleum ether/Et_2O 9:1, v/v) of the crude mixture gave **7nc** as a white solid (33 mg, 65%). The *ee* was determined to be 89 % using chiral HPLC (OD-H, *i*-propanol/*n*-hexane = 10/90, flow rate = 0.5 mL/min, l = 254 nm) t_R = 41.0 (major), 44.0 (minor).

Rf 0.50 (SiO_2, petroleum ether/EtOAc 9:1, v/v).

^{1}H NMR (400 MHz, $CDCl_3$) δ 7.68 – 7.56 (m, 3H), 7.36 – 7.28 (m, 2H), 7.20 – 7.10 (m, 1H), 7.06 – 6.95 (m, 2H), 6.58 (d, *J* = 9.5 Hz, 1H), 5.95 (dd, *J* = 9.9, 1.7 Hz, 1H), 5.23 – 5.11 (m, 2H), 2.59 (s, 1H).

^{13}C{^{1}H} NMR (101 MHz, $CDCl_3$) δ 161.0, 135.2, 134.3, 131.7, 130.2, 128.7, 128.5, 126.9, 125.6, 124.6, 119.1, 116.4, 104.7, 79.4, 72.2.

$[\alpha]^{25}_D$ = -89 (c = 1.00, $CHCl_3$).

The spectral data were consistent with the literature.[32]

***ee* determination:**

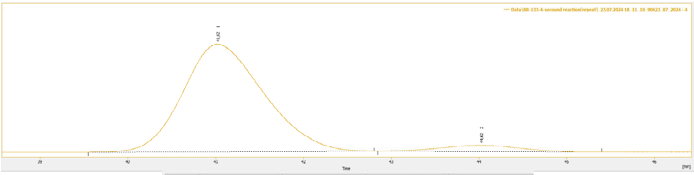 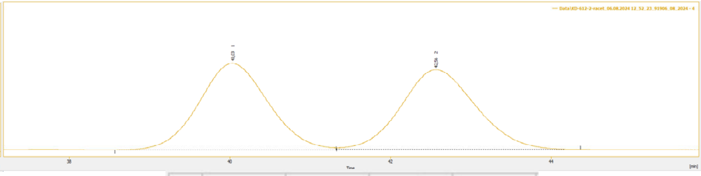

	Reten. Time [min]	Area [mAU.s]	Height [mAU]	Area [%]
1	41,024	78689,107	1229,021	94,5
2	44,024	4564,938	72,883	5,5
	Total	83254,045	1301,903	100,0

	Reten. Time [min]	Area [mAU.s]	Height [mAU]	Area [%]
1	40,034	32483,245	540,362	50,2
2	42,563	32239,030	498,727	49,8
	Total	64722,275	1039,089	100,0

(1*R*,2*R*)-2-(4-ethylphenoxy)-1,2-dihydronaphthalen-1-ol (7nd)

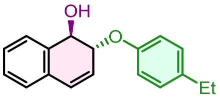

$C_{18}H_{18}O_2$
266,34 g/mol

Following **G. P. E** vial containing **Rh5ba** (4 mg, 3 mol%) was charged to glovebox and into reaction vial added oxabenzonorbornadiene (28 mg, 0.19 mmol, 1 eq.) and 4-ethylphenol (119 mg, 0.97 mmol, 5 eq.) followed by dry ACN (0.39 mL) reaction mixture was stirred at 80 °C for 16 h. After reaction was completed vial was taken from glovebox and concentrated under reduced pressure, from the crude reaction mixture prepared NMR sample. Column chromatography (SiO_2, Hex/Et_2O 9:1, v/v) of the crude mixture gave **7nd** as an oil (28 mg, 55%). The *ee* was determined to be 82 % using chiral HPLC (AD-H, *i*-propanol/*n*-hexane = 10/90, flow rate = 0.5 mL/min, l = 254 nm) t_R = 19.5 (minor), 28.1 (major).

Rf 0.19 (SiO_2, Hex/Et_2O 9:1, v/v).

^{1}H NMR (400 MHz, $CDCl_3$) δ 7.69 – 7.62 (m, 1H), 7.35 – 7.26 (m, 2H), 7.18 – 7.11 (m, 3H), 6.94 – 6.87 (m, 2H), 6.52 (dd, *J* = 9.9, 1.9 Hz, 1H), 6.05 (dd, *J* = 9.8, 2.2 Hz, 1H), 5.20 (d, *J* = 10.2 Hz, 1H), 5.10 (dt, *J* = 10.2, 2.1 Hz, 1H), 2.72 (s, 1H), 2.62 (q, *J* = 7.6 Hz, 2H), 1.24 (t, *J* = 7.6 Hz, 3H).

^{13}C{^{1}H} NMR (101 MHz, $CDCl_3$) δ 155.5, 137.5, 135.7, 132.1, 129.1, 129.0, 128.3, 128.1, 126.6, 126.5, 125.3, 116.0, 115.2, 79.4, 72.6, 28.1, 15.9.

$[\alpha]^{25}_D$ = -124 (c = 1.00, $CHCl_3$).

IR (ATR neat) ṽ =: 3294, 3037, 2961, 2928, 2870, 1719, 1606, 1506, 1454 cm^{-1}.

HRMS (ESI): *m*/*z* calculated for $C_{18}H_{18}NaO_2^+$ [M + Na$^+$] 289.1199; found 289.1197.

***ee* determination:**

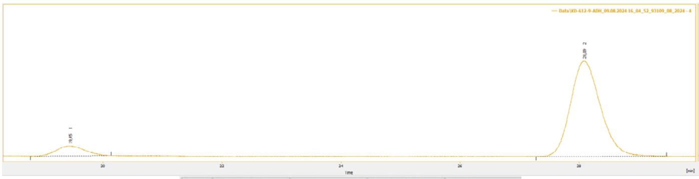 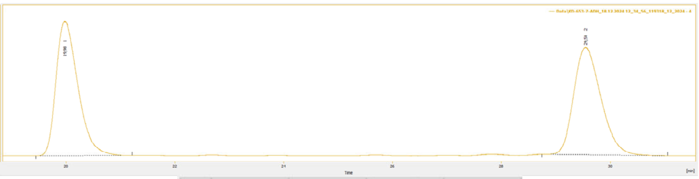

	Reten. Time [min]	Area [mAU.s]	Height [mAU]	Area [%]
1	19,453	265,554	8,043	9,1
2	28,091	2641,238	75,900	90,9
	Total	2906,792	83,943	100,0

	Reten. Time [min]	Area [mAU.s]	Height [mAU]	Area [%]
1	19,977	36966,074	1331,290	50,2
2	29,544	36640,816	1062,789	49,8
	Total	73606,889	2394,079	100,0

(1*R*,2*R*)-2-(4-propylphenoxy)-1,2-dihydronaphthalen-1-ol (7ne)

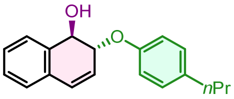

$C_{19}H_{20}O_2$
280,37 g/mol

Following **G. P. E** vial containing **Rh5ba** (4 mg, 3 mol%) was charged to glovebox and into reaction vial added oxabenzonorbornadiene (28 mg, 0.19 mmol, 1 eq.) and 4-propylphenol (132 mg, 0.97 mmol, 5 eq.) followed by dry ACN (0.39 mL) reaction mixture was stirred at 80 °C for 48 h. After reaction was completed vial was taken from glovebox and concentrated under reduced pressure, from the crude reaction mixture prepared NMR sample. Column chromatography (SiO_2, Hex/Et_2O 9:1, v/v) of the crude mixture gave **7ne** as a white solid (43 mg, 79%). The *ee* was determined to be 86 % using chiral HPLC (AD-H, *i*-propanol/*n*-hexane = 10/90, flow rate = 0.5 mL/min, l = 254 nm) t_R = 19.3 (minor), 27.0 (major).

Rf 0.24 (SiO_2, Hex/Et_2O 9:1, v/v).

^{1}H NMR (400 MHz, $CDCl_3$) δ 7.66 (d, *J* = 6.3 Hz, 1H), 7.35 – 7.26 (m, 2H), 7.16 – 7.09 (m, 3H), 6.94 – 6.86 (m, 2H), 6.52 (dd, *J* = 9.9, 1.9 Hz, 1H), 6.05 (dd, *J* = 9.9, 2.1 Hz, 1H), 5.20 (d, *J* = 10.2 Hz, 1H), 5.10 (dt, *J* = 10.2, 2.0 Hz, 1H), 2.72 (s, 1H), 2.56 (t, *J* = 7.5 Hz, 2H), 1.64 (h, *J* = 7.7 Hz, 2H), 0.96 (t, *J* = 7.3 Hz, 3H).

^{13}C{^{1}H} NMR (101 MHz, $CDCl_3$) δ 155.5, 135.9, 135.7, 132.1, 129.7, 128.9, 128.3, 128.1, 126.5, 126.5, 125.3, 115.9, 79.4, 72.6, 37.3, 24.9, 13.9.

$[\alpha]^{25}_D$ = -14 (c = 1.00, $CHCl_3$).

IR (ATR neat) ṽ =: 3347, 3053, 2957, 2927, 2860, 1596, 1578 cm^{-1}.

HRMS (ESI): *m/z* calculated for $C_{19}H_{20}NaO_2^+$ [M + Na$^+$] 303.1356; found 303.1355.

***ee* determination:**

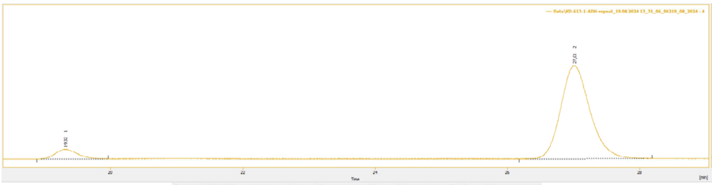

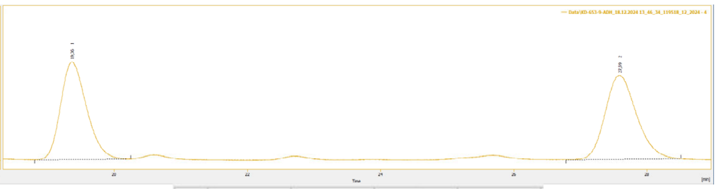

	Reten. Time [min]	Area [mAU.s]	Height [mAU]	Area [%]
1	19,317	151,412	6,474	7,2
2	27,008	1939,663	63,322	92,8
	Total	2091,076	69,796	100,0

	Reten. Time [min]	Area [mAU.s]	Height [mAU]	Area [%]
1	19,365	3185,518	117,698	49,0
2	27,590	3317,236	101,531	51,0
	Total	6502,754	219,229	100,0

(1*R*,2*R*)-2-(4-isopropylphenoxy)-1,2-dihydronaphthalen-1-ol (7nf)

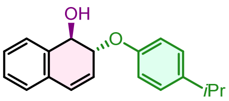

$C_{19}H_{20}O_2$
280,37 g/mol

Following **G. P. E** vial containing **Rh5ba** (4 mg, 3 mol%) was charged to glovebox and into reaction vial added oxabenzonorbornadiene (28 mg, 0.19 mmol, 1 eq.) and 4-isopropylphenol (132 mg, 0.97 mmol, 5 eq.) followed by dry ACN (0.39 mL) reaction mixture was stirred at 80 °C for 48 h. After reaction was completed vial was taken from glovebox and concentrated under reduced pressure, from the crude reaction mixture prepared NMR sample. Column chromatography (SiO_2, petroleum ether/Et_2O 85:15, v/v) of the crude mixture gave **7nf** as a white solid (37 mg, 65%). The *ee* was determined to be 89 % using chiral HPLC (AD-H, *i*-propanol/*n*-hexane = 10/90, flow rate = 0.5 mL/min, l = 254 nm) t_R = 18.5 (minor), 24.1 (major).

Rf 0.24 (SiO_2, Hex/Et_2O 9:1, v/v).

^{1}H NMR (400 MHz, $CDCl_3$) δ 7.71 – 7.62 (m, 1H), 7.35 – 7.27 (m, 2H), 7.21 – 7.16 (m, 2H), 7.17 – 7.10 (m, 1H), 6.96 – 6.88 (m, 2H), 6.52 (dd, *J* = 9.9, 1.9 Hz, 1H), 6.06 (dd, *J* = 9.9, 2.1 Hz, 1H), 5.20 (d, *J* = 10.2 Hz, 1H), 5.11 (dt, *J* = 10.3, 2.1 Hz, 1H), 2.90 (hept, *J* = 6.9 Hz, 1H), 2.74 (d, *J* = 2.7 Hz, 1H), 1.27 (d, *J* = 7.0 Hz, 6H).

^{13}C{^{1}H} NMR (101 MHz, $CDCl_3$) δ 155.5, 142.1, 135.7, 132.1, 128.9, 128.3, 128.1, 127.6, 126.5, 125.3, 115.8, 79.3, 72.5, 33.4, 24.3.

$[\alpha]^{25}_D$ = -23 (c = 0.50, $CHCl_3$).

IR (ATR neat) ṽ =: 3335, 3053, 2958, 2925, 2868, 1596, 1578, 1511, 1459 cm^{-1}.

HRMS (ESI): *m/z* calculated for $C_{19}H_{20}NaO_2^+$ [M + Na$^+$] 303.1356; found 303.1352.

***ee* determination:**

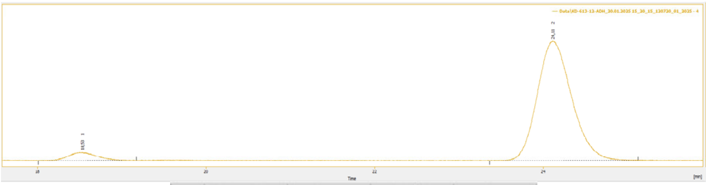

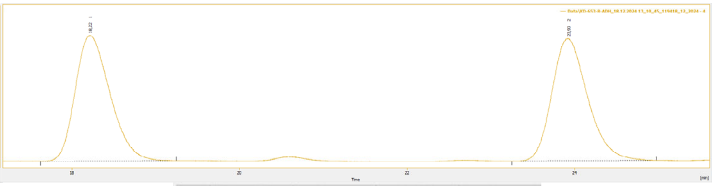

	Reten. Time [min]	Area [mAU.s]	Height [mAU]	Area [%]
1	18,529	181,377	7,311	5,7
2	24,113	2996,769	105,209	94,3
	Total	3178,147	112,520	100,0

	Reten. Time [min]	Area [mAU.s]	Height [mAU]	Area [%]
1	18,215	12193,458	422,827	50,0
2	23,928	12193,561	411,526	50,0
	Total	24387,019	834,354	100,0

(1*R*,2*R*)-2-(4-(*tert*-butyl)phenoxy)-1,2-dihydronaphthalen-1-ol (7ng)

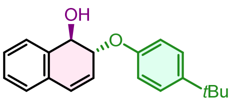

$C_{20}H_{22}O_2$
294,39 g/mol

Following **G. P. E** vial containing **Rh5ba** (4 mg, 3 mol%) was charged to glovebox and into reaction vial added oxabenzonorbornadiene (28 mg, 0.19 mmol, 1 eq.) and 4-*tert*-butylphenol (146 mg, 0.97 mmol, 5 eq.) followed by dry ACN (0.39 mL) reaction mixture was stirred at 80 °C for 48 h. After reaction was completed vial was taken from glovebox and concentrated under reduced pressure, from the crude reaction mixture prepared NMR sample. Column chromatography (SiO_2, petroleum ether/Et_2O 85:15, v/v) of the crude mixture gave **7ng** as a white solid (37 mg, 65%). The *ee* was determined to be 79 % using chiral HPLC (AD-H, *i*-propanol/*n*-hexane = 10/90, flow rate = 0.5 mL/min, l = 254 nm) t_R = 16.8 (minor), 21.4 (major).

Rf 0.22 (SiO_2, Hex/Et_2O 9:1, v/v).

^{1}H NMR (400 MHz, $CDCl_3$) δ 7.70 – 7.59 (m, 1H), 7.34 – 7.23 (m, 4H), 7.14 – 7.06 (m, 1H), 6.93 – 6.85 (m, 2H), 6.49 (dd, *J* = 9.9, 1.9 Hz, 1H), 6.03 (dd, *J* = 9.9, 2.1 Hz, 1H), 5.18 (d, *J* = 10.3 Hz, 1H), 5.09 (dt, *J* = 10.2, 2.1 Hz, 1H), 2.74 (s, 1H), 1.30 (s, 9H).

^{13}C{^{1}H} NMR (101 MHz, $CDCl_3$) δ 155.2, 144.4, 135.7, 132.1, 129.0, 128.3, 128.1, 126.6, 126.6, 126.5, 125.4, 115.4, 114.9, 104.4, 79.3, 72.6, 34.3, 31.6.

$[\alpha]^{25}_D$ = -55 (c = 1.00, $CHCl_3$).

IR (ATR neat) ṽ =: 3394, 3038, 2958, 2924, 2856, 1722, 1607, 1509, 1455 cm^{-1}.

HRMS (ESI): *m*/*z* calculated for $C_{20}H_{22}NaO_2^+$ [M + Na$^+$] 317.1512; found 317.1510.

$[\alpha]^{25}_D$ = -55 (c = 1.00, $CHCl_3$).

***ee* determination:**

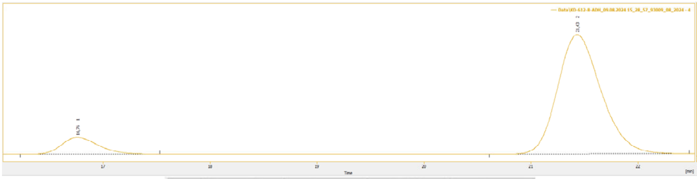

	Reten. Time [min]	Area [mAU.s]	Height [mAU]	Area [%]
1	16,762	768,570	32,641	10,5
2	21,434	6577,199	233,104	89,5
	Total	7345,769	265,745	100,0

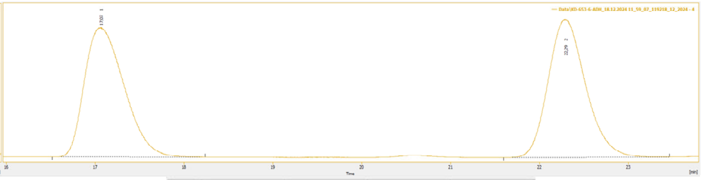

	Reten. Time [min]	Area [mAU.s]	Height [mAU]	Area [%]
1	17,065	37648,762	1220,155	49,8
2	22,293	37961,277	1301,549	50,2
	Total	75610,039	2521,704	100,0

(1*R*,2*R*)-2-([1,1'-biphenyl]-4-yloxy)-1,2-dihydronaphthalen-1-ol (7nh)

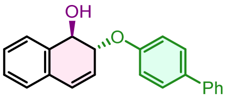

$C_{22}H_{18}O_2$
314,38 g/mol

Following **G. P. E** vial containing **Rh5ba** (4 mg, 3 mol%) was charged to glovebox and into reaction vial added oxabenzonorbornadiene (28 mg, 0.19 mmol, 1 eq.) and 4-phenylphenol (166 mg, 0.97 mmol, 5 eq.) followed by dry ACN (0.39 mL) reaction mixture was stirred at 80 °C for 48 h. After reaction was completed vial was taken from glovebox and concentrated under reduced pressure, from the crude reaction mixture prepared NMR sample. Column chromatography (SiO_2, petroleum ether/Et_2O 95:5, v/v) of the crude mixture gave **7nh** as a white solid (36 mg, 58%). The *ee* was determined to be 72 % using chiral HPLC (AD-H, *i*-propanol/*n*-hexane = 10/90, flow rate = 0.5 mL/min, l = 254 nm) t_R = 27.7 (minor), 38.8 (major).

Rf 0.32 (SiO_2, petroleum ether/Et_2O 8:2, v/v).

^{1}H NMR (400 MHz, $CDCl_3$) δ 7.68 (d, *J* = 6.3 Hz, 1H), 7.61 – 7.52 (m, 4H), 7.51 – 7.40 (m, 3H), 7.37 – 7.28 (m, 3H), 7.18 – 7.13 (m, 1H), 7.10 – 7.02 (m, 2H), 6.08 (dd, *J* = 9.8, 2.1 Hz, 1H), 5.24 (d, *J* = 10.1 Hz, 1H), 5.18 (dt, *J* = 10.1, 1.9 Hz, 1H), 2.55 (s, 1H).

^{13}C{^{1}H} NMR (101 MHz, $CDCl_3$) δ 157.1, 140.7, 135.6, 134.7, 132.0, 129.2, 128.9, 128.8, 128.5, 128.5, 128.4, 128.2, 126.9, 126.9, 126.8, 126.8, 126.6, 126.1, 125.4, 116.3, 115.8, 79.3, 72.5.

$[\alpha]^{25}_D$ = -54 (c = 1.00, $CHCl_3$).

The spectral data were consistent with the literature.[31]

***ee* determination:**

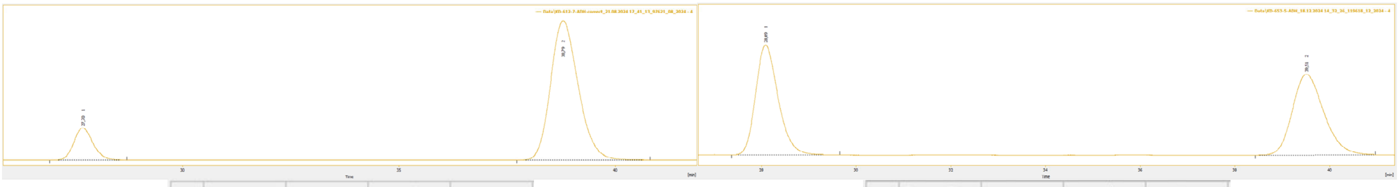

	Reten. Time [min]	Area [mAU.s]	Height [mAU]	Area [%]
1	27,701	3561,415	114,992	13,8
2	38,786	22269,396	501,417	86,2
	Total	25830,812	616,409	100,0

	Reten. Time [min]	Area [mAU.s]	Height [mAU]	Area [%]
1	28,085	27788,949	843,022	49,5
2	39,511	28300,148	619,104	50,5
	Total	56089,097	1462,126	100,0

1-(4-(((1*R*,2*R*)-1-hydroxy-1,2-dihydronaphthalen-2-yl)oxy)phenyl)ethan-1-one (7ni)

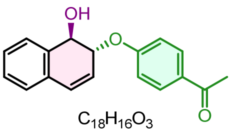

$C_{18}H_{16}O_3$
280,32 g/mol

Following **G. P. E** vial containing **Rh5ba** (4 mg, 3 mol%) was charged to glovebox and into reaction vial added oxabenzonorbornadiene (28 mg, 0.19 mmol, 1 eq.) and 4-hydroxyacetophenone (132 mg, 0.97 mmol, 5 eq.) followed by dry THF (0.39 mL) reaction mixture was stirred at 80 °C for 48 h. After reaction was completed vial was taken from glovebox and concentrated under reduced pressure, from the crude reaction mixture prepared NMR sample. Column chromatography (SiO_2, petroleum ether/EtOAc 8:2, v/v) of the crude mixture gave **7ni** as an colorless oil (29 mg, 54%). The *ee* was determined to be 78 % using chiral HPLC (AD-H, *i*-propanol/*n*-hexane = 15/85, flow rate = 1.0 mL/min, l = 254 nm) t_R = 18.8 (minor), 46.9 (major).

Rf 0.17 (SiO_2, petroleum ether/Et_2O 8:2, v/v).

^{1}H NMR (400 MHz, $CDCl_3$) δ 7.95 – 7.86 (m, 2H), 7.64 (dd, *J* = 5.7, 3.0 Hz, 1H), 7.34 – 7.23 (m, 2H), 7.17 – 7.08 (m, 1H), 6.96 (dd, *J* = 9.4, 2.4 Hz, 2H), 6.54 (d, *J* = 9.8 Hz, 1H), 5.97 (d, *J* = 9.8 Hz, 1H), 5.20 (s, 2H), 3.08 (s, 1H), 2.53 (s, 3H).

^{13}C{^{1}H} NMR (101 MHz, $CDCl_3$) δ 197.0, 161.6, 135.5, 131.8, 130.8, 130.8, 129.7, 128.5, 128.3, 126.7, 125.6, 125.2, 115.3, 79.2, 72.2, 26.4.

$[\alpha]^{25}_D$ = -205 (c = 1.00, $CHCl_3$).

The spectral data were consistent with the literature.[32]

***ee* determination:**

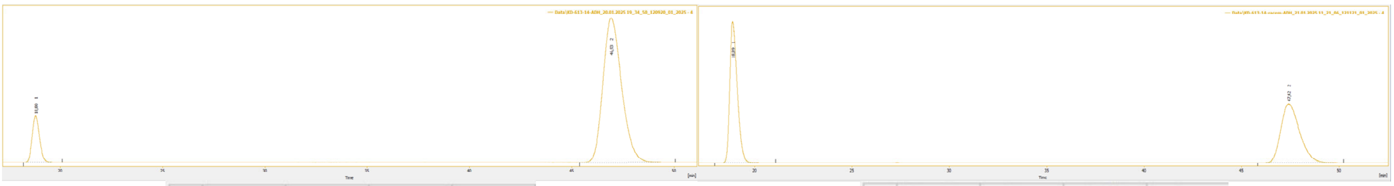

	Reten. Time [min]	Area [mAU.s]	Height [mAU]	Area [%]
1	18,803	4023,715	161,666	11,2
2	46,930	31840,255	500,755	88,8
	Total	35863,969	662,421	100,0

	Reten. Time [min]	Area [mAU.s]	Height [mAU]	Area [%]
1	18,888	26972,366	996,570	49,1
2	47,423	27939,540	416,902	50,9
	Total	54911,906	1413,472	100,0

N-(4-(((1*R*,2*R*)-1-hydroxy-1,2-dihydronaphthalen-2-yl)oxy)phenyl)acetamide (7nj)

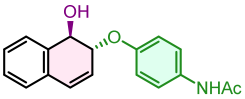

$C_{18}H_{17}NO_3$
295,34 g/mol

Following **G. P. E** vial containing **Rh5ba** (4 mg, 3 mol%) was charged to glovebox and into reaction vial added oxabenzonorbornadiene (28 mg, 0.19 mmol, 1 eq.) and 4-acetaminophenol (147 mg, 0.97 mmol, 5 eq.) followed by dry THF (0.39 mL) reaction mixture was stirred at 80 °C for 48 h. After reaction was completed vial was taken from glovebox and concentrated under reduced pressure, from the crude reaction mixture prepared NMR sample. Column chromatography (SiO_2, DCM/MeOH 9:1, v/v) of the crude mixture gave **7nj** as a white solid (50 mg, 90%). The *ee* was determined to be 84 % using chiral HPLC (AD-H, *i*-propanol/ethanol = 15/85, flow rate = 1.0 mL/min, l = 254 nm) t_R = 20.7 (minor), 38.0 (major).

Rf 0.5 (SiO_2, DCM/MeOH 9:1, v/v).

^{1}H NMR (400 MHz, MeOD) δ 7.56 (dt, *J* = 5.1, 3.4 Hz, 1H), 7.46 – 7.40 (m, 2H), 7.26 (pt, *J* = 4.6, 2.4 Hz, 2H), 7.16 – 7.09 (m, 1H), 6.99 – 6.91 (m, 2H), 6.55 (dd, *J* = 9.9, 1.3 Hz, 1H), 6.00 (dd, *J* = 9.9, 2.1 Hz, 1H), 5.07 – 4.96 (m, 2H), 2.09 (s, 3H).

$^{13}C\{^1H\}$ NMR (101 MHz, MeOD) δ 171.4, 155.9, 137.8, 133.5, 133.4, 130.1, 129.0, 127.6, 127.4, 126.9, 123.1, 117.3, 80.2, 72.8, 23.6.

$[\alpha]^{25}_D$ = -101 (c = 1.00, MeOH).

IR (ATR neat) ṽ =: 3245, 3040, 2929, 2853, 1650, 1597, 1408, 1368 cm^{-1}.

HRMS (ESI): *m/z* calculated for $C_{18}H_{17}NNaO_3^+$ [M + Na$^+$] 318.1101; found 318.1106.

***ee* determination:**

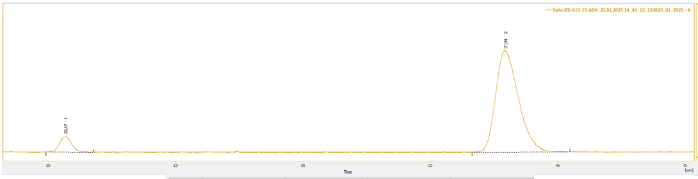

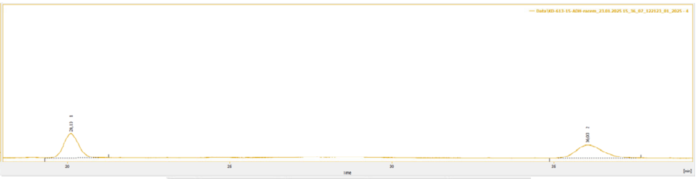

	Reten. Time [min]	Area [mAU.s]	Height [mAU]	Area [%]
1	20,667	1124,095	33,001	8,1
2	37,942	12802,390	203,892	91,9
	Total	13926,484	236,893	100,0

	Reten. Time [min]	Area [mAU.s]	Height [mAU]	Area [%]
1	20,104	1959,549	60,413	50,0
2	36,030	1961,674	33,386	50,0
	Total	3921,223	93,798	100,0

methyl 4-(((1*R*,2*R*)-1-hydroxy-1,2-dihydronaphthalen-2-yl)oxy)benzoate (7nk)

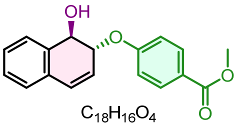

$C_{18}H_{16}O_4$
296,32 g/mol

Following **G. P. E** vial containing **Rh5ba** (4 mg, 3 mol%) was charged to glovebox and into reaction vial added oxabenzonorbornadiene (28 mg, 0.19 mmol, 1 eq.) and methyl 4-hydroxybenzoate (147 mg, 0.97 mmol, 5 eq.) followed by dry THF (0.39 mL) reaction mixture was stirred at 80 °C for 48 h. After reaction was completed vial was taken from glovebox and concentrated under reduced pressure, from the crude reaction mixture prepared NMR sample. Column chromatography (SiO_2, petroleum ether/Et_2O 9:1, v/v) of the crude mixture gave **7nk** as a white solid (17.5 mg, 30%). The *ee* was determined to be 84 % using chiral HPLC (OD-H, *i*-propanol/*n*-hexane = 10/90, flow rate = 0.5 mL/min, l = 254 nm) t_R = 37.6 (minor), 54.5 (major).

Rf 0.24 (SiO_2, petroleum ether/EtOAc 9:1, v/v).

^1H NMR (400 MHz, $CDCl_3$) δ 8.06 – 7.97 (m, 2H), 7.67 – 7.61 (m, 1H), 7.35 – 7.27 (m, 2H), 7.19 – 7.09 (m, 1H), 7.01 – 6.93 (m, 2H), 6.56 (d, *J* = 9.7 Hz, 1H), 5.99 (dd, *J* = 9.9, 1.5 Hz, 1H), 5.25 – 5.14 (m, 2H), 3.89 (s, 3H), 2.60 (s, 1H).

$^{13}C\{^1H\}$ NMR (101 MHz, $CDCl_3$) δ 166.9, 161.4, 135.4, 131.9, 131.9, 129.8, 128.5, 128.3, 126.8, 125.5, 125.3, 123.4, 115.3, 79.3, 72.3, 52.1.

$[\alpha]^{25}_D$ = -28 (c = 1.00, $CHCl_3$).

IR (ATR neat) ṽ =: 3441, 3037, 2950, 2851, 1712, 1601, 1578 cm^{-1}.

HRMS (ESI): *m/z* calculated for $C_{18}H_{16}NaO_4^+$ [M + Na$^+$] 319.0941; found 319.0934.

***ee* determination:**

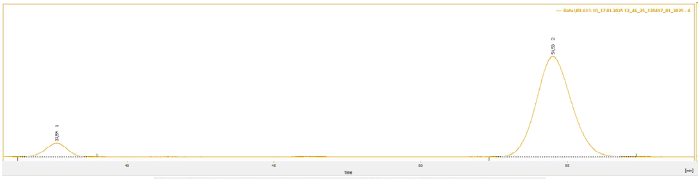

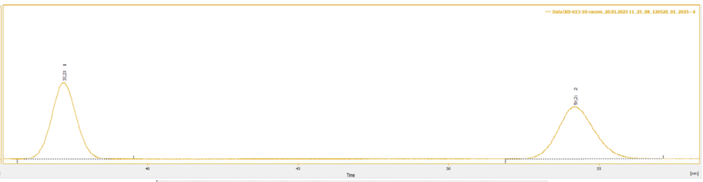

	Reten. Time [min]	Area [mAU.s]	Height [mAU]	Area [%]
1	37,589	3636,703	66,126	8,2
2	54,503	40866,568	486,434	91,8
	Total	44503,271	552,561	100,0

	Reten. Time [min]	Area [mAU.s]	Height [mAU]	Area [%]
1	37,232	4429,457	76,022	49,9
2	54,214	4455,647	51,821	50,1
	Total	8885,104	127,843	100,0

methyl 2-(4-(((1*R*,2*R*)-1-hydroxy-1,2-dihydronaphthalen-2-yl)oxy)phenyl)acetate (7nl)

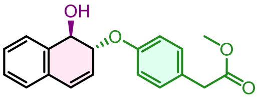

$C_{19}H_{18}O_4$
310,35 g/mol

Following **G. P. E** vial containing **Rh5ba** (4 mg, 3 mol%) was charged to glovebox and into reaction vial added oxabenzonorbornadiene (28 mg, 0.19 mmol, 1 eq.) and methyl 4-hydroxyphenylacetate (162 mg, 0.97 mmol, 5 eq.) followed by dry THF (0.39 mL) reaction mixture was stirred at 80 °C for 48 h. After reaction was completed vial was taken from glovebox and concentrated under reduced pressure, from the crude reaction

mixture prepared NMR sample. Column chromatography (SiO_2, petroleum ether/EtOAc 85:15, v/v) of the crude mixture gave **7nl** as a white solid (48 mg, 80%). The *ee* was determined to be 78 % using chiral HPLC (AD-H, *i*-propanol/*n*-hexane = 15/85, flow rate = 1.0 mL/min, l = 254 nm) t_R = 14.1 (minor), 29.1 (major).

Rf 0.20 (SiO_2, petroleum ether/EtOAc 8:2, v/v).

^{1}H NMR (400 MHz, $CDCl_3$) δ 7.64 (d, *J* = 6.1 Hz, 1H), 7.29 (tt, *J* = 8.2, 3.9 Hz, 2H), 7.21 (d, *J* = 8.6 Hz, 2H), 7.16 – 7.09 (m, 1H), 6.92 (d, *J* = 8.7 Hz, 2H), 6.51 (dd, *J* = 9.9, 1.6 Hz, 1H), 6.01 (dd, *J* = 9.8, 2.1 Hz, 1H), 5.18 (d, *J* = 10.1 Hz, 1H), 5.09 (dt, *J* = 10.0, 1.9 Hz, 1H), 3.70 (s, 3H), 3.58 (s, 2H), 2.84 (d, *J* = 2.9 Hz, 1H).

^{13}C{^{1}H} NMR (101 MHz, $CDCl_3$) δ 172.4, 156.6, 135.7, 132.0, 130.6, 129.1, 128.3, 128.1, 127.0, 126.5, 126.1, 125.4, 116.0, 79.2, 72.4, 52.1, 40.3.

$[\alpha]^{25}_D$ = -115 (c = 1.00, $CHCl_3$).

IR (ATR neat) ṽ =: 3298, 3070, 2954, 2922, 2849, 1732, 1612, 1510, 1457, 1439 cm^{-1}.

HRMS (ESI): *m*/*z* calculated for $C_{19}H_{18}NaO_4^+$ [M + Na^+] 333.1097; found 333.1098.

***ee* determination:**

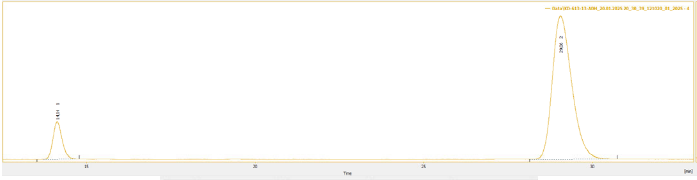

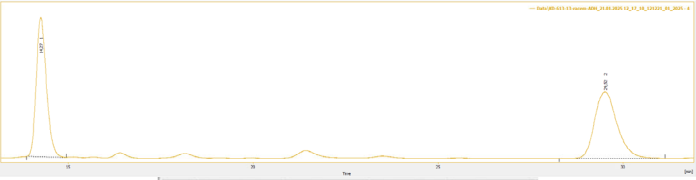

	Reten. Time [min]	Area [mAU.s]	Height [mAU]	Area [%]
1	14,135	2739,957	146,280	10,9
2	29,062	22323,045	563,300	89,1
	Total	25063,002	709,581	100,0

	Reten. Time [min]	Area [mAU.s]	Height [mAU]	Area [%]
1	14,265	13271,975	689,151	49,3
2	29,518	13662,283	324,415	50,7
	Total	26934,258	1013,566	100,0

(1*R*,2*R*)-2-(3-chlorophenoxy)-1,2-dihydronaphthalen-1-ol (7o)

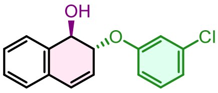

$C_{16}H_{13}ClO_2$
272,73 g/mol

Following **G. P. E** vial containing **Rh5ba** (4 mg, 3 mol%) was charged to glovebox and into reaction vial added oxabenzonorbornadiene (28 mg, 0.19 mmol, 1 eq.) and 3-chlorophenol (125 mg, 0.97 mmol, 5 eq.) followed by dry THF (0.39 mL) reaction mixture was stirred at 80 °C for 48 h. After reaction was completed vial was taken from glovebox and concentrated under reduced pressure, from the crude reaction mixture prepared NMR sample. Column chromatography (SiO_2, petroleum ether/Et_2O 9:1, v/v) of the crude mixture gave **7o** as a white solid (11 mg, 21%). The *ee* was determined to be 83 % using chiral HPLC (AD-H, *i*-propanol/*n*-hexane = 10/90, flow rate = 1.0 mL/min, l = 254 nm) t_R = 9.5 (minor), 12.5 (major).

Rf 0.25 (SiO_2, petroleum ether/EtOAc 9:1, v/v).

^{1}H NMR (400 MHz, $CDCl_3$) δ 7.68 – 7.57 (m, 1H), 7.35 – 7.27 (m, 2H), 7.28 – 7.17 (m, 1H), 7.19 – 7.09 (m, 1H), 7.00 – 6.95 (m, 2H), 6.85 (ddd, *J* = 8.4, 2.3, 1.0 Hz, 1H), 6.54 (dd, *J* = 9.9, 1.9 Hz, 1H), 5.99 (dd, *J* = 9.9, 2.2 Hz, 1H), 5.18 (d, *J* = 10.0 Hz, 1H), 5.09 (dt, *J* = 9.9, 2.0 Hz, 1H), 2.56 (s, 1H).

^{13}C{^{1}H} NMR (101 MHz, $CDCl_3$) δ 158.3, 135.5, 135.3, 131.9, 130.6, 129.6, 128.5, 128.3, 126.7, 125.5, 125.5, 121.8, 116.5, 114.2, 79.5, 72.4.

$[\alpha]^{25}_D$ = -105 (c = 1.00, $CHCl_3$).

The spectral data were consistent with the literature.[31]

***ee* determination:**

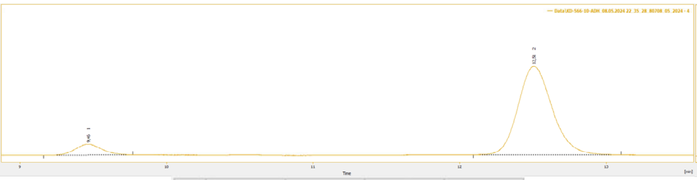

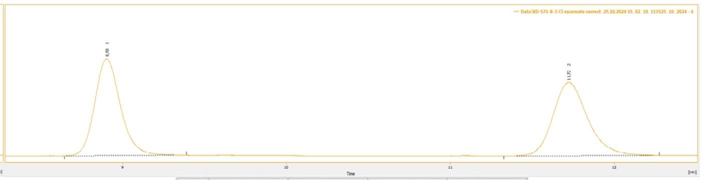

	Reten. Time [min]	Area [mAU.s]	Height [mAU]	Area [%]
1	9,464	201,225	16,086	8,6
2	12,507	2146,163	133,109	91,4
	Total	2347,388	149,196	100,0

	Reten. Time [min]	Area [mAU.s]	Height [mAU]	Area [%]
1	8,903	2680,564	247,381	49,5
2	11,723	2732,688	187,228	50,5
	Total	5413,253	434,608	100,0

(1*R*,2*R*)-2-(furan-2-ylmethoxy)-1,2-dihydronaphthalen-1-ol (7p)

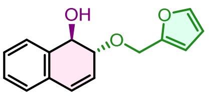

$C_{15}H_{14}O_3$
242,27 g/mol

Following **G. P. E** vial containing **Rh5ba** (4 mg, 3 mol%) was charged to glovebox and into reaction vial added oxabenzonorbornadiene (28 mg, 0.19 mmol, 1 eq.) and 3-chlorophenol (85 µL, 0.97 mmol, 5 eq.) followed by dry THF (0.39 mL) reaction mixture was stirred at 80 °C for 48 h. After reaction was completed vial was taken from glovebox and concentrated under reduced pressure, from the crude reaction mixture prepared NMR sample. Column chromatography (SiO_2, pentane/Et_2O 8:2, v/v) of the crude mixture gave **7p** as a white solid (13 mg, 28%). The *ee* was determined to be 69 % using chiral HPLC (OD-H, *i*-propanol/*n*-hexane = 10/90, flow rate = 0.5 mL/min, l = 254 nm) t_R = 14.6 (major), 15.5 (minor).

Rf 0.30 (SiO_2, pentane/Et_2O 6:4, v/v).

^{1}H NMR (400 MHz, $CDCl_3$) δ 7.61 – 7.53 (m, 1H), 7.44 (t, *J* = 1.4 Hz, 1H), 7.29 – 7.19 (m, 2H), 7.06 (dd, *J* = 7.1, 1.7 Hz, 1H), 6.44 (dd, *J* = 9.9, 2.1 Hz, 1H), 6.35 (d, *J* = 1.4 Hz, 2H), 5.96 (dd, *J* = 9.9, 2.2 Hz, 1H), 4.94 (dd, *J* = 10.5, 2.3 Hz, 1H), 4.71 (d, *J* = 13.0 Hz, 1H), 4.62 (d, *J* = 13.0 Hz, 1H), 4.32 (dt, *J* = 10.5, 2.2 Hz, 1H), 2.63 (d, *J* = 3.4 Hz, 1H).

^{13}C{^{1}H} NMR (101 MHz, $CDCl_3$) δ 151.5, 143.2, 135.9, 132.1, 128.5, 128.1, 127.9, 127.6, 126.4, 125.1, 110.6, 109.8, 80.6, 72.8, 63.6.

$[\alpha]^{25}_D$ = -45 (c = 1.00, $CHCl_3$).

IR (ATR neat) ṽ =: 3420, 3064, 3037, 2956, 2923, 1722, 1454, 1149, 1073, 1050 cm^{-1}.

HRMS (ESI): *m*/*z* calculated for $C_{15}H_{14}NaO_3^+$ [M + Na^+] 265.0825; found 265.0826.

***ee* determination:**

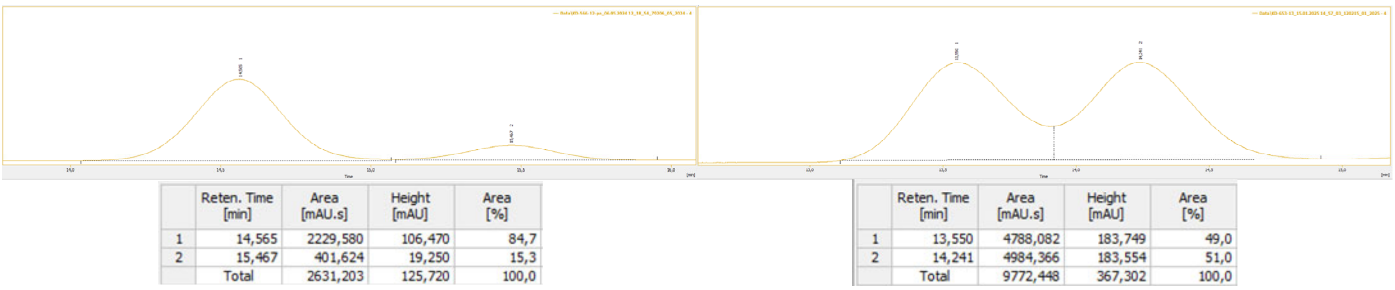

	Reten. Time [min]	Area [mAU.s]	Height [mAU]	Area [%]
1	14,565	2229,580	106,470	84,7
2	15,467	401,624	19,250	15,3
	Total	2631,203	125,720	100,0

	Reten. Time [min]	Area [mAU.s]	Height [mAU]	Area [%]
1	13,550	4788,082	183,749	49,0
2	14,241	4984,366	183,554	51,0
	Total	9772,448	367,302	100,0

(1*R*,2*R*)-2-(4-((*E*)-styryl)phenoxy)-1,2-dihydronaphthalen-1-ol (7r)

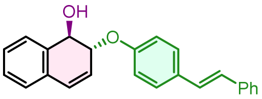

$C_{24}H_{20}O_2$
340,42 g/mol

Following **G. P. E** vial containing **Rh5ba** (4 mg, 3 mol%) was charged to glovebox and into reaction vial added oxabenzonorbornadiene (28 mg, 0.19 mmol, 1 eq.) and *trans*-4-Hydroxystilbene (190 mg, 0.97 mmol, 5 eq.) followed by dry THF (0.39 mL) reaction mixture was stirred at 80 °C for 48 h. After reaction was completed vial was taken from glovebox and concentrated under reduced pressure, from the crude reaction mixture prepared NMR sample. Column chromatography (SiO_2, petroleum ether/Et_2O 9:1, v/v) of the crude mixture gave **7r** as a white solid (54 mg, 67%). The *ee* was determined to be 67 % using chiral HPLC (AD-H, *i*-propanol/*n*-hexane = 15/85, flow rate = 1.0 mL/min, l = 254 nm) t_R = 16.4 (minor), 35.5 (major).

Rf 0.75 (SiO_2, petroleum ether/DCM 1:1, v/v).

^{1}H NMR (400 MHz, $CDCl_3$) δ 7.68 – 7.63 (m, 1H), 7.53 – 7.45 (m, 4H), 7.36 (t, *J* = 7.6 Hz, 2H), 7.33 – 7.27 (m, 2H), 7.27 – 7.22 (m, 1H), 7.17 – 7.12 (m, 1H), 7.05 (dd, *J* = 16.8, 16.3 Hz, 2H), 7.01 – 6.92 (m, 2H), 6.54 (dd, *J* = 9.9, 1.9 Hz, 1H), 6.04 (dd, *J* = 9.9, 2.1 Hz, 1H), 5.21 (dd, *J* = 10.1, 2.9 Hz, 1H), 5.14 (dt, *J* = 10.1, 2.0 Hz, 1H), 2.60 (d, *J* = 3.7 Hz, 1H).

^{13}C{^{1}H} NMR (101 MHz, $CDCl_3$) δ 157.2, 137.7, 135.6, 132.0, 131.1, 129.3, 128.8, 128.4, 128.2, 128.1, 128.1, 127.5, 127.3, 126.7, 126.4, 126.1, 125.4, 116.2, 79.3, 72.5.

$[\alpha]^{25}_D$ = -21 (c = 0.25, $CHCl_3$).

IR (ATR neat) ṽ: 3370, 3054, 3023, 2925, 2853, 1604, 1595, 1571, 1508, 1451 cm^{-1}.

HRMS (ESI): *m*/*z* calculated for $C_{24}H_{20}NaO_2^+$ [M + Na^+] 363.1356; found 363.1355.

***ee* determination:**

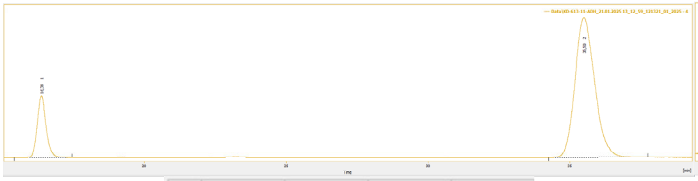

	Reten. Time [min]	Area [mAU.s]	Height [mAU]	Area [%]
1	16,375	3292,696	153,640	16,4
2	35,501	16818,606	349,334	83,6
	Total	20111,302	502,974	100,0

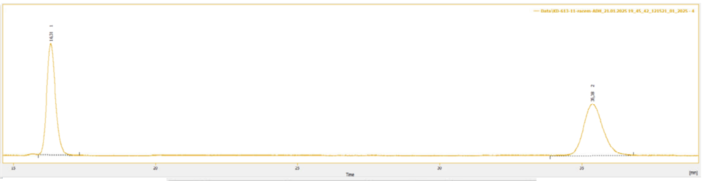

	Reten. Time [min]	Area [mAU.s]	Height [mAU]	Area [%]
1	16,313	1667,498	77,566	49,4
2	35,376	1710,117	36,007	50,6
	Total	3377,615	113,574	100,0

5.3 Prepared modifications for product **7ab**.

5.3.1 Scale up for synthesis with **7ab**.

(1*R*,2*R*)-2-((4-chlorophenyl)(methyl)amino)-1,2-dihydronaphthalen-1-ol (7ab)

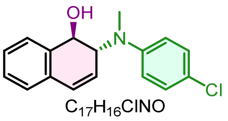

$C_{17}H_{16}ClNO$
285,77 g/mol

Following **G. P. E** vial containing **Rh5ba** (45 mg, 3 mol%) was charged to glovebox and into reaction vial added oxabenzonorbornadiene (300 mg, 2.08 mmol, 1 eq.) and 4-chloro-*N*-methylaniline (1.26 mL, 10.4 mmol, 5 eq.) followed by dry MeTHF (4.2 mL) reaction mixture was stirred at 80 °C for 20 h. After reaction was completed vial was taken from glovebox and concentrated under reduced pressure. Column chromatography (SiO_2, Hex/Et_2O 9:1, followed by Hex/Et_2O 3:1, v/v) of the crude mixture gave **7ab** as a light yellow oil (475 mg, 80%). The *ee* was determined to be 83 % using chiral HPLC (AD-H, *i*-propanol/*n*-hexane = 5/95, flow rate = 0.5 mL/min, l = 254 nm) t_R = 31.4 (major), 36.0 (minor).

***ee* determination:**

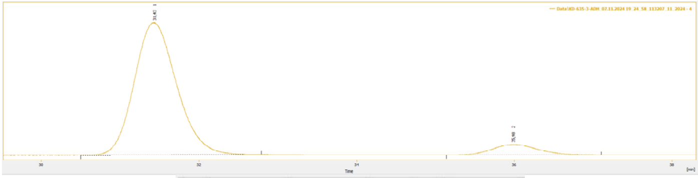

	Reten. Time [min]	Area [mAU.s]	Height [mAU]	Area [%]
1	31,435	33016,551	884,238	91,7
2	35,985	2975,666	71,242	8,3
	Total	35992,216	955,480	100,0

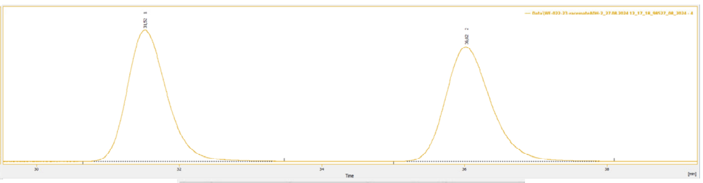

	Reten. Time [min]	Area [mAU.s]	Height [mAU]	Area [%]
1	31,518	23108,408	601,634	50,0
2	36,023	23130,727	524,982	50,0
	Total	46239,135	1126,616	100,0

5.3.2 Reduction of **7ab**.

(1*R*,2*R*)-2-((4-chlorophenyl)(methyl)amino)-1,2,3,4-tetrahydronaphthalen-1-ol (8a)

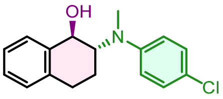

$C_{17}H_{18}ClNO$
287,79 g/mol

In a 10 mL vial, **7ab** (26 mg, 0.091 mmol, 1 eq.) and $Rh(PPh_3)_3Cl$ (5 mg, 4.56 μmol, 5 mol%) were dissolved in dry and degassed THF (2 mL). This solution was transferred to a reaction flask in laminar and subjected to 25 bar of H_2 at room temperature for 16 hours. After the reaction, the solution was filtered through a short pad of Celite and washed with THF. The solvent was removed under reduced pressure to obtain the crude product. Column chromatography (SiO_2, Hex/Et_2O 9:1, v/v) of the crude mixture yielded **8a** as a light yellow oil (25 mg, 96%). The *ee* was determined to be 82 % using chiral HPLC (AD-H, *i*-propanol/*n*-hexane = 5/95, flow rate = 0.5 mL/min, l = 254 nm) t_R = 28.3 (major), 43.8 (minor).

Rf 0.91 (SiO_2, petroleum ether/EtOAc 1:1, v/v).

^{1}H NMR (400 MHz, $CDCl_3$) δ 7.67 (d, *J* = 7.5 Hz, 1H), 7.30 – 7.21 (m, 2H), 7.21 – 7.17 (m, 2H), 7.11 (d, *J* = 7.5 Hz, 1H), 6.94 – 6.87 (m, 2H), 4.95 (d, *J* = 9.7 Hz, 1H), 3.94 – 3.74 (m, 1H), 3.04 – 2.88 (m, 2H), 2.84 (s, 3H), 2.76 (s, 1H), 1.99 – 1.86 (m, 2H).

^{13}C{^{1}H} NMR (101 MHz, $CDCl_3$) δ 150.1, 137.8, 135.2, 129.1, 128.4, 127.4, 127.2, 126.5, 123.5, 116.8, 69.6, 64.9, 31.3, 29.5, 23.8.

$[\alpha]^{25}_D$ = +137 (c = 1.00, $CHCl_3$).

IR (ATR neat) ṽ: 3412, 2922, 2852, 1594, 1494, 1454, 1434, 1396, 1343, 1300 cm^{-1}.

HRMS (ESI): *m*/*z* calculated for $C_{17}H_{18}ClNNaO^+$ [M + Na^+] 310.0969; found 310.0962.

***ee* determination:**

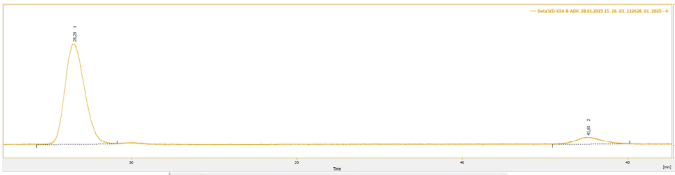

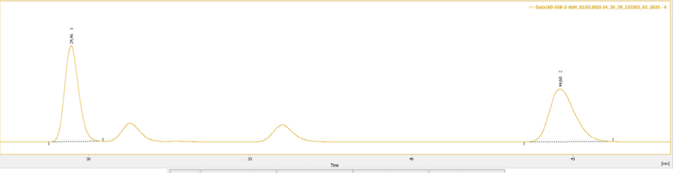

	Reten. Time [min]	Area [mAU.s]	Height [mAU]	Area [%]
1	28,277	3374,576	81,771	91,2
2	43,809	323,894	5,731	8,8
	Total	3698,470	87,503	100,0

	Reten. Time [min]	Area [mAU.s]	Height [mAU]	Area [%]
1	29,457	14760,273	485,768	50,0
2	44,601	14735,819	268,840	50,0
	Total	29496,092	754,608	100,0

5.3.3 Friedel-Crafts reaction of **8a**.

(1*S*,2*R*)-*N*-(4-chlorophenyl)-1-(4-methoxyphenyl)-*N*-methyl-1,2,3,4-tetrahydronaphthalen-2-amine (8b)

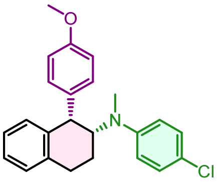

$C_{24}H_{24}ClNO$
377,91 g/mol

In a 10 mL vial, **8a** (26 mg, 0.093 mmol, 1 eq.) was dissolved in dry DCM (0.4 mL). To this solution, anisole (0.2 mL, 1.87 mmol, 20 eq.) was added, followed by $AlCl_3$ (25 mg, 0.187 mmol, 2 eq.). The solution was stirred at room temperature for 16 hours. After the reaction, the solution was diluted with DCM and extracted with aqueous $NaHCO_3$. The aqueous phase was washed three times with DCM. The combined organic fractions were dried with $MgSO_4$ and filtered. The solvent was removed under reduced pressure to obtain the crude product. Column chromatography (SiO_2, pentane/Et_2O 30:1, followed by 95:5, v/v) of the crude mixture yielded **8b** as an oil (22 mg, 63%). The *ee* was determined to be 84 % using chiral HPLC (AD-H, *i*-propanol/*n*-hexane = 1/99, flow rate = 0.5 mL/min, l = 254 nm) t_R = 10.7 (major), 12.4 (minor).

Rf 0.54 (SiO_2, pentane/Et_2O 95:5, v/v).

^{1}H NMR (400 MHz, $CDCl_3$) δ 7.13 (dd, *J* = 4.1, 1.5 Hz, 2H), 7.08 – 6.94 (m, 5H), 6.83 (d, *J* = 7.5 Hz, 1H), 6.78 – 6.73 (m, 2H), 6.44 – 6.36 (m, 2H), 4.25 (d, *J* = 10.3 Hz, 1H), 4.04 (td, *J* = 10.8, 3.8 Hz, 1H), 3.75 (s, 3H), 3.07 (ddd, *J* = 16.8, 11.6, 5.5 Hz, 1H), 2.97 (ddd, *J* = 16.6, 4.8, 2.9 Hz, 1H), 2.81 (s, 3H), 2.12 – 1.96 (m, 2H).

^{13}C{^{1}H} NMR (101 MHz, $CDCl_3$) δ 158.3, 149.3, 140.1, 136.6, 136.4, 130.6, 130.2, 128.6, 128.5, 126.1, 126.1, 121.5, 114.9, 113.9, 64.5, 55.3, 48.8, 31.5, 30.3, 26.7.

$[\alpha]^{25}_D$ = +62 (c = 1.00, $CHCl_3$).

IR (ATR neat) ṽ: 2922, 2852, 1726, 1610, 1594, 1509, 1495, 1452, 1389, 1342, 1301 cm^{-1}.

HRMS (ESI): *m*/*z* calculated for $C_{24}H_{24}ClNNaO^+$ [M + Na^+] 400.1439; found 400.1446.

***ee* determination:**

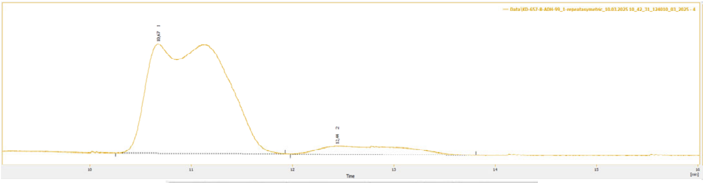

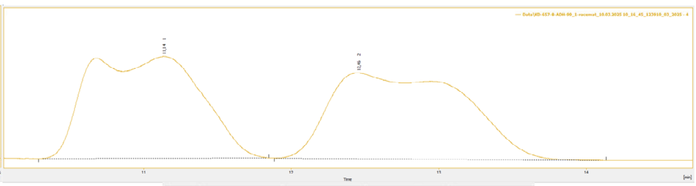

	Reten. Time [min]	Area [mAU.s]	Height [mAU]	Area [%]
1	10,672	6513,030	121,187	91,9
2	12,440	574,266	9,174	8,1
	Total	7087,296	130,360	100,0

	Reten. Time [min]	Area [mAU.s]	Height [mAU]	Area [%]
1	11,138	29787,718	555,106	49,8
2	12,456	30012,907	468,402	50,2
	Total	59800,625	1023,508	100,0

5.3.4 Acetylation of **7ab**.

(1*R*,2*R*)-2-((4-chlorophenyl)(methyl)amino)-1,2-dihydronaphthalen-1-yl acetate (8c)

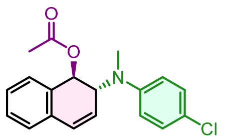

$C_{19}H_{18}ClNO_2$
327,81 g/mol

In a 10 mL vial, **7ab** (80 mg, 0.28 mmol, 1 eq.) was dissolved in Ac_2O (2 mL) under a nitrogen atmosphere. The solution was stirred for 10 minutes at room temperature and then dry NaOAc (69 mg, 0.84 mmol, 3 eq.) was added. The reaction was carried out at 90 °C (oil bath) for 24 hours. After the reaction was finished, the mixture was cooled down to 0 °C and quenched by the addition of 1 M HCl (2 mL), and stirred for another 10 minutes. The extraction was performed using EtOAc (3 x 30 mL), and the combined organic fractions were washed with an aqueous solution of $NaHCO_3$. The organic fraction was then dried with Na_2SO_4, filtered, and the solvent was removed under reduced pressure. This product was then dried under high vacuum to provide **8c** as a light yellow oil (92 mg, 100%). The *ee* was determined to be 84 % using chiral HPLC (AD-H, *i*-propanol/*n*-hexane = 5/95, flow rate = 0.5 mL/min, l = 254 nm) t_R = 12.0 (major), 14.8 (minor).

^{1}H NMR (400 MHz, $CDCl_3$) δ 7.29 (td, *J* = 7.3, 1.0 Hz, 1H), 7.23 (td, *J* = 7.4, 1.4 Hz, 1H), 7.21 – 7.10 (m, 4H), 6.82 – 6.73 (m, 2H), 6.63 (dd, *J* = 9.8, 2.5 Hz, 1H), 6.38 (d, *J* = 9.6 Hz, 1H), 5.89 (dd, *J* = 9.8, 3.0 Hz, 1H), 4.93 (dt, *J* = 9.6, 2.8 Hz, 1H), 2.78 (s, 3H), 1.84 (s, 3H).

^{13}C{^{1}H} NMR (101 MHz, $CDCl_3$) δ 170.6, 148.5, 133.1, 130.1, 129.0, 128.7, 128.2, 126.9, 125.9, 115.0, 71.9, 60.6, 33.3, 21.0.

$[\alpha]^{25}_D$ = +12 (c = 1.00, $CHCl_3$).

IR (ATR neat) ṽ: 3039, 2917, 2817, 1740, 1594, 1494, 1452, 1368, 1300, 1265 cm^{-1}.

HRMS (ESI): *m*/*z* calculated for $C_{19}H_{18}ClNNaO_2^+$ [M + Na$^+$] 350.0918; found 350.0925.

***ee* determination:**

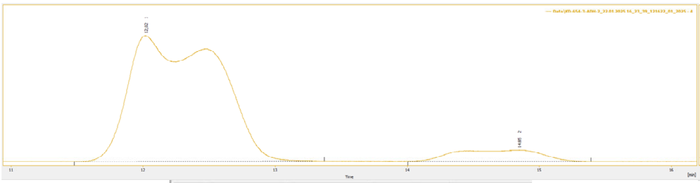

	Reten. Time [min]	Area [mAU.s]	Height [mAU]	Area [%]
1	12,022	15206,979	321,079	91,8
2	14,849	1362,609	29,070	8,2
	Total	16569,588	350,149	100,0

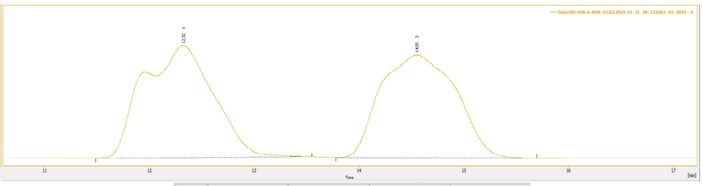

	Reten. Time [min]	Area [mAU.s]	Height [mAU]	Area [%]
1	12,322	14199,744	304,759	50,3
2	14,552	14002,582	280,086	49,7
	Total	28202,326	584,845	100,0

5.3.5 Oxydation of **8c**.

(1*R*,2*R*,3*S*,4*R*)-2-((4-chlorophenyl)(methyl)amino)-3,4-dihydroxy-1,2,3,4-tetrahydronaphthalen-1-yl acetate (8d)

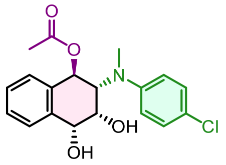

$C_{19}H_{20}ClNO_4$
361,82 g/mol

In a 10 mL vial, **8c** (28 mg, 0.085 mmol, 1 eq.) and NMO (50 mg, 0.425 mmol, 5 eq.) were dissolved in a mixture of *t*BuOH/THF/H_2O (7:2:1, 2 mL), and then OsO_4 (60 µL, 2.5% *t*BuOH) was added. The reaction vial was sealed with a Teflon cap and stirred at room temperature for 16 hours. After that, the solution was quenched with $Na_2S_2O_3$ and extracted with DCM (4 x 20 mL). The combined organic fractions were dried over $MgSO_4$, filtered, and the solvent was removed under reduced pressure. Column chromatography (SiO_2, petroleum ether/EtOAc 6:4, v/v) of the crude mixture yielded **8d** as a light yellow oil (26 mg, 84%). The *ee* was determined to be 83 % using chiral HPLC (AD-H, *i*-propanol/*n*-hexane = 5/95, flow rate = 0.5 mL/min, l = 254 nm) t_R = 78.1 (major), 82.4 (minor).

Rf 0.27 (SiO_2, petroleum ether/EtOAc 6:4, v/v).

^{1}H NMR (400 MHz, $CDCl_3$) δ 7.45 – 7.39 (m, 1H), 7.39 – 7.32 (m, 2H), 7.20 – 7.14 (m, 2H), 7.14 – 7.09 (m, 1H), 6.92 – 6.82 (m, 2H), 6.26 (d, *J* = 9.7 Hz, 1H), 5.00 (d, *J* = 3.4 Hz, 1H), 4.61 (dd, *J* = 10.8, 9.9 Hz, 1H), 4.03 (dd, *J* = 10.9, 3.3 Hz, 1H), 3.09 (s, 1H), 3.02 (s, 1H), 2.86 (s, 3H), 1.70 (s, 3H).

^{13}C{^{1}H} NMR (101 MHz, $CDCl_3$) δ 171.0, 150.1, 134.6, 134.1, 130.8, 129.4, 129.0, 128.9, 127.4, 123.7, 116.3, 70.3, 69.1, 67.9, 61.4, 31.0, 20.8.

$[\alpha]^{25}_D$ = -5 (c = 1.00, $CHCl_3$).

IR (ATR neat) ṽ: 3400, 2923, 1730, 1594, 1496, 1456, 1430, 1370, 1303, cm^{-1}.

HRMS (ESI): *m/z* calculated for $C_{19}H_{20}ClNNaO_4^+$ [M + Na^+] 384.0973; found 384.0976.

***ee* determination:**

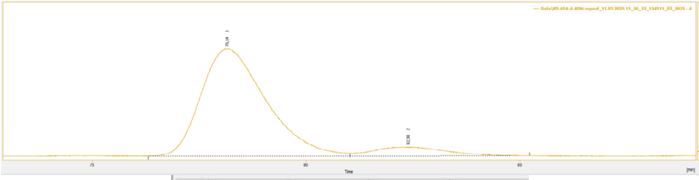

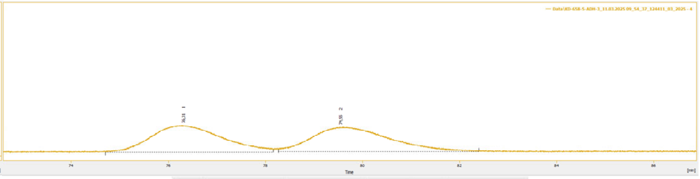

	Reten. Time [min]	Area [mAU.s]	Height [mAU]	Area [%]
1	78,143	28435,878	270,081	91,3
2	82,375	2724,119	22,313	8,7
	Total	31159,997	292,394	100,0

	Reten. Time [min]	Area [mAU.s]	Height [mAU]	Area [%]
1	76,310	2493,968	24,706	50,3
2	79,546	2464,877	22,525	49,7
	Total	4958,845	47,231	100,0

5.3.6 Reduction of **8c**.

(1*R*,2*R*)-2-((4-chlorophenyl)(methyl)amino)-1,2,3,4-tetrahydronaphthalen-1-yl acetate (8e)

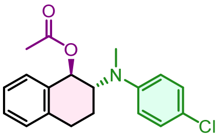

$C_{19}H_{20}ClNO_2$
329,82 g/mol

In a 10 mL vial, **8c** (58 mg, 0.177 mmol, 1 eq.) and $Rh(PPh_3)_3Cl$ (10 mg, 8.85 µmol, 5 mol%) were dissolved in dry and degassed THF (3 mL). This solution was transferred to a reaction flask in laminar and subjected to 25 bar of H_2 at room temperature for 16 hours. After the reaction, the solution was filtered through a short pad of Celite and washed with THF. The solvent was removed under reduced pressure to obtain the crude product. Column chromatography (SiO_2, Hex/Et_2O 95:5, v/v) of the crude mixture yielded **8e** as a light yellow oil (48 mg, 83%). The *ee* was determined to be 83 % using chiral HPLC (AD-H, *i*-propanol/*n*-hexane = 2/98, flow rate = 0.5 mL/min, l = 254 nm) t_R = 16.9 (major), 22.0 (minor).

Rf 0.24 (SiO_2, Hex/Et_2O 95:5, v/v).

^{1}H NMR (400 MHz, $CDCl_3$) δ 7.26 – 7.19 (m, 2H), 7.18 – 7.11 (m, 4H), 6.84 – 6.75 (m, 2H), 6.34 (d, *J* = 9.7 Hz, 1H), 4.15 (ddd, *J* = 11.5, 9.8, 3.8 Hz, 1H), 3.06 (ddt, *J* = 15.3, 9.3, 4.7 Hz, 1H), 3.02 – 2.91 (m, 1H), 2.80 (s, 3H), 2.16 – 2.00 (m, 2H), 1.84 (s, 3H).

^{13}C{^{1}H} NMR (101 MHz, $CDCl_3$) δ 171.3, 149.3, 136.5, 135.1, 128.8, 128.7, 127.9, 127.4, 126.5, 121.9, 115.0, 71.3, 60.5, 31.2, 29.7, 26.5, 21.0.

$[\alpha]^{25}_D$ = +90 (c = 1.00, $CHCl_3$).

IR (ATR neat) ṽ: 2925, 1731, 1593, 1494, 1368, 1345, 1306, 1227, 1205, 1169, 1117 cm^{-1}.

HRMS (ESI): *m/z* calculated for $C_{19}H_{20}ClNNaO_2^+$ [M + Na^+] 352.1075; found 352.1077.

***ee* determination:**

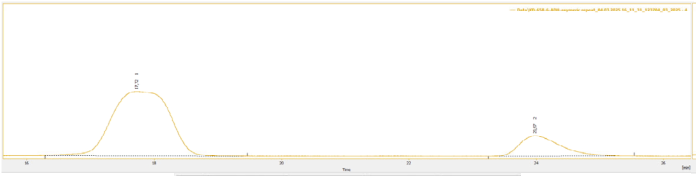

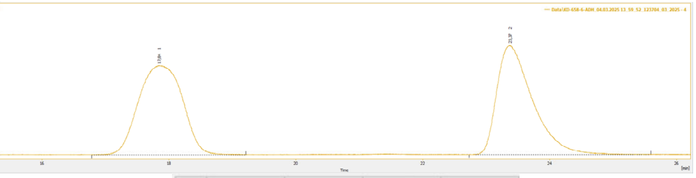

	Reten. Time [min]	Area [mAU.s]	Height [mAU]	Area [%]
1	17,716	62827,817	764,807	91,5
2	23,971	5826,931	87,601	8,5
	Total	68654,748	852,408	100,0

	Reten. Time [min]	Area [mAU.s]	Height [mAU]	Area [%]
1	17,839	8060,375	167,189	50,2
2	23,369	7986,304	204,706	49,8
	Total	16046,679	371,895	100,0

6. References

(1) Neufeld, E.; Pounder, A.; Tam, W. Rhodium-Catalyzed Ring-Opening Reactions of Heterobicyclic Alkenes with Heteroarene Nucleophiles. *Tetrahedron Lett.* **2023**, *127*, 154685. https://doi.org/10.1016/j.tetlet.2023.154685.

(2) Pounder, A.; Farkas, M.; Chen, L. D.; Tam, W. Iridium/Zinc-Co-Catalyzed Ring-Opening Reactions of Oxabicyclic Alkenes with Indole Nucleophiles: A Combined Experimental and Theoretical Study. *Organometallics* **2023**, *42* (9), 780–792. https://doi.org/10.1021/acs.organomet.3c00017.

(3) Kamzol, D.; Bahramiveleshkolaei, M.; Wilhelm, R. Camphor-Based NHC Ligands with a Sulfur Ligator Atom in Rhodium Catalysis: Catalytic Advances in the Asymmetric Ring Opening of *N* -Protected Azabenzonorbornenes. *Org. Lett.* **2025**, *27* (31), 8417–8422. https://doi.org/10.1021/acs.orglett.5c02110.

(4) Seki, M.; Yoshida, K. Chiral Bicyclic NHC/Rh Complexes and Their Application to Catalytic Asymmetric Ring-Opening Reaction of Oxabenzonorbornadienes with Amines. *J. Org. Chem.* **2022**, *87* (5), 3007–3013. https://doi.org/10.1021/acs.joc.1c02836.

(5) Zhou, Y.; Lu, Z.; Han, B.; Zeng, C.; Zhang, Z.; Fan, B. Iridium-Catalyzed Highly Enantioselective Ring Opening Reaction of Oxabenzonorbornadienes with Amines. *Tetrahedron Asymmetry* **2015**, *26* (23), 1354–1359. https://doi.org/10.1016/j.tetasy.2015.10.017.

(6) Bannwarth, C.; Caldeweyher, E.; Ehlert, S.; Hansen, A.; Pracht, P.; Seibert, J.; Spicher, S.; Grimme, S. Extended TIGHT-BINDING Quantum Chemistry Methods. *WIREs Comput. Mol. Sci.* **2021**, *11* (2), e1493. https://doi.org/10.1002/wcms.1493.

(7) Pracht, P.; Bohle, F.; Grimme, S. Automated Exploration of the Low-Energy Chemical Space with Fast Quantum Chemical Methods. *Phys. Chem. Chem. Phys.* **2020**, *22* (14), 7169–7192. https://doi.org/10.1039/C9CP06869D.

(8) Grimme, S. Exploration of Chemical Compound, Conformer, and Reaction Space with Meta-Dynamics Simulations Based on Tight-Binding Quantum Chemical Calculations. *J. Chem. Theory Comput.* **2019**, *15* (5), 2847–2862. https://doi.org/10.1021/acs.jctc.9b00143.

(9) Bohle, F.; Grimme, S. Hydrocarbon Macrocycle Conformer Ensembles and^{13} C-NMR Spectra. *Angew. Chem. Int. Ed.* **2022**, *61* (14), e202113905. https://doi.org/10.1002/anie.202113905.

(10) Grimme, S.; Bohle, F.; Hansen, A.; Pracht, P.; Spicher, S.; Stahn, M. Efficient Quantum Chemical Calculation of Structure Ensembles and Free Energies for Nonrigid Molecules. *J. Phys. Chem. A* **2021**, *125* (19), 4039–4054. https://doi.org/10.1021/acs.jpca.1c00971.

(11) Neese, F. The ORCA Program System. *WIREs Comput. Mol. Sci.* **2012**, *2* (1), 73–78. https://doi.org/10.1002/wcms.81.

(12) Neese, F. Software Update: The ORCA Program System, Version 4.0. *WIREs Comput. Mol. Sci.* **2018**, *8* (1), e1327. https://doi.org/10.1002/wcms.1327.

(13) Grimme, S.; Ehrlich, S.; Goerigk, L. Effect of the Damping Function in Dispersion Corrected Density Functional Theory. *J. Comput. Chem.* **2011**, *32* (7), 1456–1465. https://doi.org/10.1002/jcc.21759.

(14) Grimme, S.; Antony, J.; Ehrlich, S.; Krieg, H. A Consistent and Accurate *Ab Initio* Parametrization of Density Functional Dispersion Correction (DFT-D) for the 94 Elements H-Pu. *J. Chem. Phys.* **2010**, *132* (15), 154104. https://doi.org/10.1063/1.3382344.

(15) Weigend, F.; Ahlrichs, R. Balanced Basis Sets of Split Valence, Triple Zeta Valence and Quadruple Zeta Valence Quality for H to Rn: Design and Assessment of Accuracy. *Phys. Chem. Chem. Phys.* **2005**, *7* (18), 3297. https://doi.org/10.1039/b508541a.

(16) Neese, F. Software Update: The ORCA Program System—Version 6.0. *WIREs Comput. Mol. Sci.* **2025**, *15* (2). https://doi.org/10.1002/wcms.70019.

(17) Uzarewicz-Baig, M.; Koppenwallner, M.; Tabassum, S.; Wilhelm, R. Highly Regioselective Synthesis of Chiral Diamines via a Buchwald–Hartwig Amination from Camphoric Acid and Their Application in the Henry Reaction. *Appl. Organomet. Chem.* **2014**, *28* (7), 552–558. https://doi.org/10.1002/aoc.3162.

(18) Koppenwallner, M.; Rais, E.; Uzarewicz-Baig, M.; Tabassum, S.; Gilani, M.; Wilhelm, R. Synthesis of New Camphor-Based Carbene Ligands and Their Application in a Copper-Catalyzed Michael Addition with B2Pin2. *Synthesis* **2014**, *47* (06), 789–800. https://doi.org/10.1055/s-0034-1379877.

(19) Rais, E.; Flörke, U.; Wilhelm, R. Reactivity of Grubbs–Hoveyda II Complexes Including Extended N-Heterocyclic Carbenes with a Bicyclic Camphor-Based Framework. *Synthesis* **2017**, *49* (13), 2852–2864. https://doi.org/10.1055/s-0036-1588849.

(20) Espina, M.; Rivilla, I.; Conde, A.; Díaz-Requejo, M. M.; Pérez, P. J.; Álvarez, E.; Fernández, R.; Lassaletta, J. M. Chiral, Sterically Demanding N-Heterocyclic Carbenes Fused into a Heterobiaryl Skeleton: Design, Synthesis, and Structural Analysis. *Organometallics* **2015**, *34* (7), 1328–1338. https://doi.org/10.1021/acs.organomet.5b00046.

(21) Seiders, T. J.; Ward, D. W.; Grubbs, R. H. Enantioselective Ruthenium-Catalyzed Ring-Closing Metathesis. *Org. Lett.* **2001**, *3* (20), 3225–3228. https://doi.org/10.1021/ol0165692.

(22) Wolf, S.; Plenio, H. Synthesis of (NHC)Rh(Cod)Cl and (NHC)RhCl(CO)2 Complexes – Translation of the Rh- into the Ir-Scale for the Electronic Properties of NHC Ligands. *J. Organomet. Chem.* **2009**, *694* (9–10), 1487–1492. https://doi.org/10.1016/j.jorganchem.2008.12.047.

(23) Pandey, G.; Varkhedkar, R.; Tiwari, D. Efficient Access to Enantiopure 1,3-Disubstituted Isoindolines from Selective Catalytic Fragmentation of an Original Desymmetrized Rigid Overbred Template. *Org. Biomol. Chem.* **2015**, *13* (15), 4438–4448. https://doi.org/10.1039/C5OB00229J.

(24) Banerjee, S.; Vivek Kumar, S.; Punniyamurthy, T. Site-Selective Rh-Catalyzed C-7 and C-6 Dual C–H Functionalization of Indolines: Synthesis of Functionalized Pyrrolocarbazoles. *J. Org. Chem.* **2020**, *85* (4), 2793–2805. https://doi.org/10.1021/acs.joc.9b03180.

(25) Qiu, S.; Zhai, S.; Wang, H.; Chen, X.; Zhai, H. One-Pot Synthesis of Benzo[*b*]Fluorenones *via* a Cobalt-Catalyzed MHP-Directed [3+2] Annulation/Ring-Opening/Dehydration Sequence. *Chem. Commun.* **2019**, *55* (29), 4206–4209. https://doi.org/10.1039/C9CC00948E.

(26) Caster, K. C.; Keck, C. G.; Walls, R. D. Synthesis of Benzonorbornadienes: Regioselective Benzyne Formation. *J. Org. Chem.* **2001**, *66* (9), 2932–2936. https://doi.org/10.1021/jo001277k.

(27) Yang, D.; Hu, P.; Long, Y.; Wu, Y.; Zeng, H.; Wang, H.; Zuo, X. Iridium-Catalyzed Asymmetric Ring-Opening Reactions of Oxabicyclic Alkenes with Secondary Amine Nucleophiles. *Beilstein J. Org. Chem.* **2009**, *5*. https://doi.org/10.3762/bjoc.5.53.

(28) Lautens, M.; Fagnou, K.; Rovis, T. Rhodium-Catalyzed Asymmetric Alcoholysis and Aminolysis of Oxabenzonorbornadiene: A New Enantioselective Carbon−Heteroatom Bond Forming Process. *J. Am. Chem. Soc.* **2000**, *122* (23), 5650–5651. https://doi.org/10.1021/ja000134c.

(29) Gao, P.; Foster, D.; Sipos, G.; Skelton, B. W.; Sobolev, A. N.; Dorta, R. Chiral NHC-Iridium Complexes and Their Performance in Enantioselective Intramolecular Hydroamination and Ring-Opening Amination Reactions. *Organometallics* **2020**, *39* (4), 556–573. https://doi.org/10.1021/acs.organomet.9b00770.

(30) Yu, L.; Zhou, Y.; Xu, X.; Li, S.; Xu, J.; Fan, B.; Lin, C.; Bian, Z.; Chan, A. S. C. Asymmetric Ring Opening Reaction of Oxabenzonorbornadienes with Amines Promoted by Iridium/NMDPP Complex. *Tetrahedron Lett.* **2014**, *55* (46), 6315–6318. https://doi.org/10.1016/j.tetlet.2014.09.089.

(31) Meng, L.; Yang, W.; Pan, X.; Tao, M.; Cheng, G.; Wang, S.; Zeng, H.; Long, Y.; Yang, D. Platinum-Catalyzed Asymmetric Ring-Opening Reactions of Oxabenzonorbornadienes with Phenols. *J. Org. Chem.* **2015**, *80* (5), 2503–2512. https://doi.org/10.1021/acs.joc.5b00065.

(32) Lautens, M.; Fagnou, K.; Taylor, M.; Rovis, T. Rhodium-Catalysed Asymmetric Ring Opening of Oxabicyclic Alkenes with Heteroatom Nucleophiles. *J. Organomet. Chem.* **2001**, *624* (1–2), 259–270. https://doi.org/10.1016/S0022-328X(00)00904-9.

7. Copy of NMR data Analysis

^{1}H NMR (600 MHz, $CDCl_3$), ^{13}C{^{1}H} NMR (151 MHz, $CDCl_3$) and ^{15}N HSQC NMR (61 MHz, $CDCl_3$) Analysis of Compound **2f**

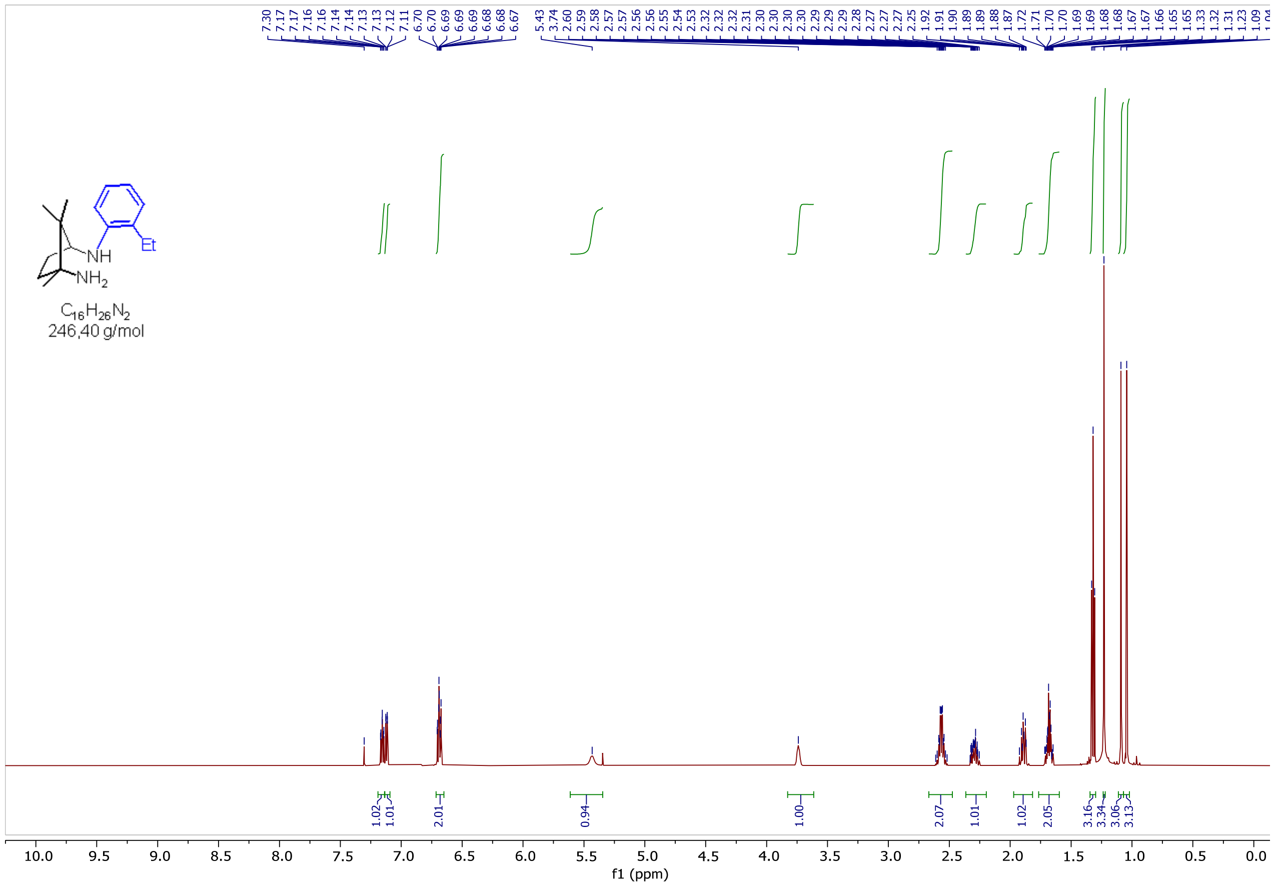

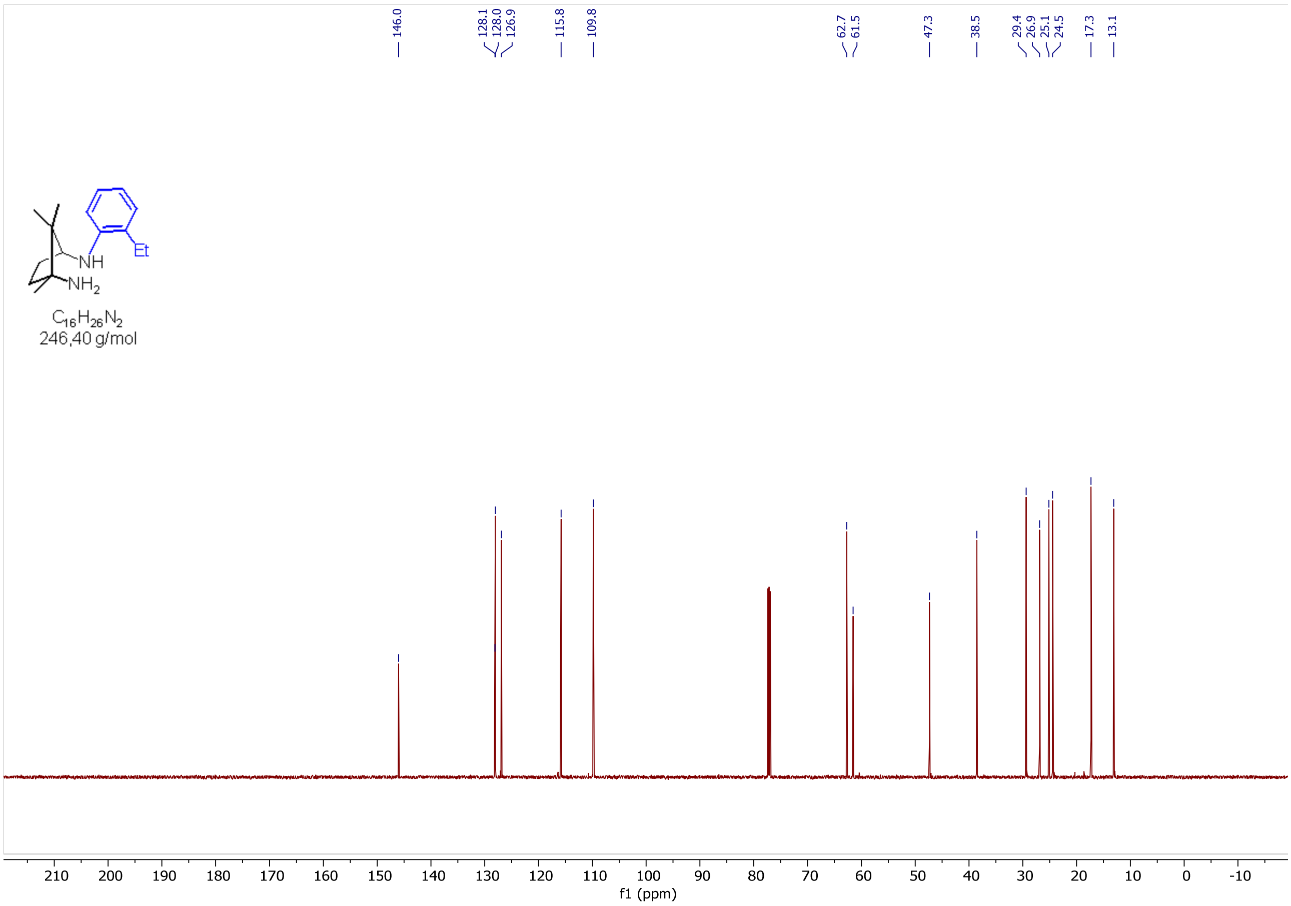

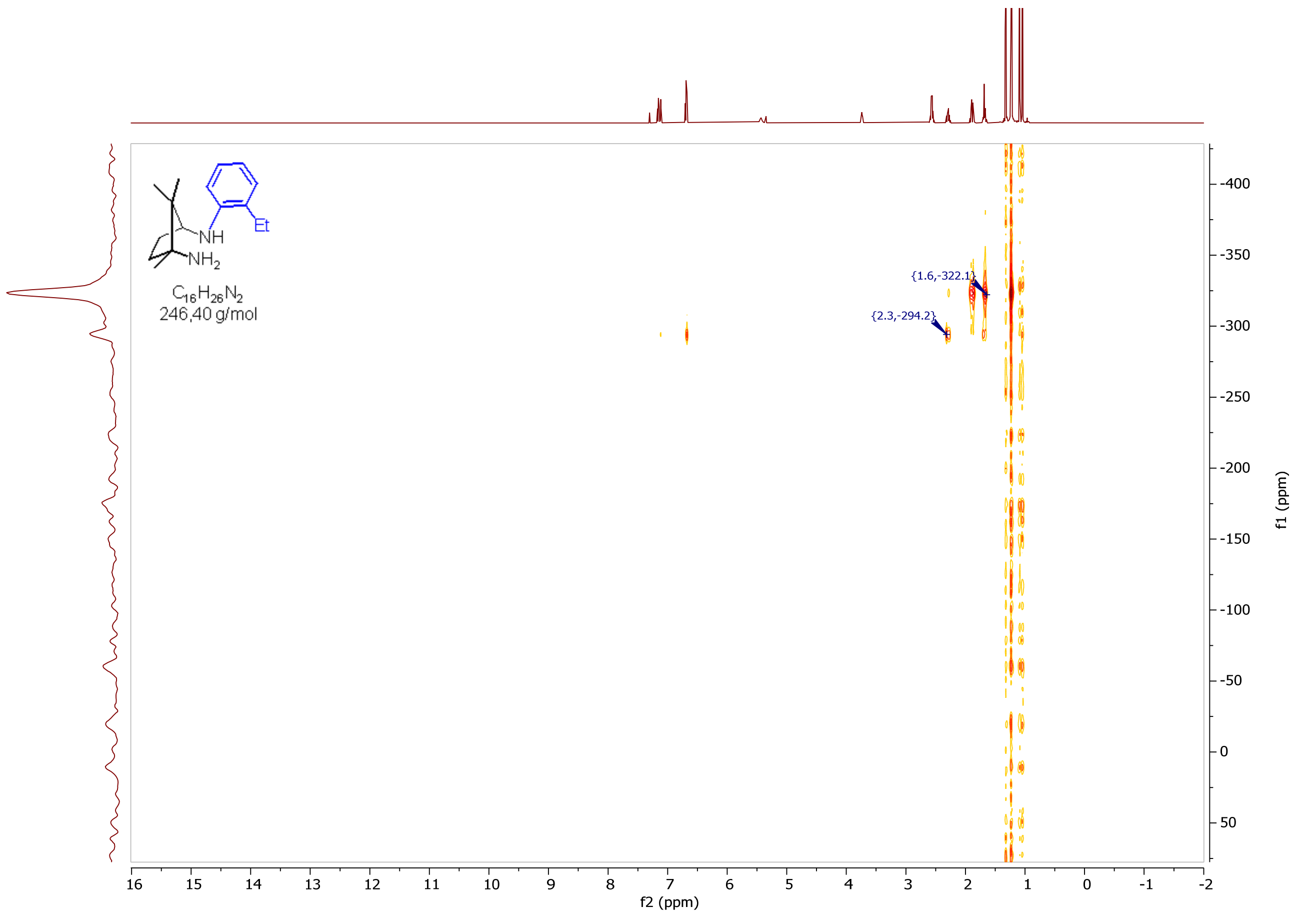
NH
NH2
Et
C16H26N2
246,40 g/mol
{1.6,-322.1}
{2.3,-294.2}
f2 (ppm)
f1 (ppm)

^{1}H NMR (600 MHz, CDCl3), ^{13}C{^{1}H} NMR (151 MHz, CDCl3), ^{15}N HSQC NMR (61 MHz, CDCl3) and ^{19}F NMR (337 MHz, CDCl3) Analysis of Compound **2h**

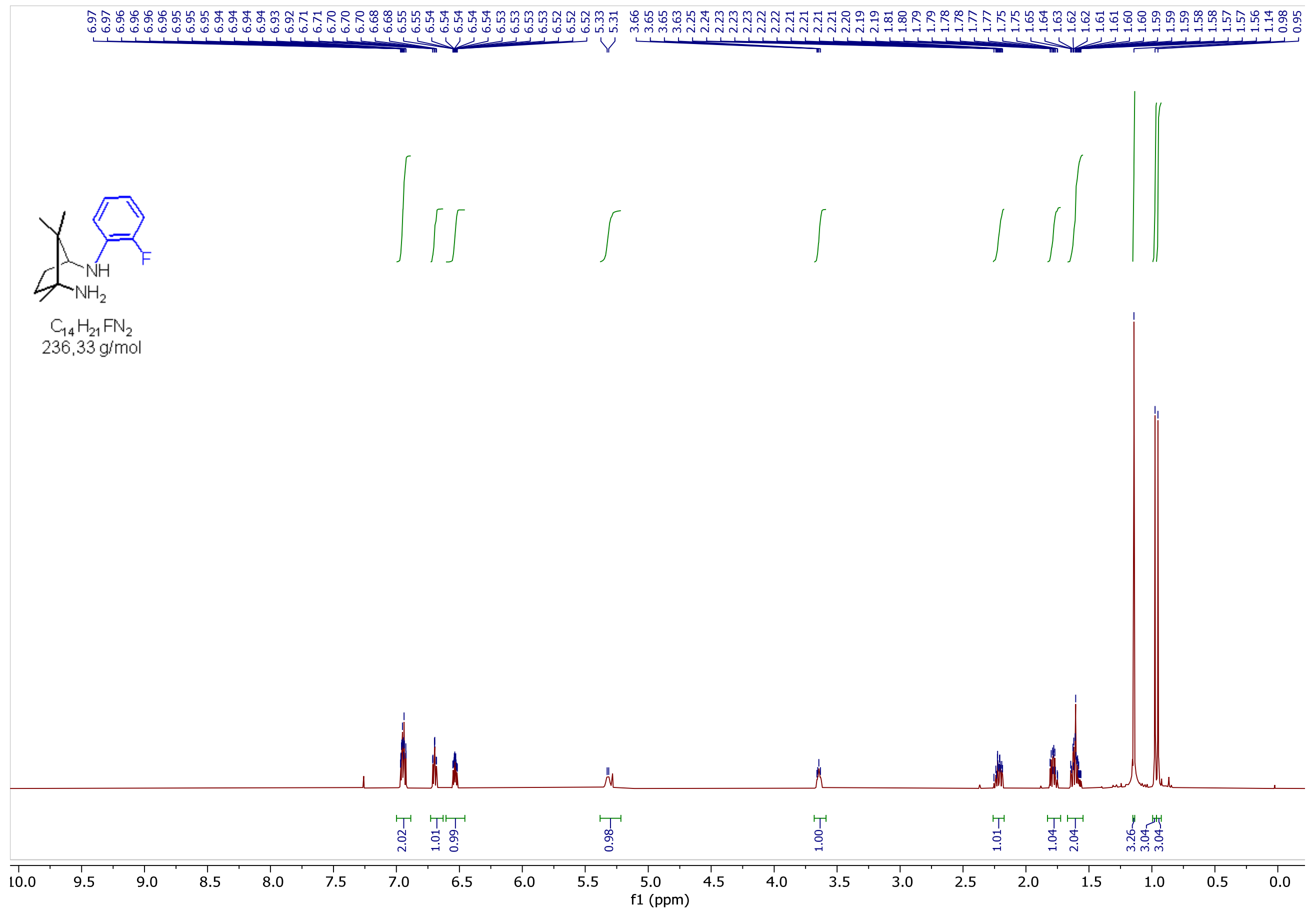

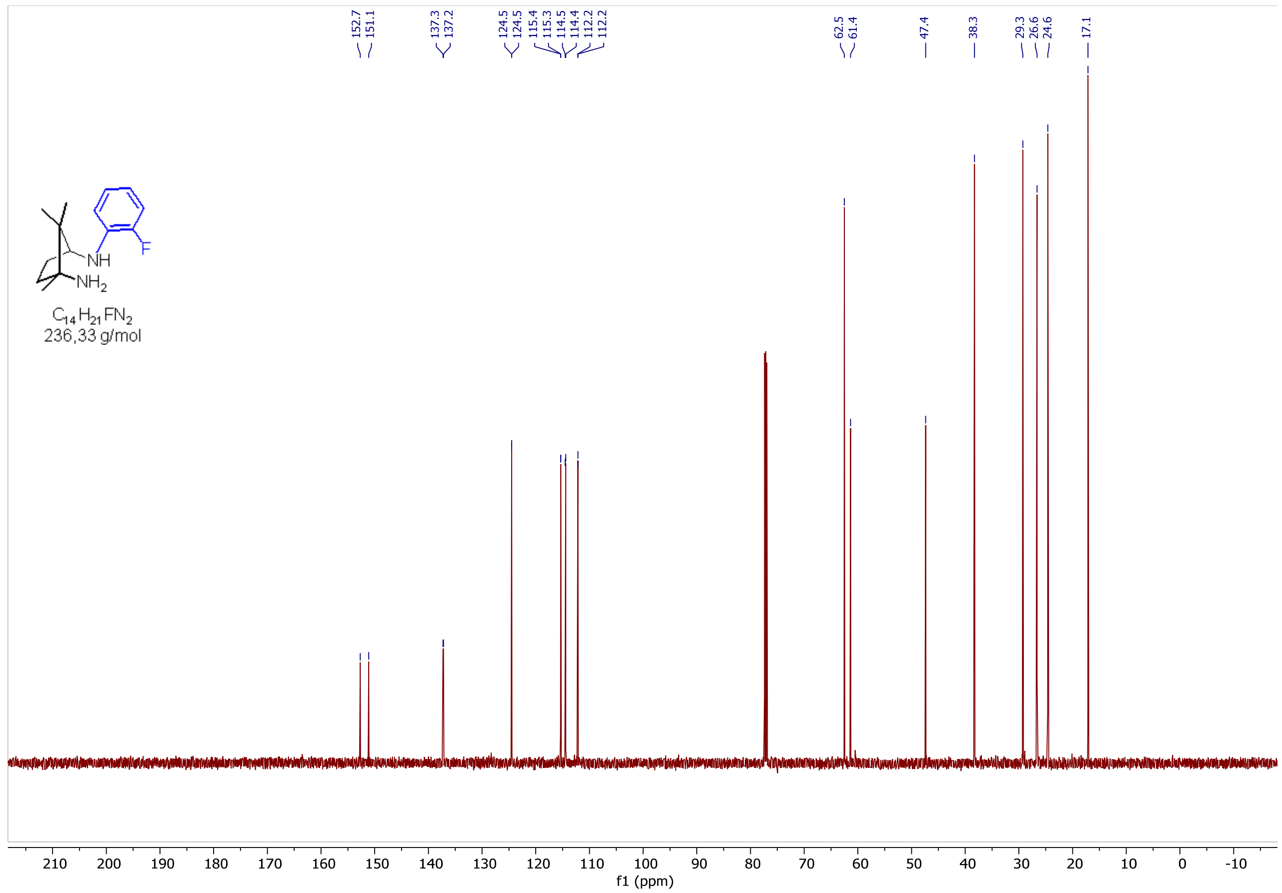

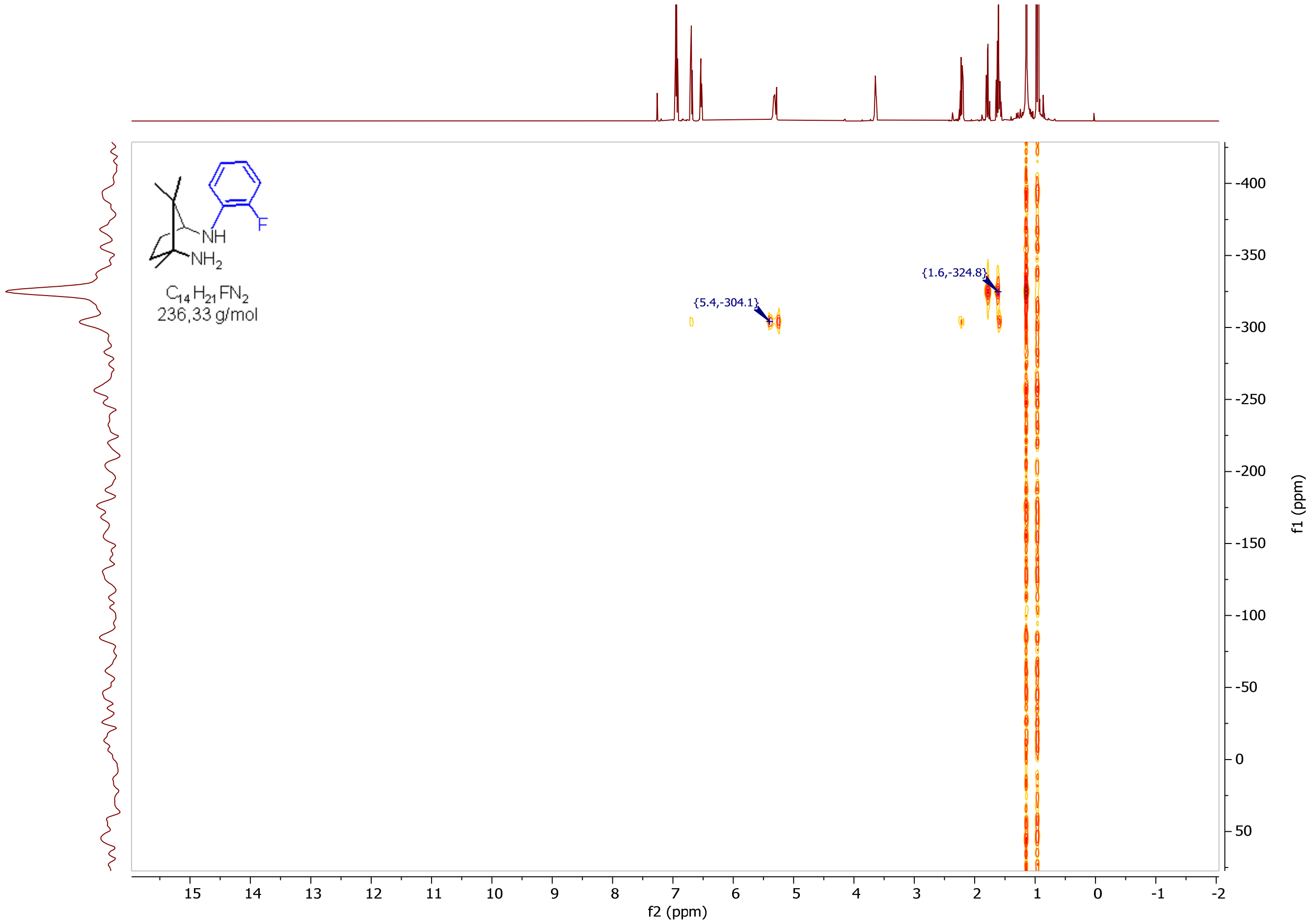

NH
NH2
F
C14H21FN2
236,33 g/mol
{5.4,-304.1}
{1.6,-324.8}
f2 (ppm)
f1 (ppm)

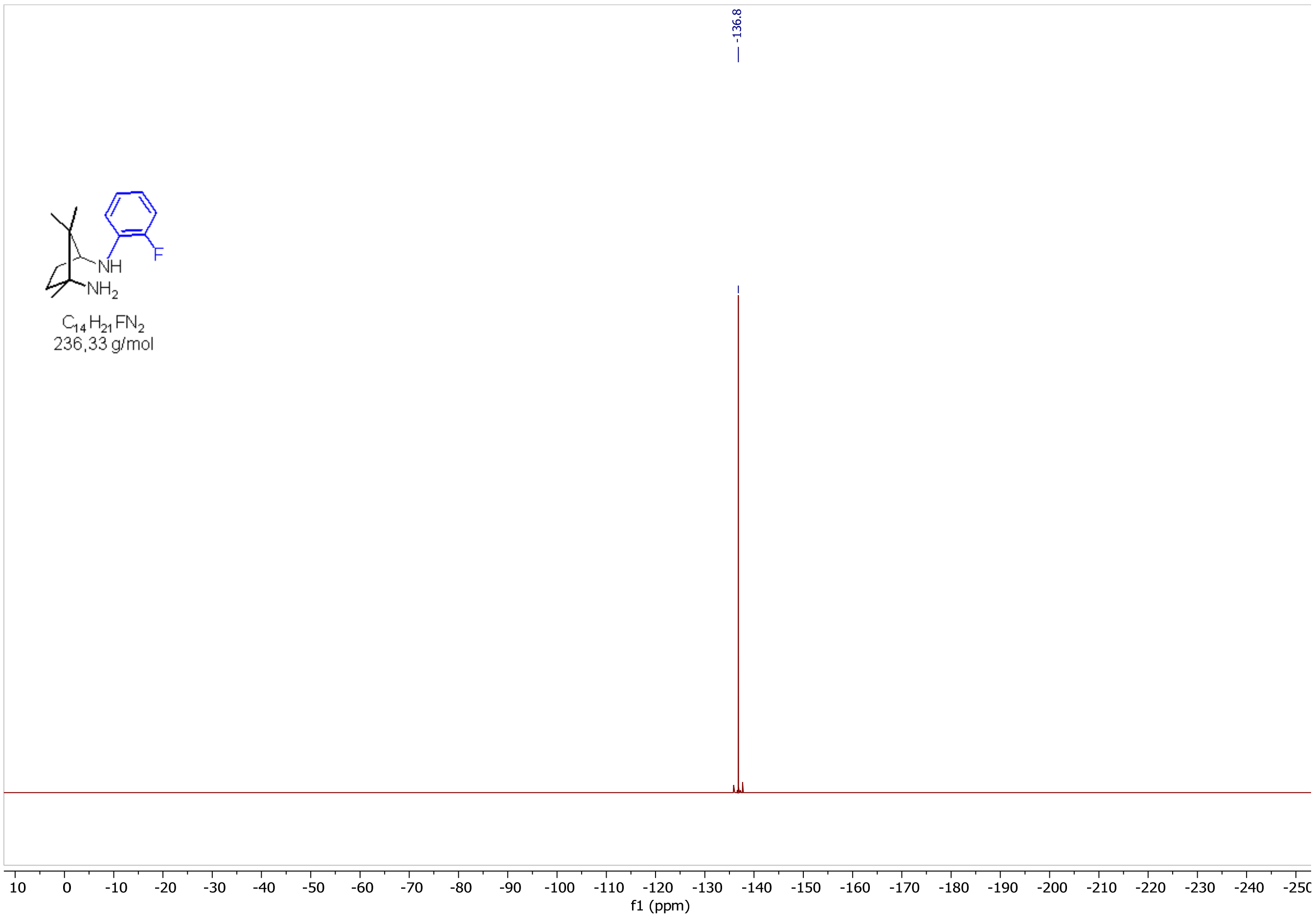

-136.8
NH
NH2
F
C14H21FN2
236,33 g/mol
f1 (ppm)

^{1}H NMR (600 MHz, $CDCl_3$), $^{13}C\{^{1}H\}$ NMR (151 MHz, $CDCl_3$), ^{15}N HSQC NMR (61 MHz, $CDCl_3$) and ^{19}F NMR (337 MHz, $CDCl_3$) Analysis of Compound **2i**

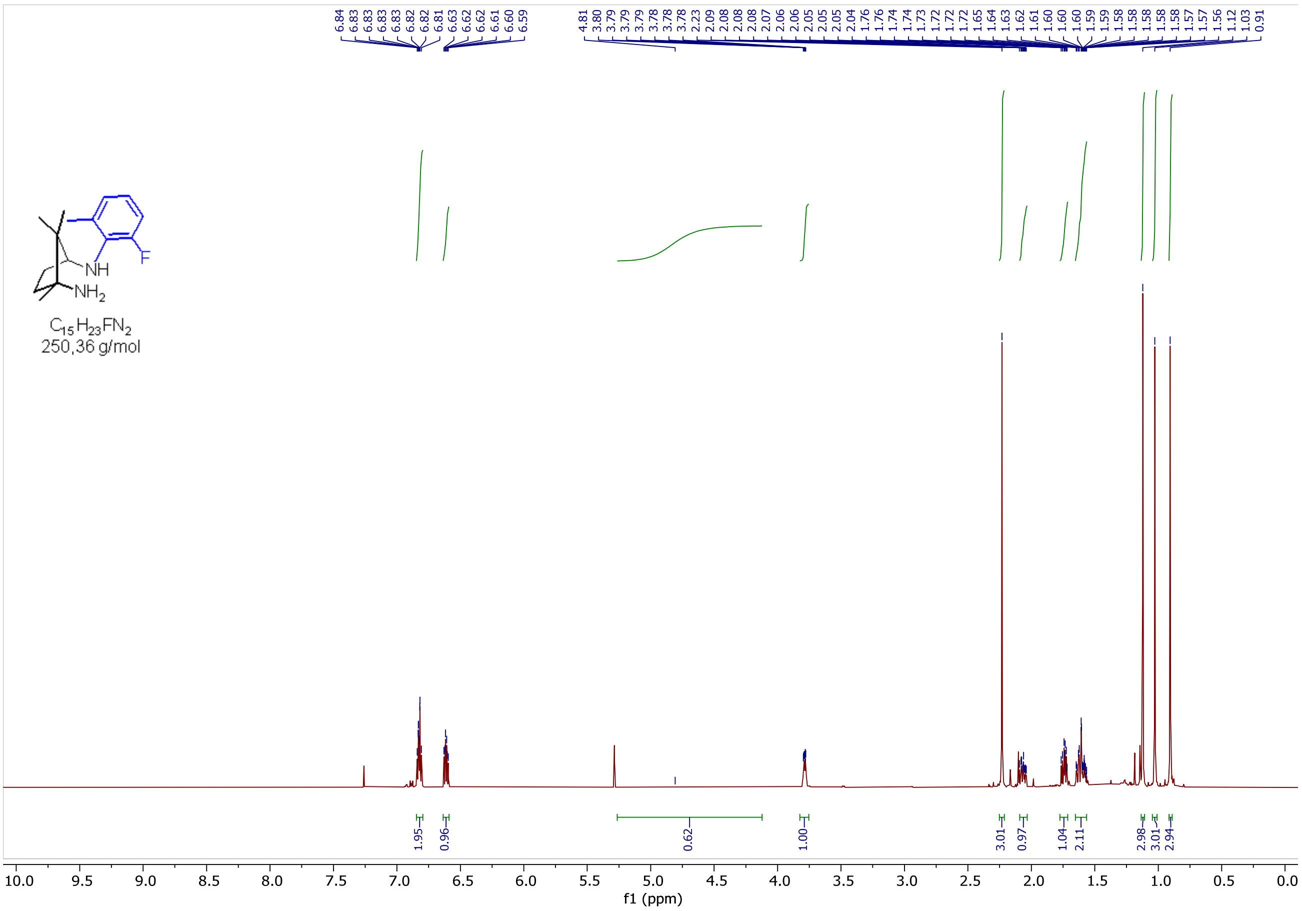

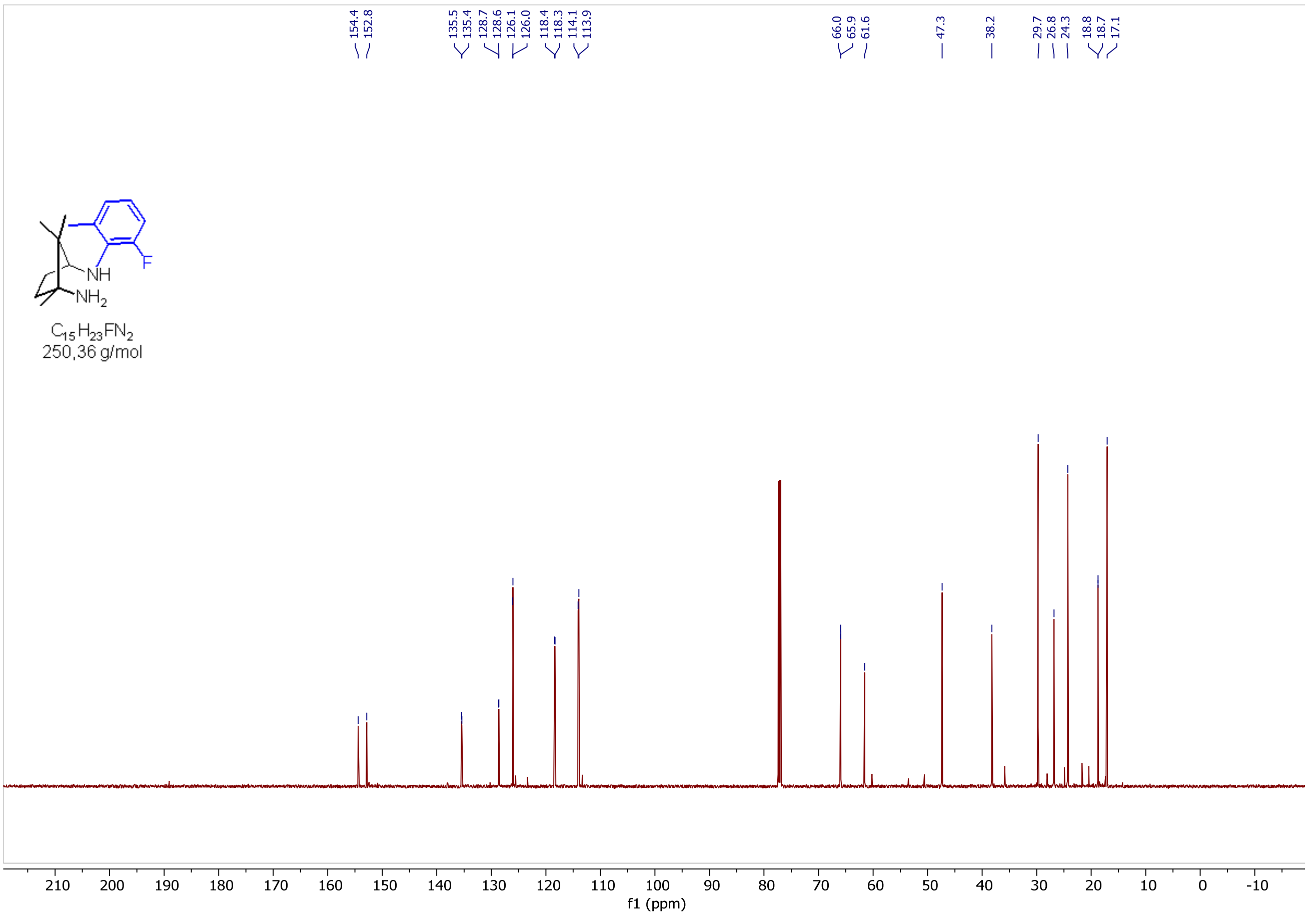

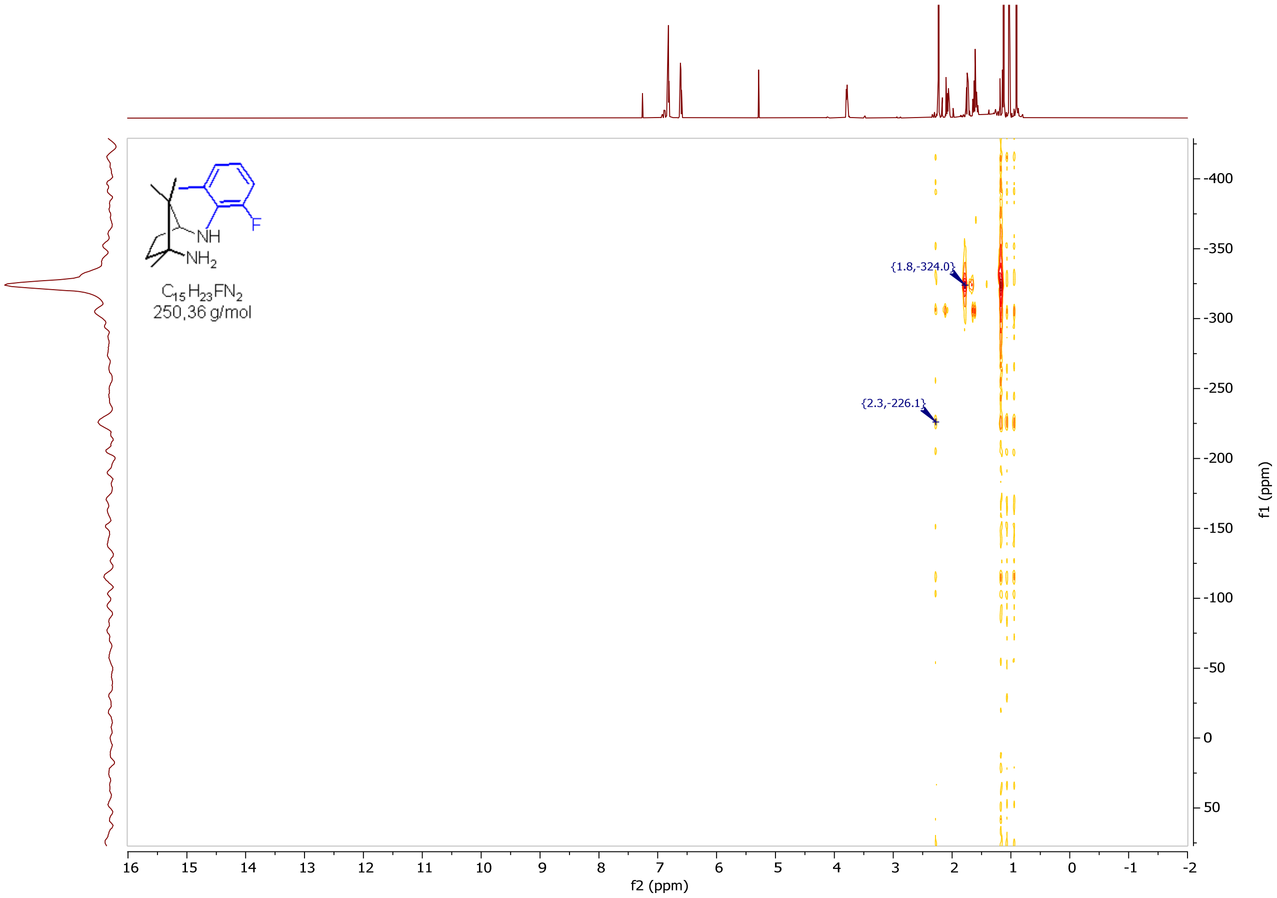
NH
NH2
F
C15H23FN2
250,36 g/mol
{1.8,-324.0}
{2.3,-226.1}
f2 (ppm)
f1 (ppm)

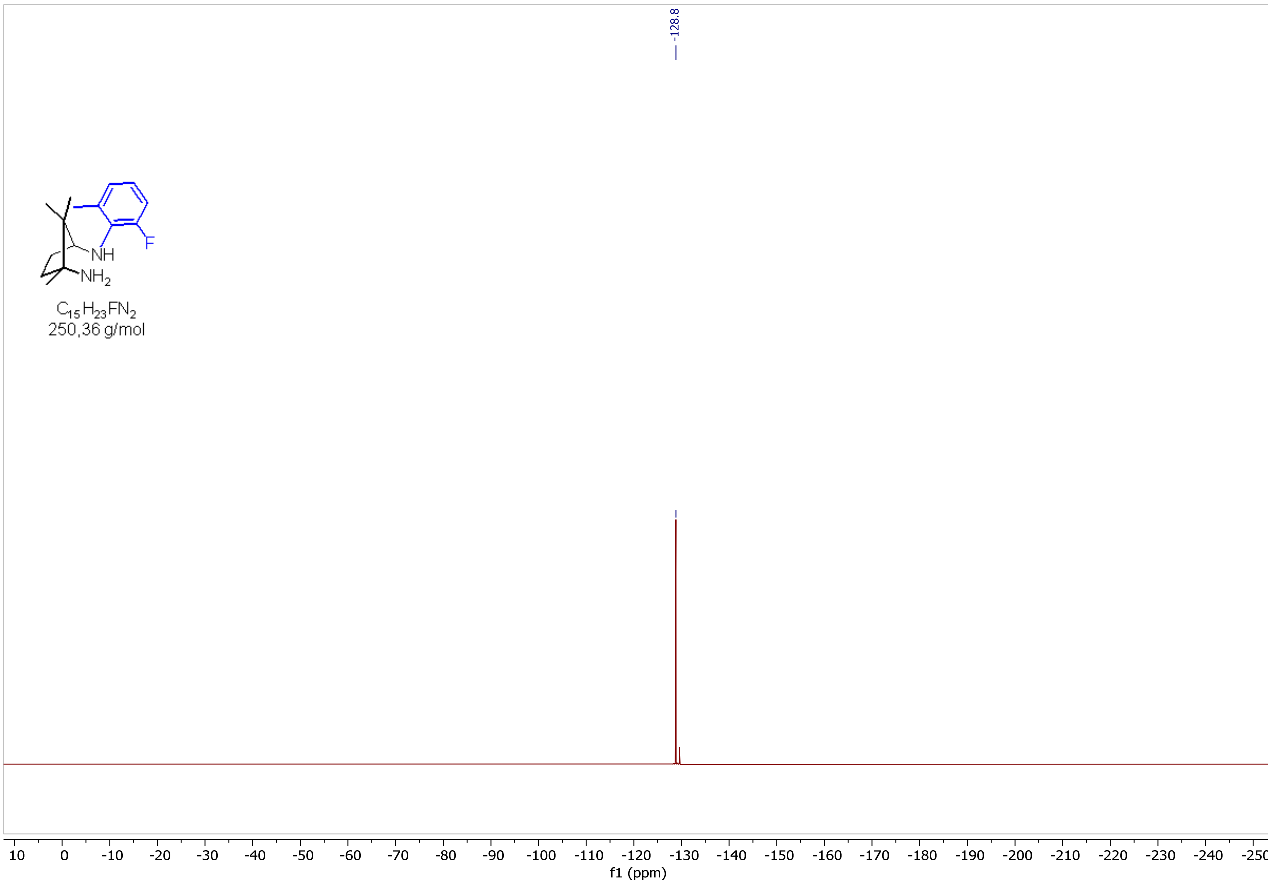
-128.8
NH
NH2
F
C15H23FN2
250,36 g/mol
f1 (ppm)

^{1}H NMR (600 MHz, $CDCl_3$), ^{13}C{^{1}H} NMR (151 MHz, $CDCl_3$) and ^{15}N HSQC NMR (61 MHz, $CDCl_3$) Analysis of Compound **2j**

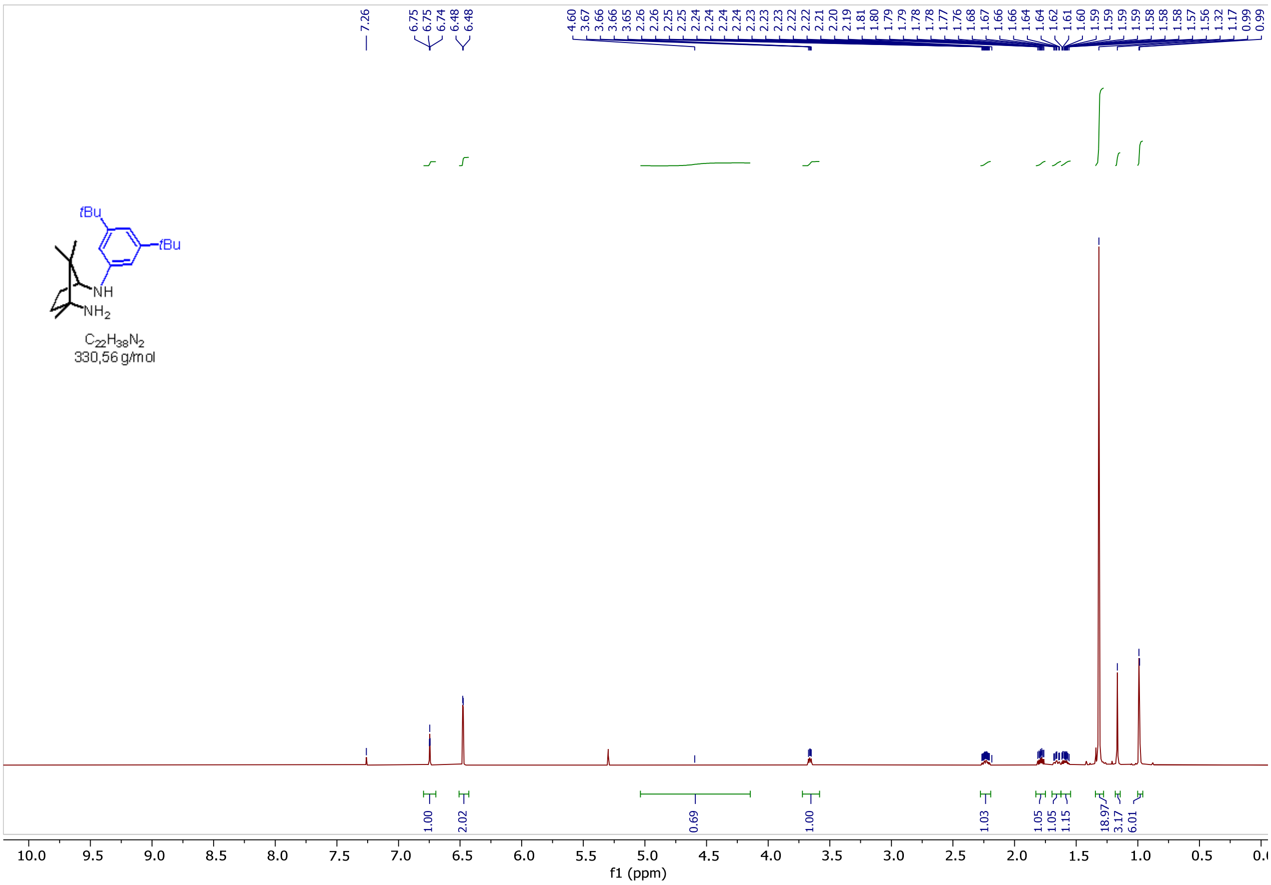

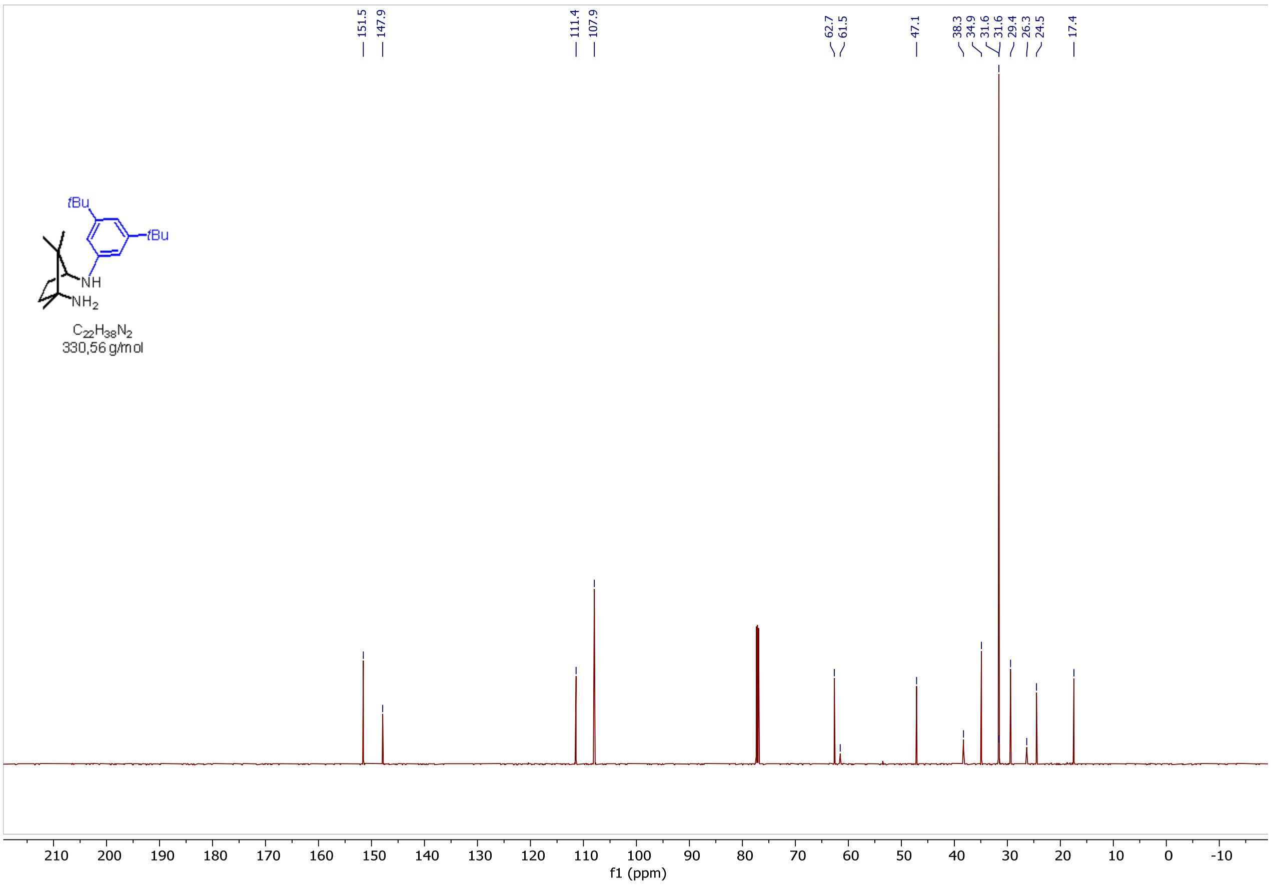

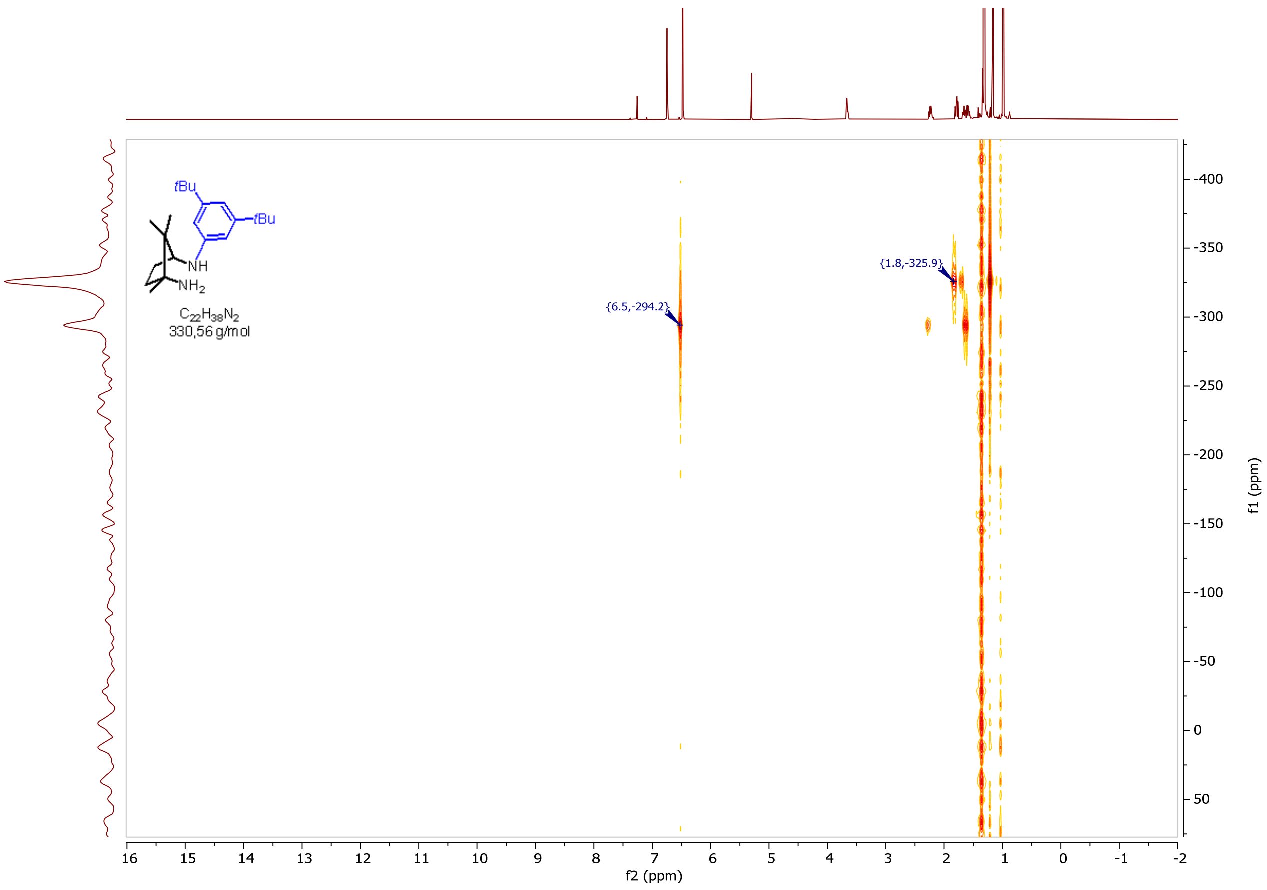
tBu
tBu
NH
NH2
C22H38N2
330,56 g/mol
{6.5,-294.2}
{1.8,-325.9}
f2 (ppm)
f1 (ppm)

^{1}H NMR (600 MHz, $CDCl_3$), ^{13}C{^{1}H} NMR (151 MHz, $CDCl_3$), ^{15}N HSQC NMR (61 MHz, $CDCl_3$) and ^{19}F NMR (337 MHz, $CDCl_3$) Analysis of Compound **2k**

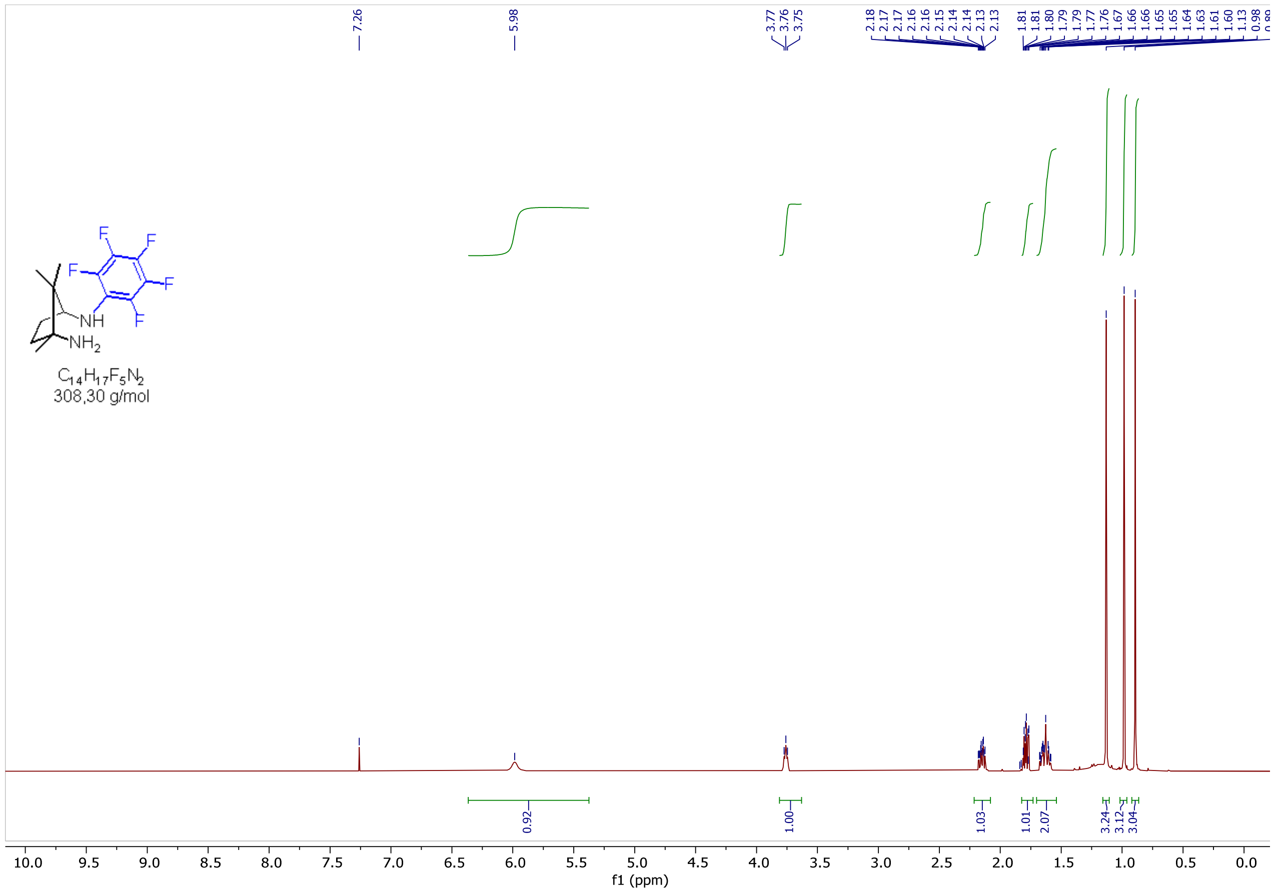

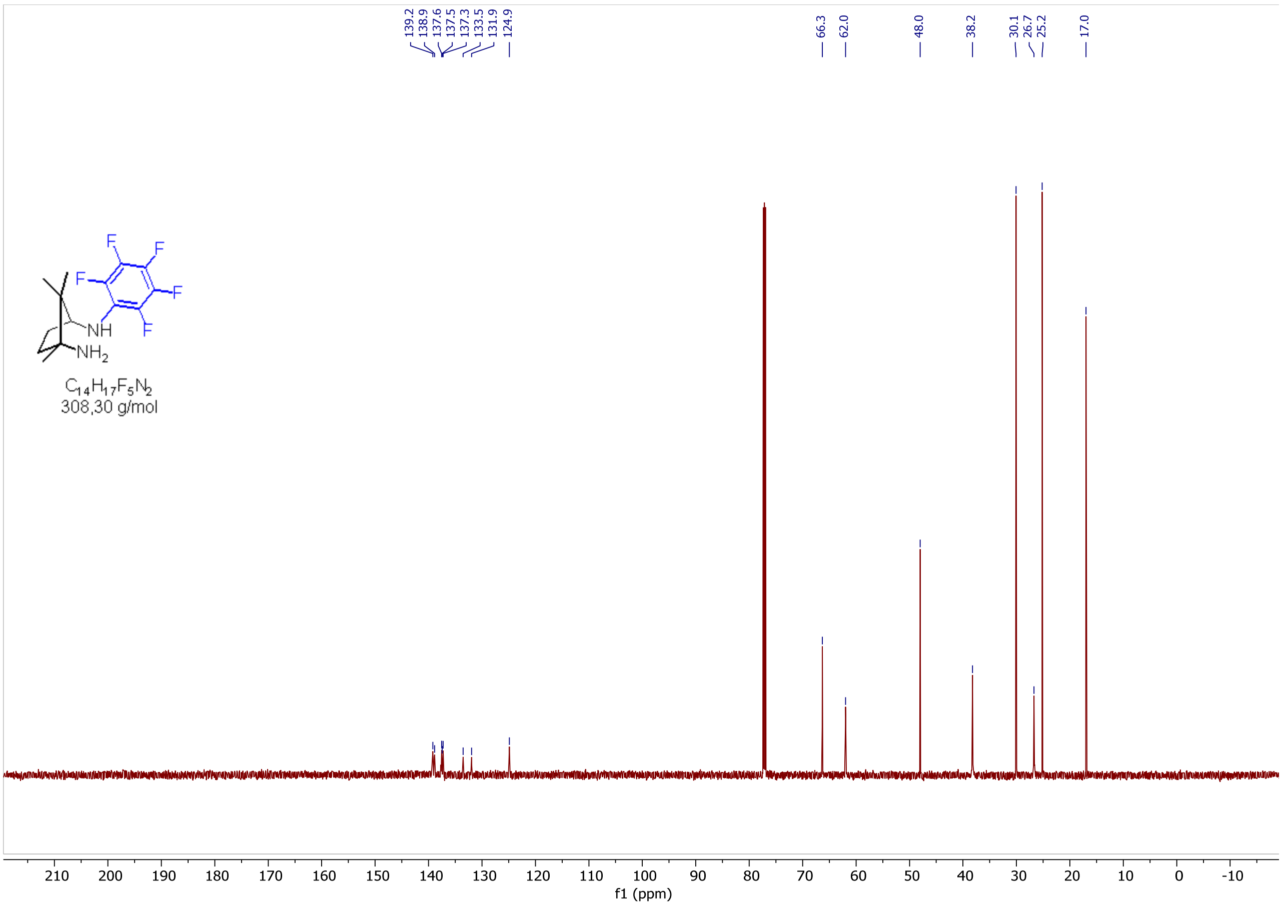

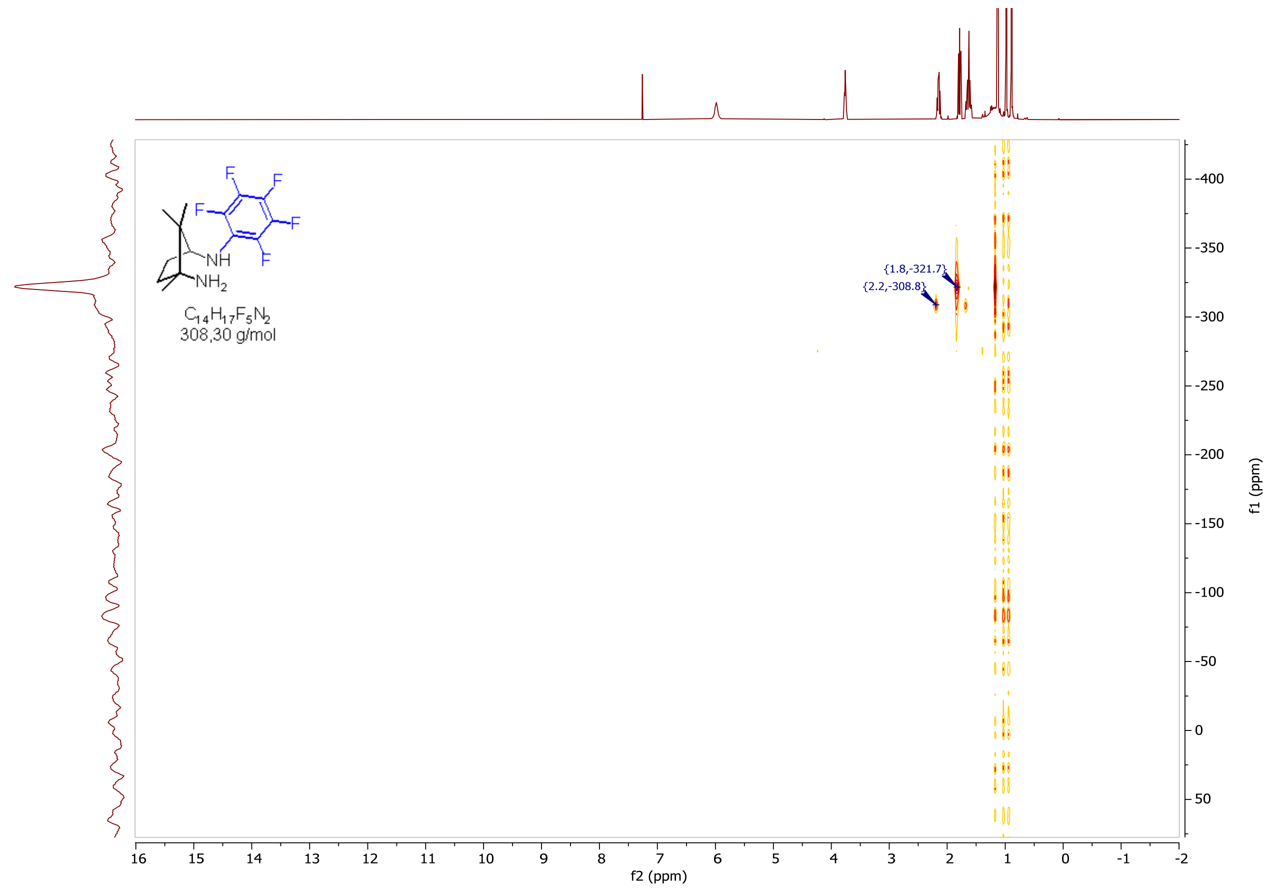

C14H17F5N2
308,30 g/mol
{1.8,-321.7}
{2.2,-308.8}
f2 (ppm)
f1 (ppm)

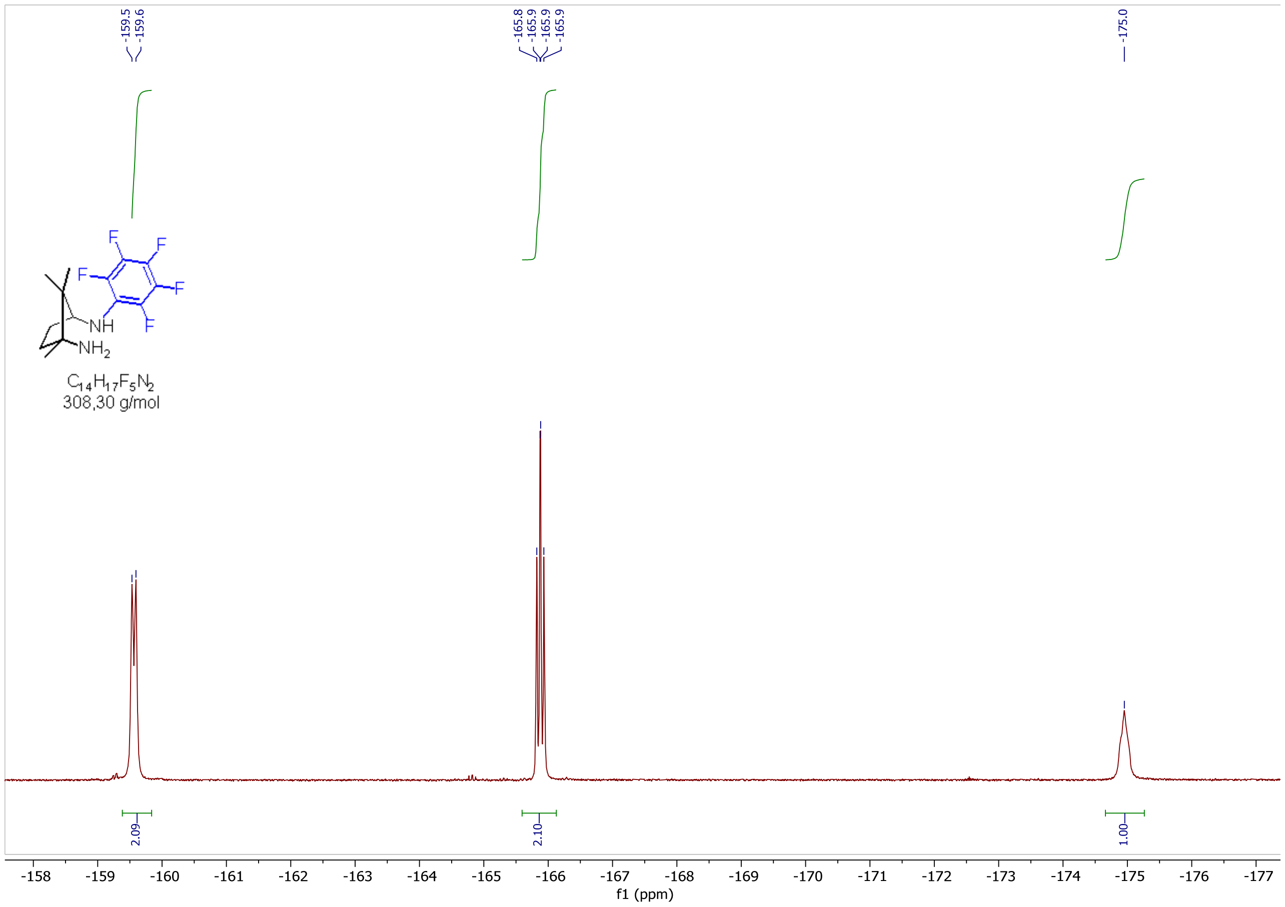

-159.5
-159.6
-165.8
-165.9
-165.9
-165.9
-175.0
C14H17F5N2
308,30 g/mol
2.09
2.10
1.00
f1 (ppm)

^{1}H NMR (600 MHz, $CDCl_3$), ^{13}C{^{1}H} NMR (151 MHz, $CDCl_3$) and ^{15}N HSQC NMR (61 MHz, $CDCl_3$) Analysis of Compound **2n**

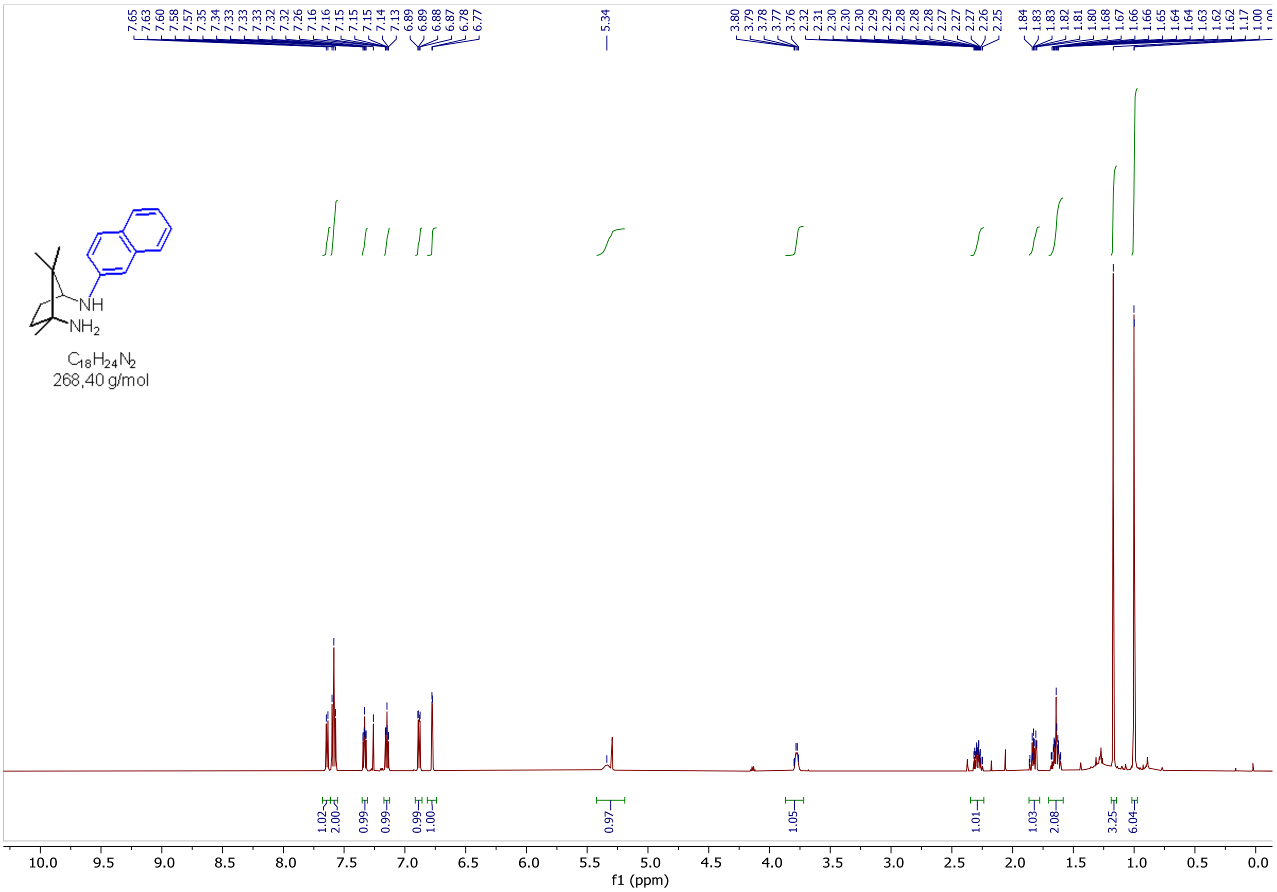

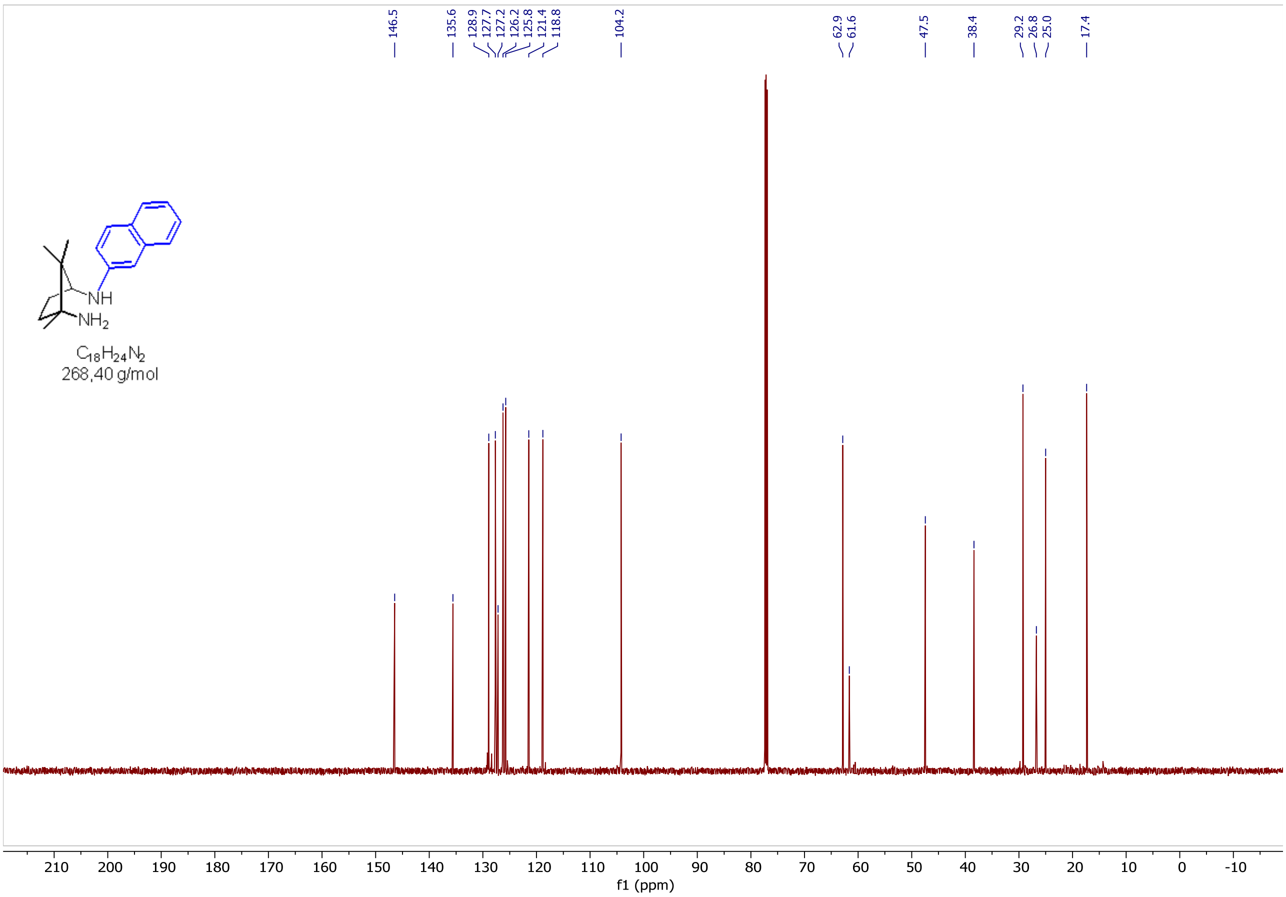

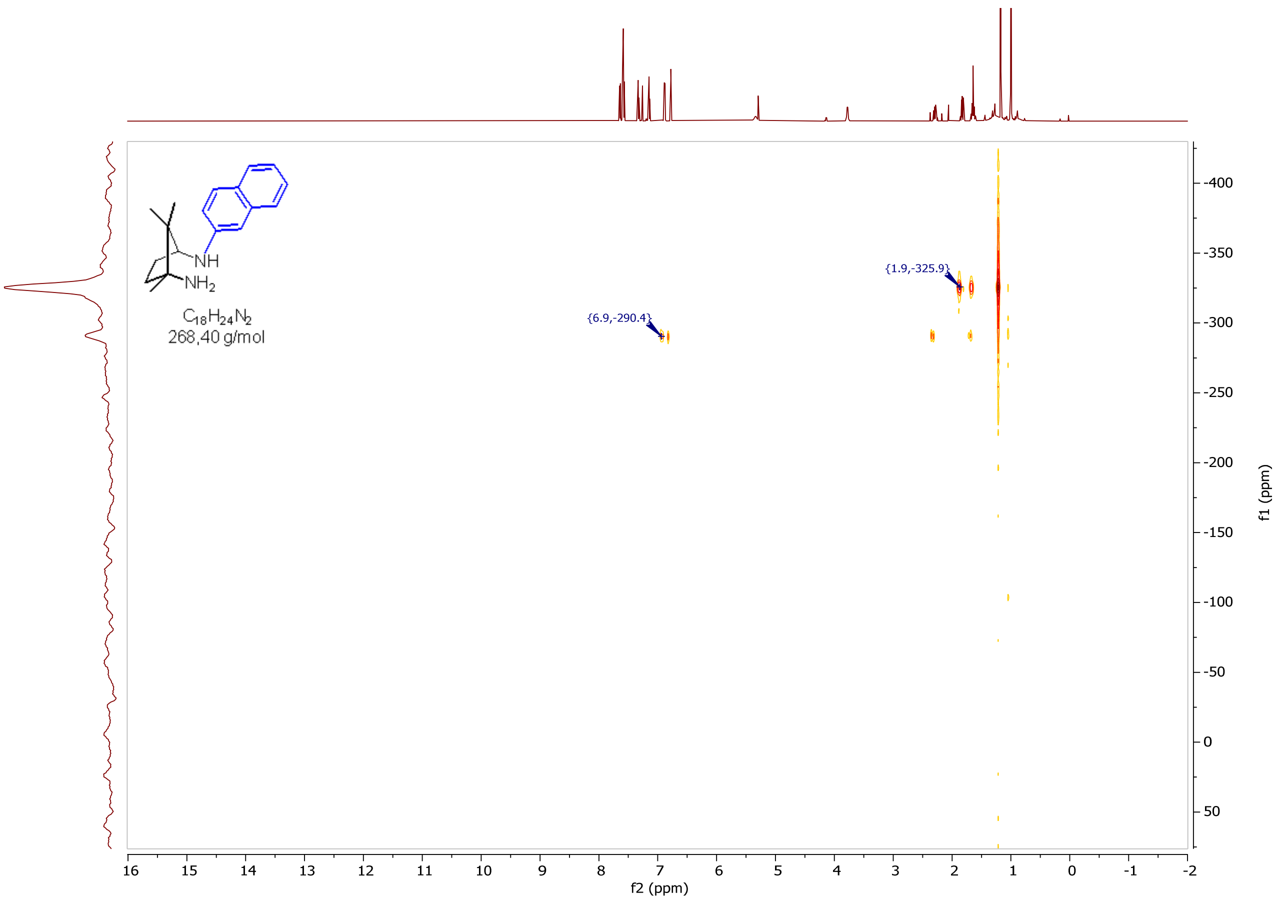

$C_{18}H_{24}N_2$
268,40 g/mol
NH
NH_2
{1.9,-325.9}
{6.9,-290.4}
f2 (ppm)
f1 (ppm)

^{1}H NMR (600 MHz, $CDCl_3$), ^{13}C{^{1}H} NMR (151 MHz, $CDCl_3$) and ^{15}N HSQC NMR (61 MHz, $CDCl_3$) Analysis of Compound **3ad**

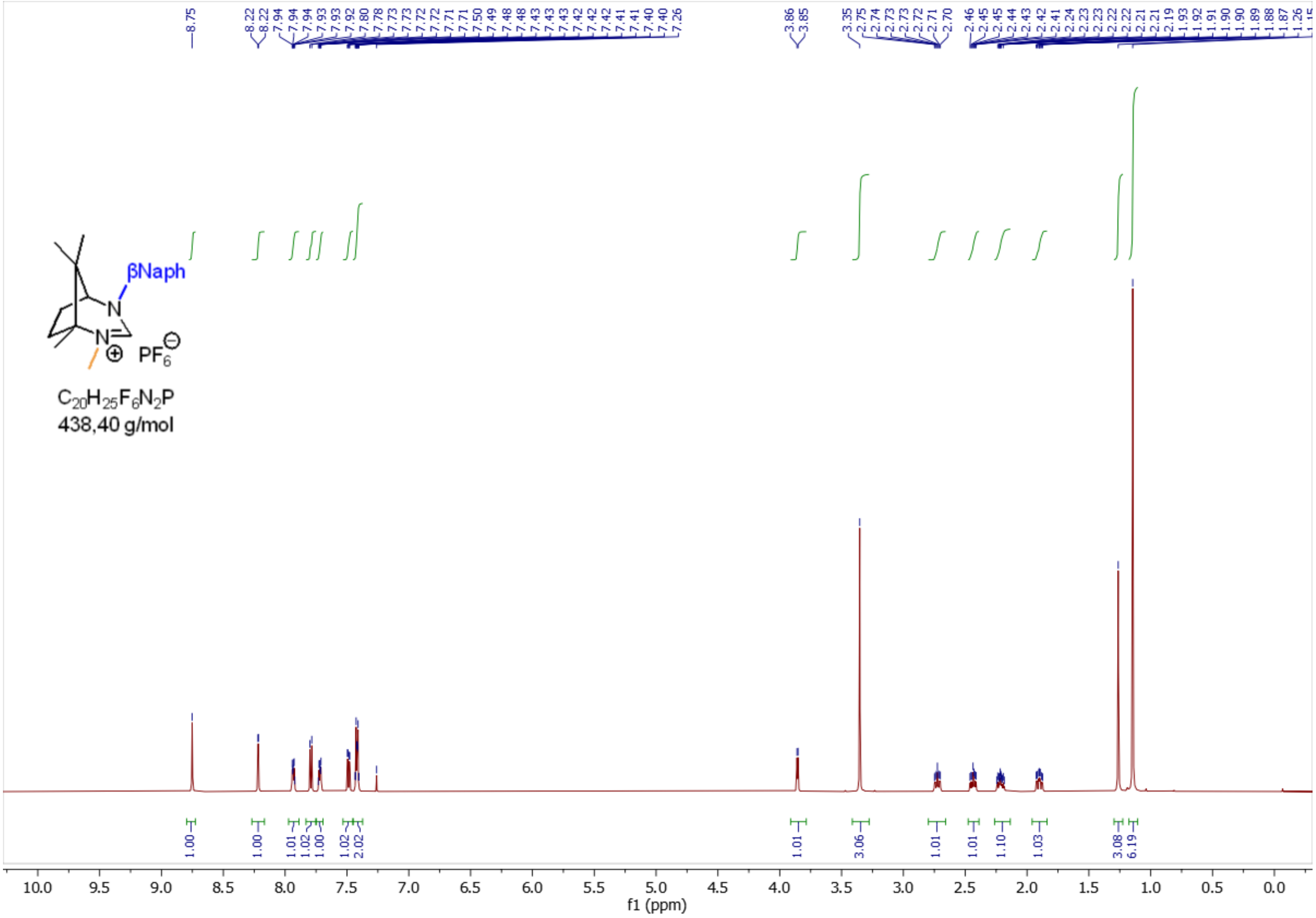

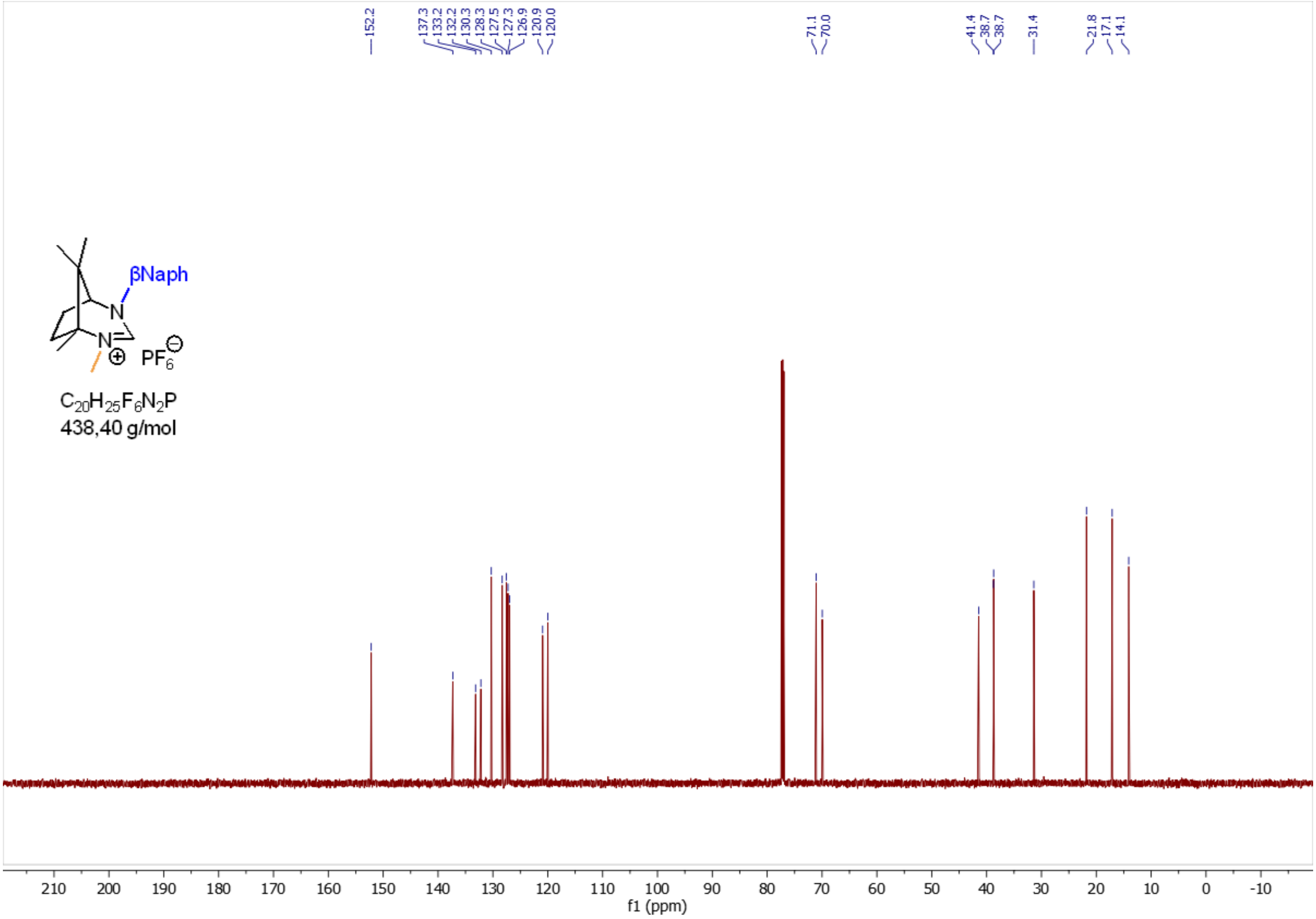

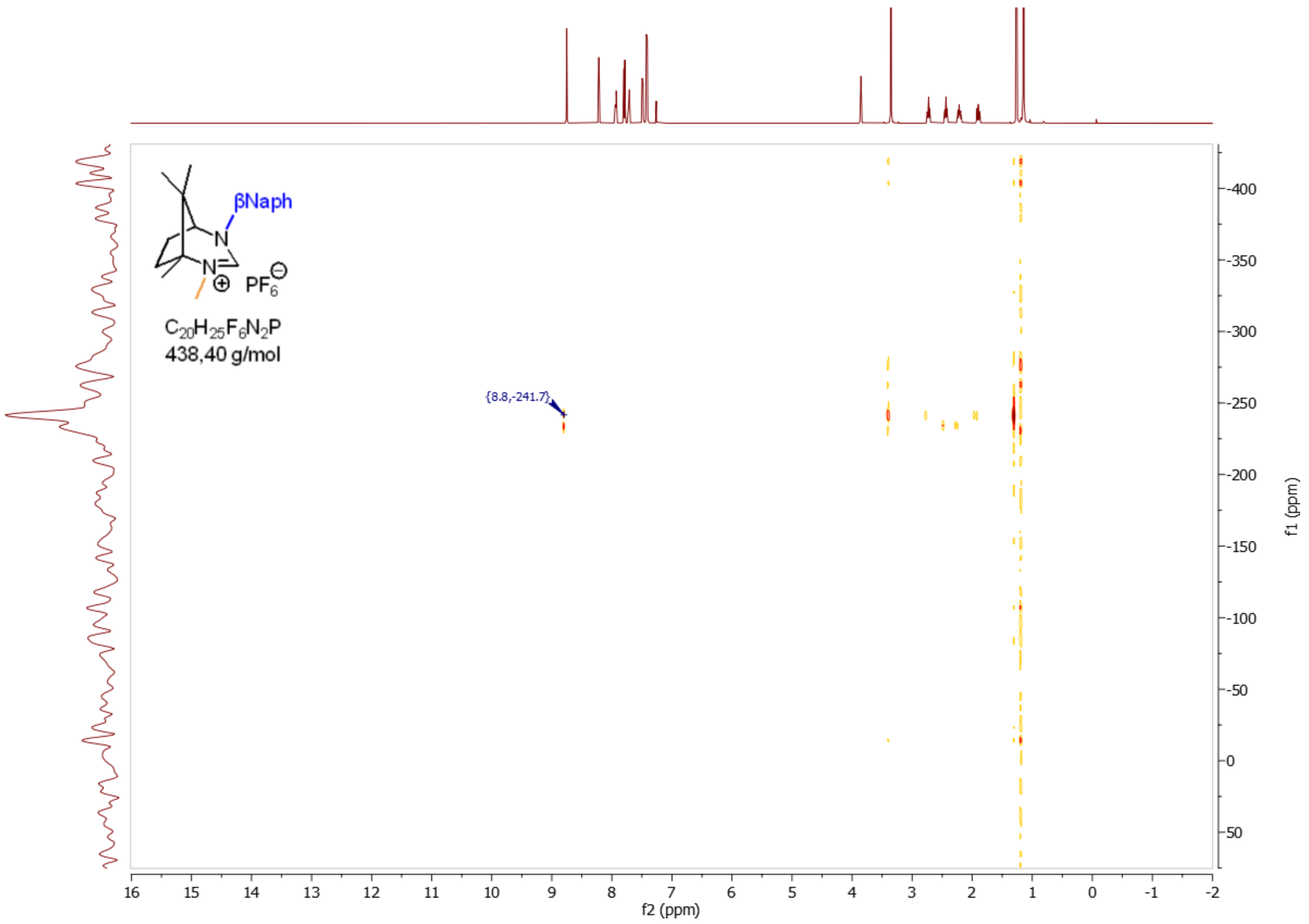
βNaph
PF6
C20H25F6N2P
438,40 g/mol
{8.8,-241.7}
f2 (ppm)
f1 (ppm)

^{1}H NMR (600 MHz, $CDCl_3$), ^{13}C{^{1}H} NMR (151 MHz, $CDCl_3$) and ^{15}N HSQC NMR (61 MHz, $CDCl_3$) Analysis of Compound **3af**

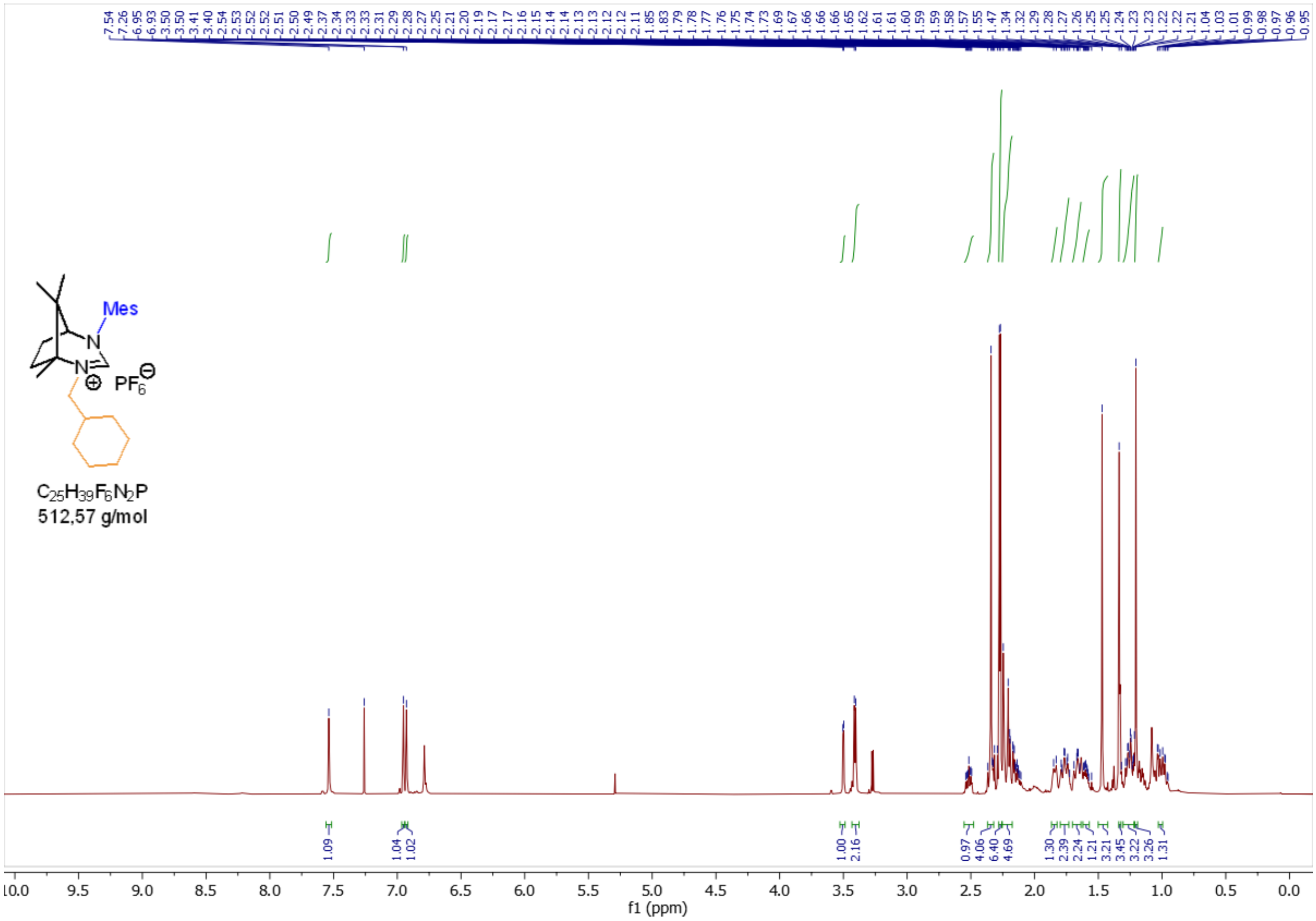

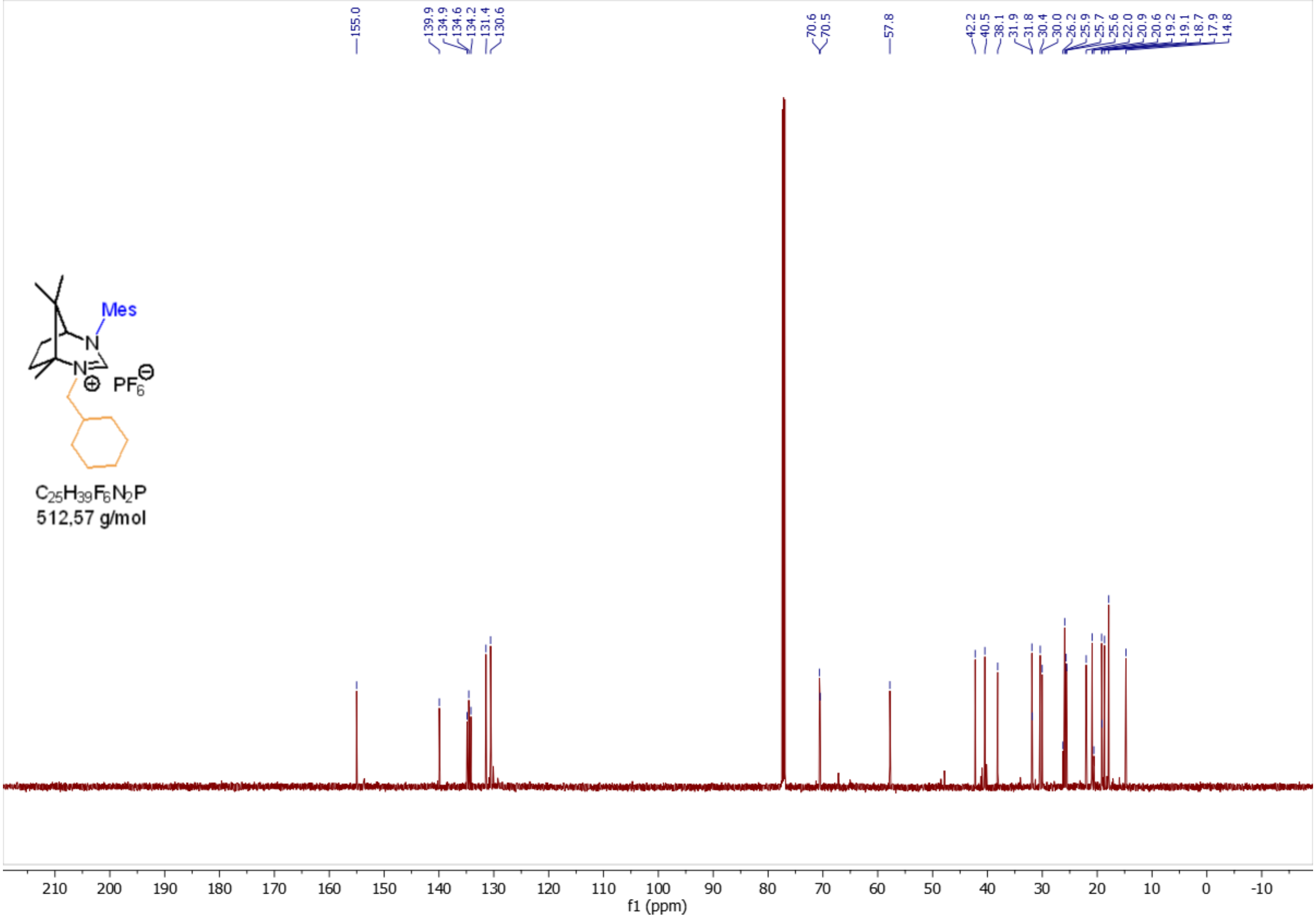

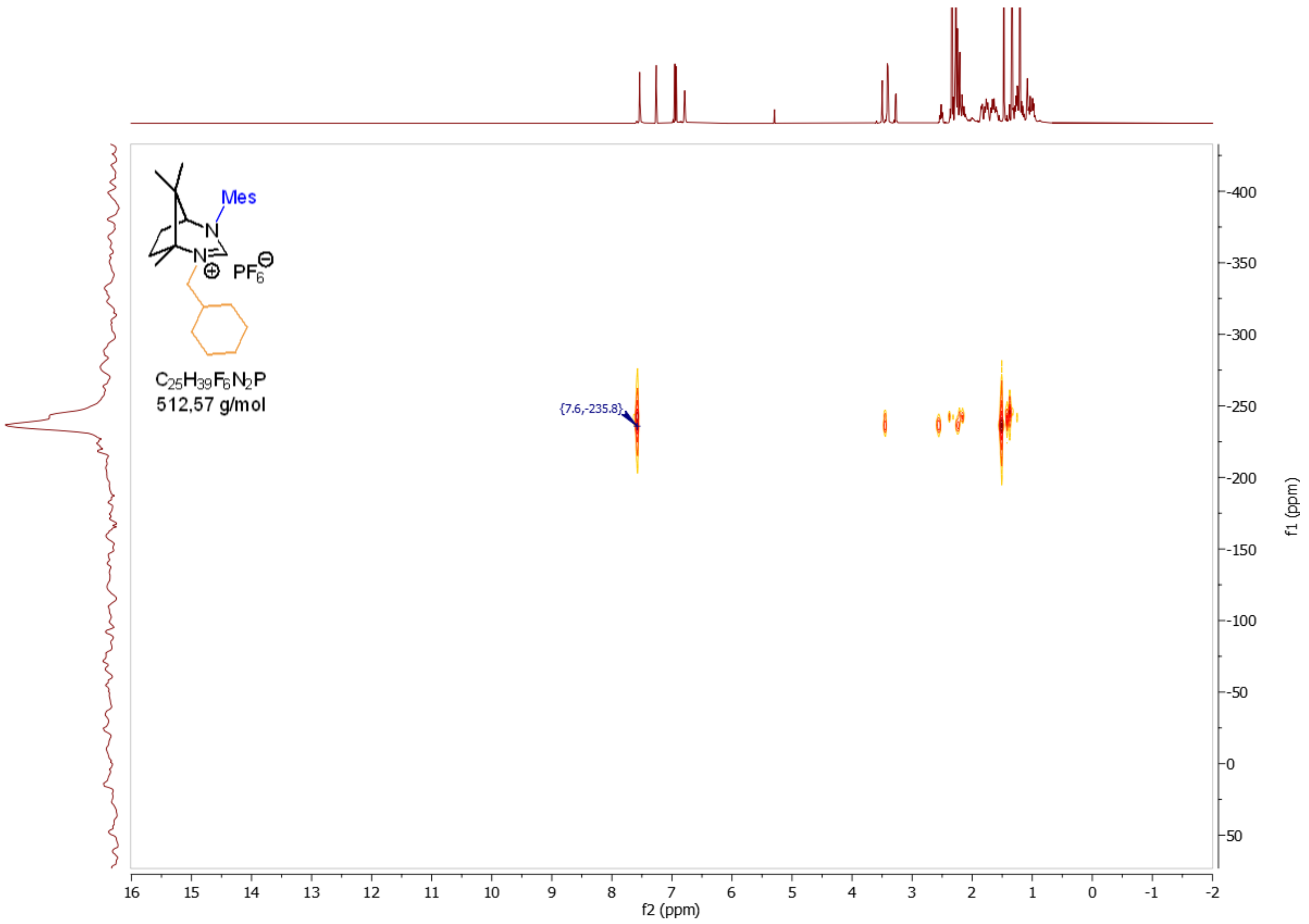
Mes
N
N
PF6
C25H39F6N2P
512,57 g/mol
{7.6,-235.8}
f2 (ppm)
f1 (ppm)

^{1}H NMR (600 MHz, $CDCl_3$), ^{13}C{^{1}H} NMR (151 MHz, $CDCl_3$) and ^{15}N HSQC NMR (61 MHz, $CDCl_3$) Analysis of Compound **3ai**

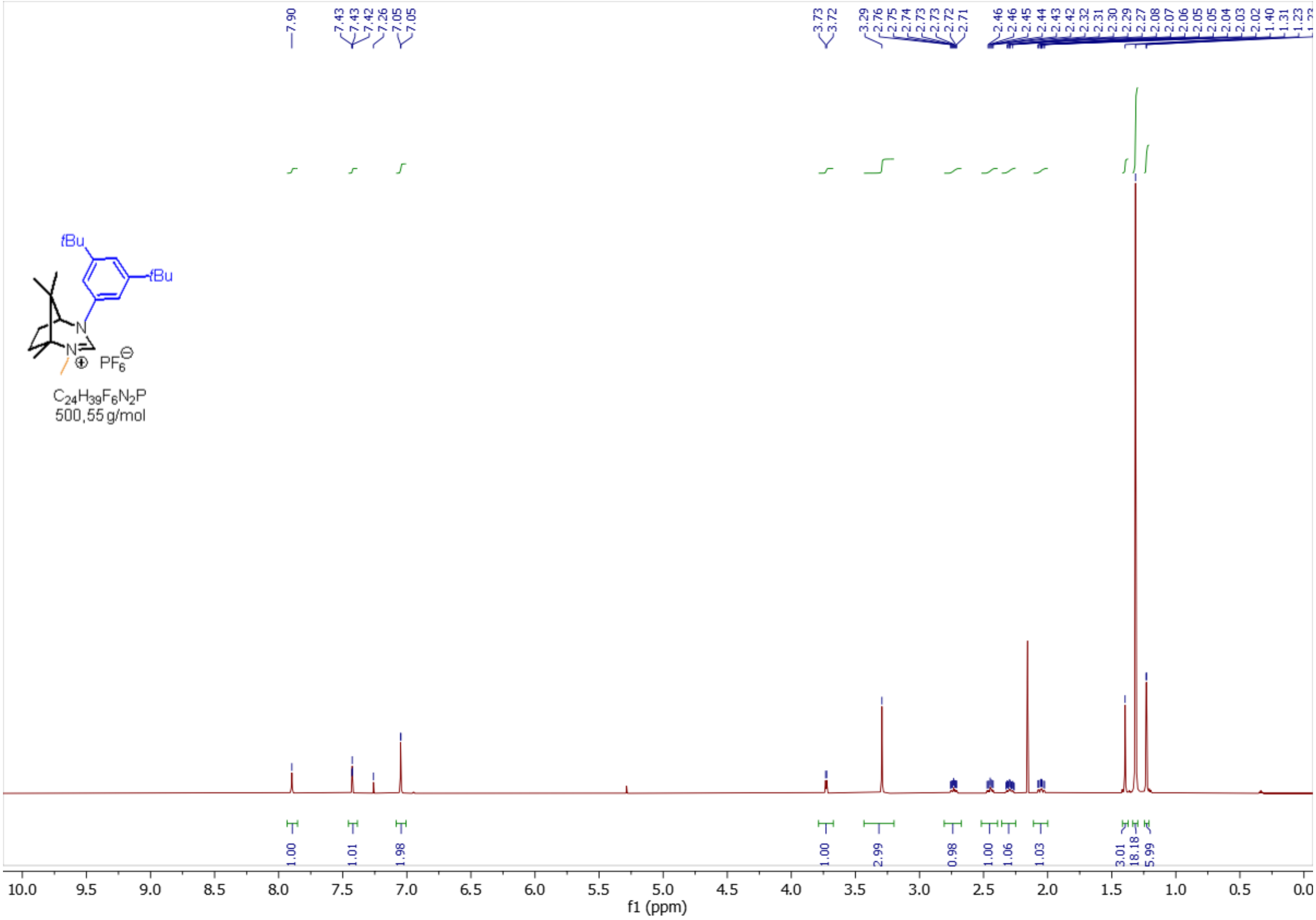

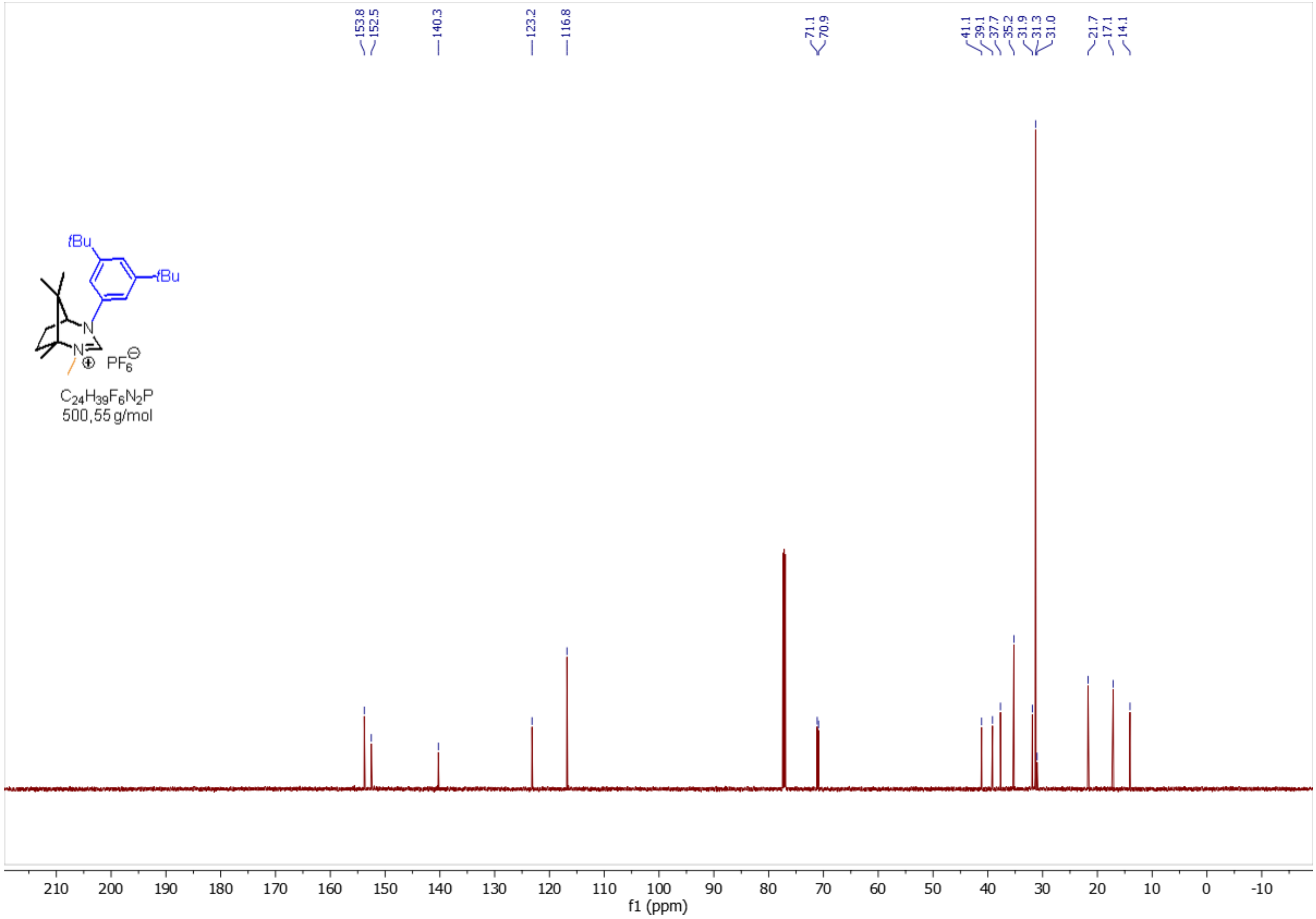

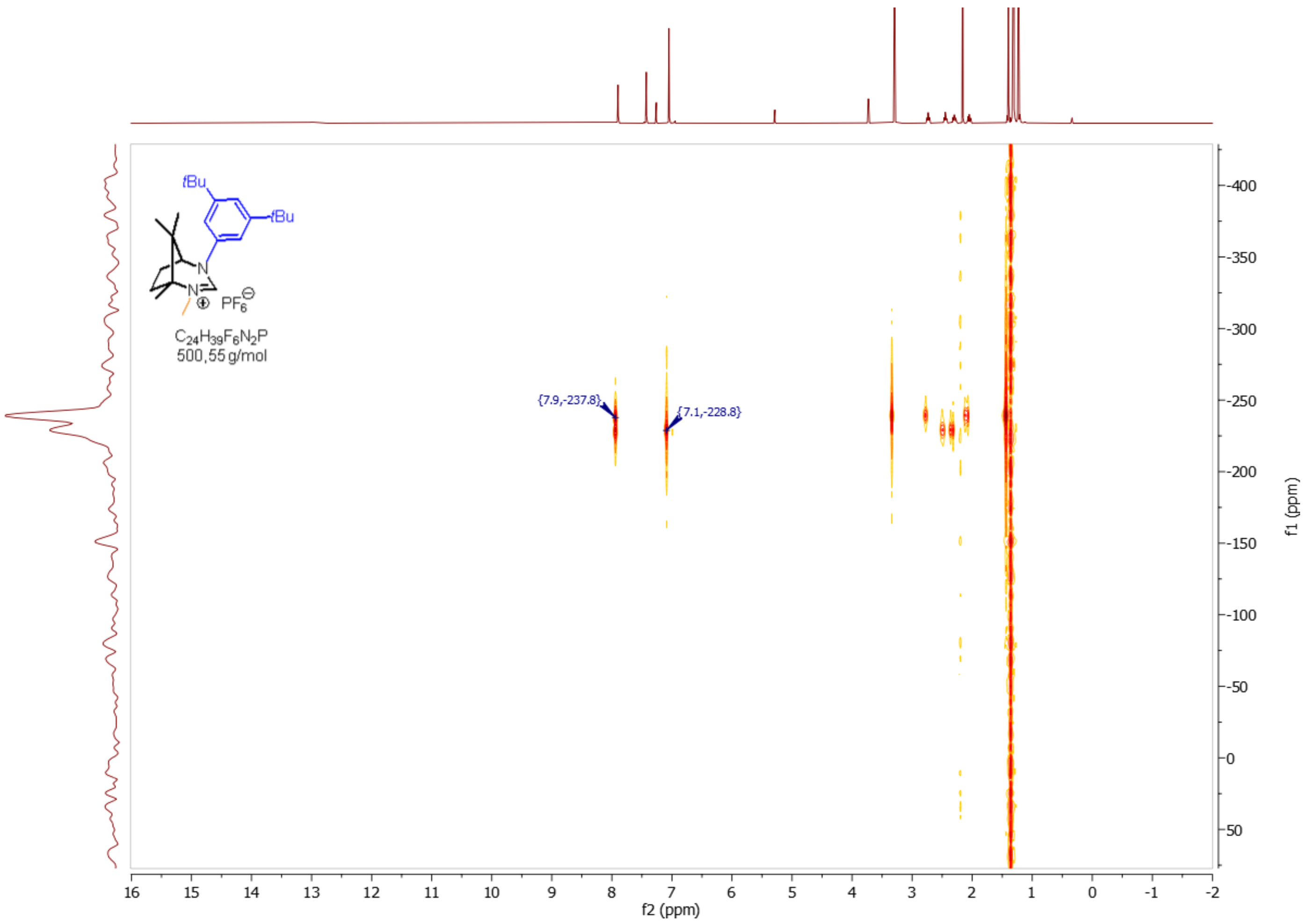
tBu
tBu
N
N
PF6
C24H39F6N2P
500,55 g/mol
{7.9,-237.8}
{7.1,-228.8}
f2 (ppm)
f1 (ppm)

^{1}H NMR (600 MHz, $CDCl_3$), $^{13}C\{^{1}H\}$ NMR (151 MHz, $CDCl_3$) and ^{15}N HSQC NMR (61 MHz, $CDCl_3$) Analysis of Compound **3ba**

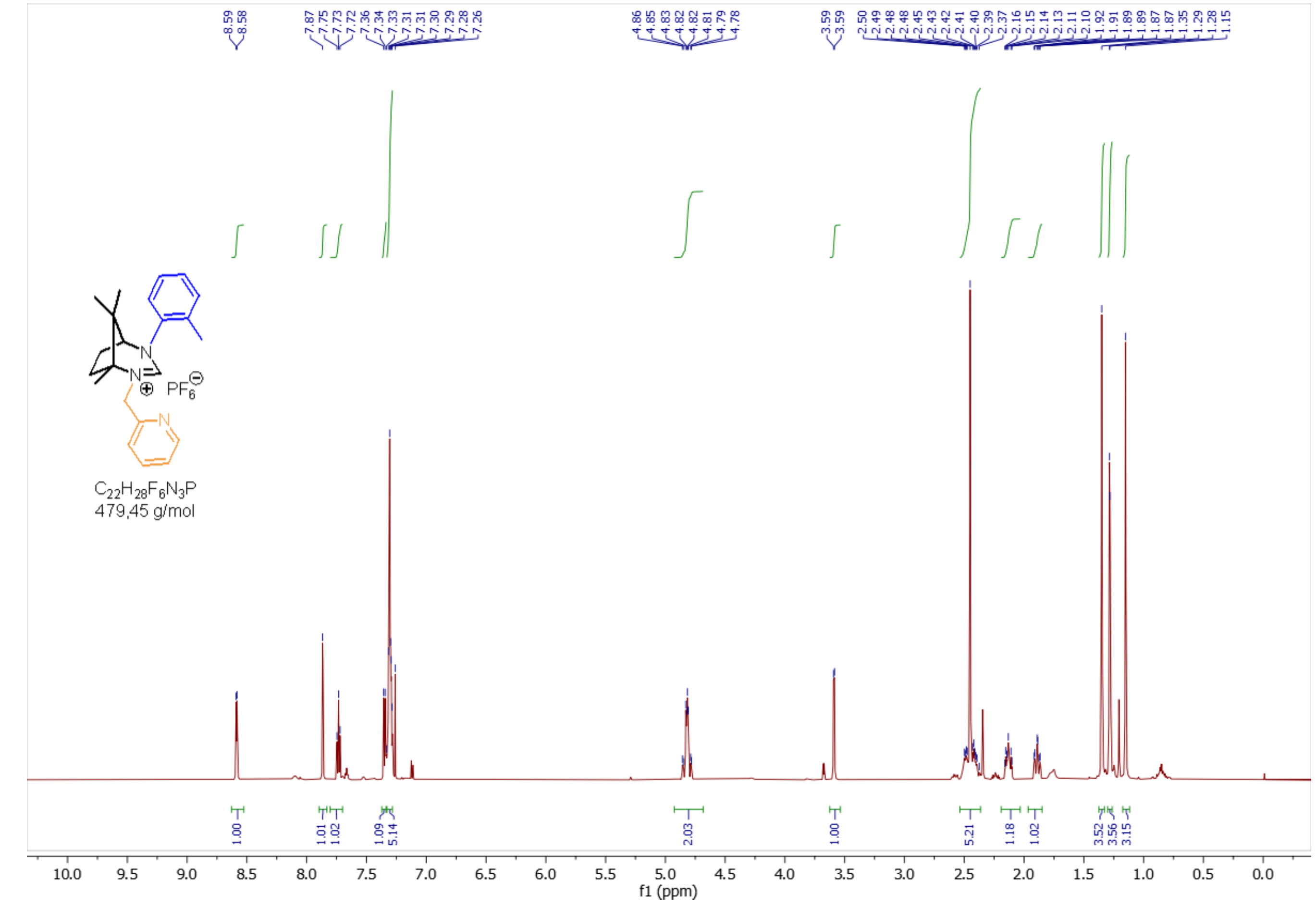

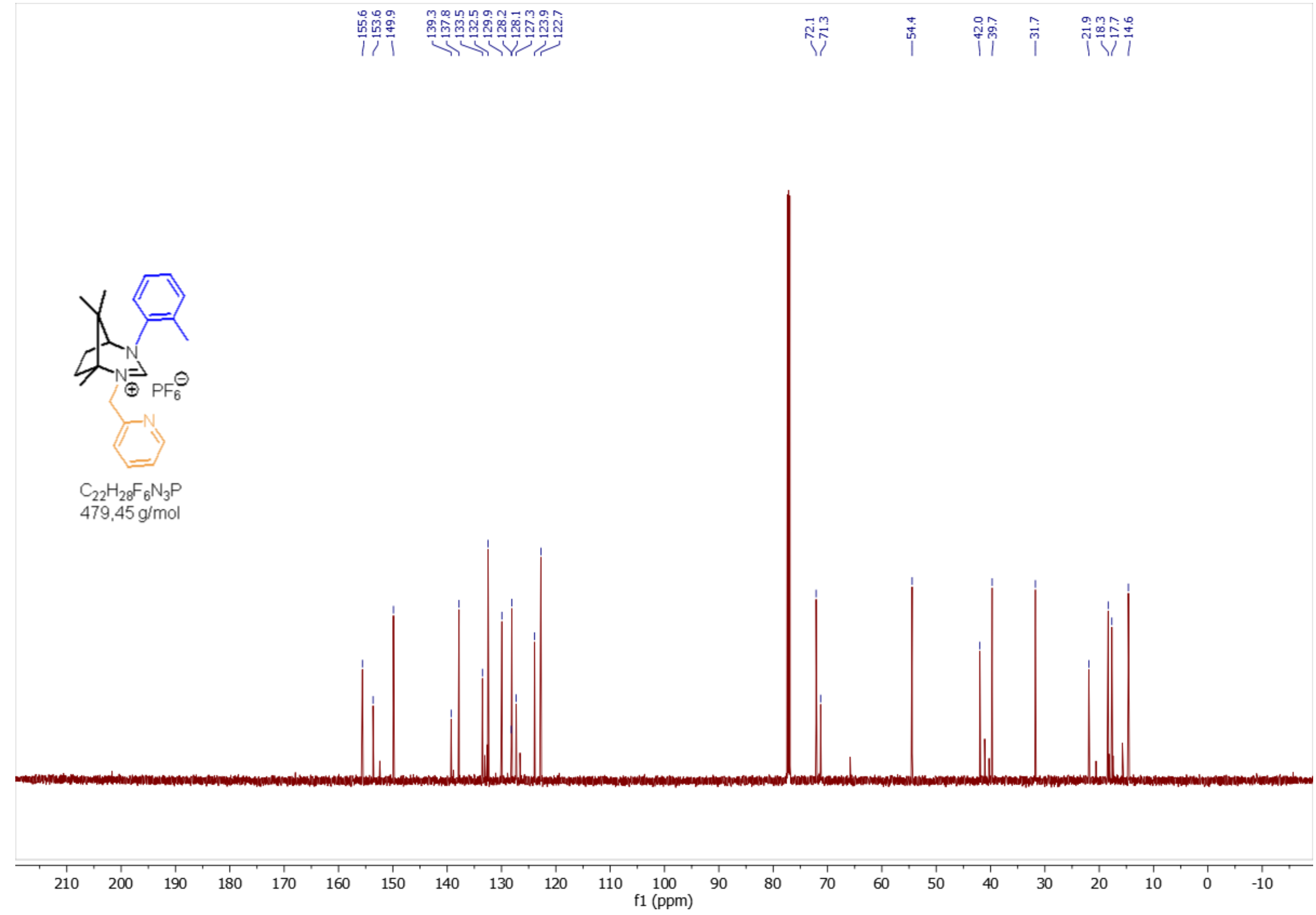

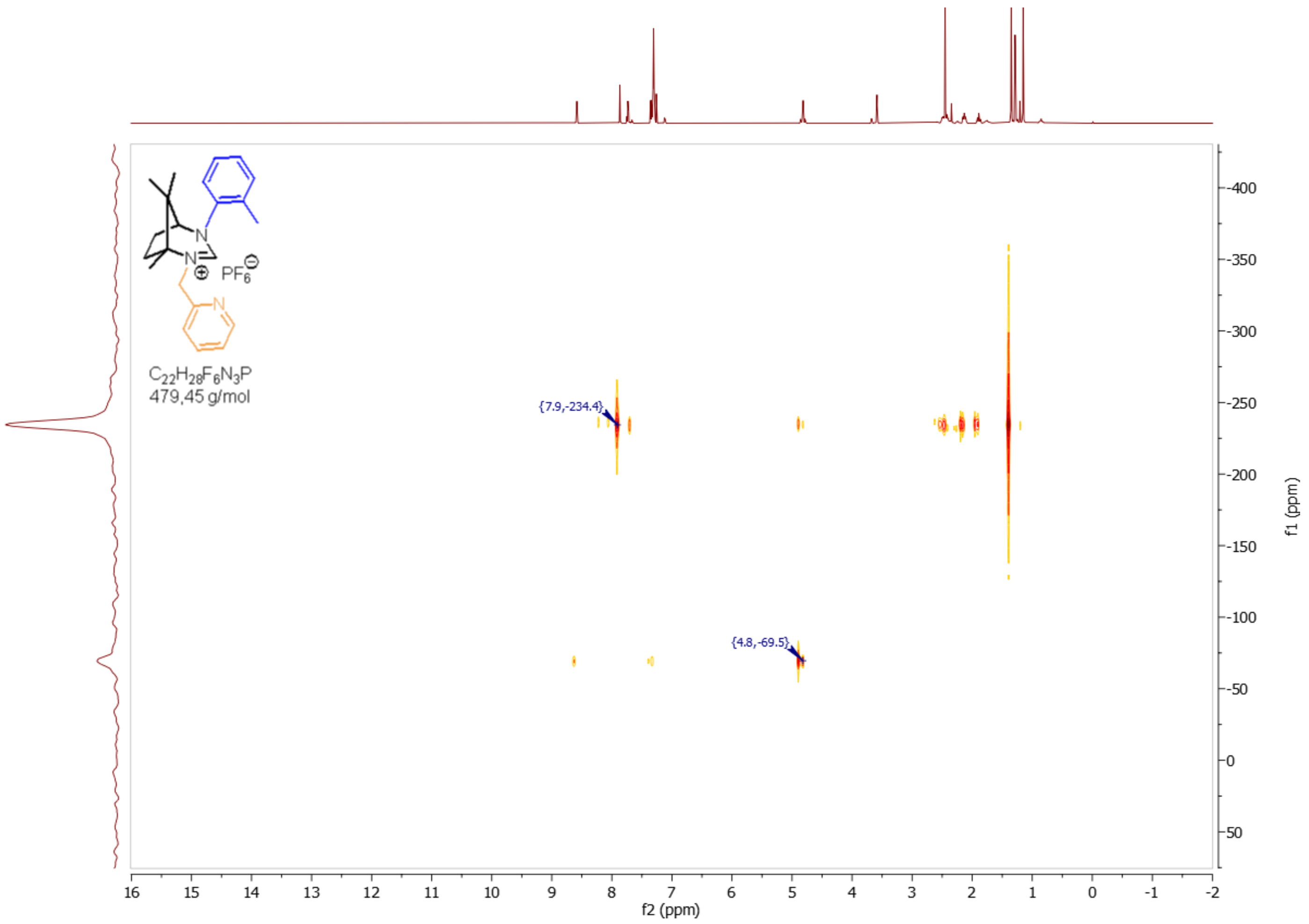

PF6
C22H28F6N3P
479,45 g/mol
{7.9,-234.4}
{4.8,-69.5}
f2 (ppm)
f1 (ppm)

^{1}H NMR (600 MHz, $CDCl_3$), $^{13}C\{^{1}H\}$ NMR (151 MHz, $CDCl_3$) and ^{15}N HSQC NMR (61 MHz, $CDCl_3$) Analysis of Compound **3bb**

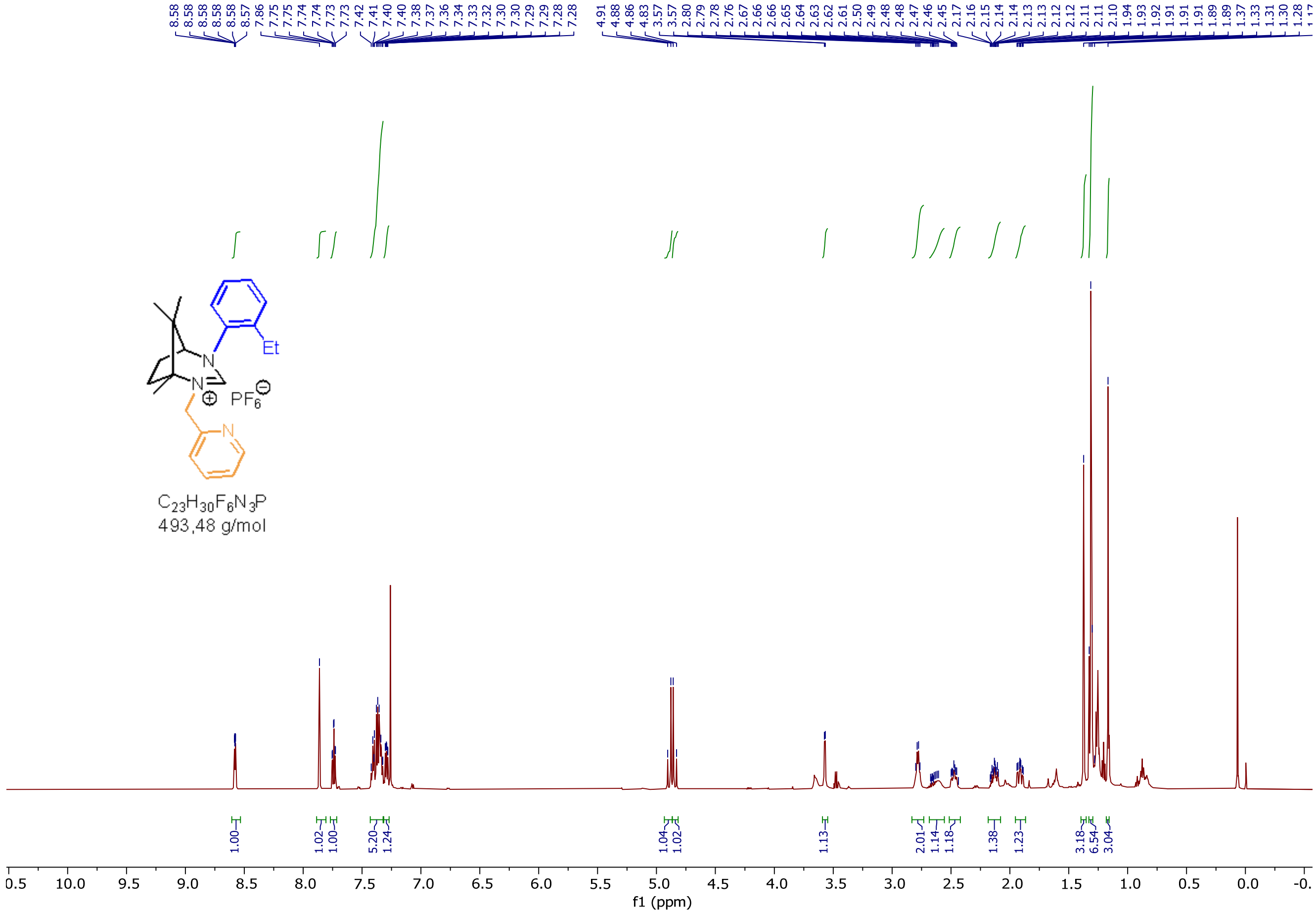

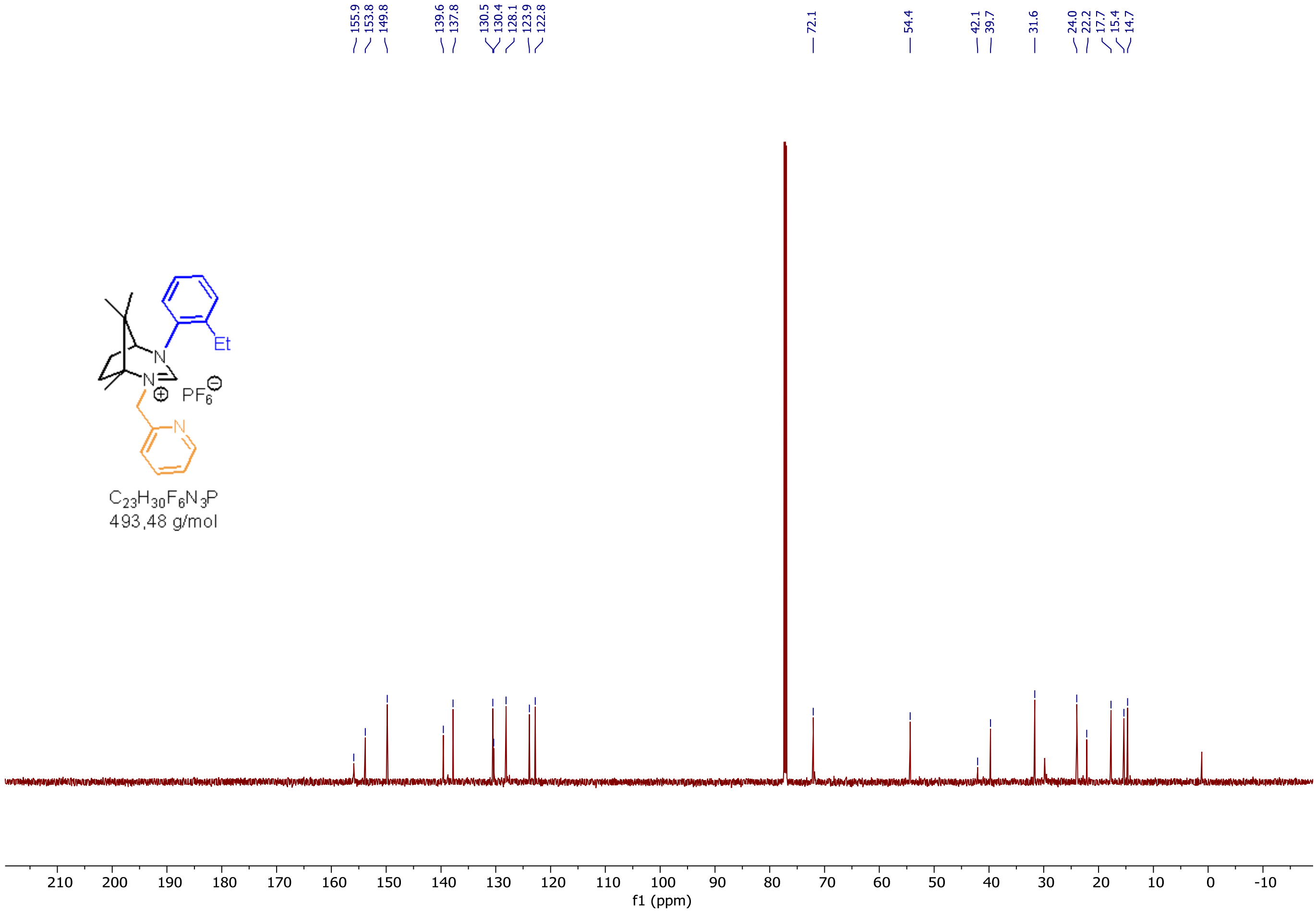

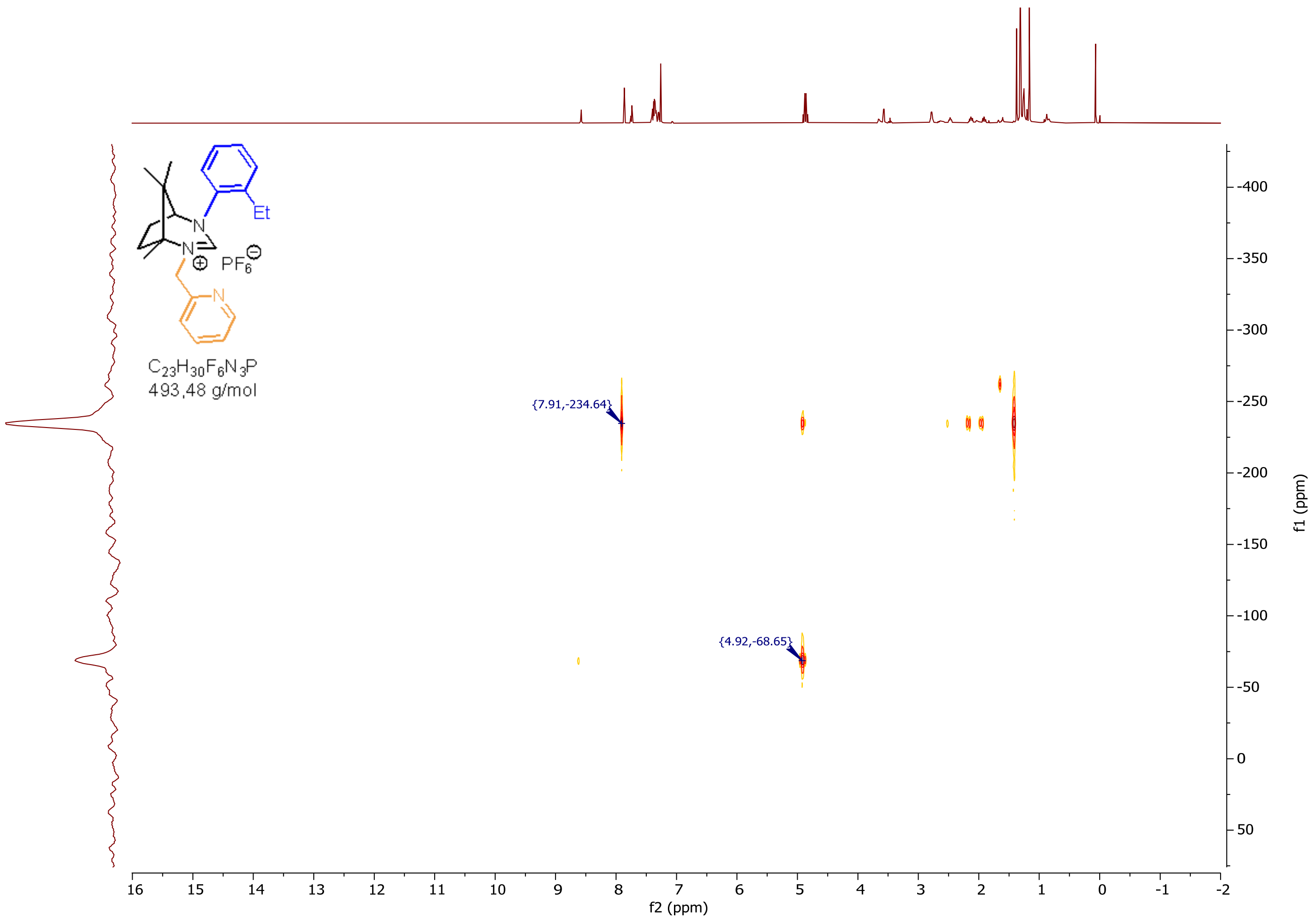
Et
N
N
PF6
N
C23H30F6N3P
493,48 g/mol
{7.91,-234.64}
{4.92,-68.65}
f1 (ppm)
f2 (ppm)

^{1}H NMR (600 MHz, TCE), $^{13}C\{^{1}H\}$ NMR (151 MHz, TCE) and ^{15}N HSQC NMR (61 MHz, TCE) Analysis of Compound **3bc** at 80 °C

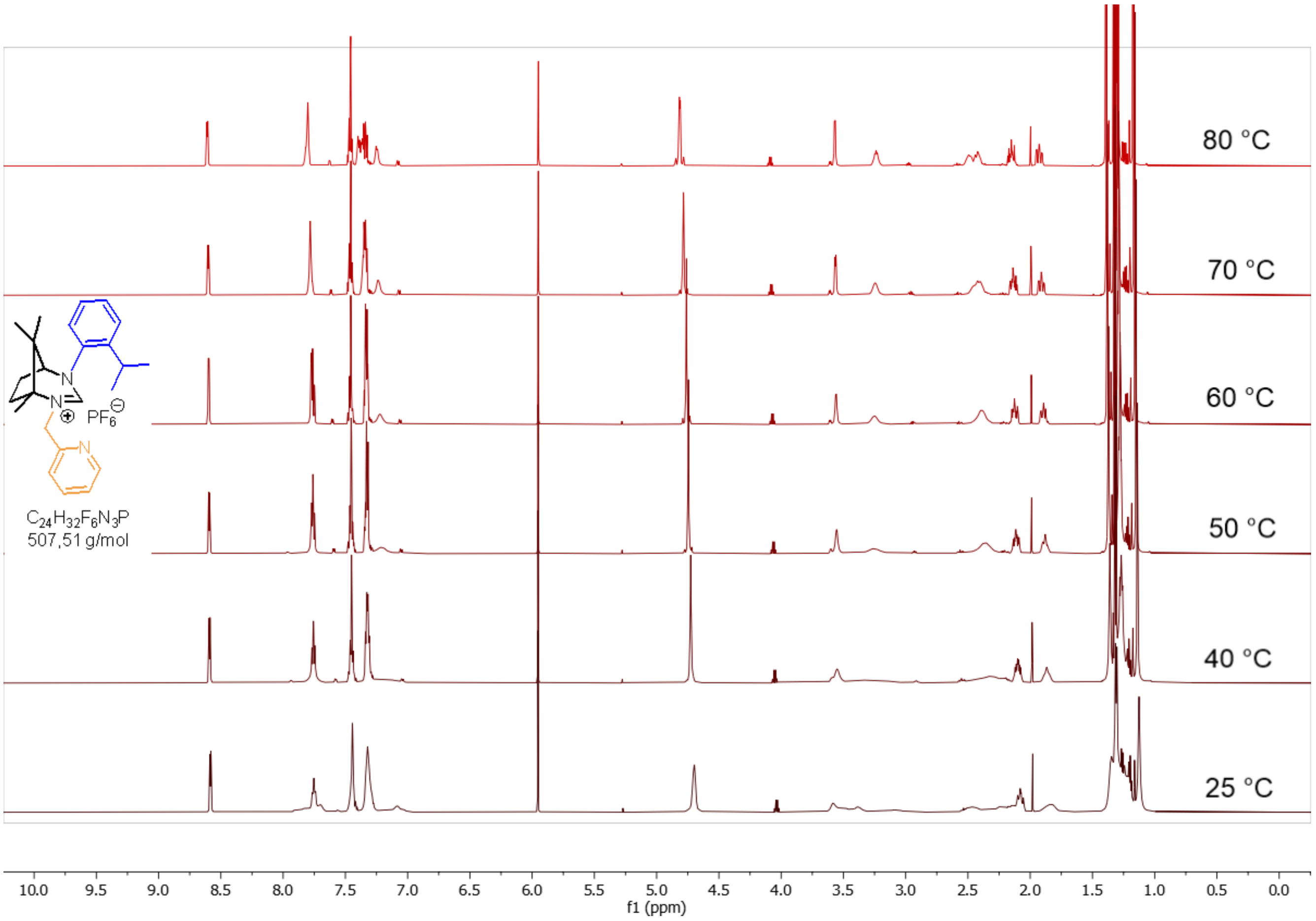

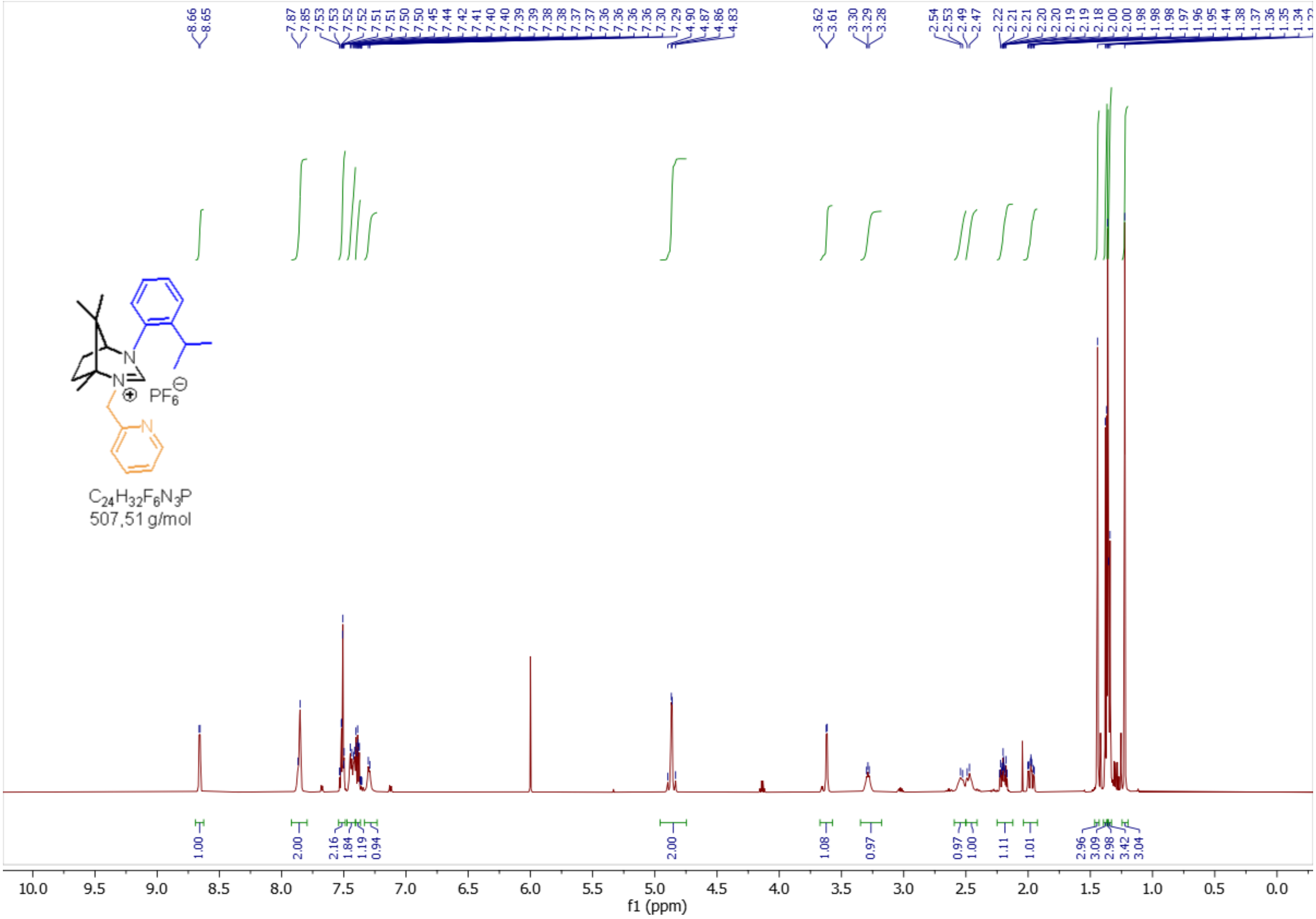

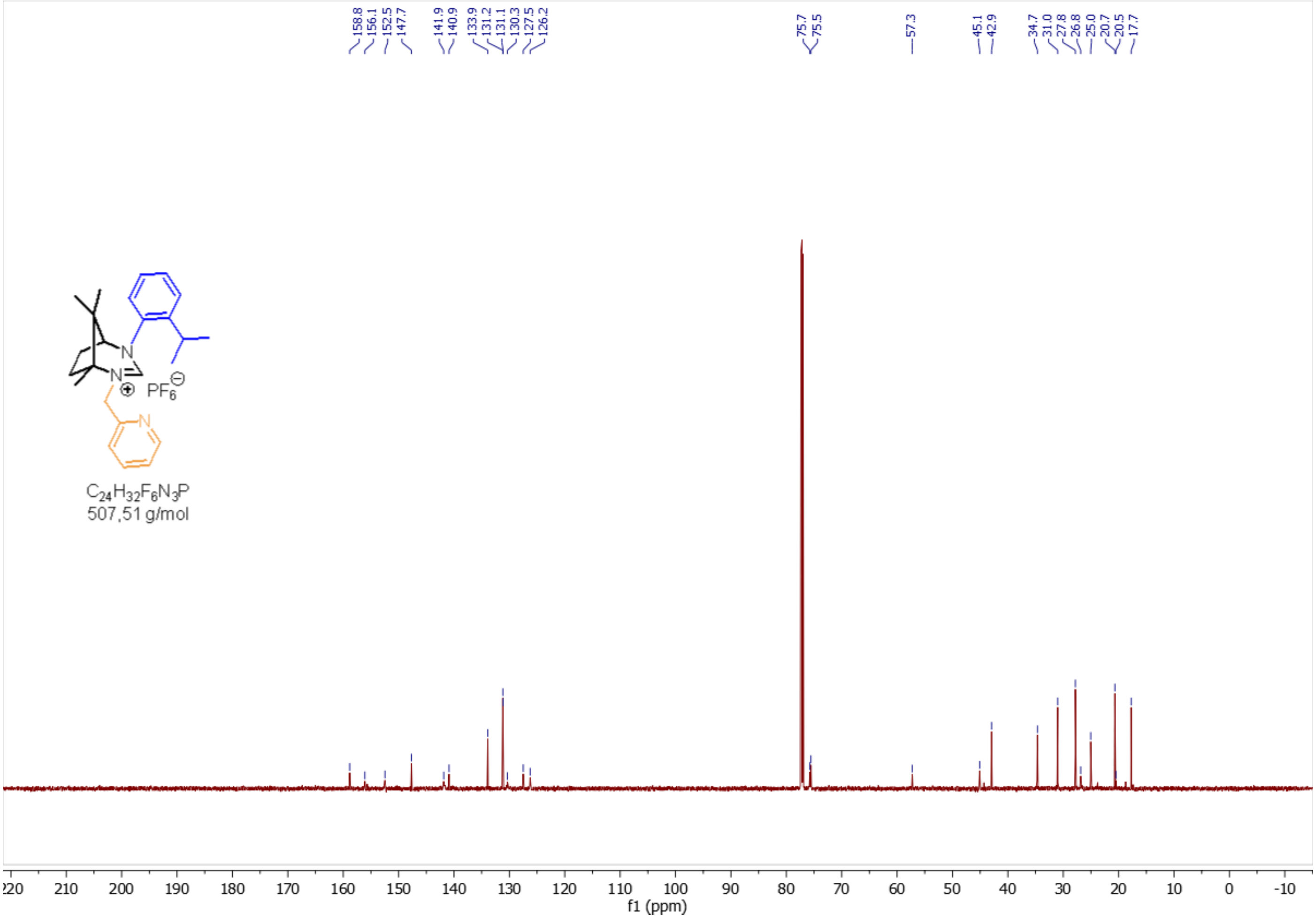

158.8
156.1
152.5
147.7
141.9
140.9
133.9
131.2
131.1
130.3
127.5
126.2
75.7
75.5
57.3
45.1
42.9
34.7
31.0
27.8
26.8
25.0
20.7
20.5
17.7
$PF_6^{\ominus}$
$C_{24}H_{32}F_6N_3P$
507,51 g/mol
f1 (ppm)

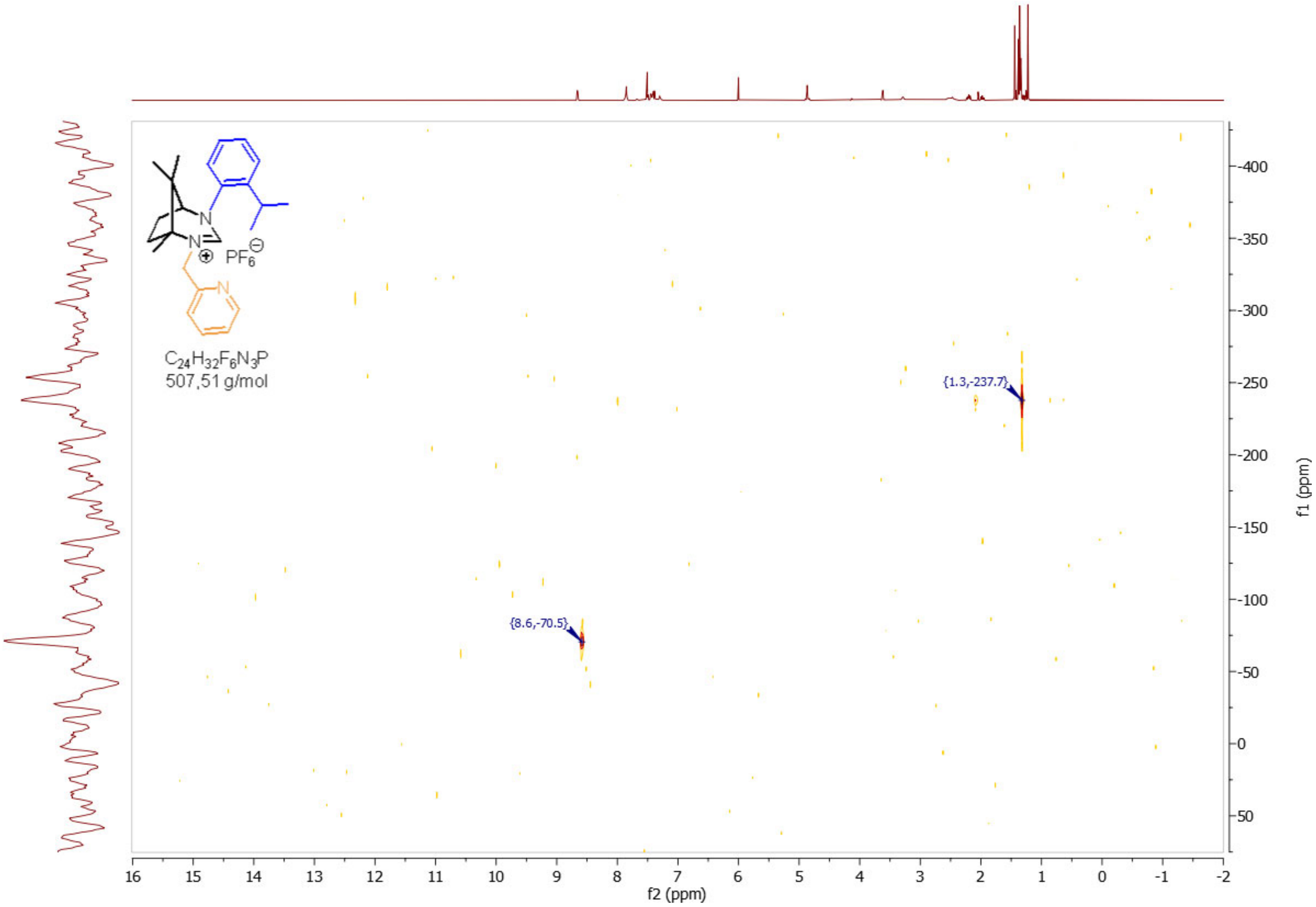

$PF_6^{\ominus}$
$C_{24}H_{32}F_6N_3P$
507,51 g/mol
{1.3,-237.7}
{8.6,-70.5}
f2 (ppm)
f1 (ppm)

^{1}H NMR (600 MHz, CD_2Cl_2), ^{13}C{^{1}H} NMR (151 MHz, CD_2Cl_2) and ^{15}N HSQC NMR (61 MHz, CD_2Cl_2) Analysis of Compound Compound **3bd**

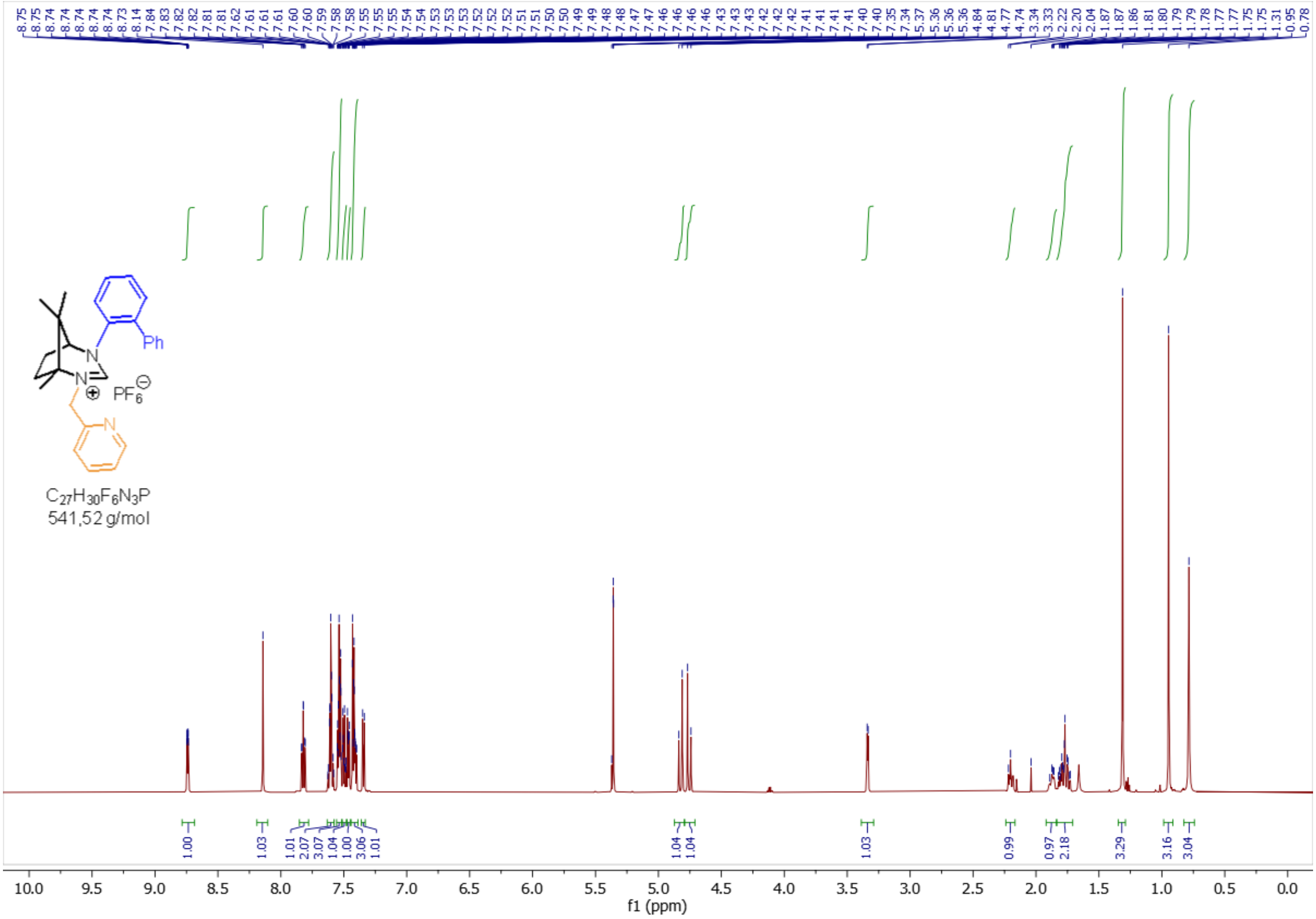

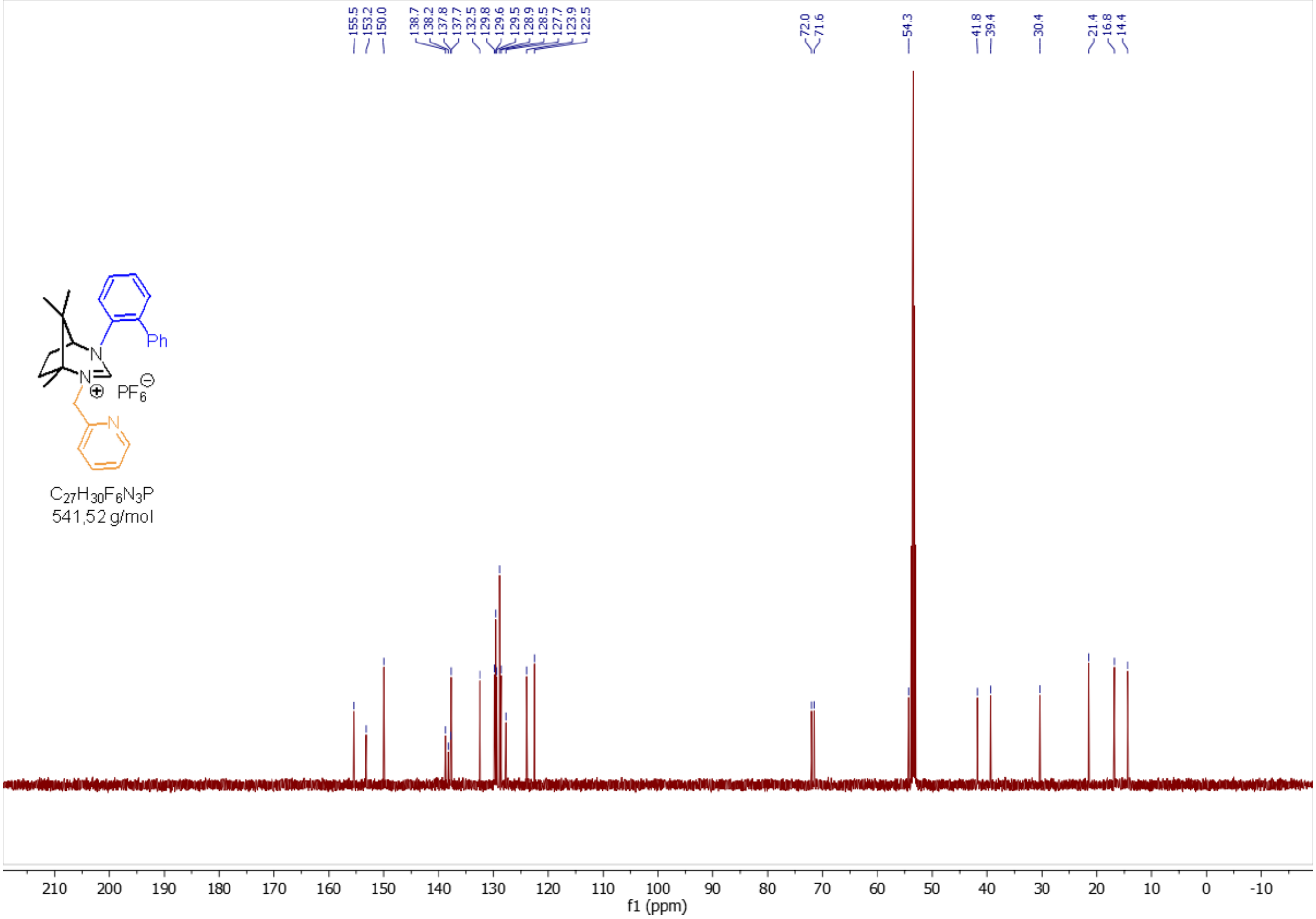

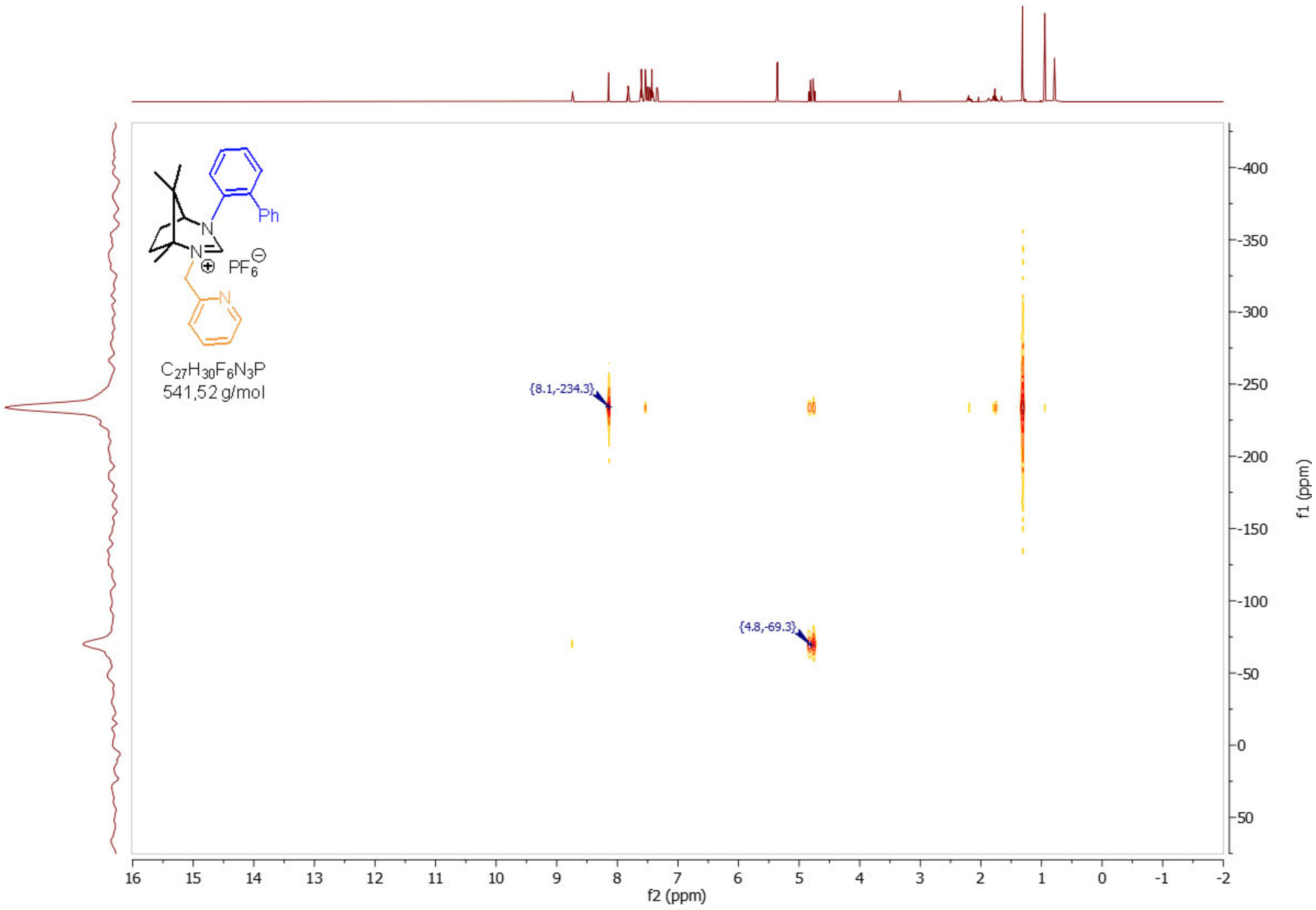
Ph
N
N
PF6
N
C27H30F6N3P
541,52 g/mol
{8.1,-234.3}
{4.8,-69.3}
f1 (ppm)
f2 (ppm)

^{1}H NMR (600 MHz, ACN), $^{13}C\{^{1}H\}$ NMR (151 MHz, ACN), ^{15}N HSQC NMR (61 MHz, ACN) and ^{19}F NMR (337 MHz, ACN) Analysis of Compound **3be**

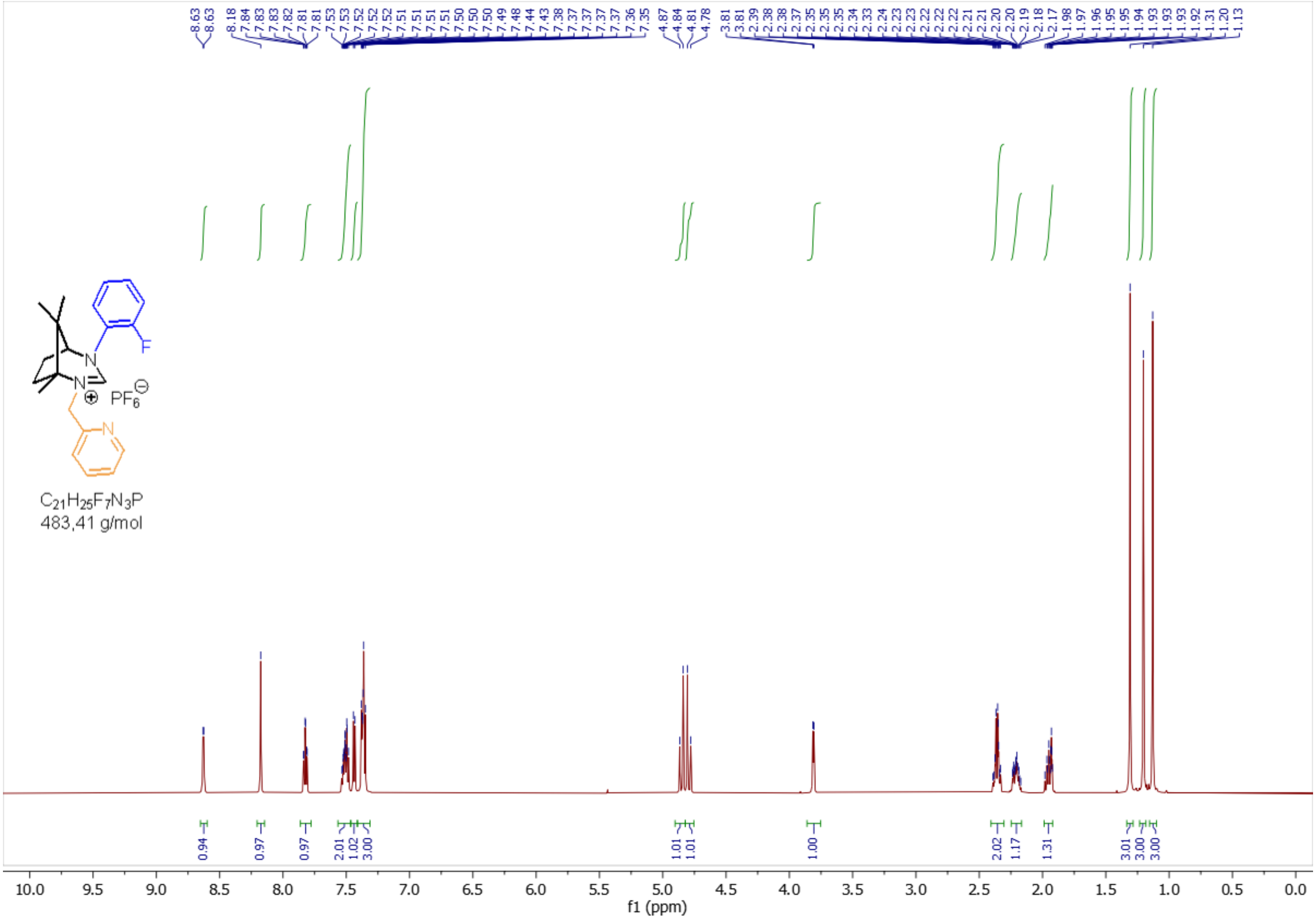

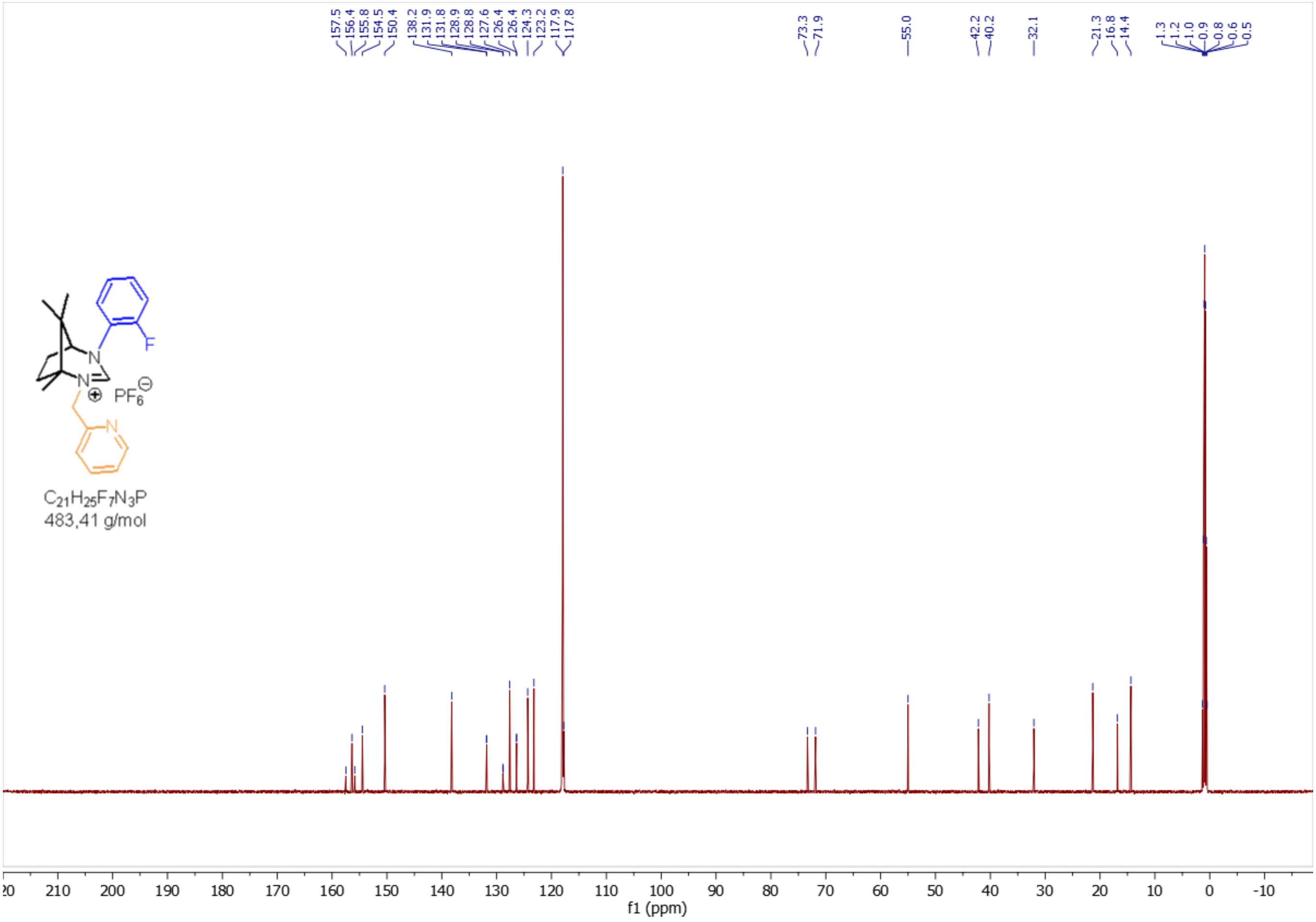

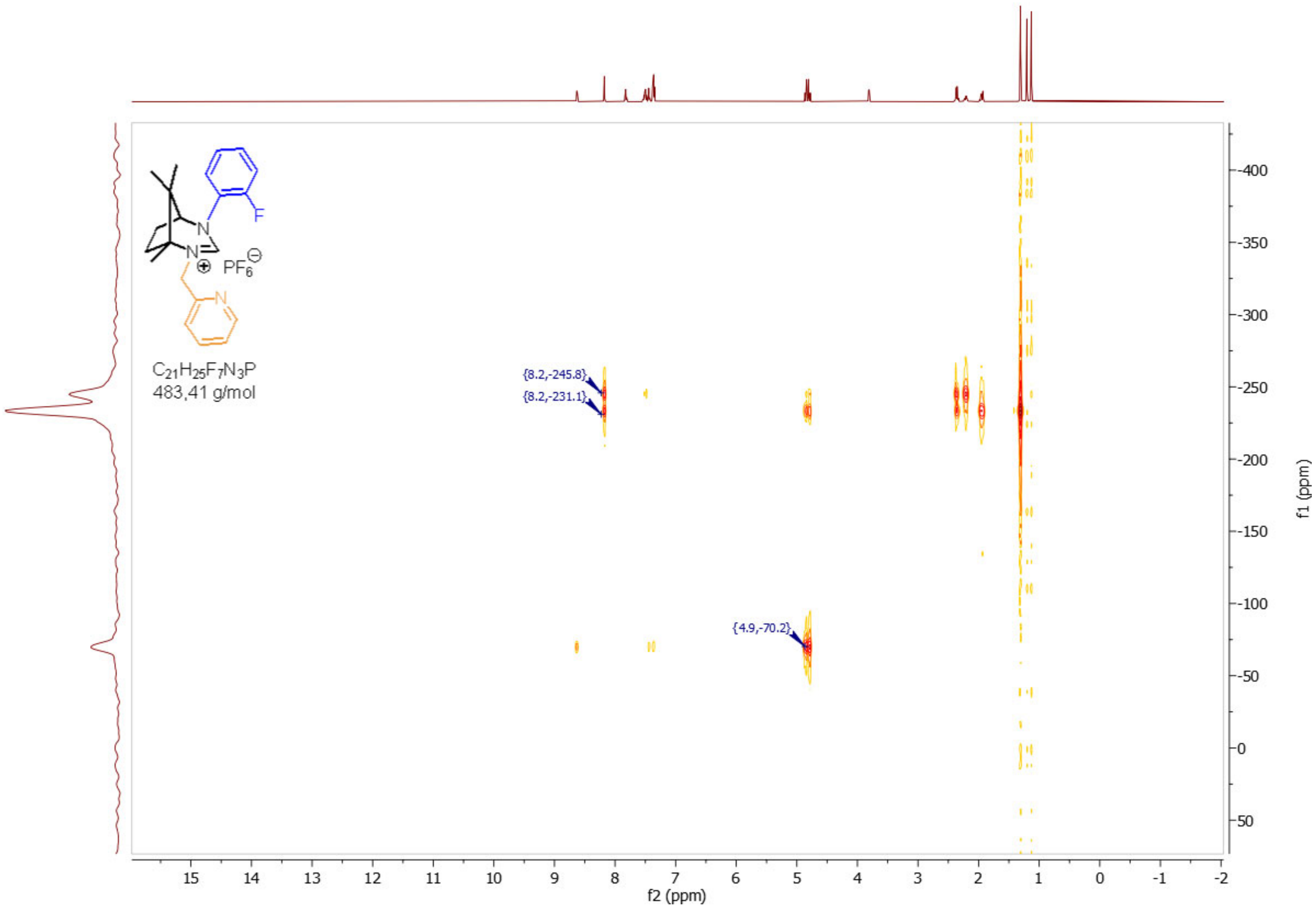
C21H25F7N3P
483,41 g/mol
{8.2,-245.8}
{8.2,-231.1}
{4.9,-70.2}
f2 (ppm)
f1 (ppm)

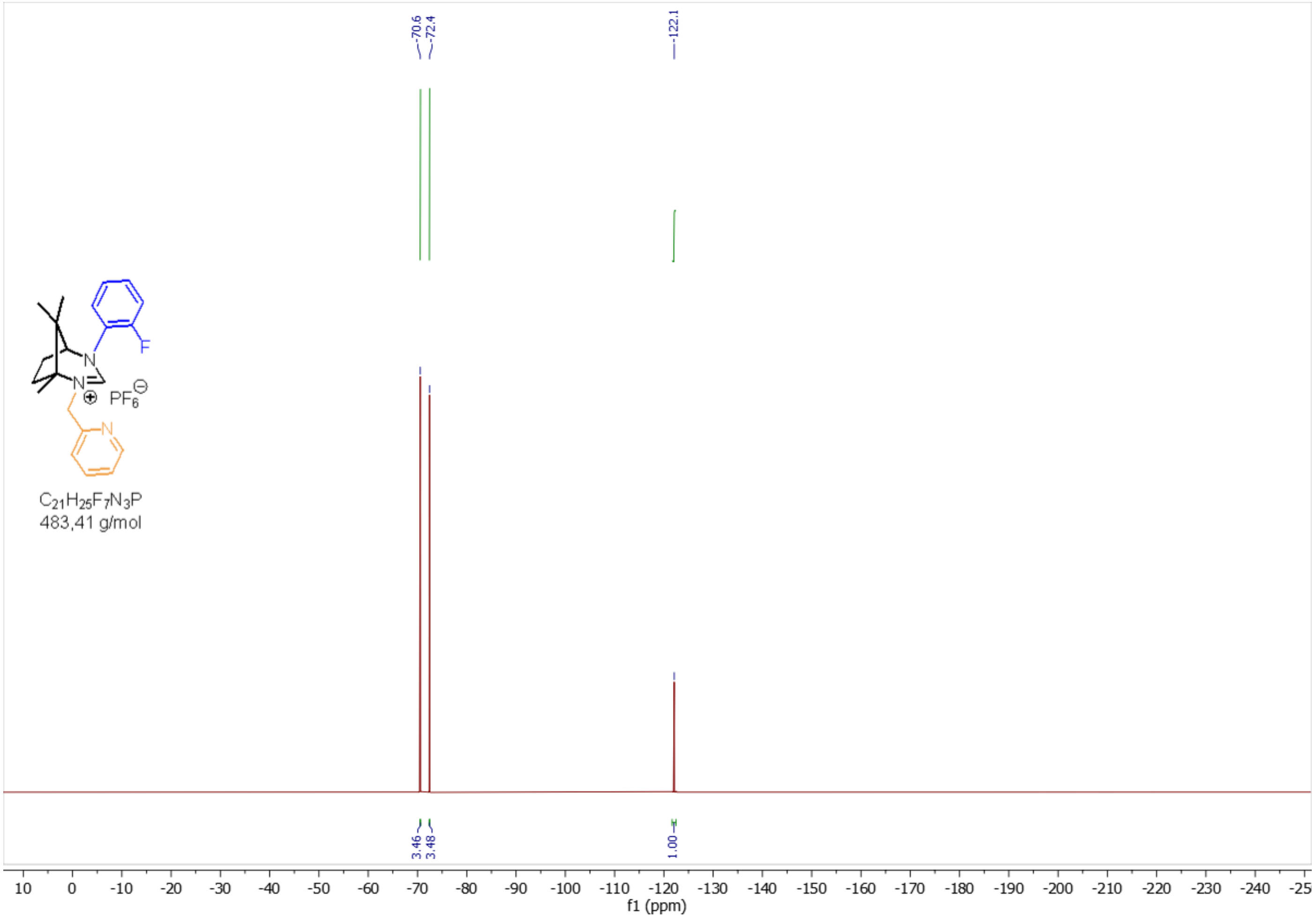
-70.6
-72.4
-122.1
C21H25F7N3P
483,41 g/mol
3.46
3.48
1.00
f1 (ppm)

^{1}H NMR (600 MHz, $CDCl_3$), ^{13}C{^{1}H} NMR (151 MHz, $CDCl_3$) and ^{15}N HSQC NMR (61 MHz, $CDCl_3$) Analysis of Compound **3bf**

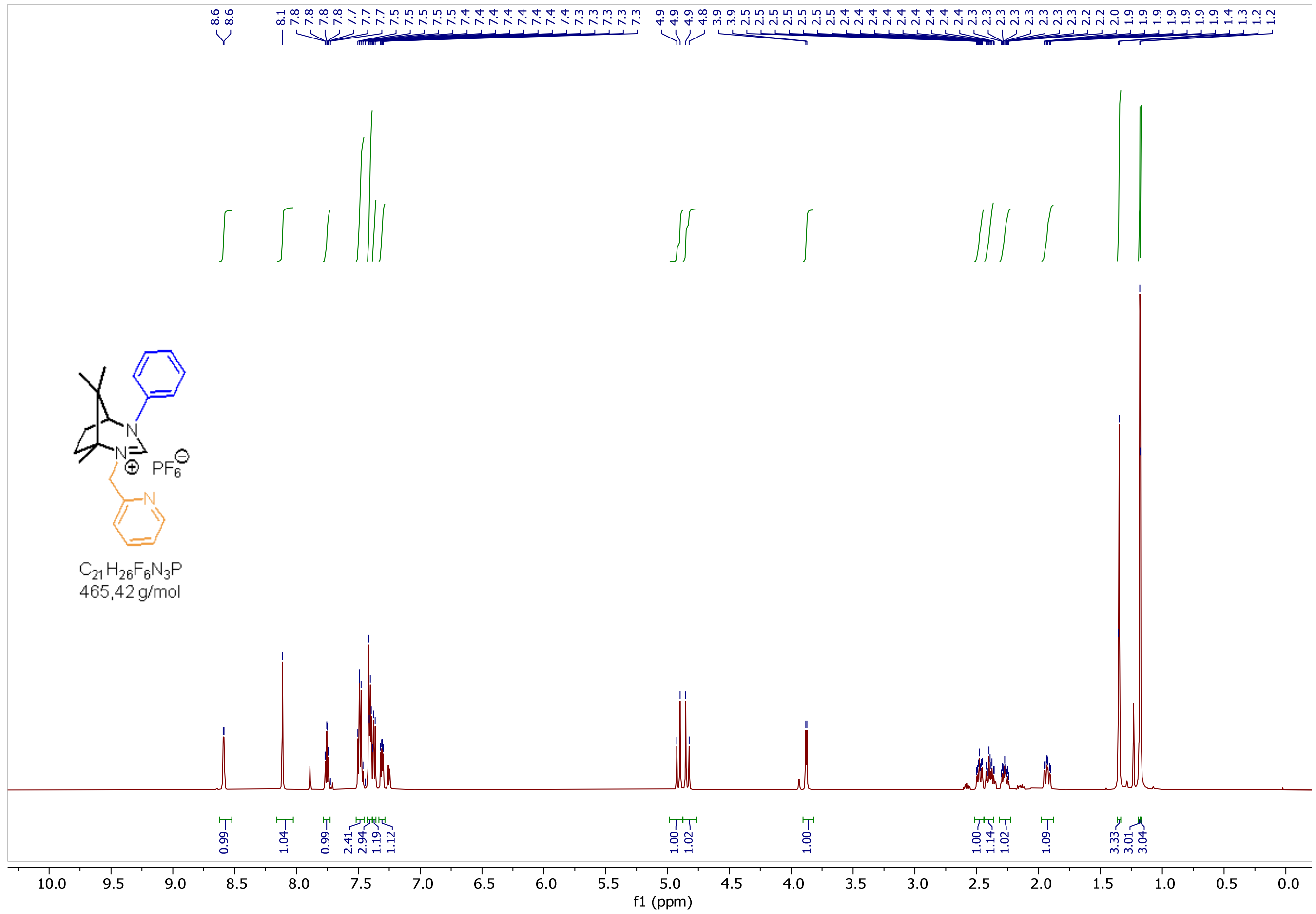

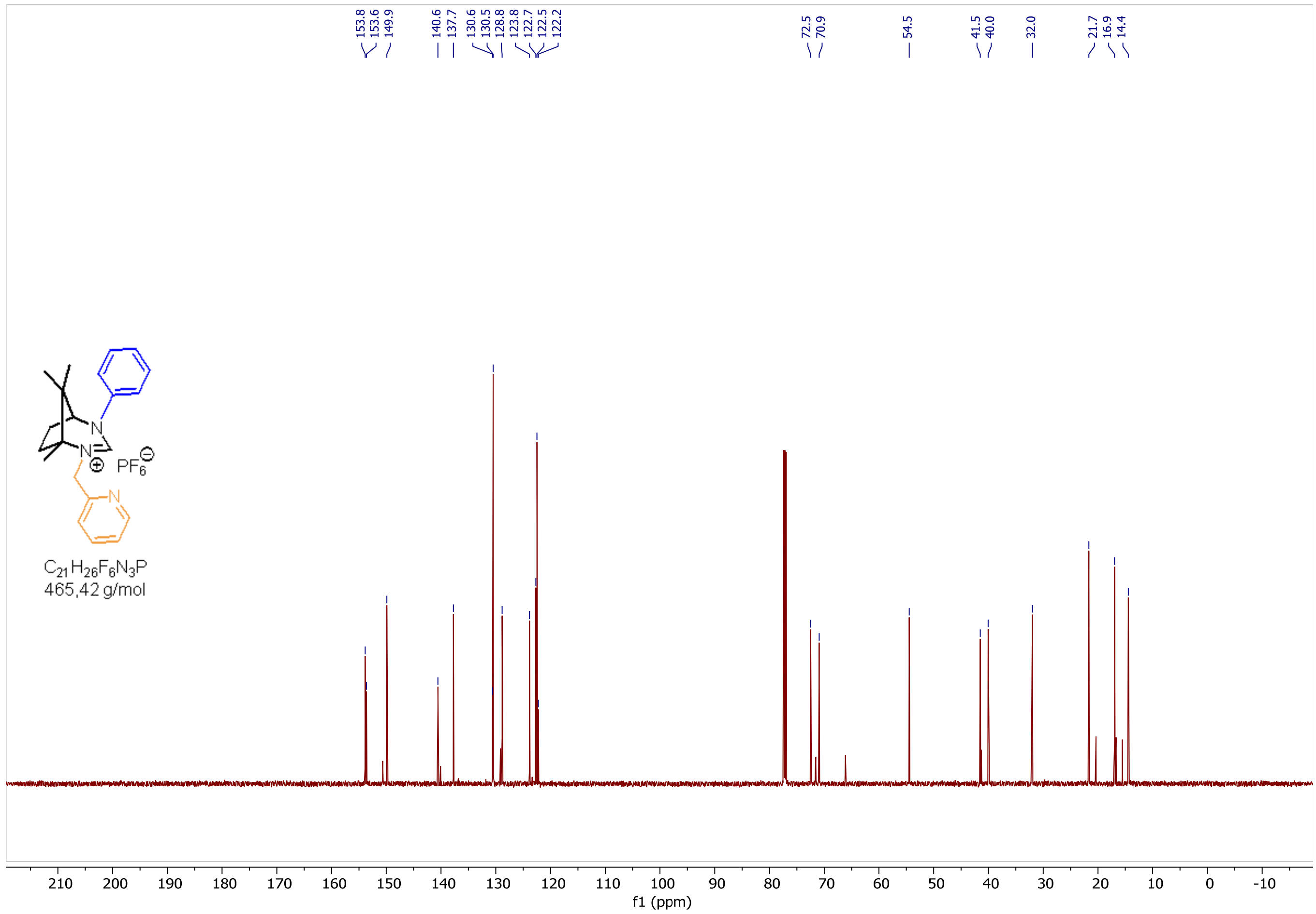

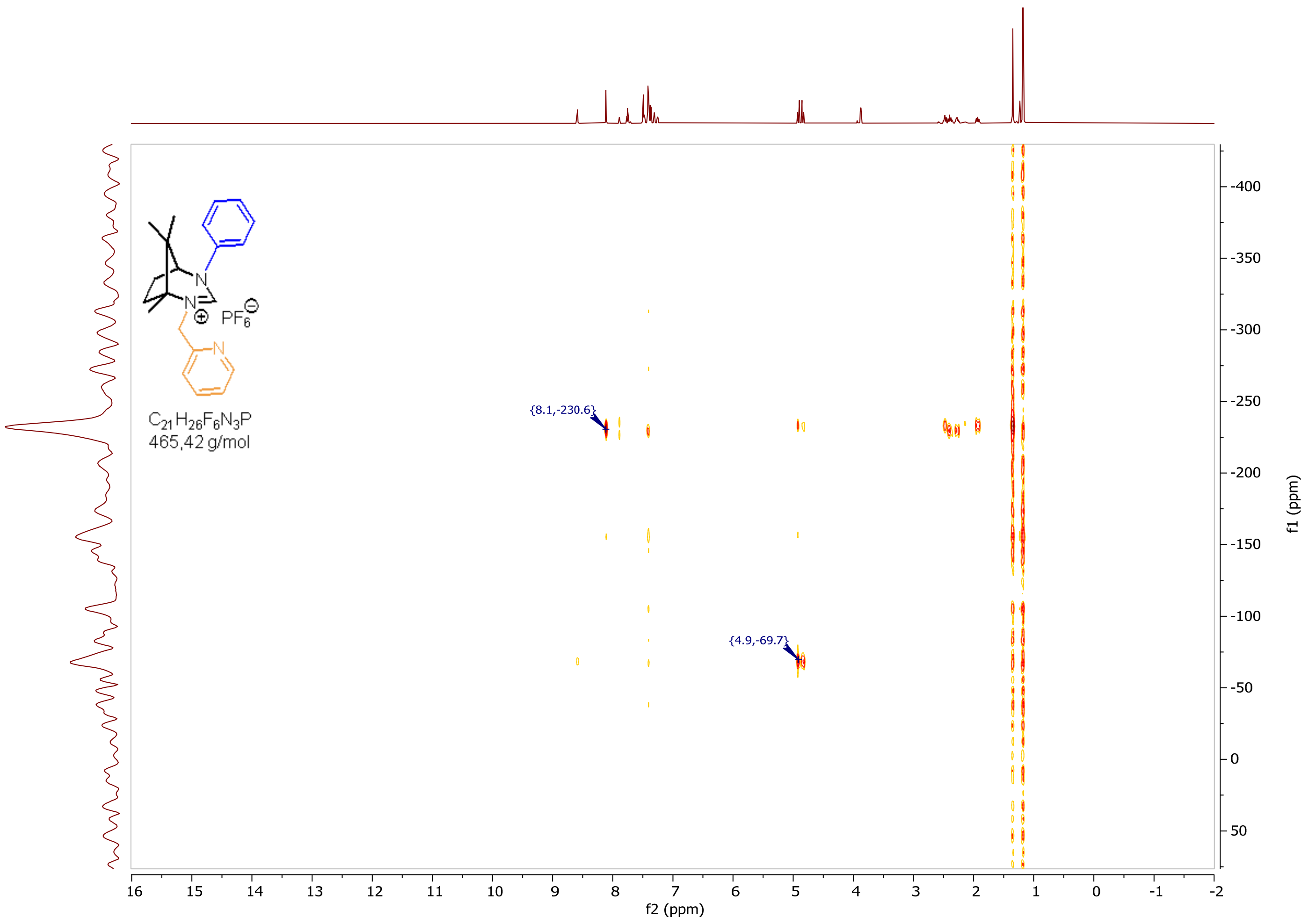
PF6
C21H26F6N3P
465,42 g/mol
{8.1,-230.6}
{4.9,-69.7}
f2 (ppm)
f1 (ppm)

^{1}H NMR (600 MHz, CDCl$_3$), ^{13}C{^{1}H} NMR (151 MHz, CDCl$_3$) and ^{15}N HSQC NMR (61 MHz, CDCl$_3$) Analysis of Compound **3bg**

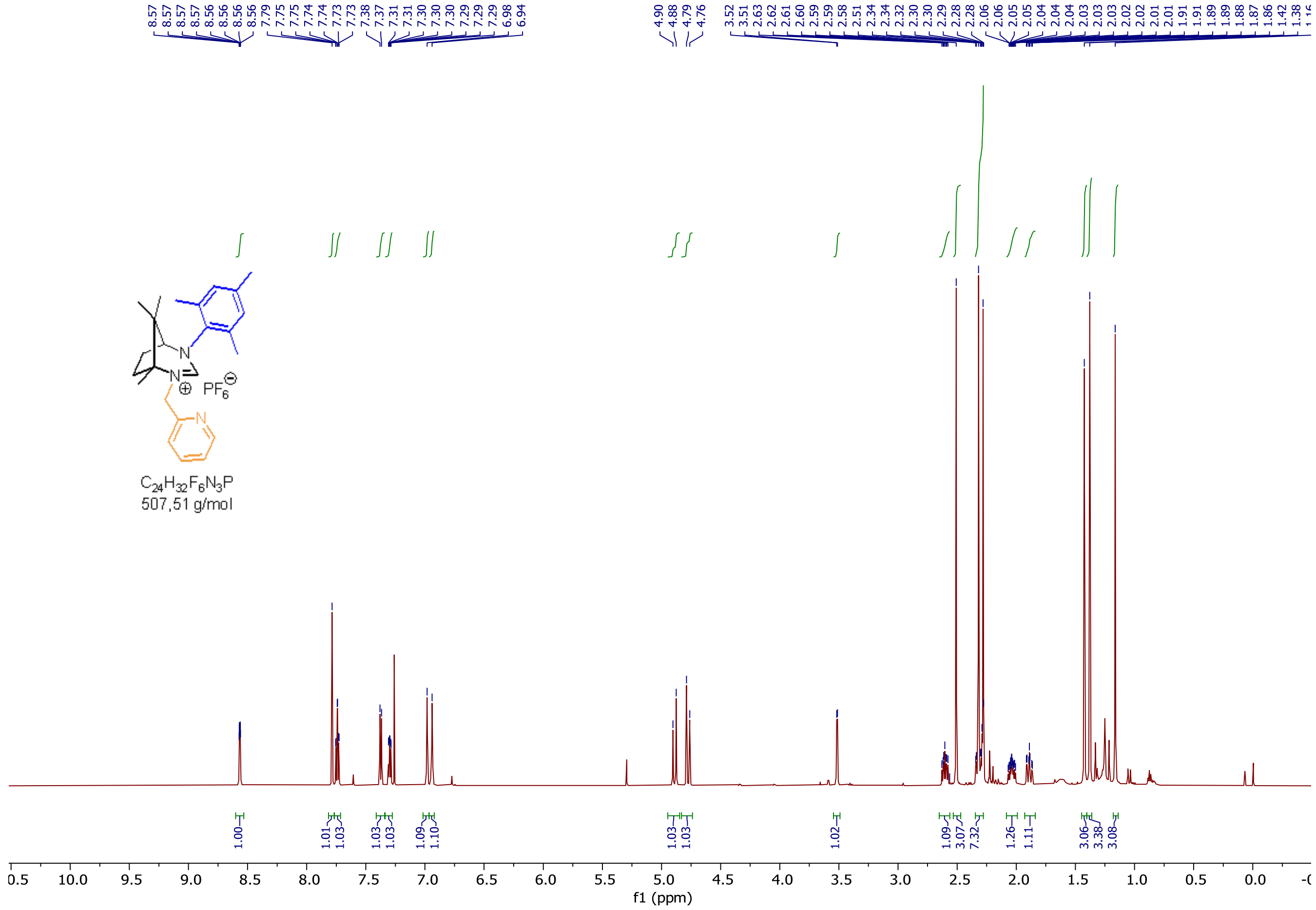

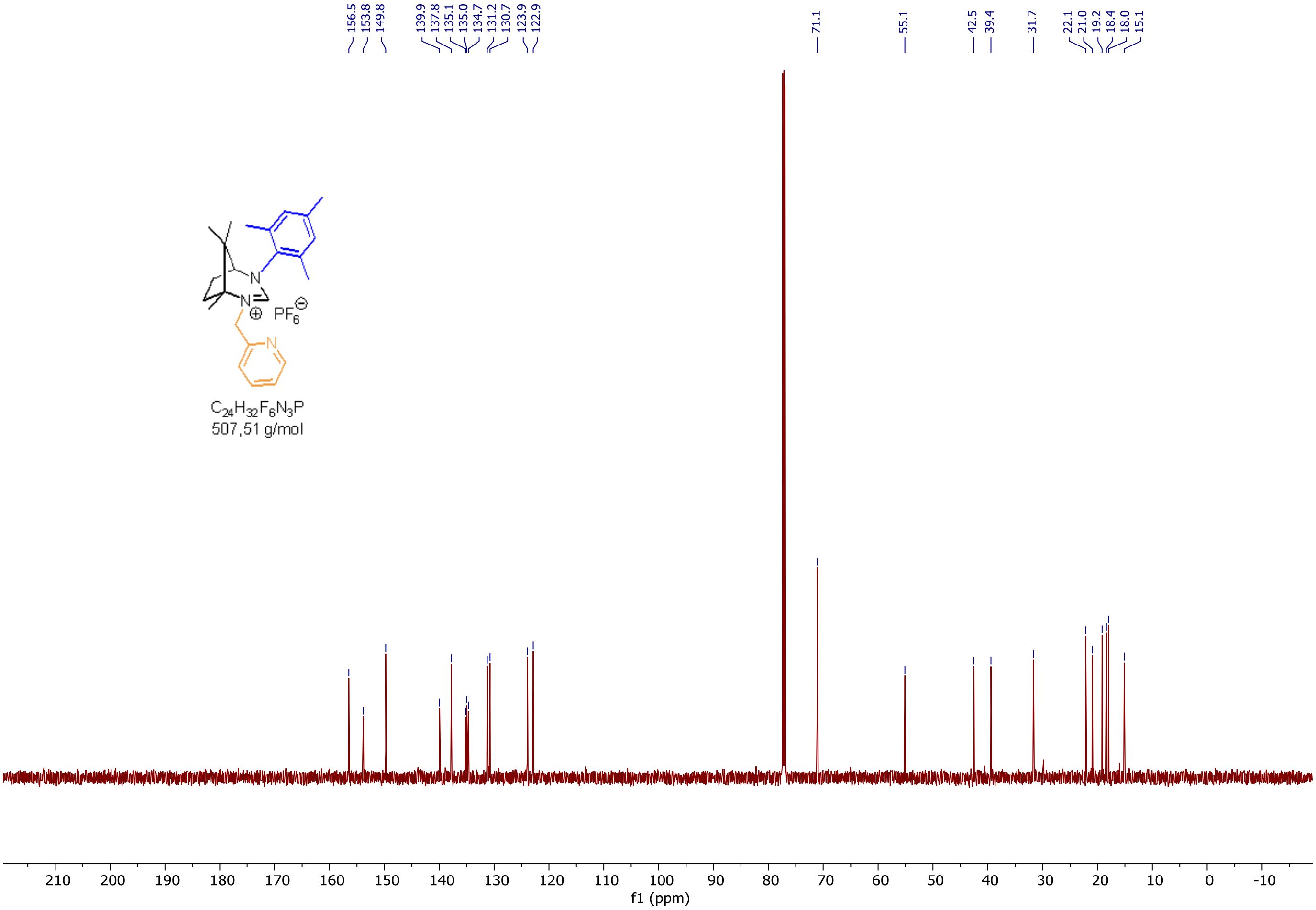

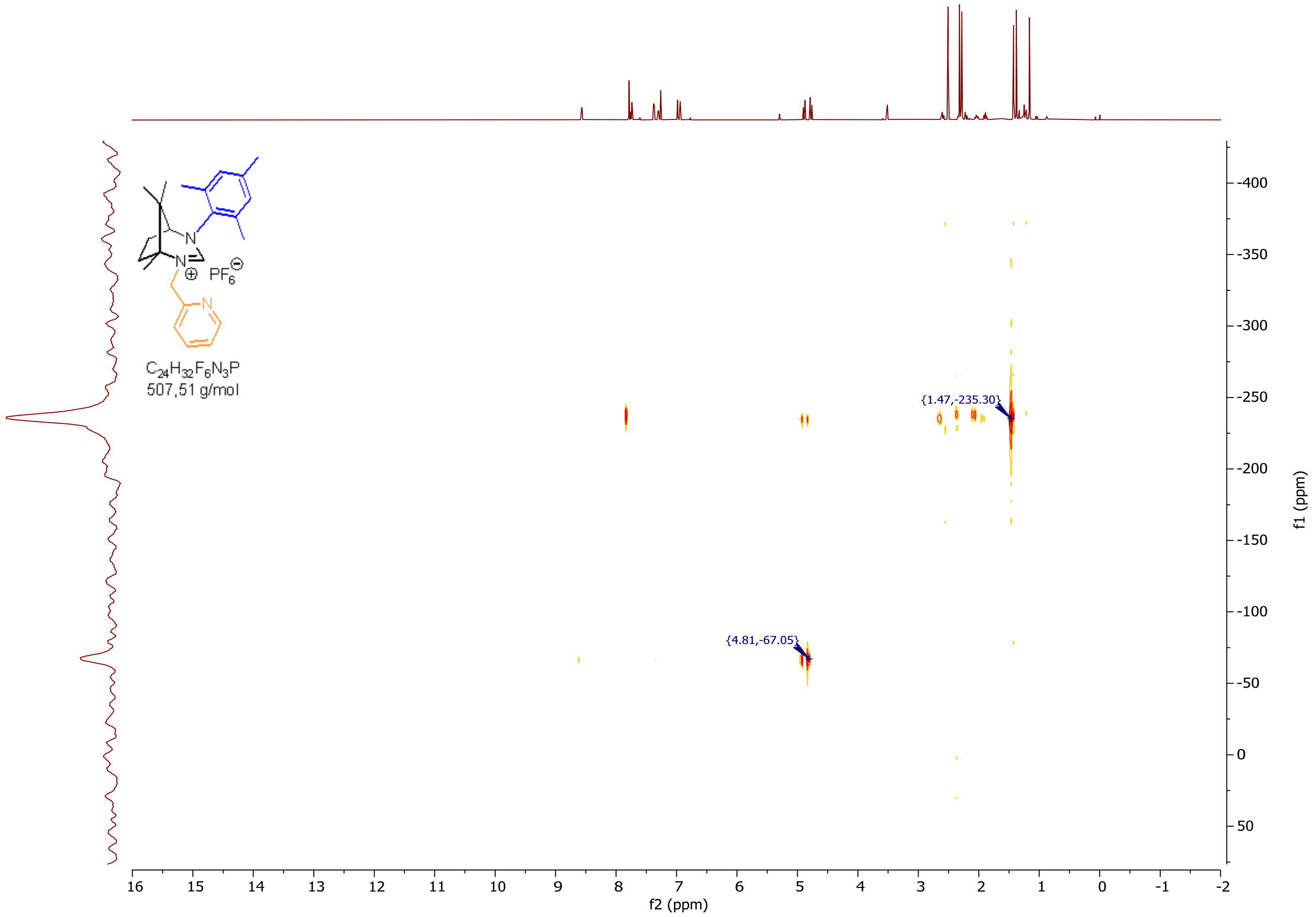

$C_{24}H_{32}F_6N_3P$
507,51 g/mol
{1.47,-235.30}
{4.81,-67.05}
f2 (ppm)
f1 (ppm)

^{1}H NMR (600 MHz, DMSO), ^{13}C{^{1}H} NMR (151 MHz, DMSO) and ^{15}N HSQC NMR (61 MHz, DMSO) Analysis of Compound **3bh** at 80 °C

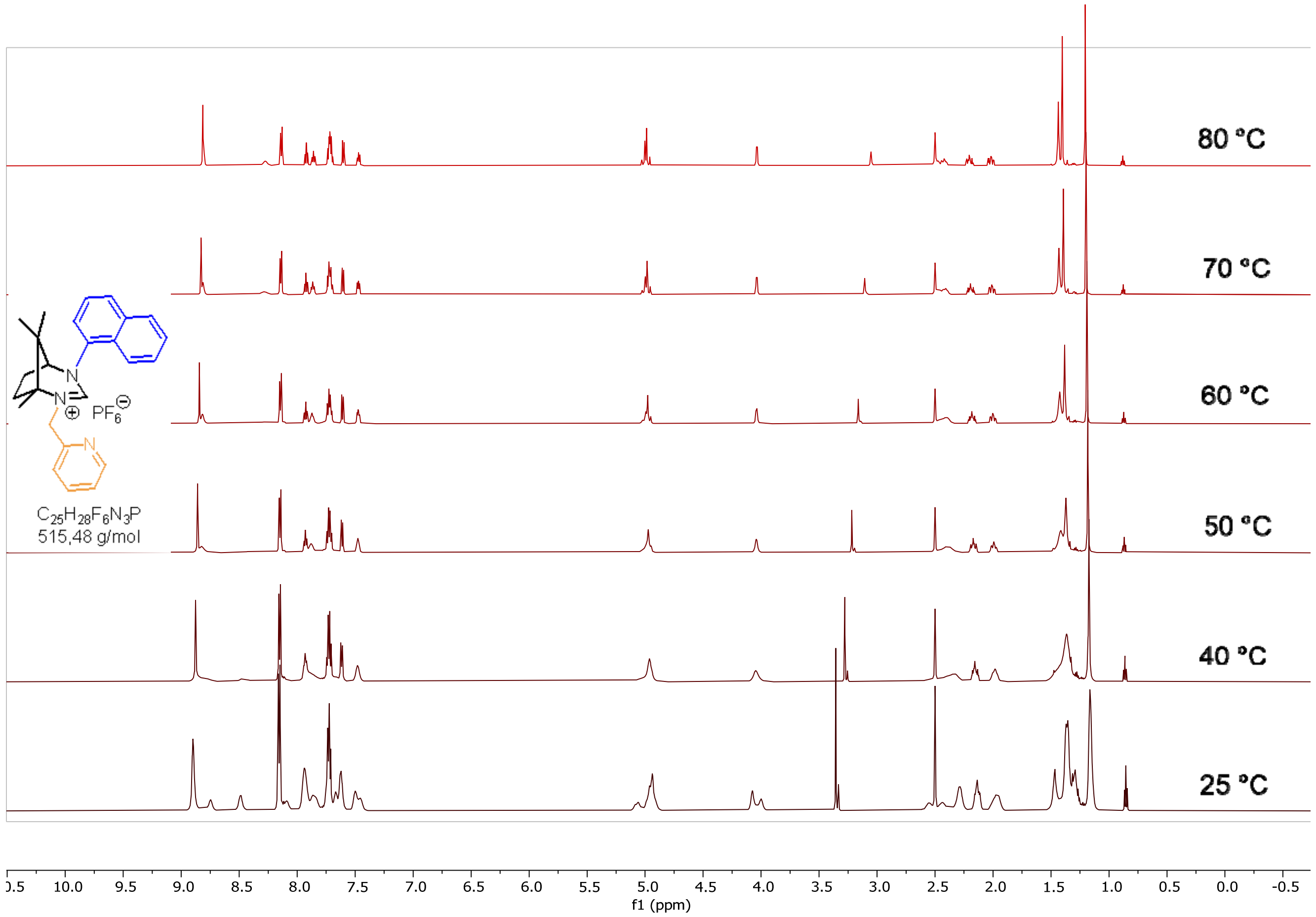

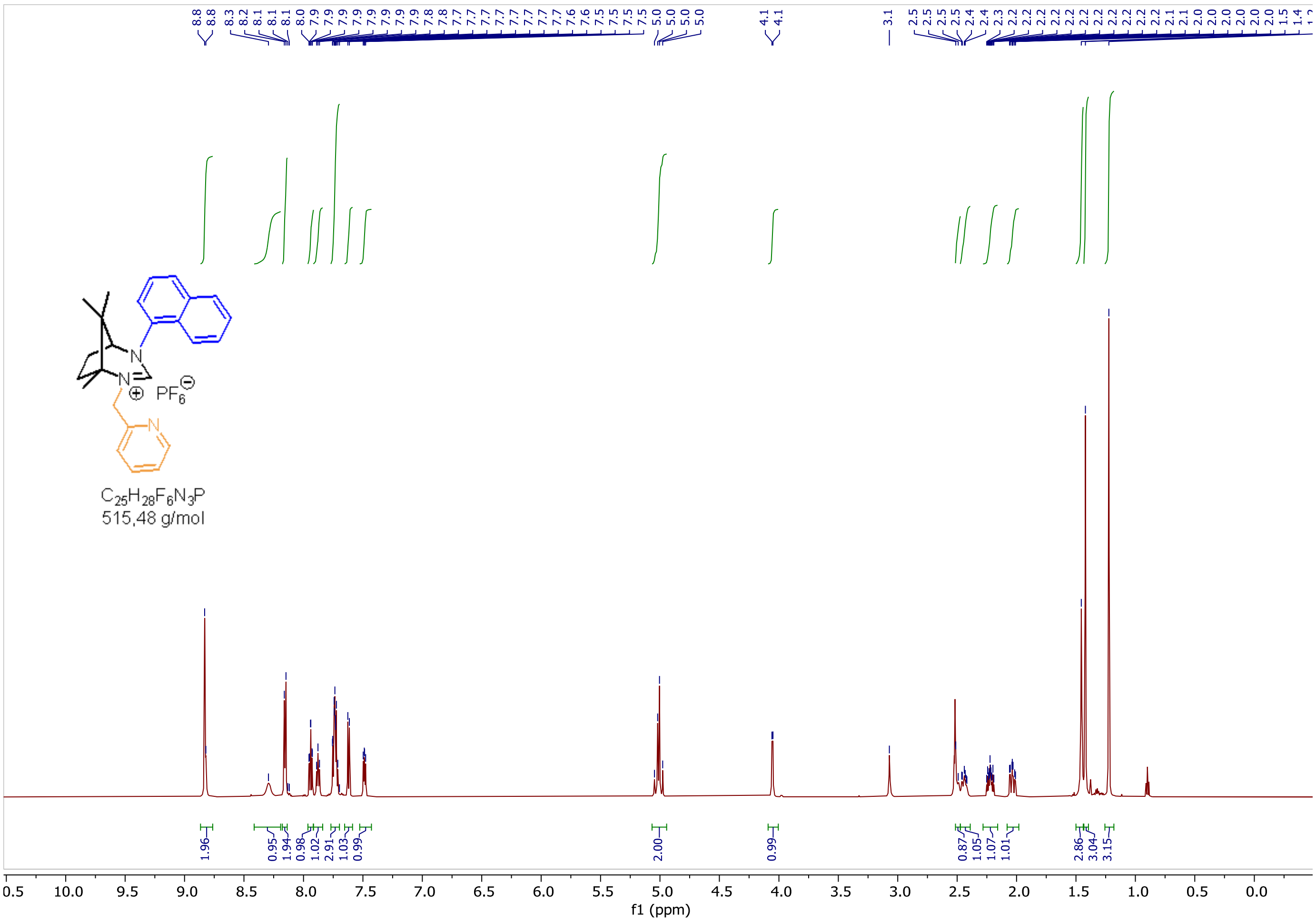

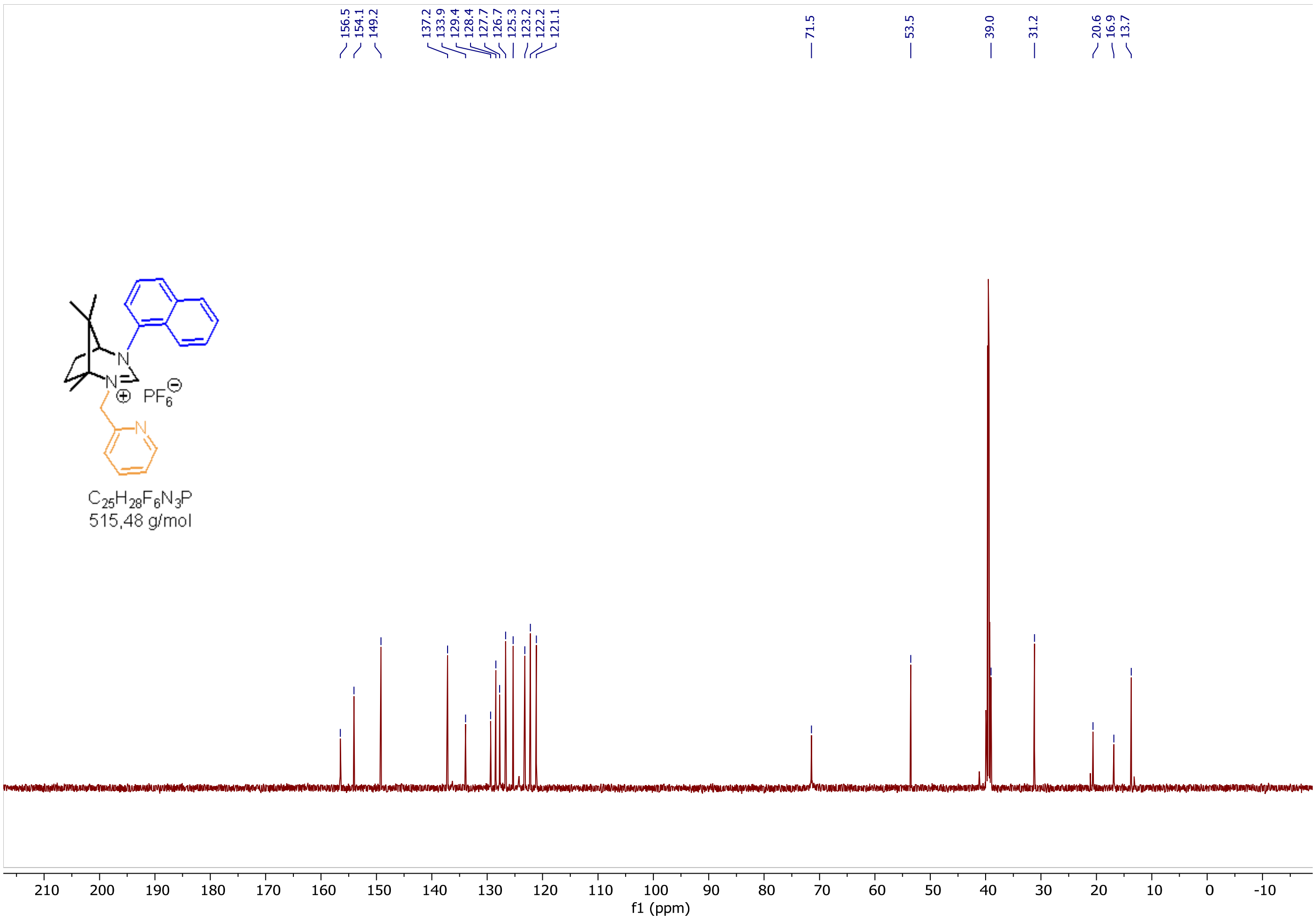
156.5
154.1
149.2
137.2
133.9
129.4
128.4
127.7
126.7
125.3
123.2
122.2
121.1
71.5
53.5
39.0
31.2
20.6
16.9
13.7
$C_{25}H_{28}F_6N_3P$
515,48 g/mol
f1 (ppm)

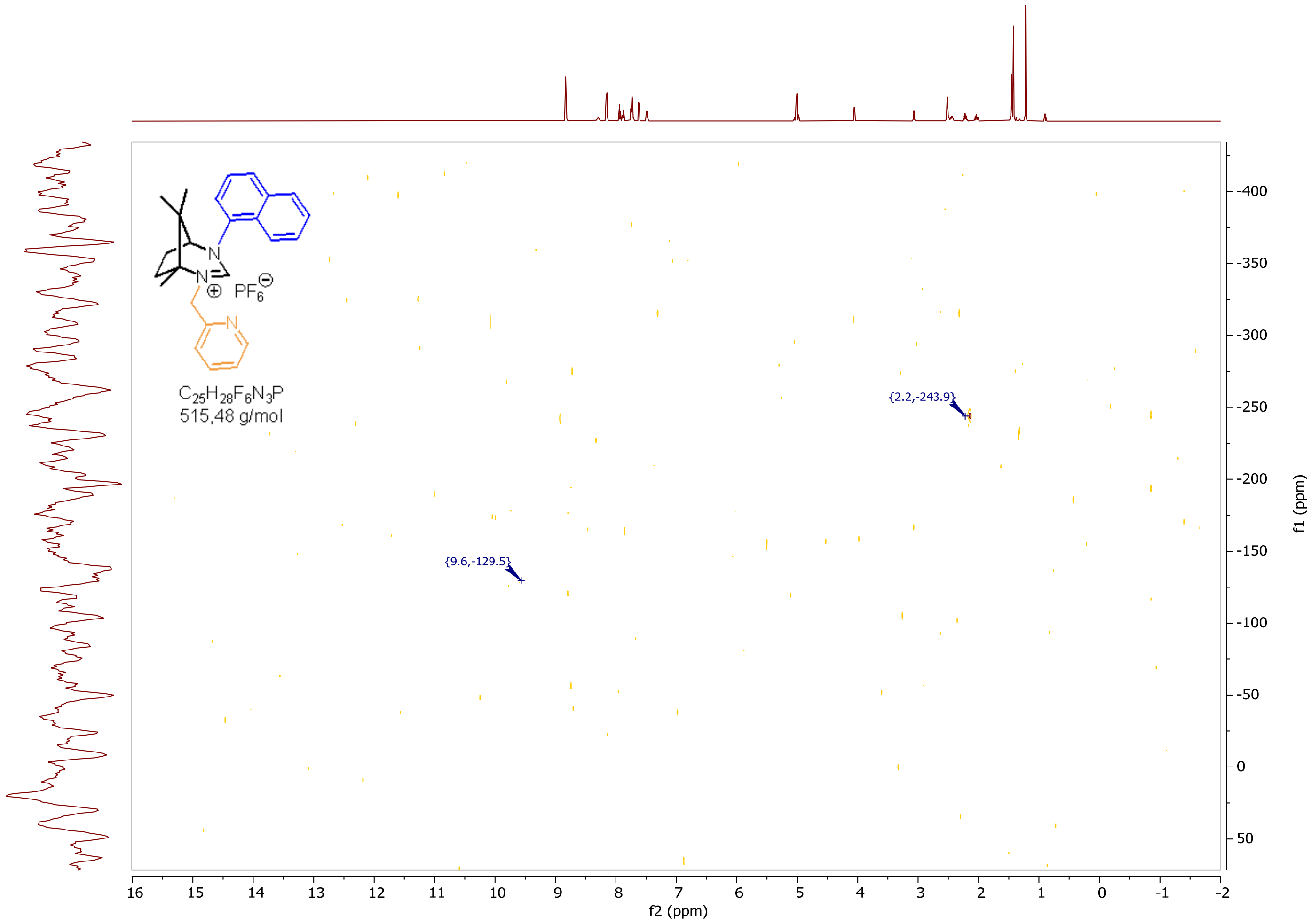
{2.2,-243.9}
{9.6,-129.5}
$C_{25}H_{28}F_6N_3P$
515,48 g/mol
f2 (ppm)
f1 (ppm)

^{1}H NMR (600 MHz, $CDCl_3$), ^{13}C{^{1}H} NMR (151 MHz, $CDCl_3$) and ^{15}N HSQC NMR (61 MHz, $CDCl_3$) Analysis of Compound **3bi**

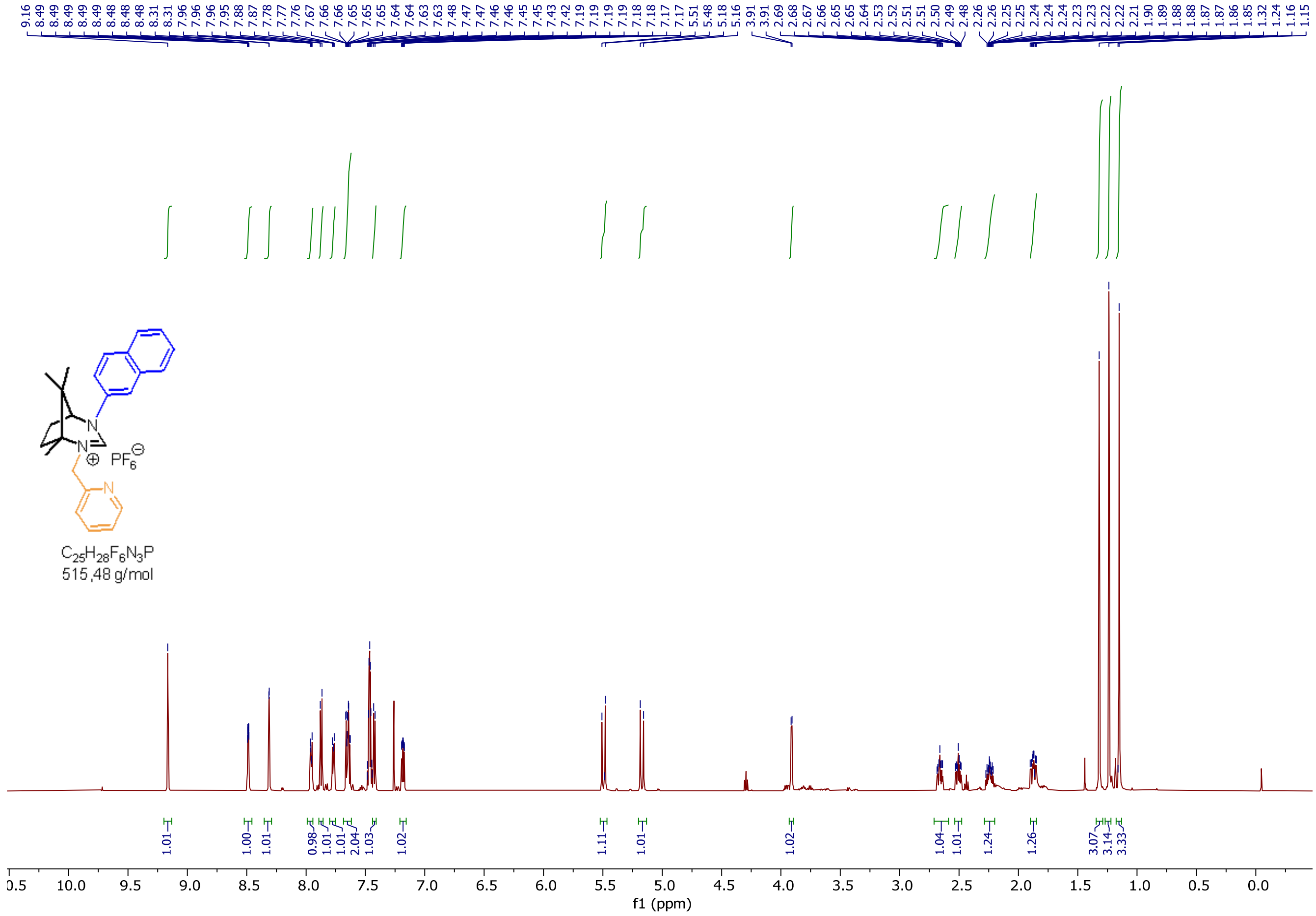

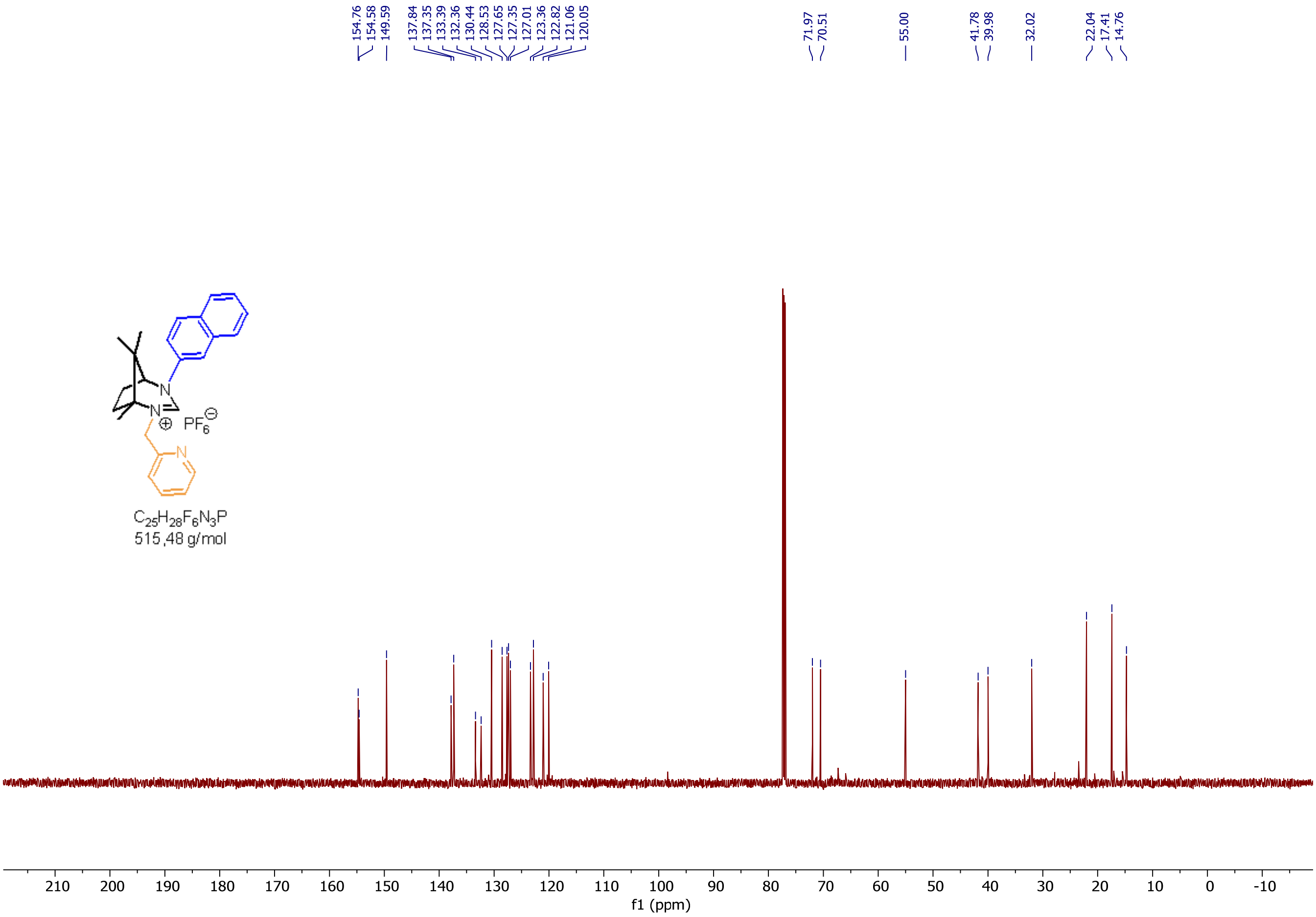

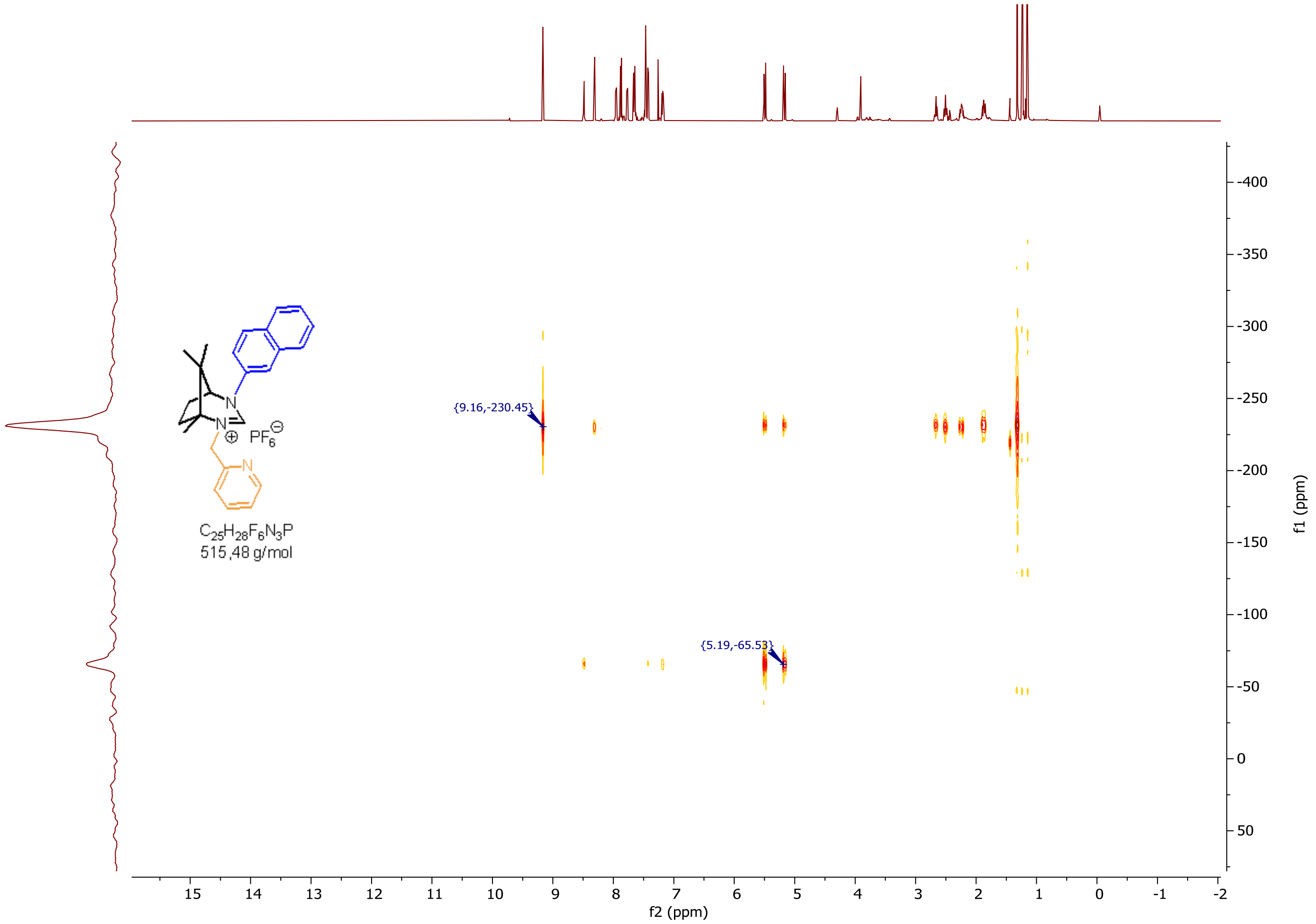

{9.16,-230.45}
{5.19,-65.53}
N
N
PF6
C25H28F6N3P
515.48 g/mol
f2 (ppm)
f1 (ppm)

^{1}H NMR (600 MHz, $CDCl_3$), ^{13}C{^{1}H} NMR (151 MHz, $CDCl_3$), ^{15}N HSQC NMR (61 MHz, $CDCl_3$) and ^{19}F NMR (337 MHz, $CDCl_3$) Analysis of Compound **3bj**

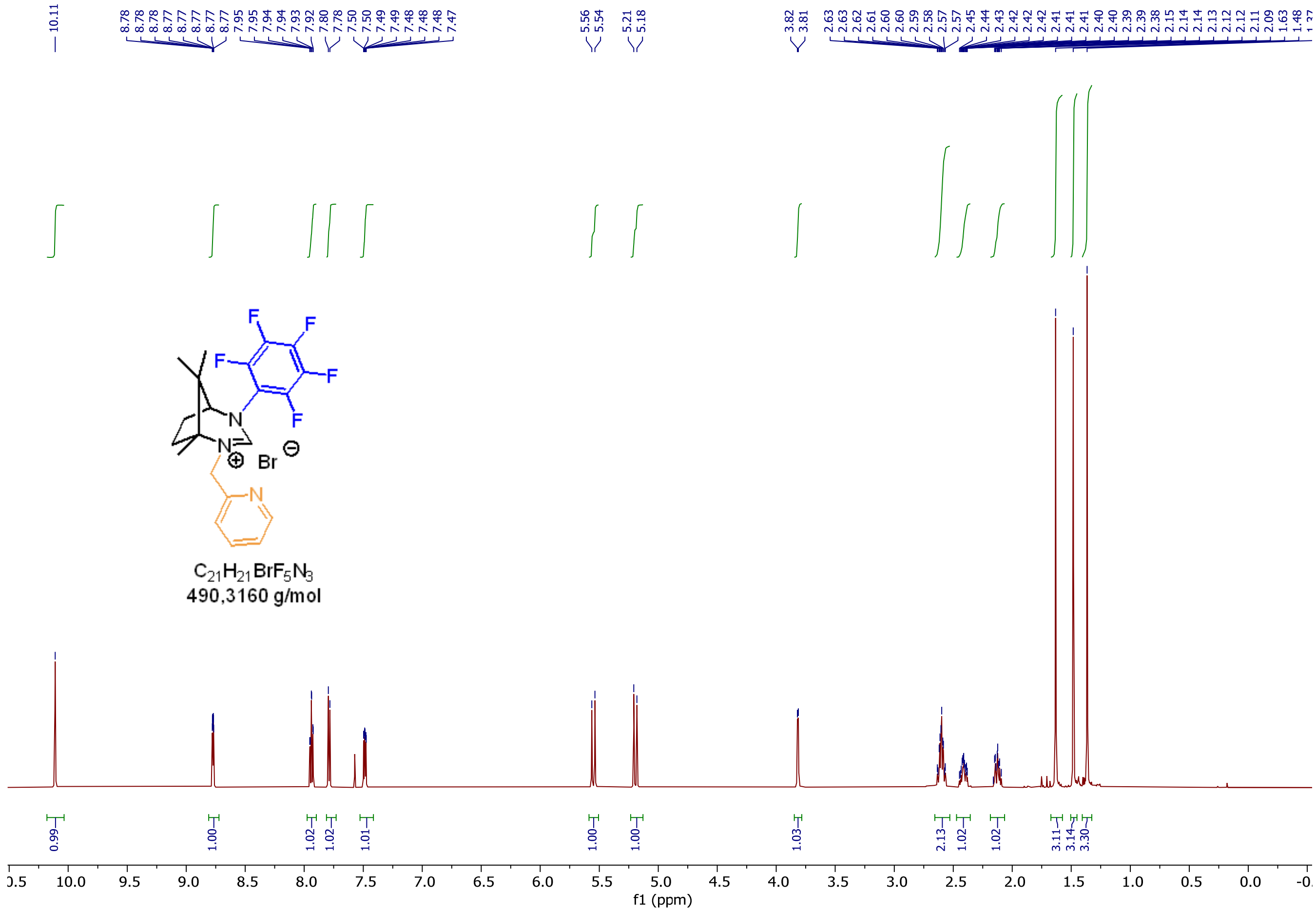

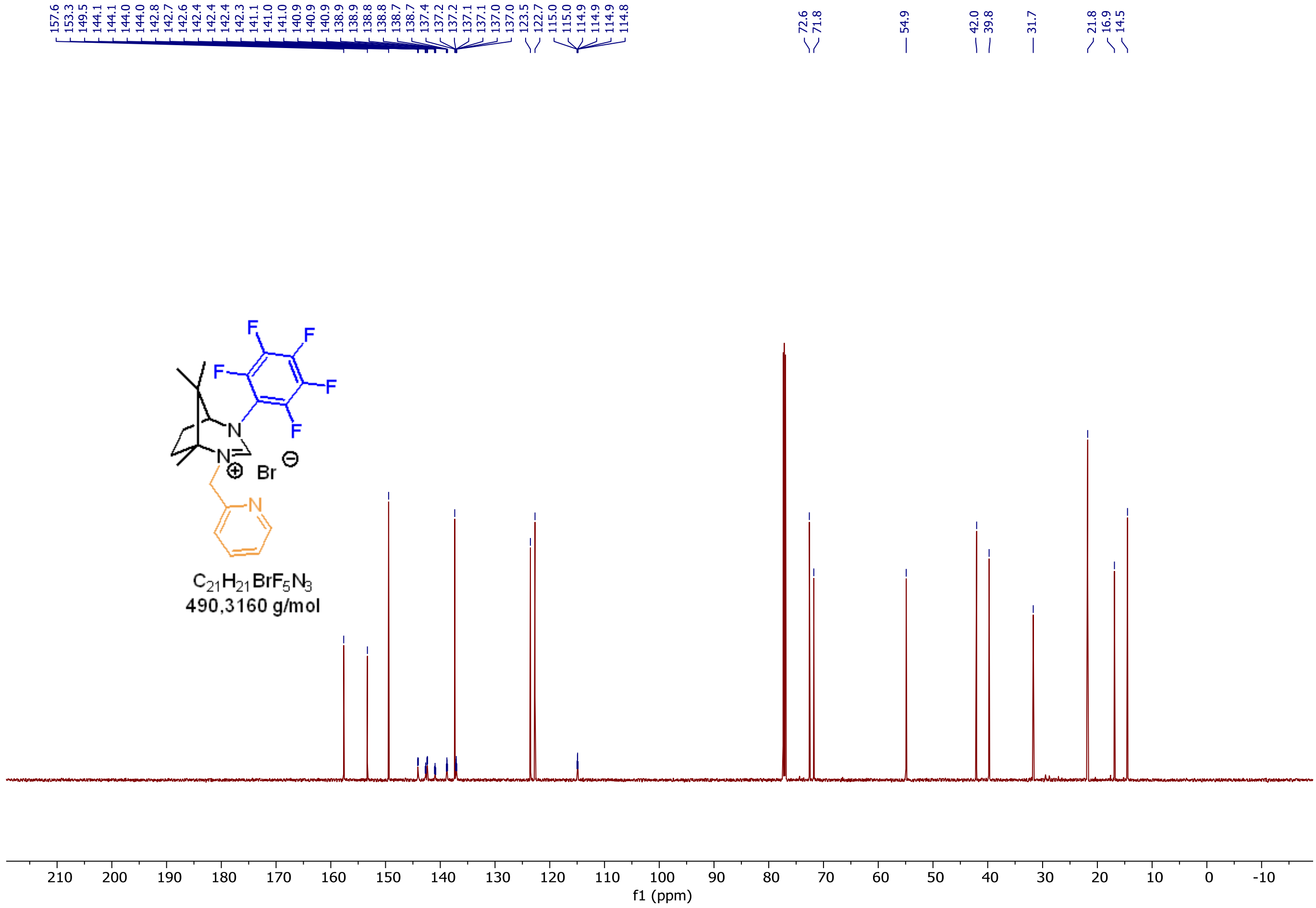

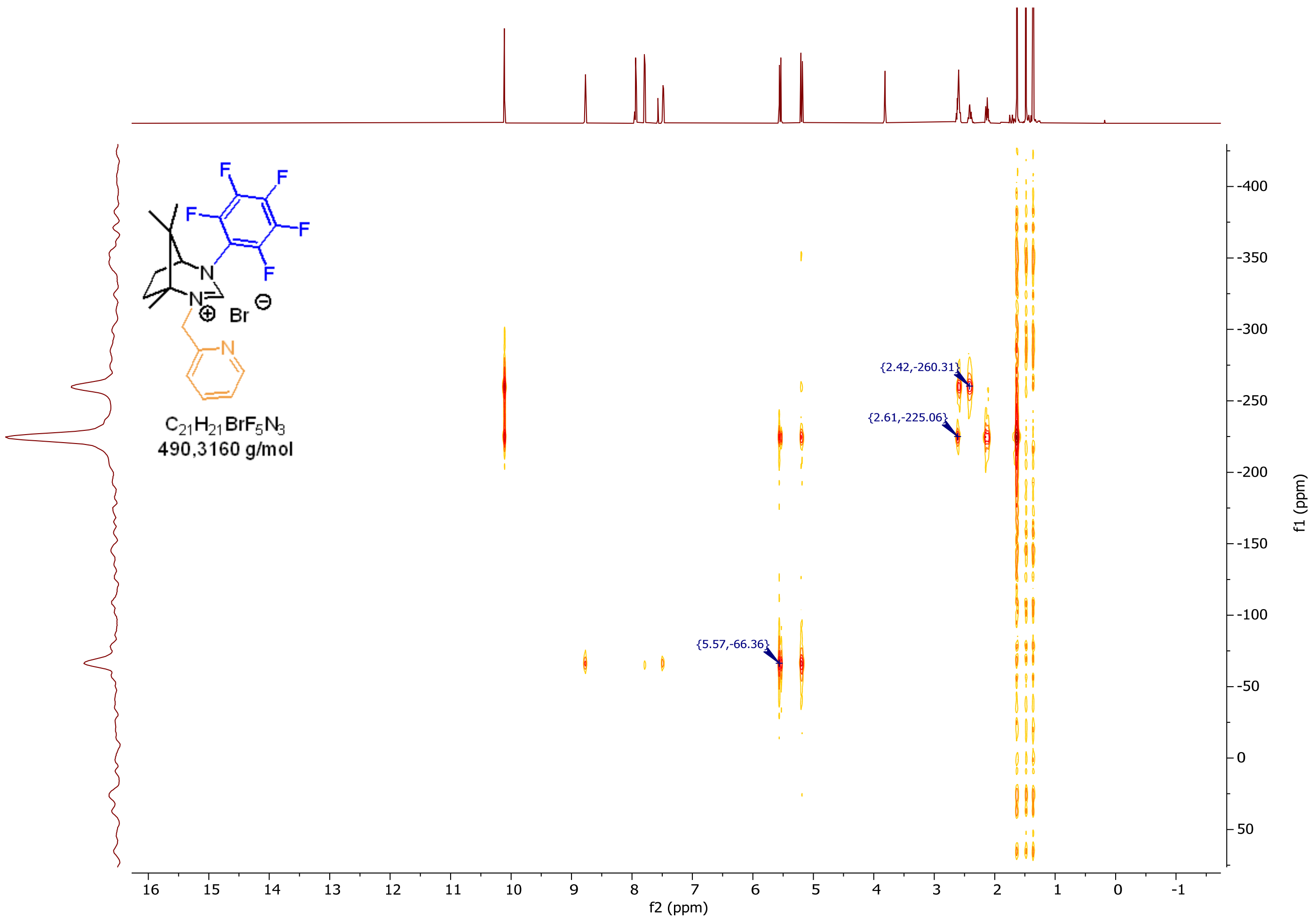

C21H21BrF5N3
490,3160 g/mol
{2.42,-260.31}
{2.61,-225.06}
{5.57,-66.36}
f2 (ppm)
f1 (ppm)

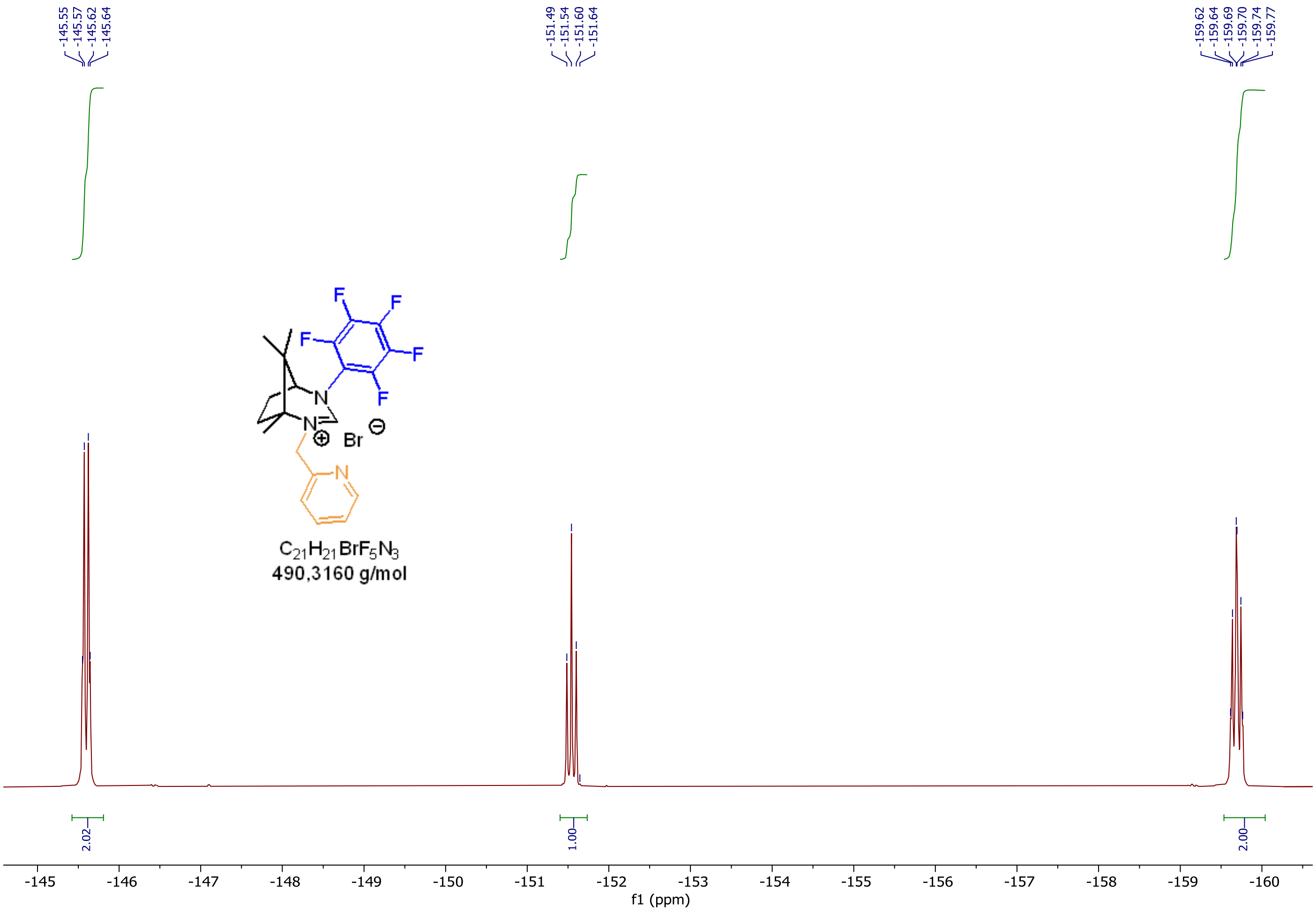

-145.55
-145.57
-145.62
-145.64
-151.49
-151.54
-151.60
-151.64
-159.62
-159.64
-159.69
-159.70
-159.74
-159.77
C21H21BrF5N3
490,3160 g/mol
2.02
1.00
2.00
f1 (ppm)

^{1}H NMR (600 MHz, $CDCl_3$), ^{13}C{^{1}H} NMR (151 MHz, $CDCl_3$), ^{15}N HSQC NMR (61 MHz, $CDCl_3$) and ^{19}F NMR (337 MHz, $CDCl_3$) Analysis of Compound **3bk**

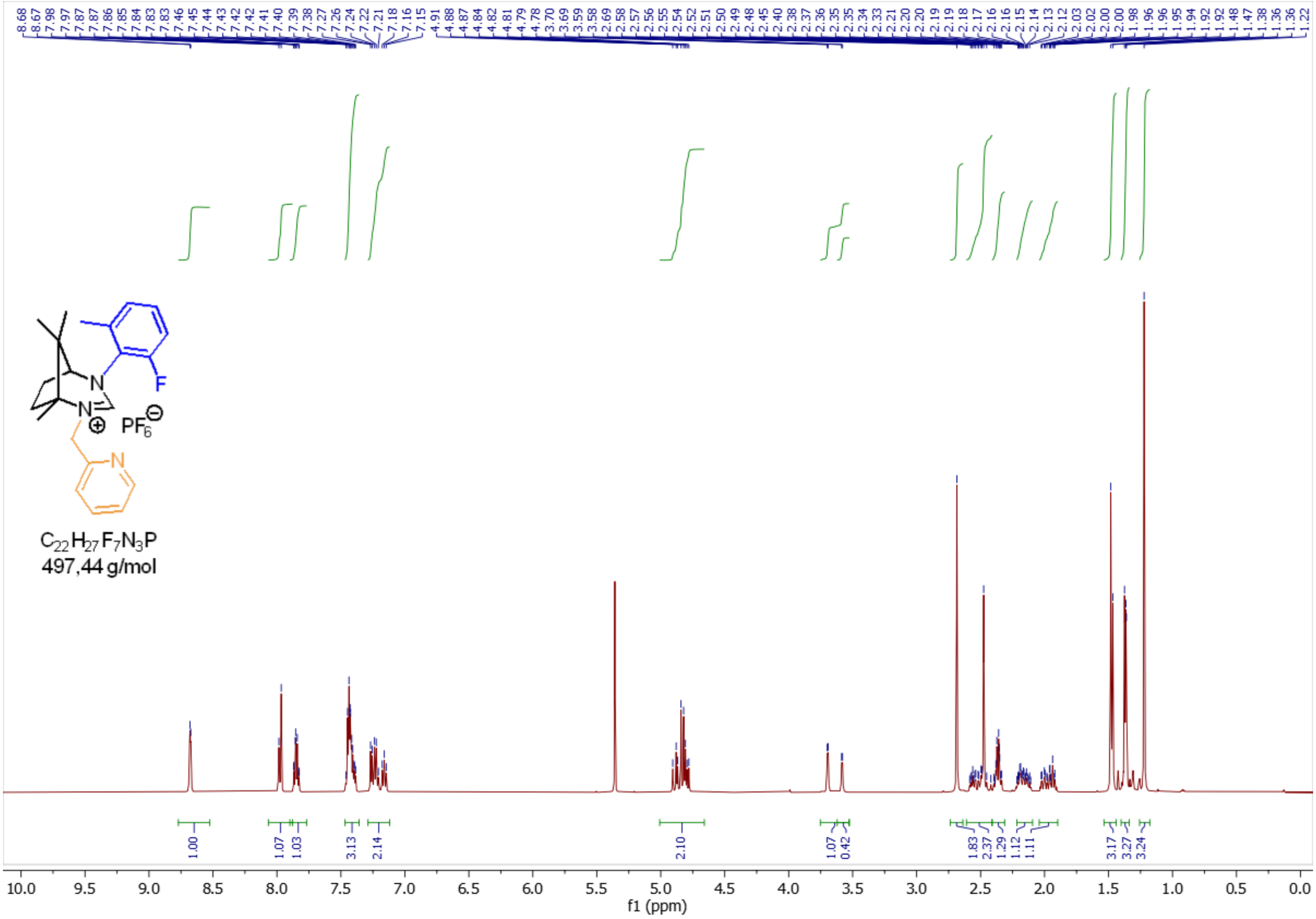

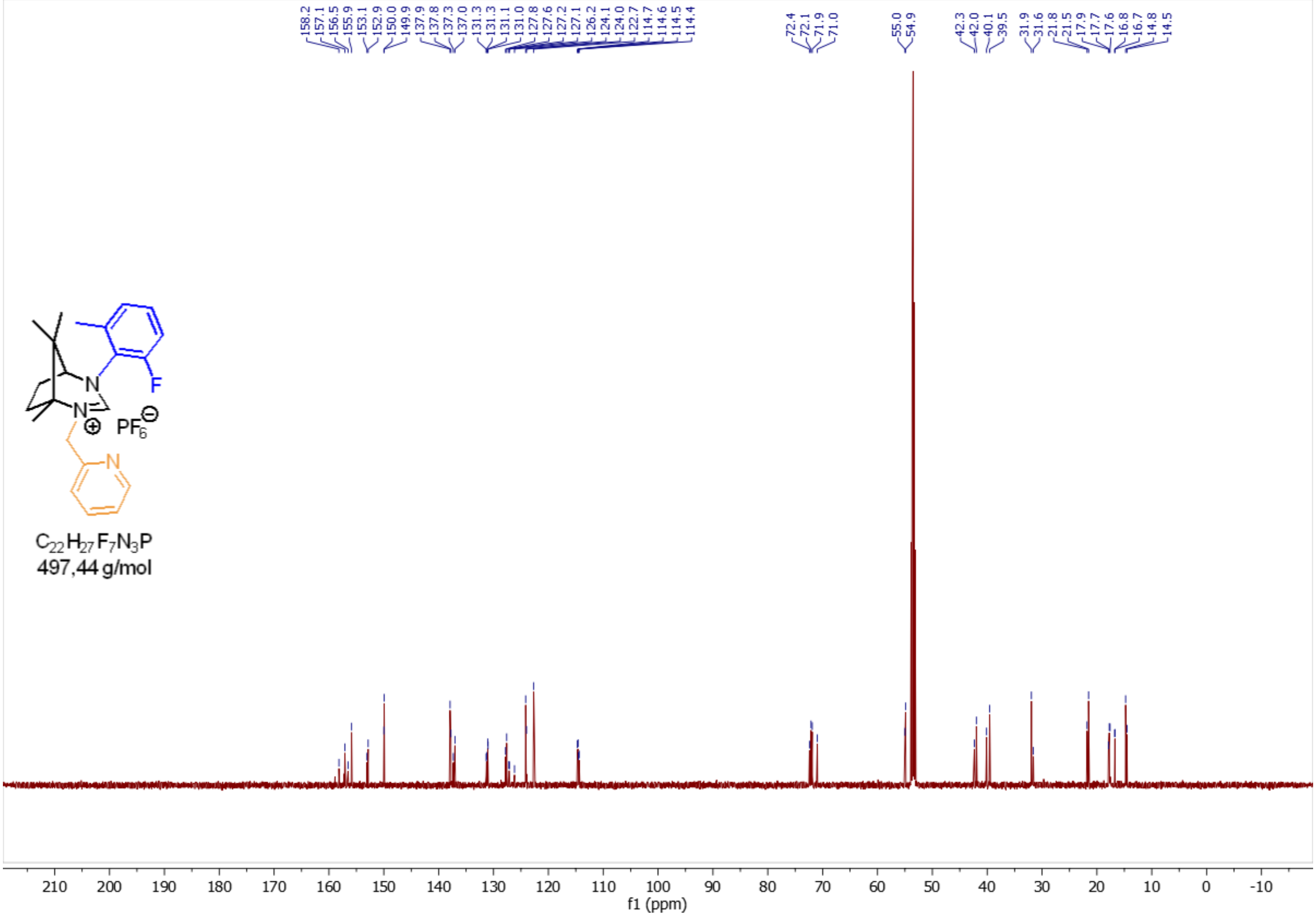

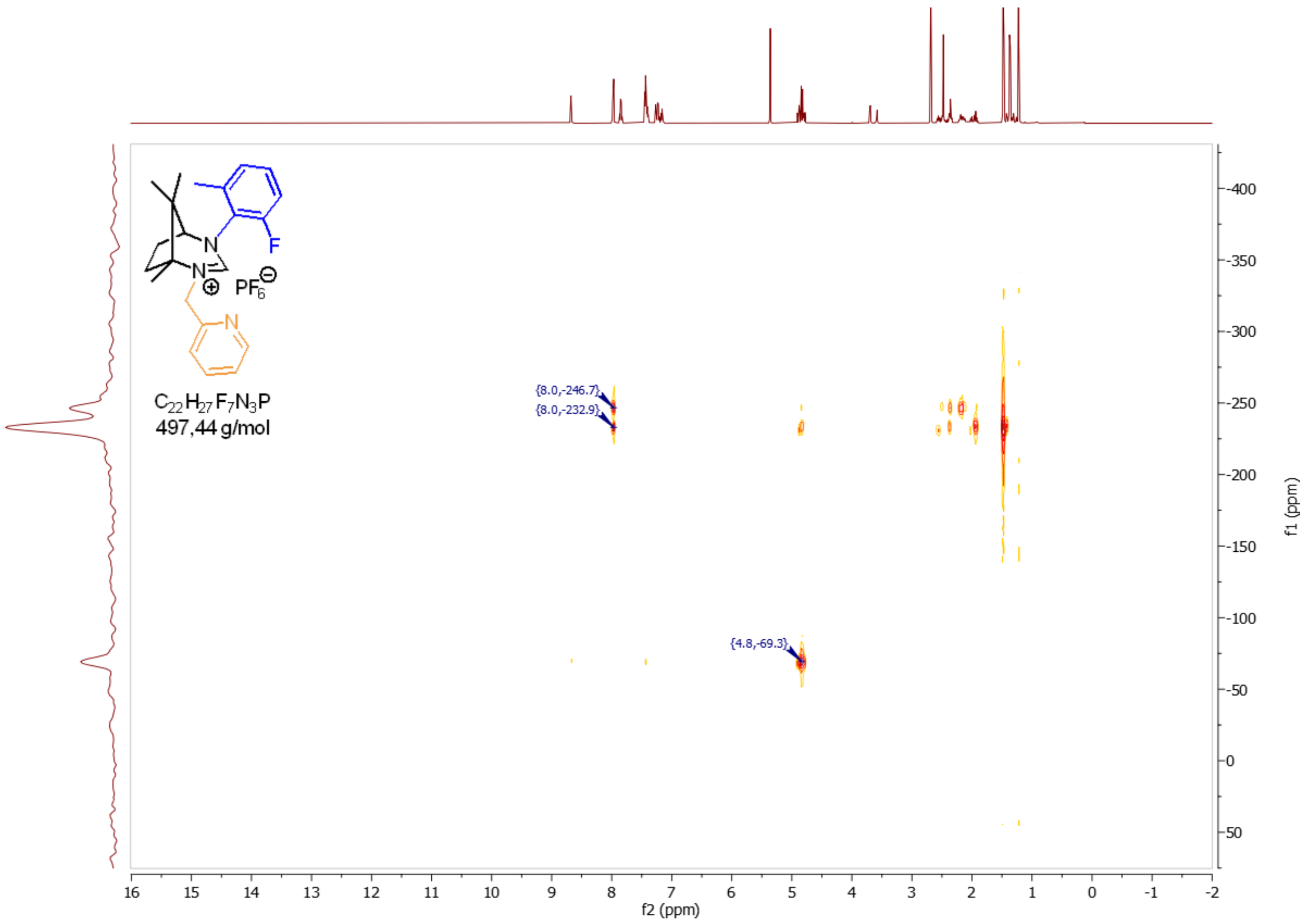
N
F
N
PF6
N
C22H27F7N3P
497,44 g/mol
{8.0,-246.7}
{8.0,-232.9}
{4.8,-69.3}
f2 (ppm)
f1 (ppm)

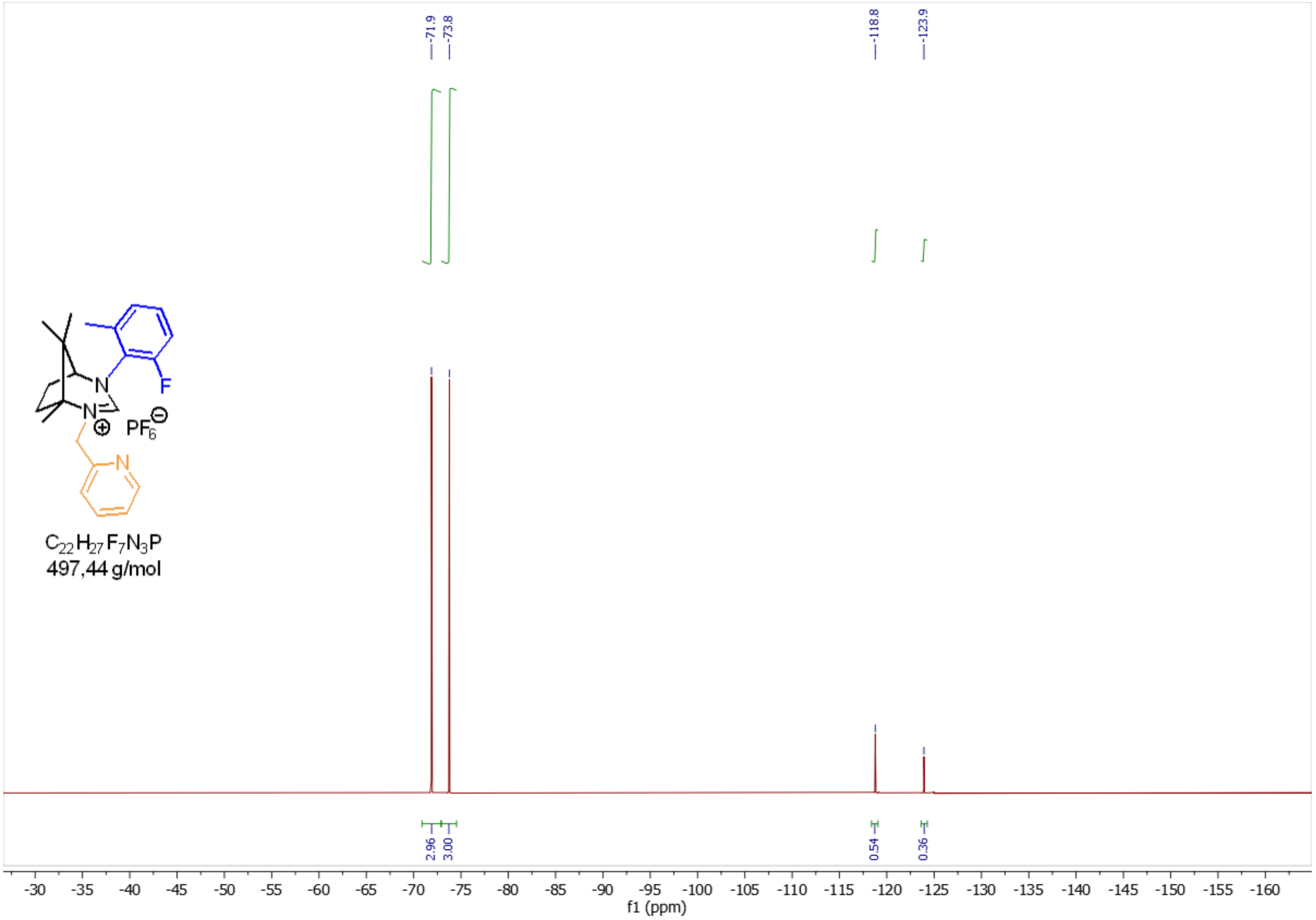
-71.9
-73.8
-118.8
-123.9
N
F
N
PF6
N
C22H27F7N3P
497,44 g/mol
2.96
3.00
0.54
0.36
f1 (ppm)

^{1}H NMR (600 MHz, $CDCl_3$), $^{13}C\{^{1}H\}$ NMR (151 MHz, $CDCl_3$) and ^{15}N HSQC NMR (61 MHz, $CDCl_3$) Analysis of Compound **3bl**

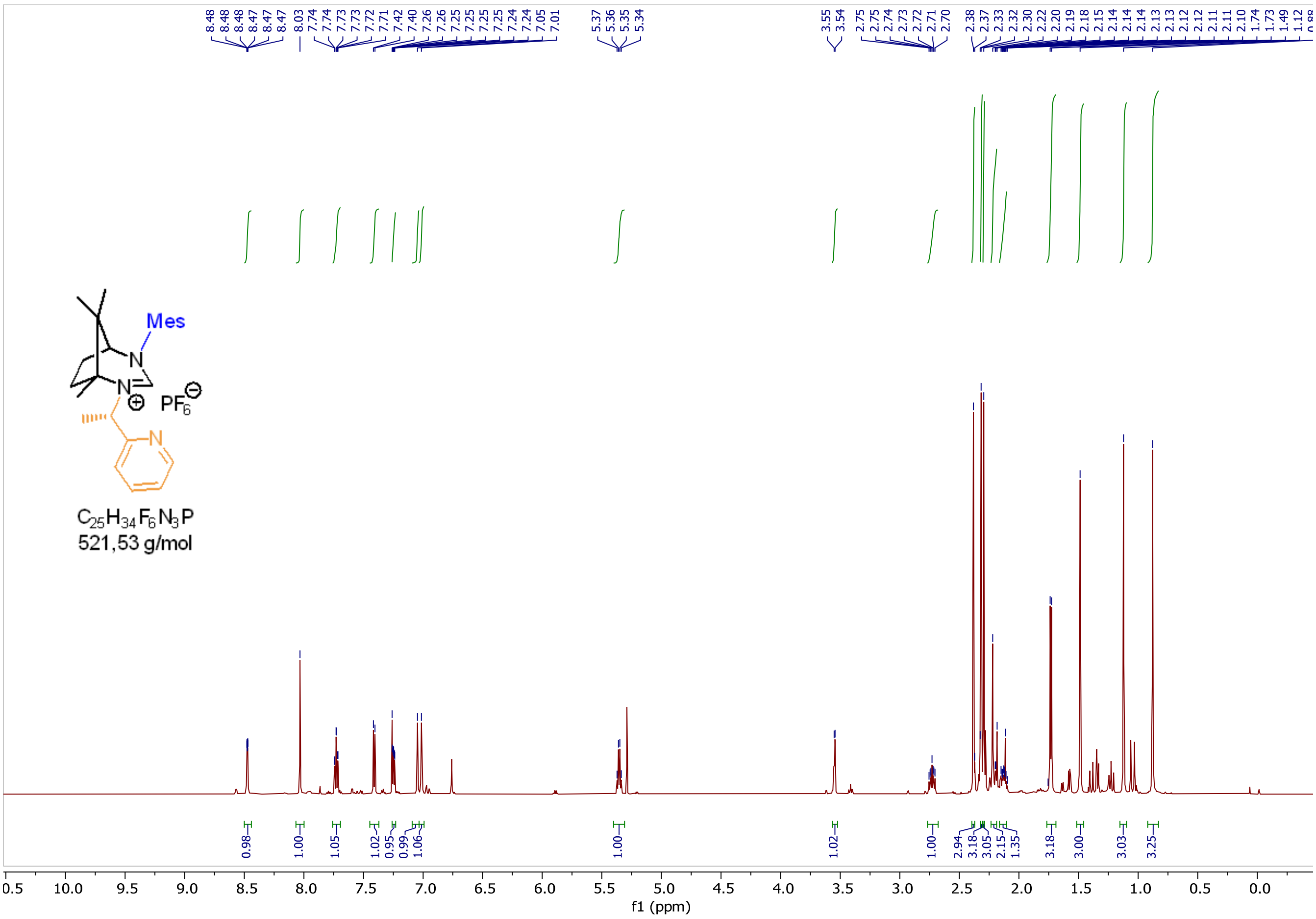

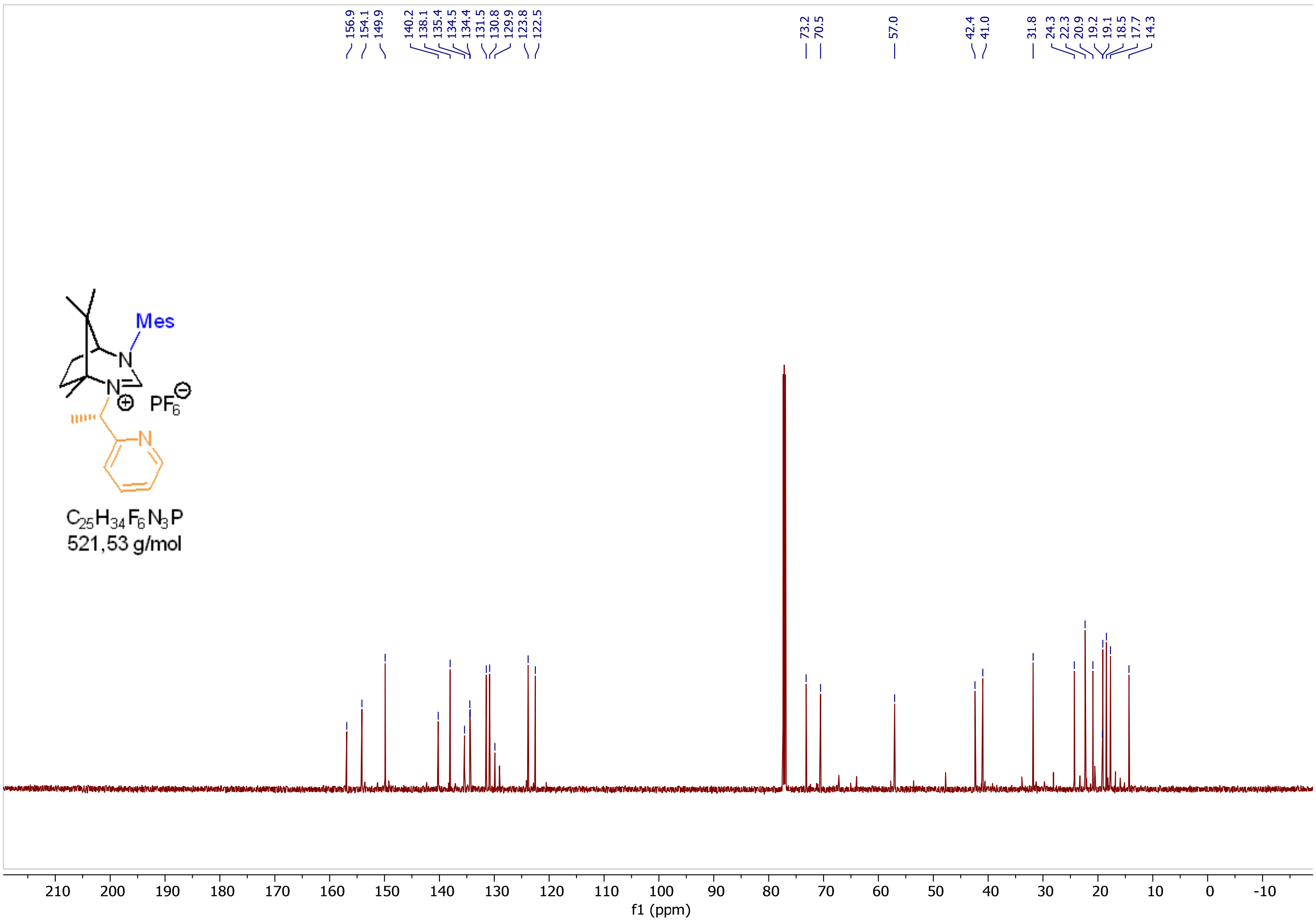

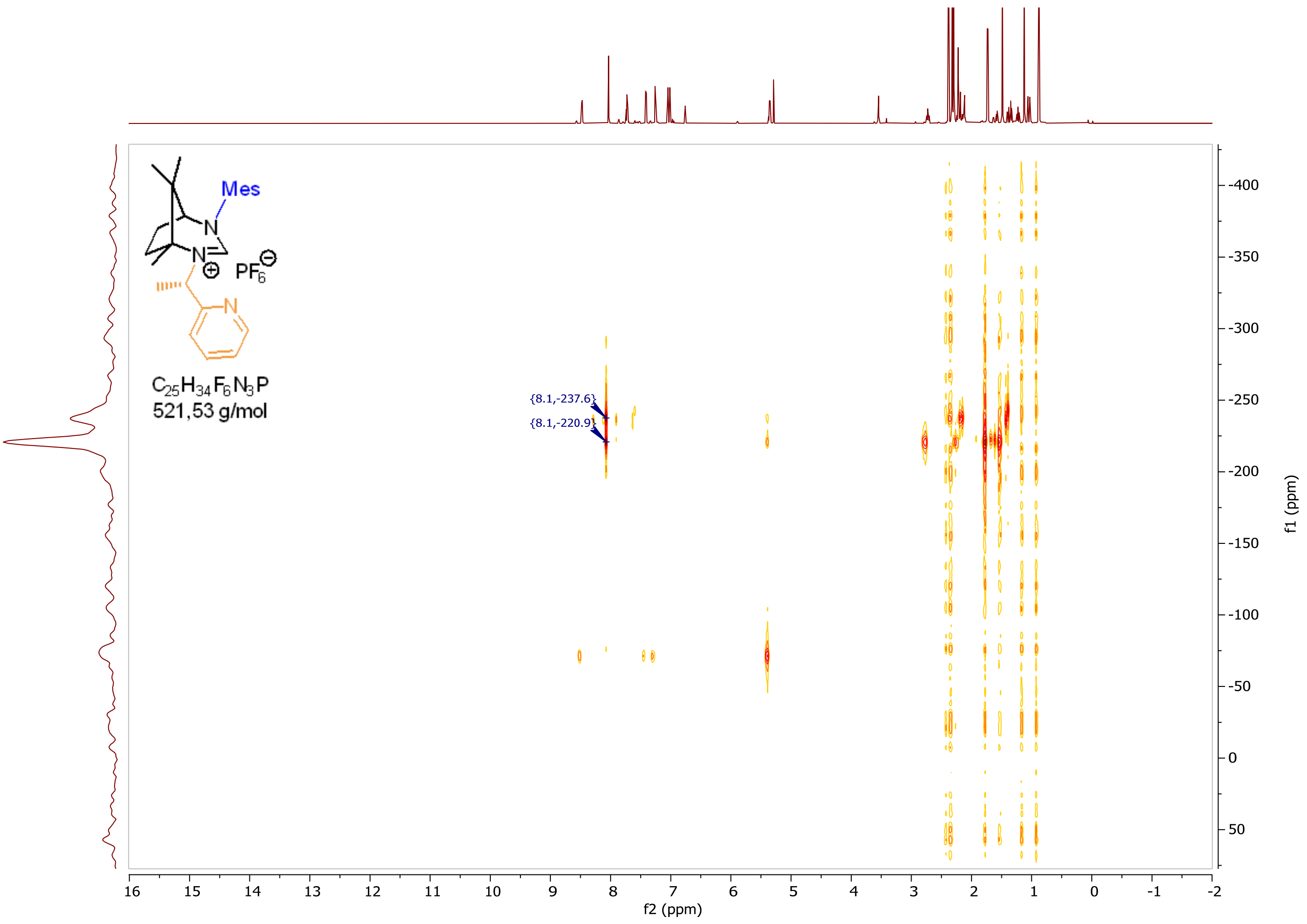
Mes
PF6
C25H34F6N3P
521,53 g/mol
{8.1,-237.6}
{8.1,-220.9}
f2 (ppm)
f1 (ppm)

^{1}H NMR (600 MHz, $CDCl_3$), $^{13}C\{^{1}H\}$ NMR (151 MHz, $CDCl_3$) and ^{15}N HSQC NMR (61 MHz, $CDCl_3$) Analysis of Compound **3bm**

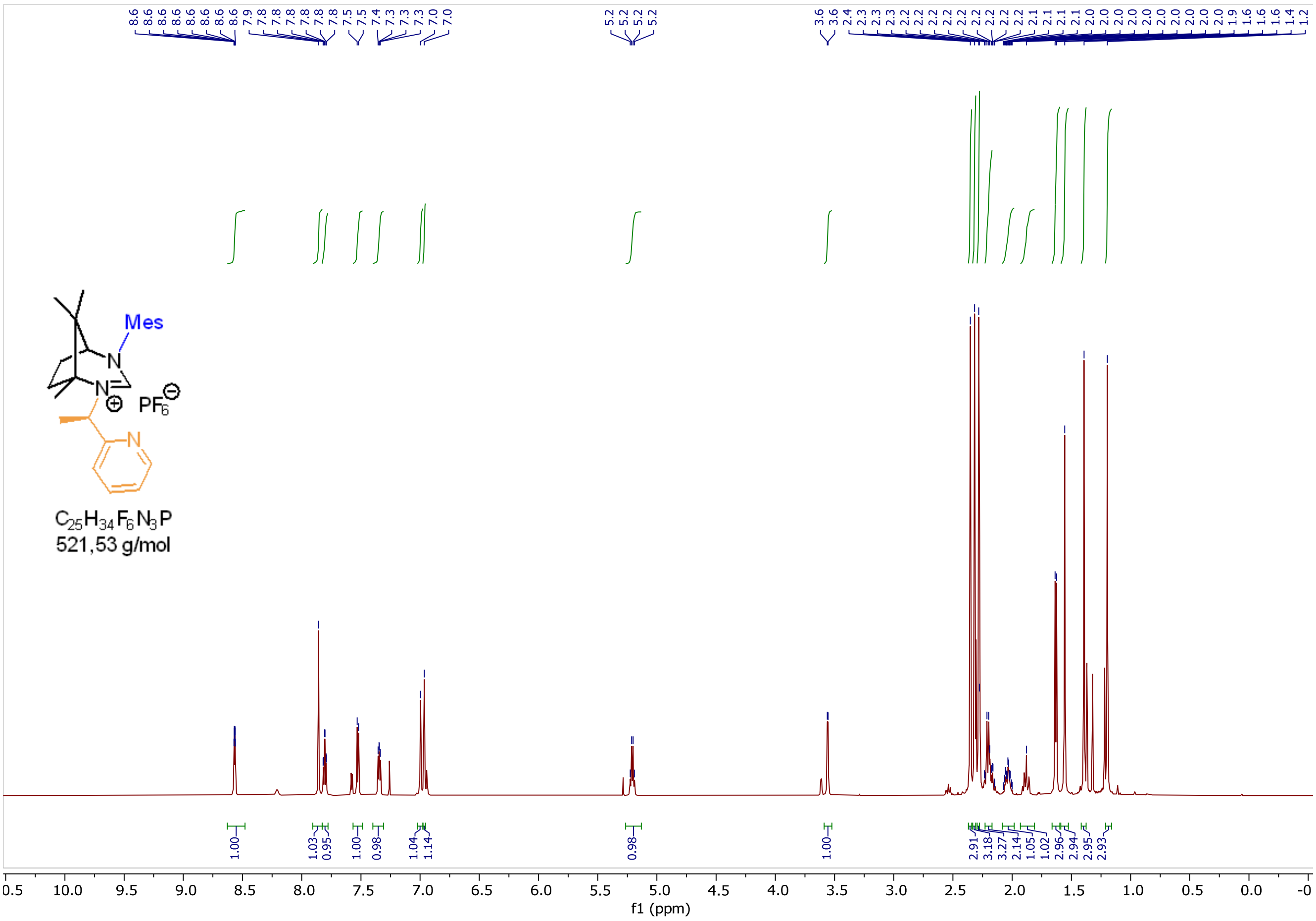

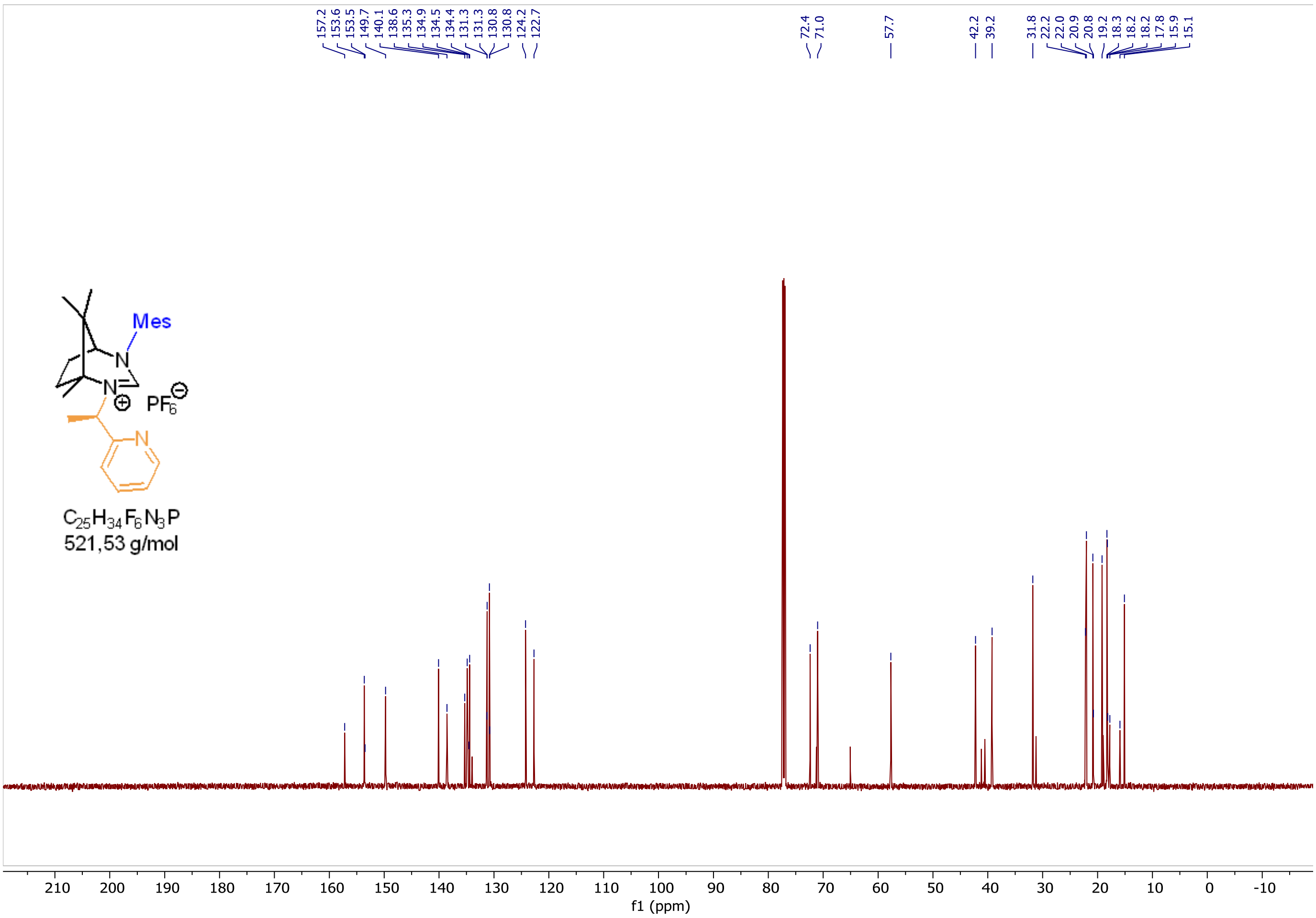

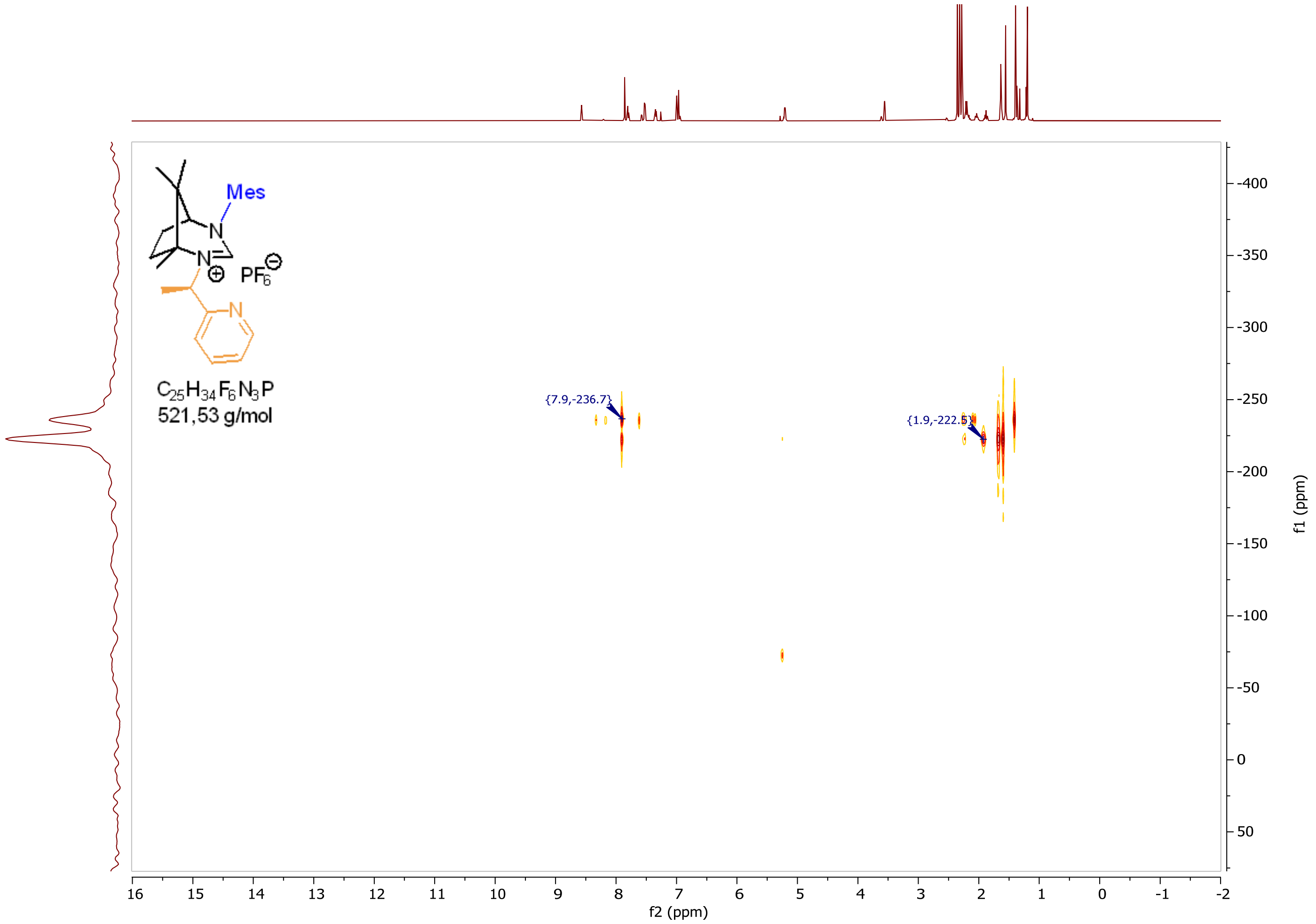
Mes
N
N
PF6
N
C25H34F6N3P
521,53 g/mol
{7.9,-236.7}
{1.9,-222.5}
f2 (ppm)
f1 (ppm)

^{1}H NMR (600 MHz, CD_2Cl_2), ^{13}C{^{1}H} NMR (151 MHz, CD_2Cl_2) and ^{15}N HSQC NMR (61 MHz, CD_2Cl_2) Analysis of Compound **3bn**

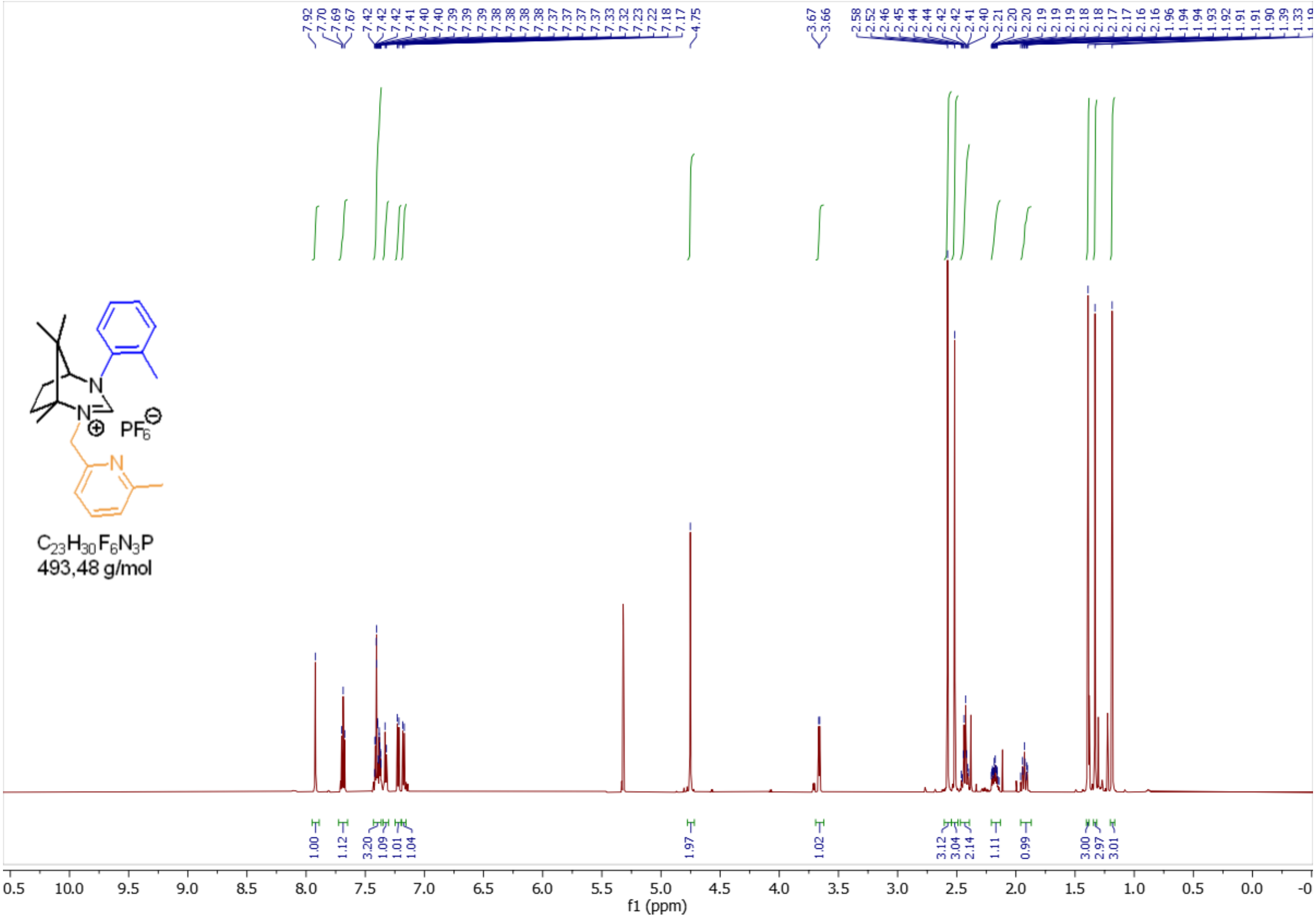

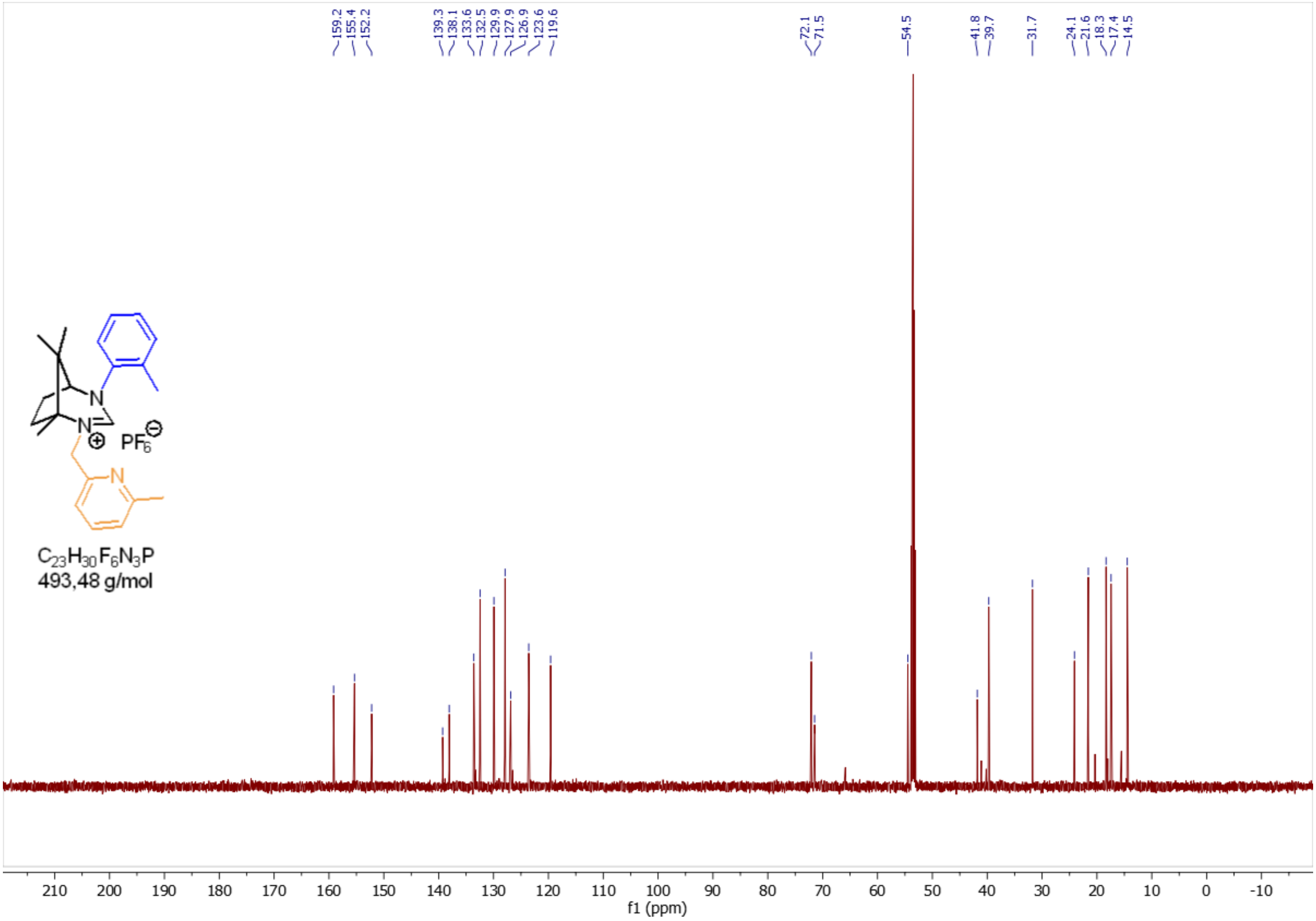

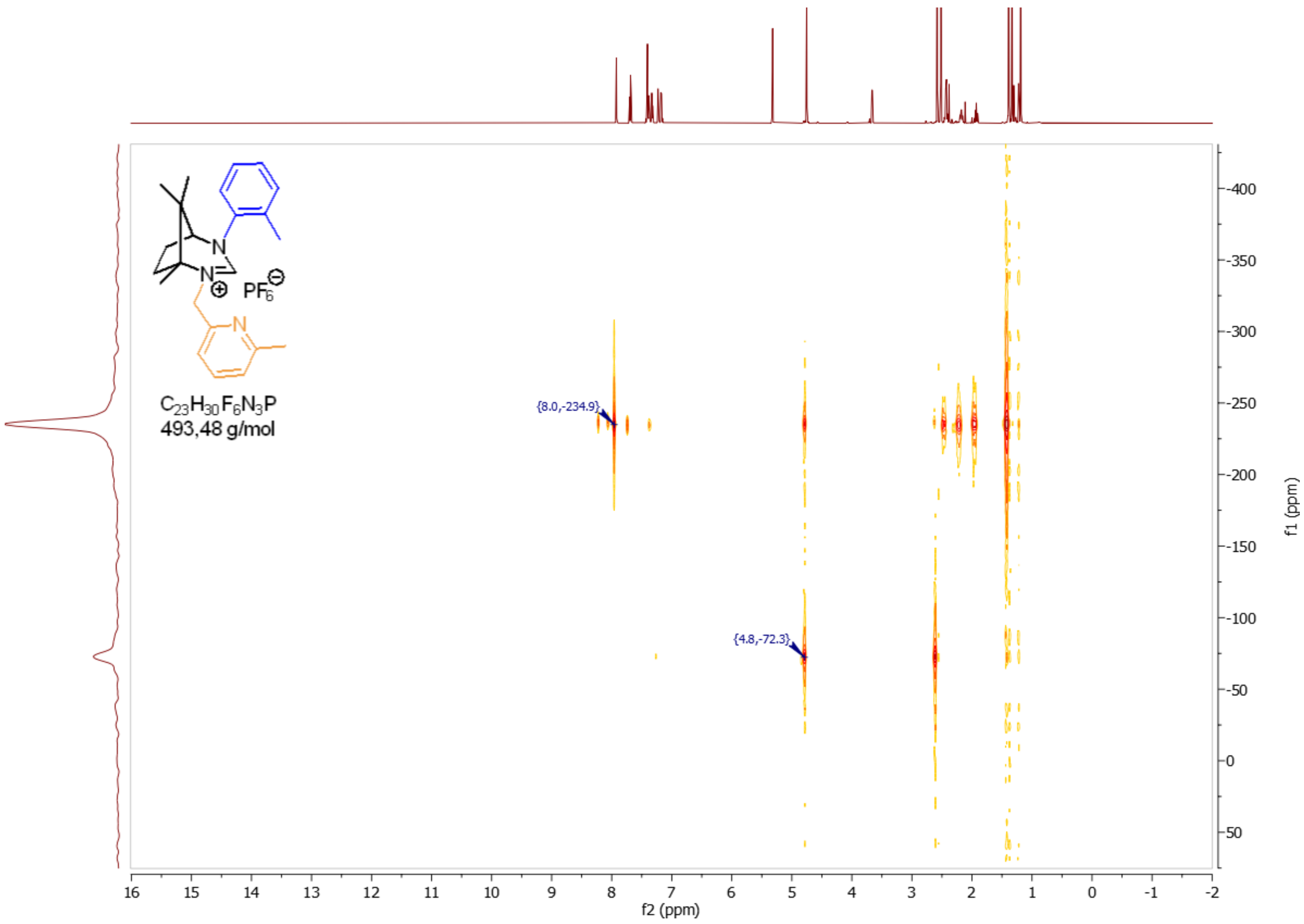

N
N
PF6
N
C23H30F6N3P
493,48 g/mol
{8.0,-234.9}
{4.8,-72.3}
f2 (ppm)
f1 (ppm)

^{1}H NMR (600 MHz, $CDCl_3$), $^{13}C\{^{1}H\}$ NMR (151 MHz, $CDCl_3$) and ^{15}N HSQC NMR (61 MHz, $CDCl_3$) Analysis of Compound **3bo**

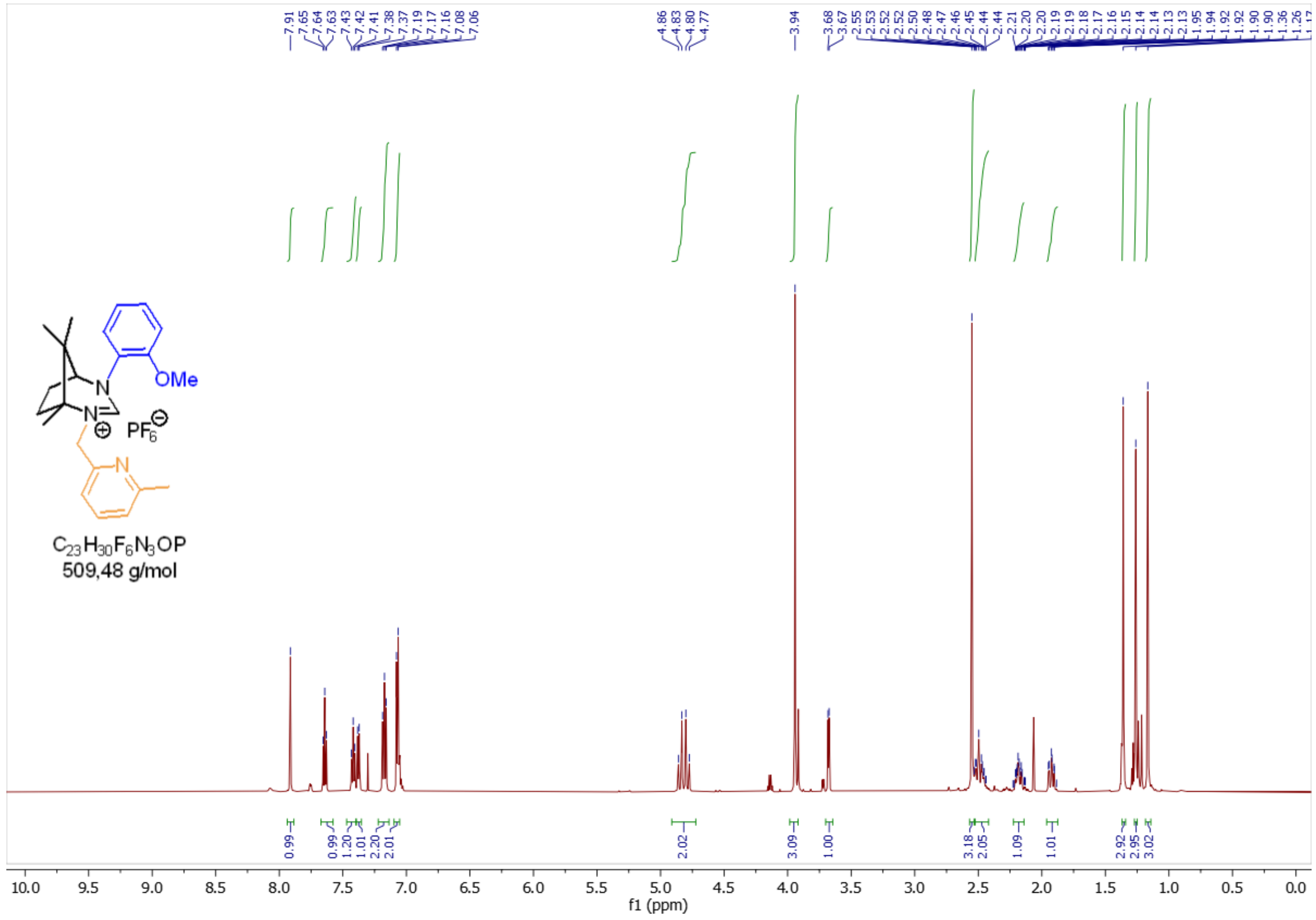

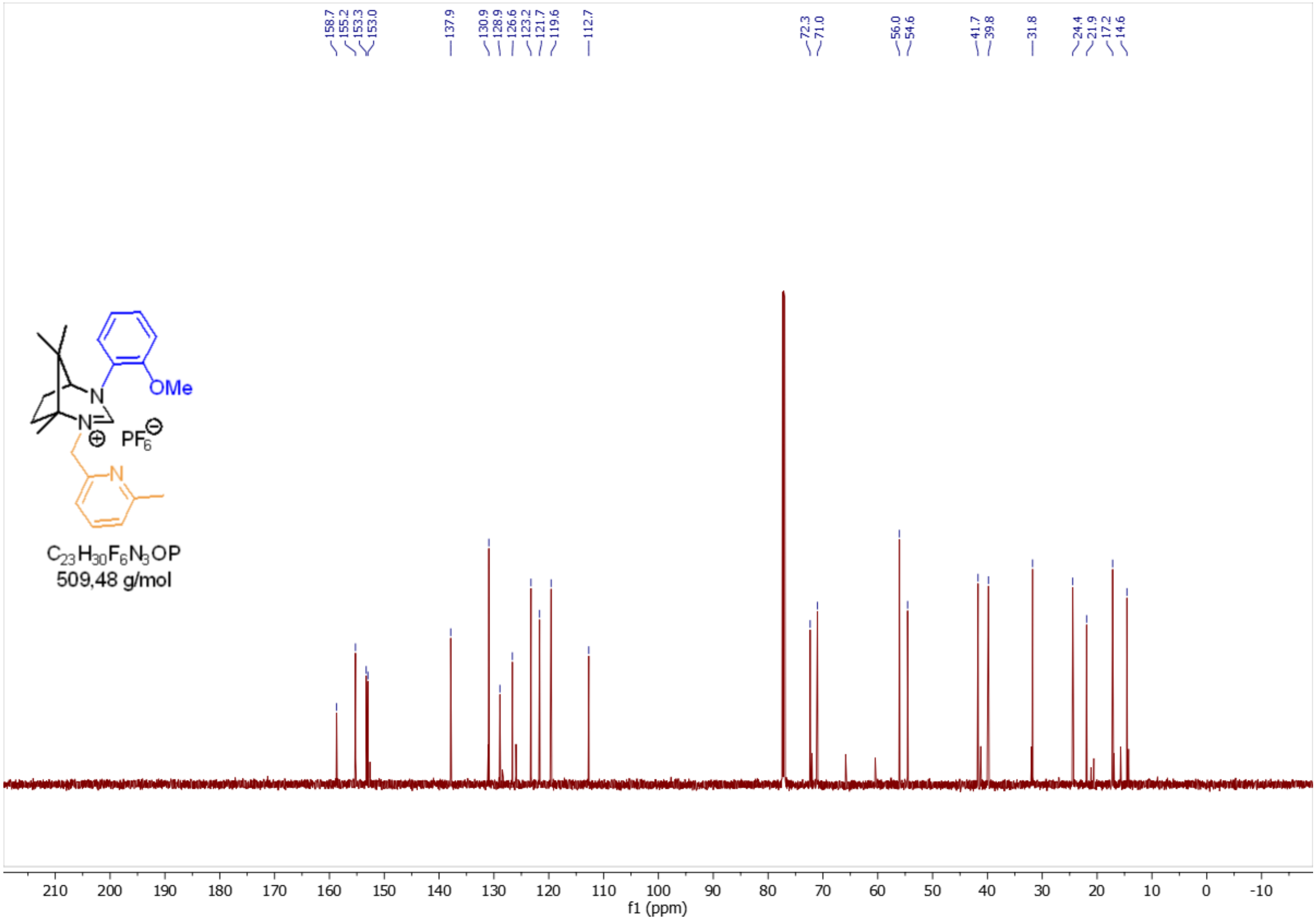

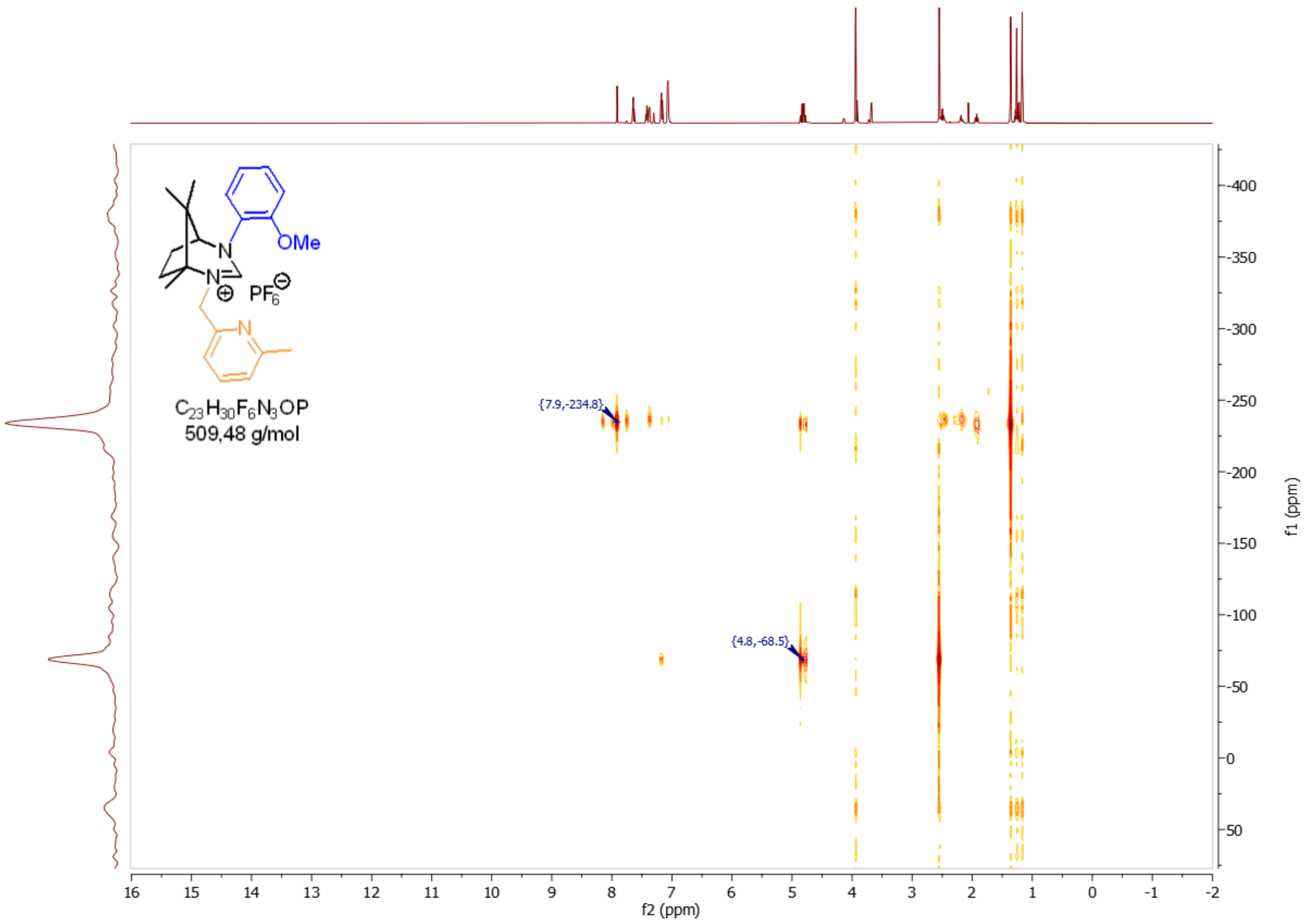

OMe
PF6
N
C23H30F6N3OP
509,48 g/mol
{7.9,-234.8}
{4.8,-68.5}
f2 (ppm)
f1 (ppm)

^{1}H NMR (600 MHz, $CDCl_3$), ^{13}C{^{1}H} NMR (151 MHz, $CDCl_3$) and ^{15}N HSQC NMR (61 MHz, $CDCl_3$) Analysis of Compound **3bp**

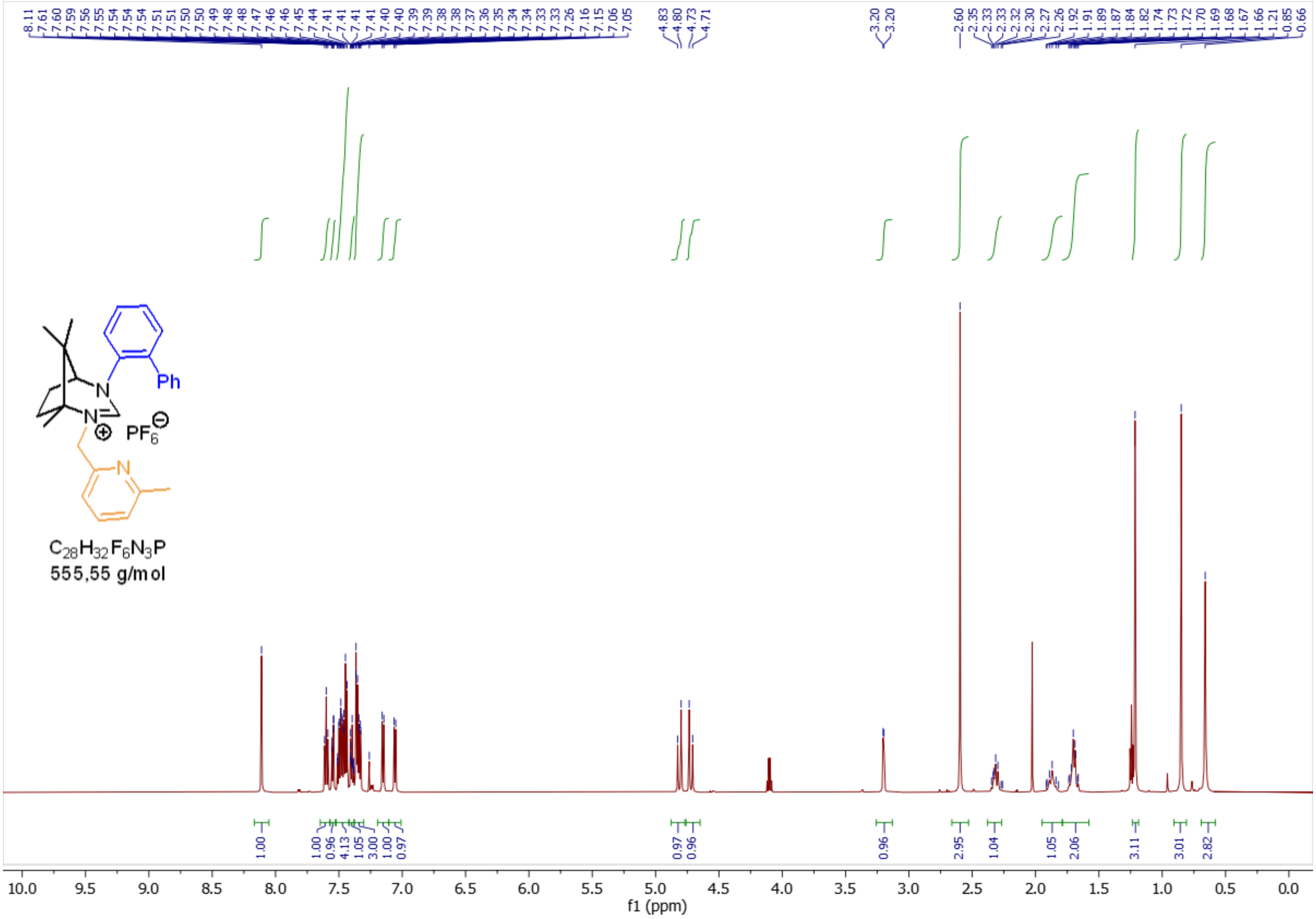

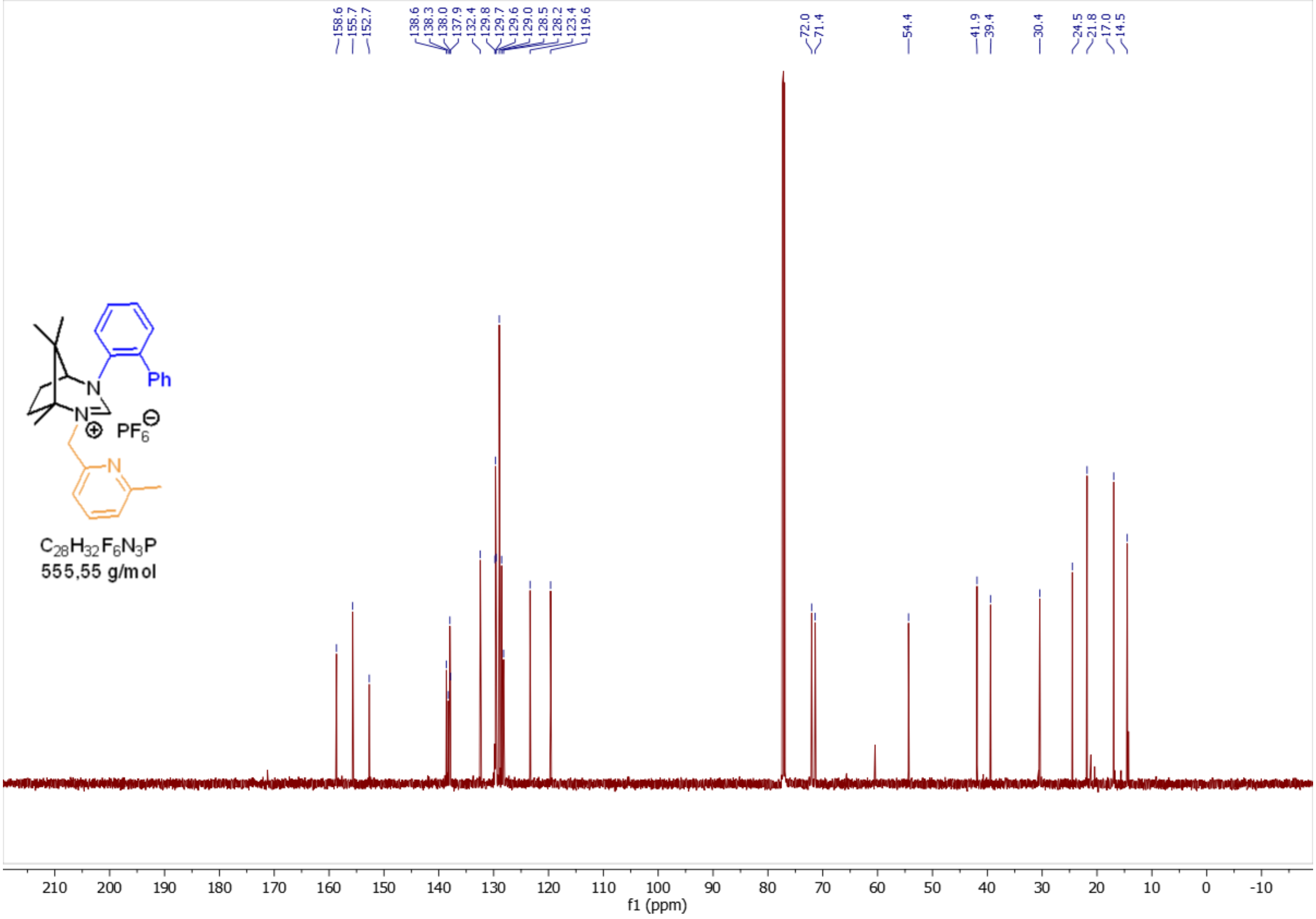

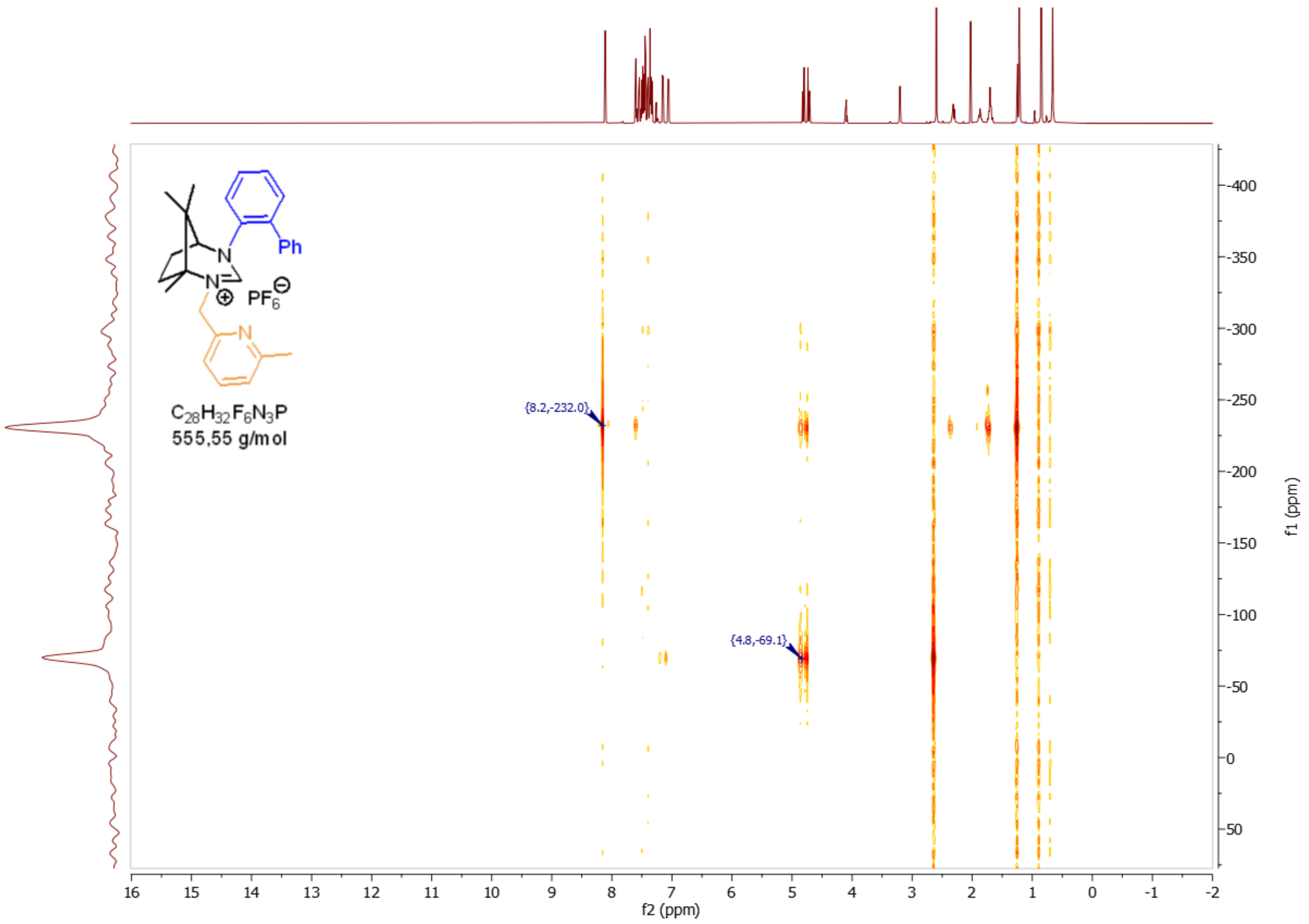

Ph
N
N
PF6
N
C28H32F6N3P
555,55 g/mol
{8.2,-232.0}
{4.8,-69.1}
f1 (ppm)
f2 (ppm)

^{1}H NMR (600 MHz, $CDCl_3$), $^{13}C\{^{1}H\}$ NMR (151 MHz, $CDCl_3$) and ^{15}N HSQC NMR (61 MHz, $CDCl_3$) Analysis of Compound **3br**

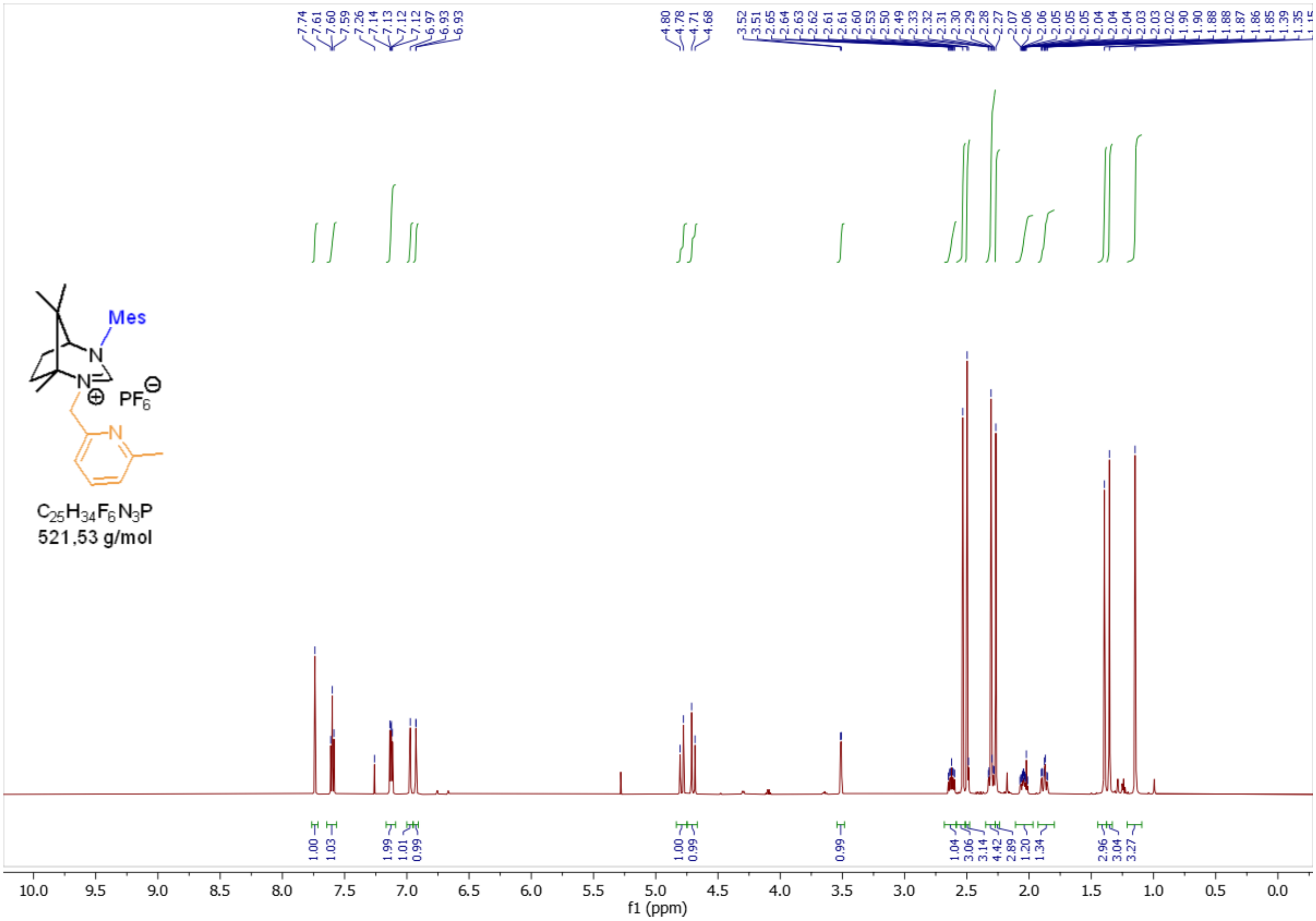

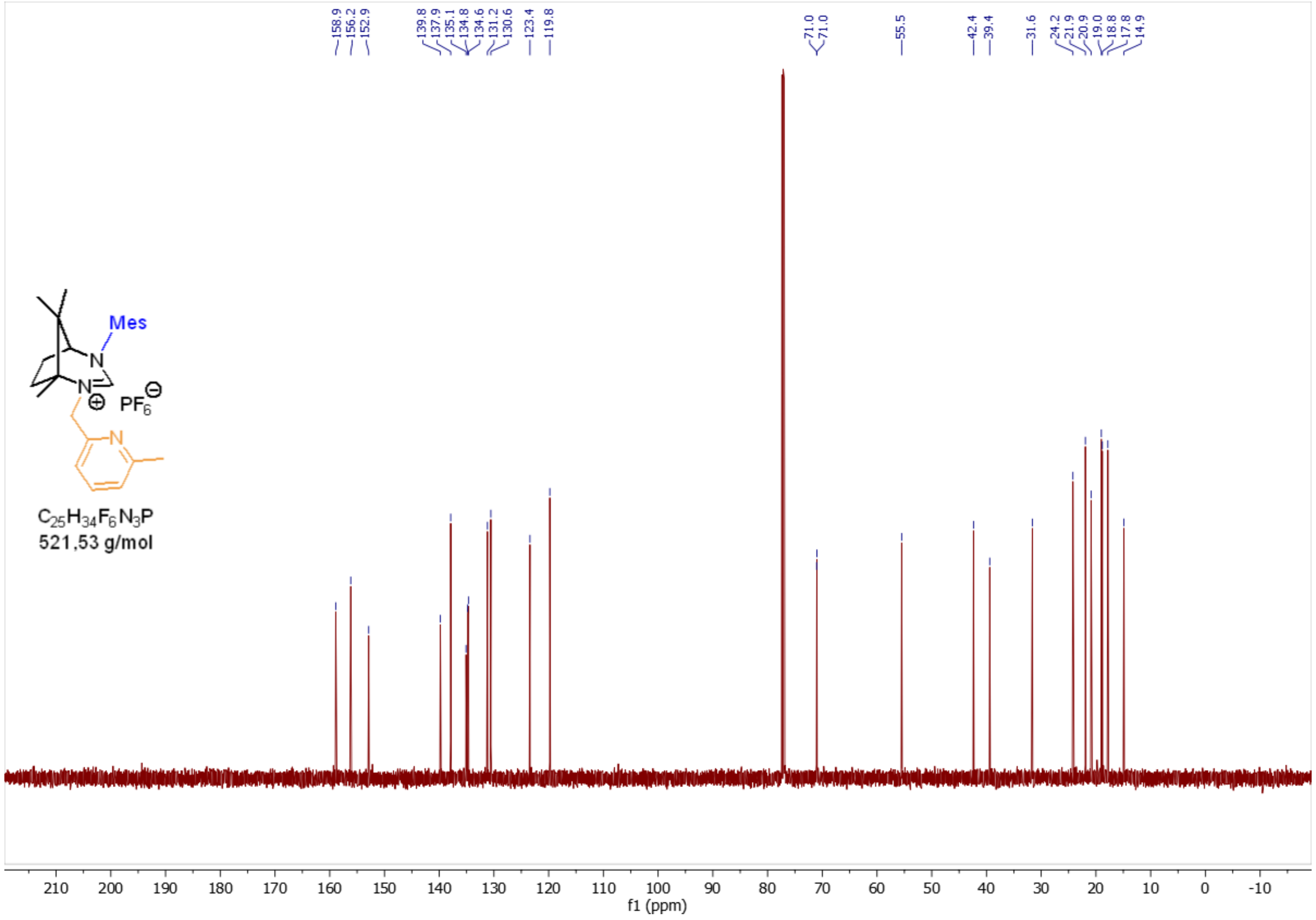

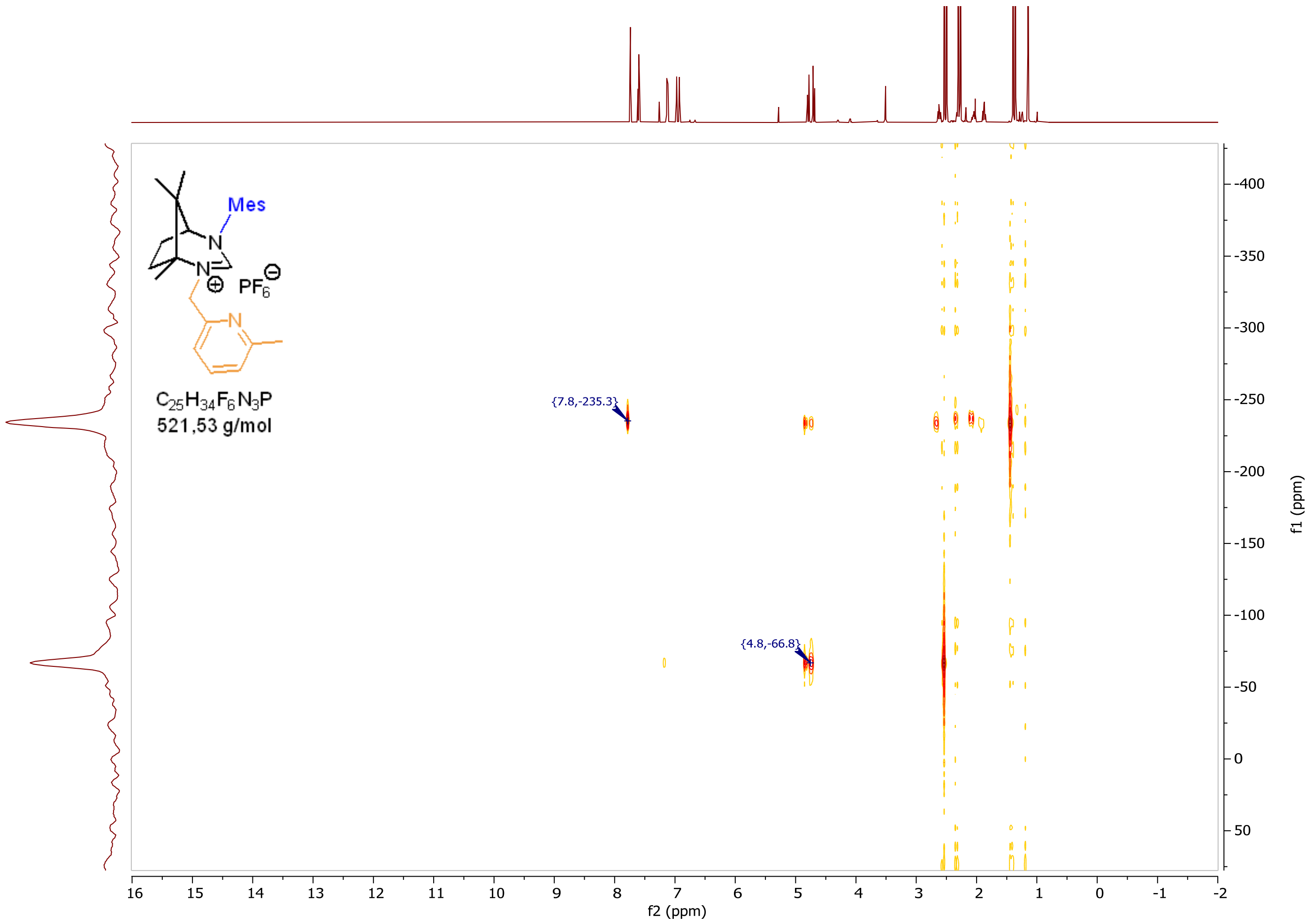

Mes
N
N
PF6
N
C25H34F6N3P
521,53 g/mol
{7.8,-235.3}
{4.8,-66.8}
f2 (ppm)
f1 (ppm)

^{1}H NMR (600 MHz, $CDCl_3$), ^{13}C{^{1}H} NMR (151 MHz, $CDCl_3$) and ^{15}N HSQC NMR (61 MHz, $CDCl_3$) Analysis of Compound **3bs**

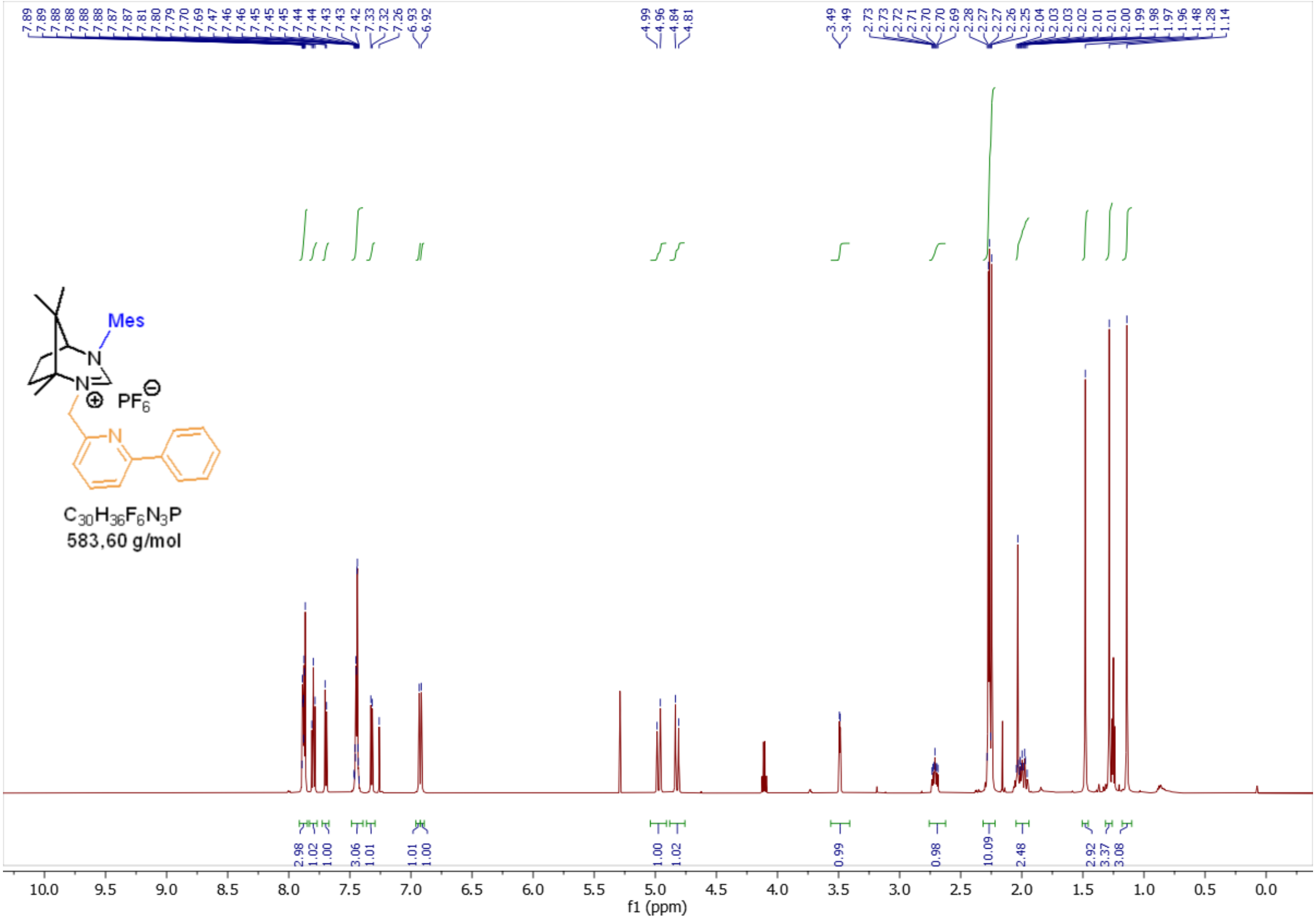

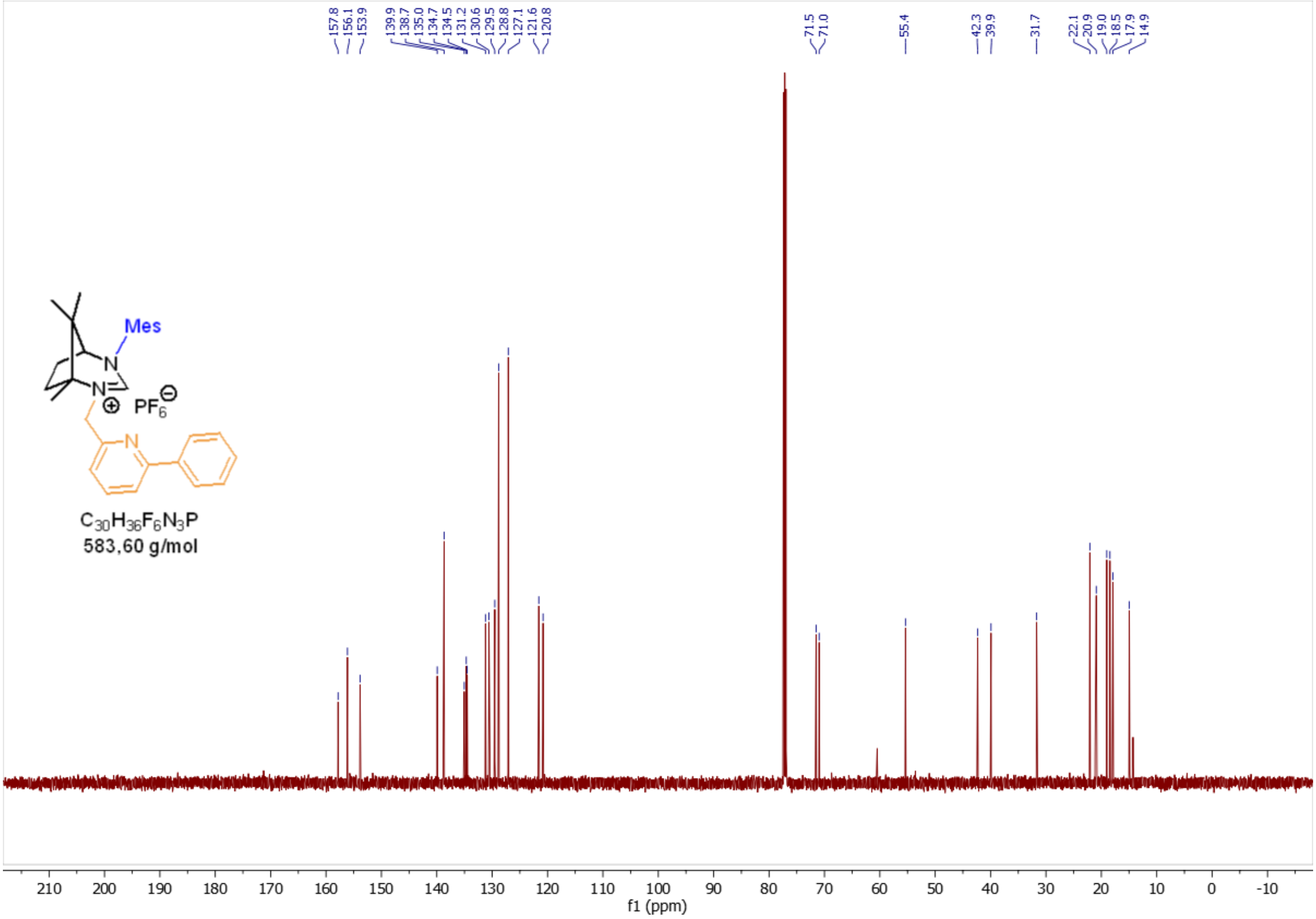

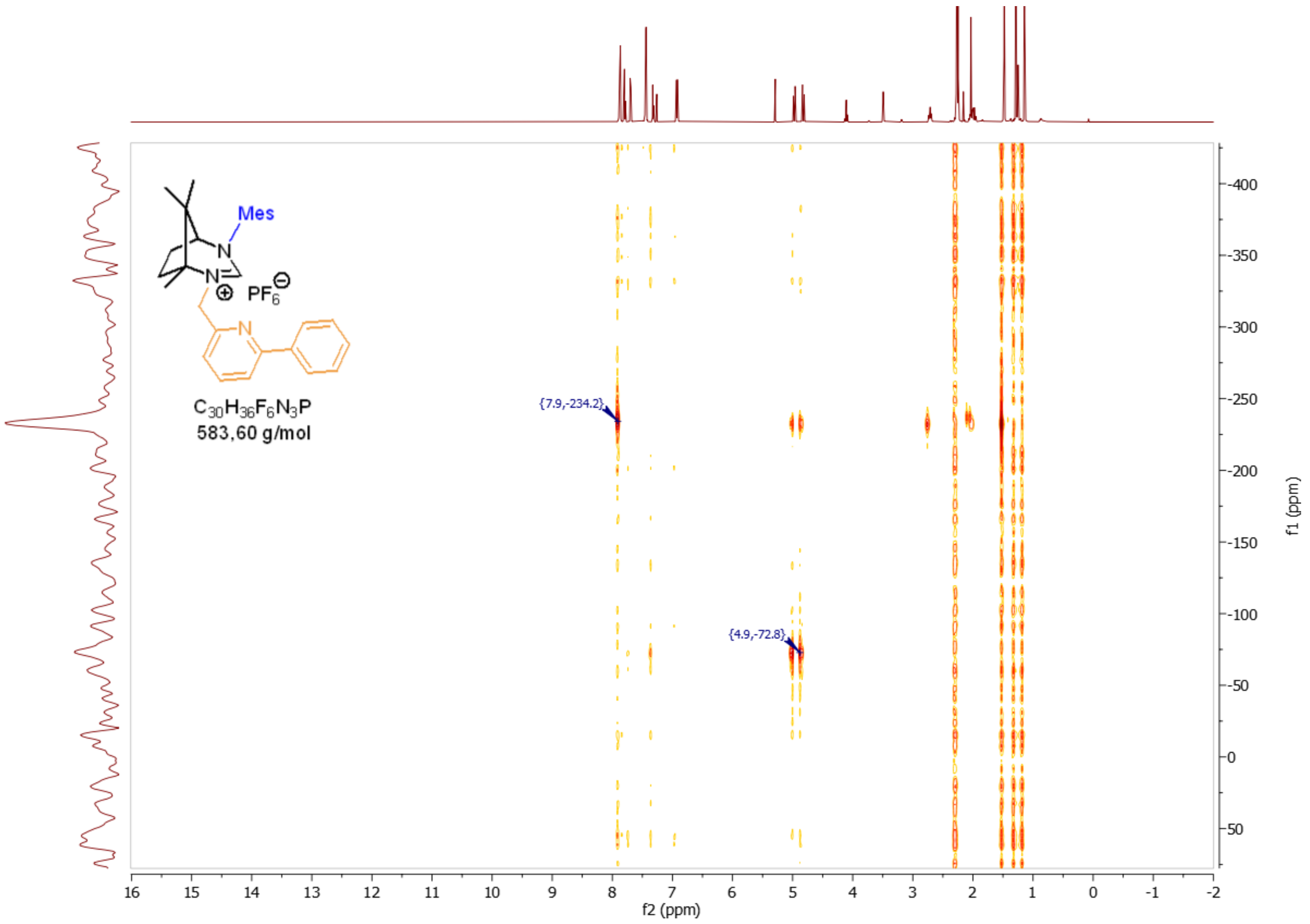

Mes
PF6
C30H36F6N3P
583,60 g/mol
{7.9,-234.2}
{4.9,-72.8}
f2 (ppm)
f1 (ppm)

^{1}H NMR (600 MHz, $CDCl_3$), $^{13}C\{^1H\}$ NMR (151 MHz, $CDCl_3$) and ^{15}N HSQC NMR (61 MHz, $CDCl_3$) Analysis of Compound **3bt**

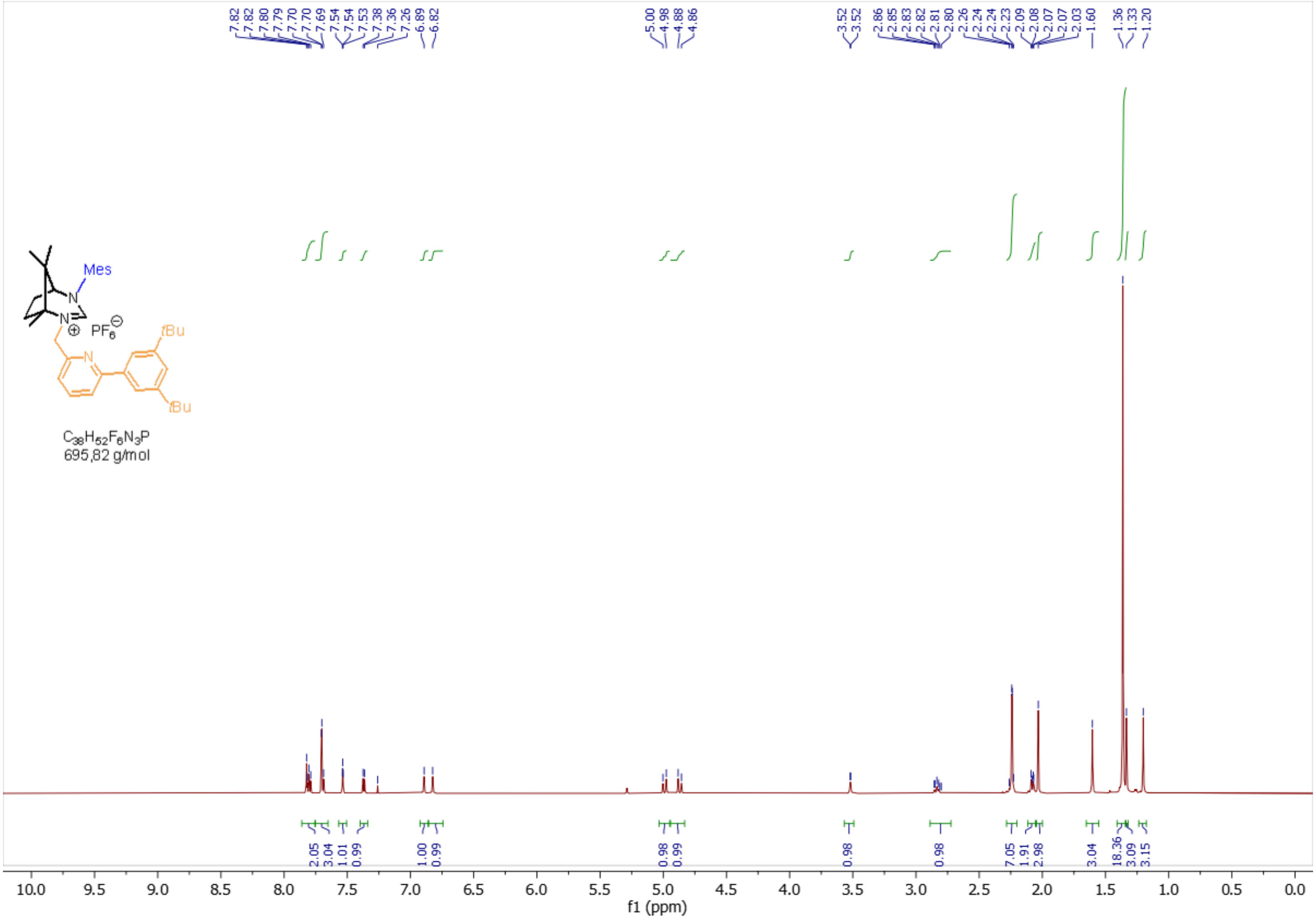

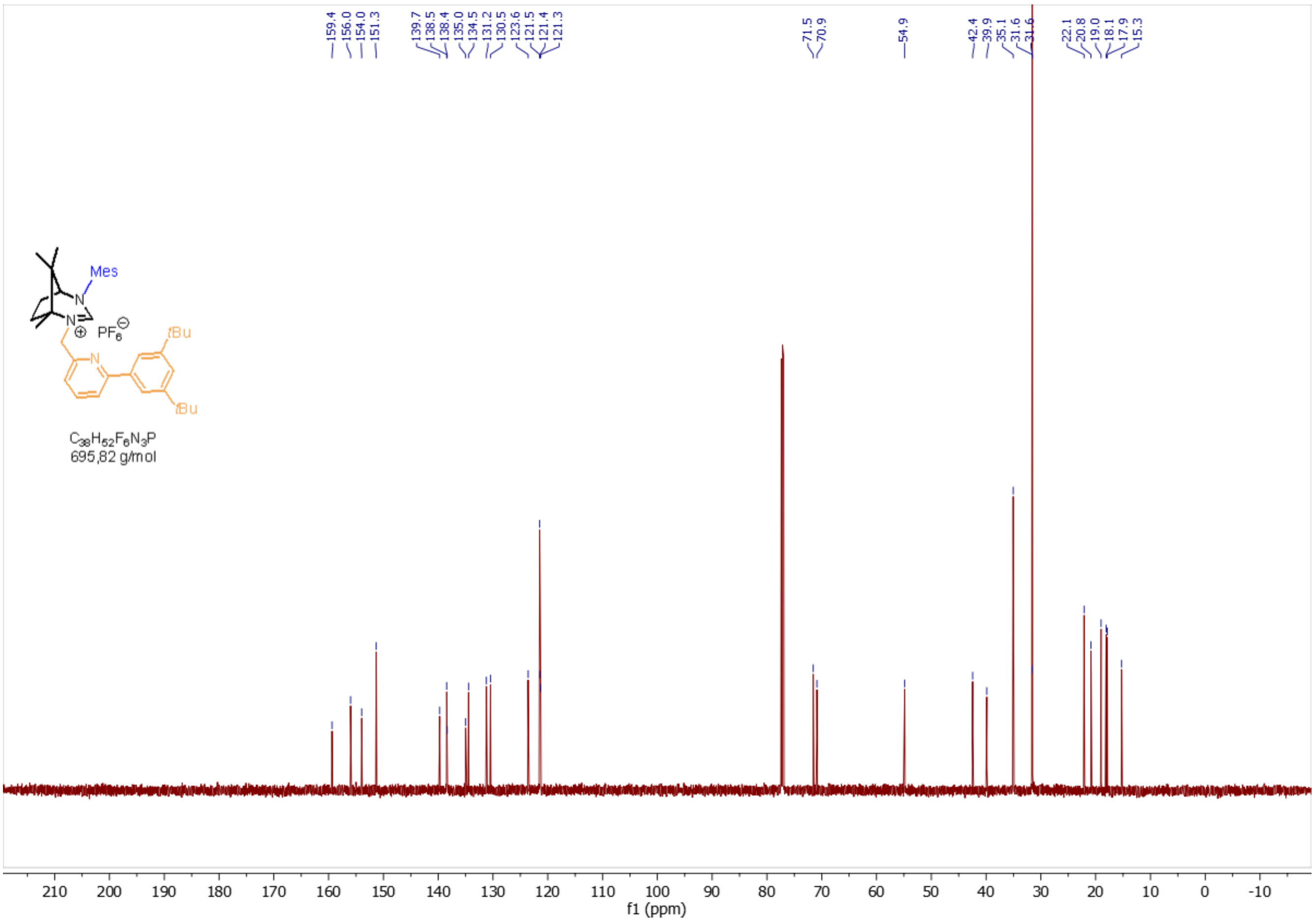

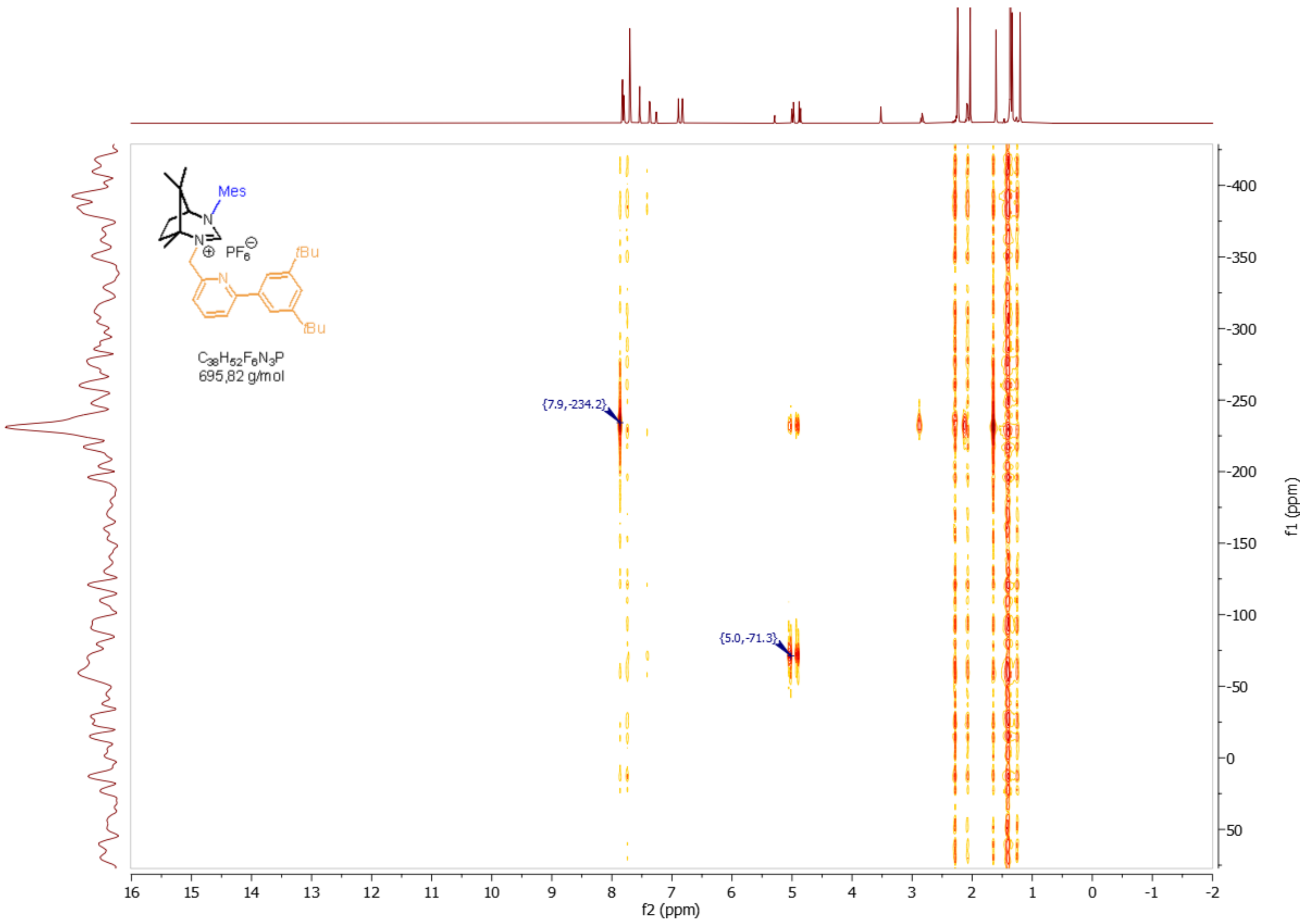

Mes
PF6
tBu
tBu
C38H52F6N3P
695,82 g/mol
{7.9,-234.2}
{5.0,-71.3}
f1 (ppm)
f2 (ppm)

^{1}H NMR (600 MHz, $CDCl_3$) and $^{13}C\{^{1}H\}$ NMR (151 MHz, $CDCl_3$) Analysis of **Complex Rh4aa**

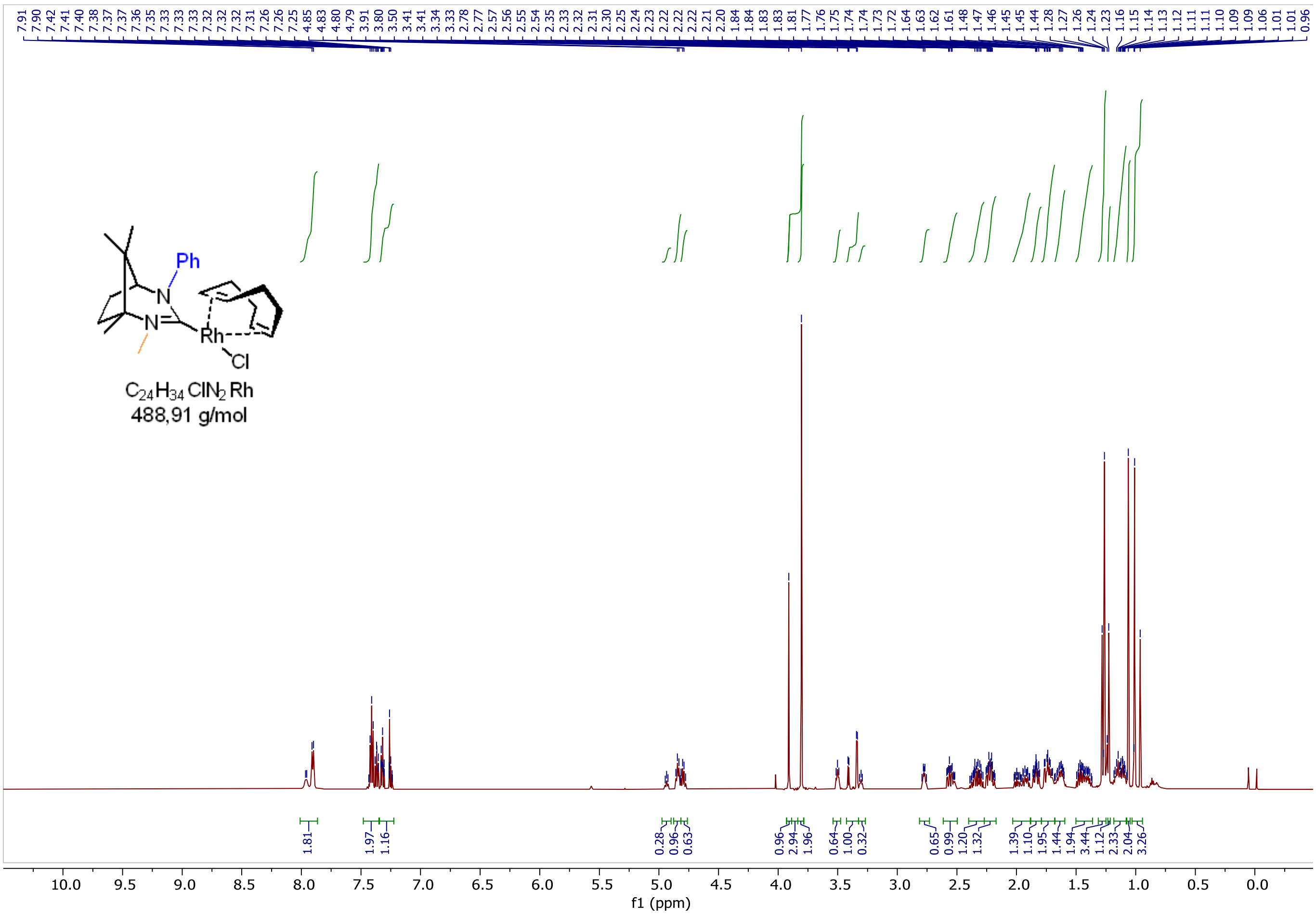

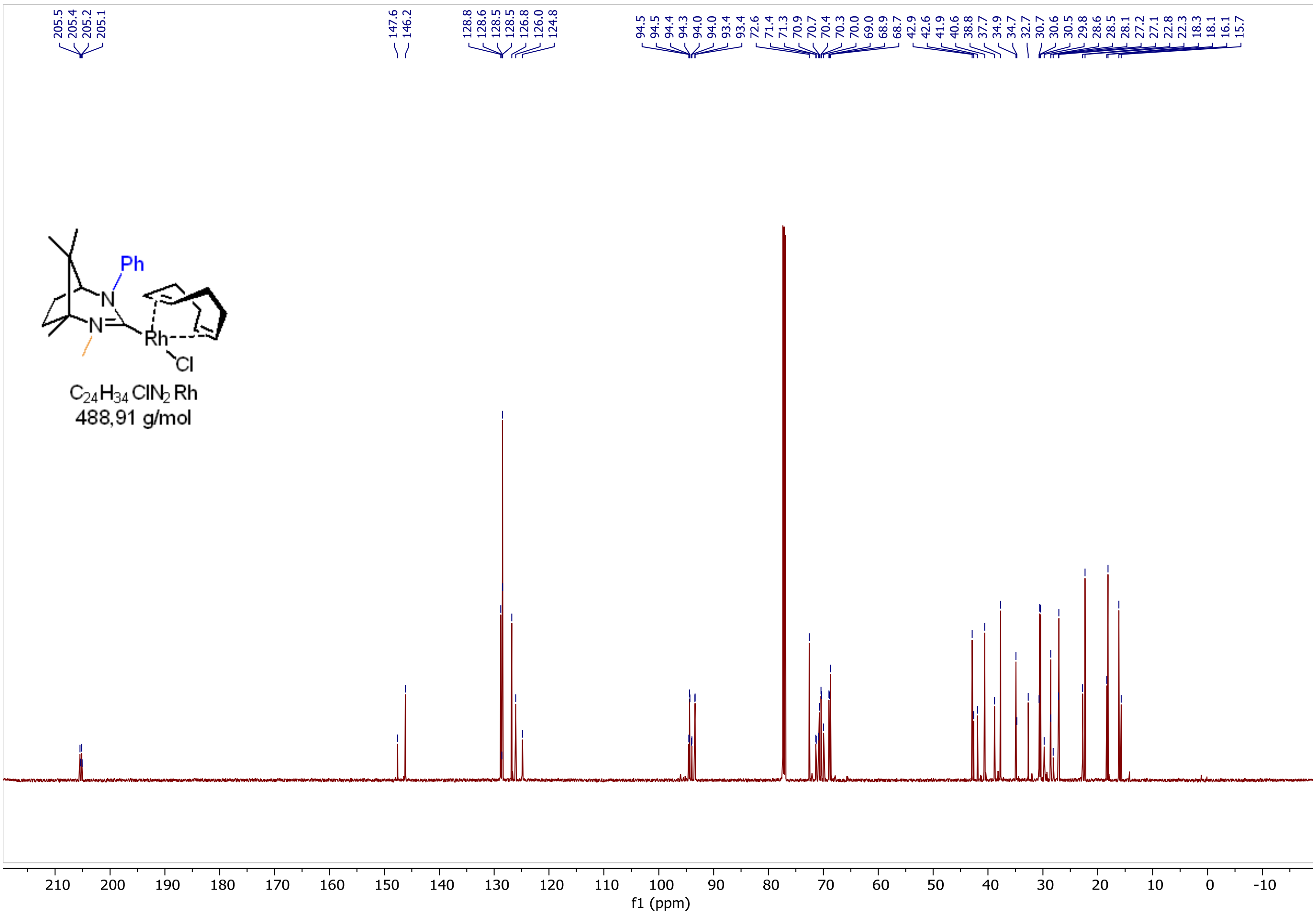

^{1}H NMR (600 MHz, $CDCl_3$) and $^{13}C\{^{1}H\}$ NMR (151 MHz, $CDCl_3$) Analysis of **Complex Rh4ab**

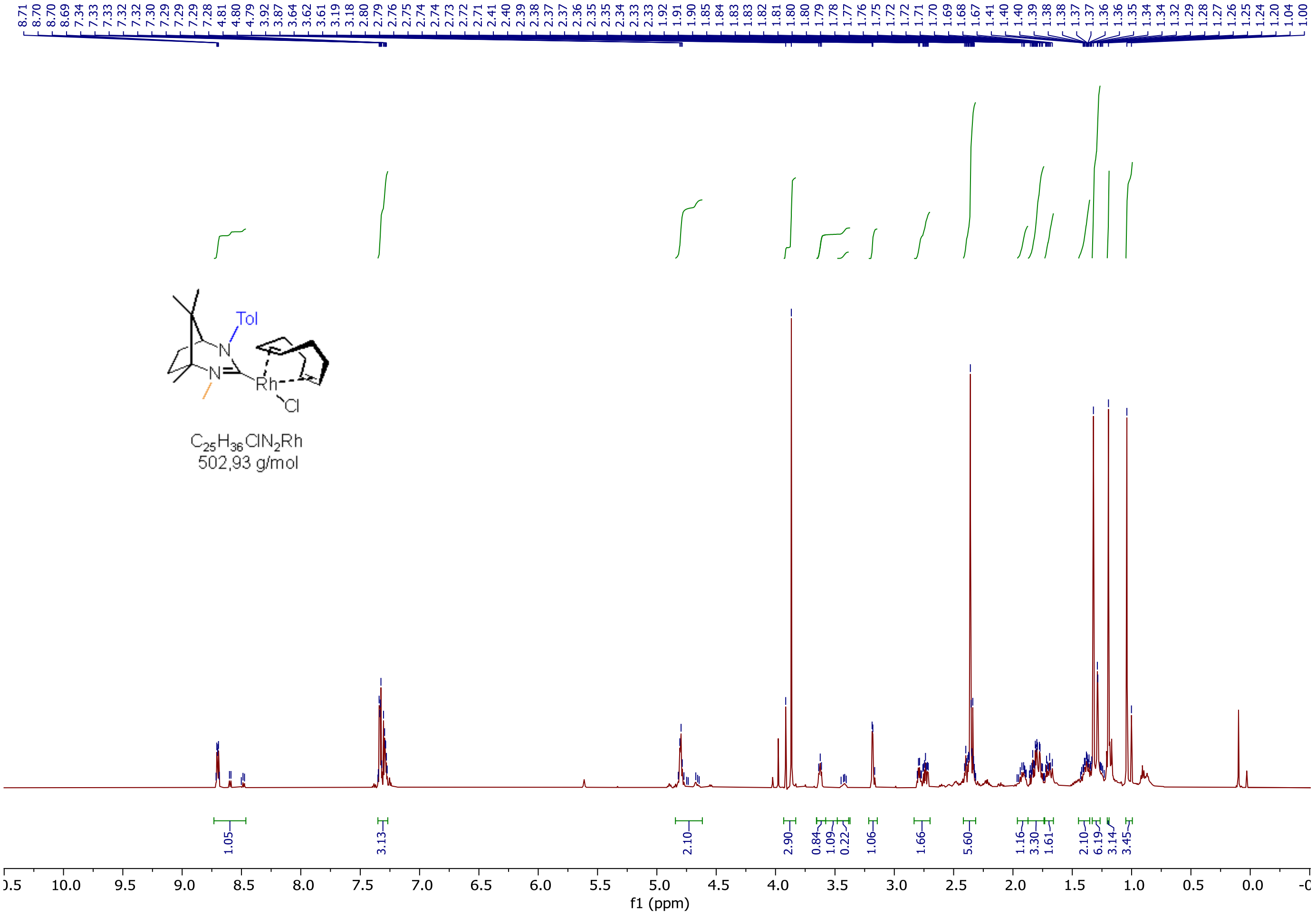

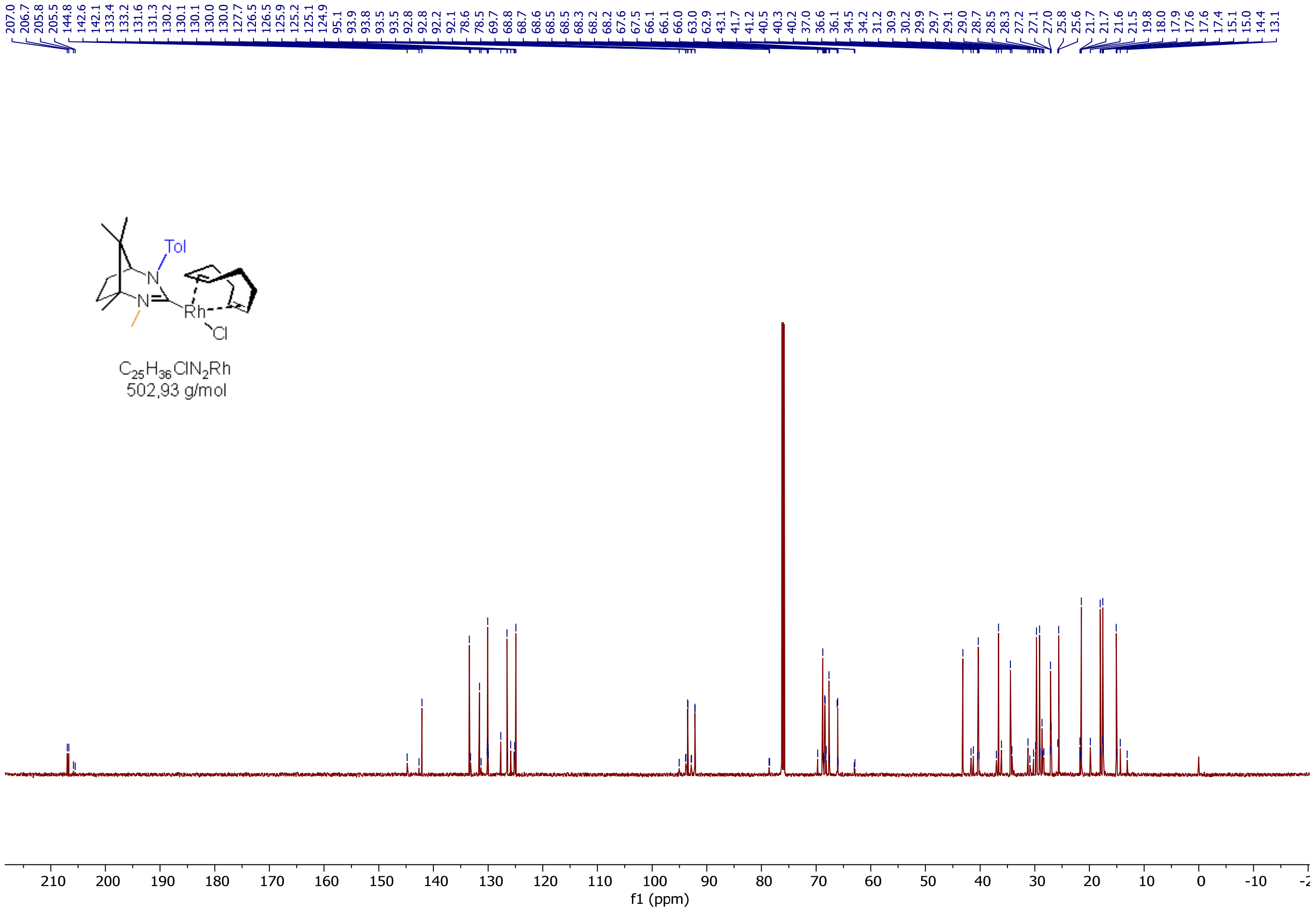

^{1}H NMR (600 MHz, $CDCl_3$) and $^{13}C\{^{1}H\}$ NMR (151 MHz, $CDCl_3$) Analysis of **Complex Rh4ac**

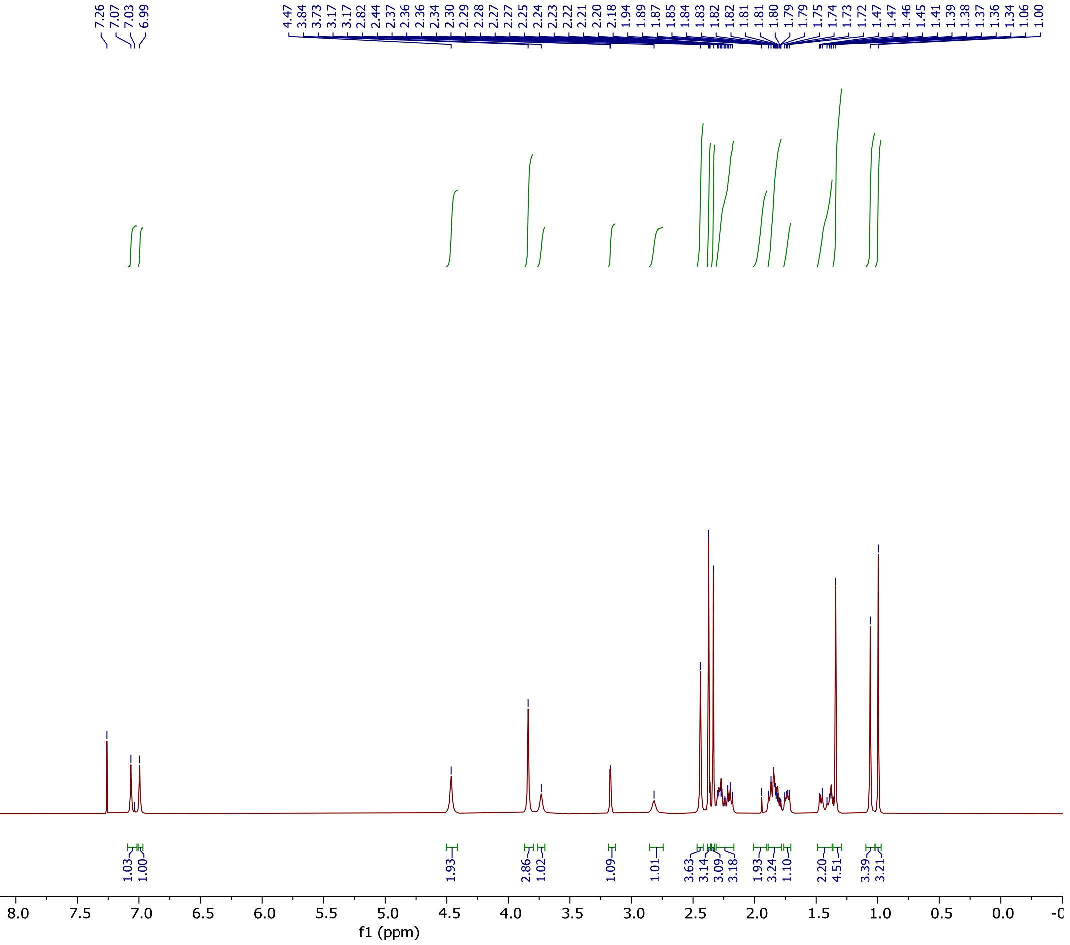

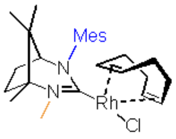

$C_{27}H_{40}ClN_2Rh$
530,99 g/mol

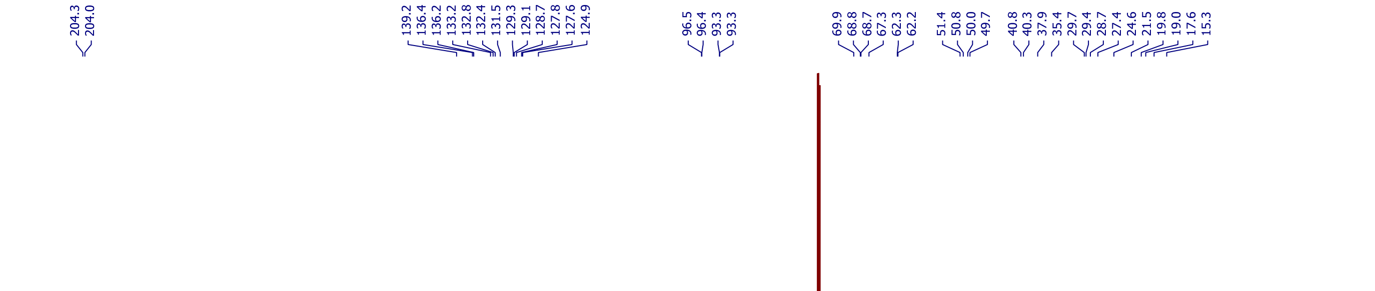

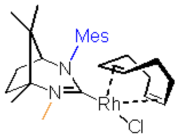

$C_{27}H_{40}ClN_2Rh$
530,99 g/mol

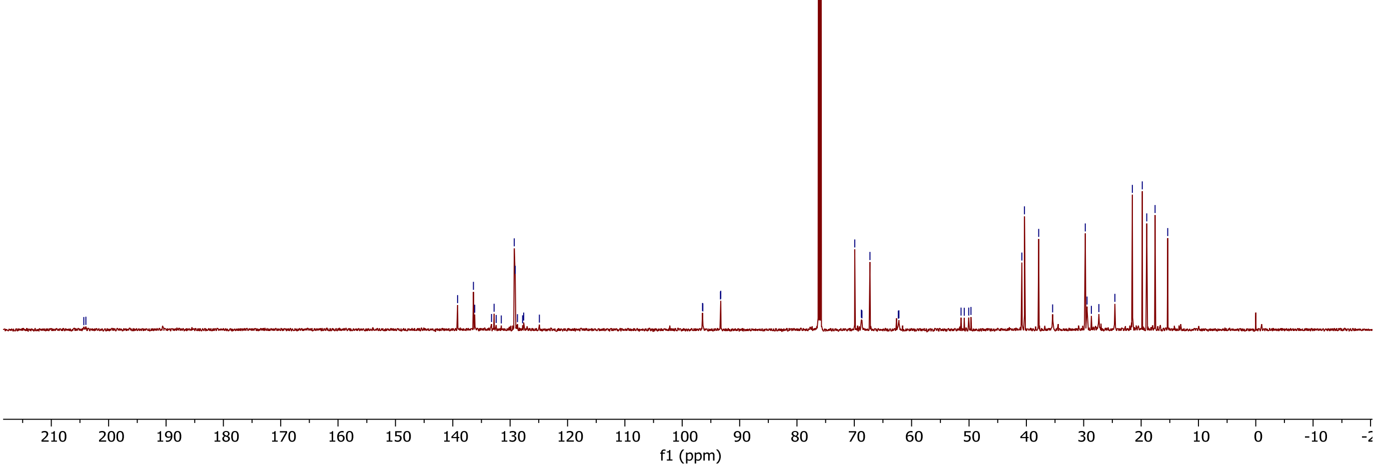

^{1}H NMR (600 MHz, $CDCl_3$) and $^{13}C\{^{1}H\}$ NMR (151 MHz, $CDCl_3$) Analysis of **Complex Rh4ad**

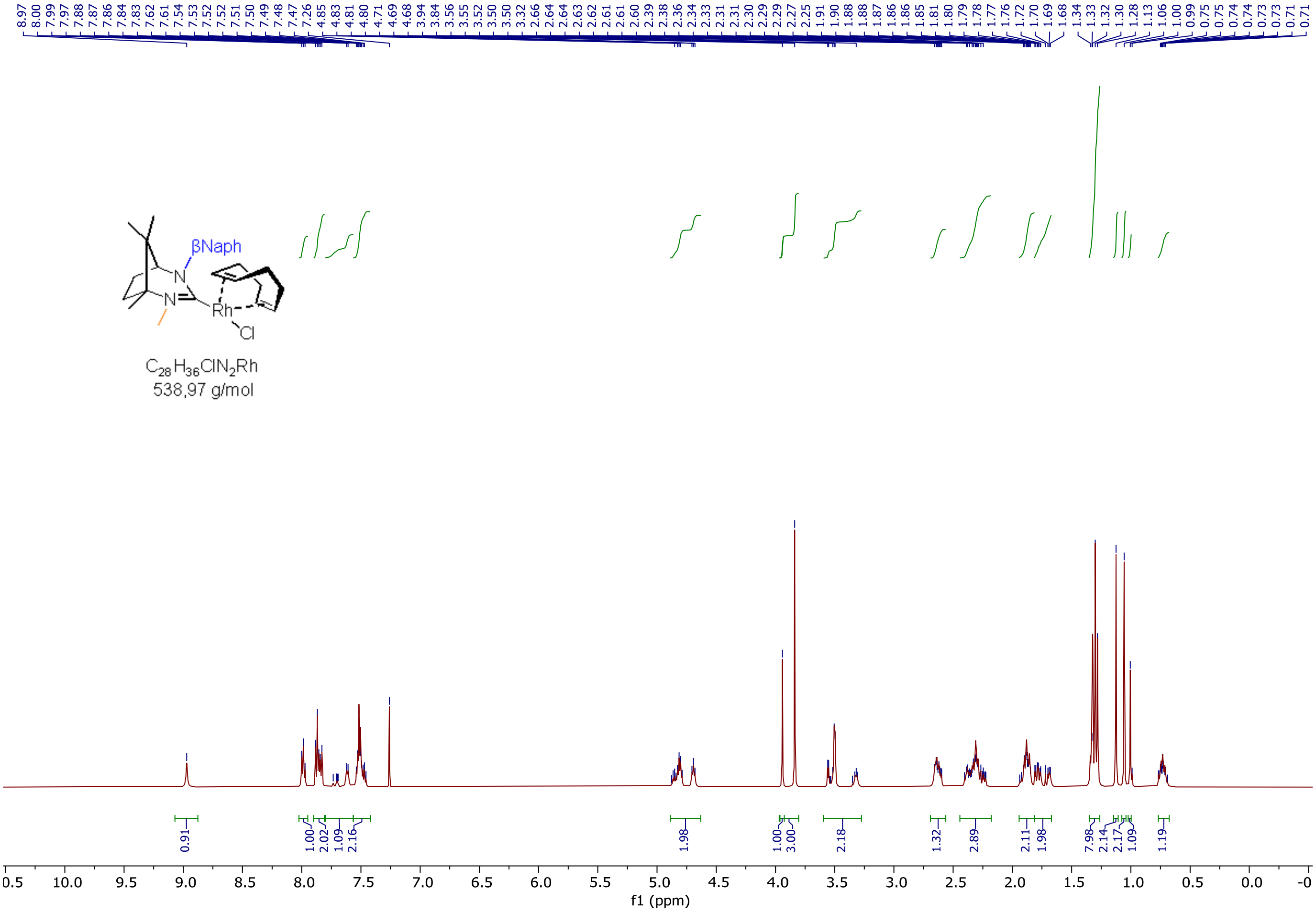

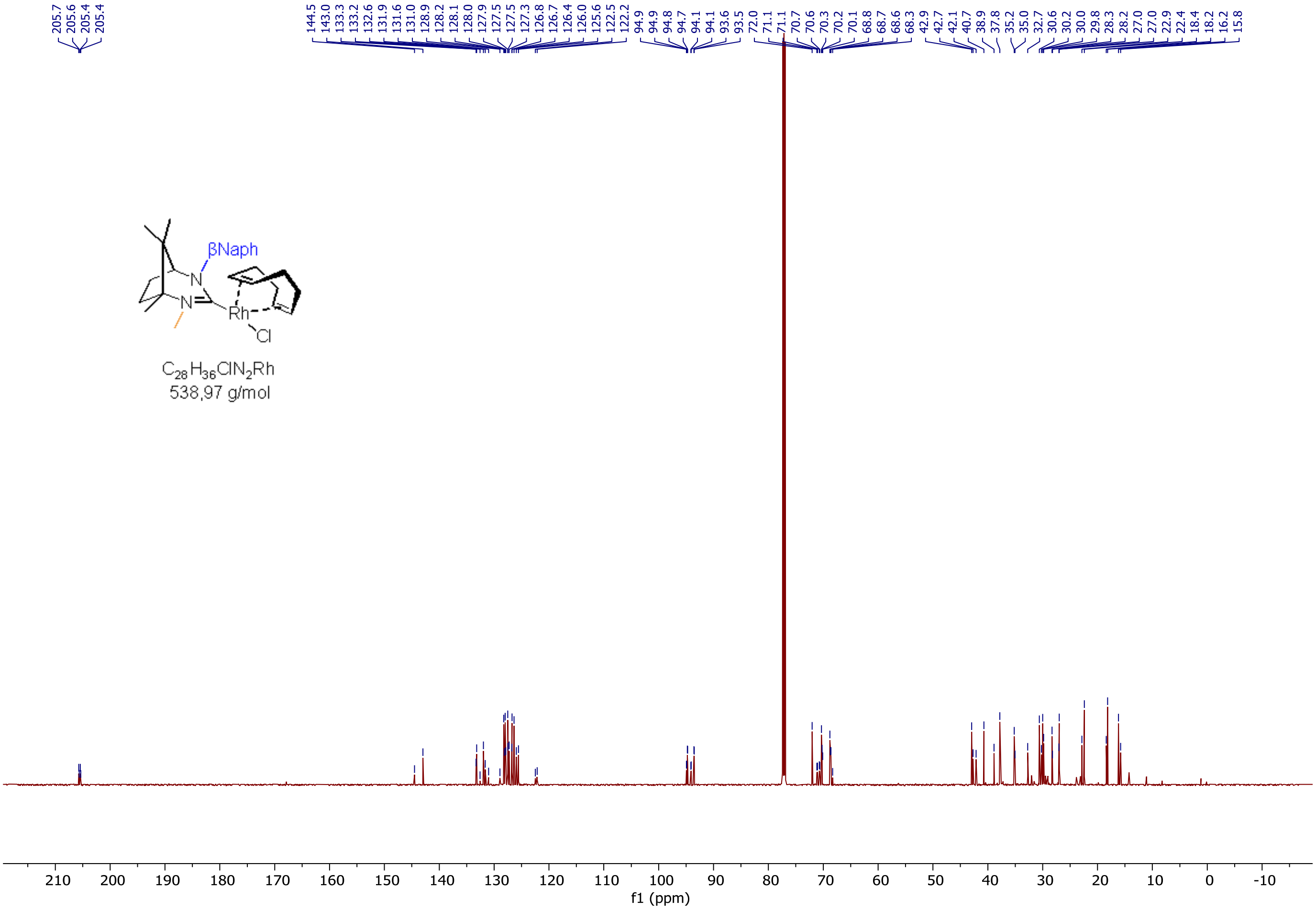

^{1}H NMR (600 MHz, $CDCl_3$) and ^{13}C{^{1}H} NMR (151 MHz, $CDCl_3$) Analysis of **Complex Rh4ae**

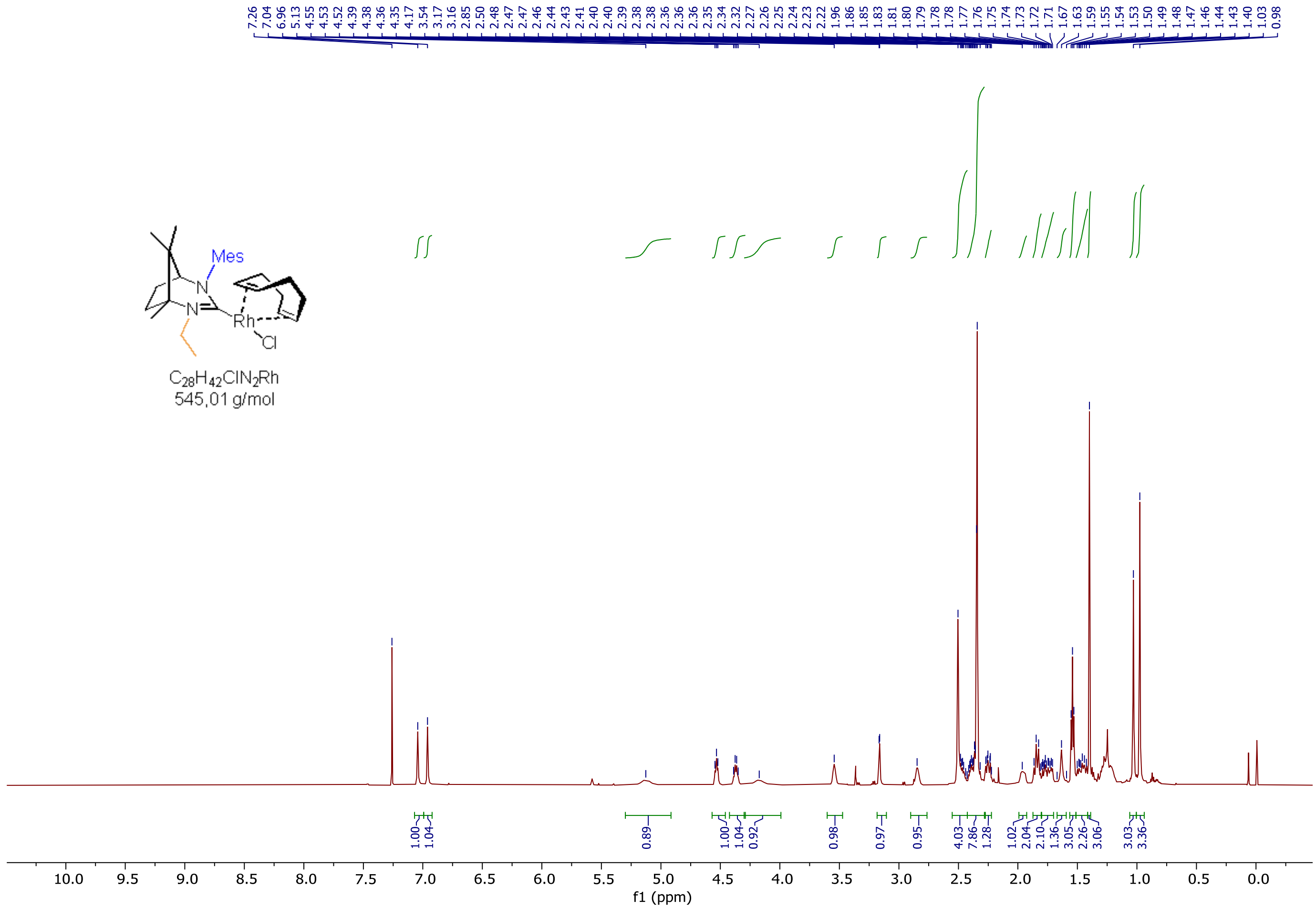

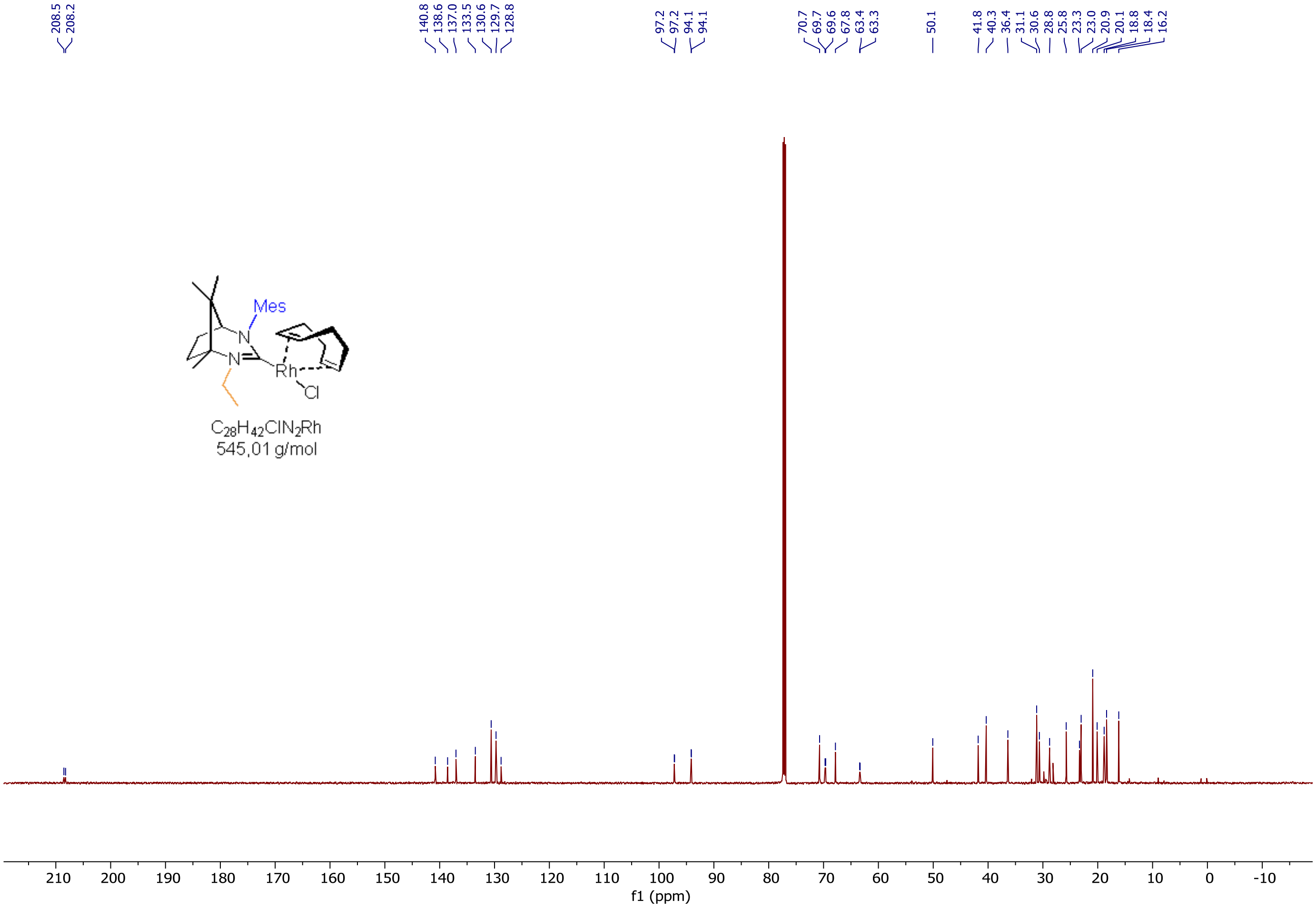

^{1}H NMR (600 MHz, $CDCl_3$) and $^{13}C\{^{1}H\}$ NMR (151 MHz, $CDCl_3$) Analysis of **Complex Rh4af**

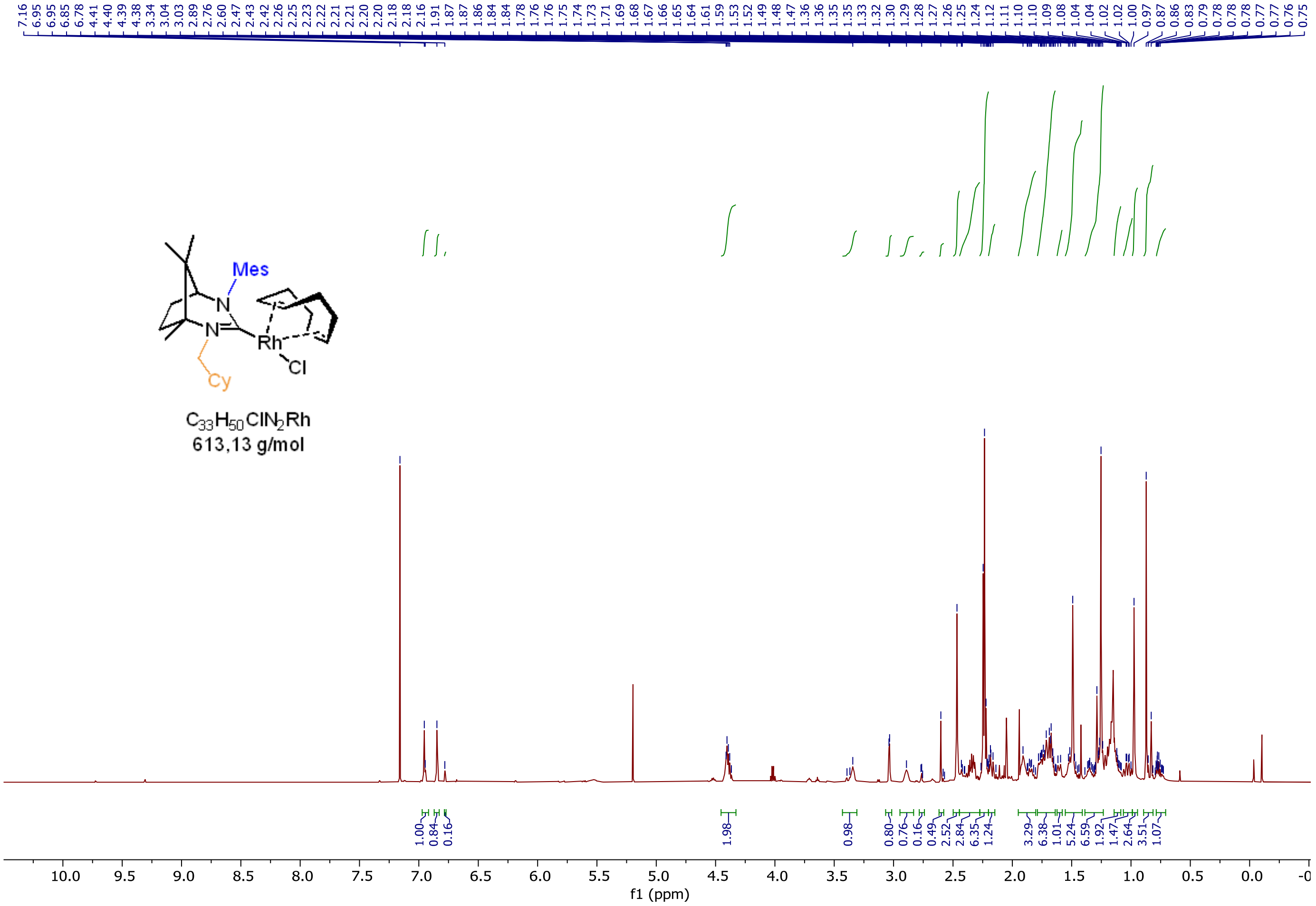

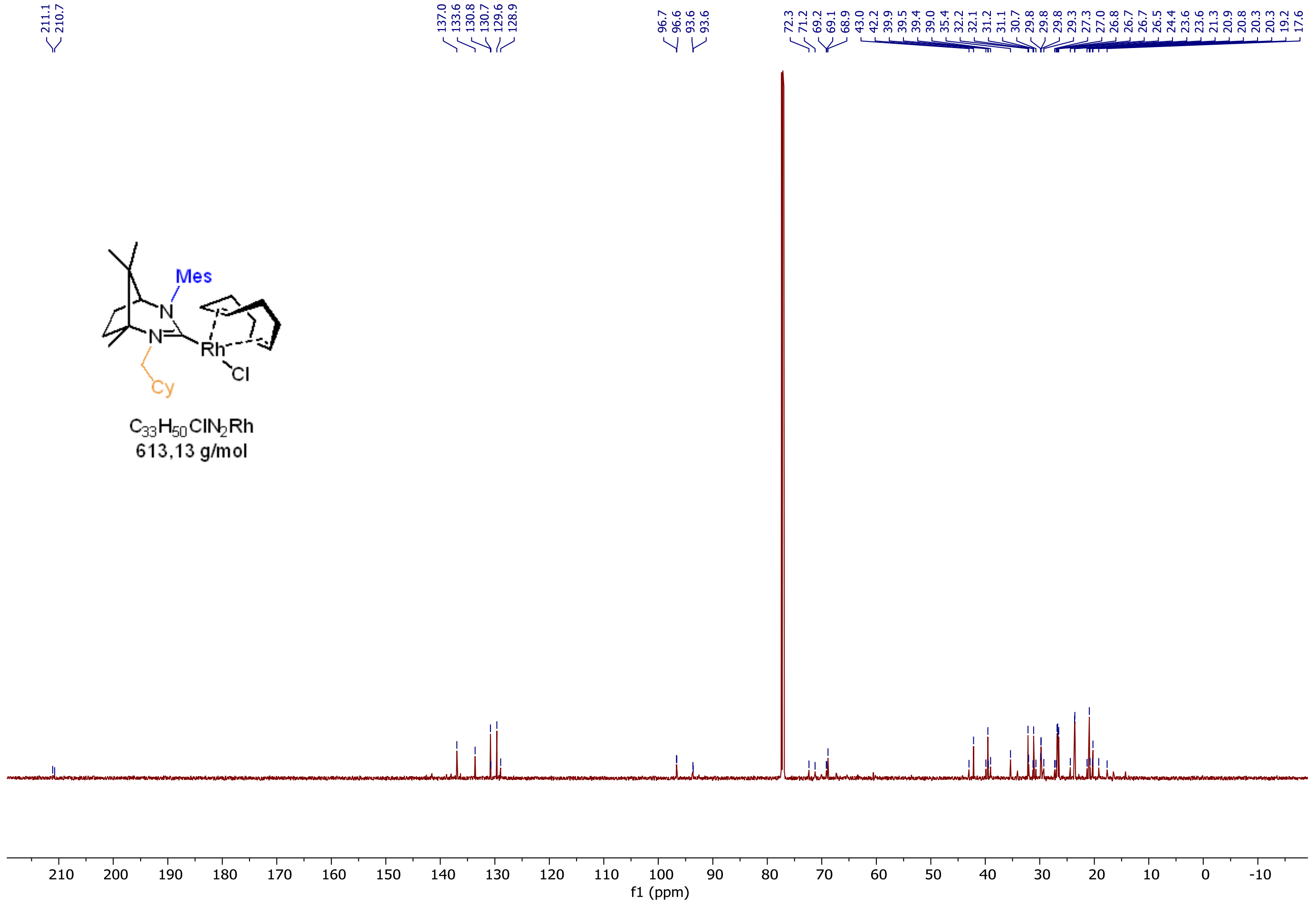

^1H NMR (600 MHz, $CDCl_3$) and $^{13}C\{^1H\}$ NMR (151 MHz, $CDCl_3$) Analysis of **Complex Rh4ag**

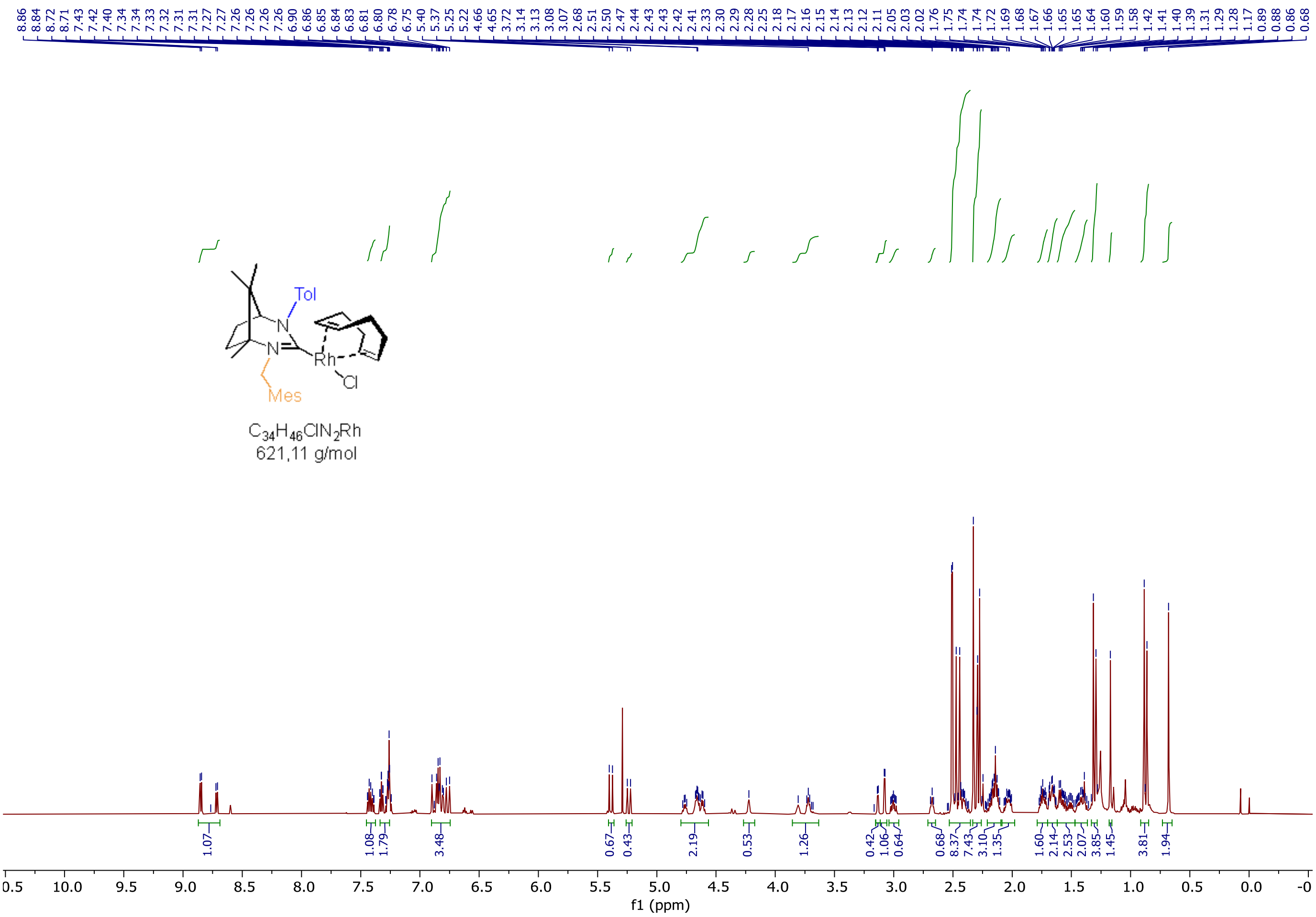

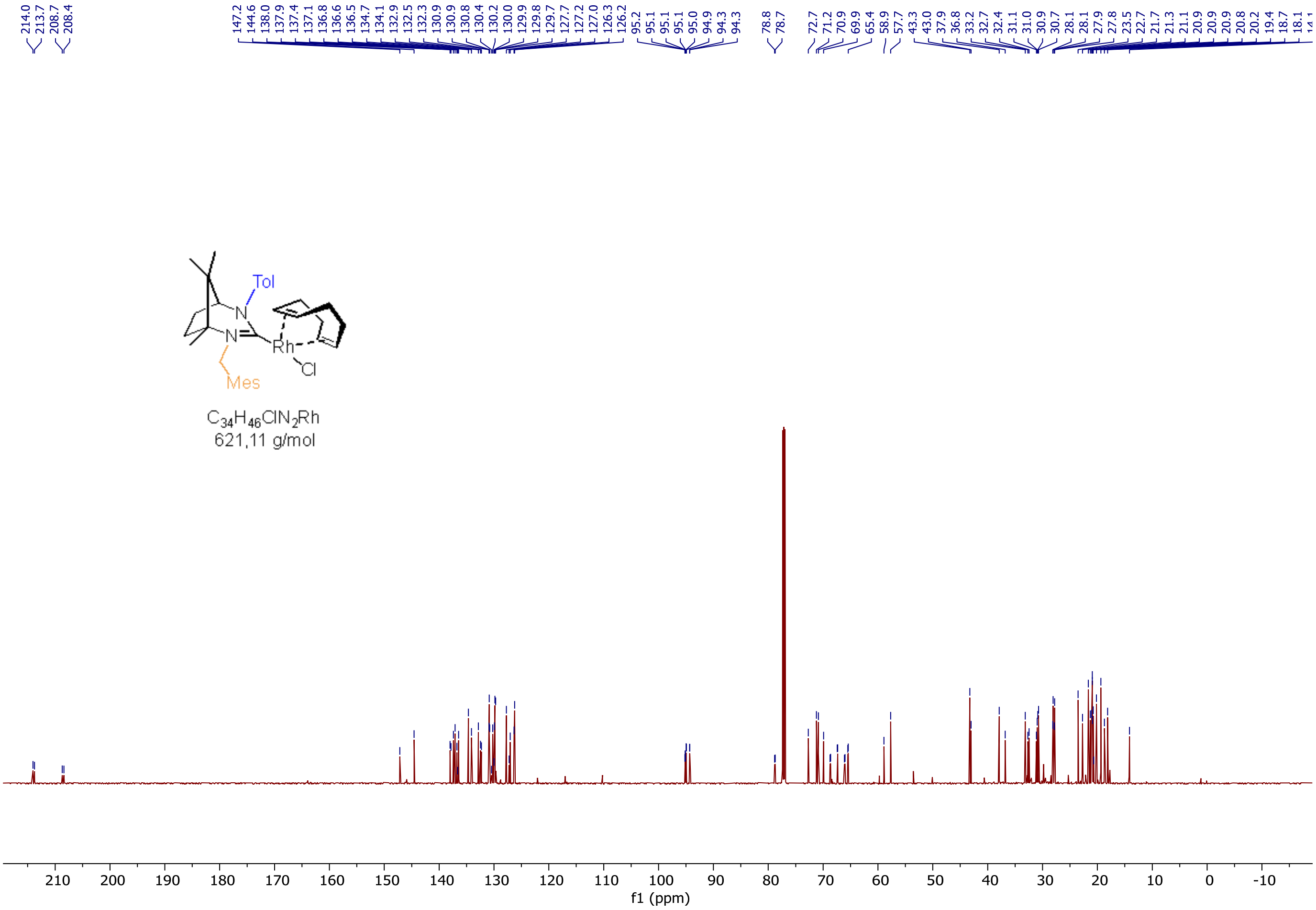

^{1}H NMR (600 MHz, $CDCl_3$) and $^{13}C\{^{1}H\}$ NMR (151 MHz, $CDCl_3$) Analysis of **Complex Rh4ah**

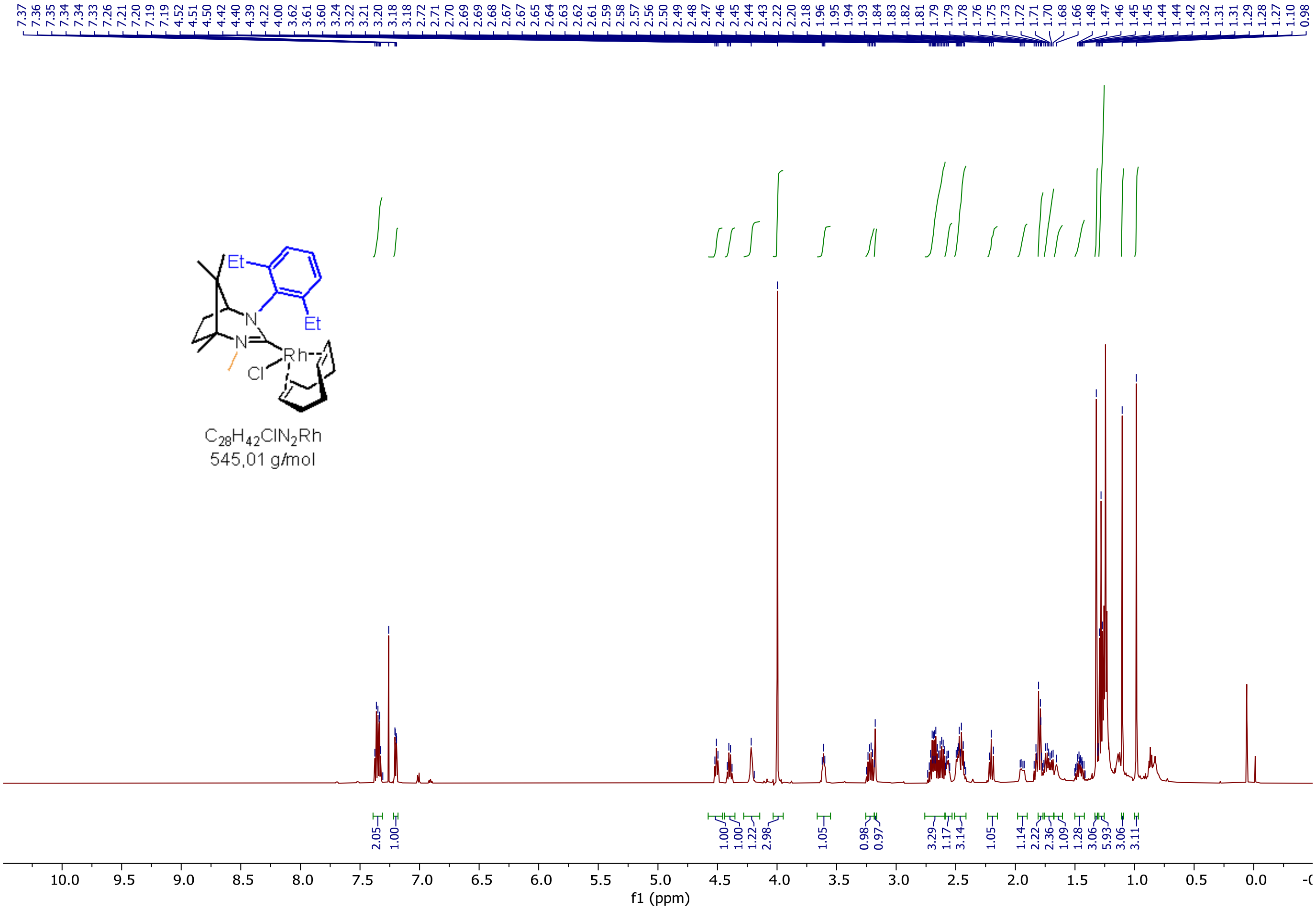

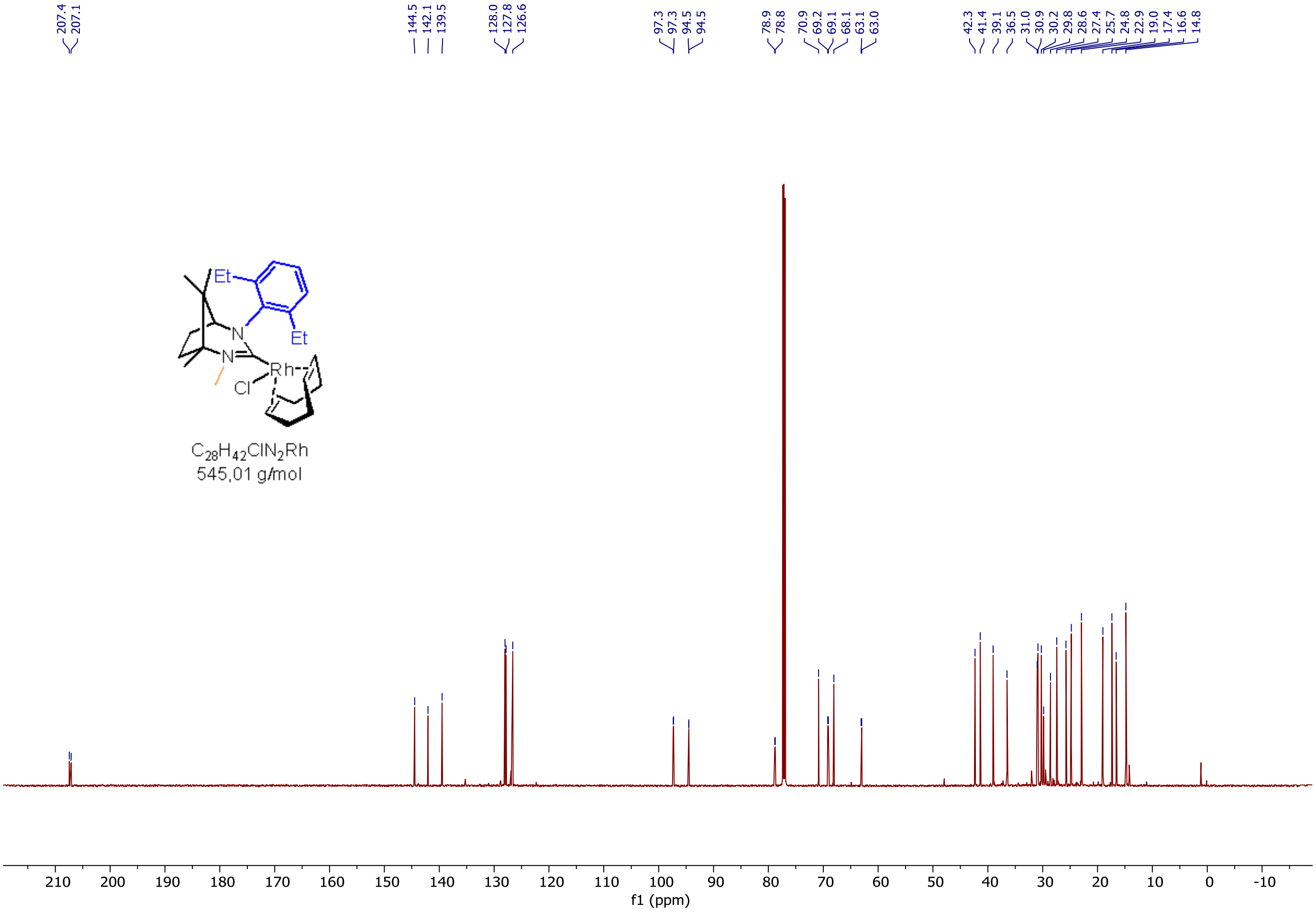

^1H NMR (600 MHz, CD_2Cl_2) and $^{13}C\{^1H\}$ NMR (151 MHz, CD_2Cl_2) Analysis of **Complex Rh4ai**

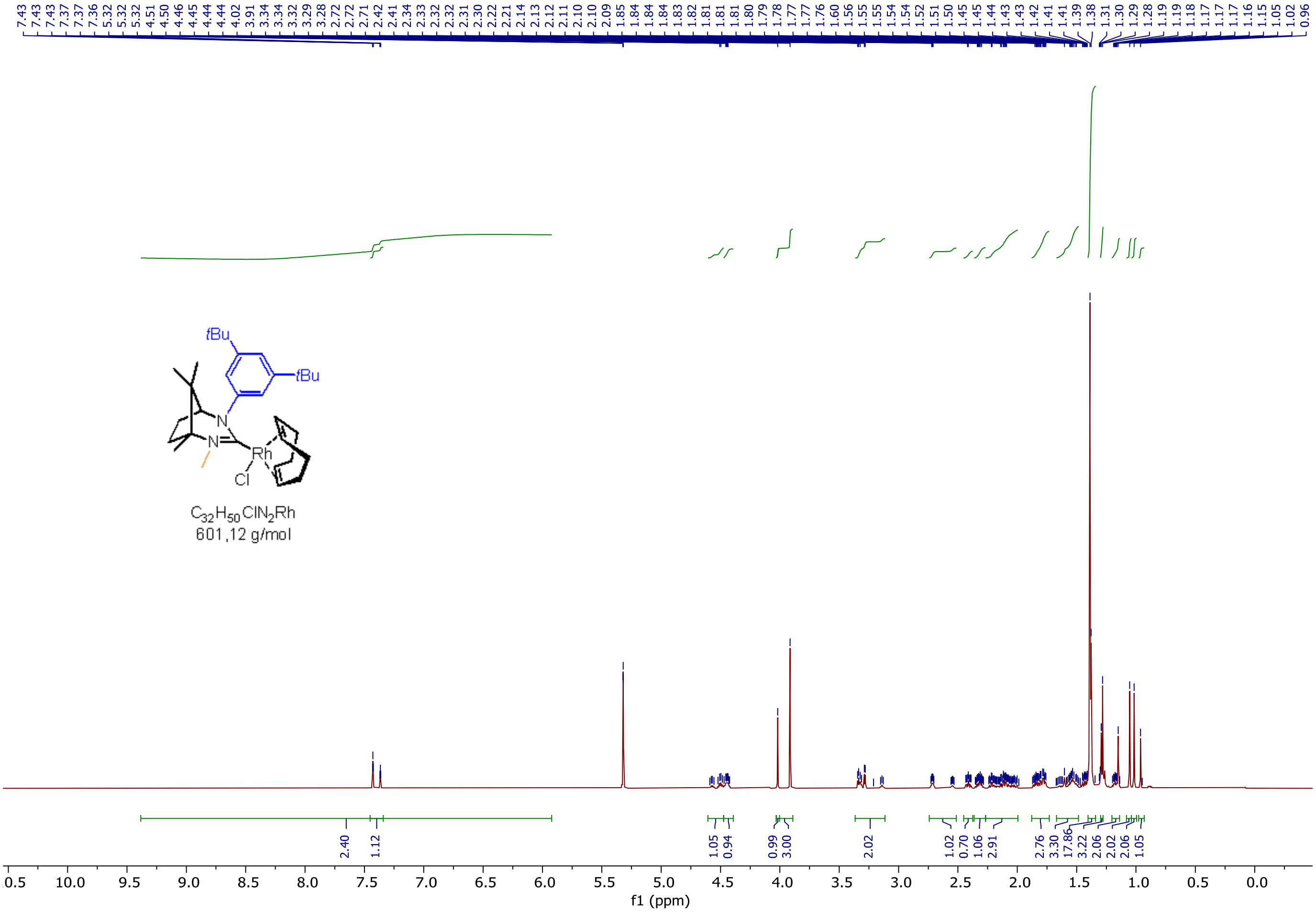

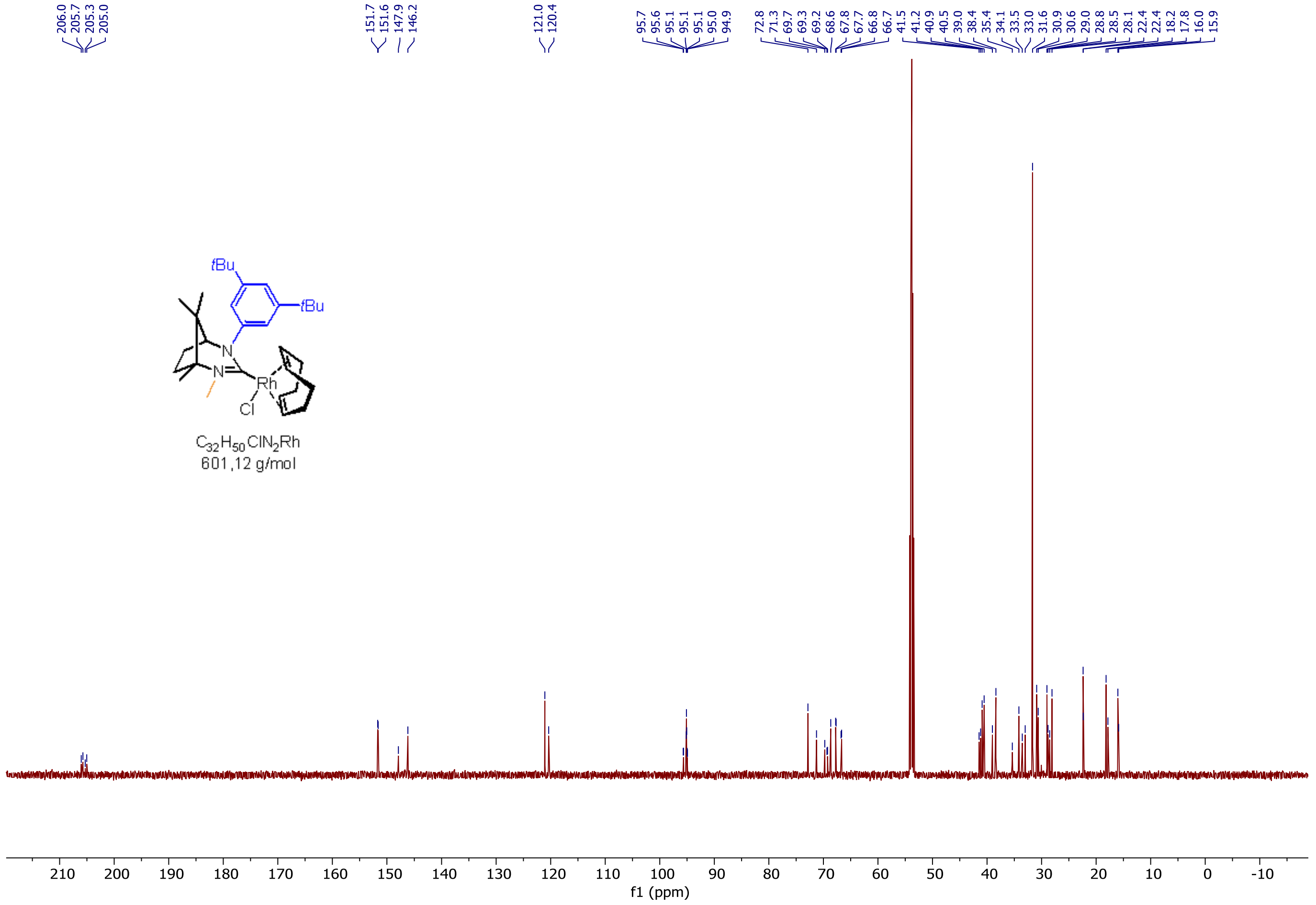

^{1}H NMR (600 MHz, CD_2Cl_2), $^{13}C\{^{1}H\}$ NMR (151 MHz, CD_2Cl_2), ^{15}N HSQC NMR (61 MHz, CD_2Cl_2) and ^{19}F NMR (337 MHz, CD_2Cl_2) and ^{31}P NMR (162 MHz, CD_2Cl_2) Analysis of **Complex Rh5ba**

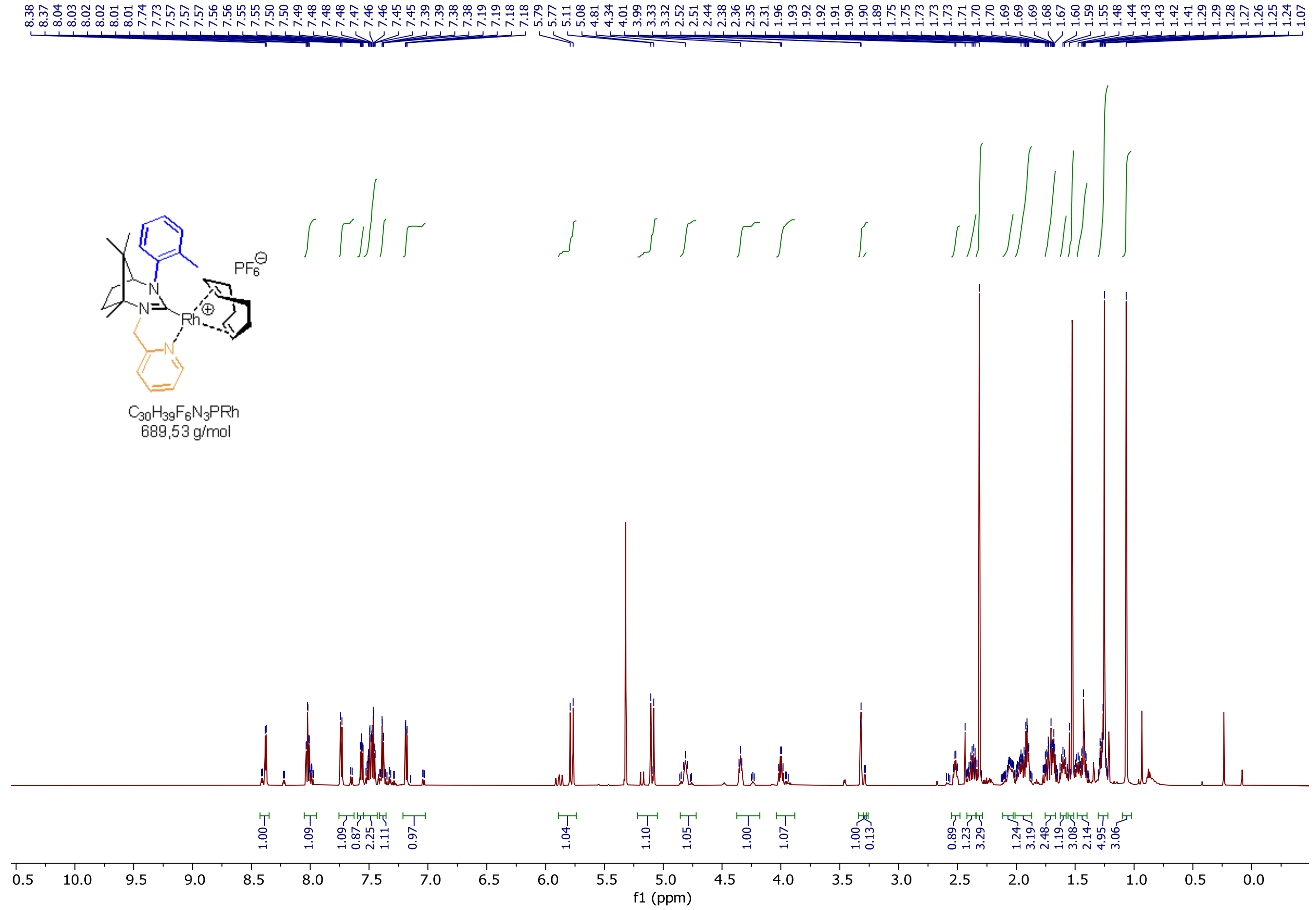

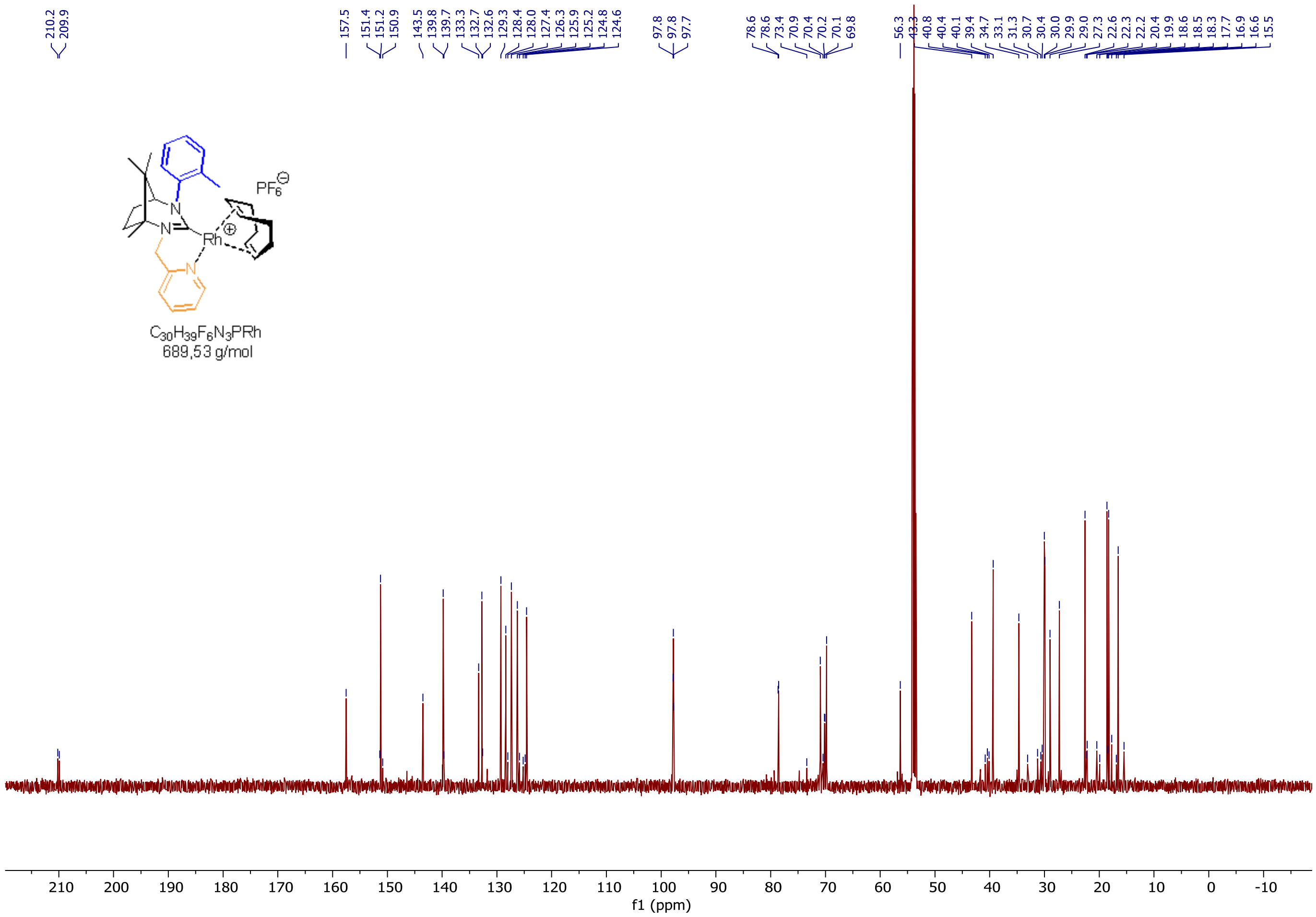

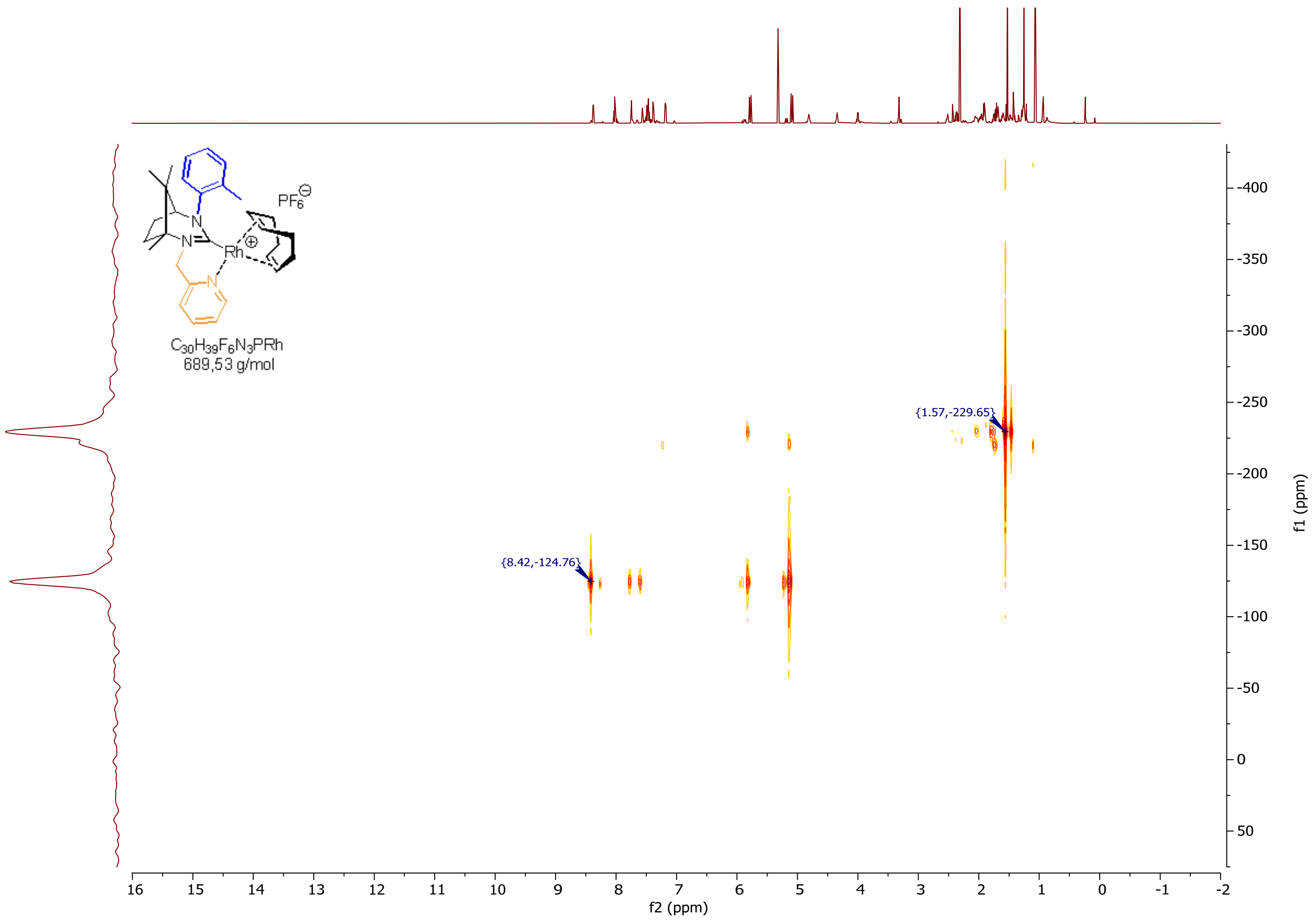

PF6
Rh
C30H39F6N3PRh
689,53 g/mol
{1.57,-229.65}
{8.42,-124.76}
f2 (ppm)
f1 (ppm)

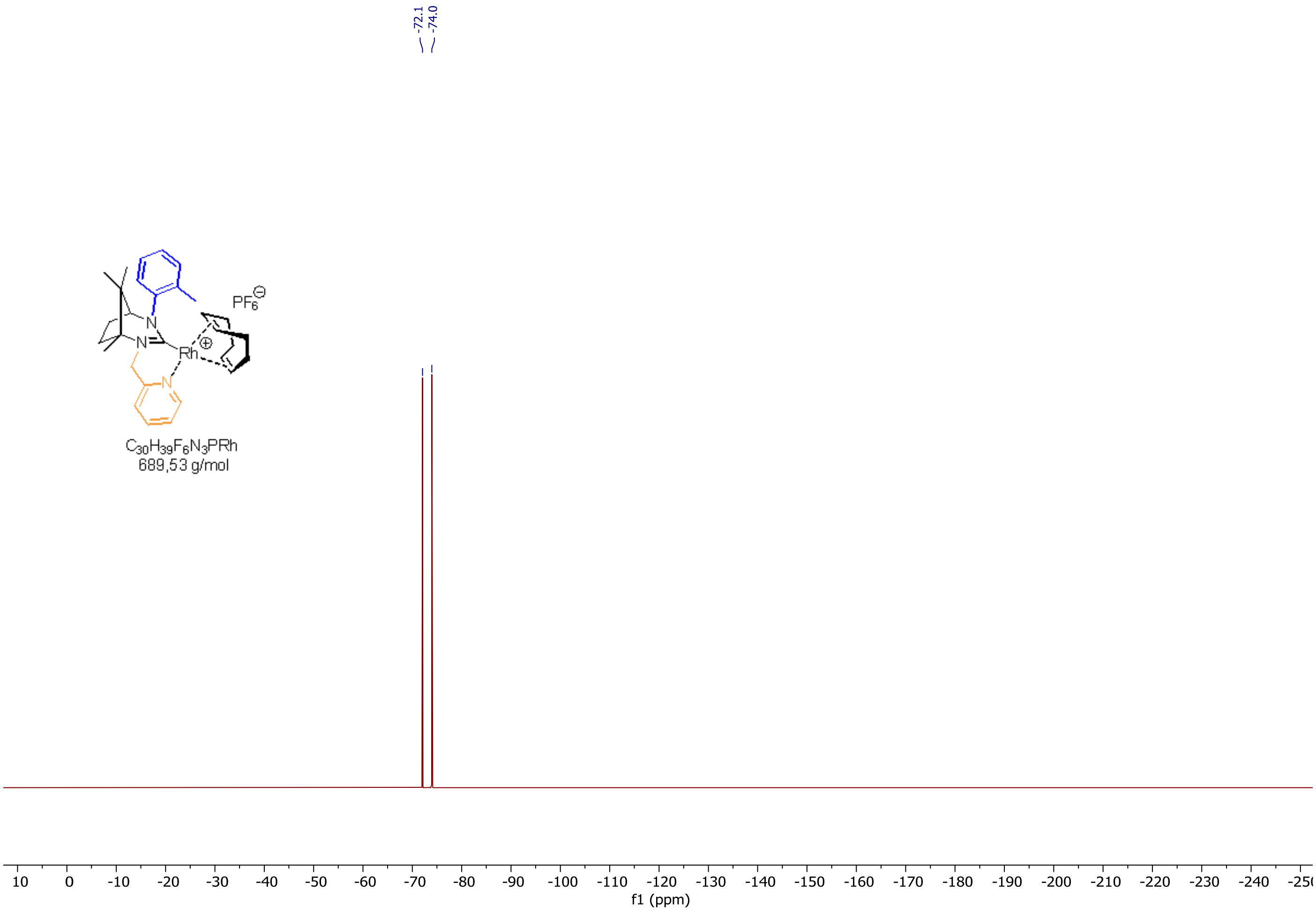

-72.1
-74.0
PF6
Rh
C30H39F6N3PRh
689,53 g/mol
f1 (ppm)

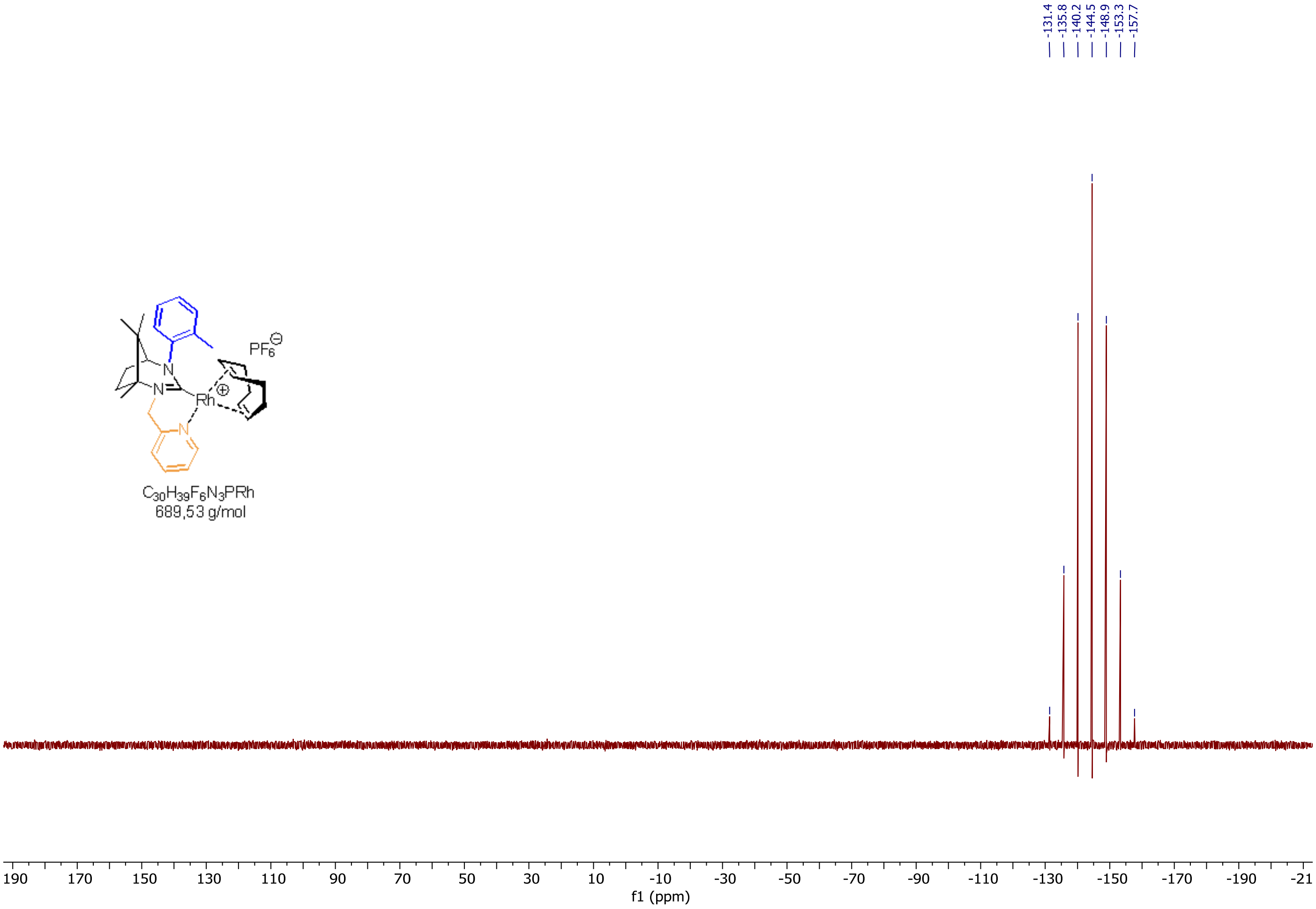

$C_{30}H_{39}F_6N_3PRh$
689,53 g/mol

^1H NMR (600 MHz, $CDCl_3$), $^{13}C\{^1H\}$ NMR (151 MHz, $CDCl_3$) and ^{15}N HSQC NMR (61 MHz, $CDCl_3$) Analysis of **Complex Rh5bb**

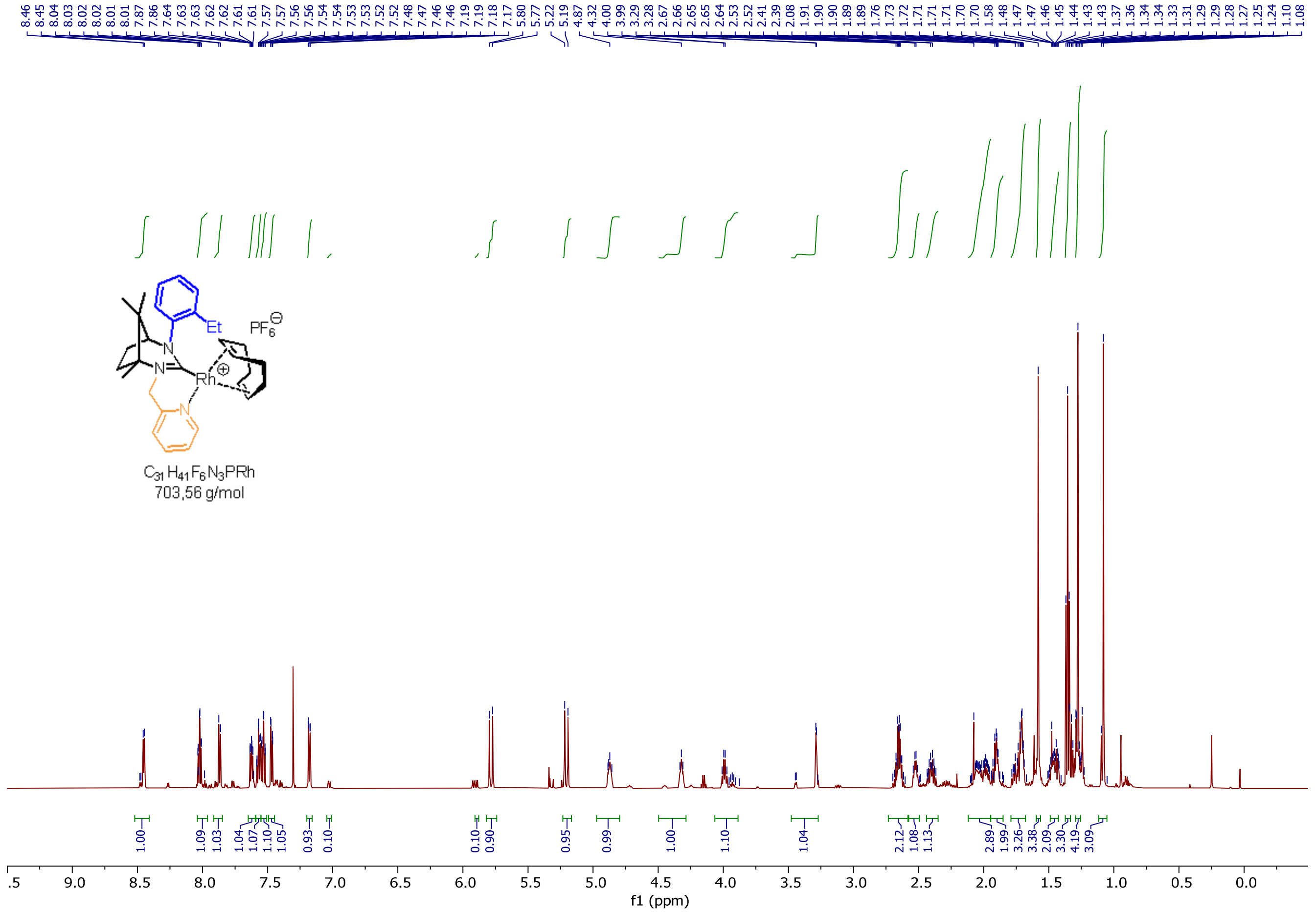

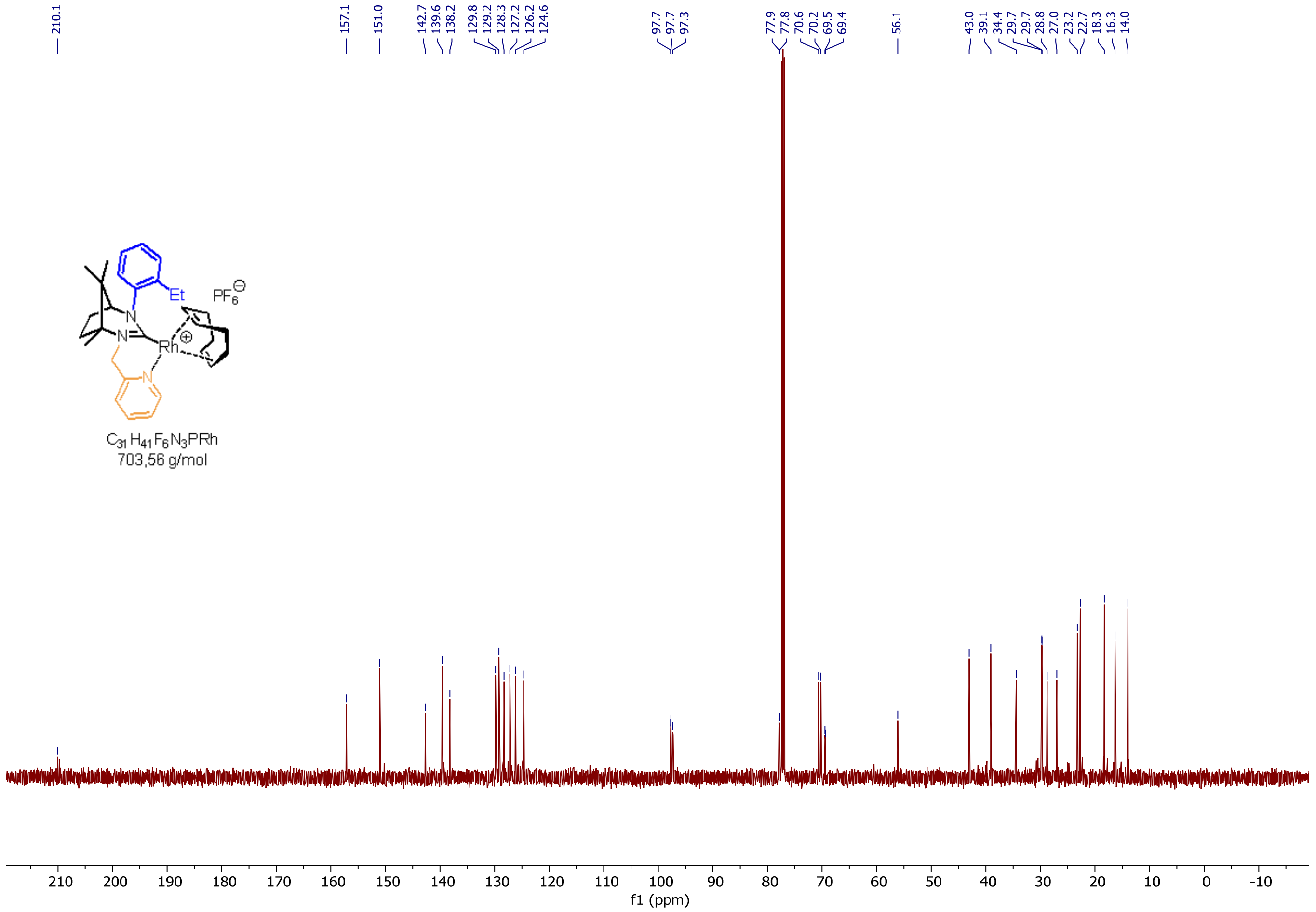

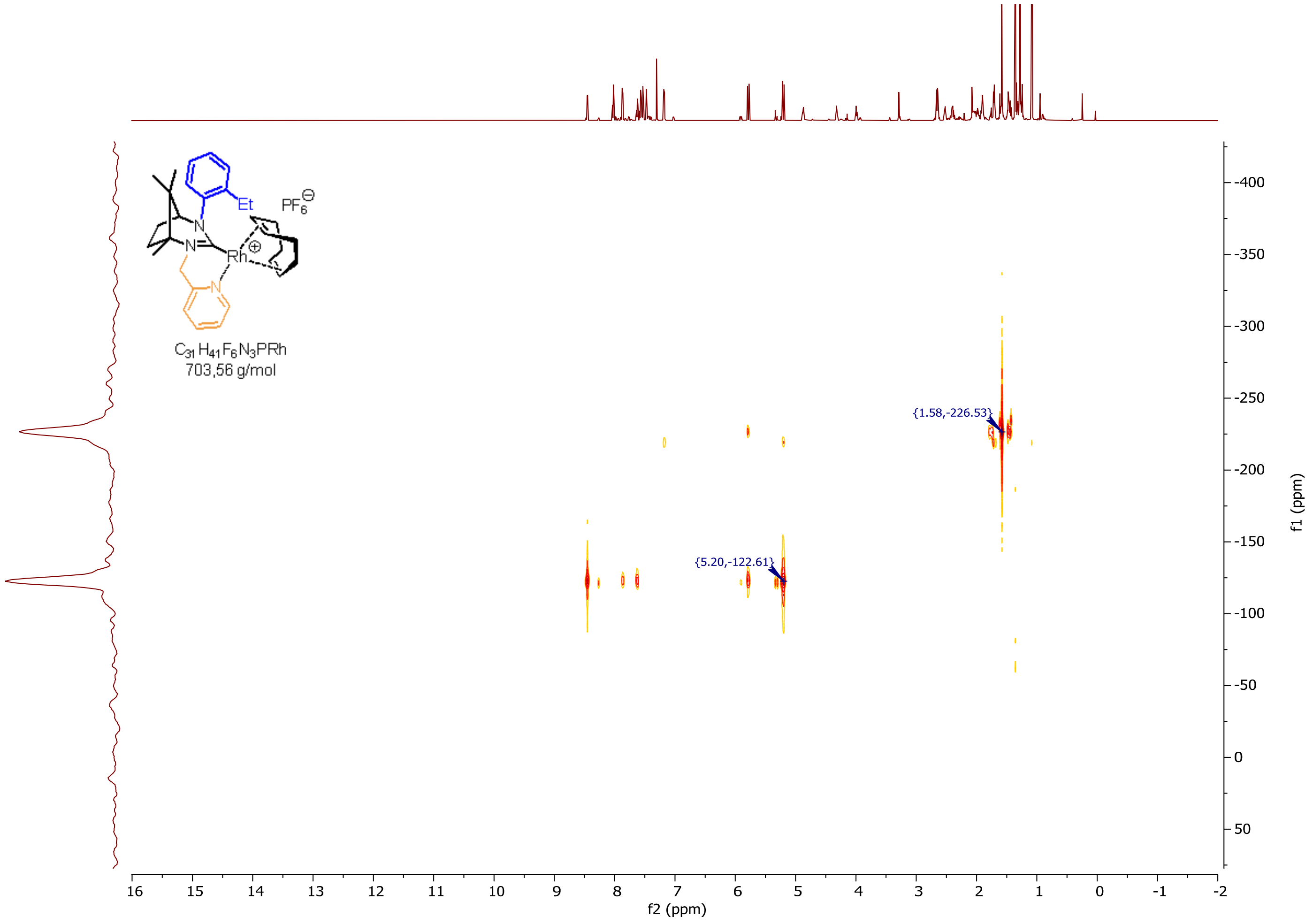

Et
PF6
Rh
N
N
C31 H41 F6 N3 PRh
703,56 g/mol
{1.58,-226.53}
{5.20,-122.61}
f1 (ppm)
f2 (ppm)

^{1}H NMR (600 MHz, $CDCl_3$), $^{13}C\{^{1}H\}$ NMR (151 MHz, $CDCl_3$) and ^{15}N HSQC NMR (61 MHz, $CDCl_3$) Analysis of **Complex Rh5bc**

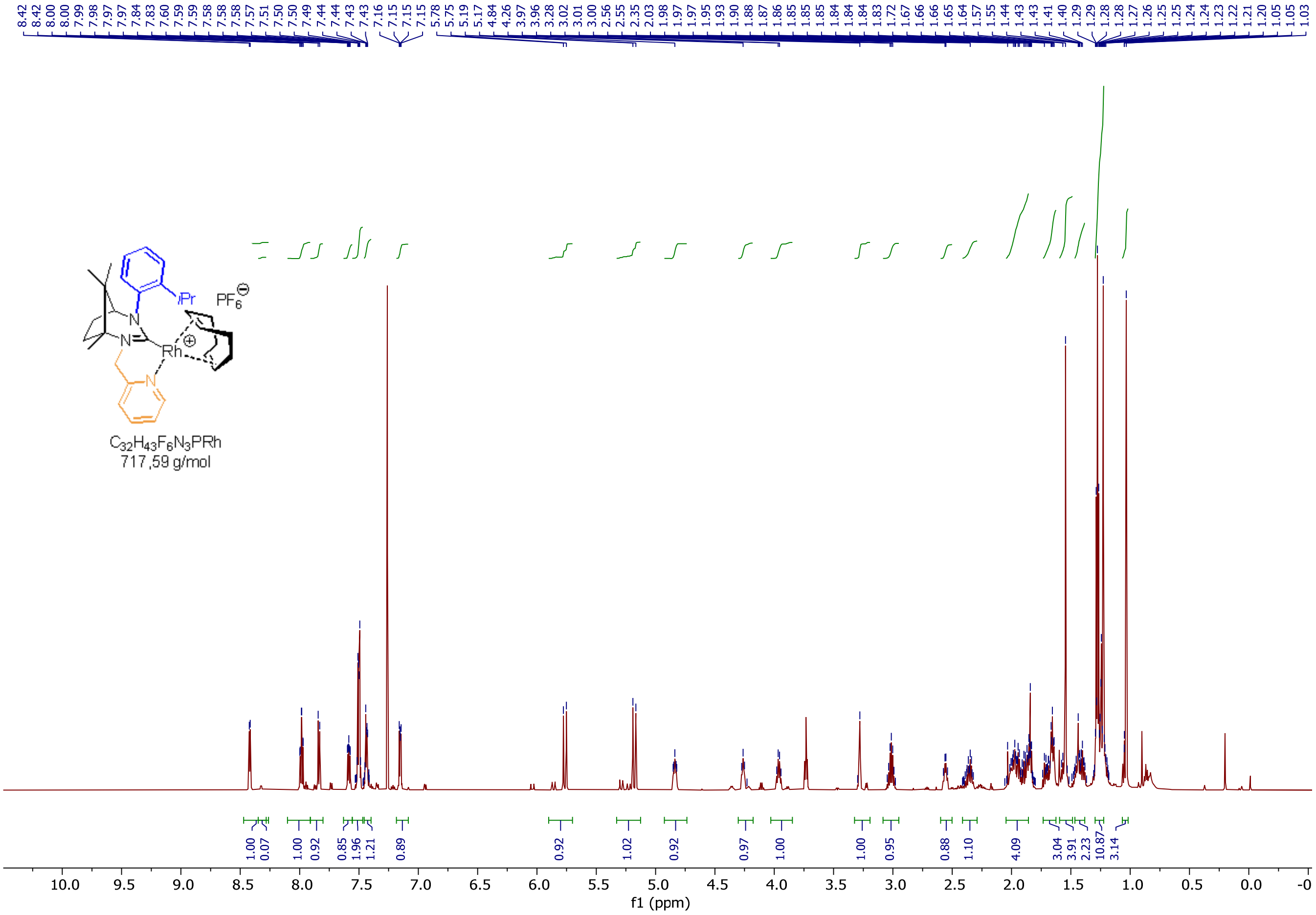

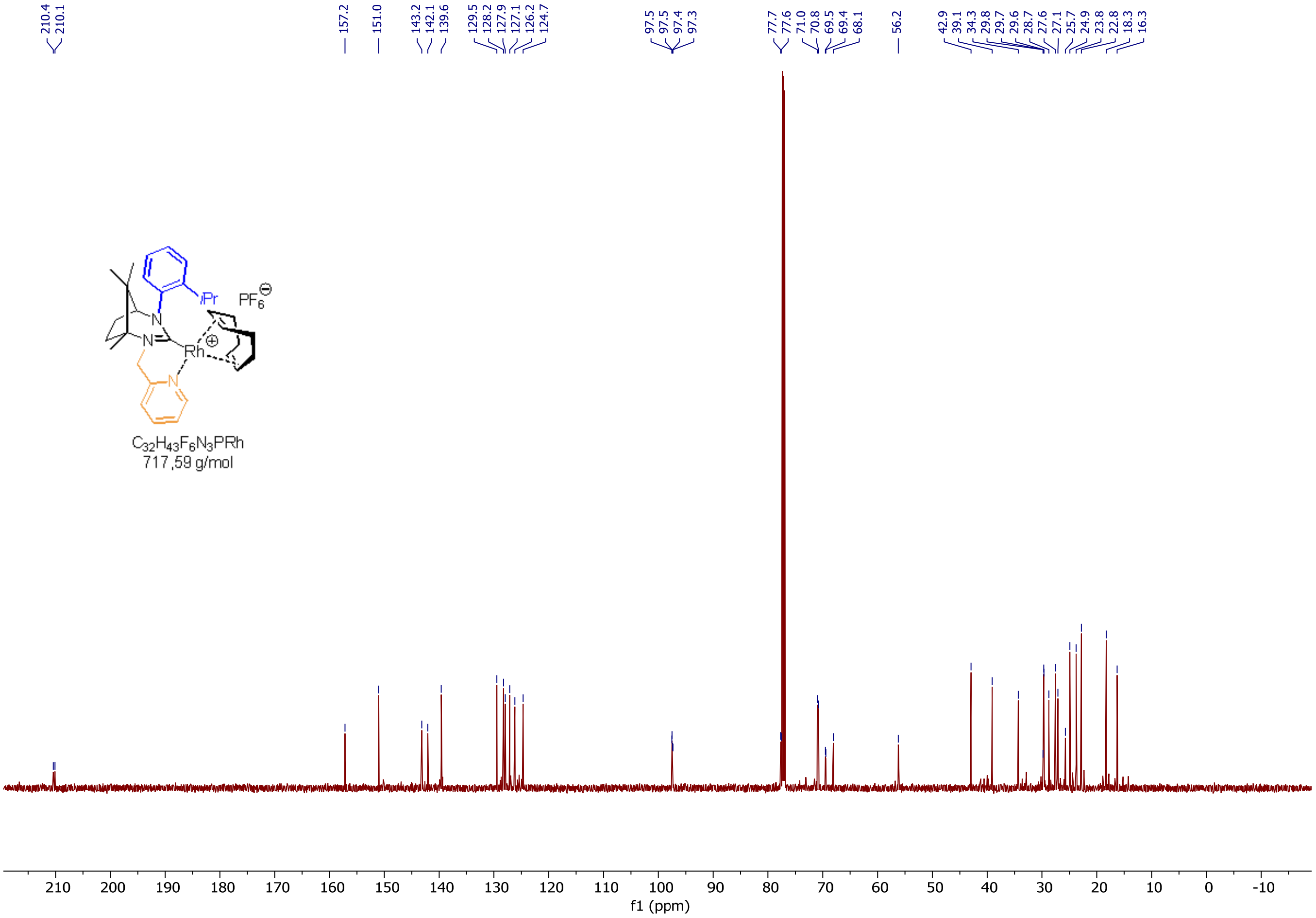

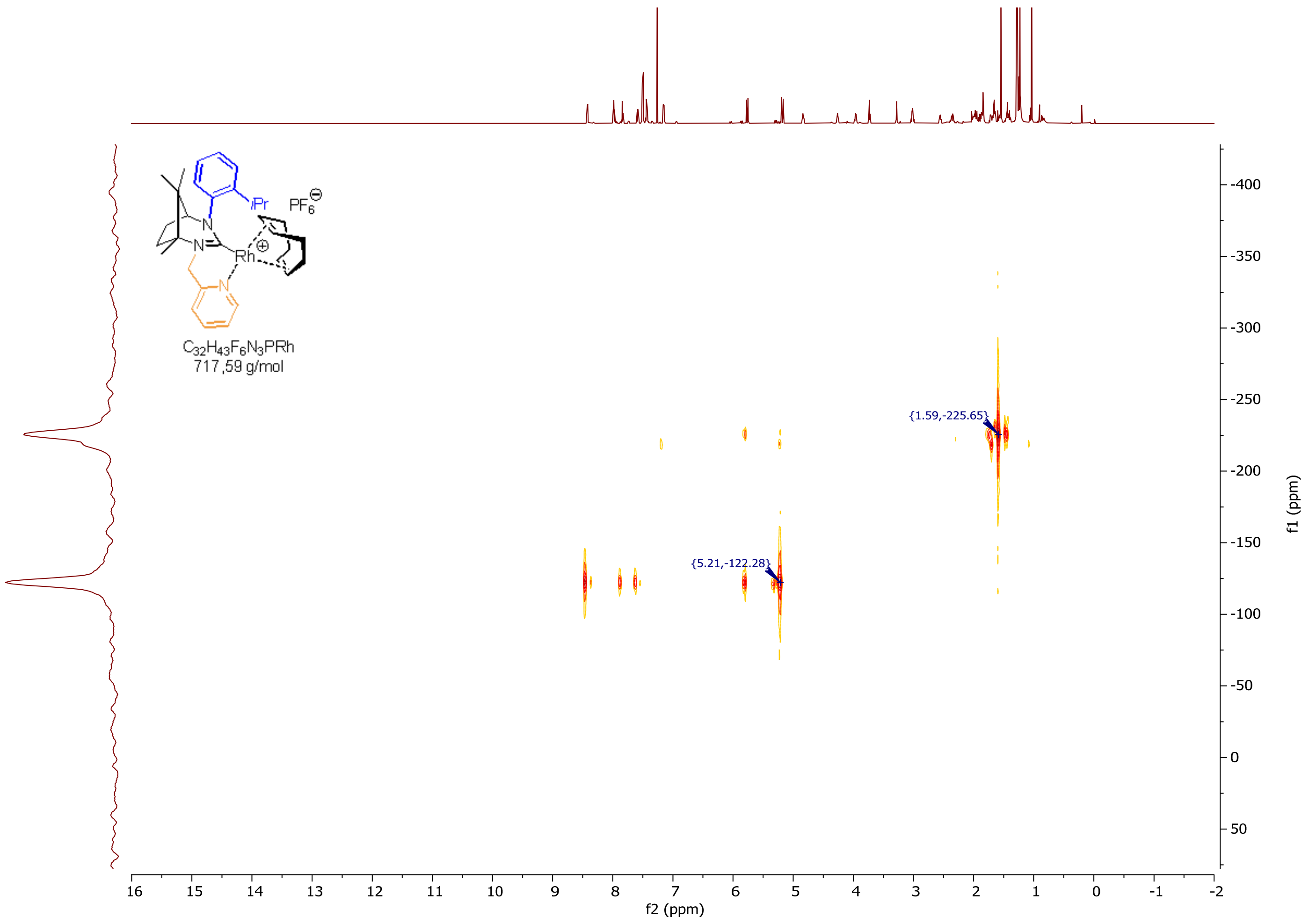

iPr
PF6
Rh
N
C32H43F6N3PRh
717,59 g/mol
{1.59,-225.65}
{5.21,-122.28}
f2 (ppm)
f1 (ppm)

^{1}H NMR (600 MHz, $CDCl_3$), $^{13}C\{^{1}H\}$ NMR (151 MHz, $CDCl_3$) and ^{15}N HSQC NMR (61 MHz, $CDCl_3$) Analysis of **Complex Rh5bd**

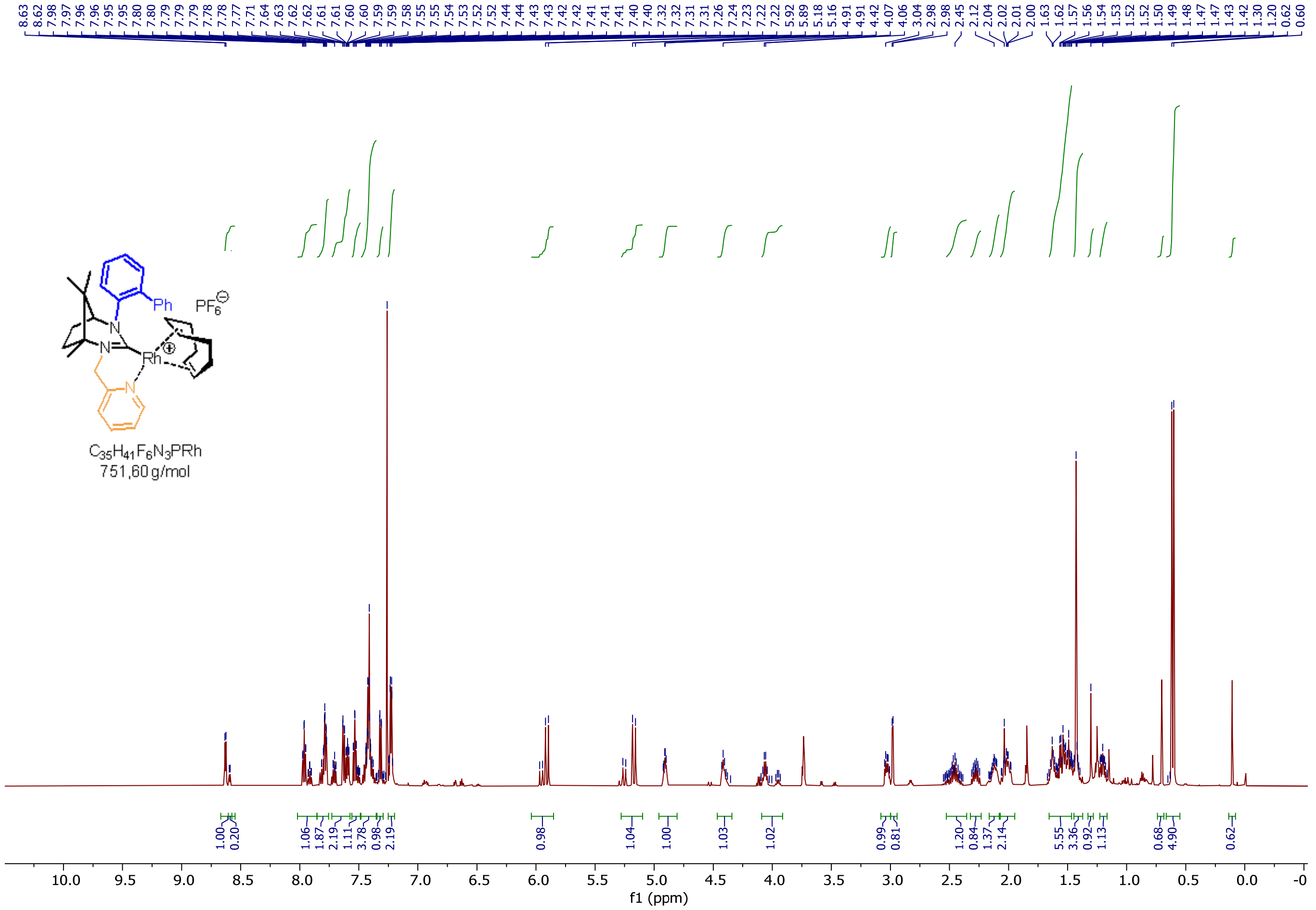

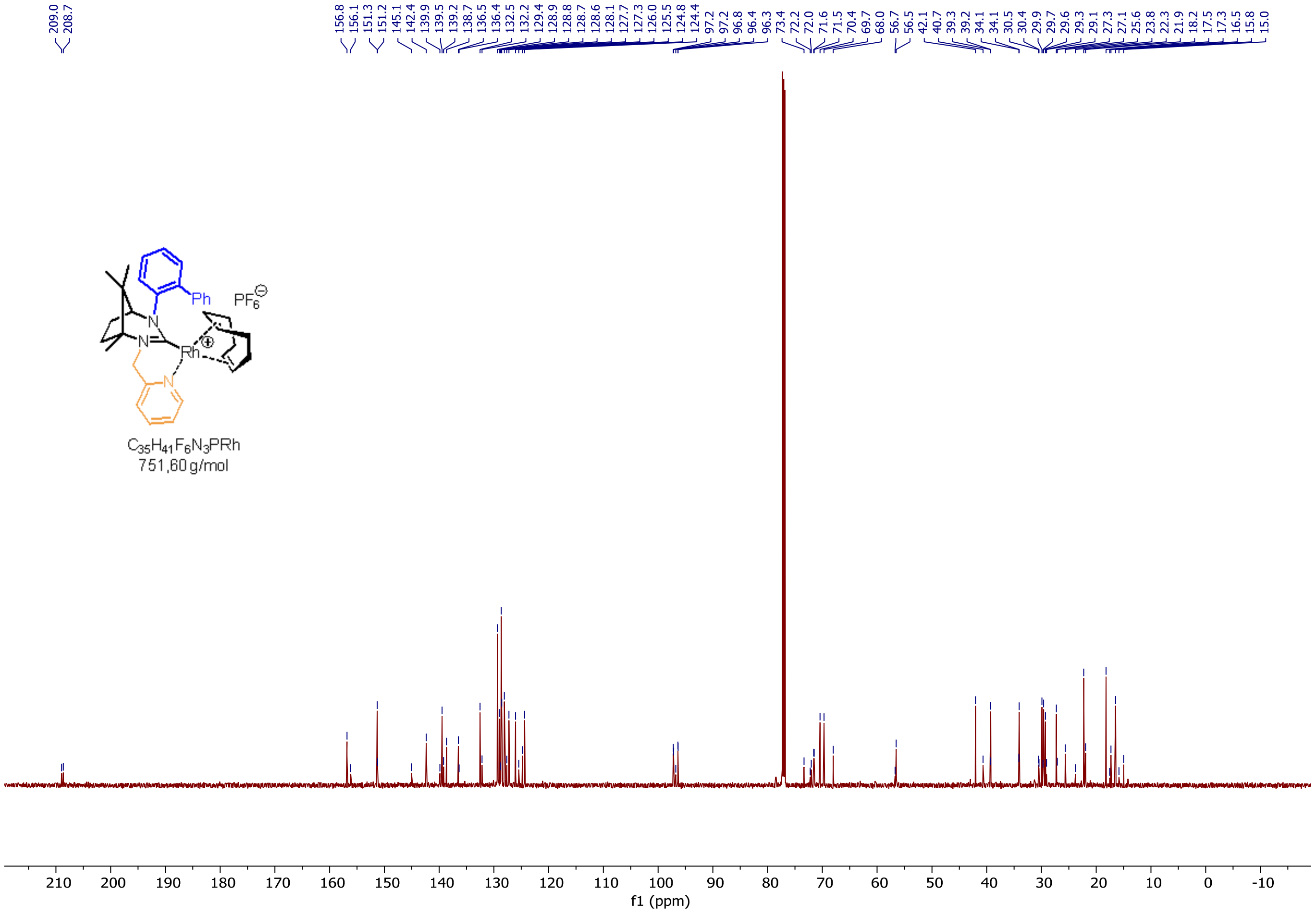

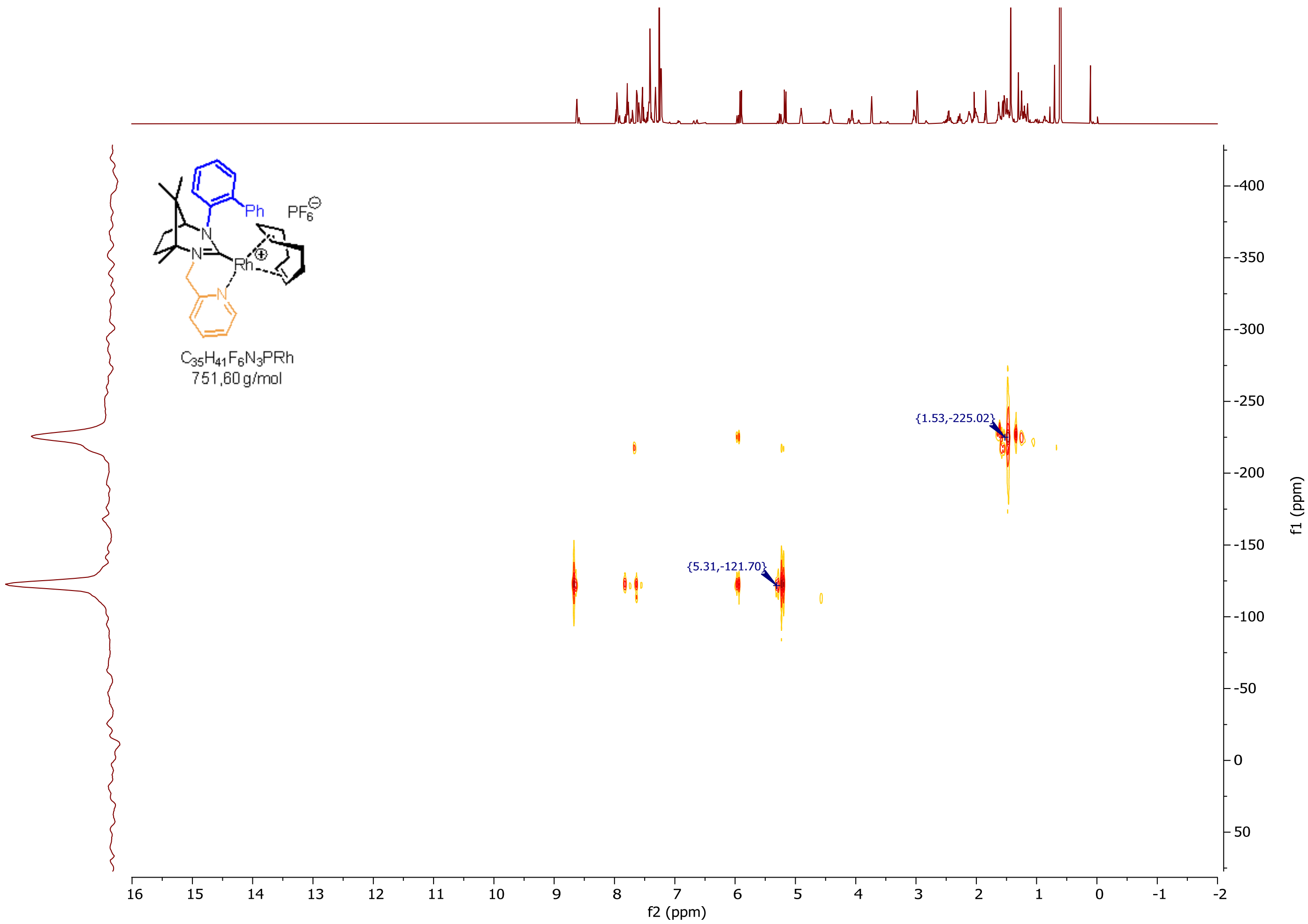

PF6
C35H41F6N3PRh
751,60 g/mol
{1.53,-225.02}
{5.31,-121.70}
f2 (ppm)
f1 (ppm)

^{1}H NMR (600 MHz, $CDCl_3$), ^{13}C{^{1}H} NMR (151 MHz, $CDCl_3$), ^{15}N HSQC NMR (61 MHz, $CDCl_3$) and ^{19}F NMR (337 MHz, $CDCl_3$) Analysis of **Complex Rh5be**

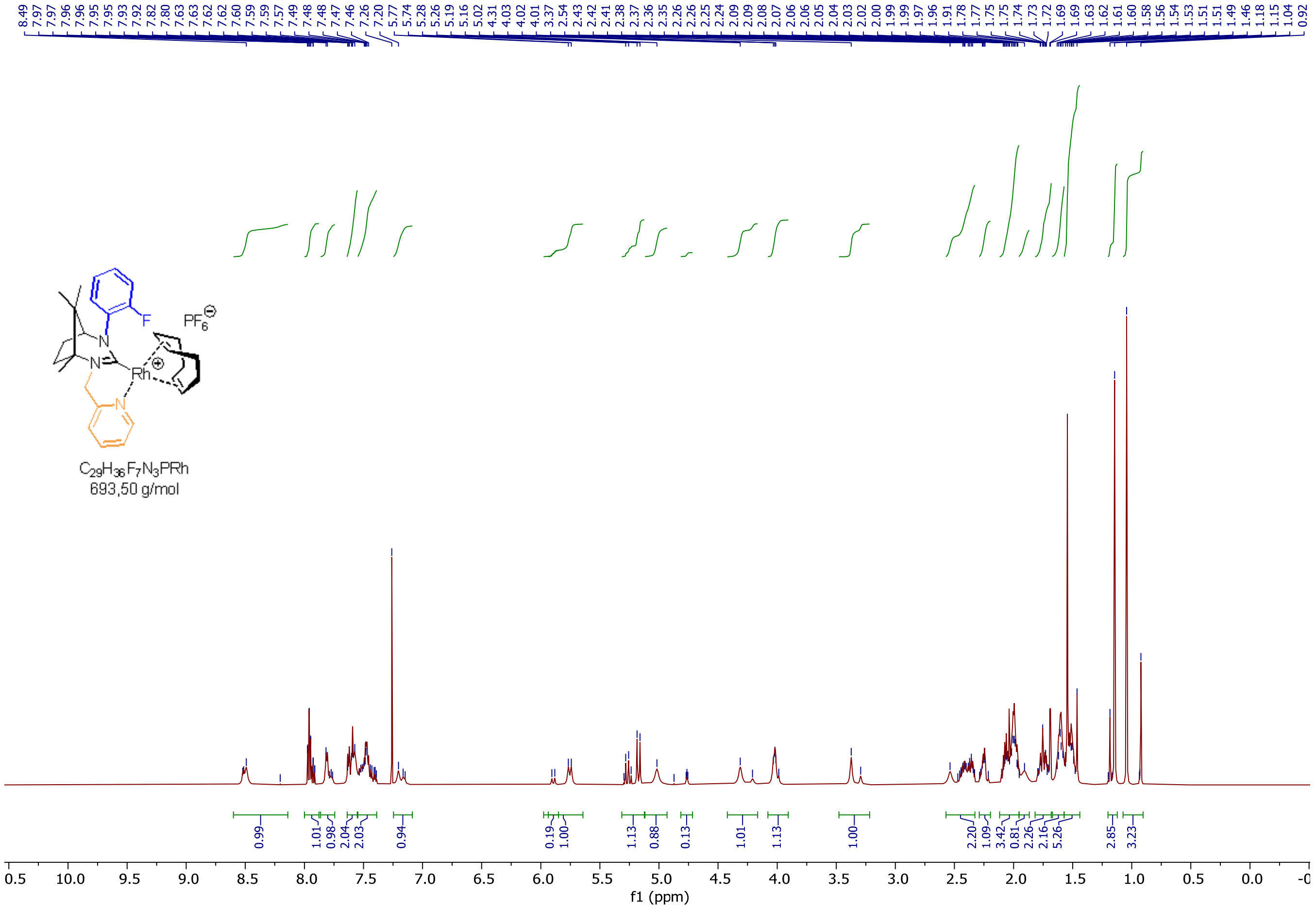

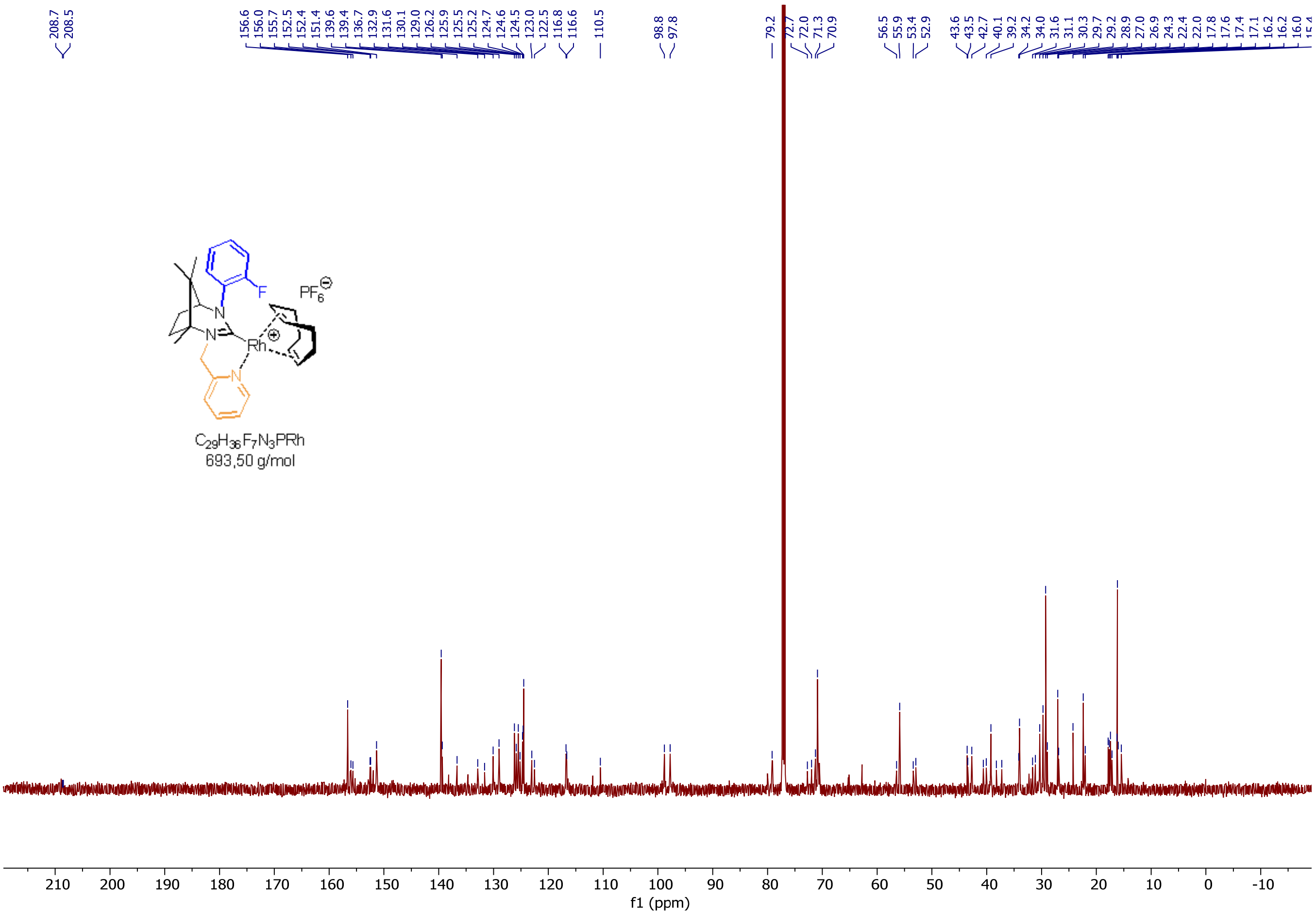

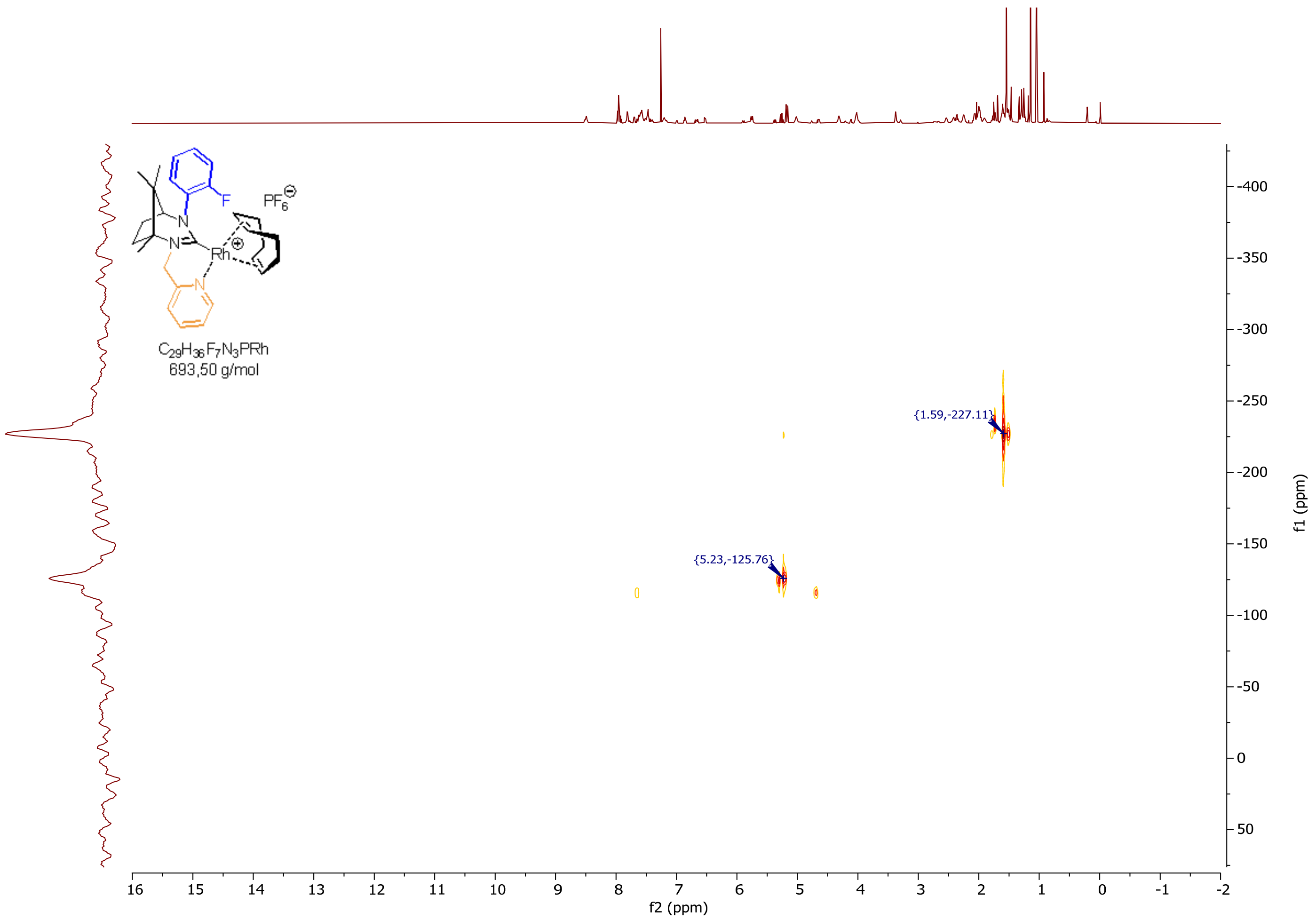
PF6
Rh
N
F
C29H36F7N3PRh
693,50 g/mol
{1.59,-227.11}
{5.23,-125.76}
f2 (ppm)
f1 (ppm)

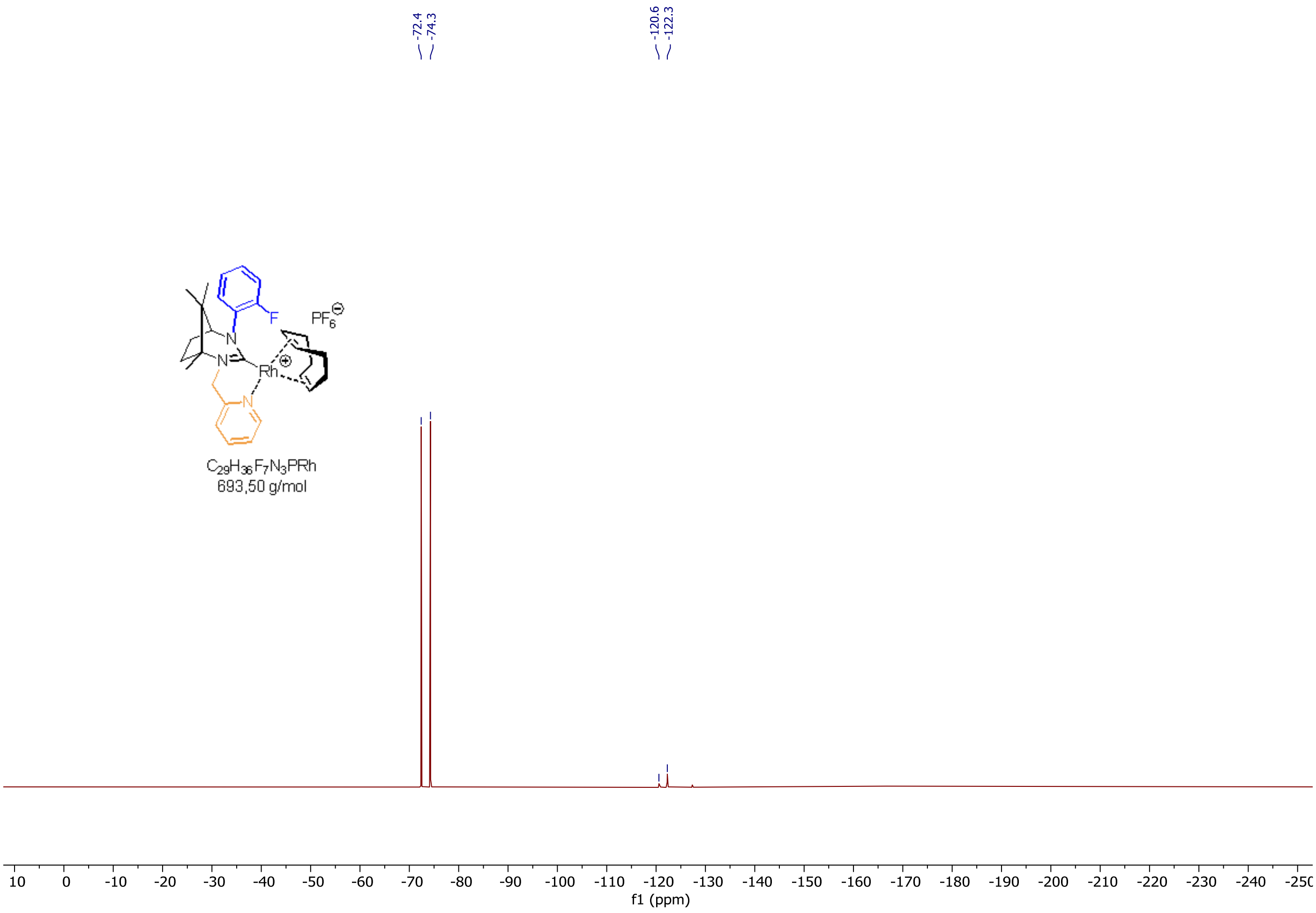
-72.4
-74.3
-120.6
-122.3
PF6
Rh
N
F
C29H36F7N3PRh
693,50 g/mol
f1 (ppm)

^{1}H NMR (600 MHz, $CDCl_3$), $^{13}C\{^{1}H\}$ NMR (151 MHz, $CDCl_3$) and ^{15}N HSQC NMR (61 MHz, $CDCl_3$) Analysis of **Complex Rh5bf**

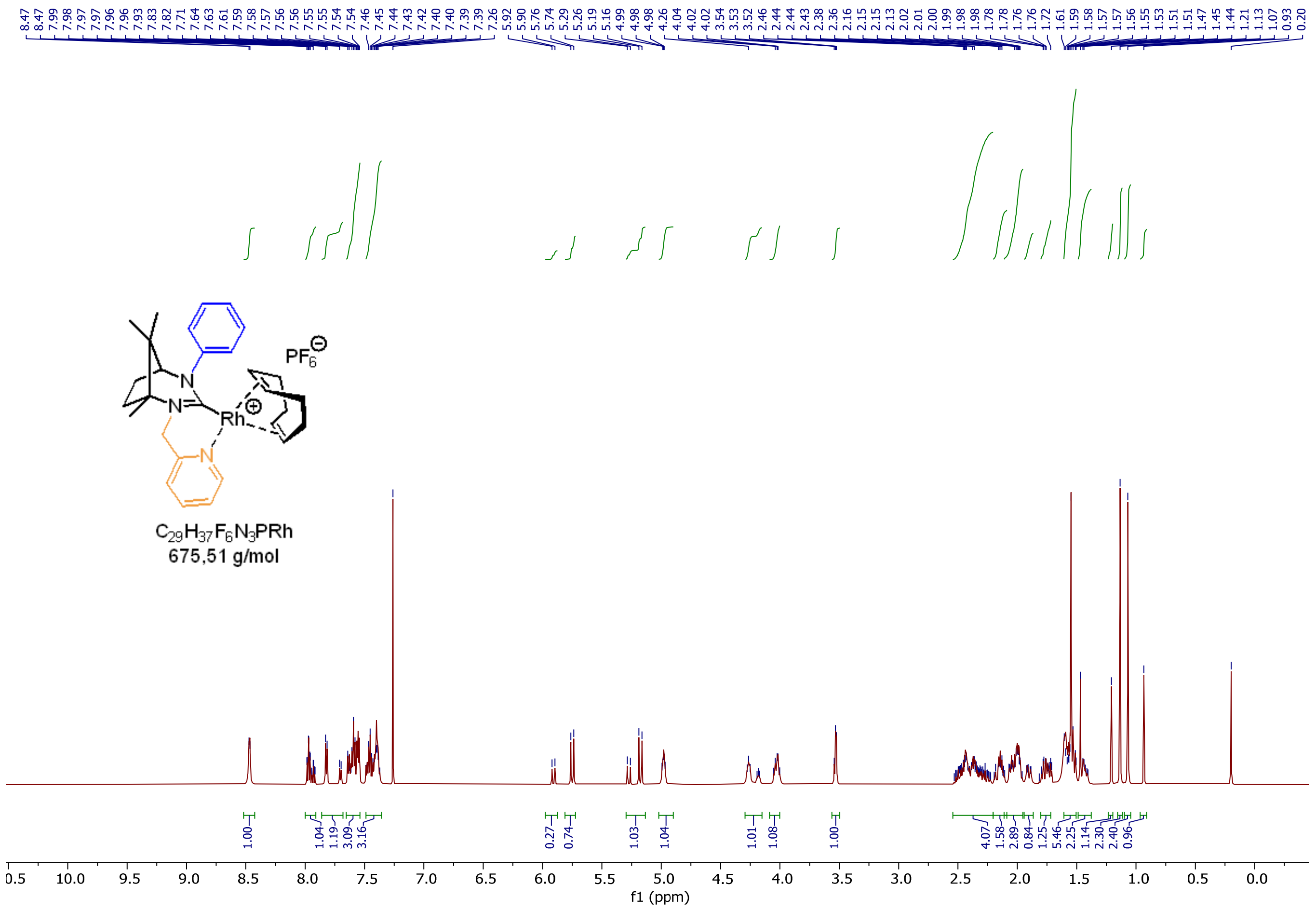

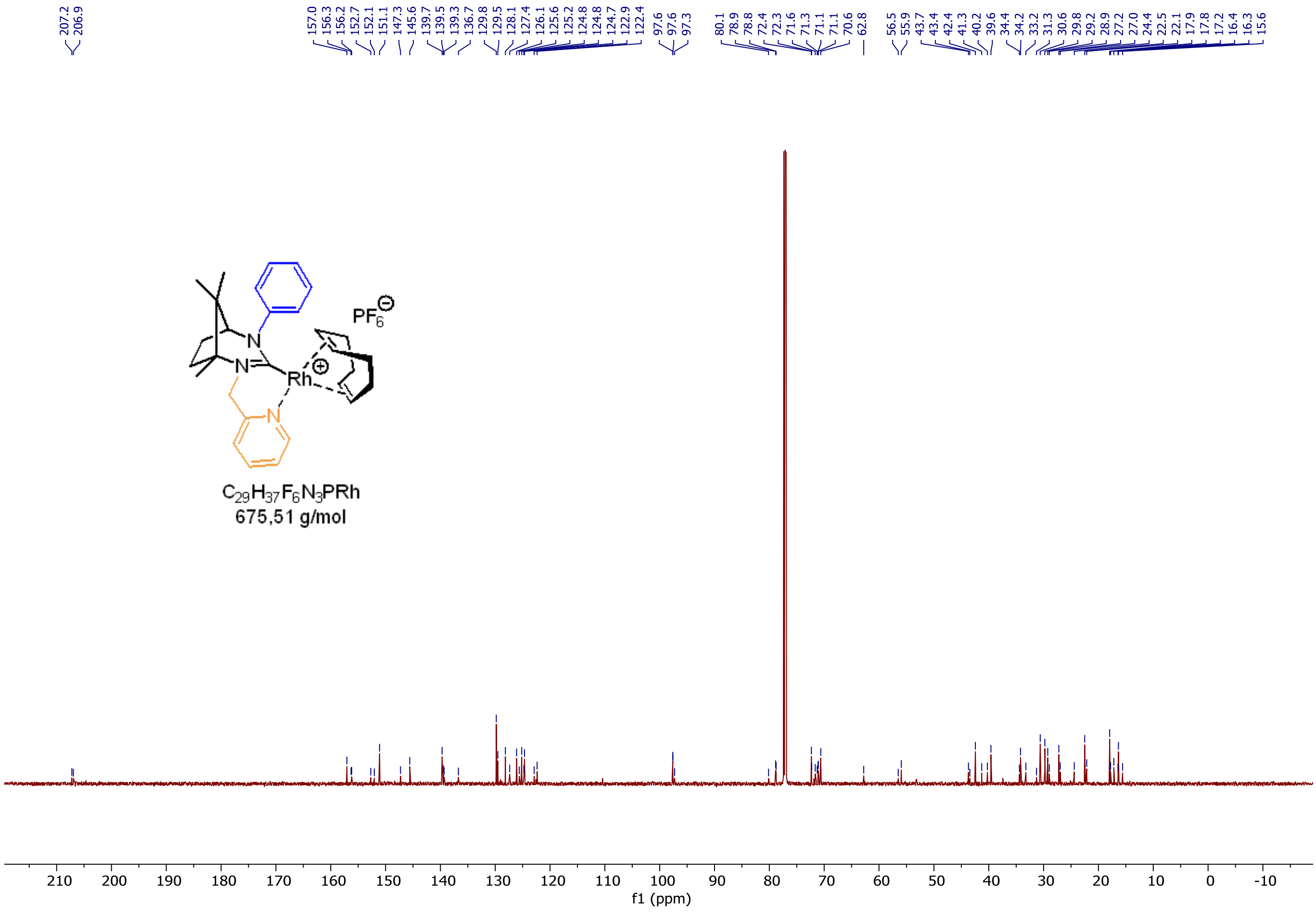

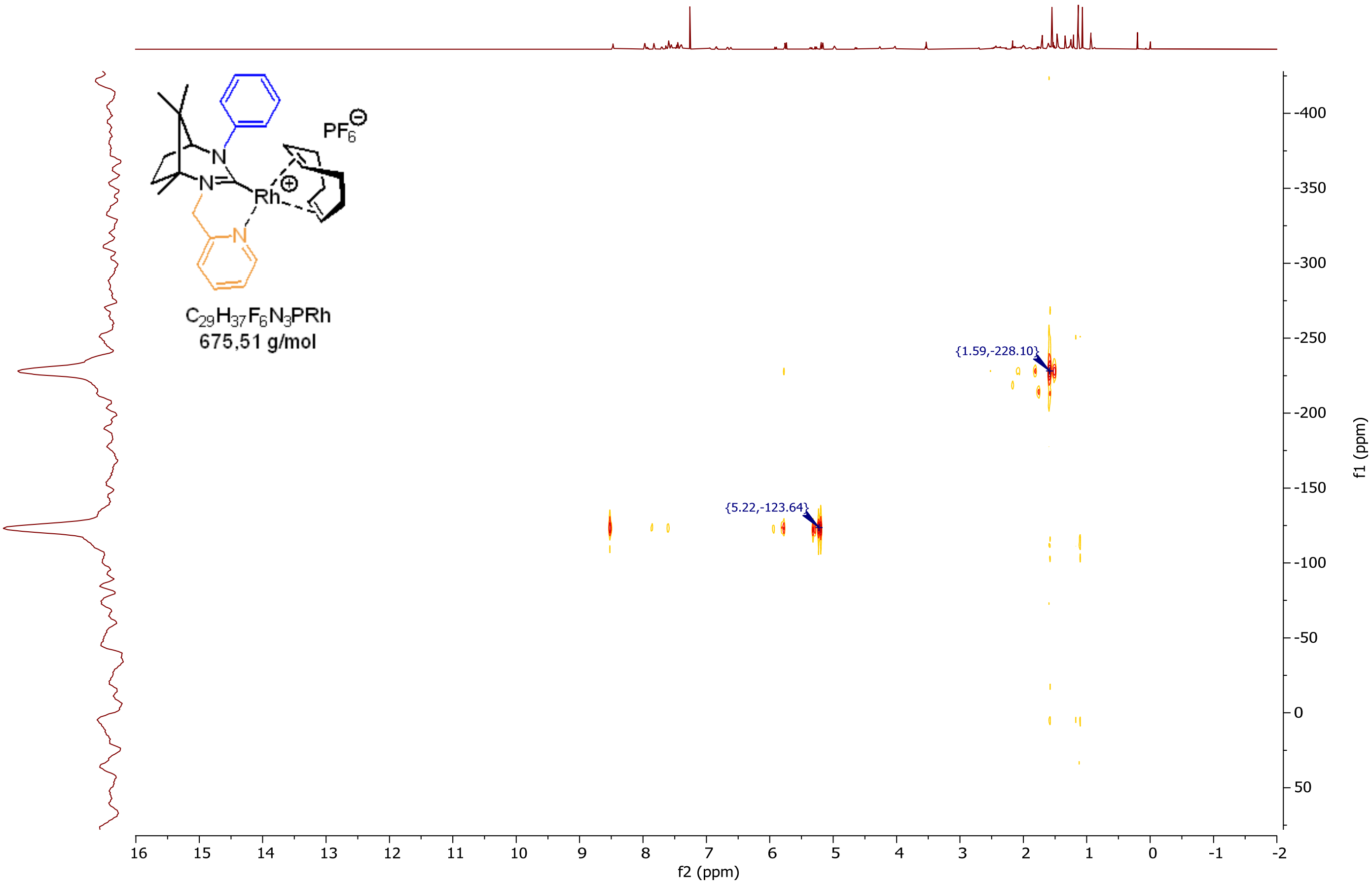
PF6
Rh
N
N
N
C29H37F6N3PRh
675,51 g/mol
{1.59,-228.10}
{5.22,-123.64}
f2 (ppm)
f1 (ppm)

^{1}H NMR (600 MHz, $CDCl_3$), ^{13}C{^{1}H} NMR (151 MHz, $CDCl_3$) and ^{15}N HSQC NMR (61 MHz, $CDCl_3$) Analysis of **Complex Rh5bg**

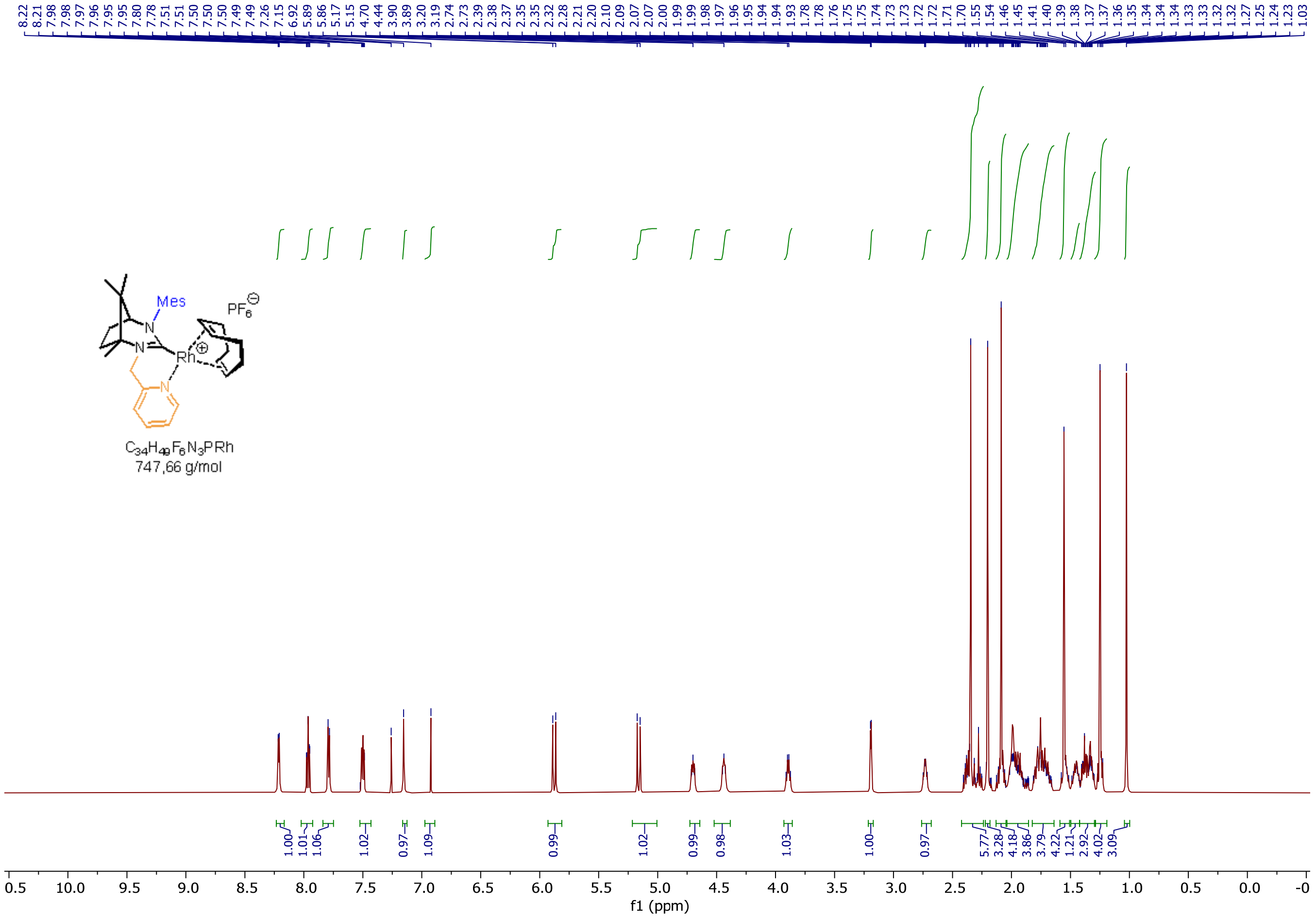

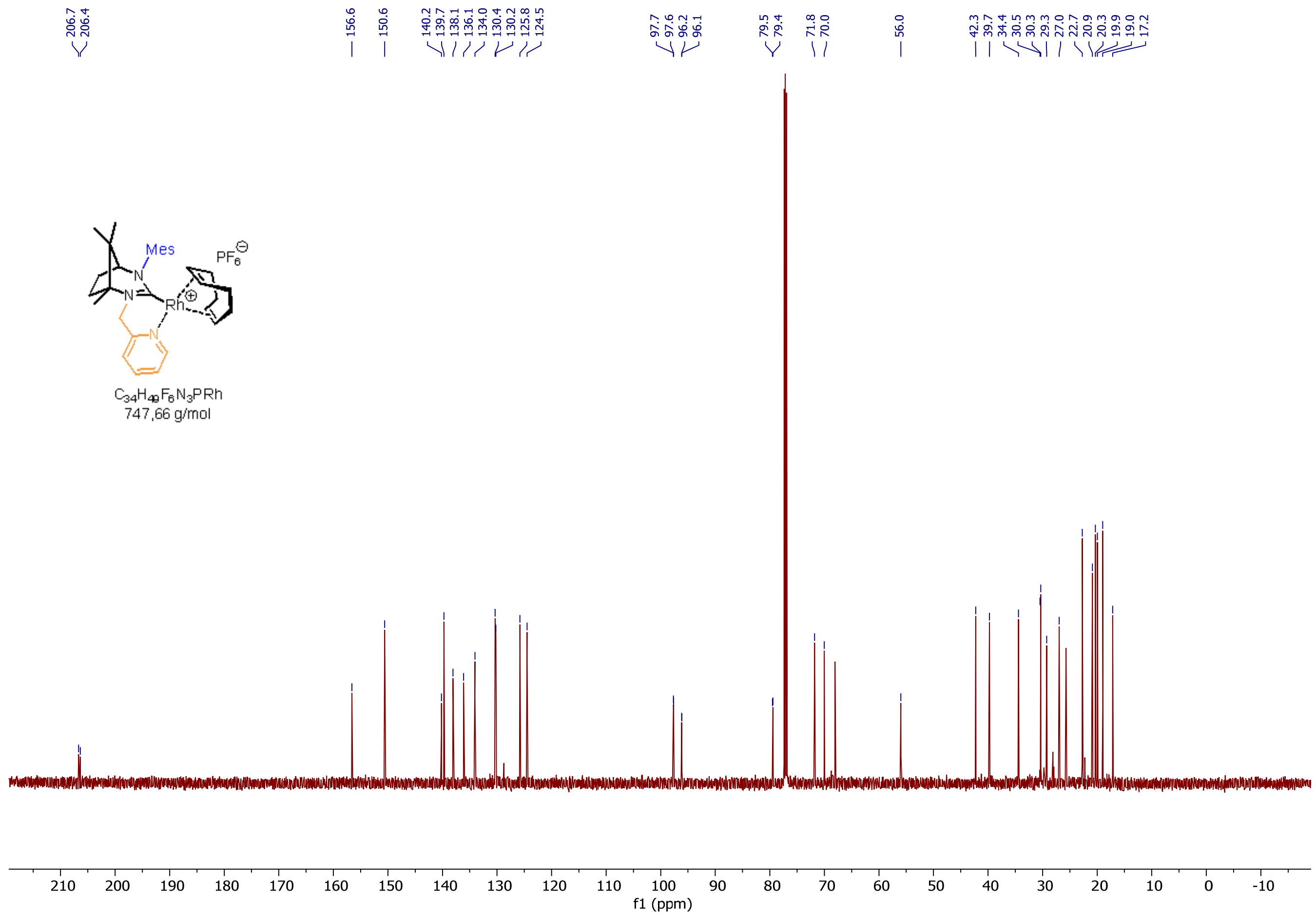

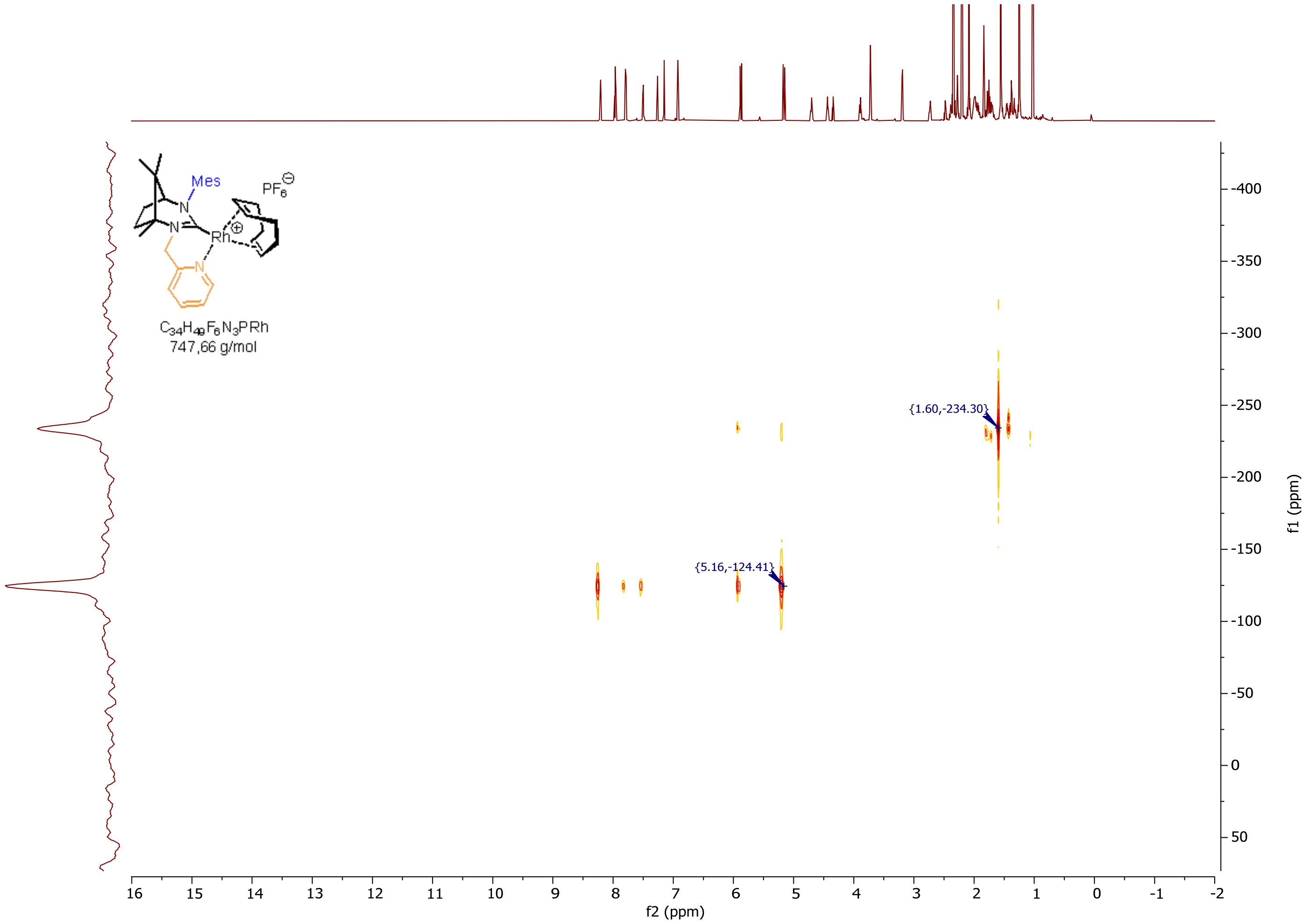
Mes
$PF_6^{\ominus}$
N
N
Rh
N
$C_{34}H_{49}F_6N_3PRh$
747,66 g/mol
{1.60,-234.30}
{5.16,-124.41}
f1 (ppm)
f2 (ppm)

^{1}H NMR (600 MHz, $CDCl_3$), $^{13}C\{^{1}H\}$ NMR (151 MHz, $CDCl_3$) and ^{15}N HSQC NMR (61 MHz, $CDCl_3$) Analysis of **Complex Rh5bh**

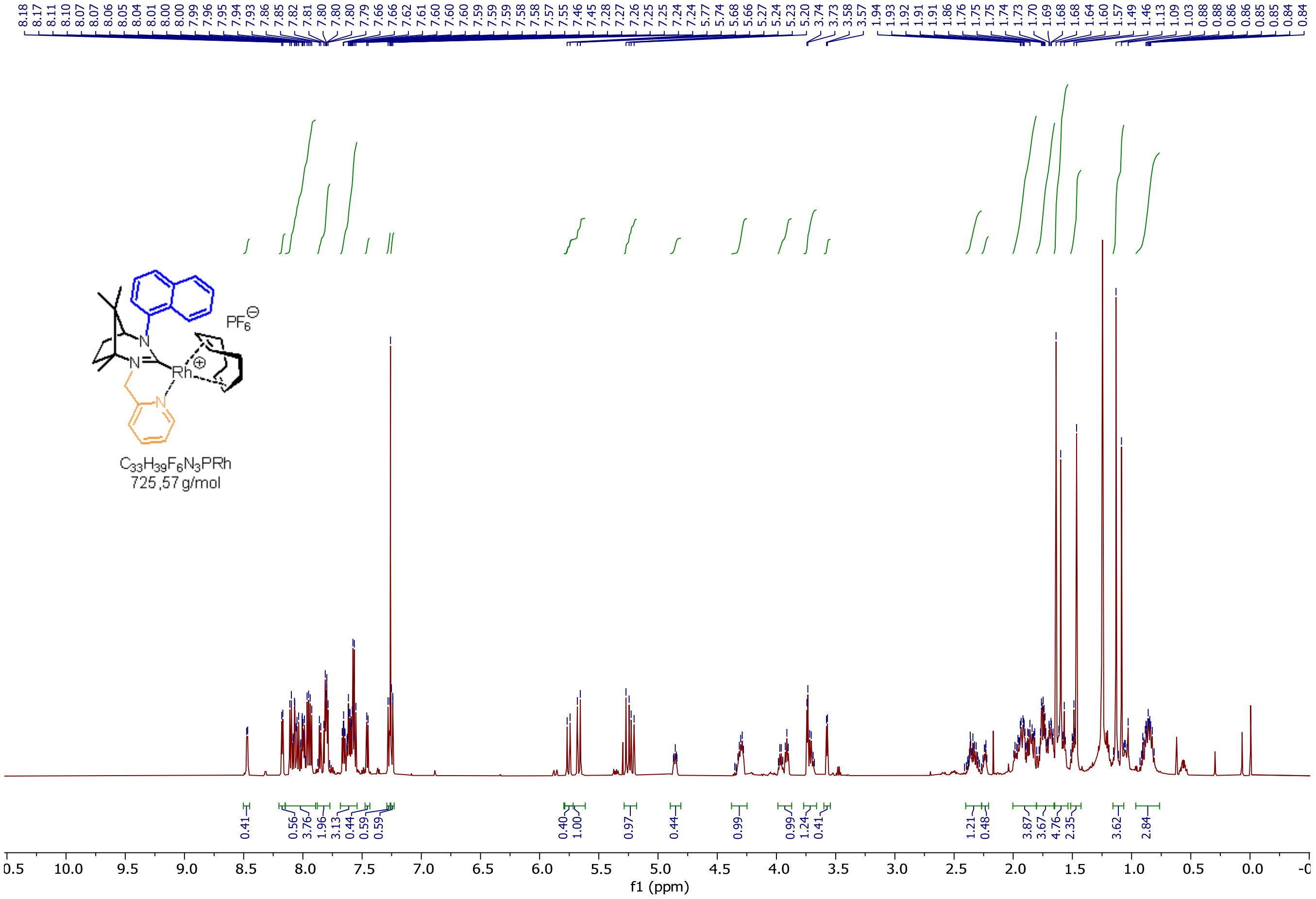

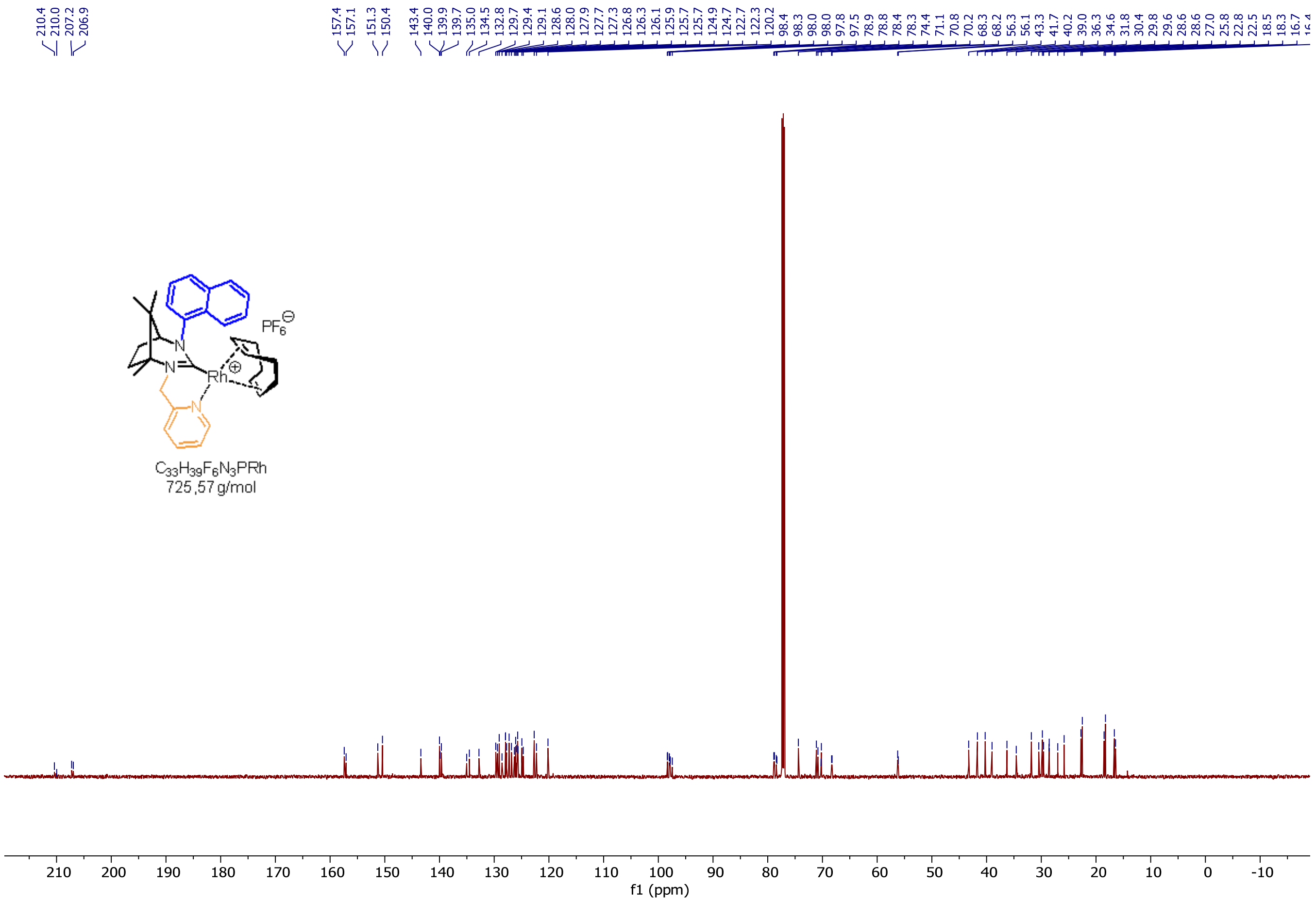

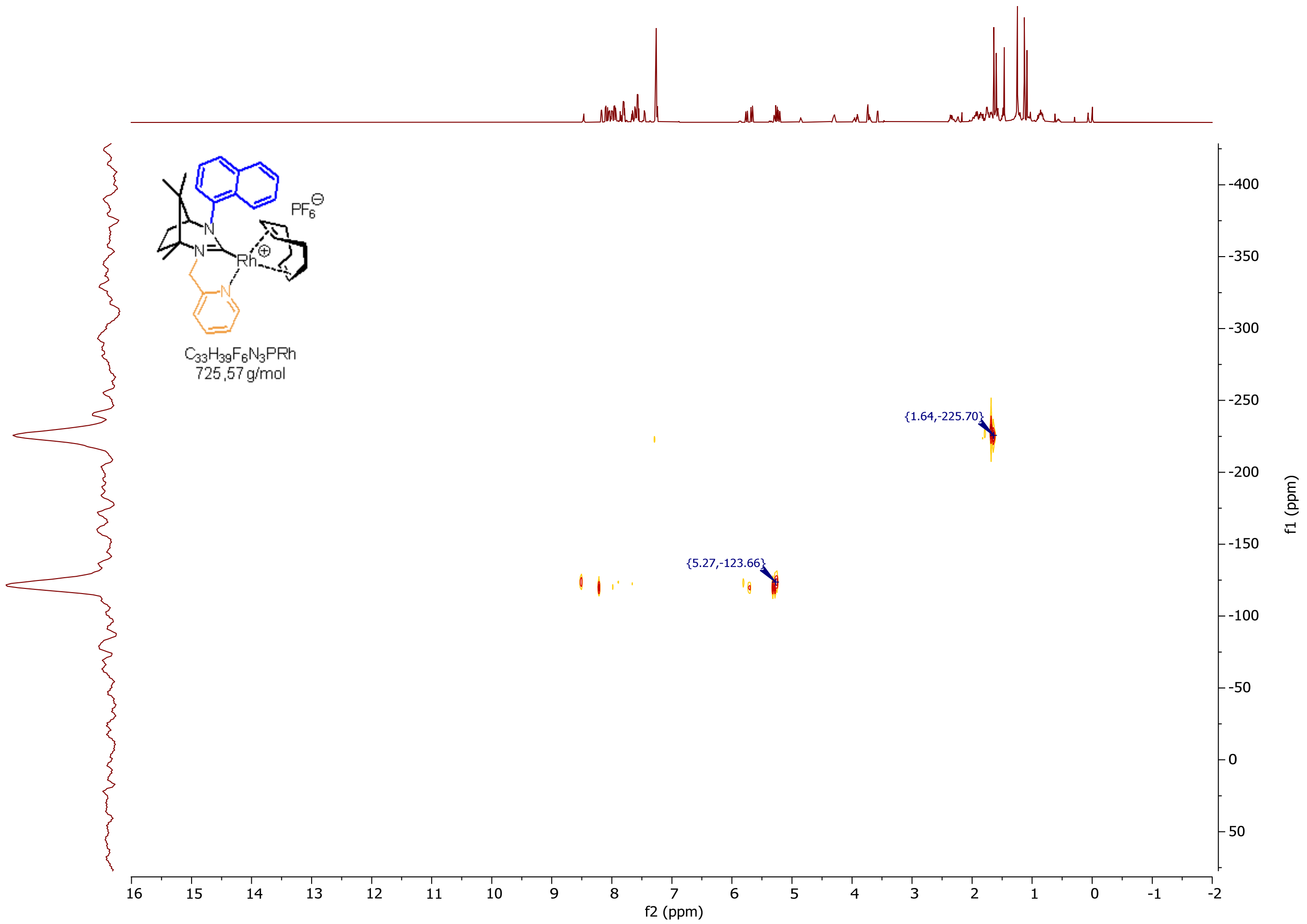

PF6
Rh
N
N
C33H39F6N3PRh
725,57 g/mol
{1.64,-225.70}
{5.27,-123.66}
f1 (ppm)
f2 (ppm)

^{1}H NMR (600 MHz, $CDCl_3$), $^{13}C\{^{1}H\}$ NMR (151 MHz, $CDCl_3$) and ^{15}N HSQC NMR (61 MHz, $CDCl_3$) Analysis of **Complex Rh5bh**

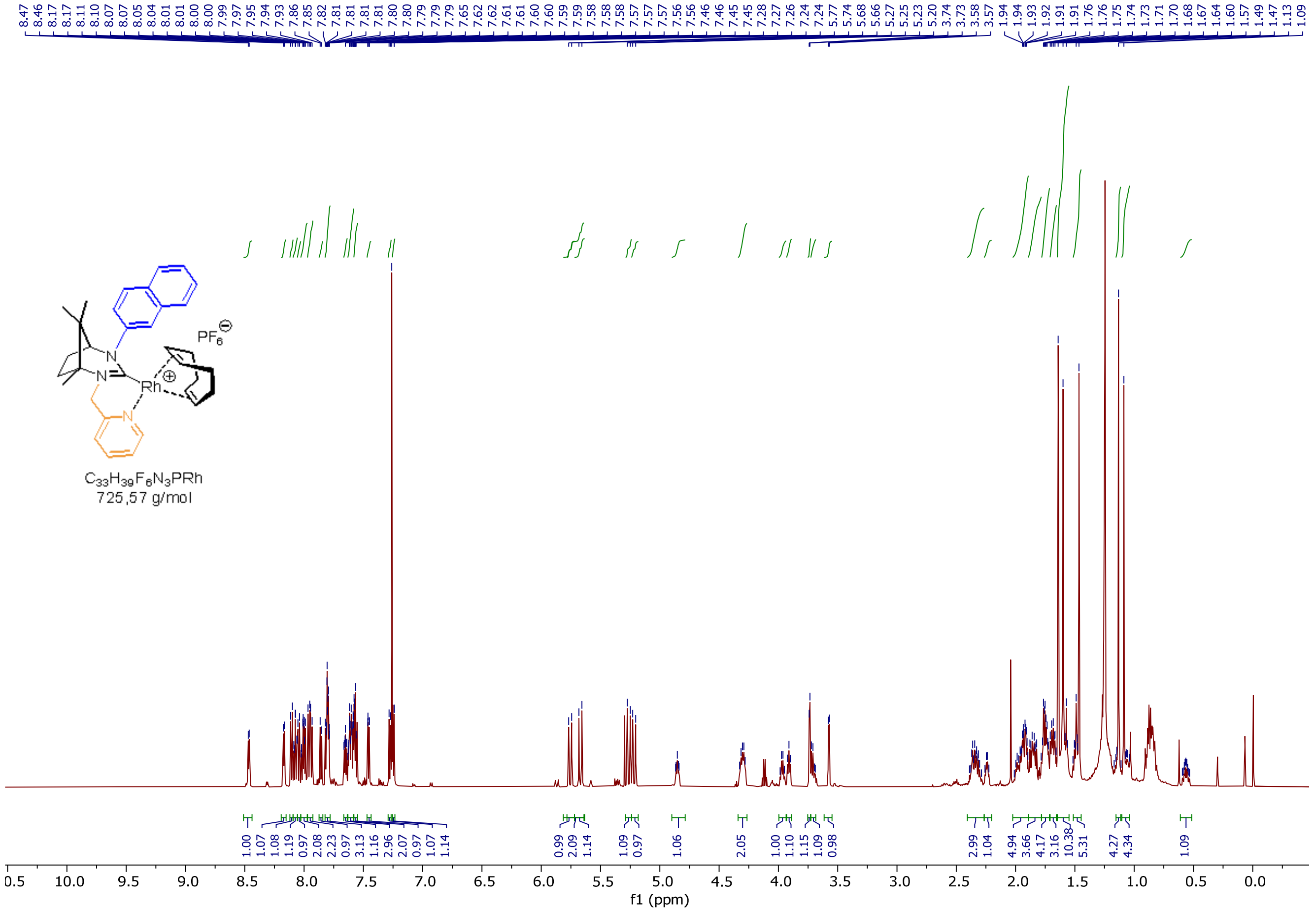

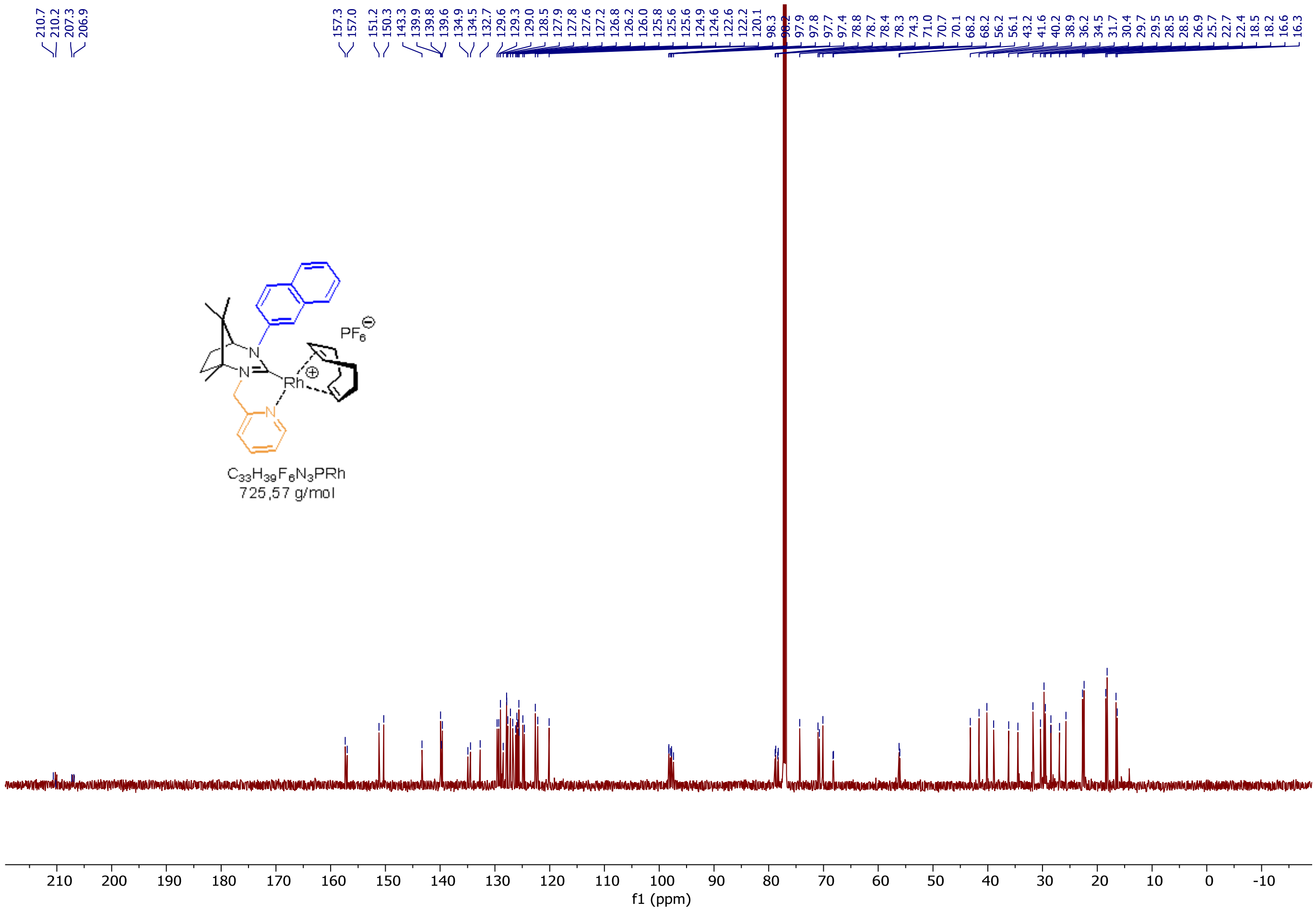

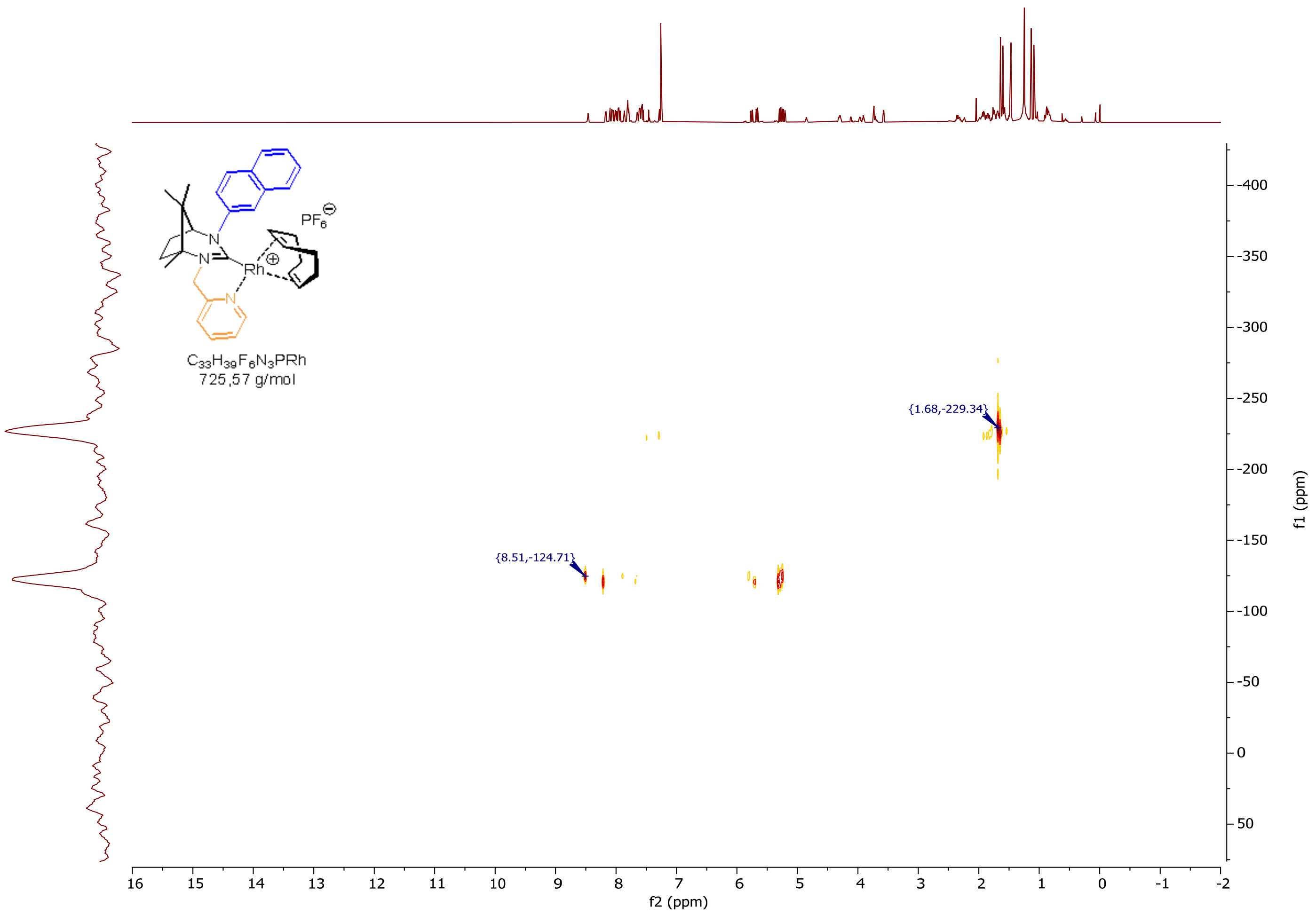
PF6
Rh
N
N
C33H39F6N3PRh
725,57 g/mol
{1.68,-229.34}
{8.51,-124.71}
f1 (ppm)
f2 (ppm)

^{1}H NMR (600 MHz, $CDCl_3$), $^{13}C\{^{1}H\}$ NMR (151 MHz, $CDCl_3$), ^{15}N HSQC NMR (61 MHz, $CDCl_3$) and ^{19}F NMR (337 MHz, $CDCl_3$) Analysis of **Complex Rh5bj**

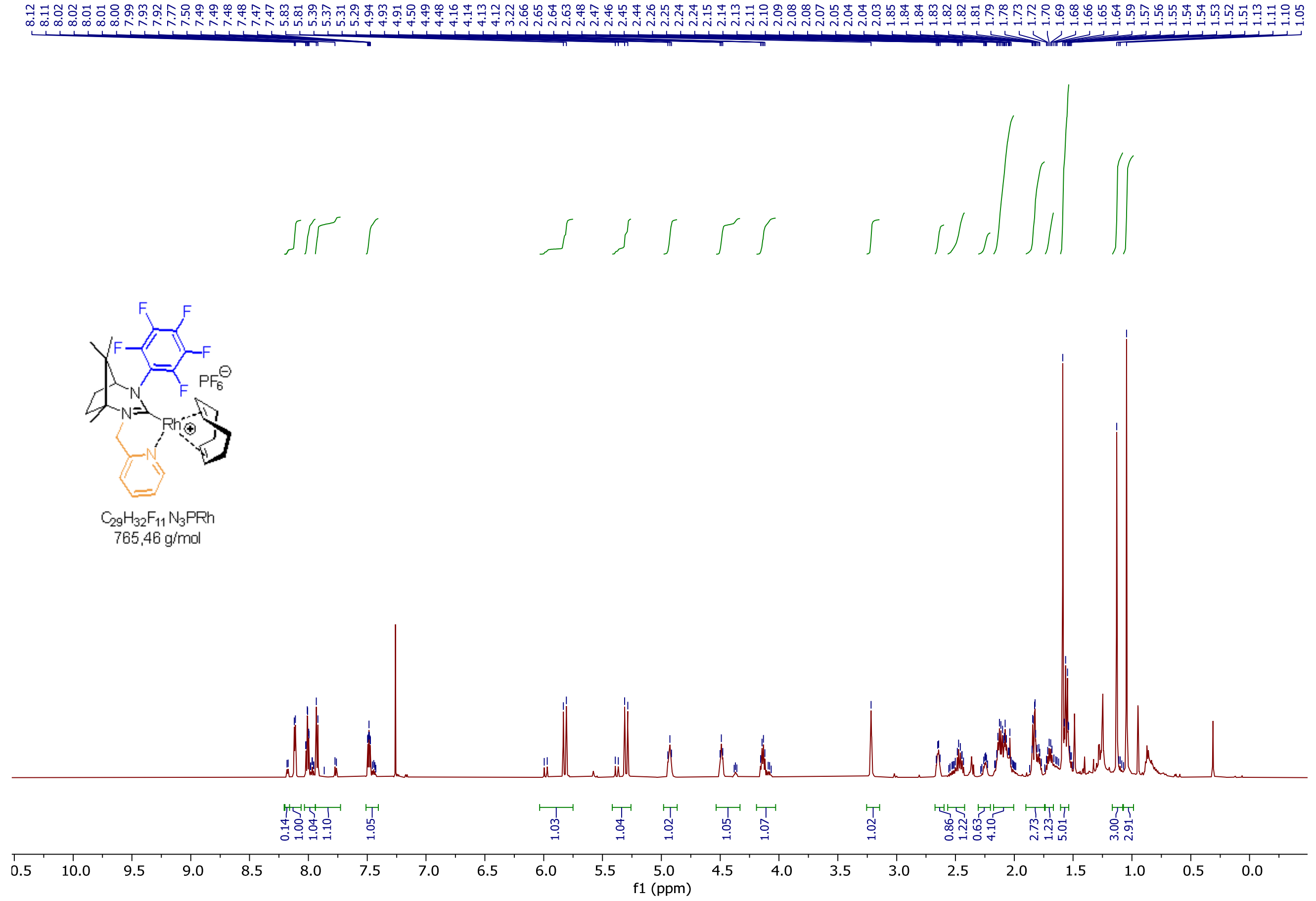

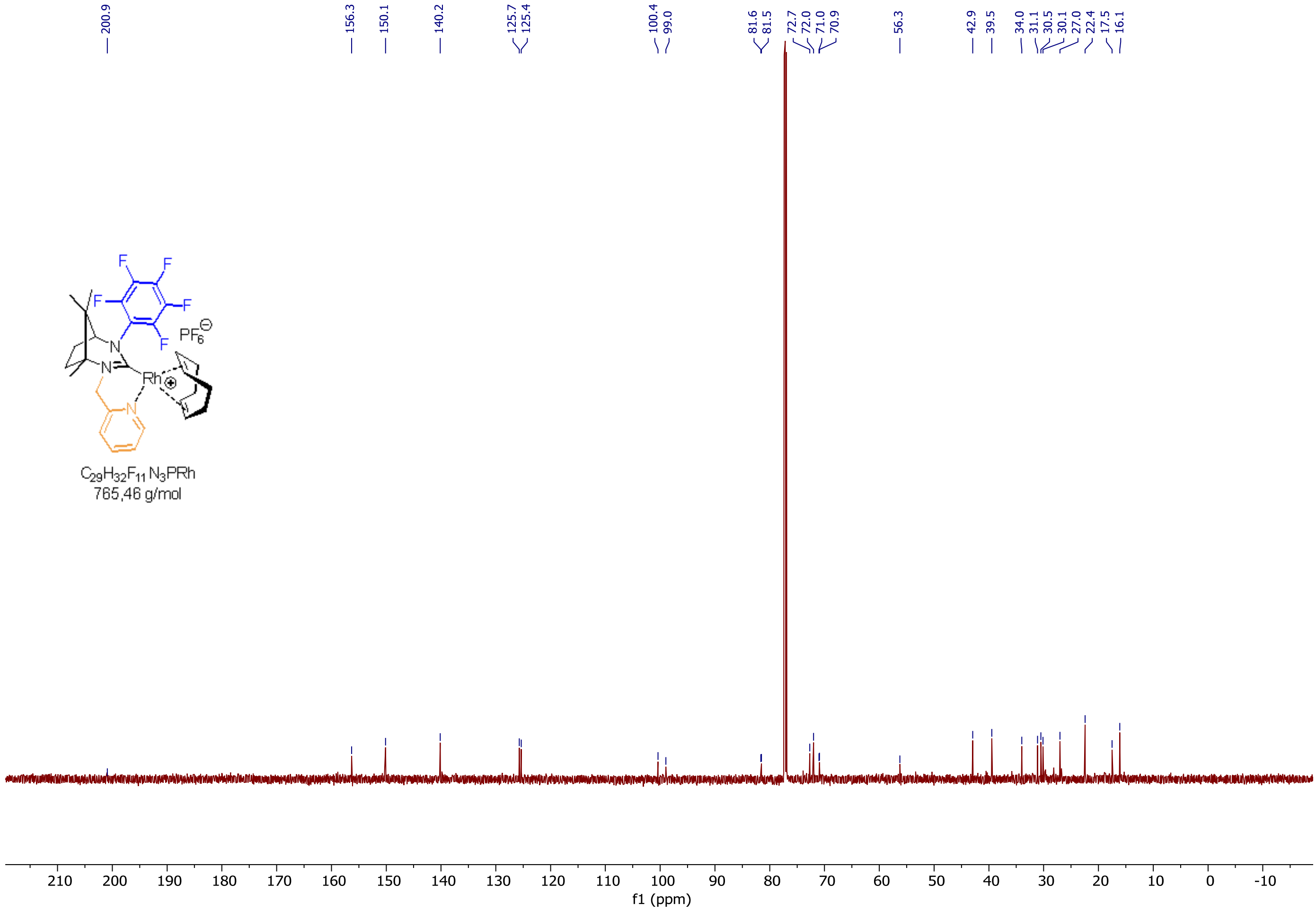

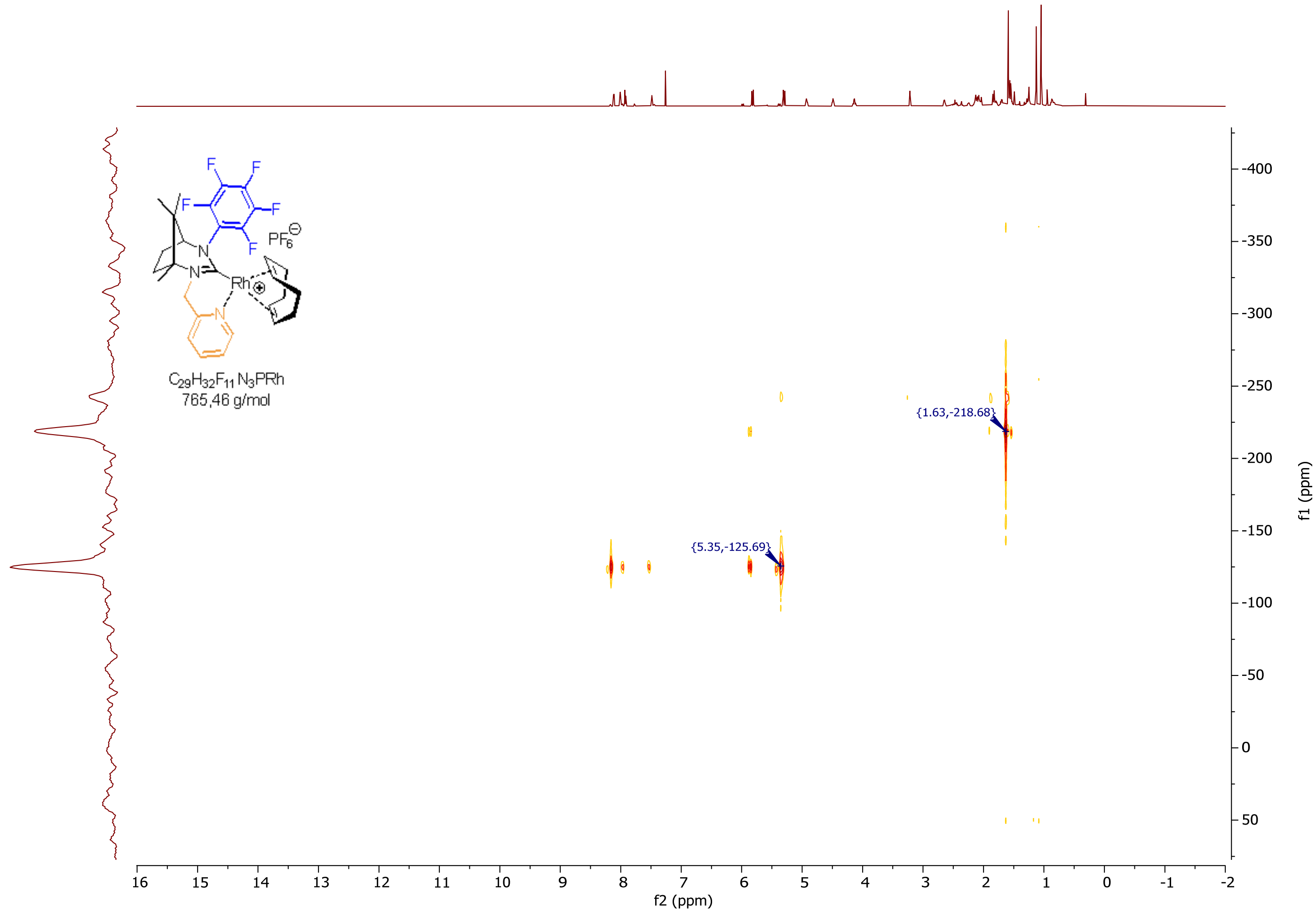

PF6
Rh
C29H32F11N3PRh
765,46 g/mol
{1.63,-218.68}
{5.35,-125.69}
f2 (ppm)
f1 (ppm)

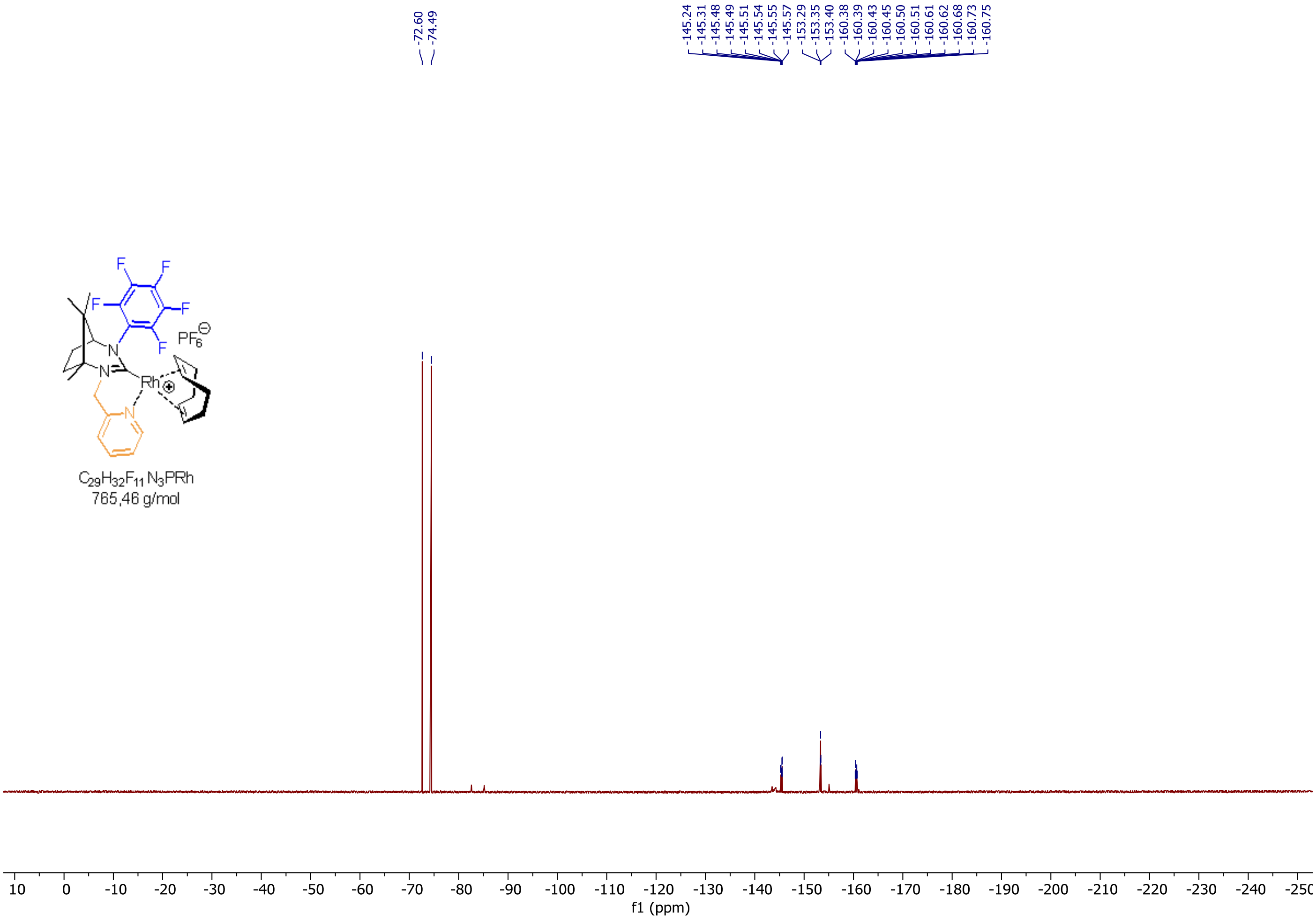

-72.60
-74.49
-145.24
-145.31
-145.48
-145.49
-145.51
-145.54
-145.55
-145.57
-153.29
-153.35
-153.40
-160.38
-160.39
-160.43
-160.45
-160.50
-160.51
-160.61
-160.62
-160.68
-160.73
-160.75
PF6
Rh
C29H32F11N3PRh
765,46 g/mol
f1 (ppm)

^{1}H NMR (600 MHz, CD_2Cl_2), ^{13}C{^{1}H} NMR (151 MHz, CD_2Cl_2), ^{15}N HSQC NMR (61 MHz, CD_2Cl_2) and ^{19}F NMR (337 MHz, CD_2Cl_2) Analysis of **Complex Rh5bk**

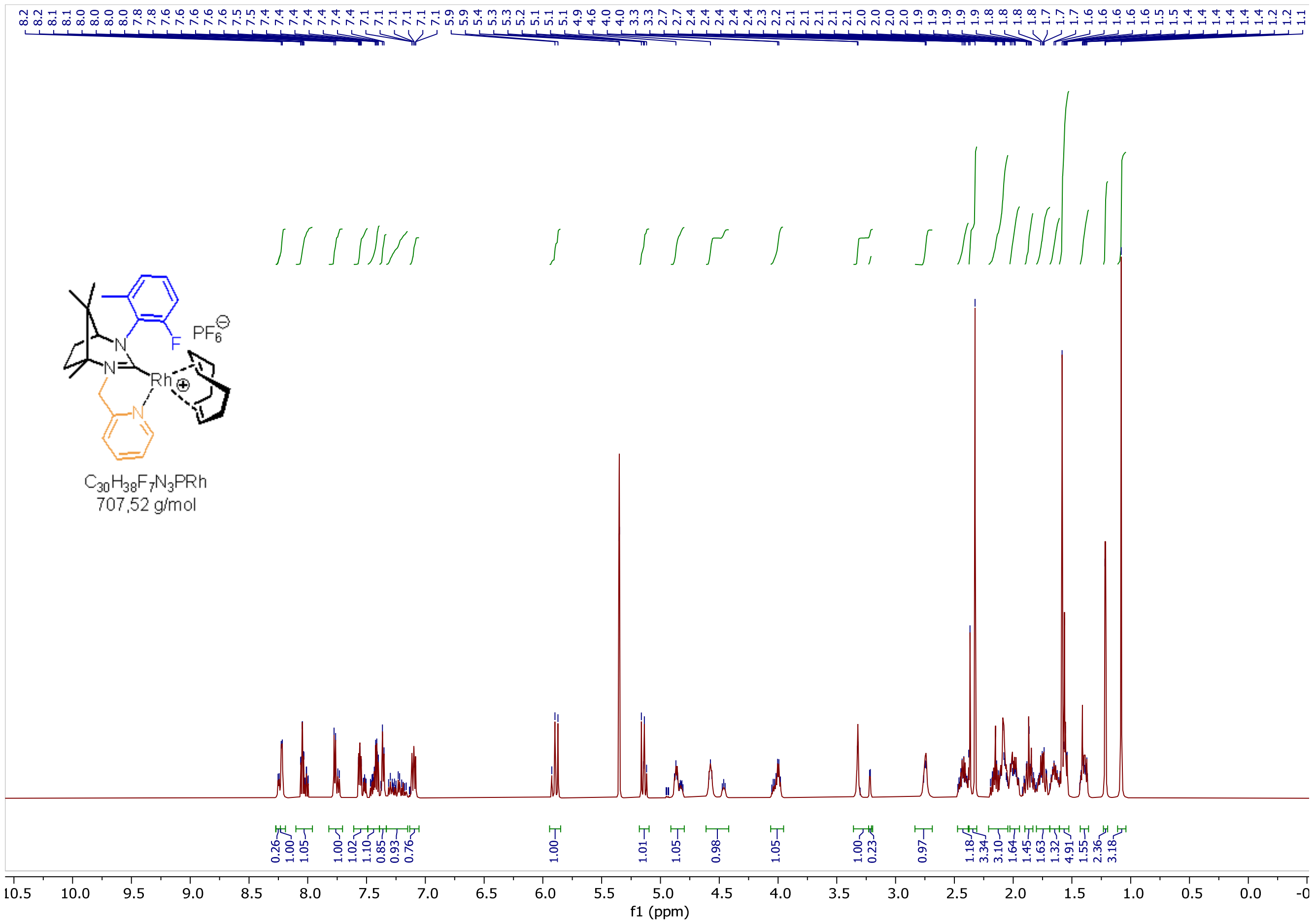

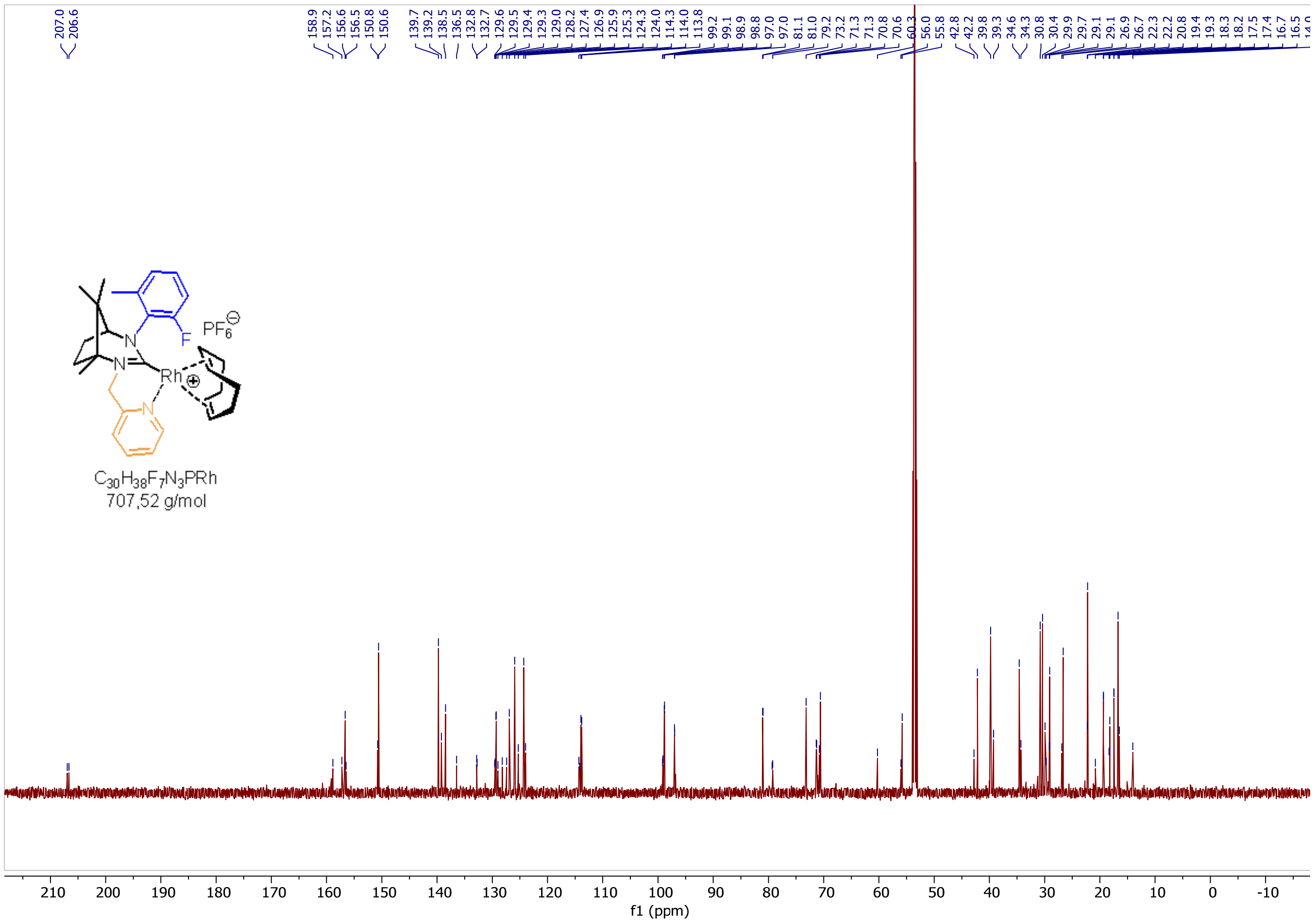

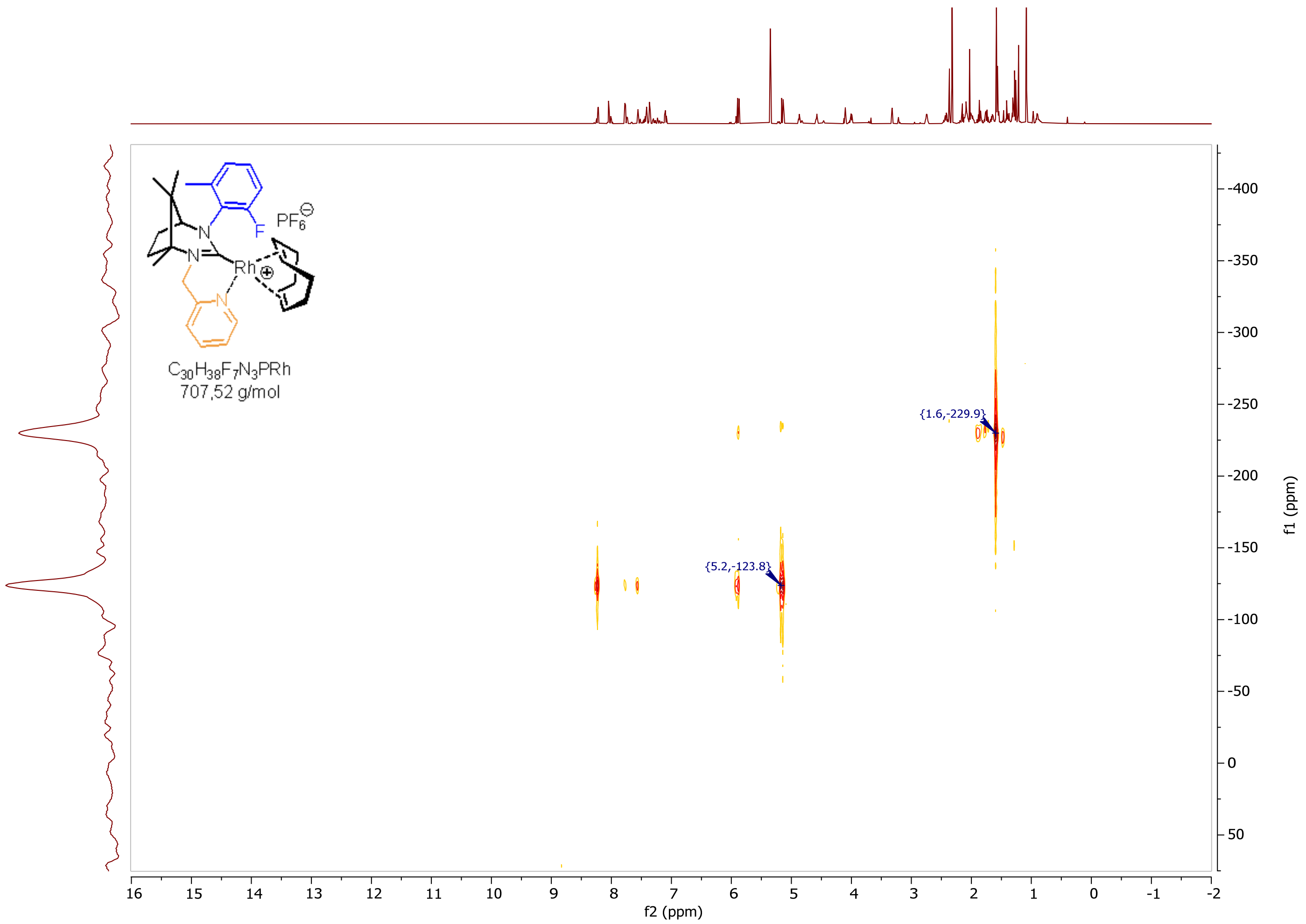
PF6
F
N
N
Rh
N
C30H38F7N3PRh
707,52 g/mol
{1.6,-229.9}
{5.2,-123.8}
f2 (ppm)
f1 (ppm)

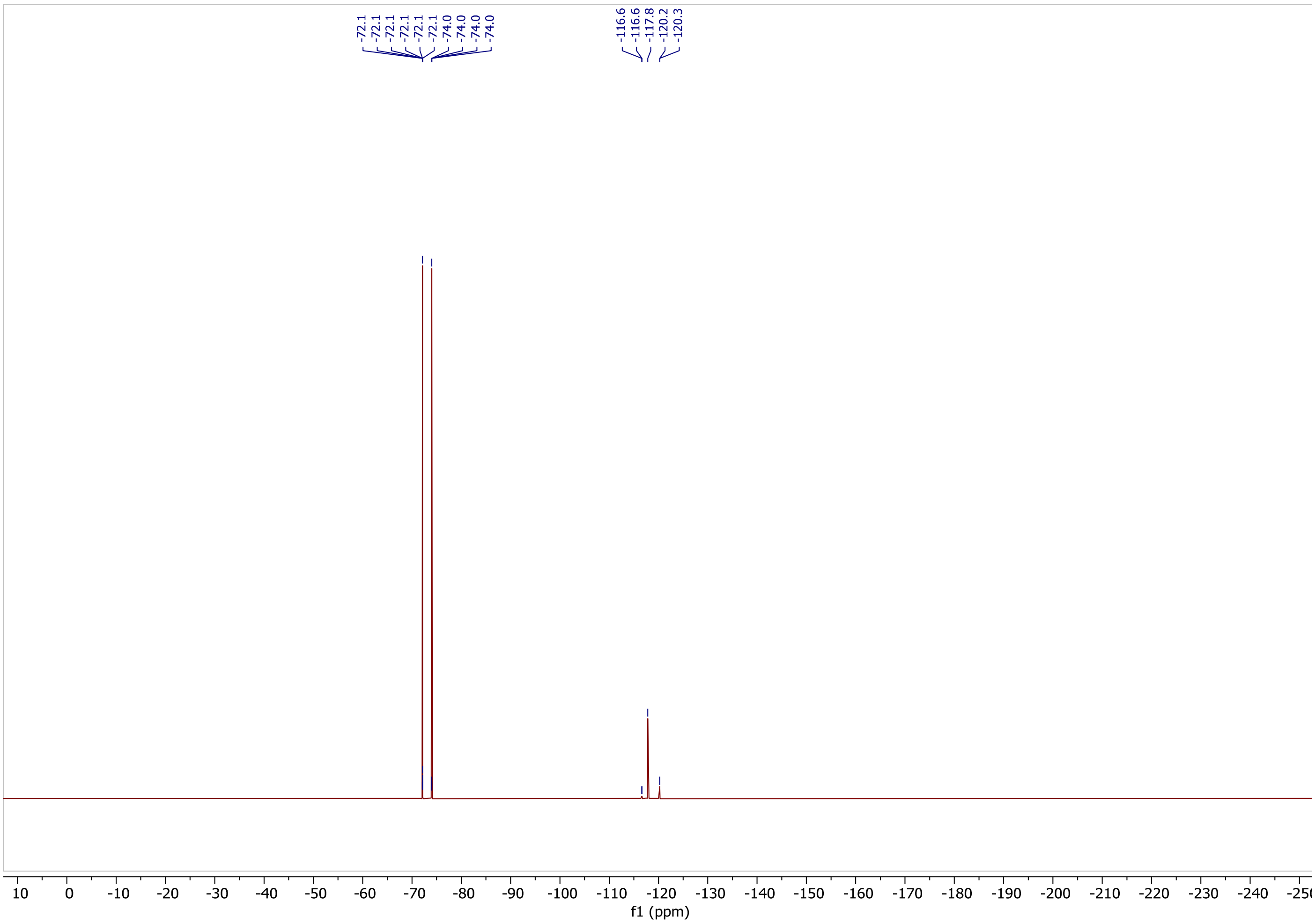
-72.1
-72.1
-72.1
-72.1
-72.1
-72.1
-74.0
-74.0
-74.0
-74.0
-116.6
-116.6
-117.8
-120.2
-120.3
f1 (ppm)

^1H NMR (600 MHz, CD_2Cl_2), $^{13}C\{^1H\}$ NMR (151 MHz, CD_2Cl_2) and ^{15}N HSQC NMR (61 MHz, CD_2Cl_2) Analysis of **Complex Rh5bl**

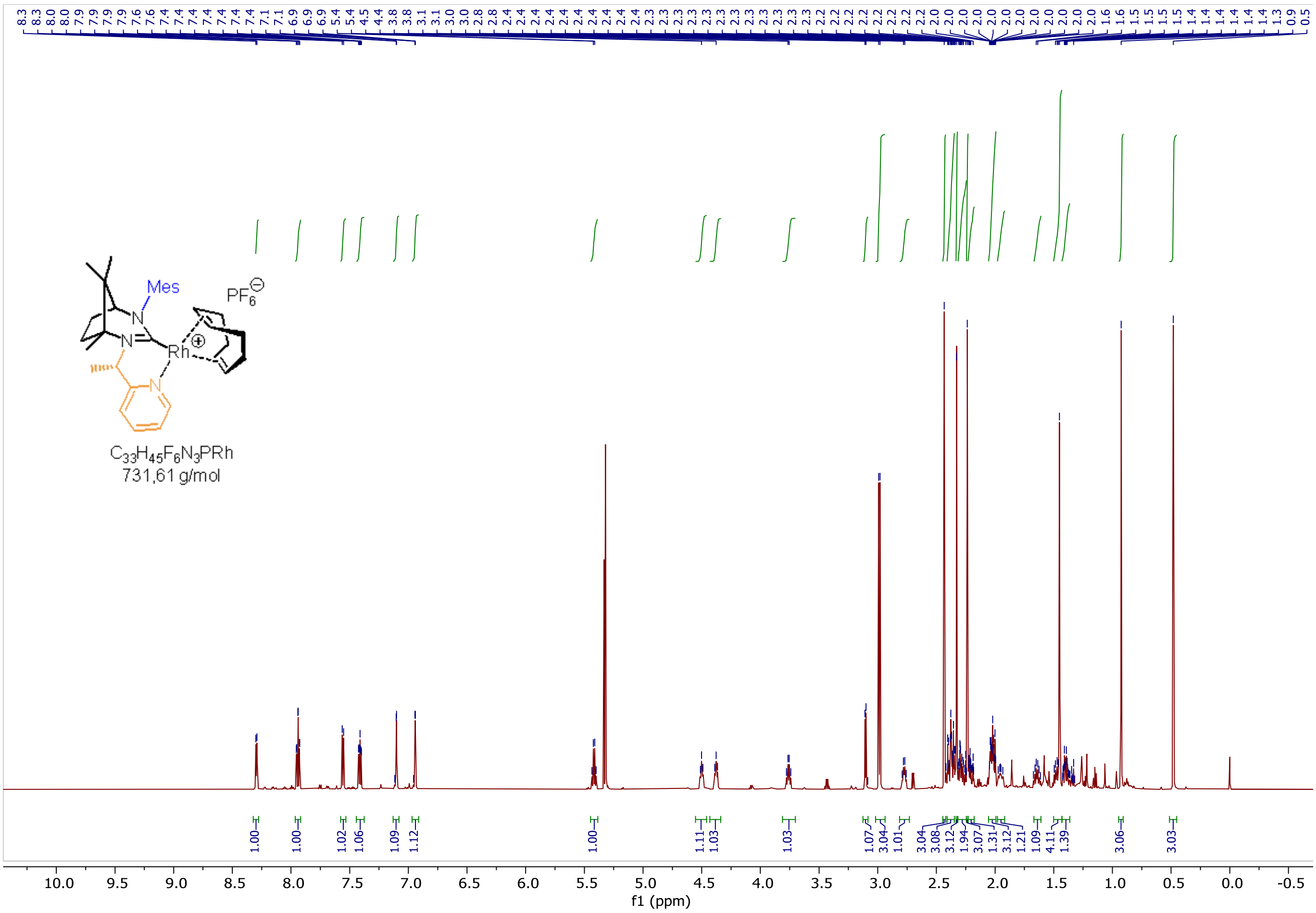

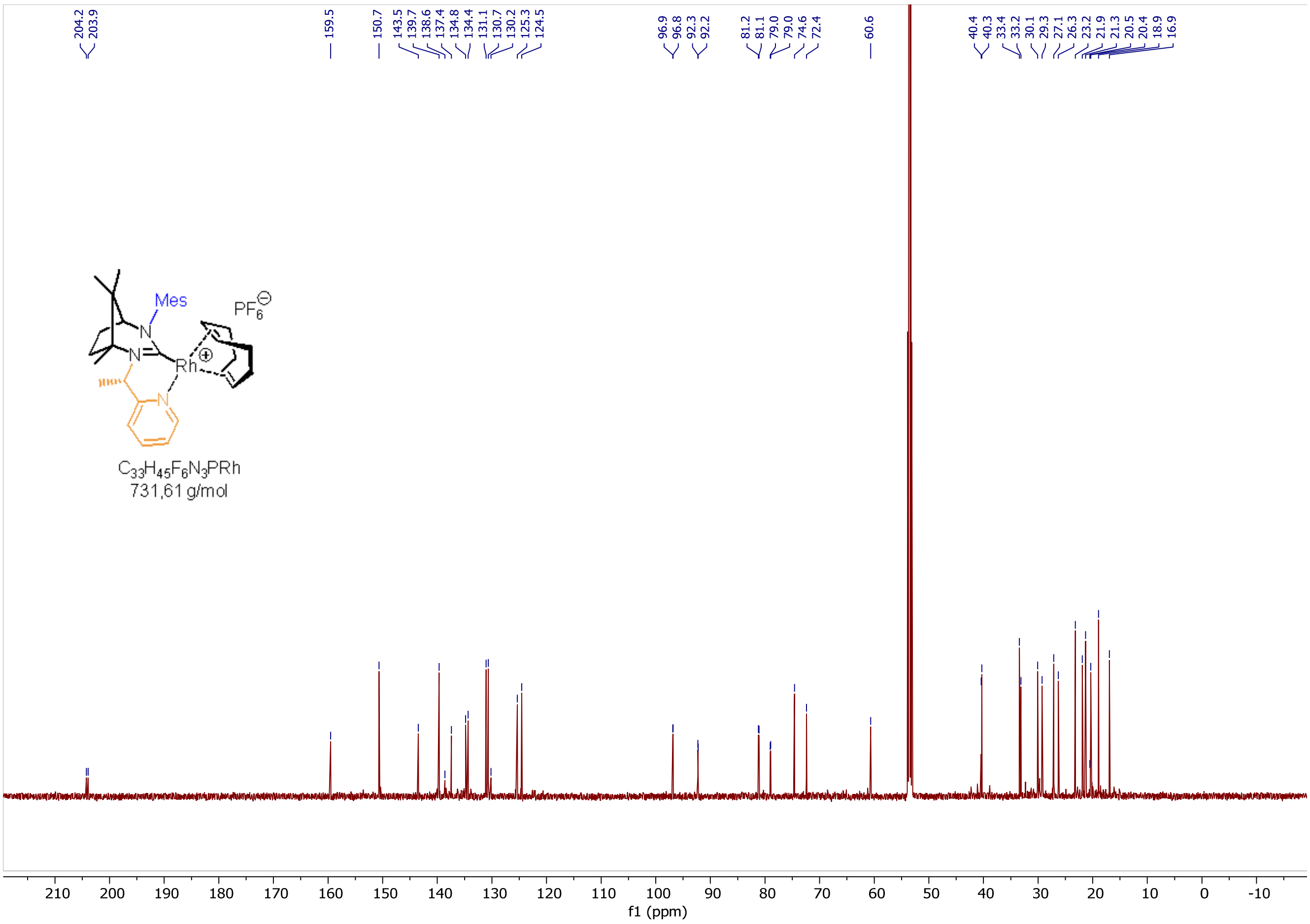

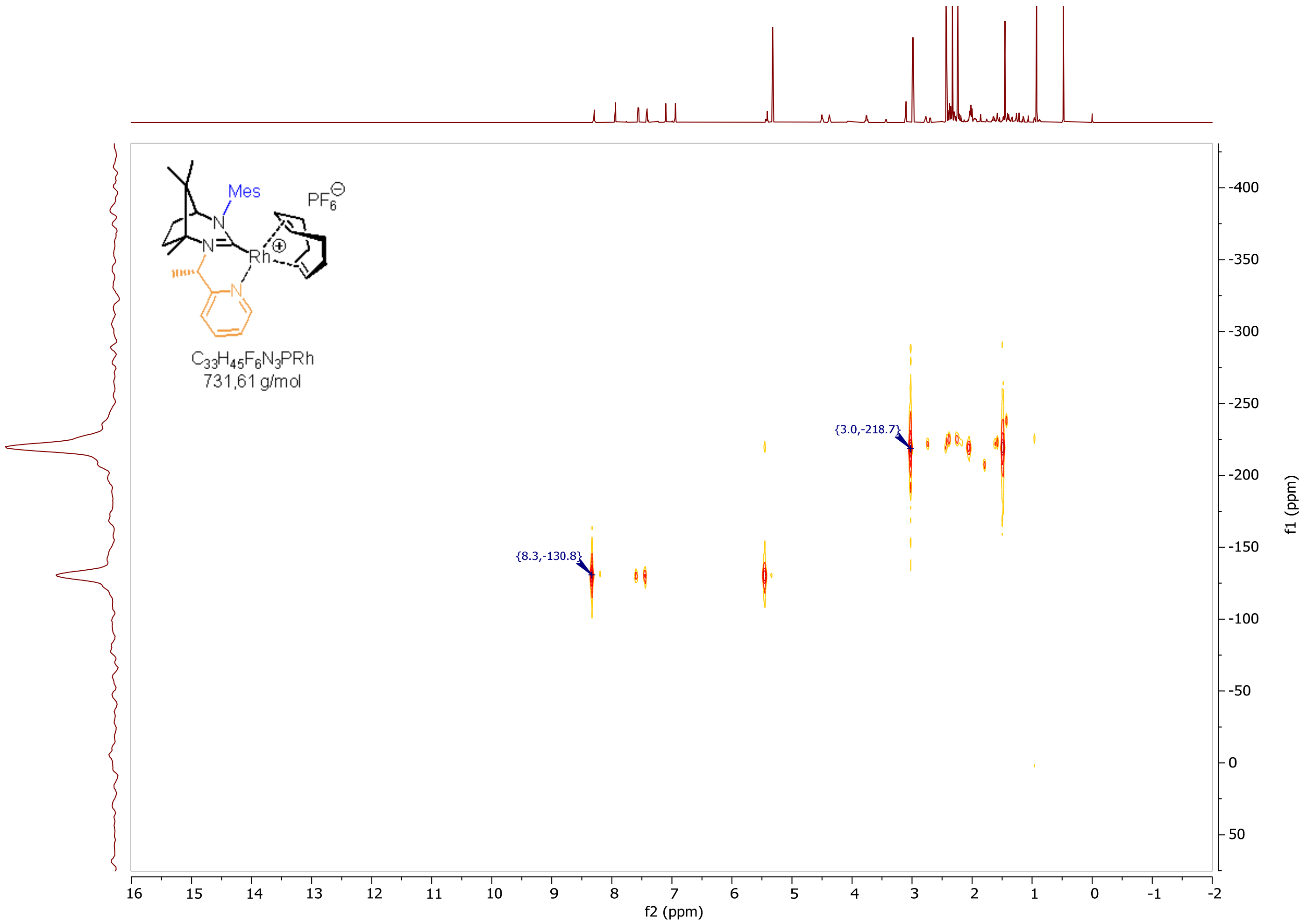

Mes
PF6
Rh
N
C33H45F6N3PRh
731,61 g/mol
{3.0,-218.7}
{8.3,-130.8}
f1 (ppm)
f2 (ppm)

^{1}H NMR (600 MHz, CD_2Cl_2), $^{13}C\{^{1}H\}$ NMR (151 MHz, CD_2Cl_2) and ^{15}N HSQC NMR (61 MHz, CD_2Cl_2) Analysis of **Complex Rh5bm**

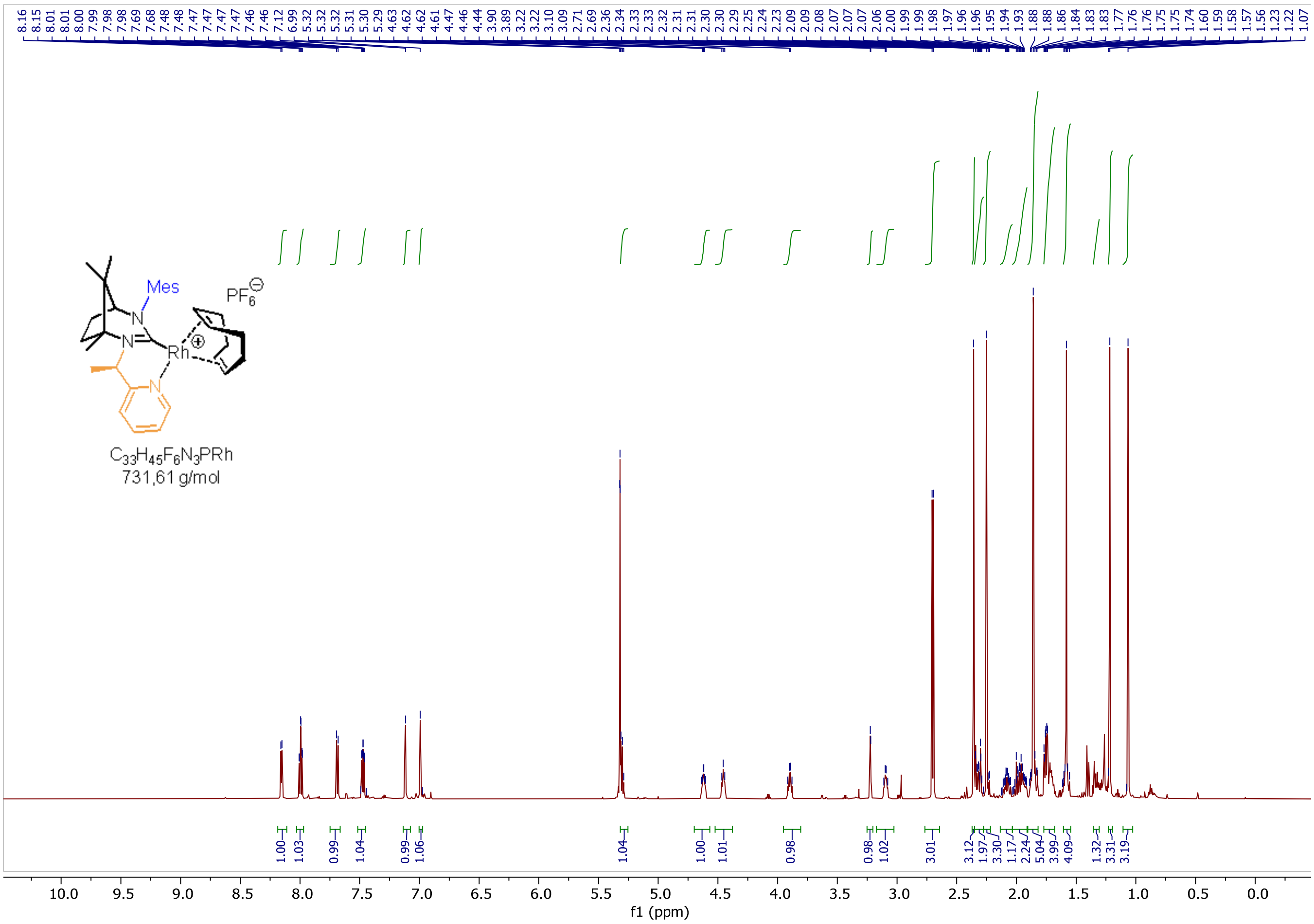

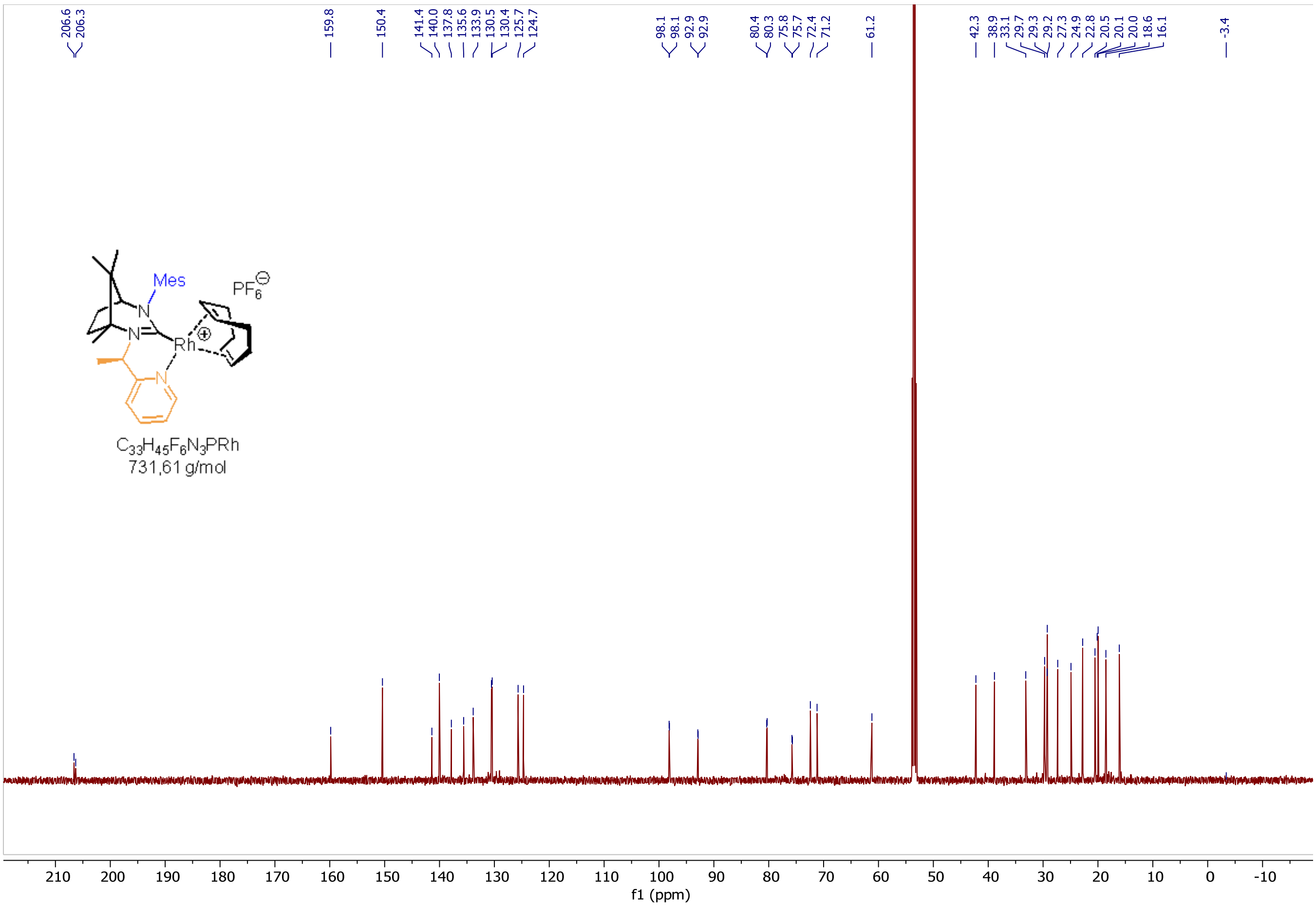

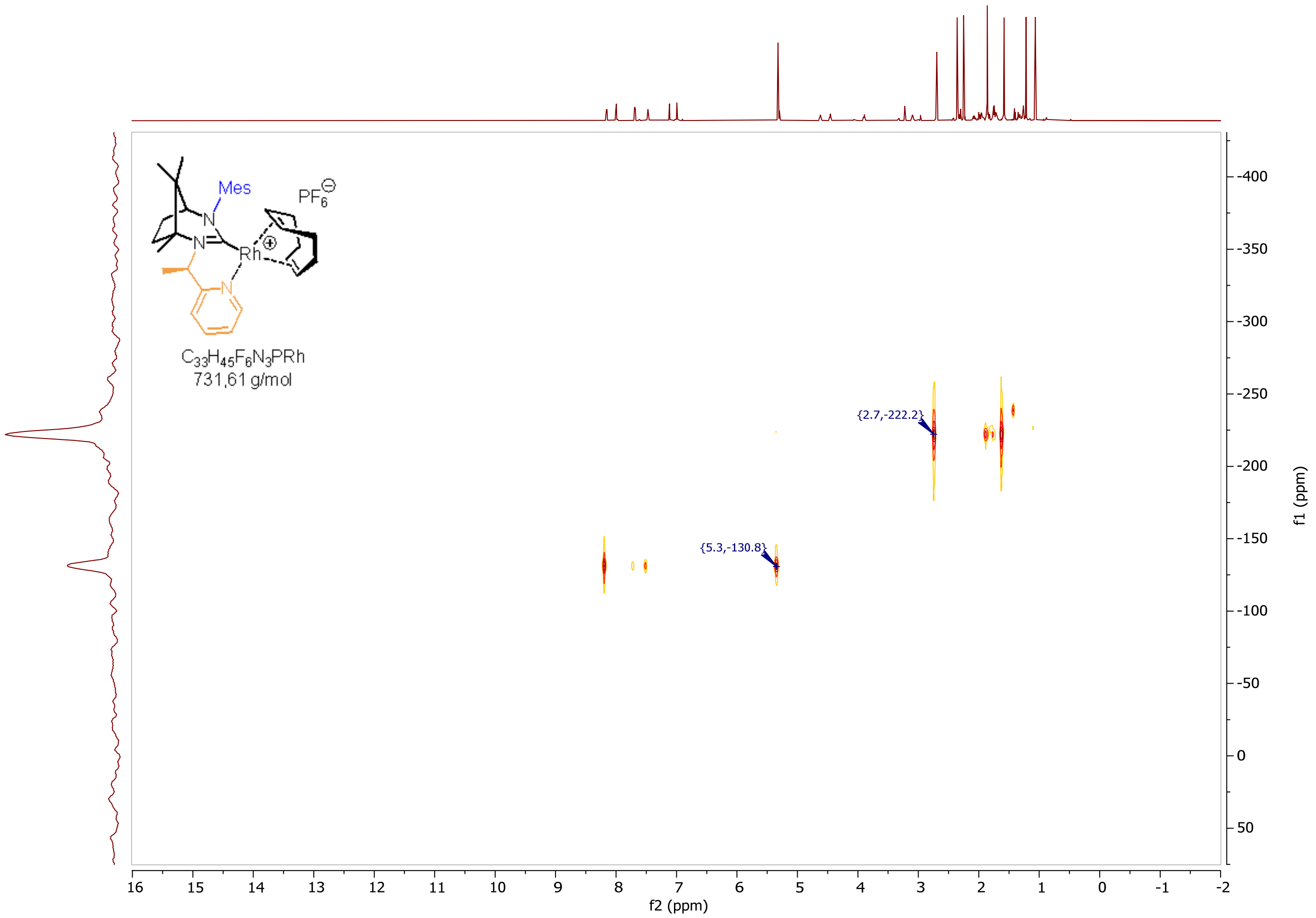

Mes
PF6
Rh
N
C33H45F6N3PRh
731,61 g/mol
{2.7,-222.2}
{5.3,-130.8}
f2 (ppm)
f1 (ppm)

^{1}H NMR (600 MHz, $CDCl_3$), $^{13}C\{^{1}H\}$ NMR (151 MHz, $CDCl_3$) and ^{15}N HSQC NMR (61 MHz, $CDCl_3$) Analysis of **Complex Rh5bn**

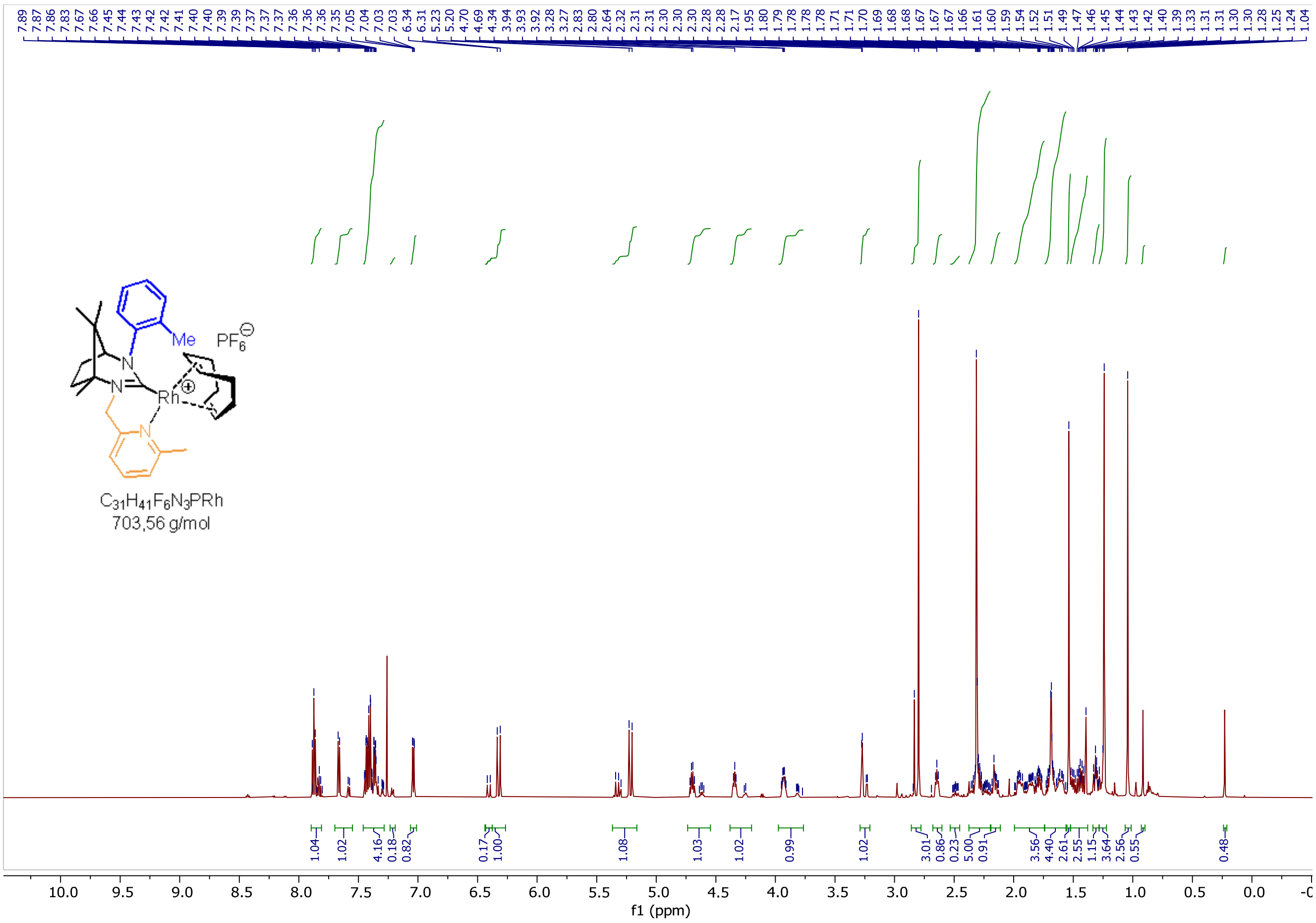

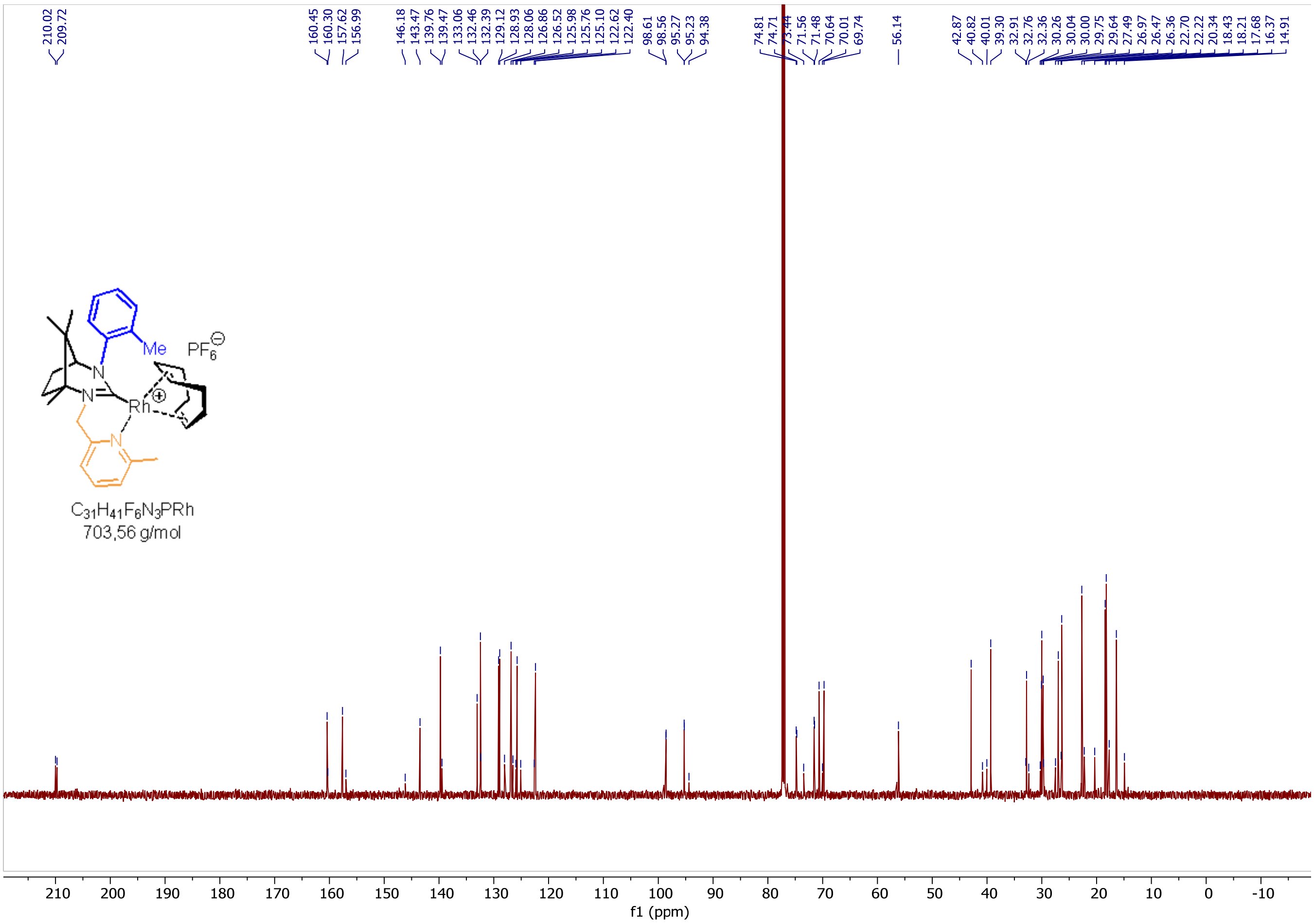

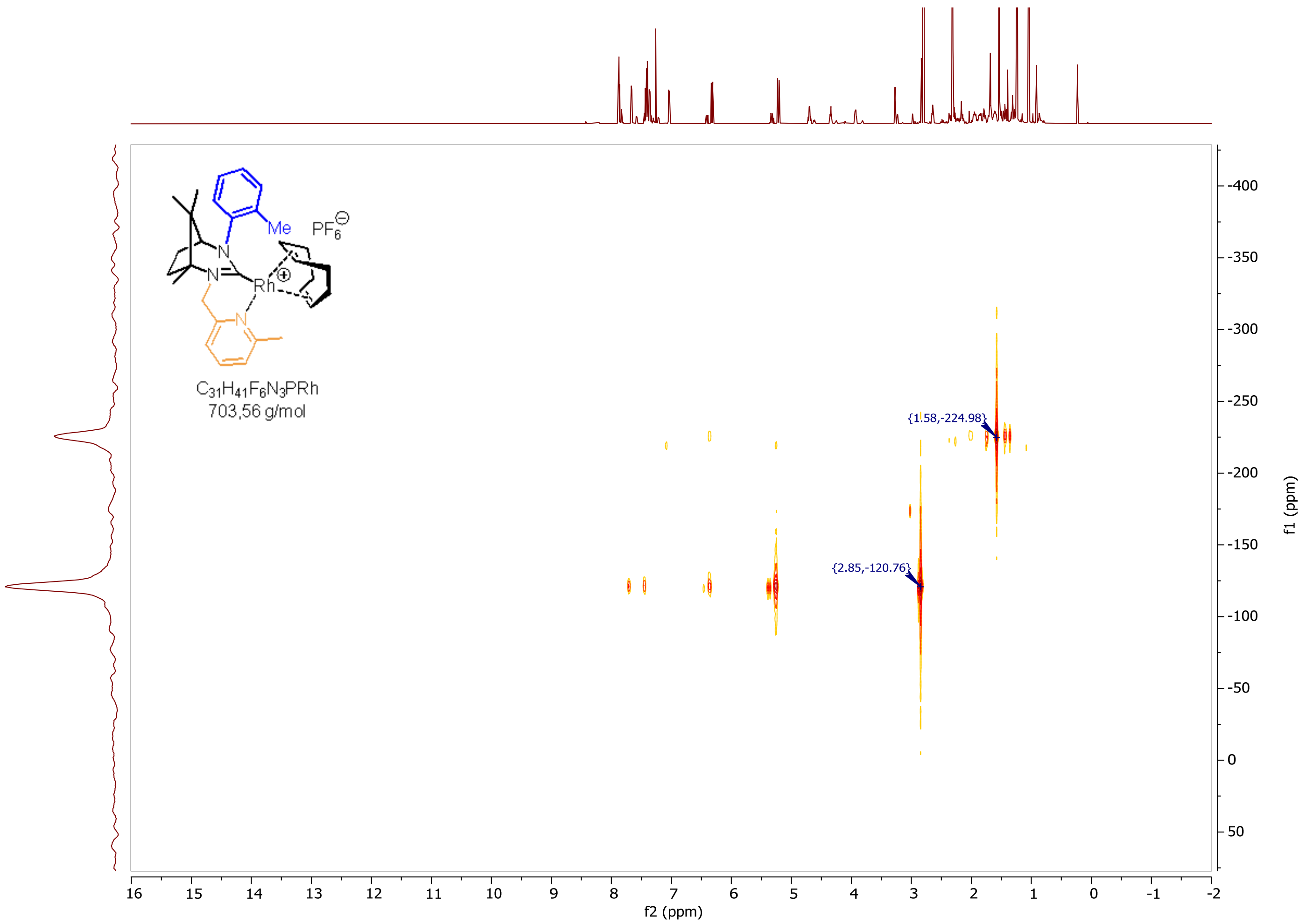
Me
PF6
N
N
Rh
N
C31H41F6N3PRh
703,56 g/mol
{1.58,-224.98}
{2.85,-120.76}
f2 (ppm)
f1 (ppm)

^{1}H NMR (600 MHz, $CDCl_3$), ^{13}C{^{1}H} NMR (151 MHz, $CDCl_3$) and ^{15}N HSQC NMR (61 MHz, $CDCl_3$) Analysis of **Complex Rh5bo**

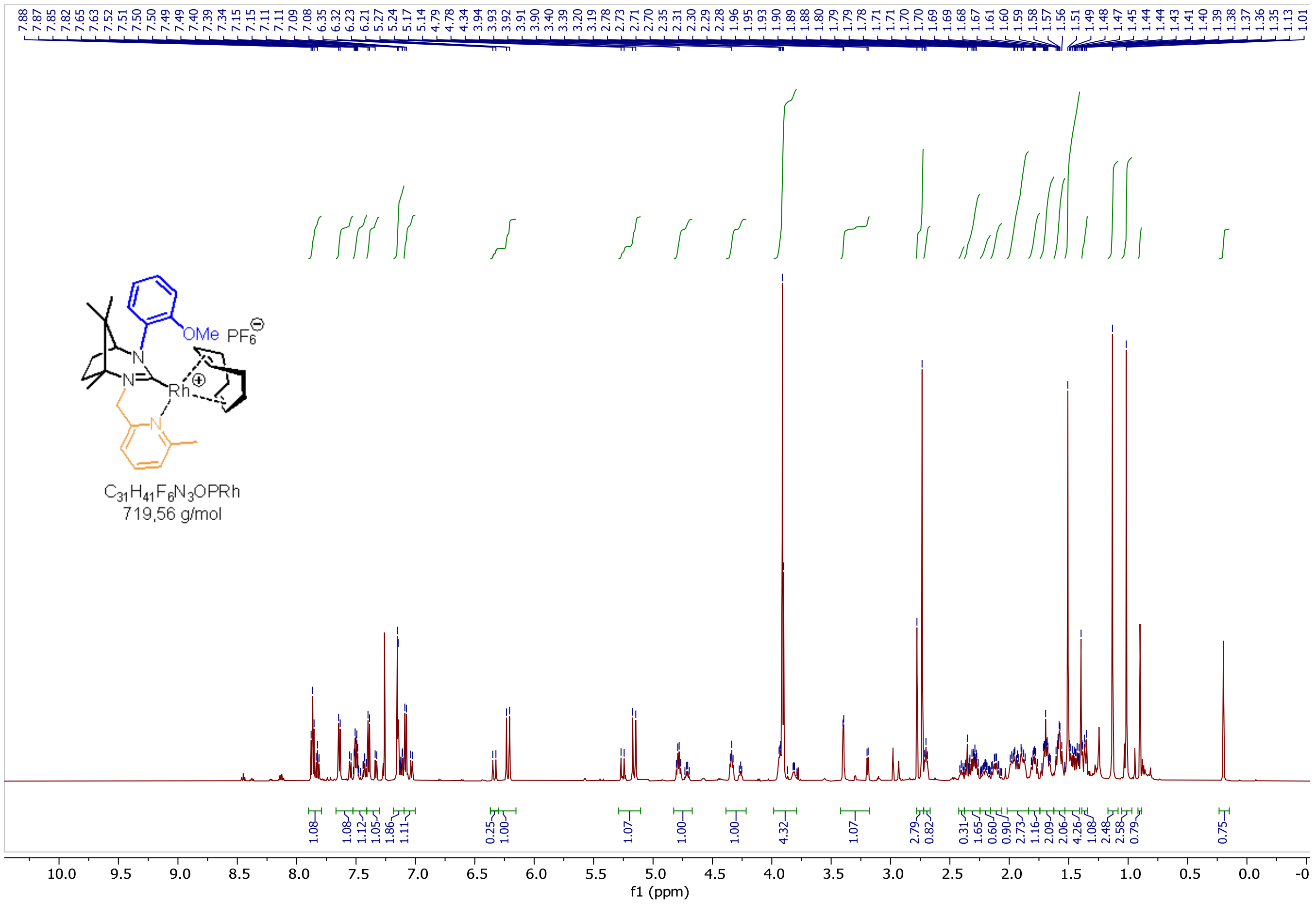

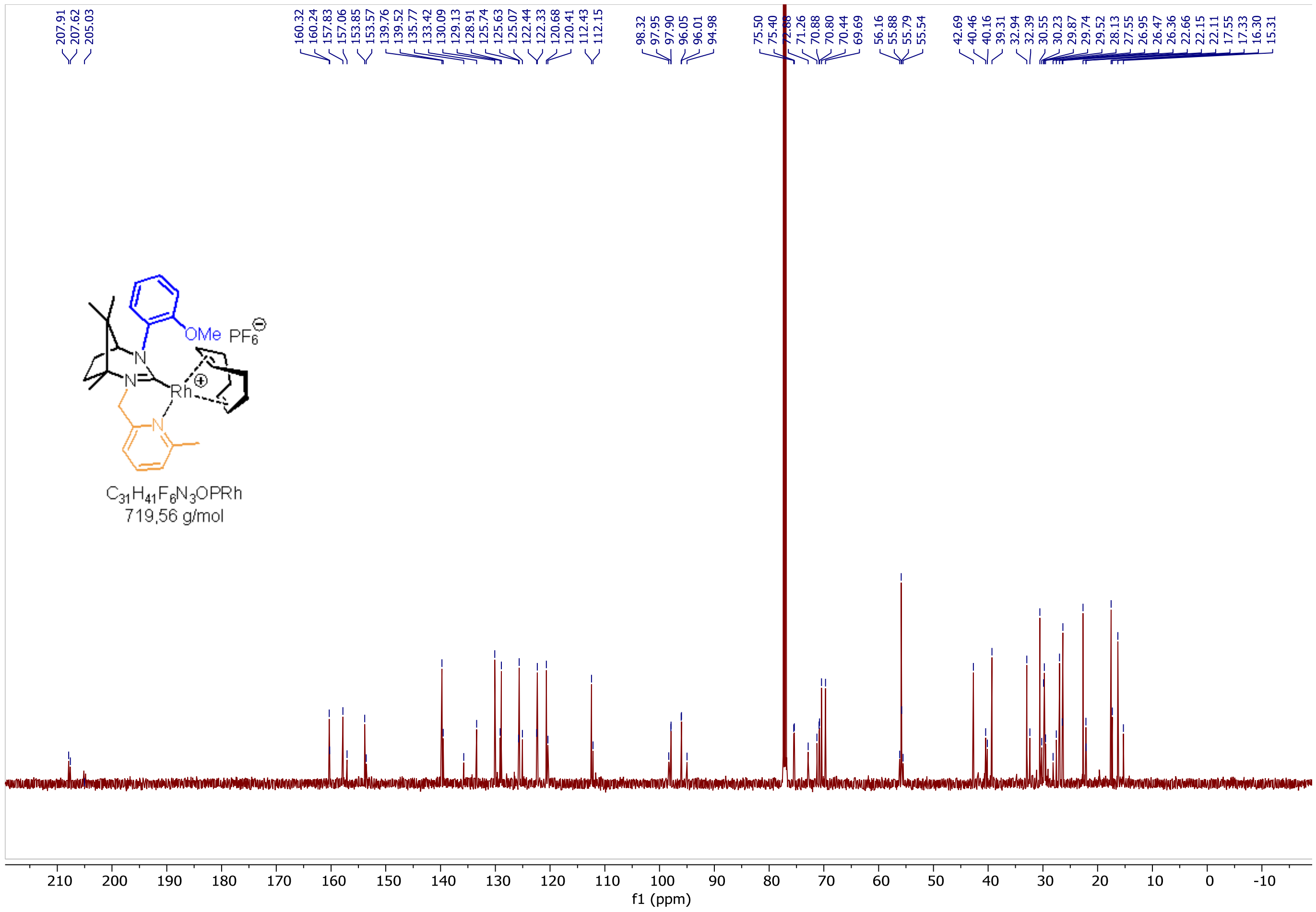

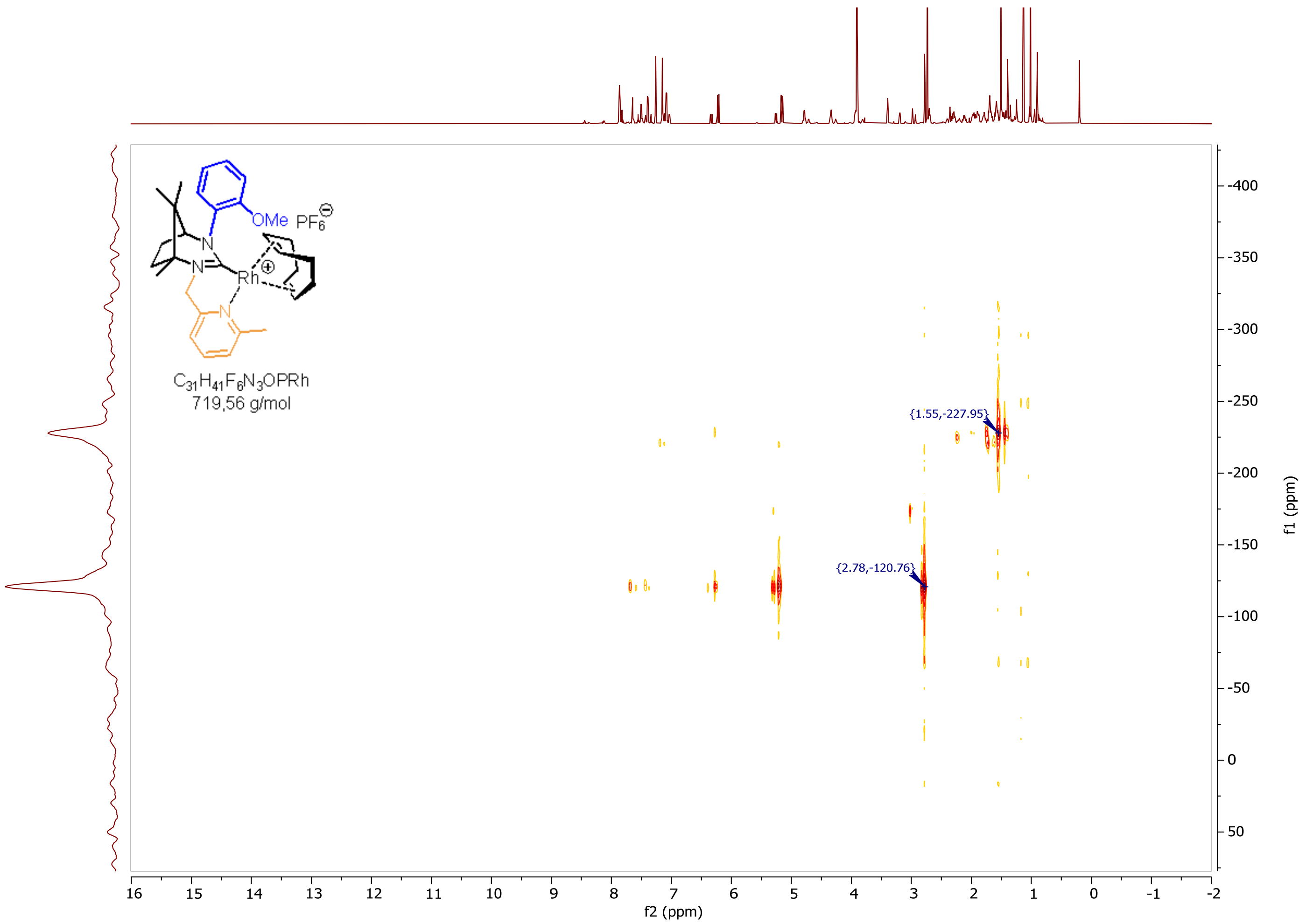

OMe
PF6
N
N
Rh
N
C31H41F6N3OPRh
719,56 g/mol
{1.55,-227.95}
{2.78,-120.76}
f2 (ppm)
f1 (ppm)

^{1}H NMR (600 MHz, $CDCl_3$), $^{13}C\{^{1}H\}$ NMR (151 MHz, $CDCl_3$) and ^{15}N HSQC NMR (61 MHz, $CDCl_3$) Analysis of **Complex Rh5bp**

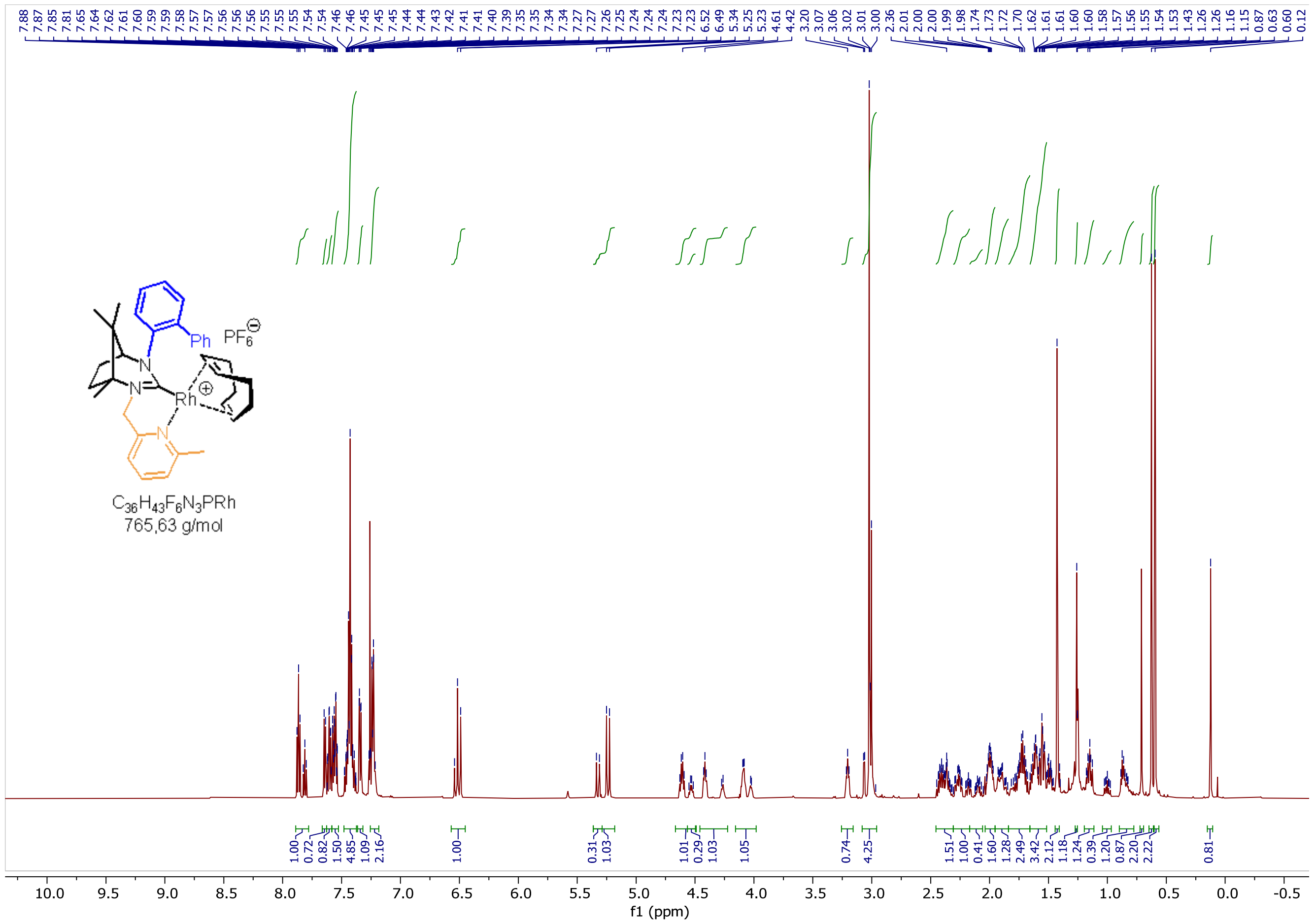

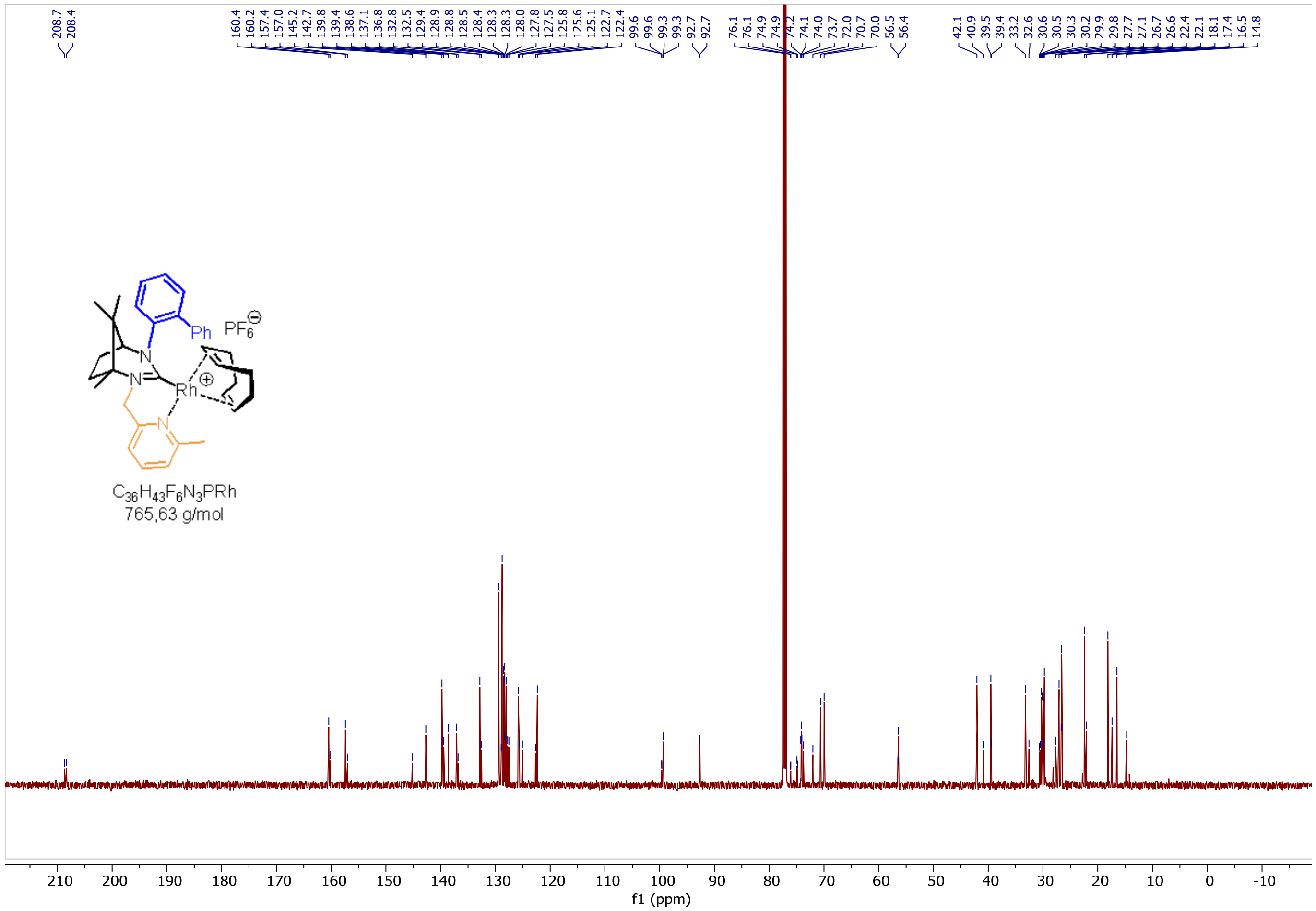

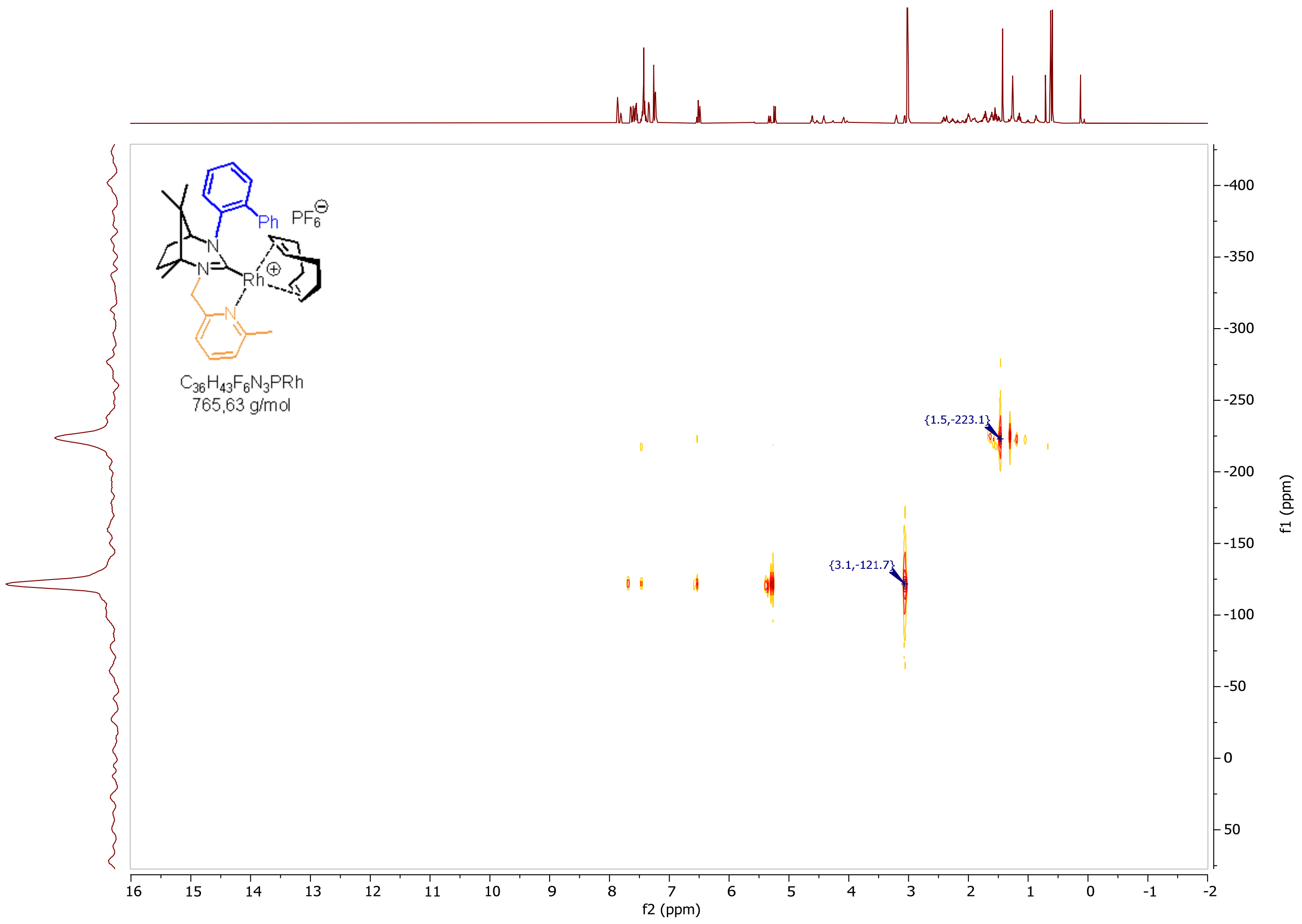

Ph
$PF_6^{\ominus}$
N
N
Rh
N
$C_{36}H_{43}F_6N_3PRh$
765,63 g/mol
{1.5,-223.1}
{3.1,-121.7}
f2 (ppm)
f1 (ppm)

^1H NMR (600 MHz, $CDCl_3$), $^{13}C\{^1H\}$ NMR (151 MHz, $CDCl_3$) and ^{15}N HSQC NMR (61 MHz, $CDCl_3$) Analysis of **Complex Rh5br**

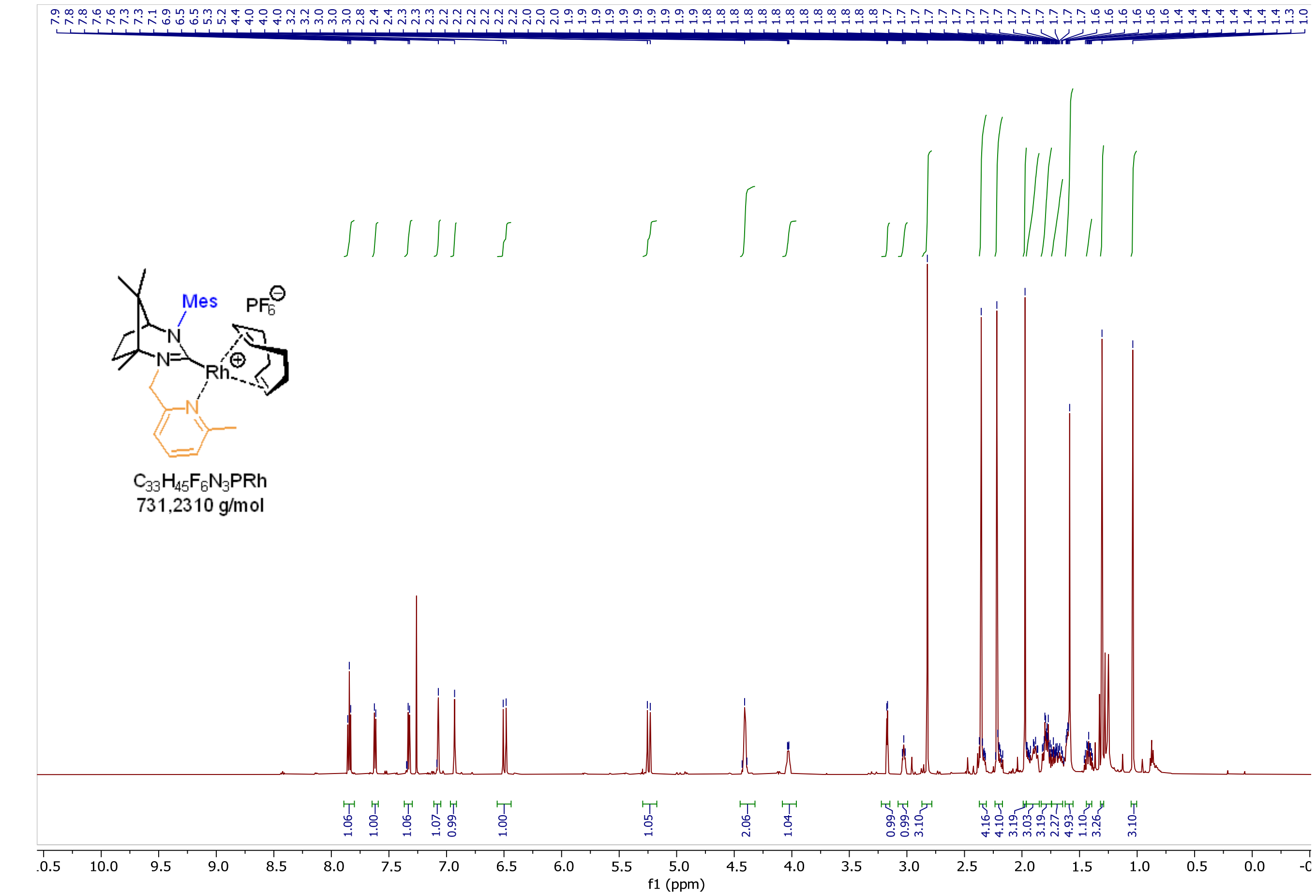

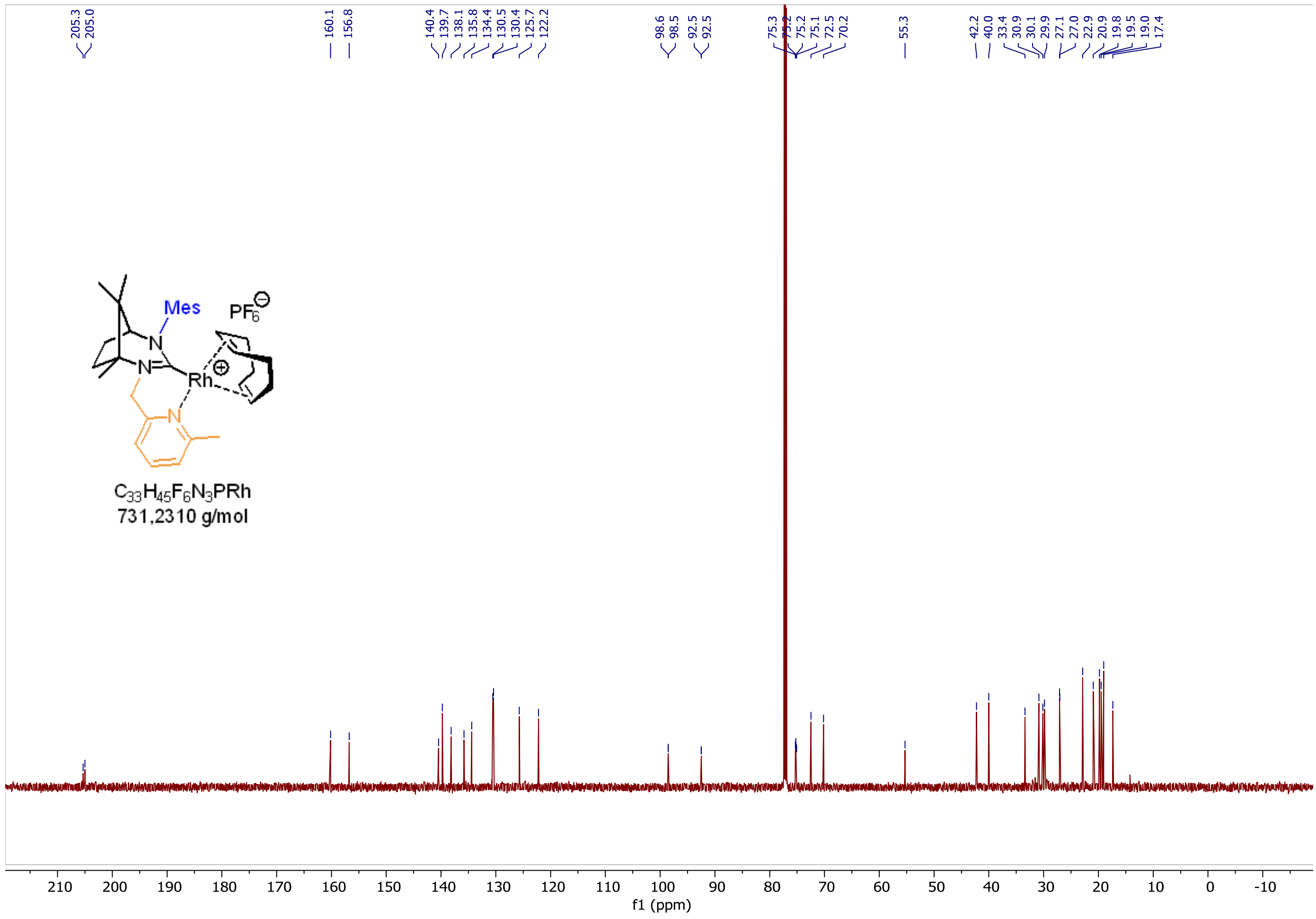

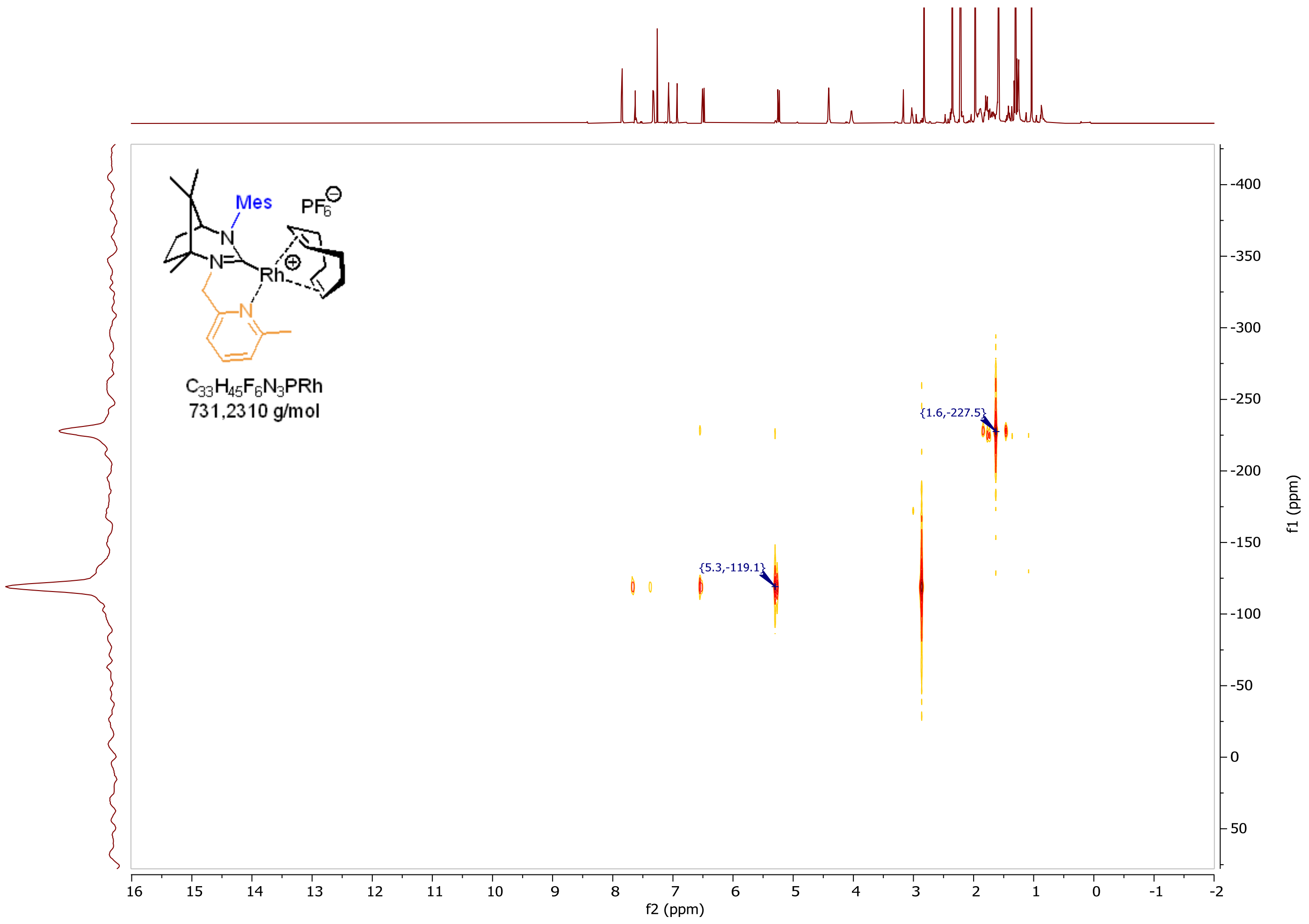
Mes
PF6
N
N
Rh
N
C33H45F6N3PRh
731,2310 g/mol
{1.6,-227.5}
{5.3,-119.1}
f2 (ppm)
f1 (ppm)

^{1}H NMR (600 MHz, $CDCl_3$), ^{13}C{^{1}H} NMR (151 MHz, $CDCl_3$) and ^{15}N HSQC NMR (61 MHz, $CDCl_3$) Analysis of **Complex Rh5bs**

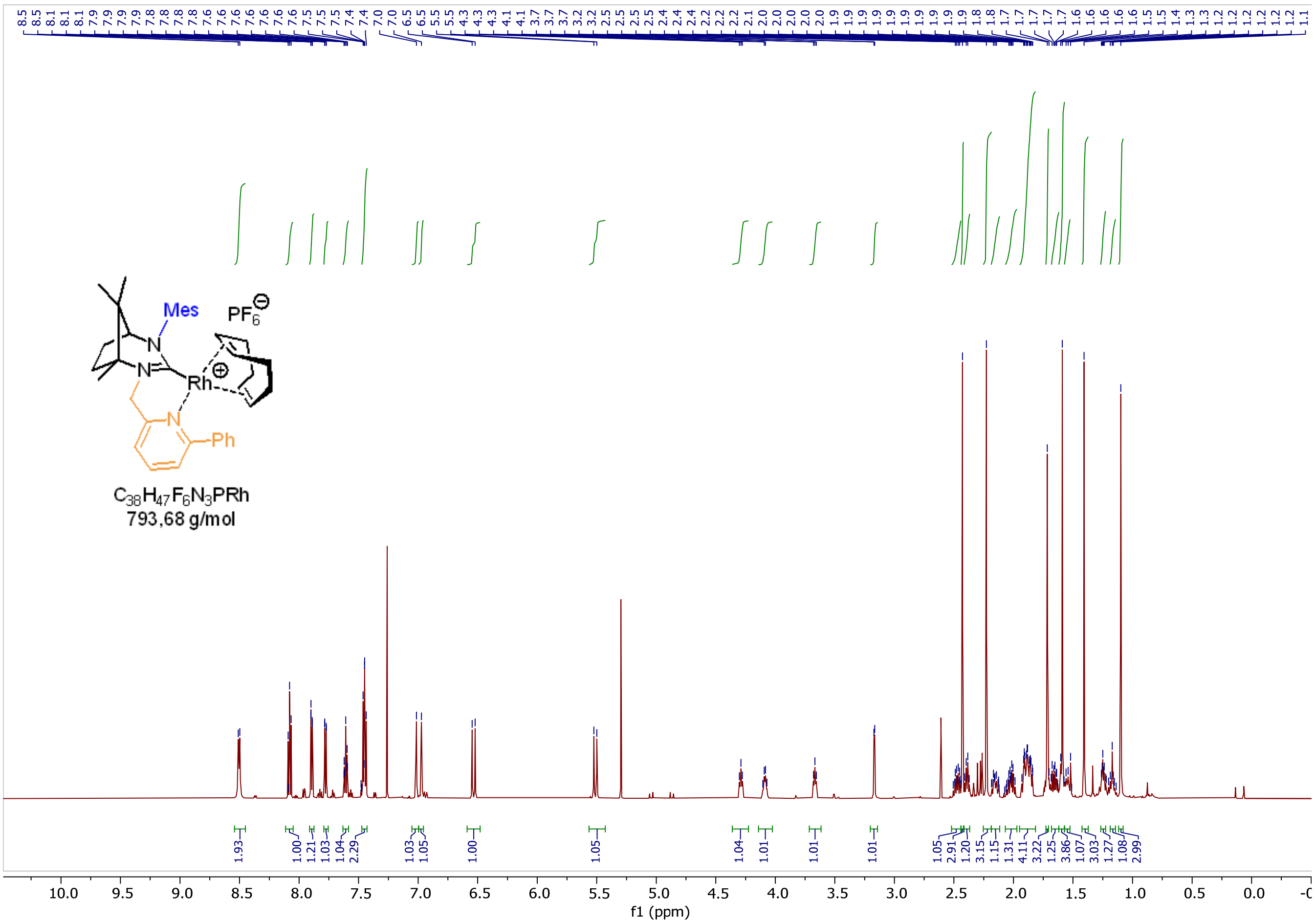

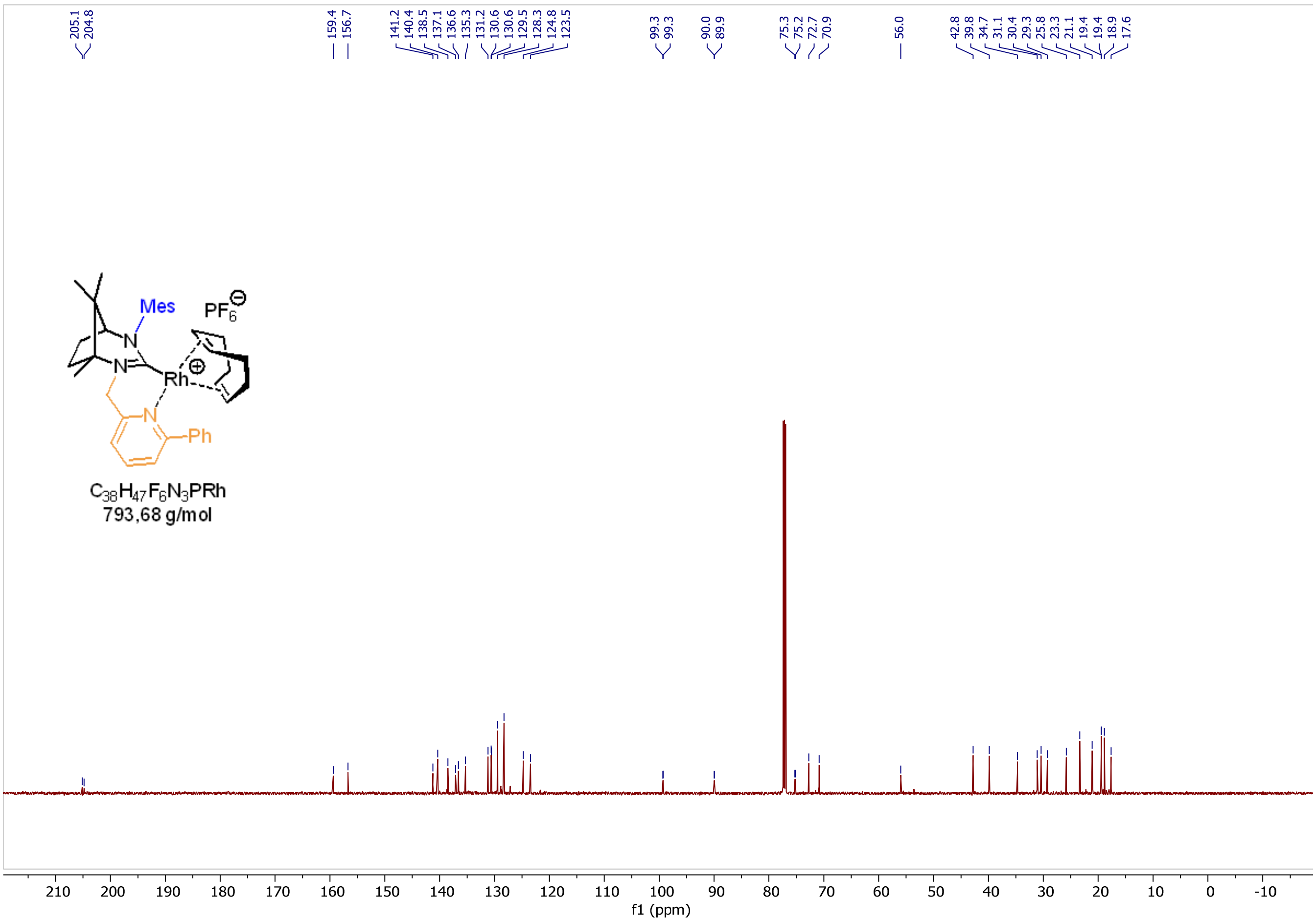

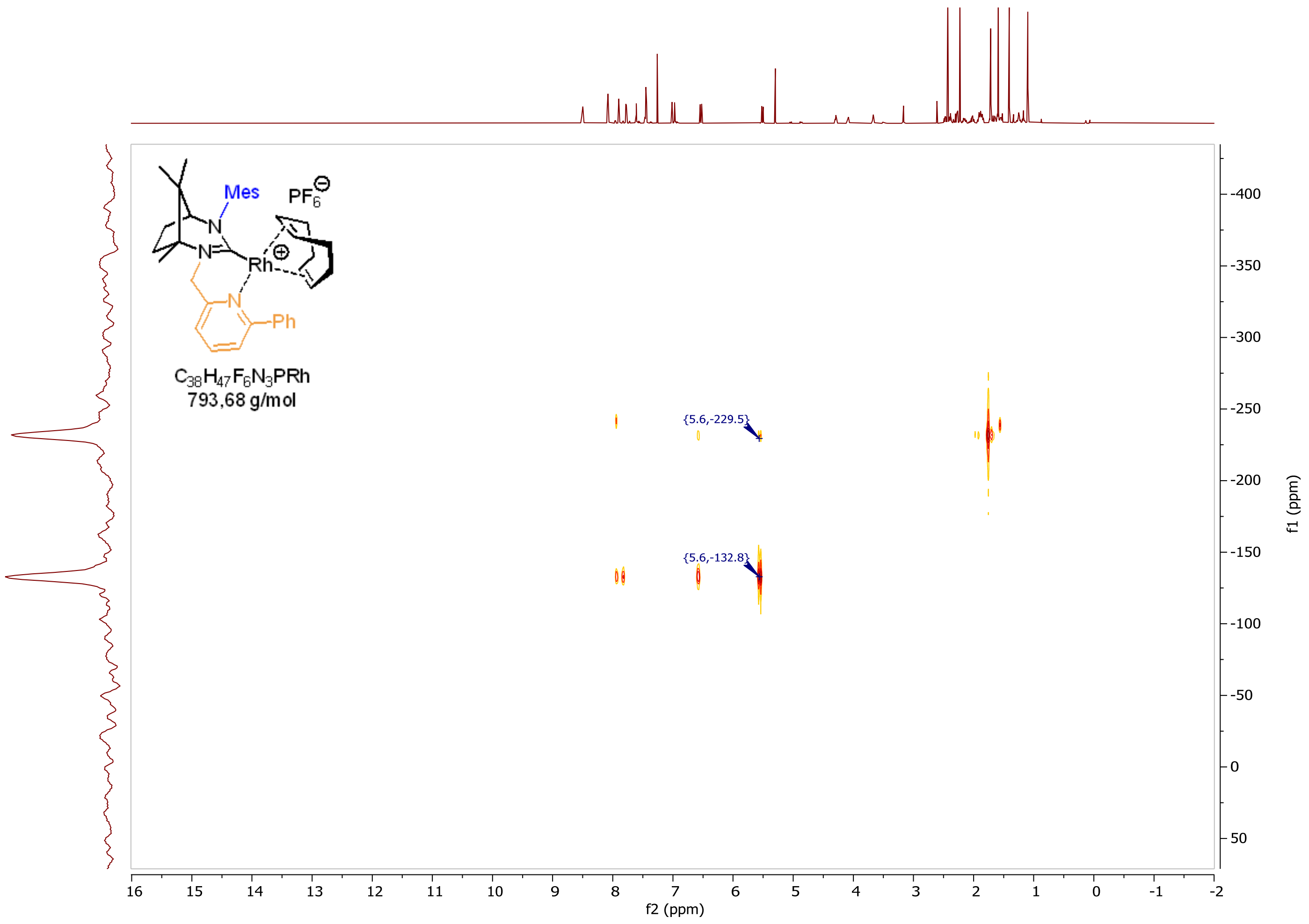

Mes
$PF_6^{\ominus}$
N
N
Rh
N
Ph
$C_{38}H_{47}F_6N_3PRh$
793,68 g/mol
{5.6,-229.5}
{5.6,-132.8}
f1 (ppm)
f2 (ppm)

^{1}H NMR (600 MHz, $CDCl_3$), ^{13}C{^{1}H} NMR (151 MHz, $CDCl_3$) and ^{15}N HSQC NMR (61 MHz, $CDCl_3$) Analysis of **Complex Rh5bt**

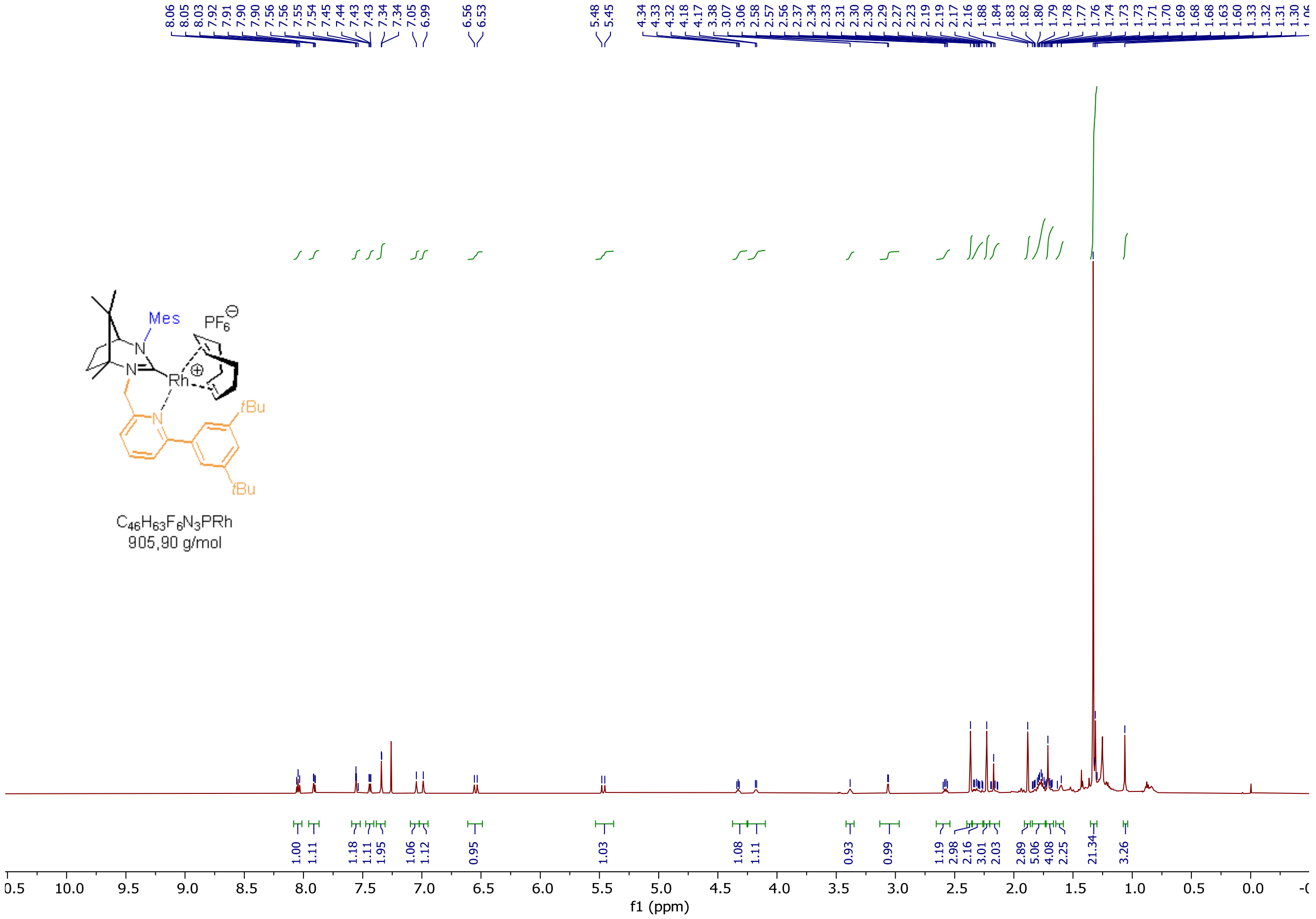

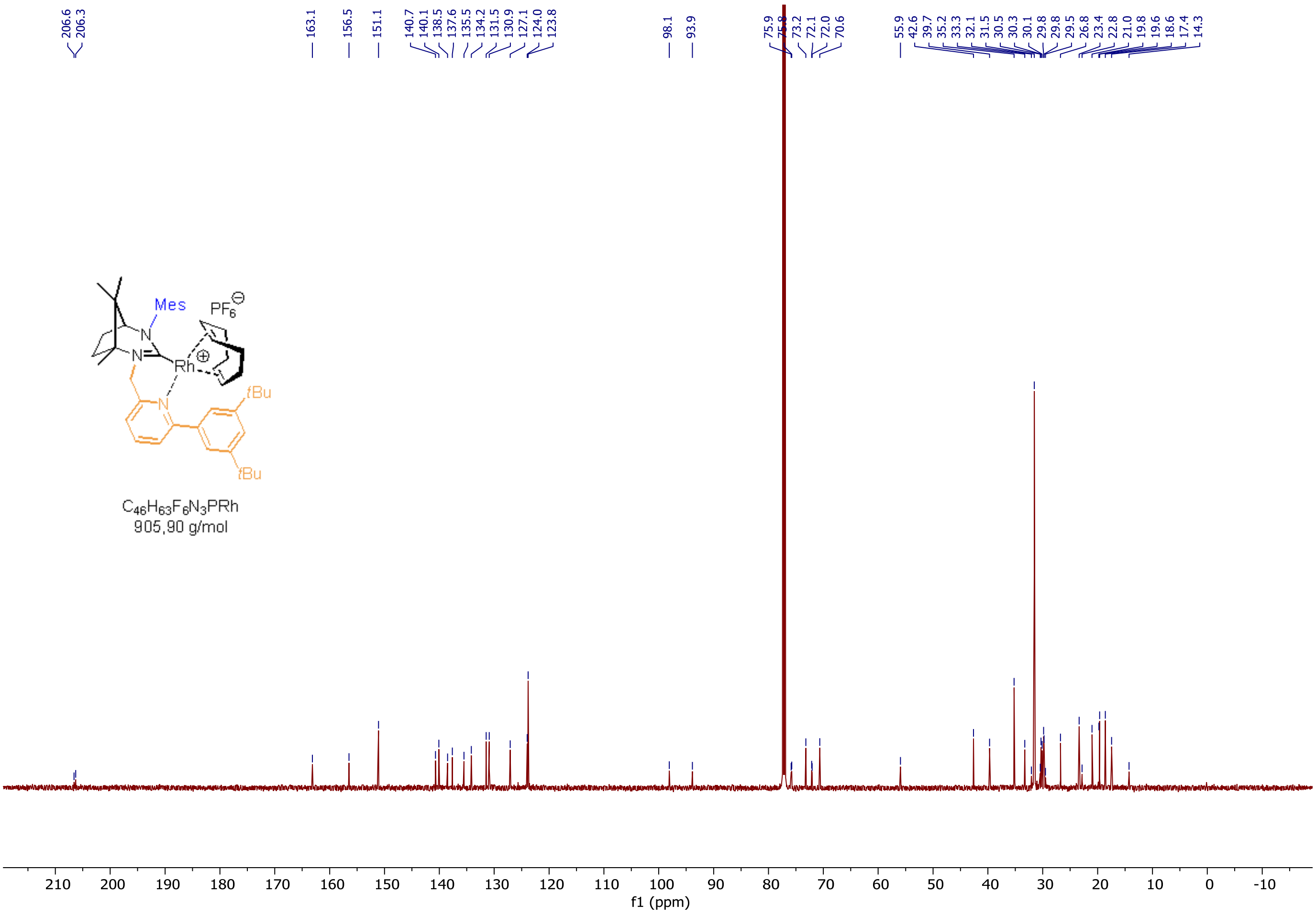

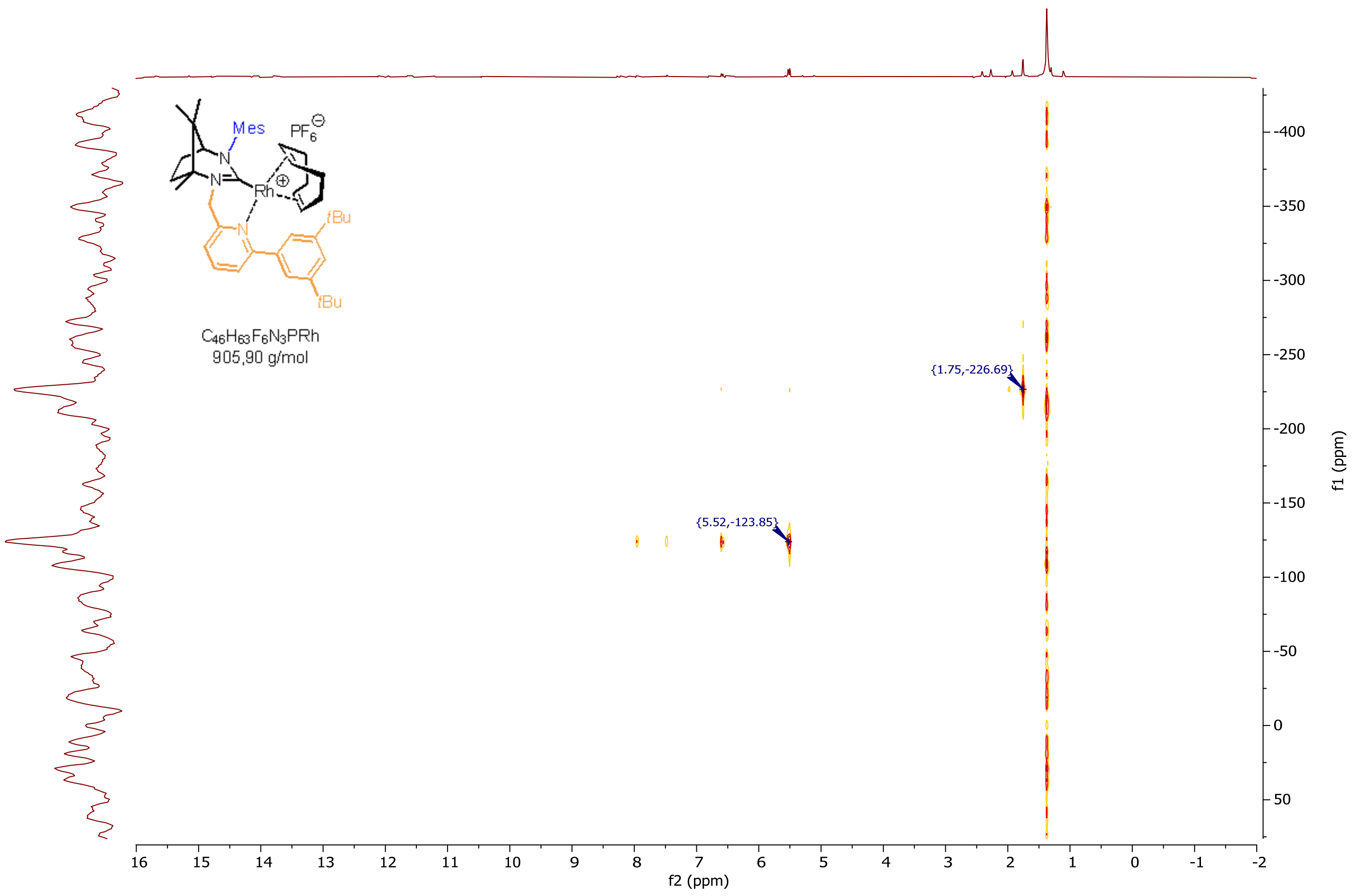
Mes
PF6
Rh
N
tBu
tBu
C46H63F6N3PRh
905,90 g/mol
{1.75,-226.69}
{5.52,-123.85}
f2 (ppm)
f1 (ppm)

^{1}H NMR (600 MHz, $CDCl_3$), $^{13}C\{^{1}H\}$ NMR (151 MHz, $CDCl_3$) and ^{15}N HSQC NMR (61 MHz, $CDCl_3$) Analysis of Compound **RhPh₂(SIMes)**

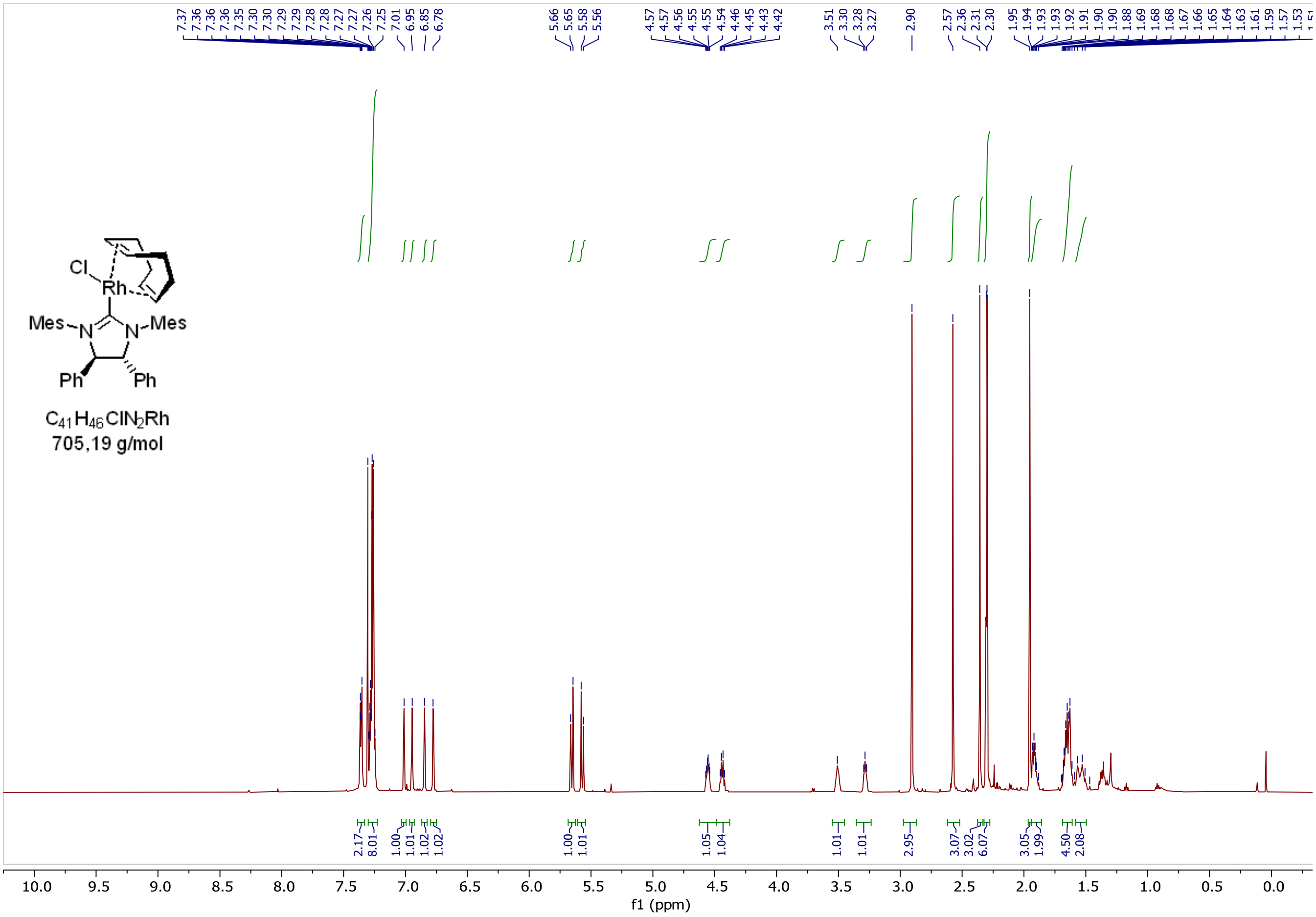

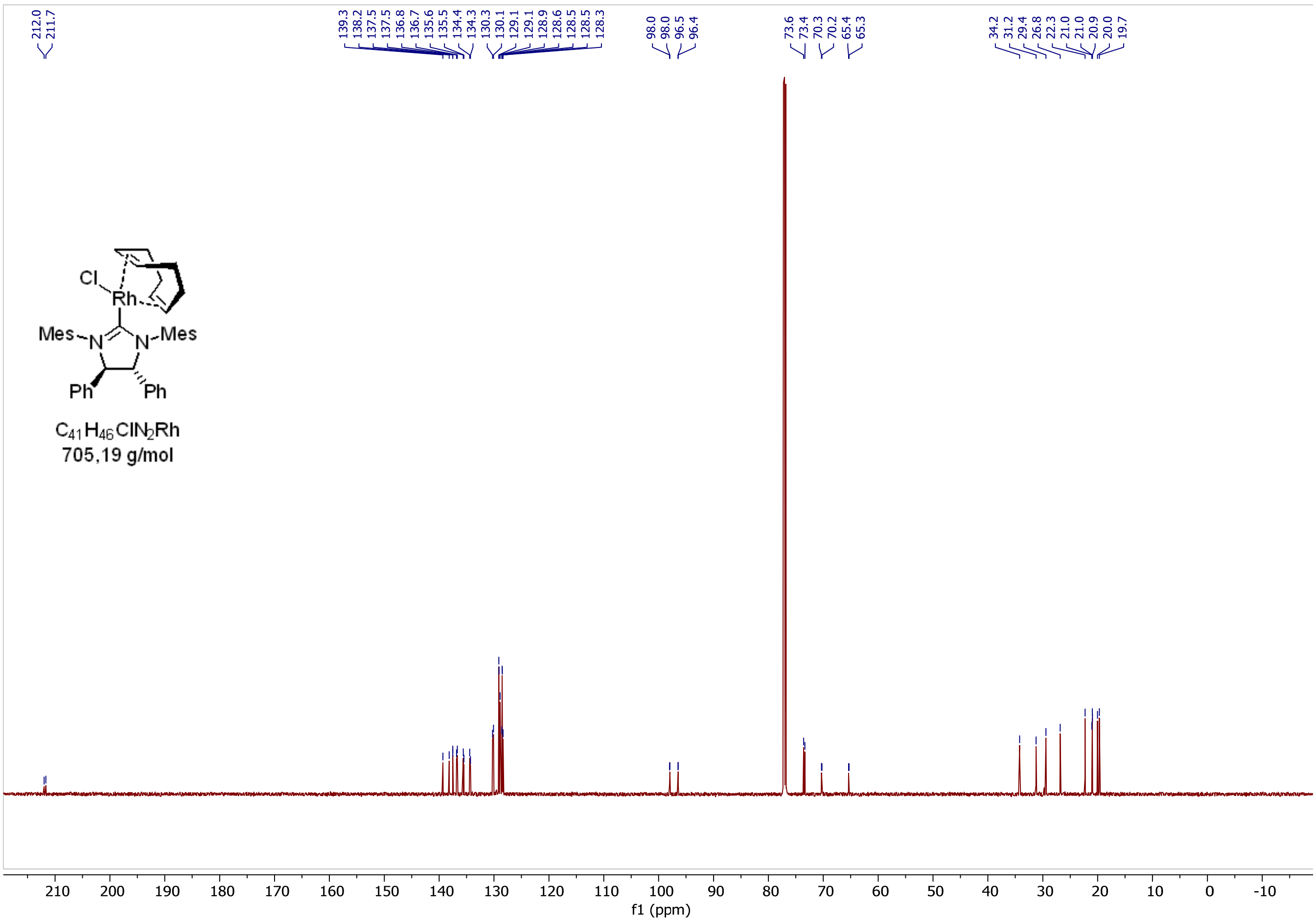

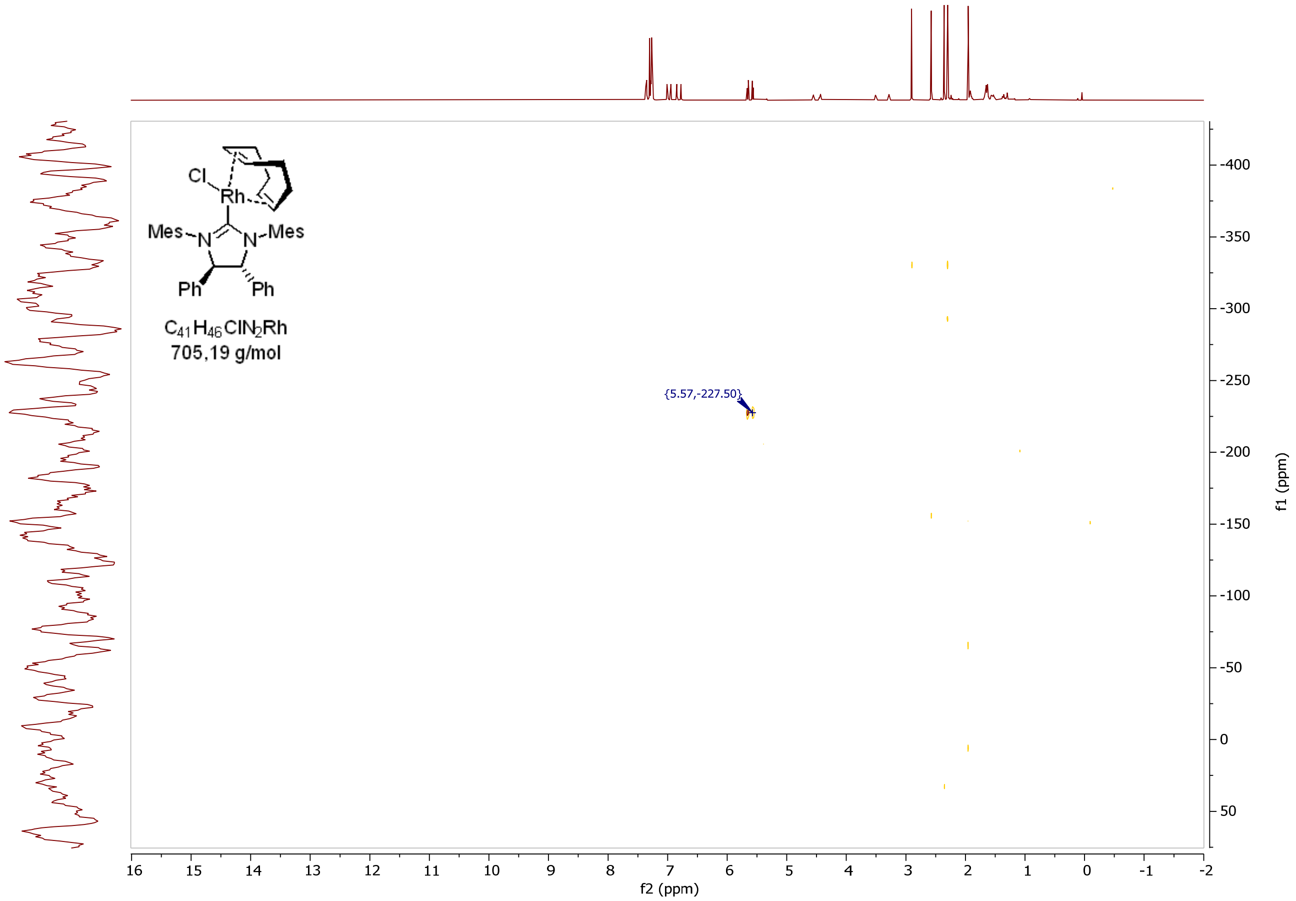
Cl
Rh
Mes
N
N
Mes
Ph
Ph
$C_{41}H_{46}ClN_2Rh$
705,19 g/mol
{5.57,-227.50}
f1 (ppm)
f2 (ppm)

^{1}H NMR (600 MHz, $CDCl_3$), $^{13}C\{^{1}H\}$ NMR (151 MHz, $CDCl_3$) and ^{15}N HSQC NMR (61 MHz, $CDCl_3$) Analysis of Compound **Rh(SIMes)**

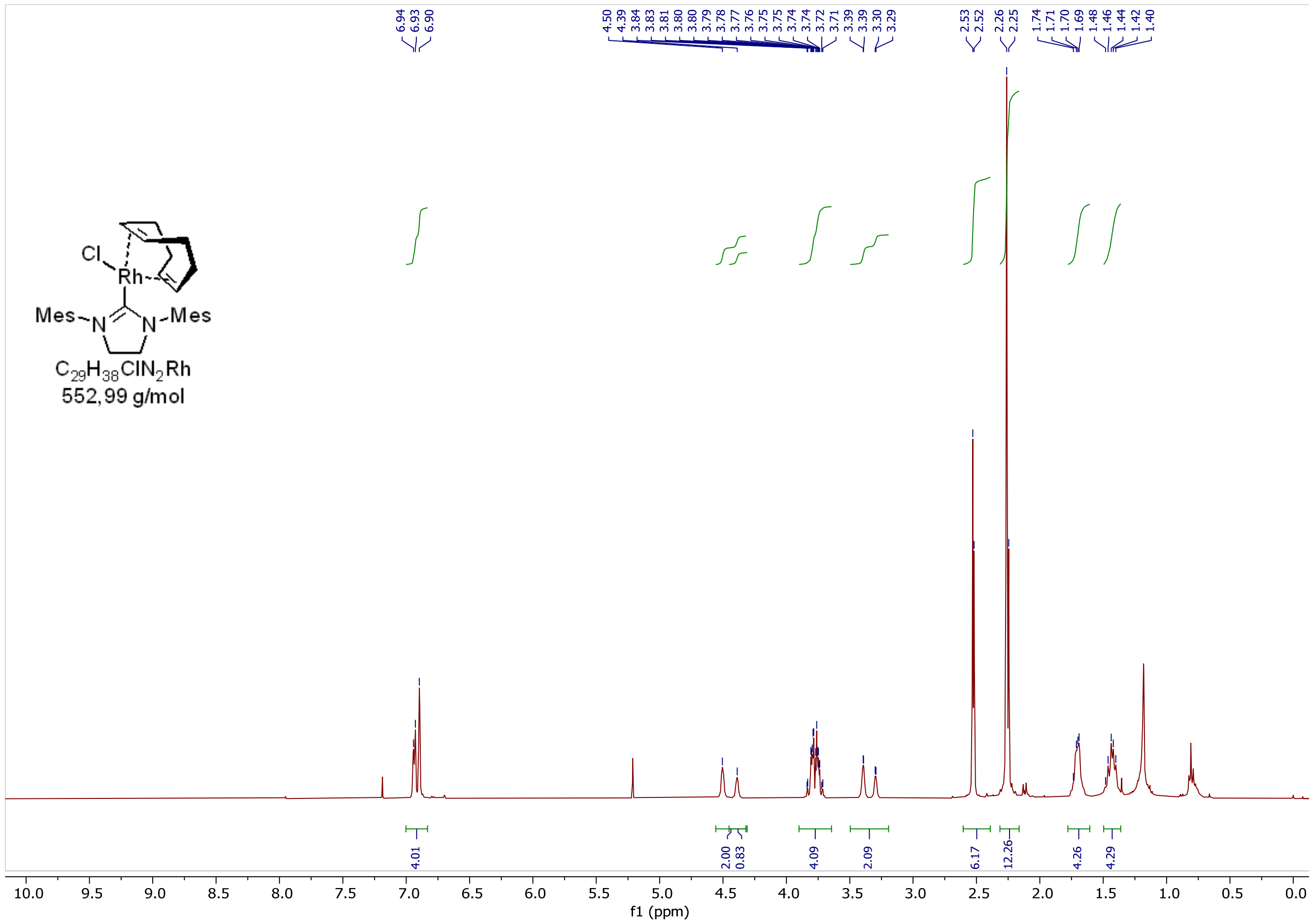

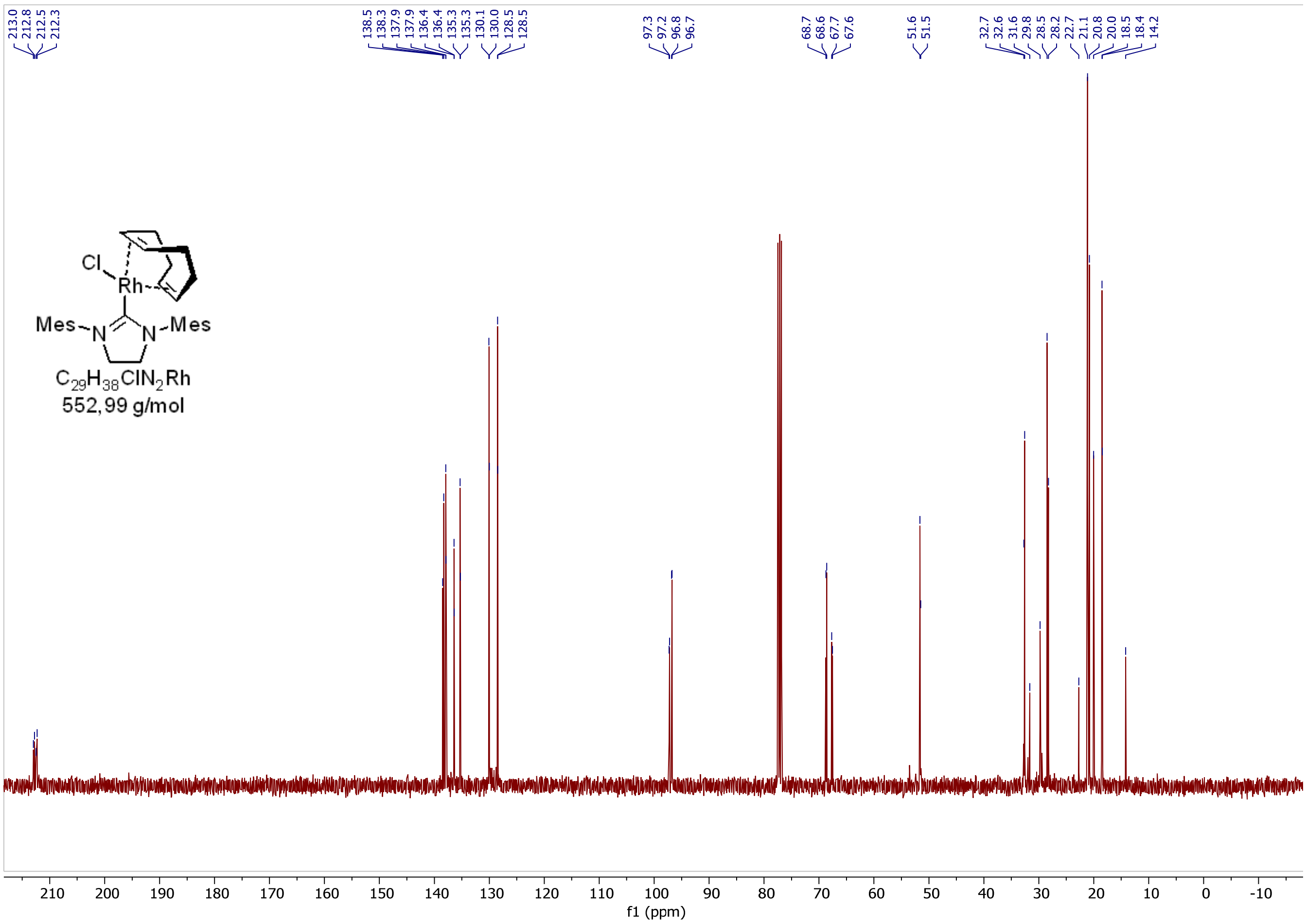

¹H NMR (400 MHz, $CDCl_3$) and $^{13}C\{^{1}H\}$ NMR (101 MHz, $CDCl_3$) Analysis of Compound **6a**

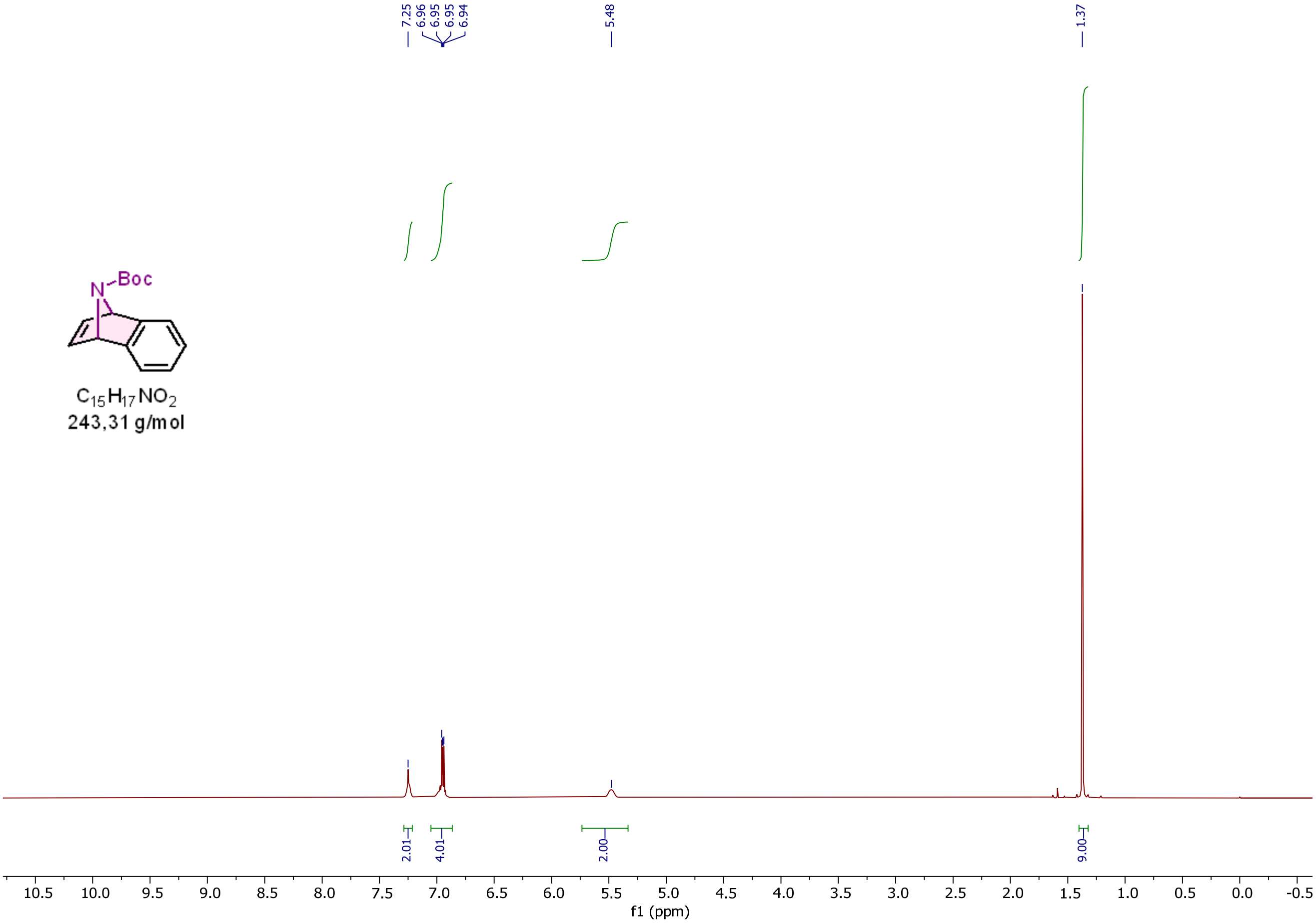

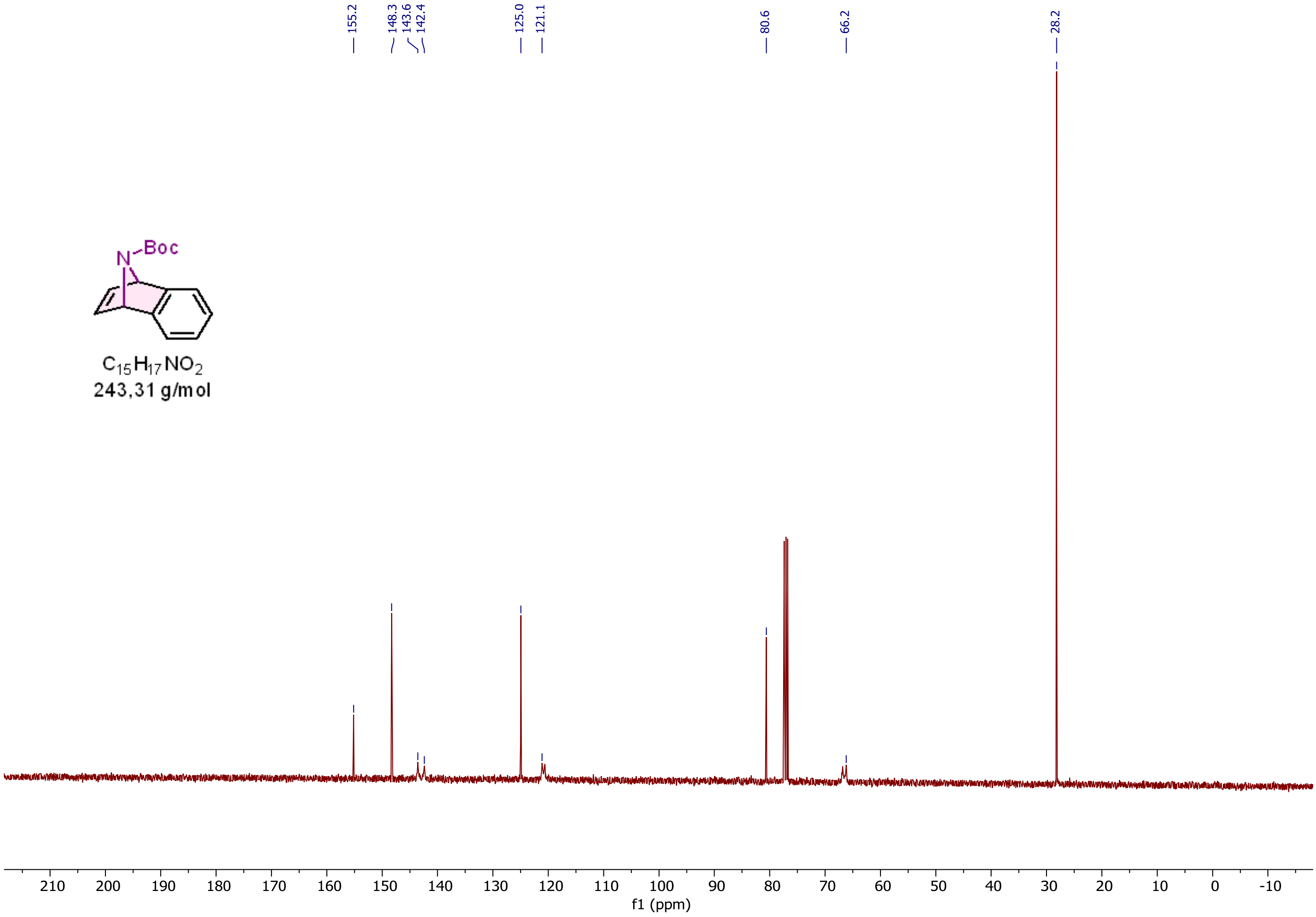

^1H NMR (400 MHz, $CDCl_3$) and $^{13}C\{^1H\}$ NMR (101 MHz, $CDCl_3$) Analysis of Compound **6b**

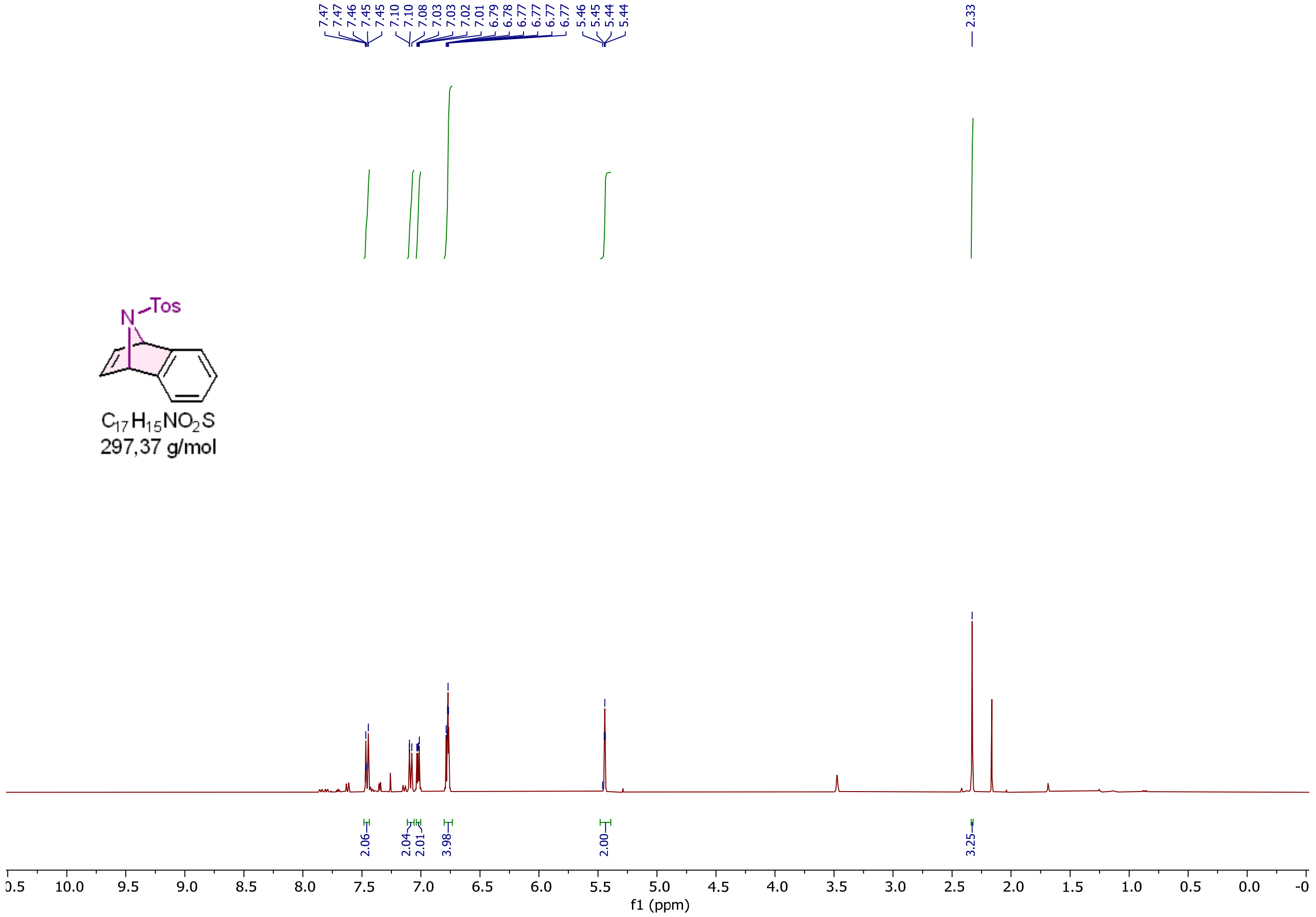

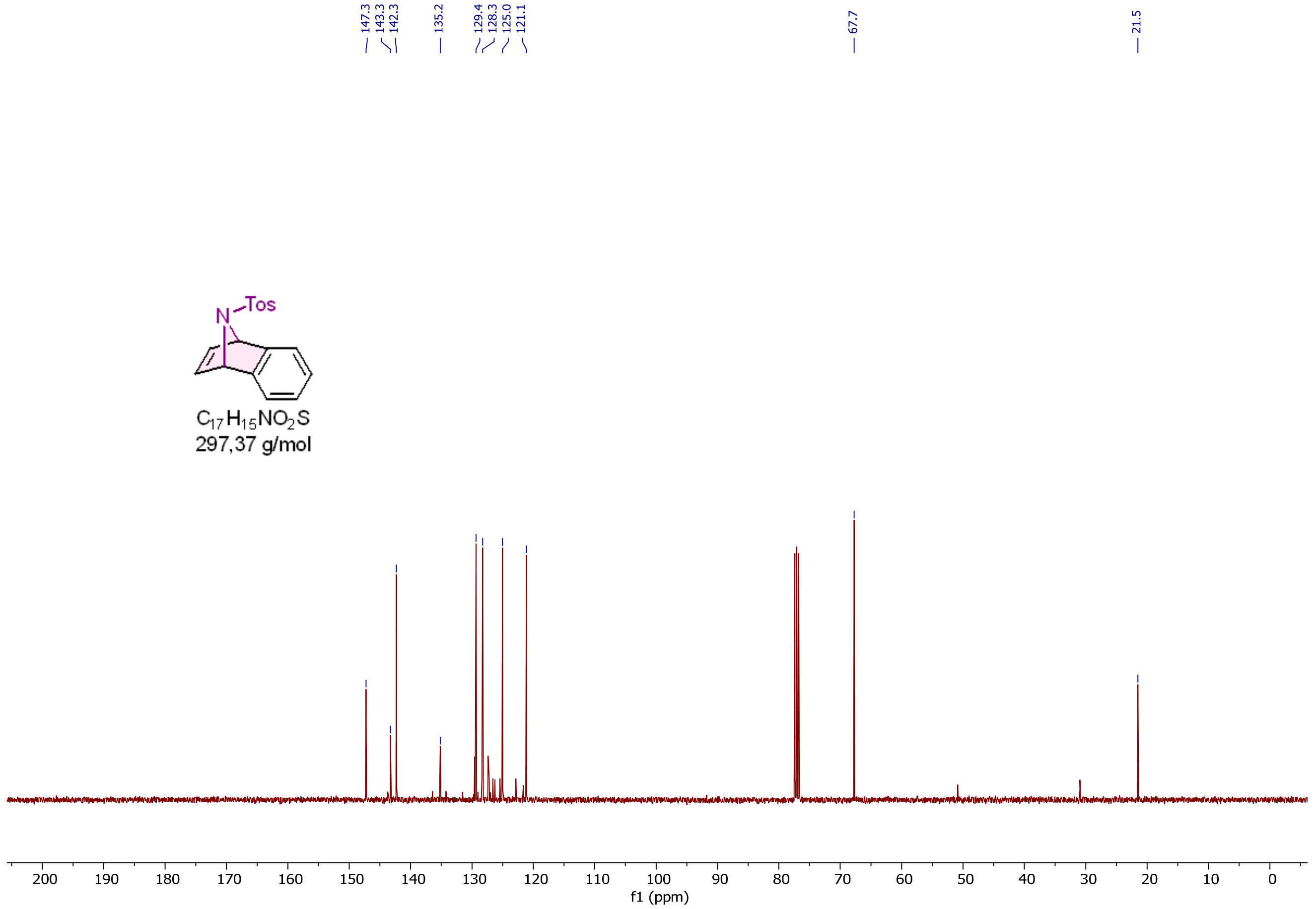

^{1}H NMR (400 MHz, $CDCl_3$) and $^{13}C\{^{1}H\}$ NMR (101 MHz, $CDCl_3$) Analysis of Compound **6c**

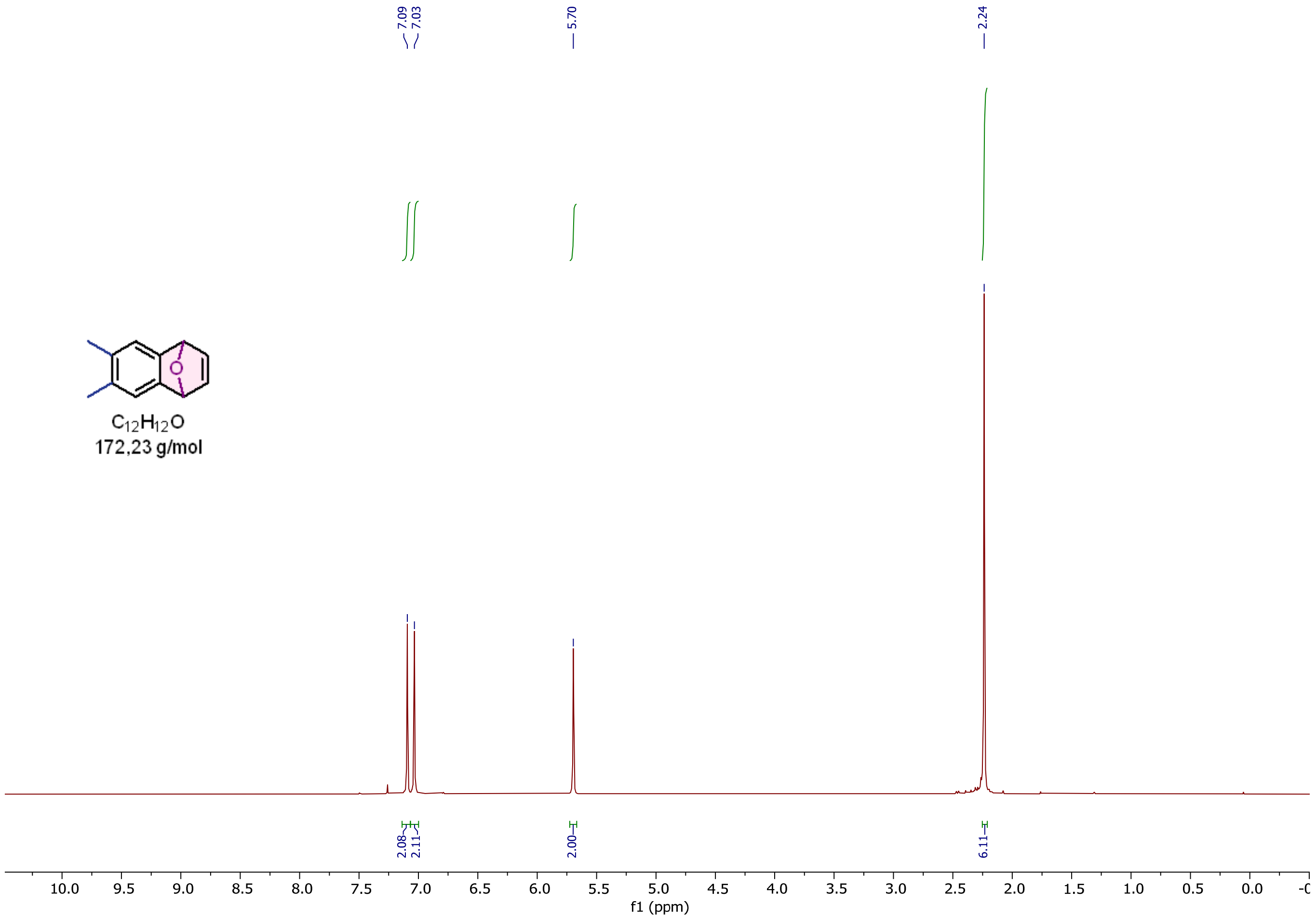

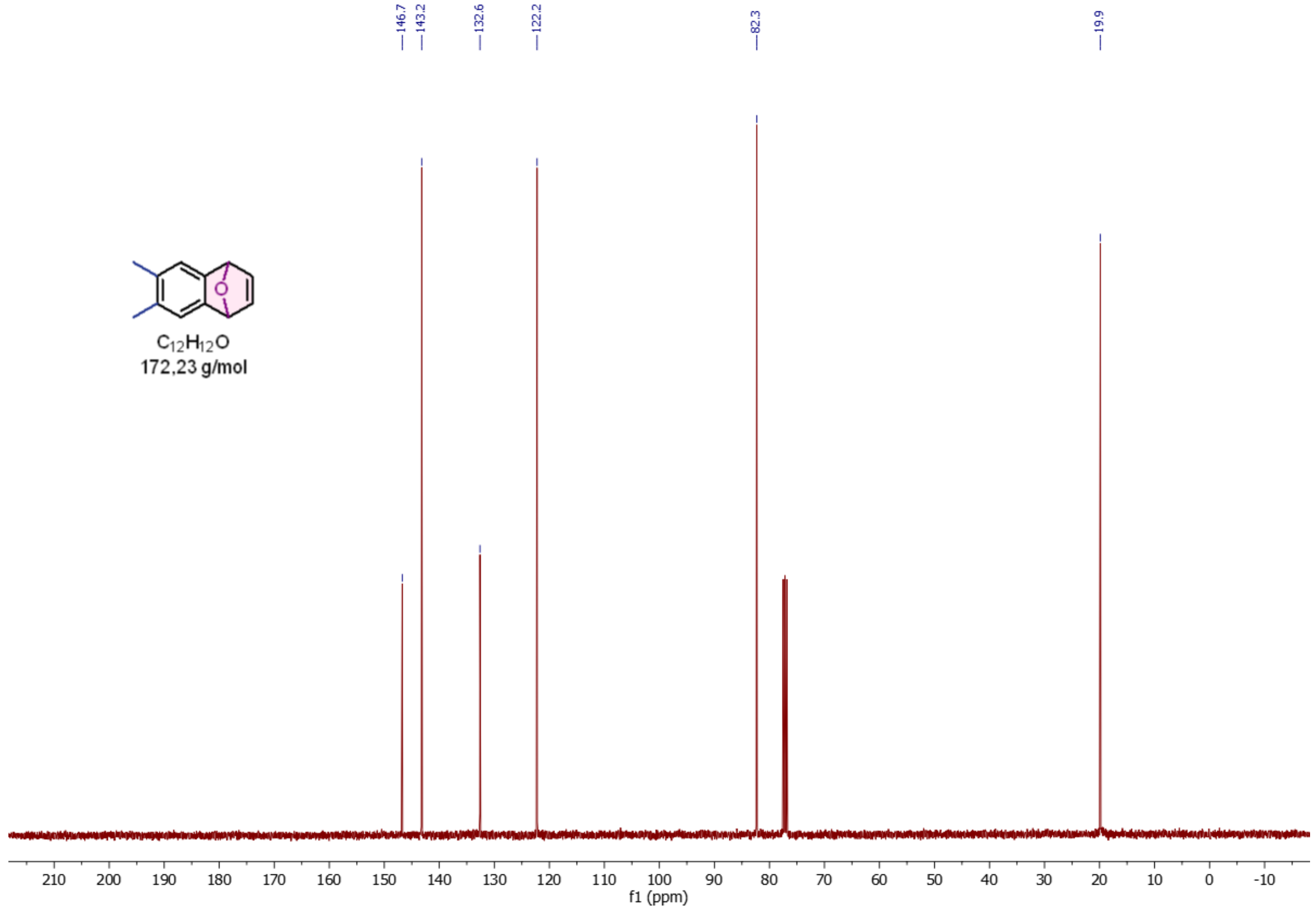

^{1}H NMR (400 MHz, $CDCl_3$) and $^{13}C\{^{1}H\}$ NMR (101 MHz, $CDCl_3$) Analysis of Compound **6d**

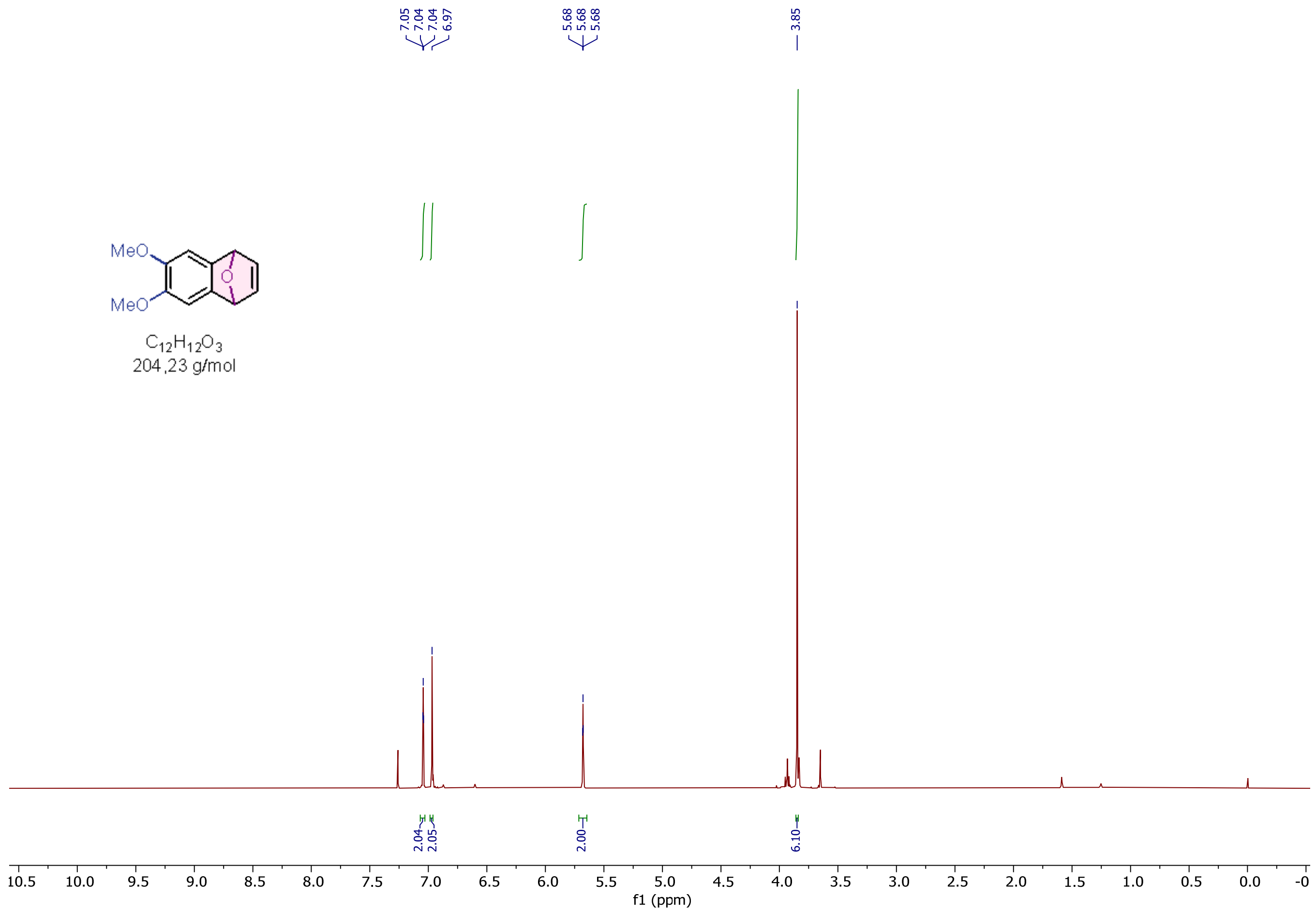

7.05
7.04
7.04
6.97
5.68
5.68
5.68
3.85
MeO
MeO
O
$C_{12}H_{12}O_3$
204,23 g/mol
2.04
2.05
2.00
6.10
10.5
10.0
9.5
9.0
8.5
8.0
7.5
7.0
6.5
6.0
5.5
5.0
4.5
4.0
3.5
3.0
2.5
2.0
1.5
1.0
0.5
0.0
-0
f1 (ppm)

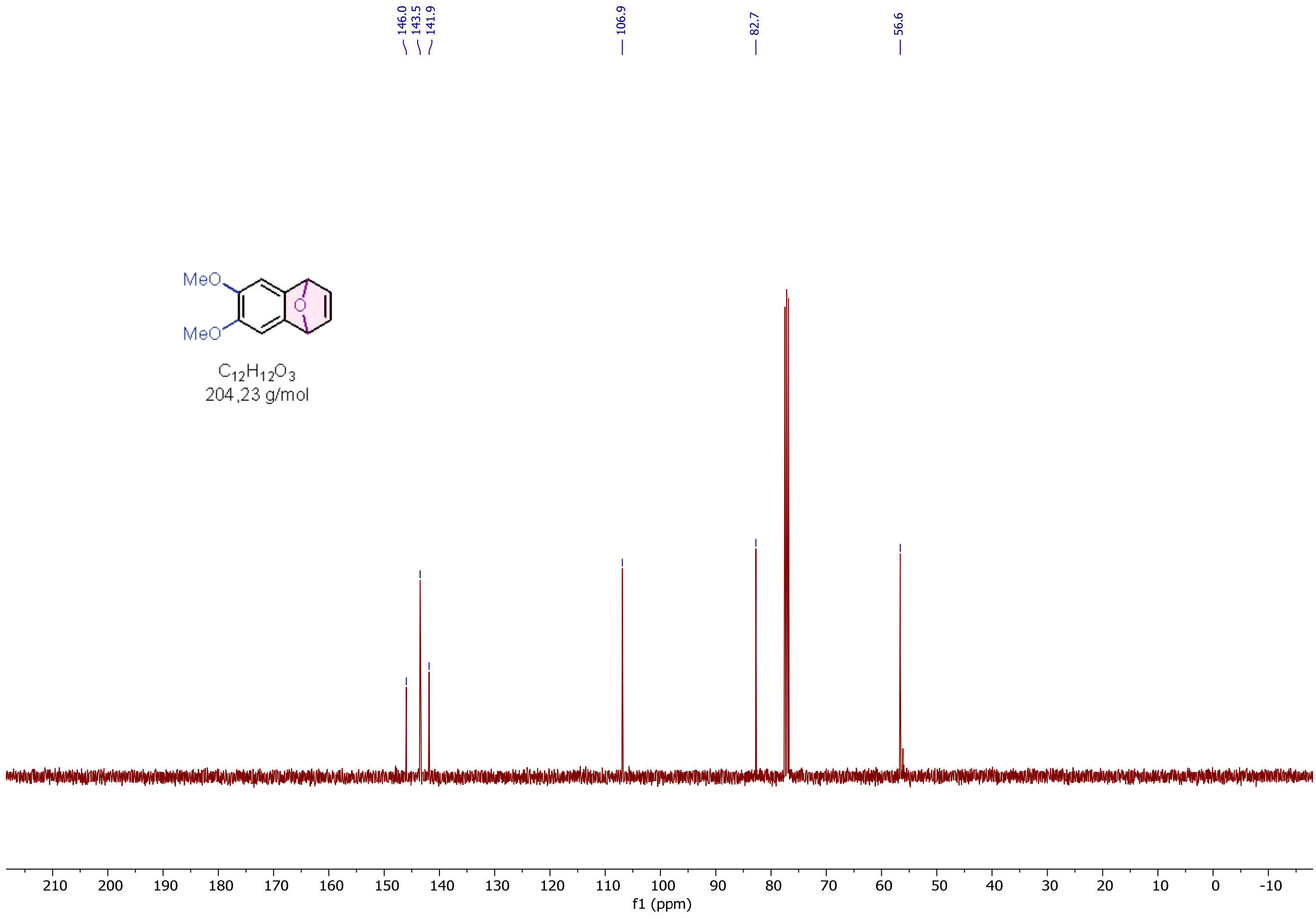

146.0
143.5
141.9
106.9
82.7
56.6
MeO
MeO
O
$C_{12}H_{12}O_3$
204,23 g/mol
210
200
190
180
170
160
150
140
130
120
110
100
90
80
70
60
50
40
30
20
10
0
-10
f1 (ppm)

^{1}H NMR (400 MHz, $CDCl_3$) and $^{13}C\{^{1}H\}$ NMR (101 MHz, $CDCl_3$) Analysis of Compound **6e**

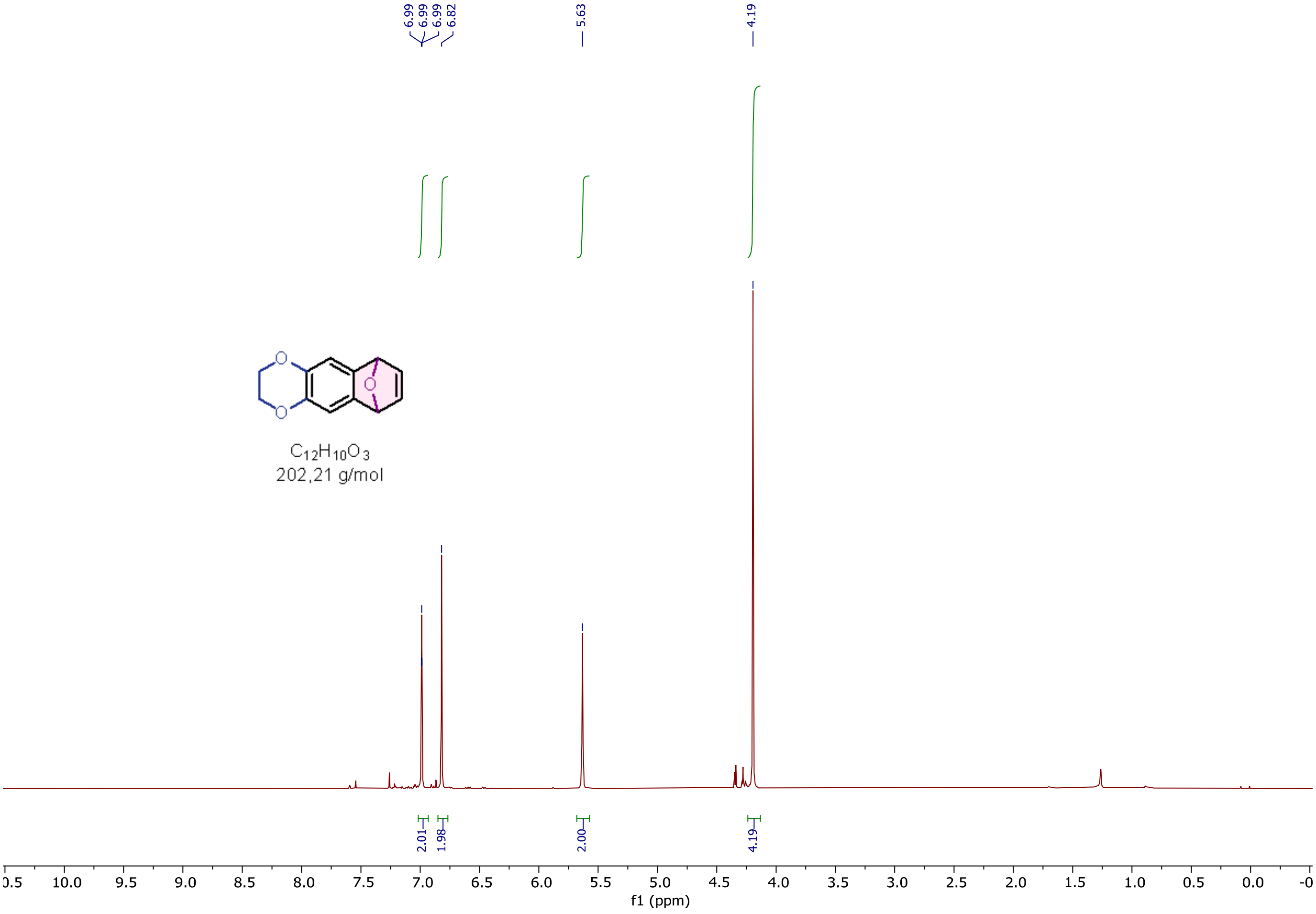

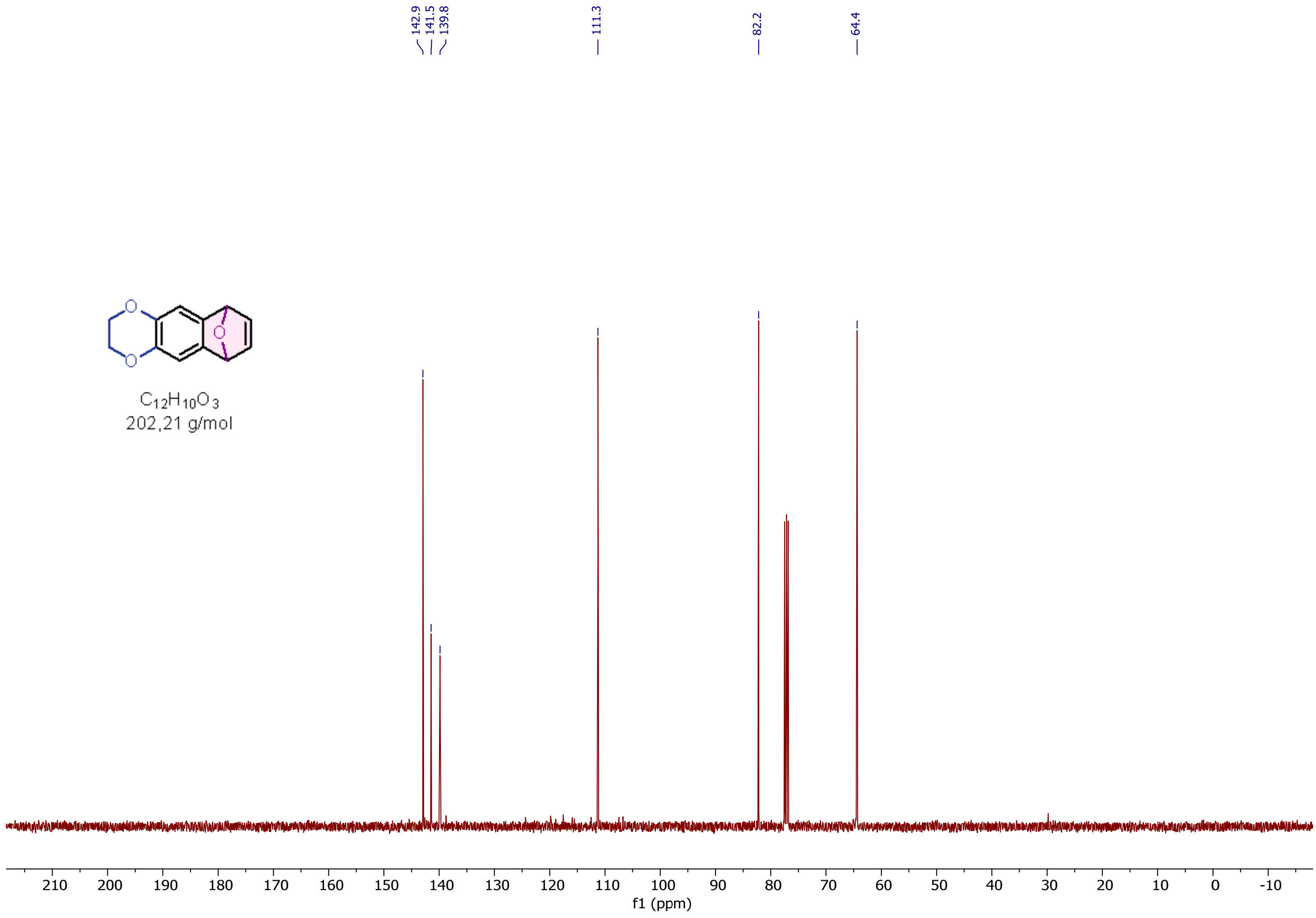

^{1}H NMR (400 MHz, CDCl$_3$) and ^{13}C{^{1}H} NMR (101 MHz, CDCl$_3$) Analysis of Compound **6f**

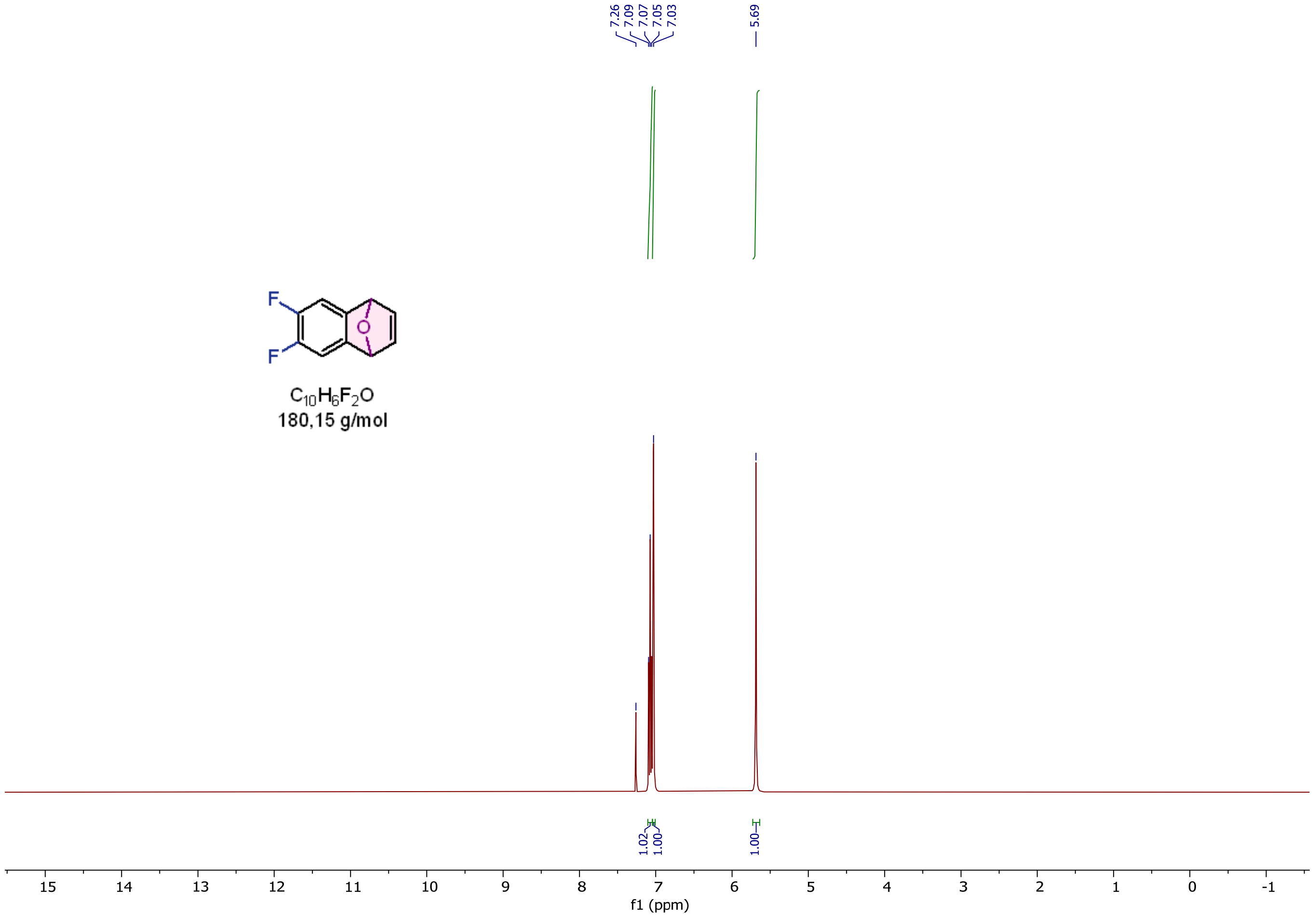

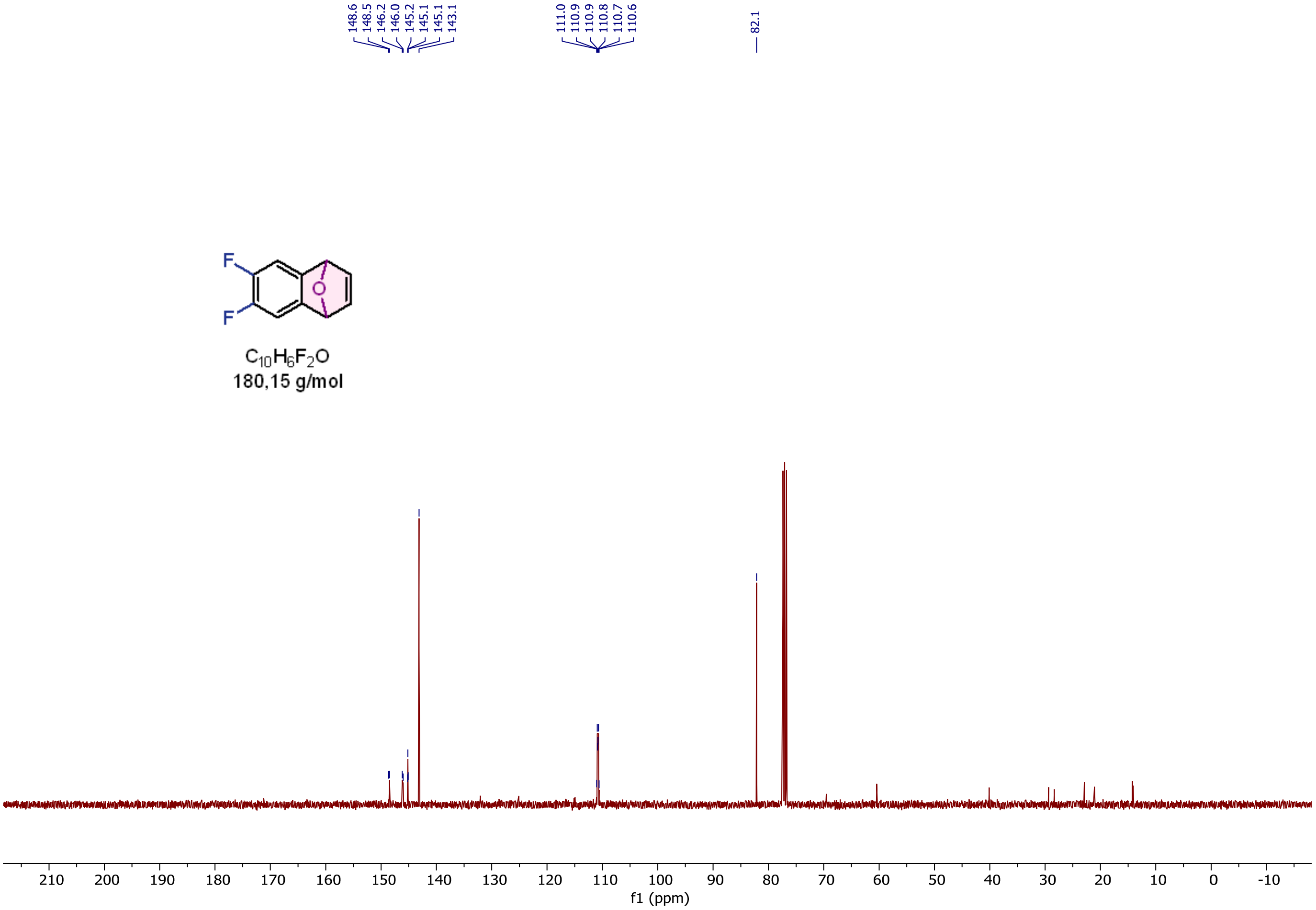

^{1}H NMR (400 MHz, $CDCl_3$) and ^{13}C{^{1}H} NMR (101 MHz, $CDCl_3$) Analysis of Compound **7aa**

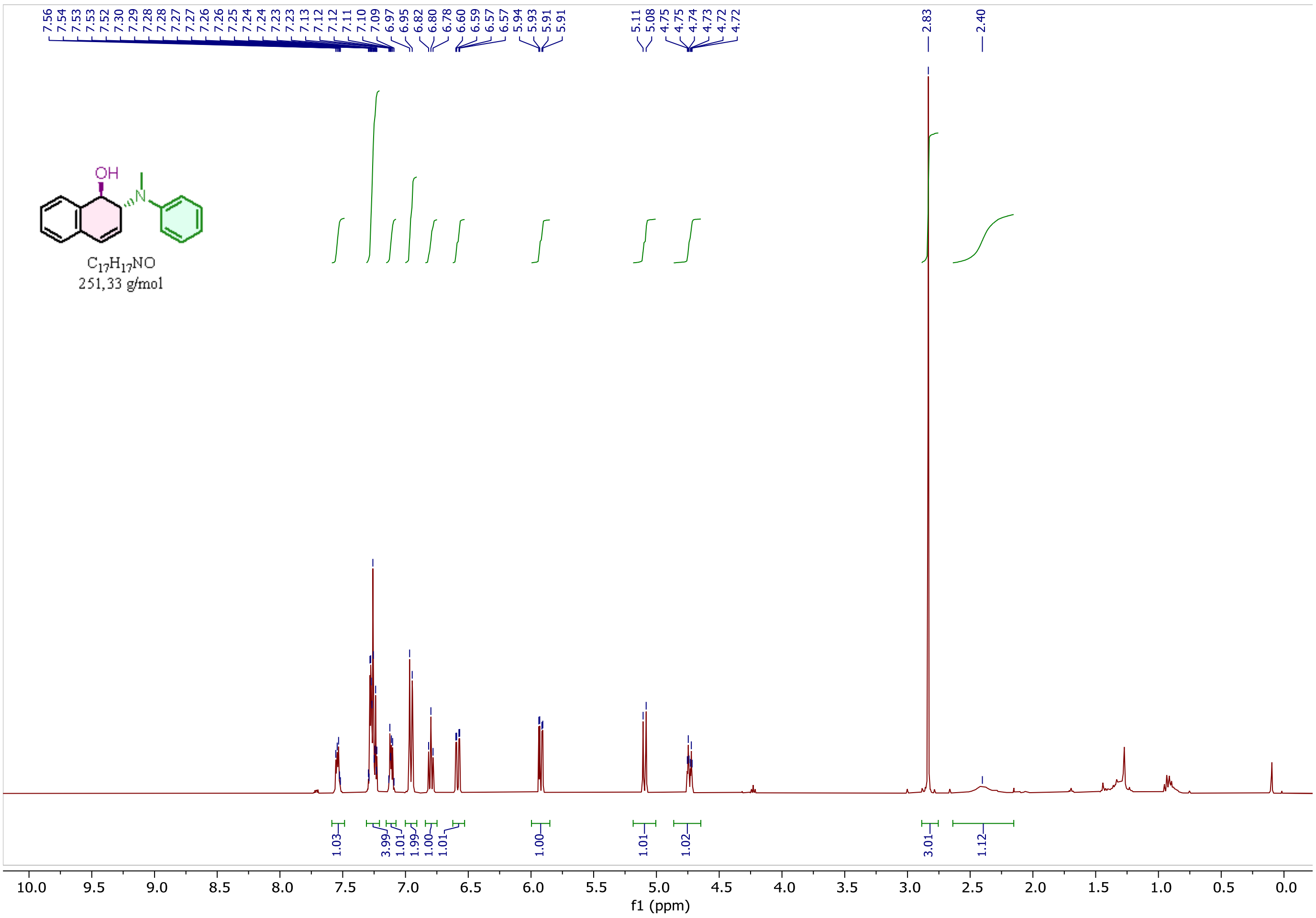

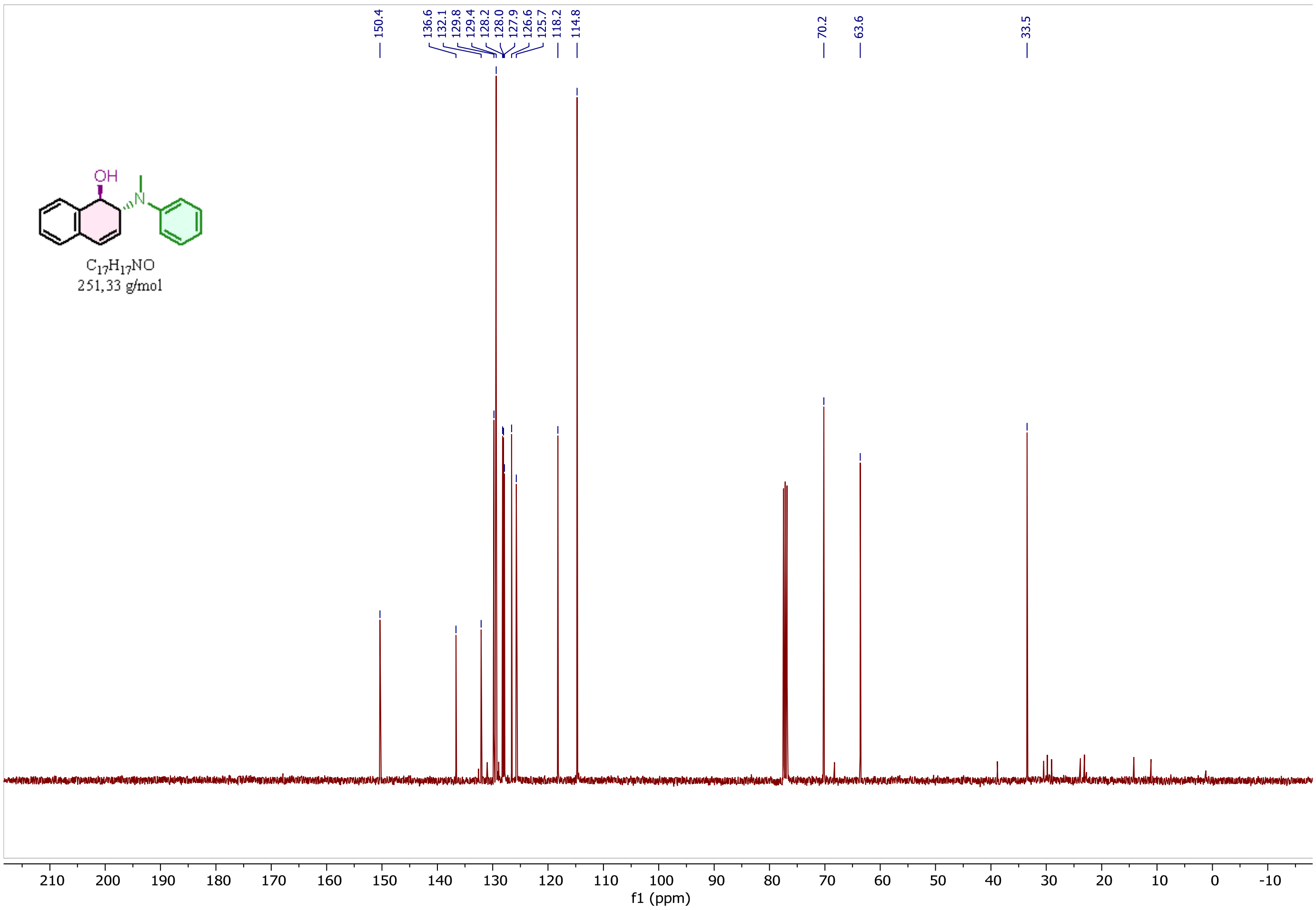

^{1}H NMR (400 MHz, $CDCl_3$) and $^{13}C\{^{1}H\}$ NMR (101 MHz, $CDCl_3$) Analysis of Compound **7ab**

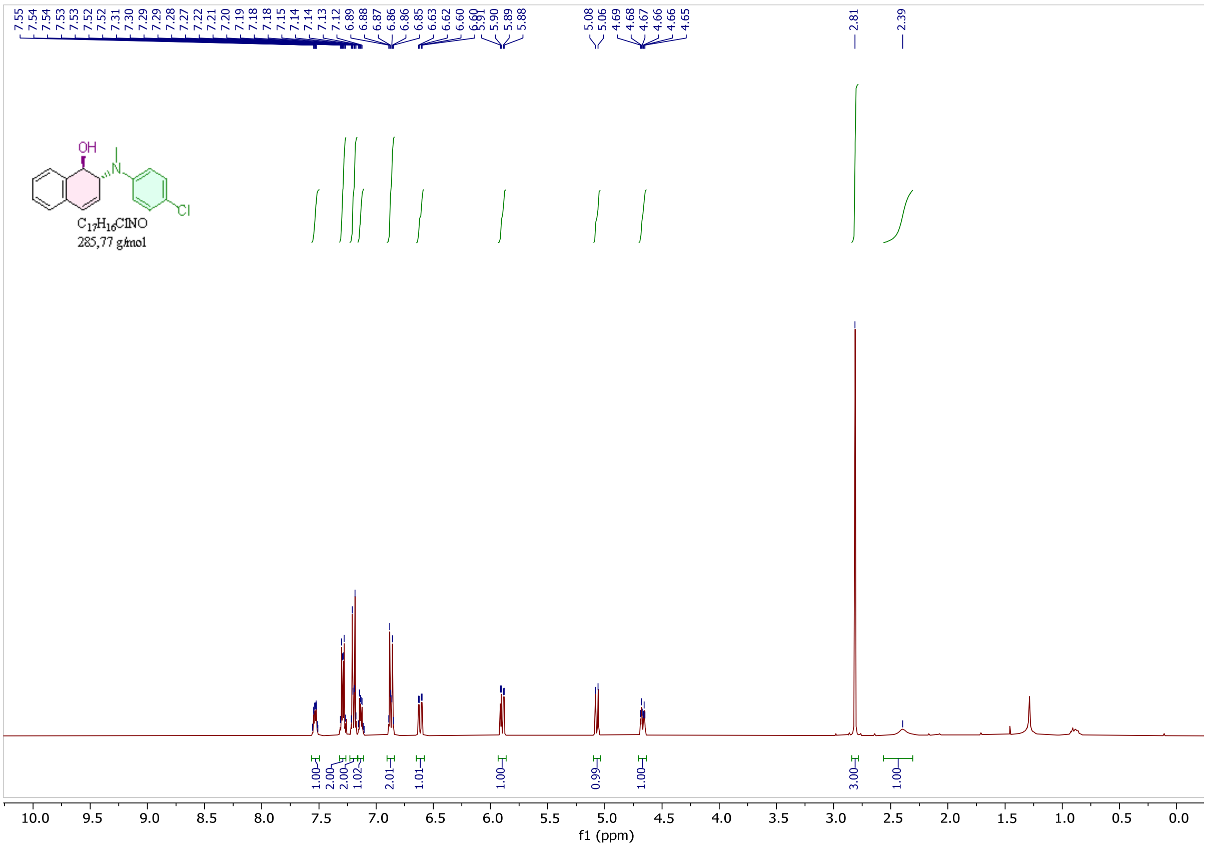

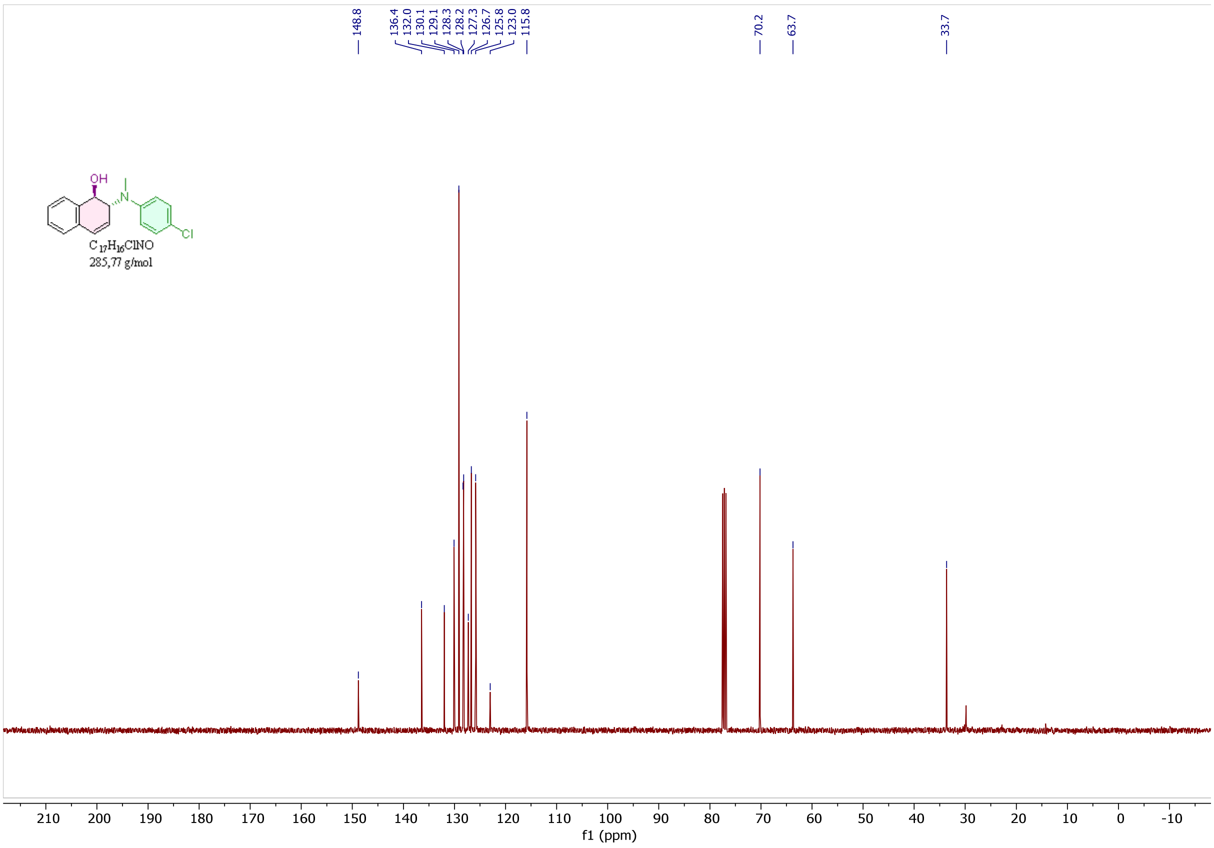

^1H NMR (400 MHz, $CDCl_3$) and $^{13}C\{^1H\}$ NMR (101 MHz, $CDCl_3$) Analysis of Compound **7ac**

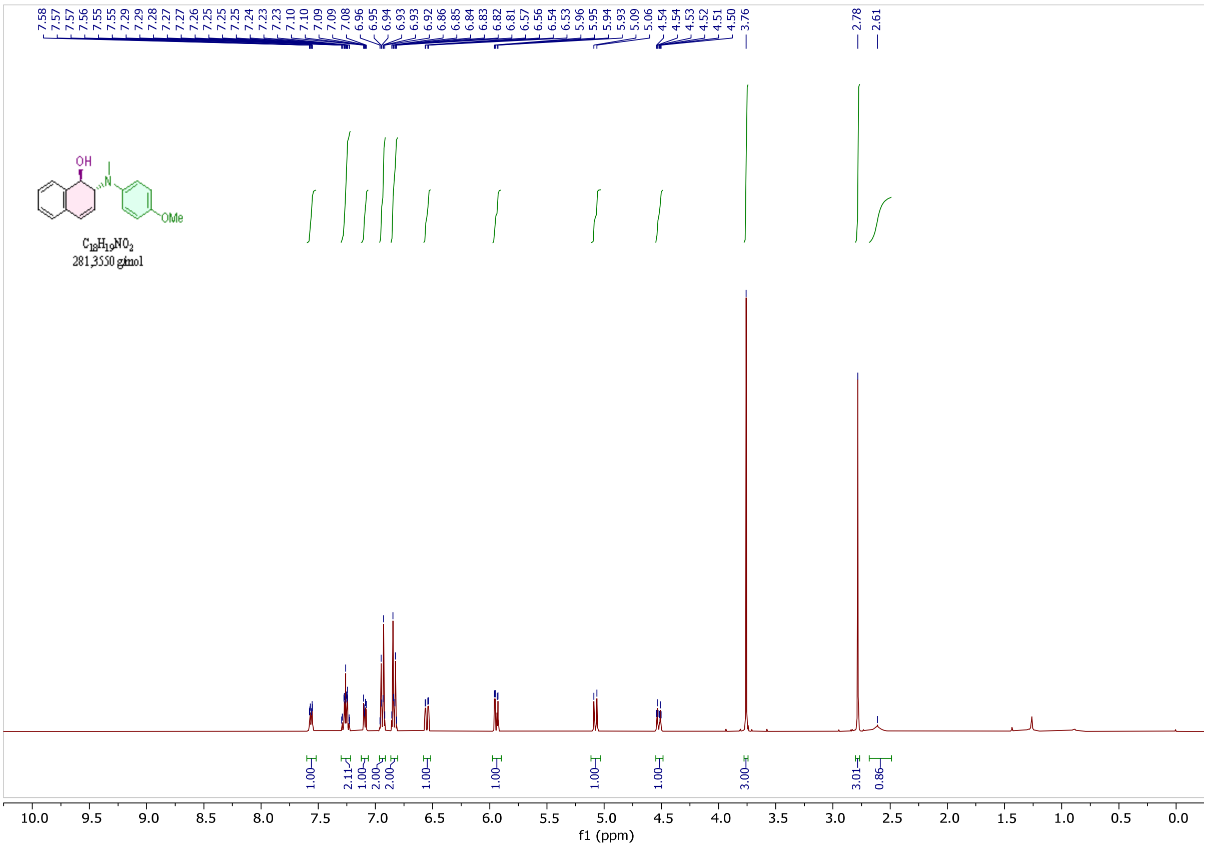

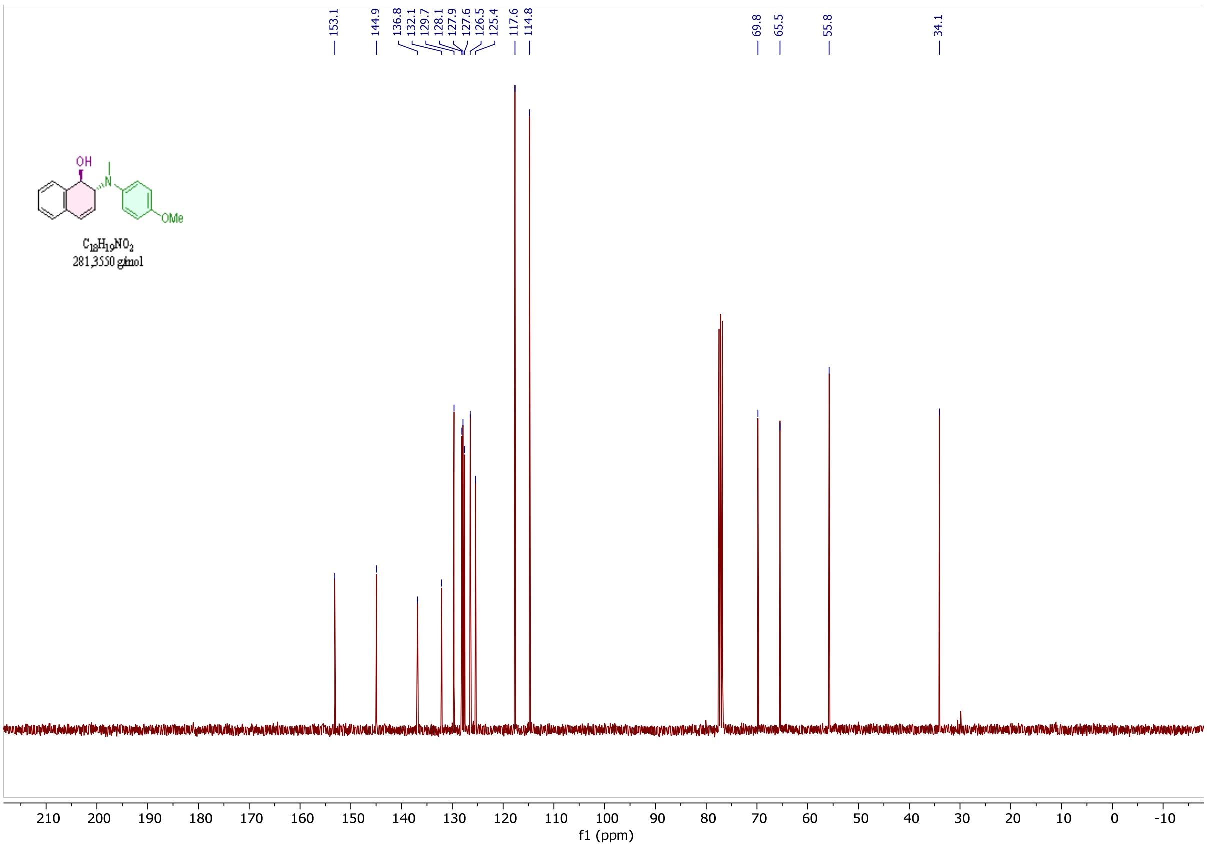

^1H NMR (400 MHz, $CDCl_3$) and $^{13}C\{^1H\}$ NMR (101 MHz, $CDCl_3$) Analysis of Compound **7ba**

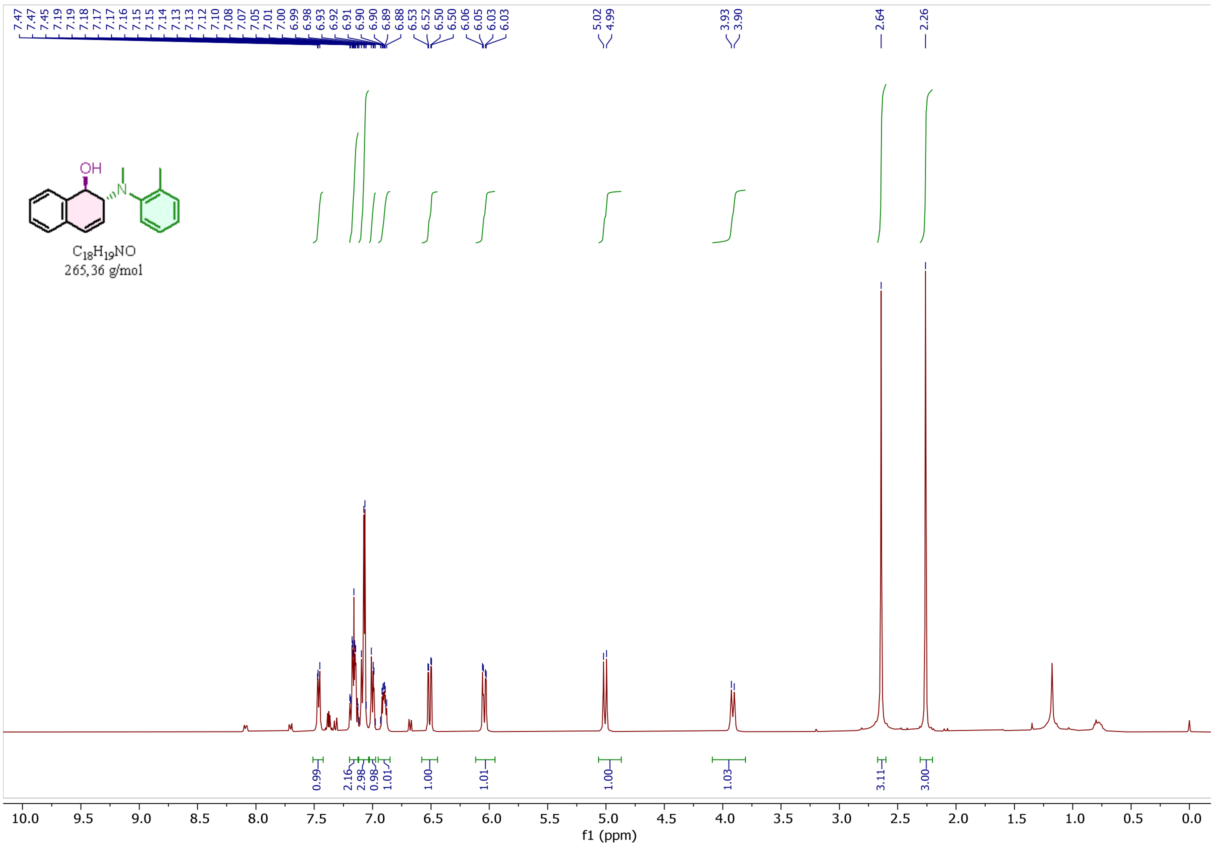

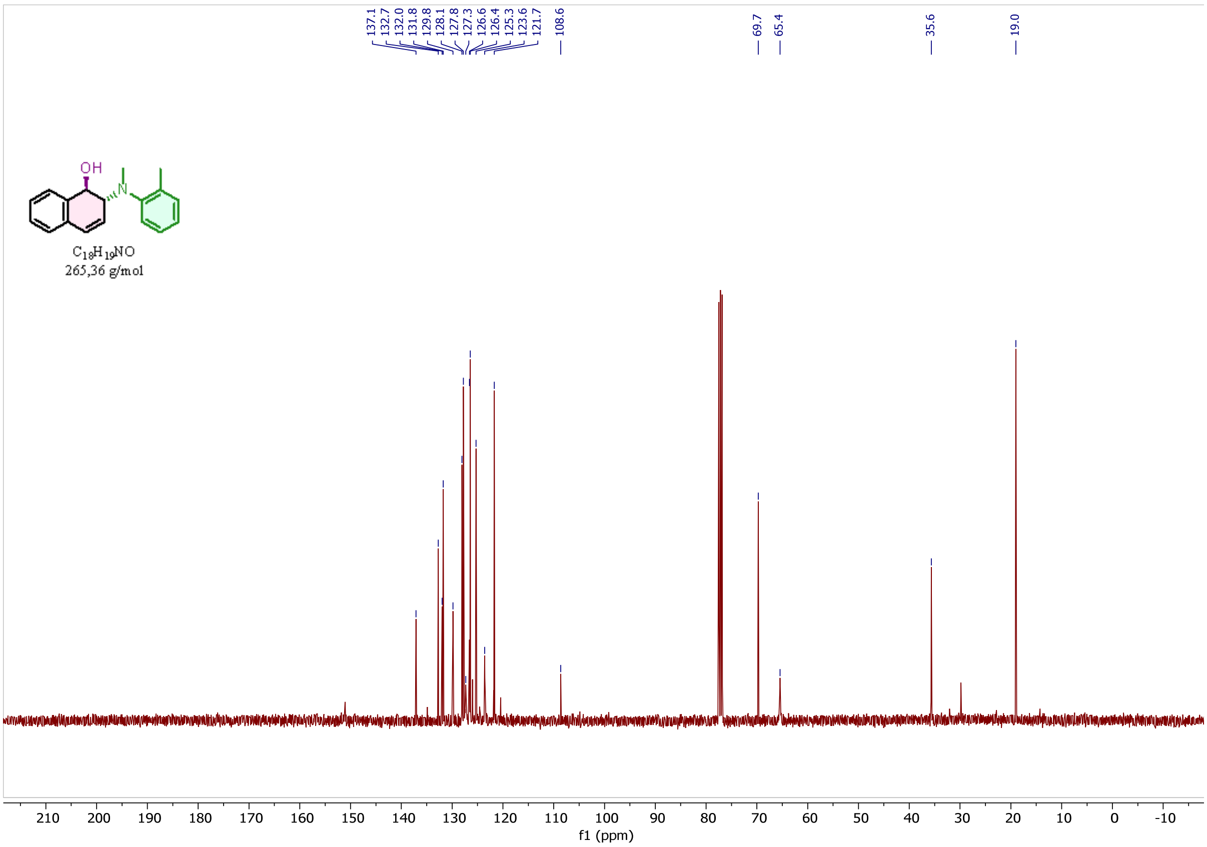

^{1}H NMR (400 MHz, $CDCl_3$) and $^{13}C\{^{1}H\}$ NMR (101 MHz, $CDCl_3$) Analysis of Compound **7bb**

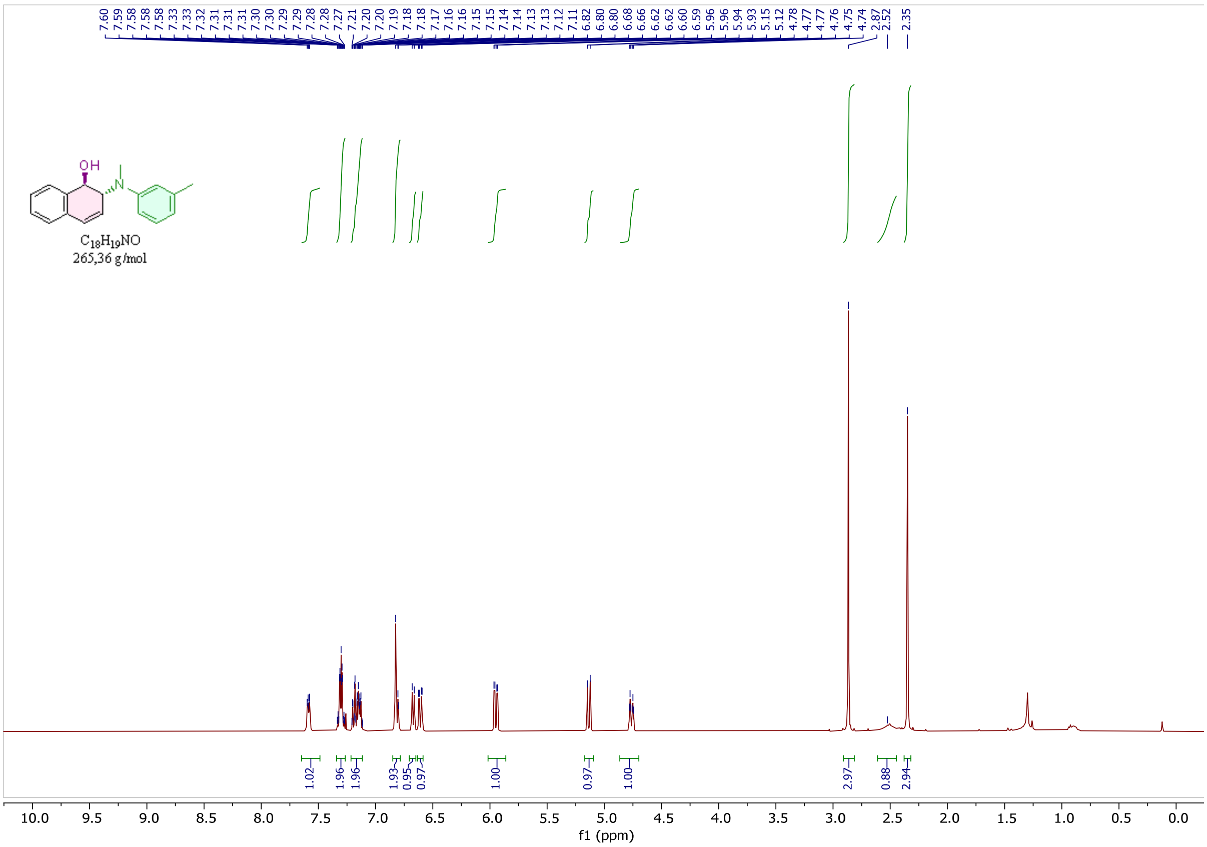

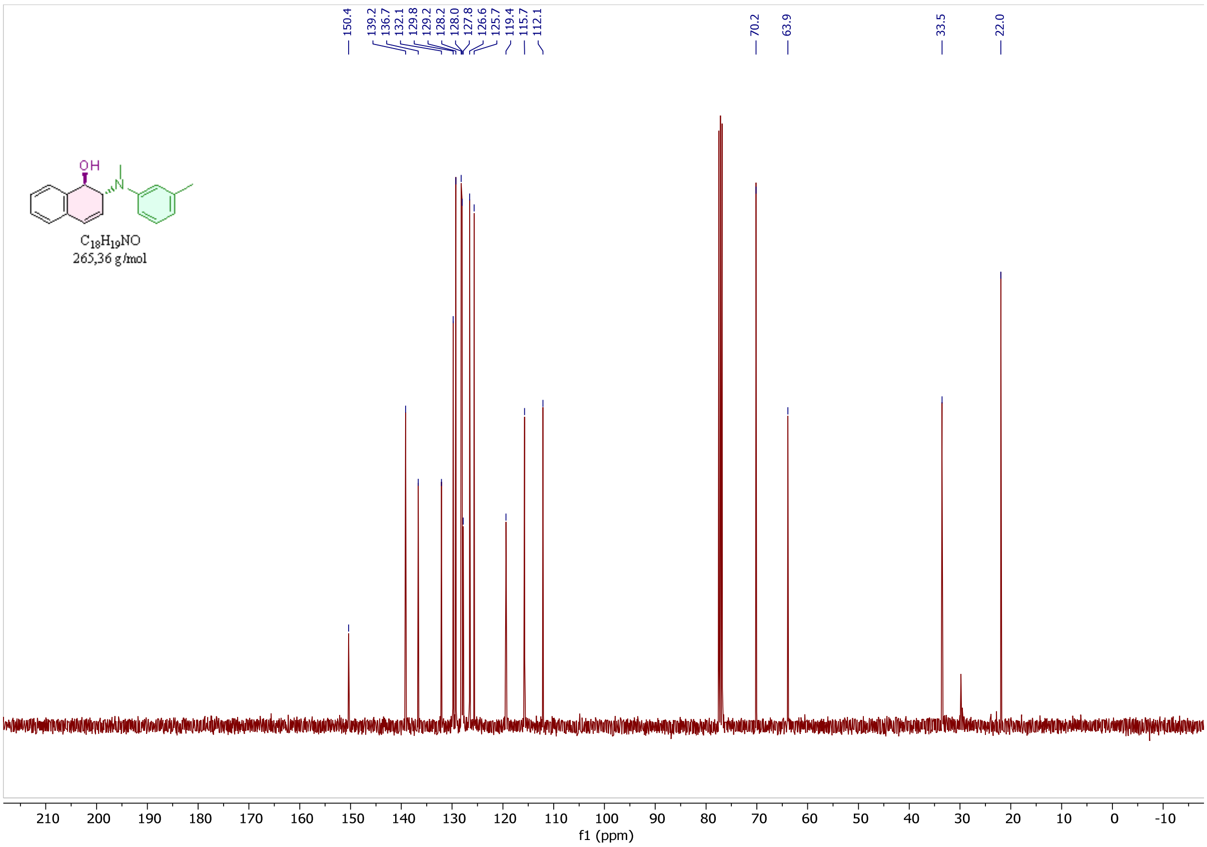

^1H NMR (400 MHz, $CDCl_3$) and $^{13}C\{^1H\}$ NMR (101 MHz, $CDCl_3$) Analysis of Compound **7bc**

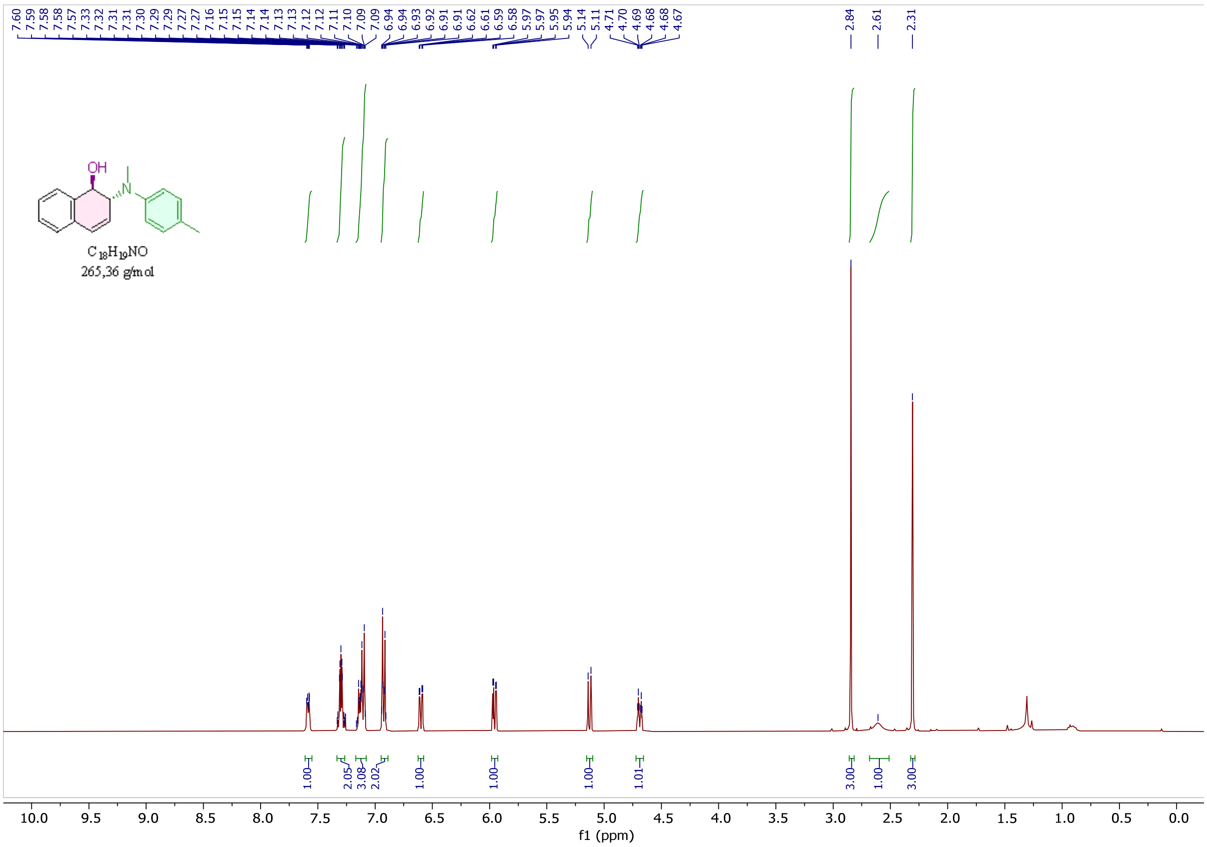

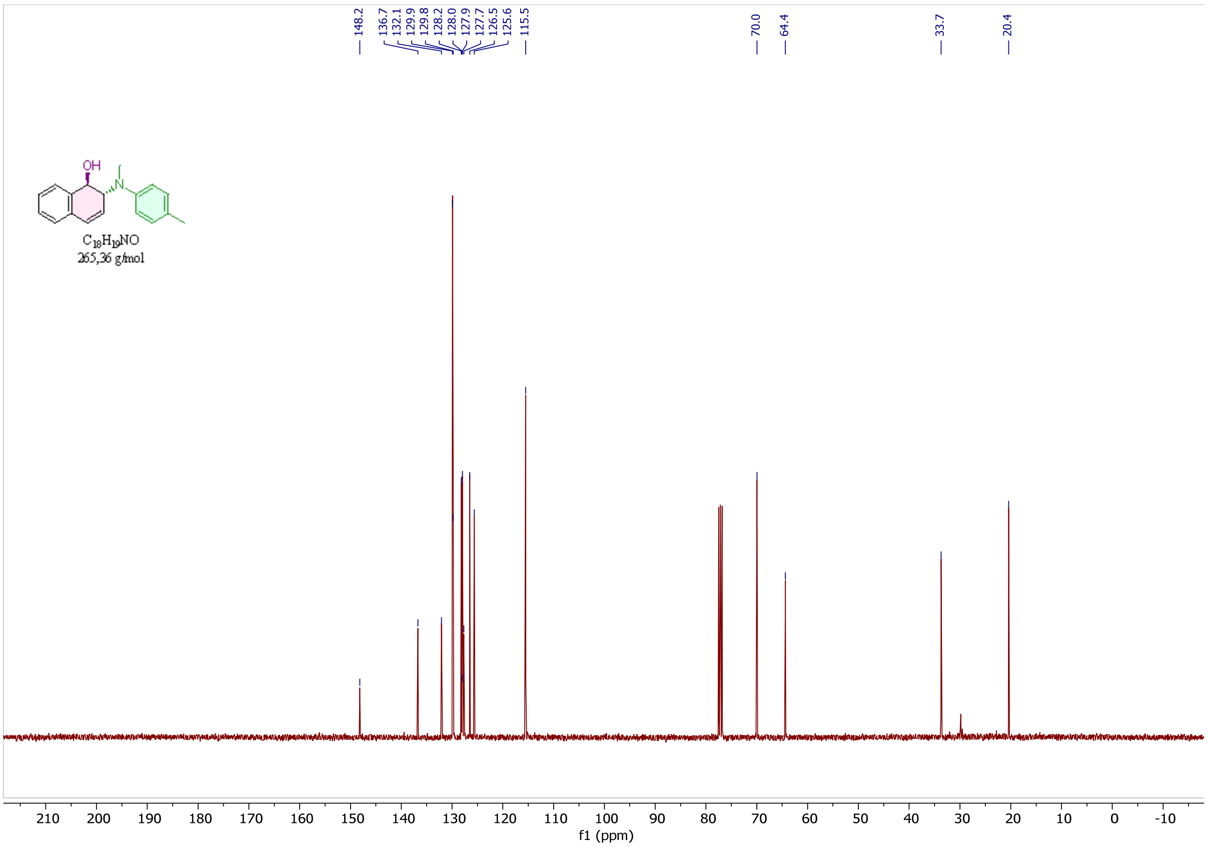

^{1}H NMR (400 MHz, $CDCl_3$) and ^{13}C{^{1}H} NMR (101 MHz, $CDCl_3$) Analysis of Compound **7ca**

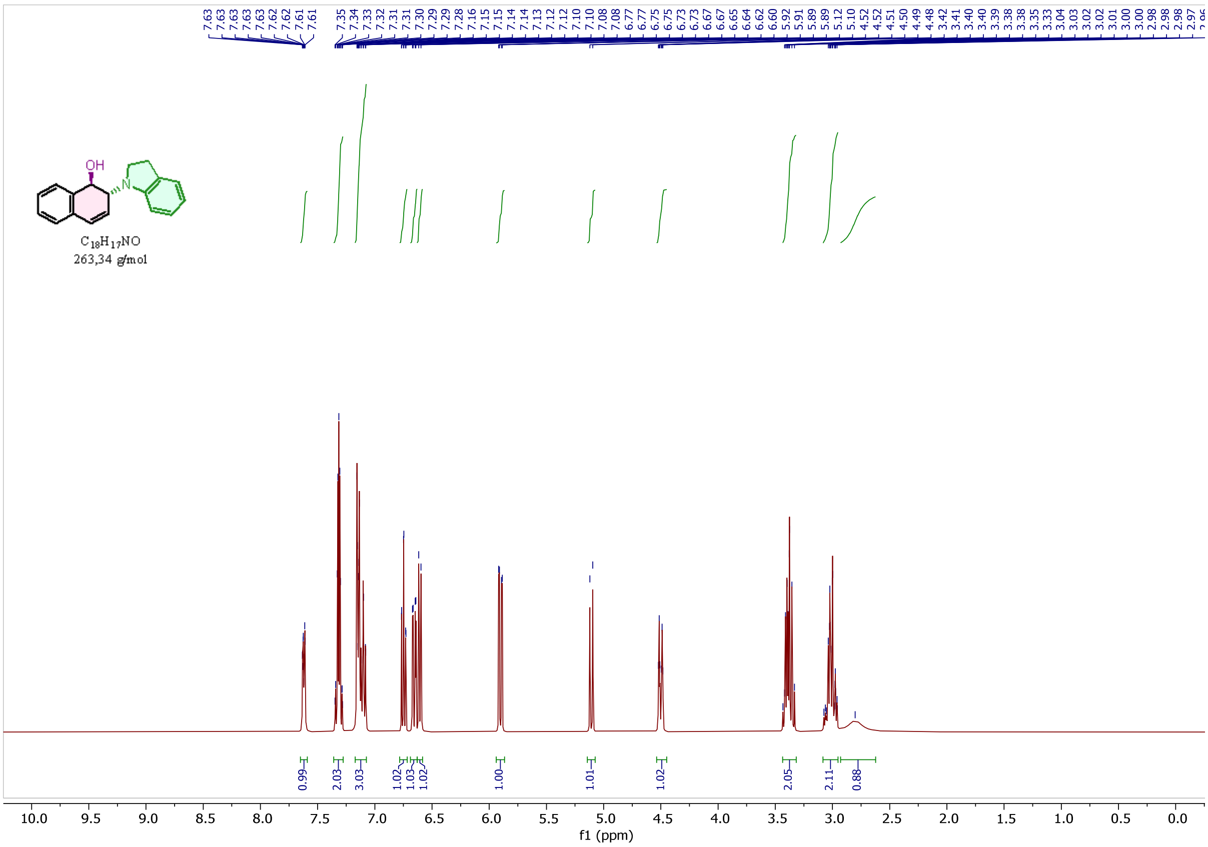

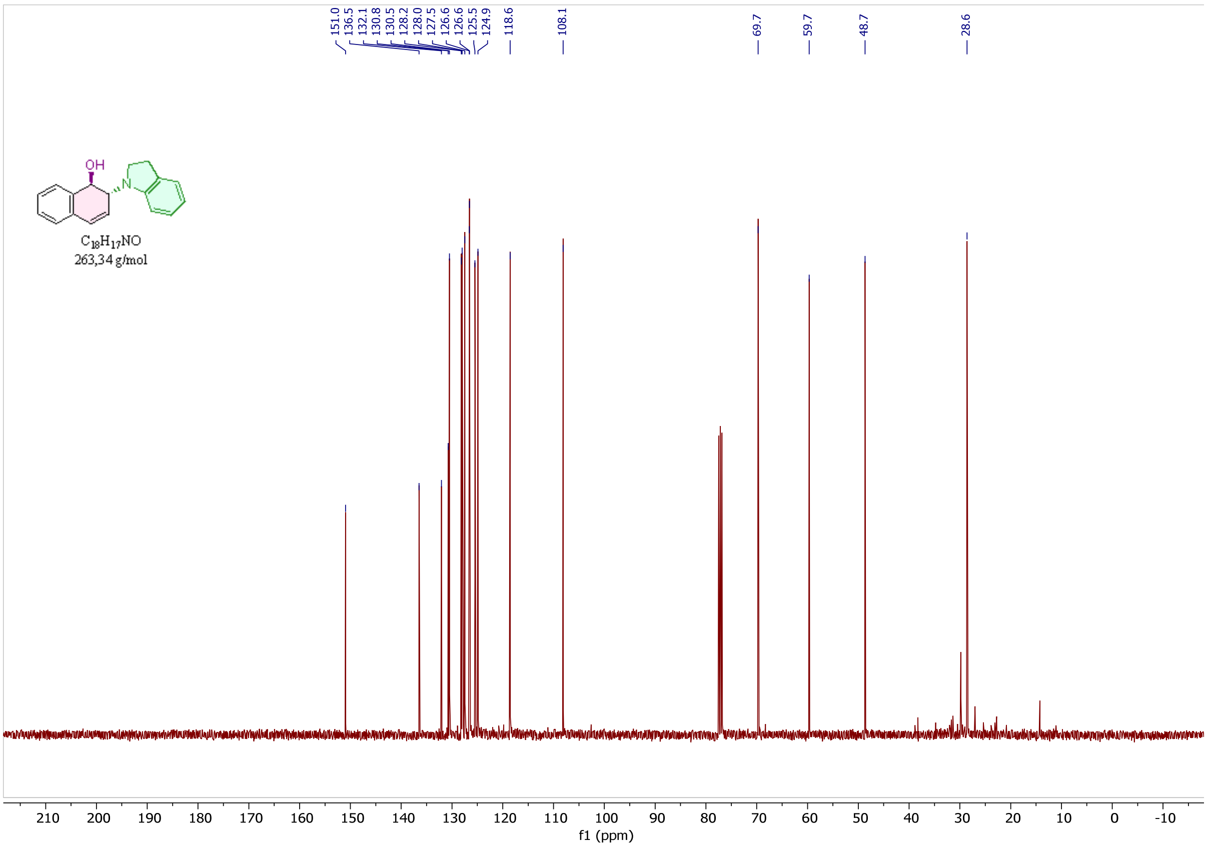

^{1}H NMR (400 MHz, $CDCl_3$) and ^{13}C{^{1}H} NMR (101 MHz, $CDCl_3$) Analysis of Compound **7cb**

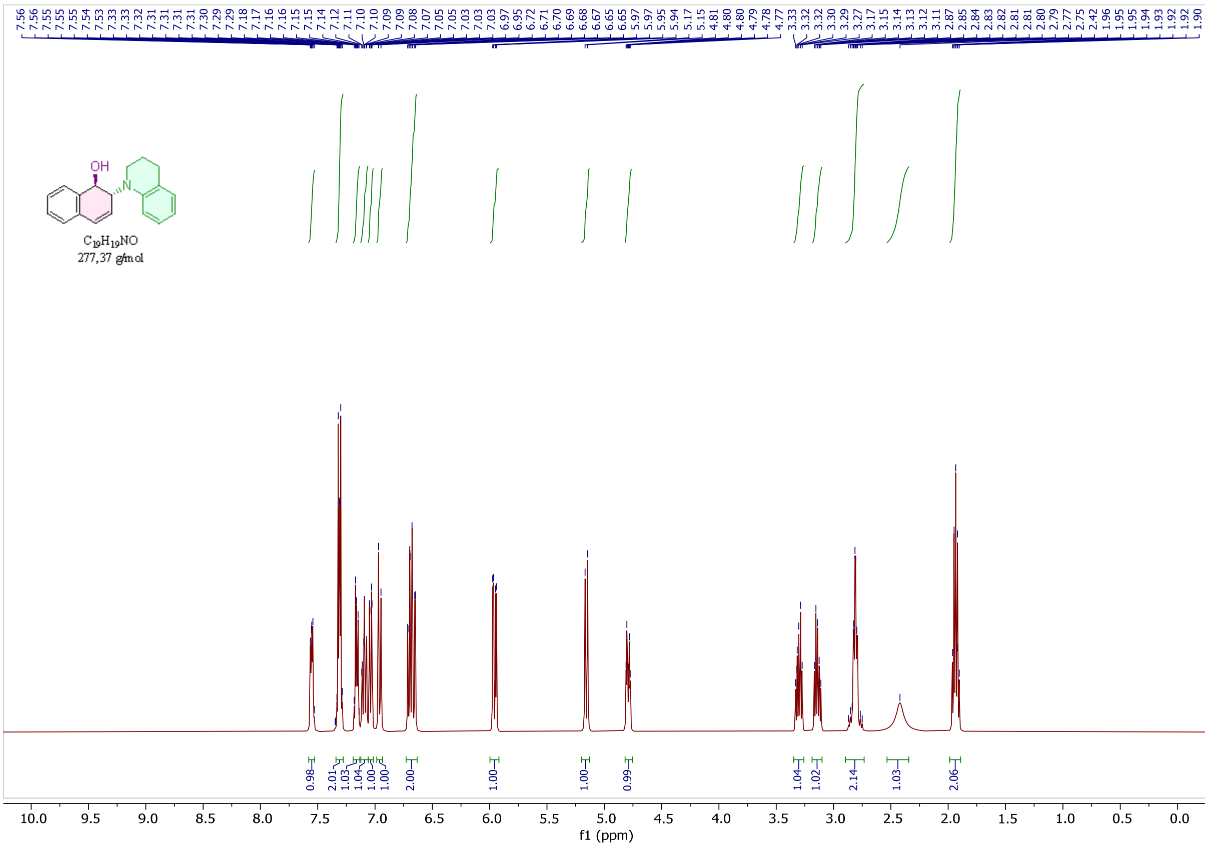

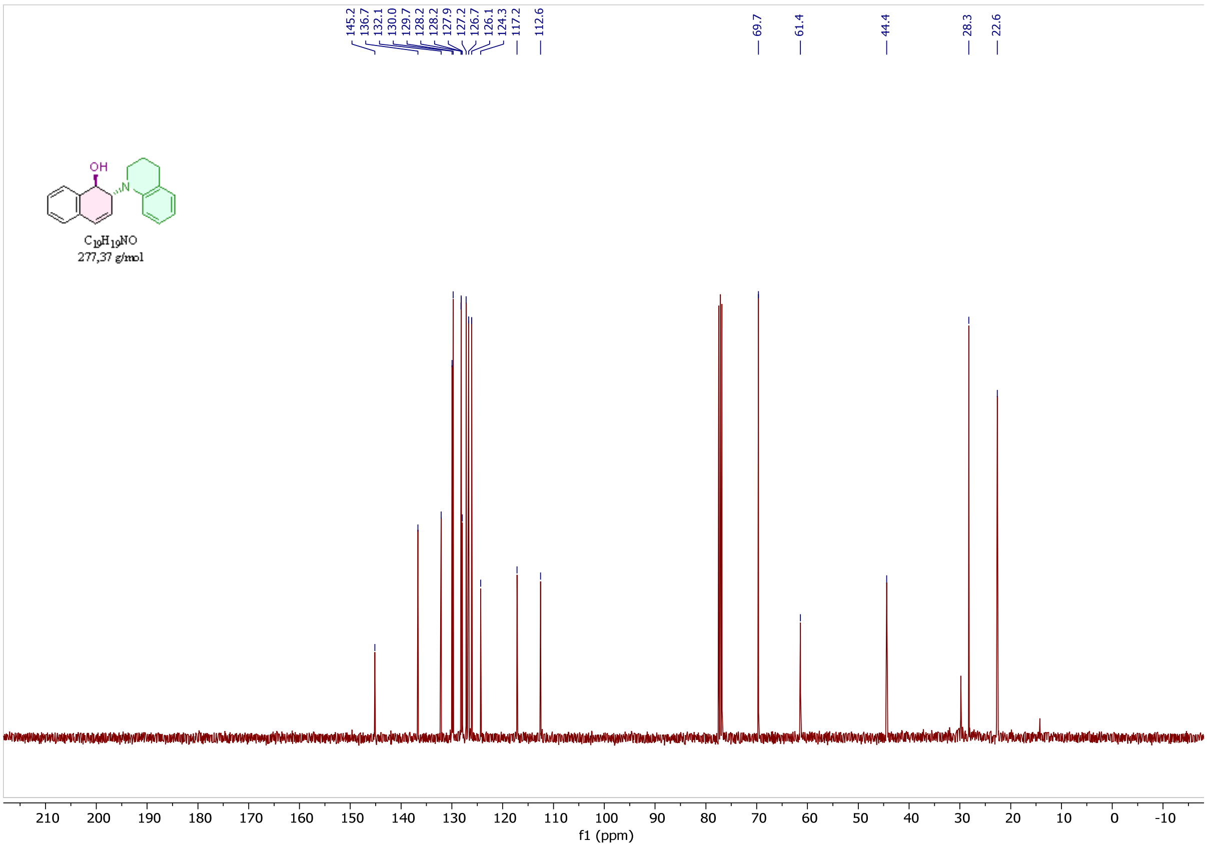

^{1}H NMR (400 MHz, $CDCl_3$) and $^{13}C\{^{1}H\}$ NMR (101 MHz, $CDCl_3$) Analysis of Compound **7cc**

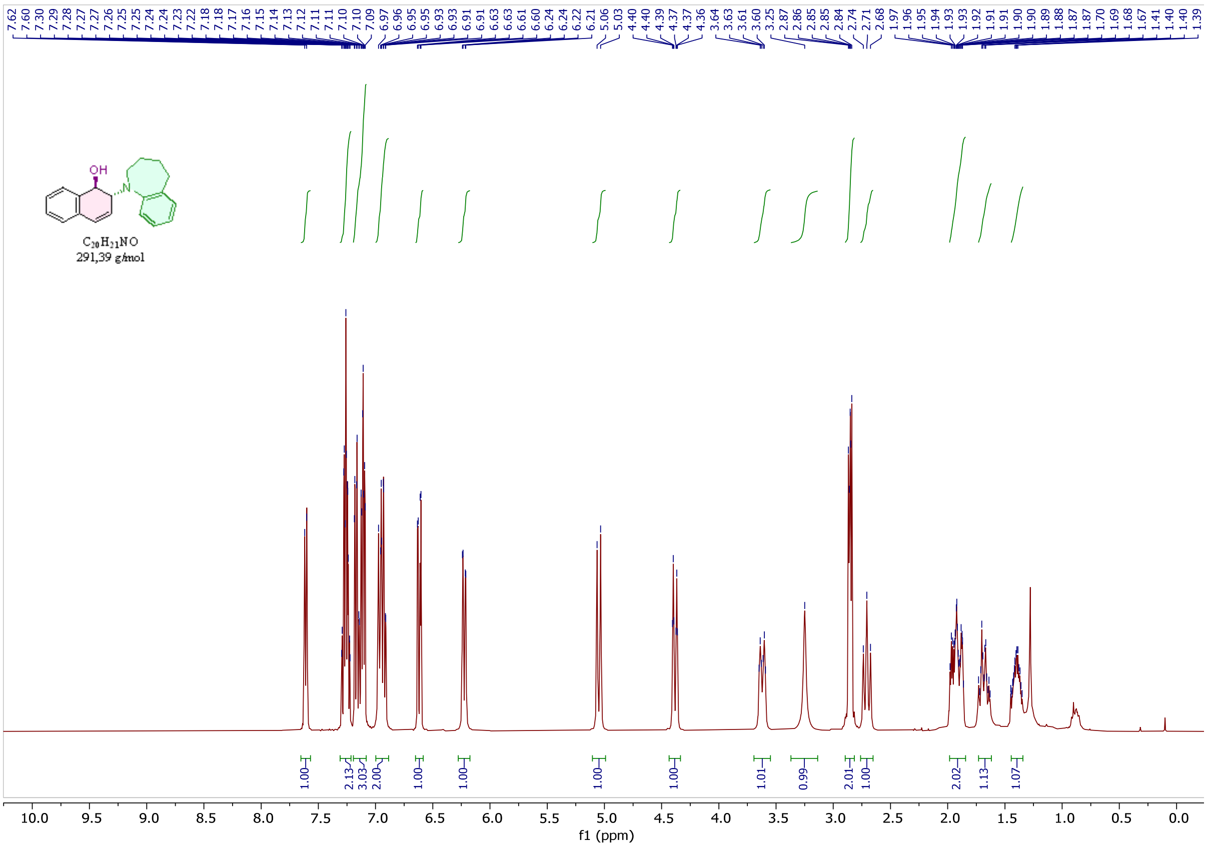

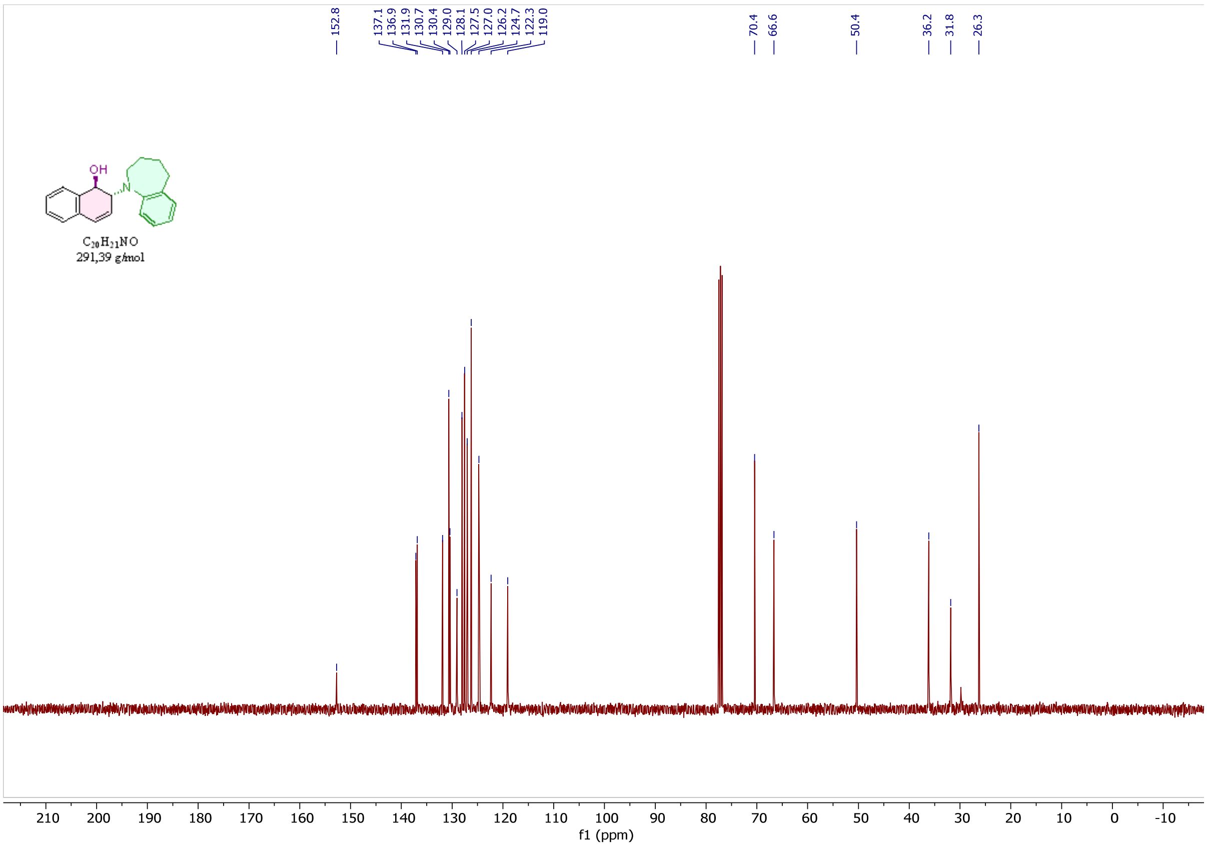

^1H NMR (400 MHz, $CDCl_3$) and $^{13}C\{^1H\}$ NMR (101 MHz, $CDCl_3$) Analysis of Compound **7da**

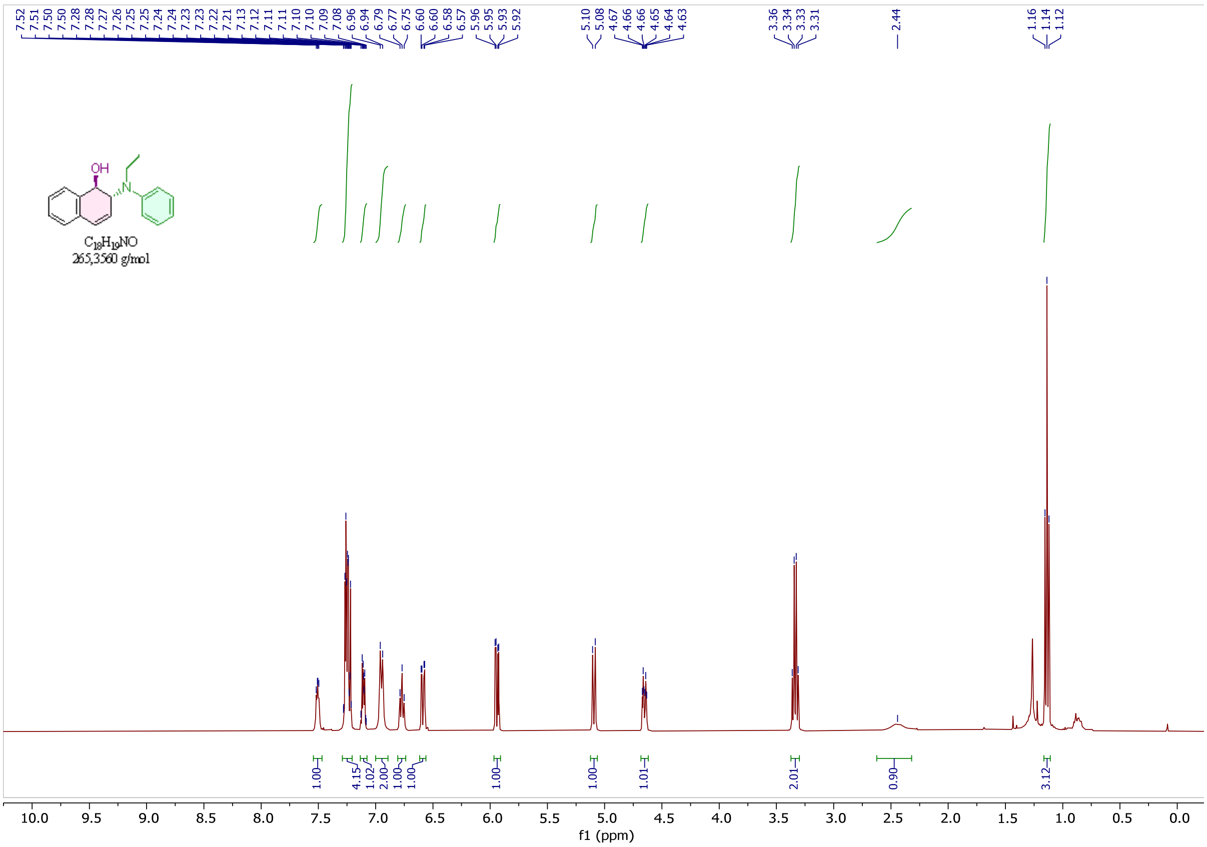

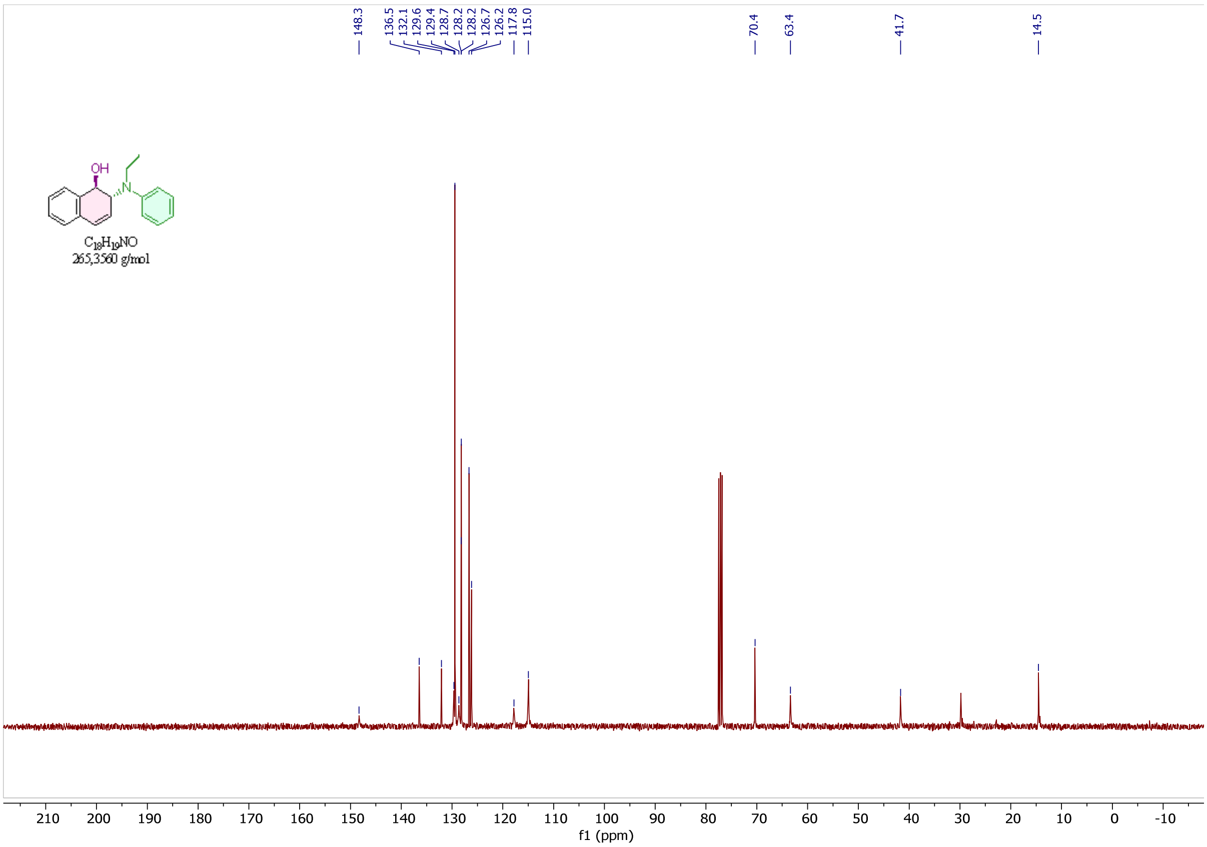

^1H NMR (400 MHz, $CDCl_3$) and $^{13}C\{^1H\}$ NMR (101 MHz, $CDCl_3$) Analysis of Compound **7db**

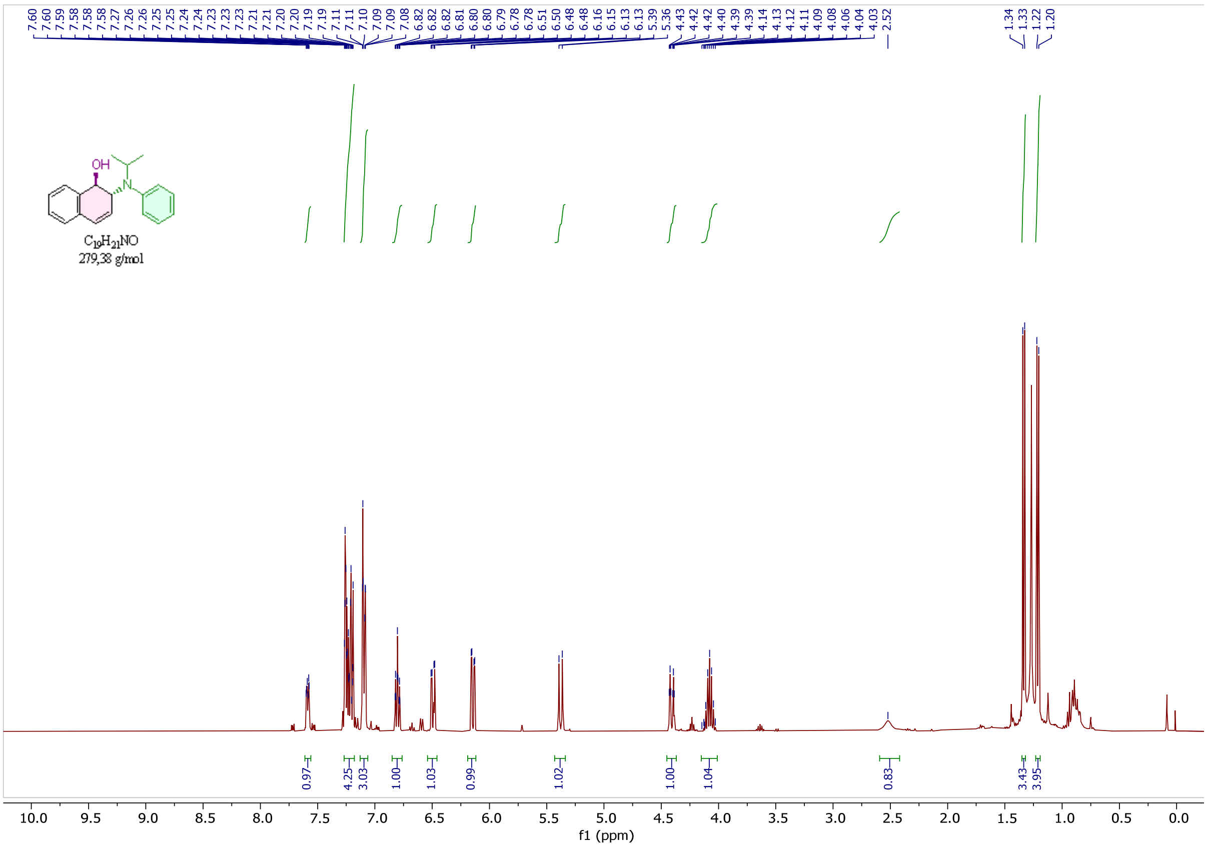

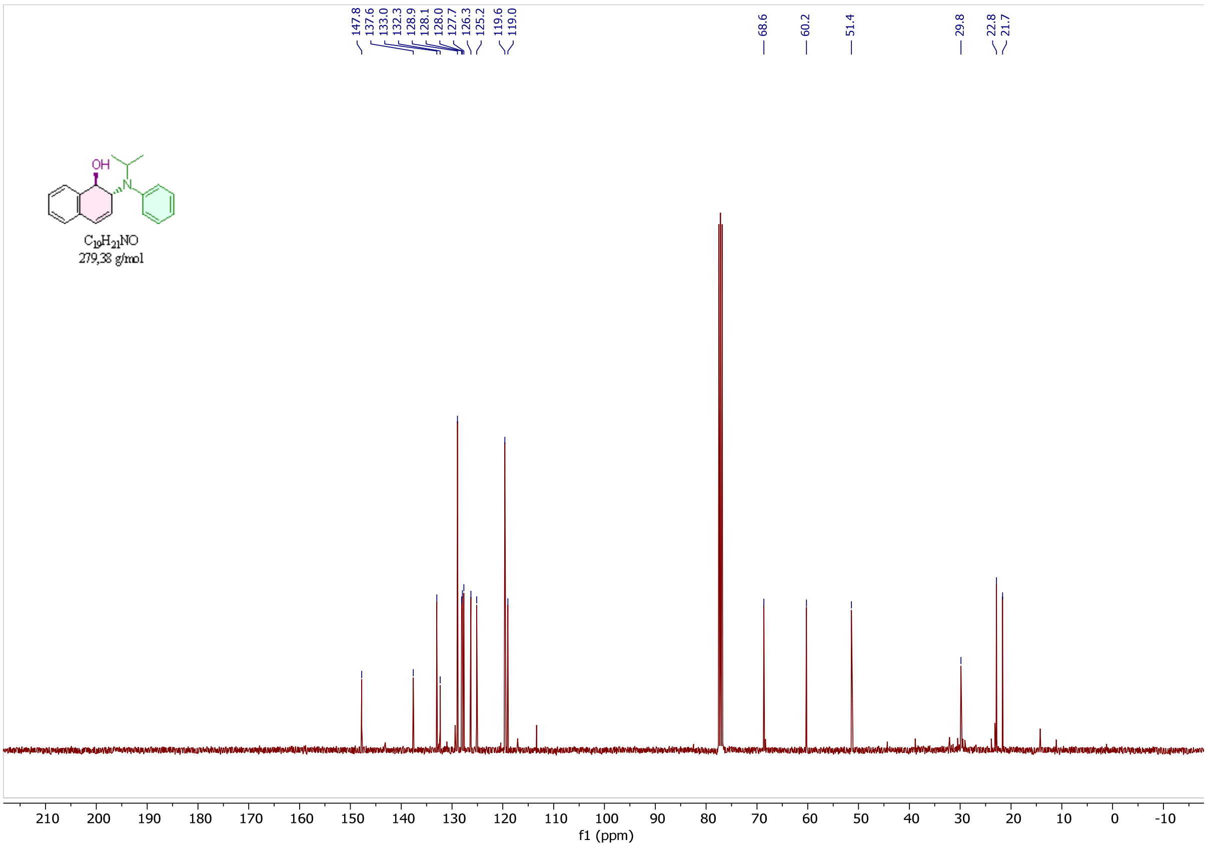

^1H NMR (400 MHz, $CDCl_3$) and $^{13}C\{^1H\}$ NMR (101 MHz, $CDCl_3$) Analysis of Compound **7dc**

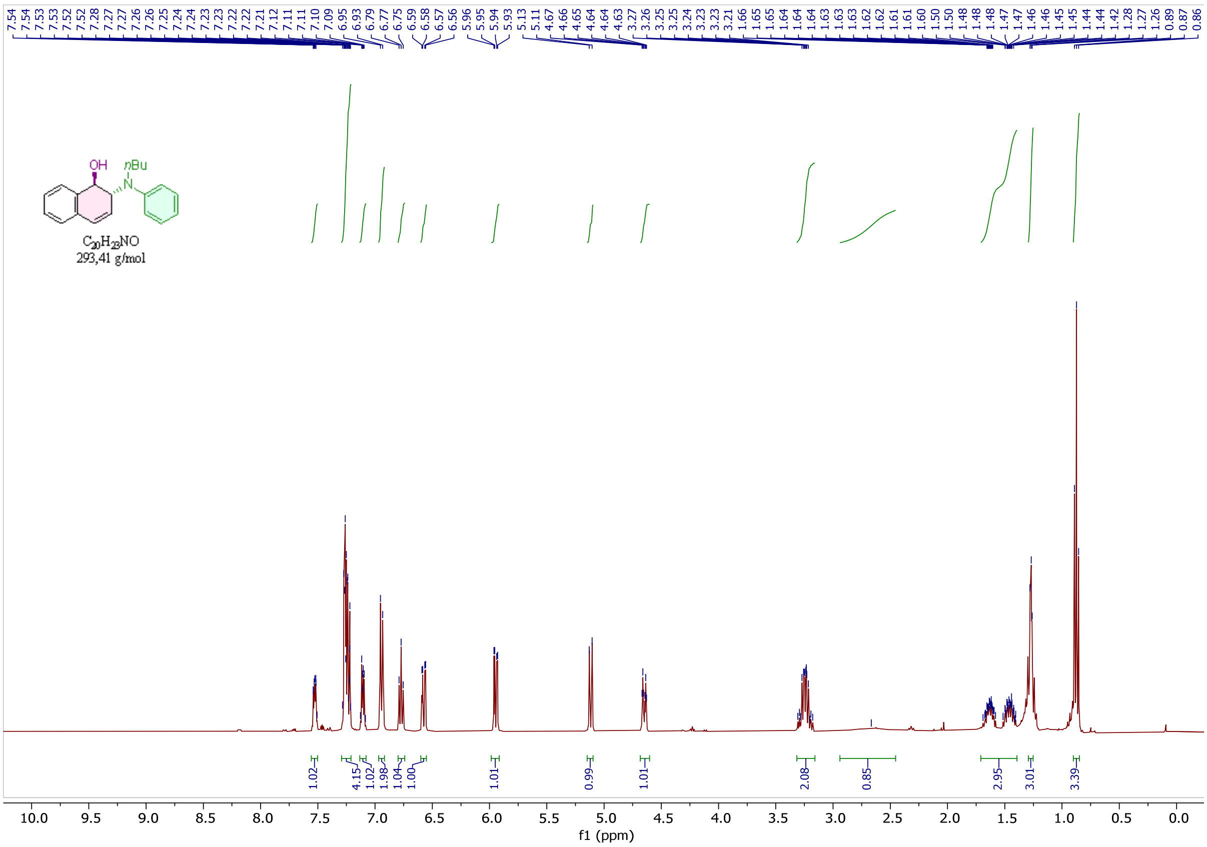

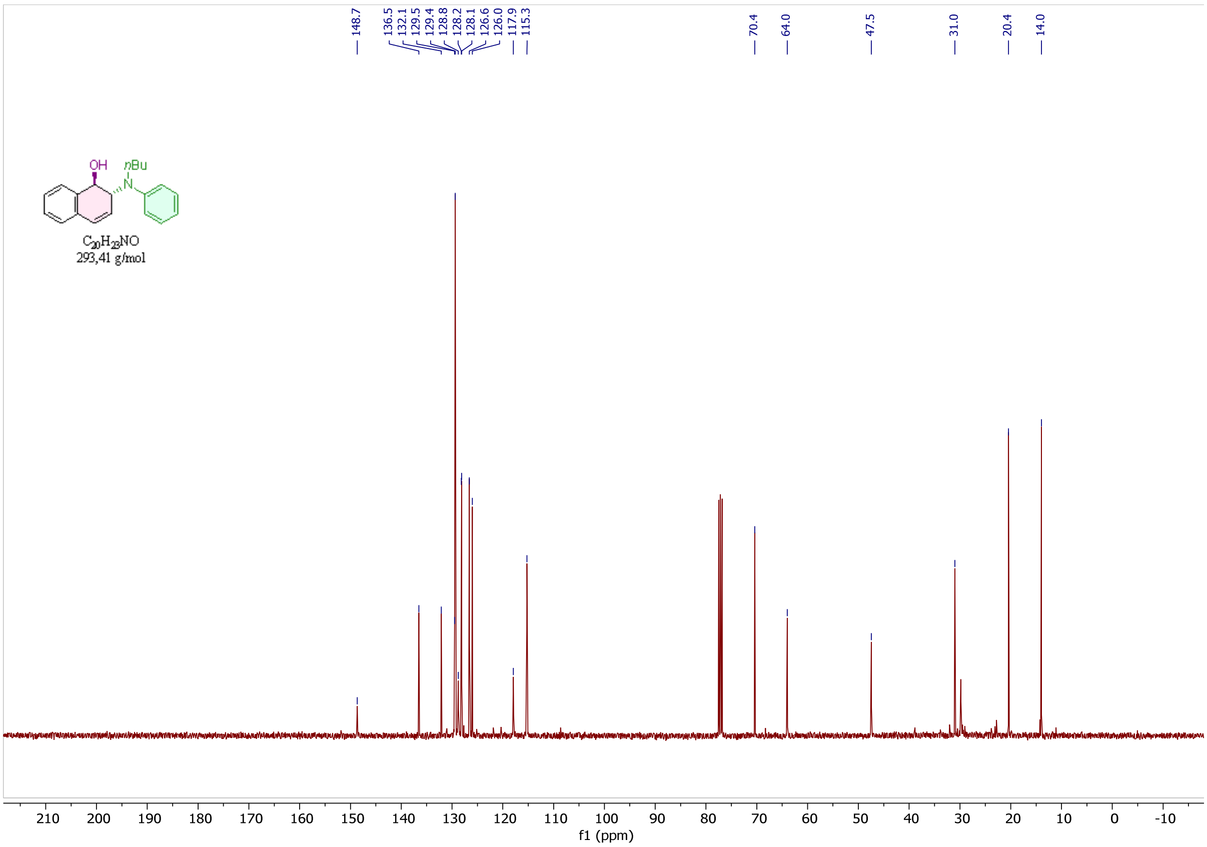

^{1}H NMR (400 MHz, $CDCl_3$) and $^{13}C\{^1H\}$ NMR (101 MHz, $CDCl_3$) Analysis of Compound **7e**

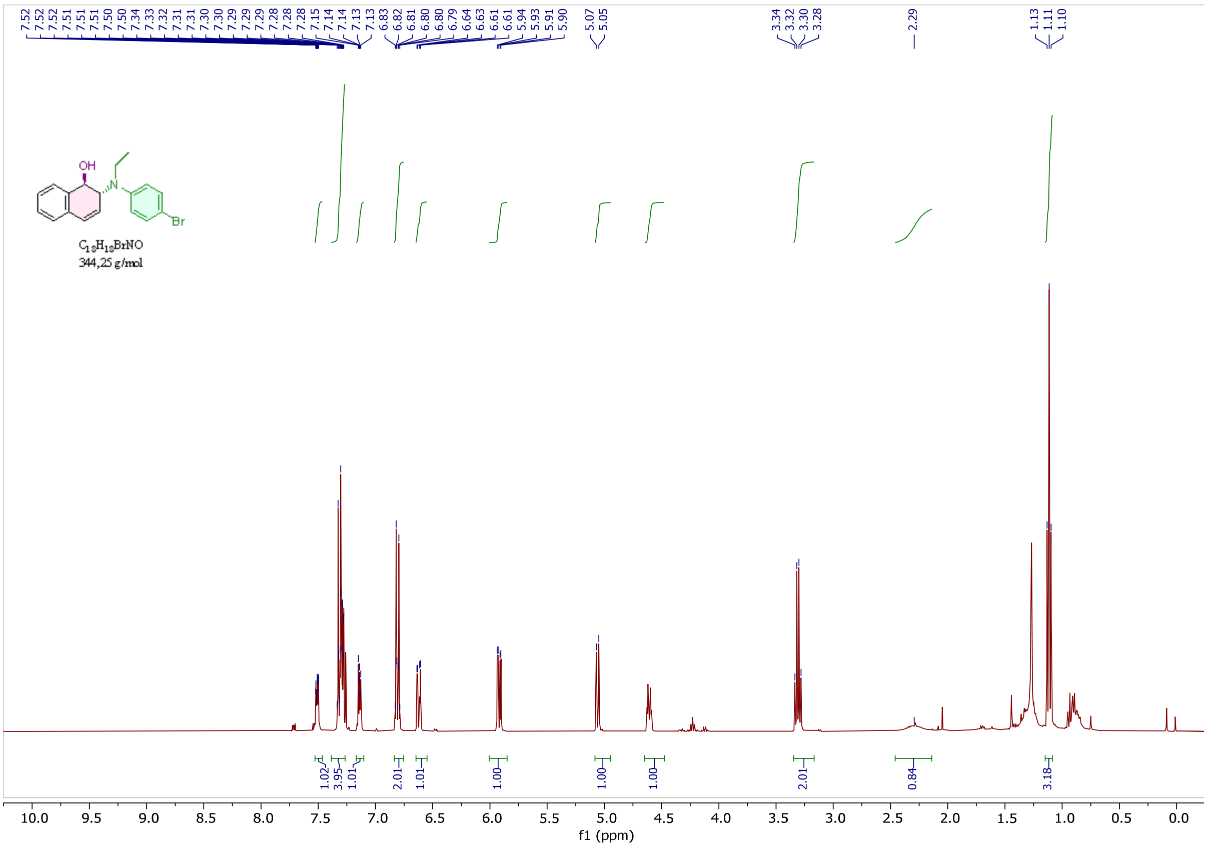

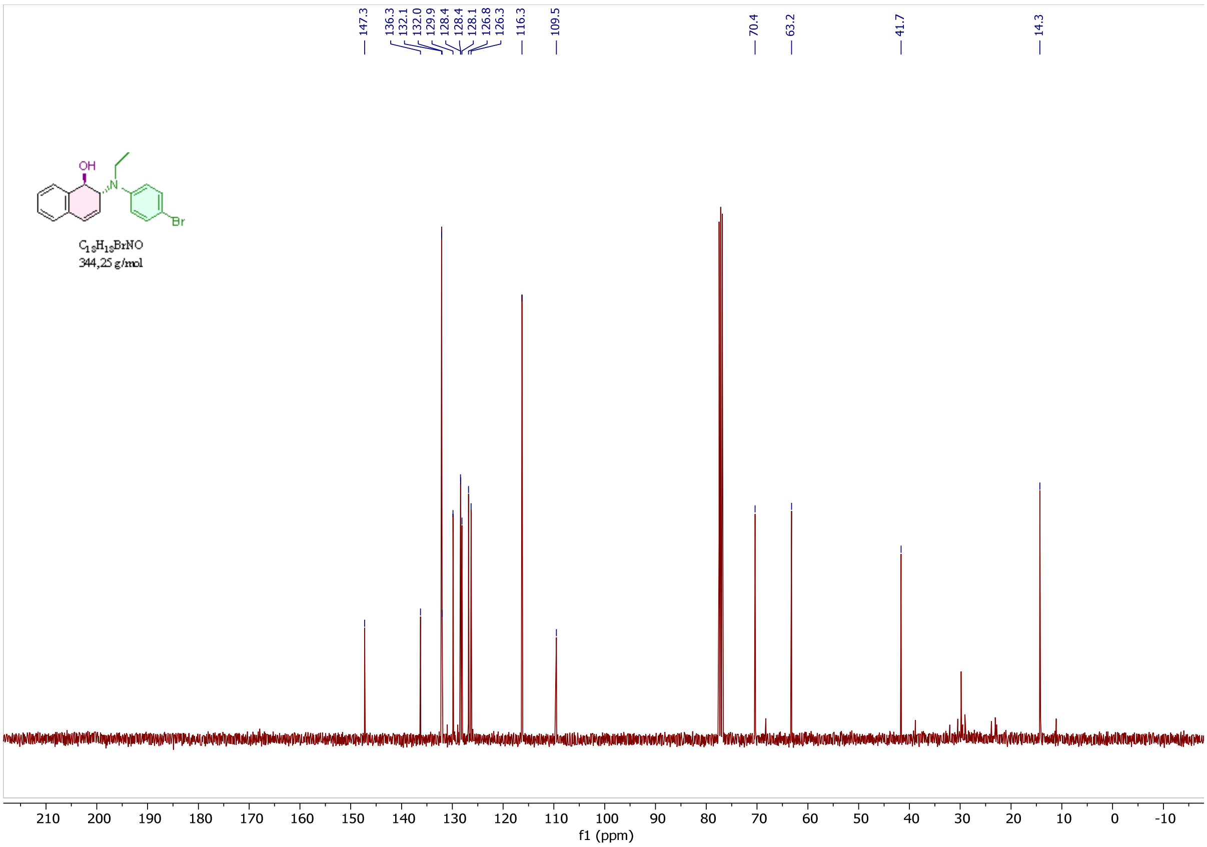

^{1}H NMR (400 MHz, $CDCl_3$) and $^{13}C\{^{1}H\}$ NMR (101 MHz, $CDCl_3$) Analysis of Compound **7f**

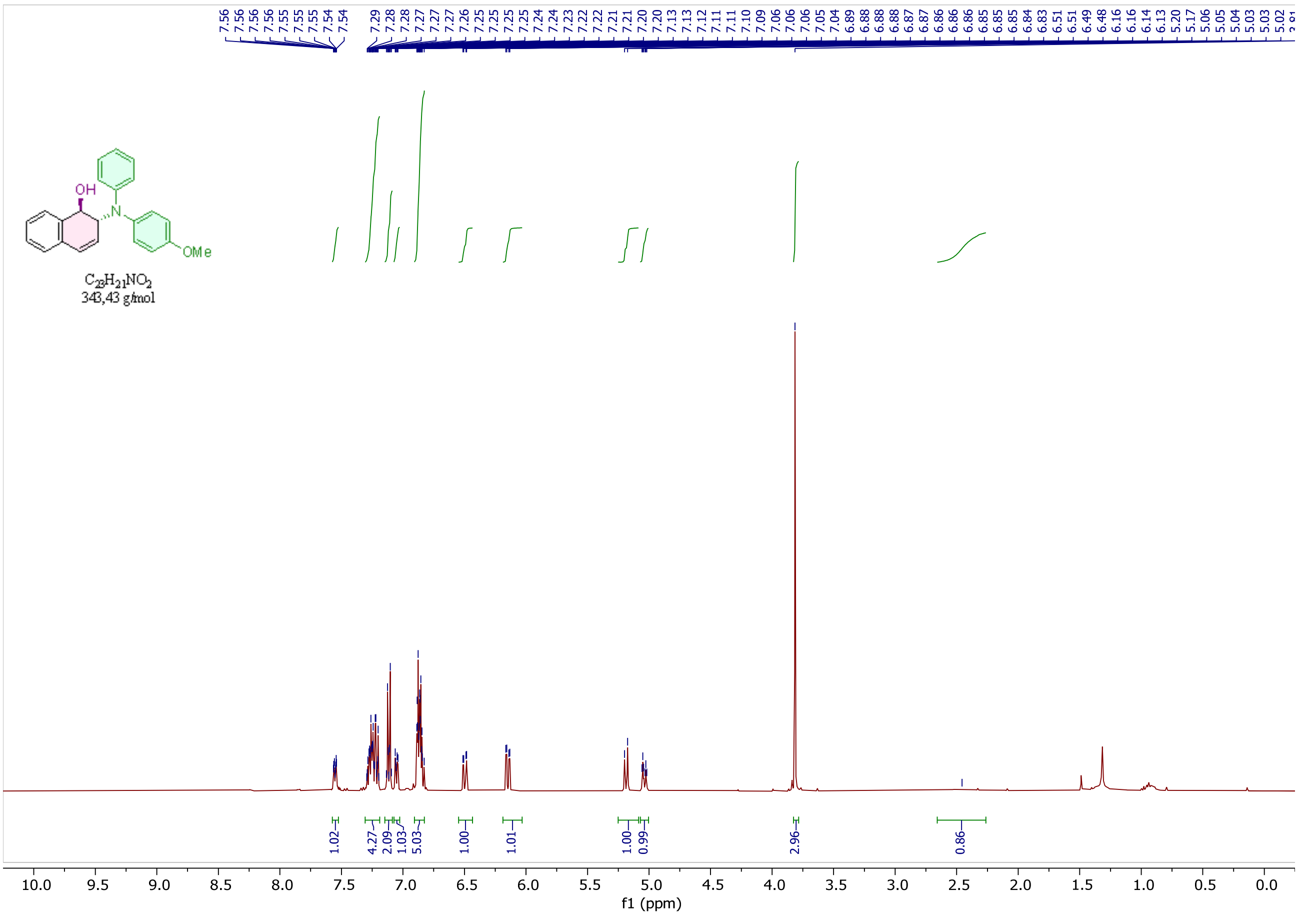

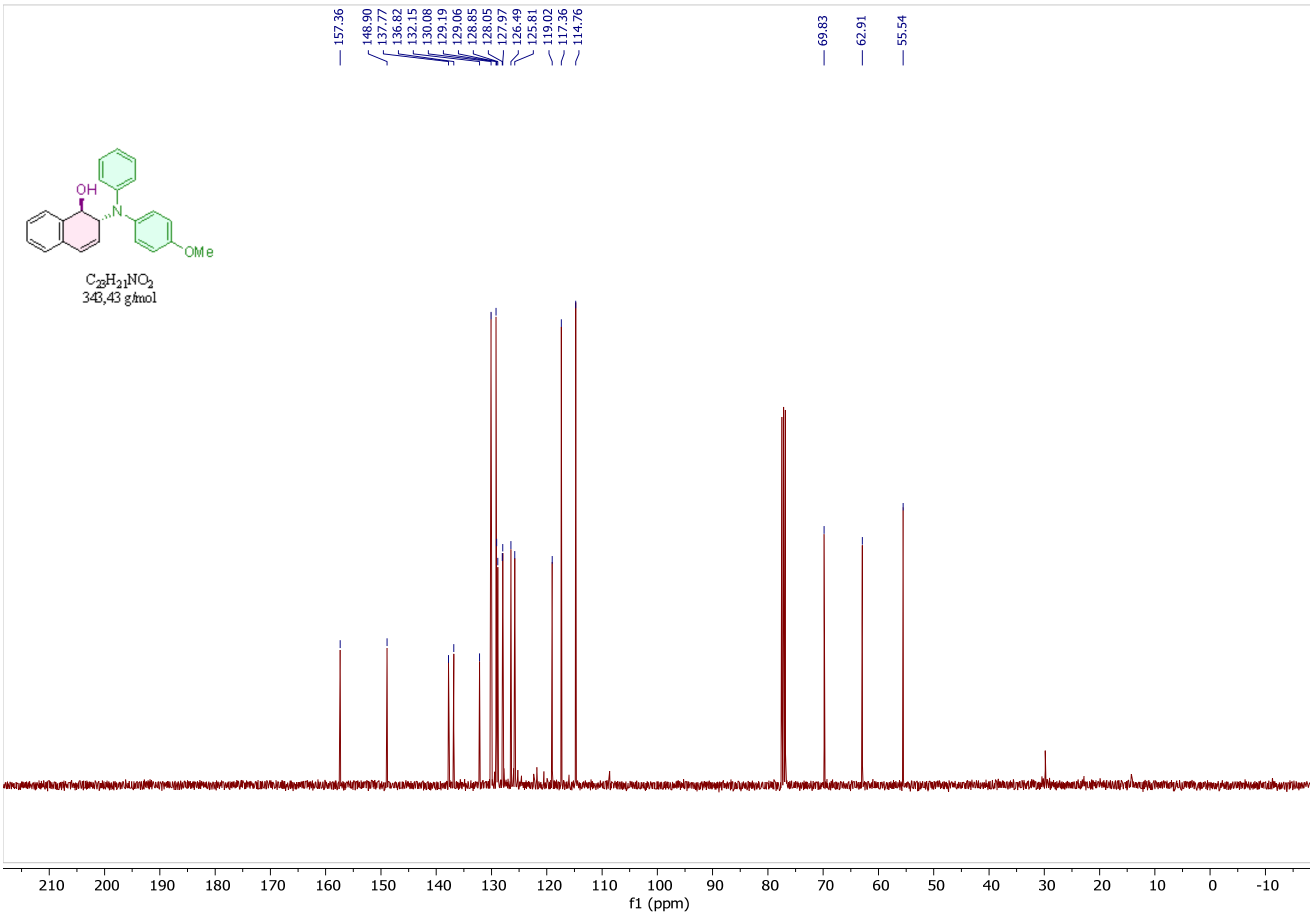

^{1}H NMR (400 MHz, $CDCl_3$) and ^{13}C{^{1}H} NMR (101 MHz, $CDCl_3$) Analysis of Compound **7ga**

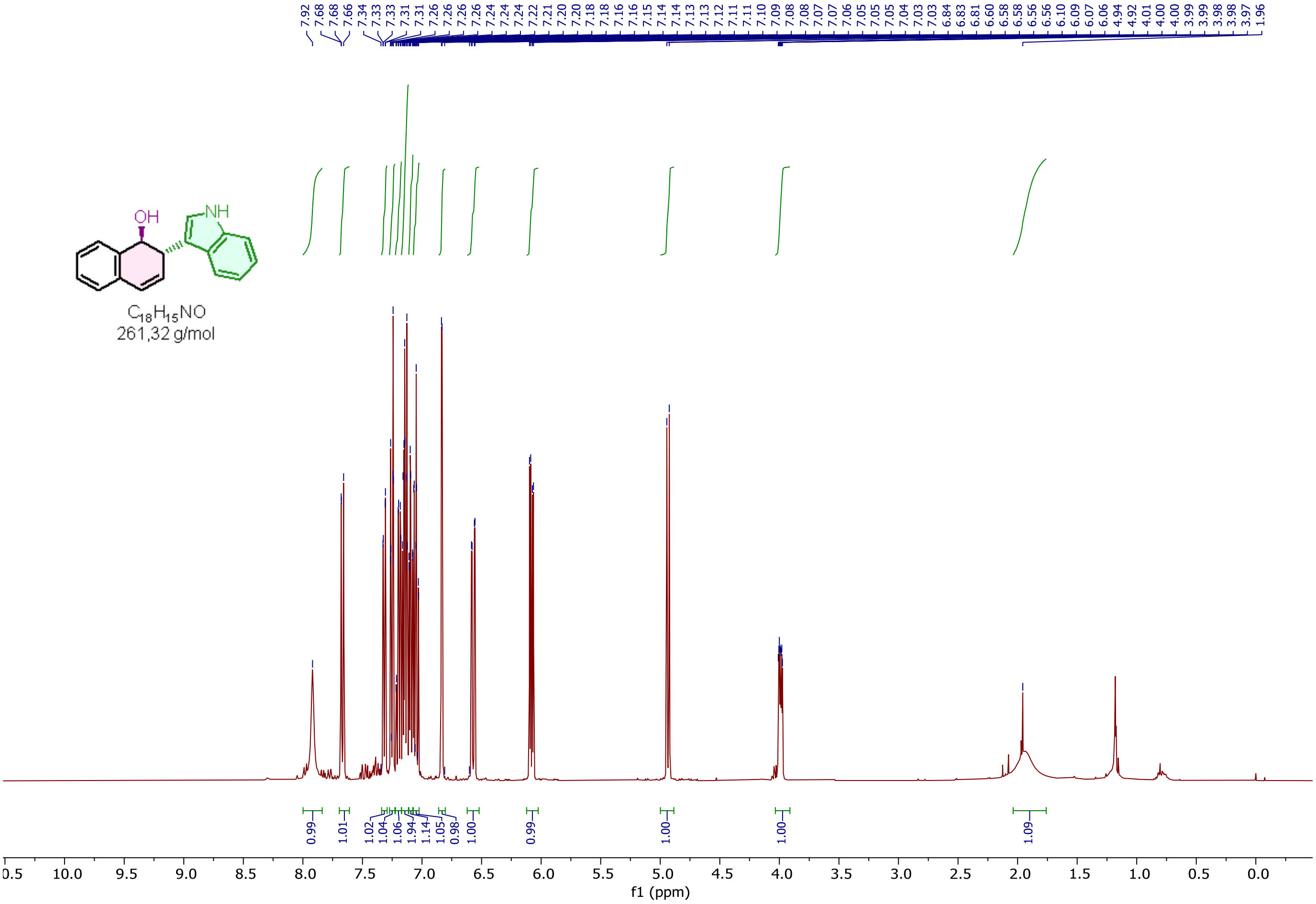

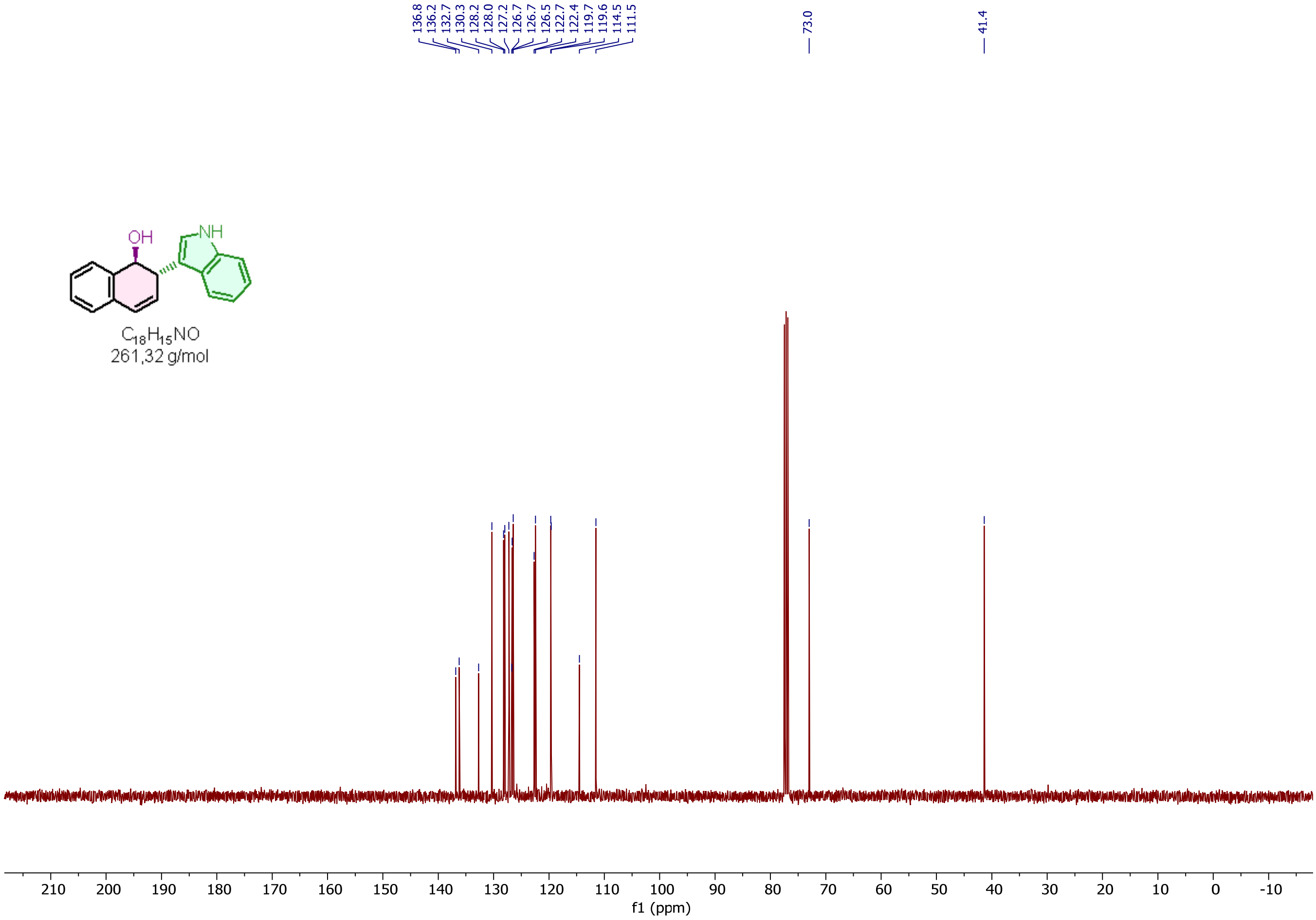

^{1}H NMR (400 MHz, $CDCl_3$) and $^{13}C\{^{1}H\}$ NMR (101 MHz, $CDCl_3$) Analysis of Compound **7gb**

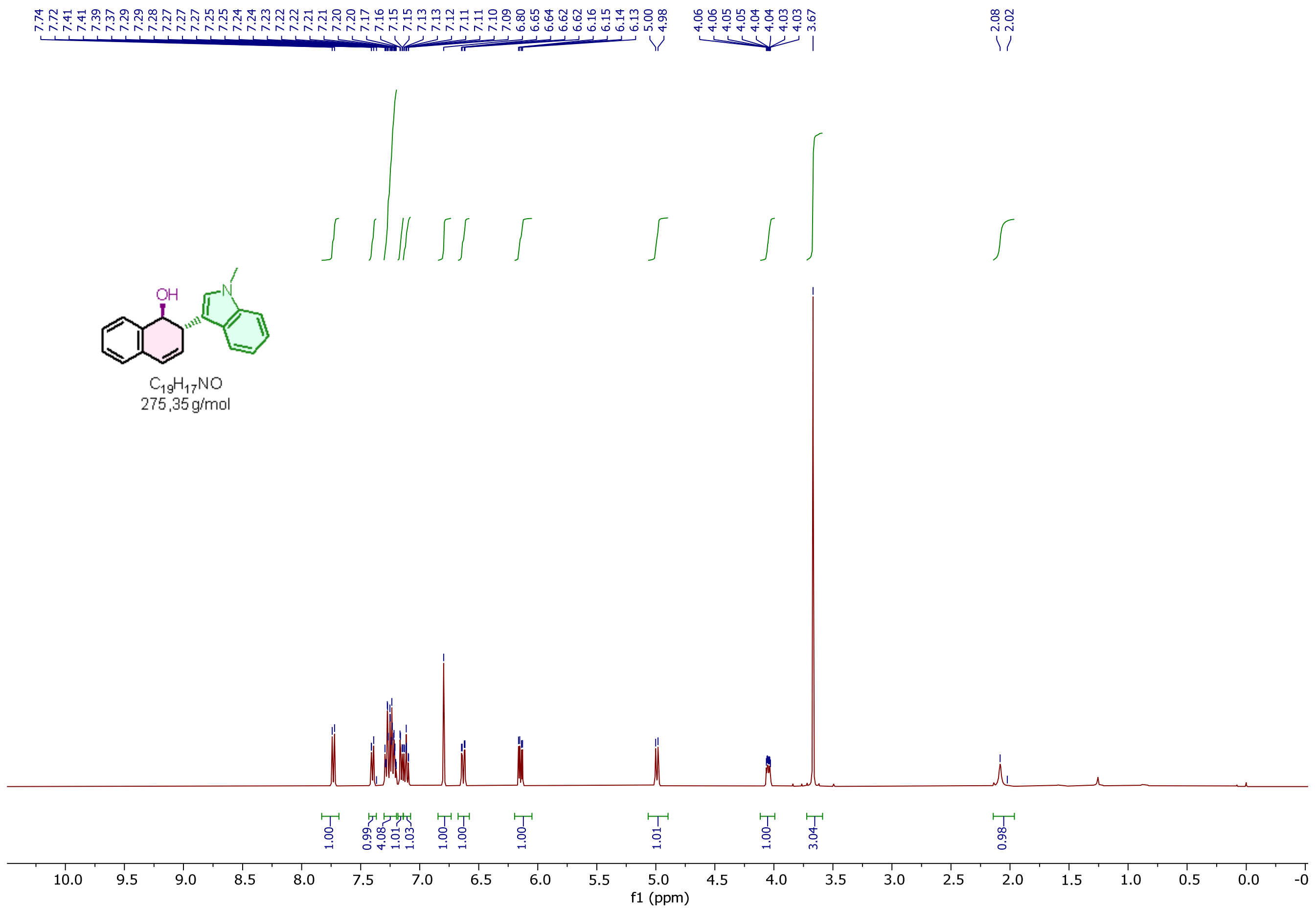

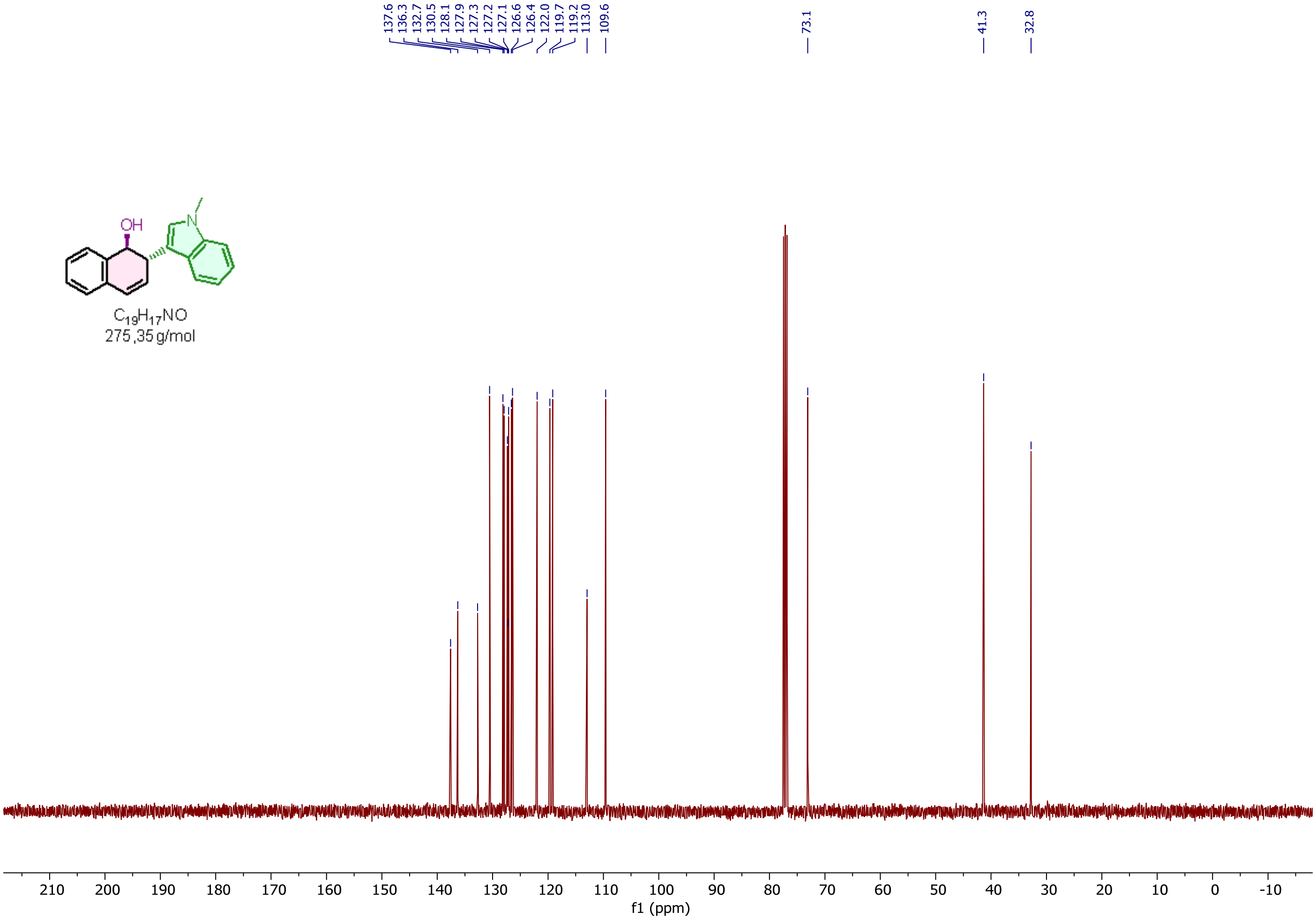

^{1}H NMR (400 MHz, $CDCl_3$) and ^{13}C{^{1}H} NMR (101 MHz, $CDCl_3$) Analysis of Compound **7ha**

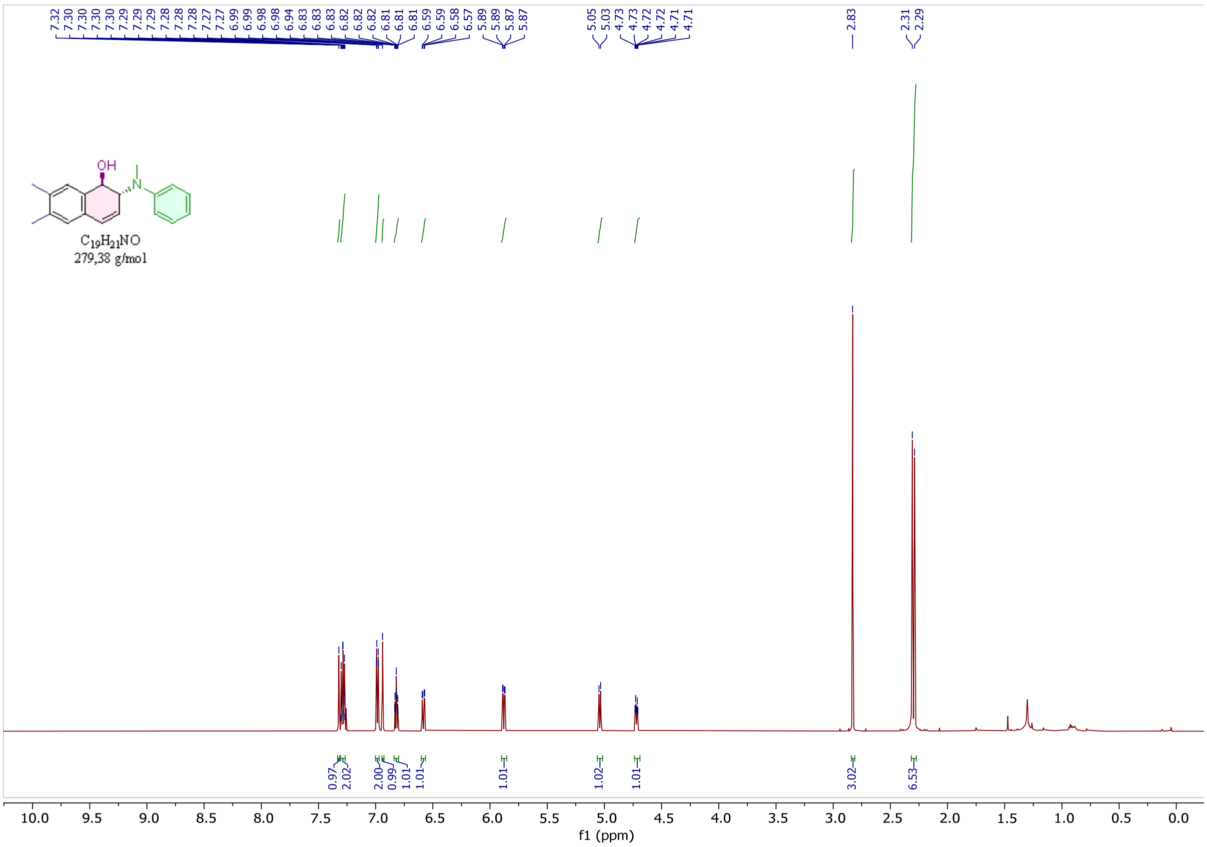

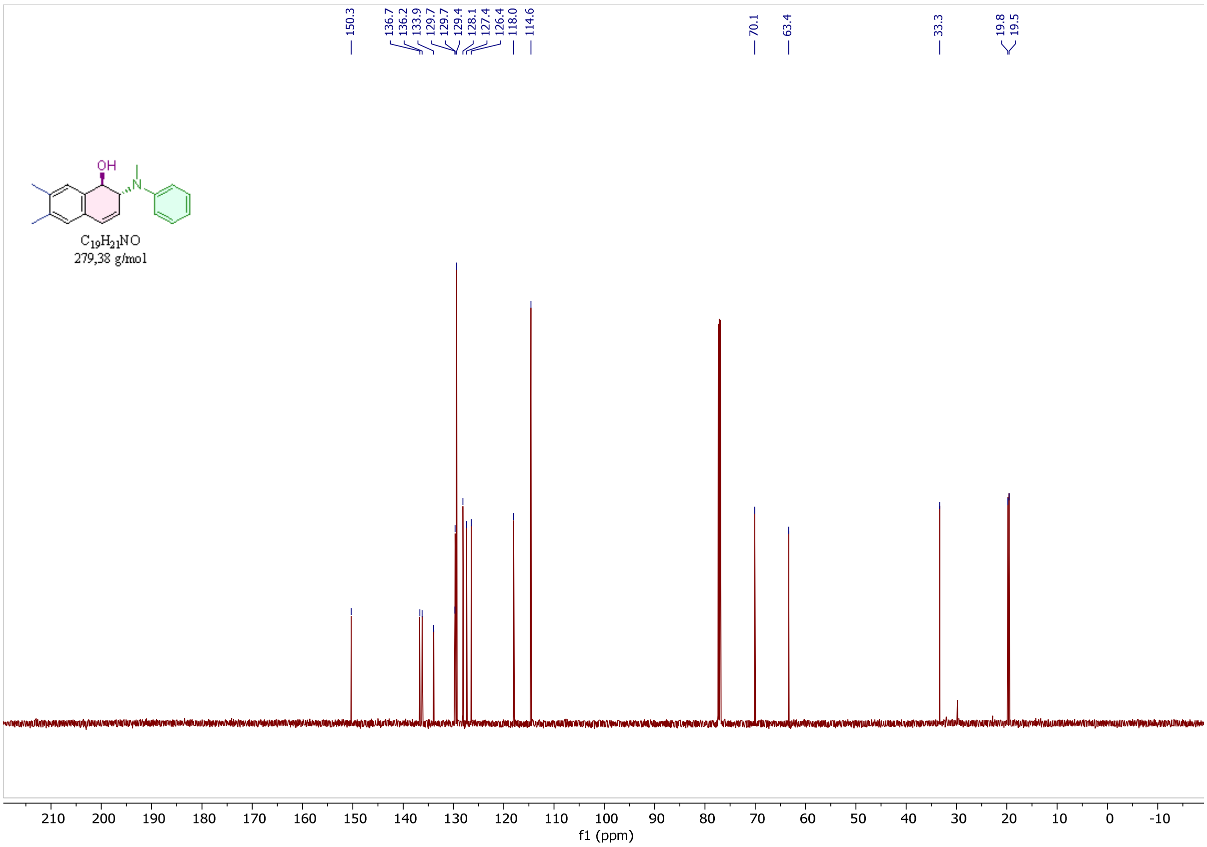

^1H NMR (400 MHz, $CDCl_3$) and $^{13}C\{^1H\}$ NMR (101 MHz, $CDCl_3$) Analysis of Compound **7hb**

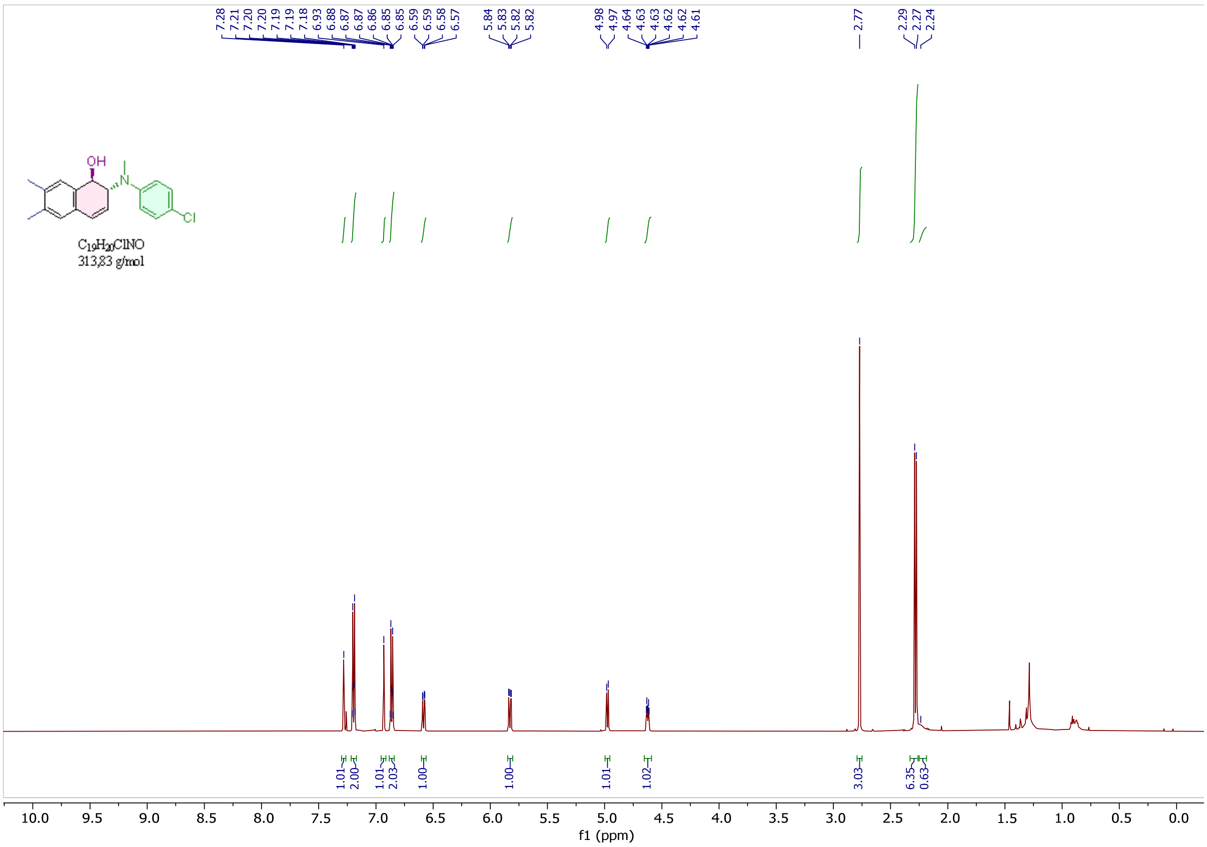

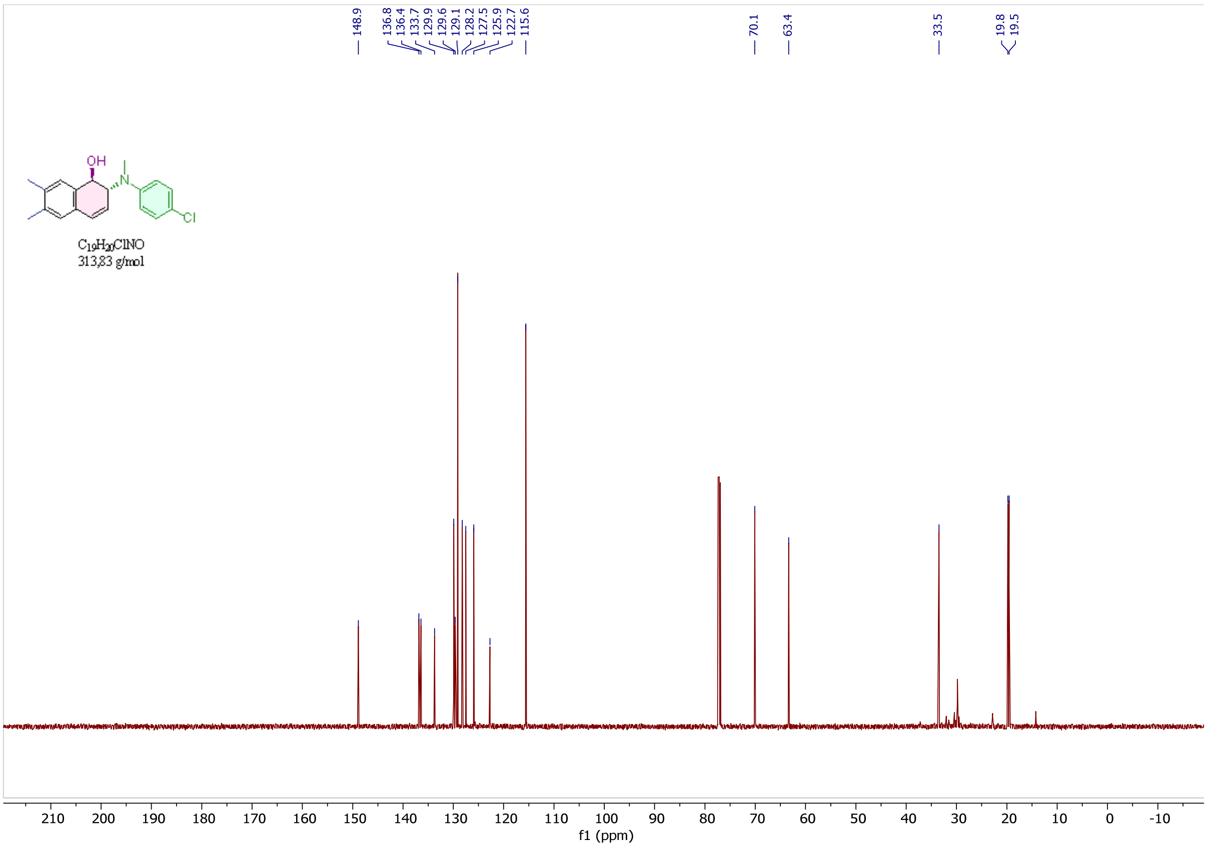

^{1}H NMR (400 MHz, $CDCl_3$) and ^{13}C{^{1}H} NMR (101 MHz, $CDCl_3$) Analysis of Compound **7hc**

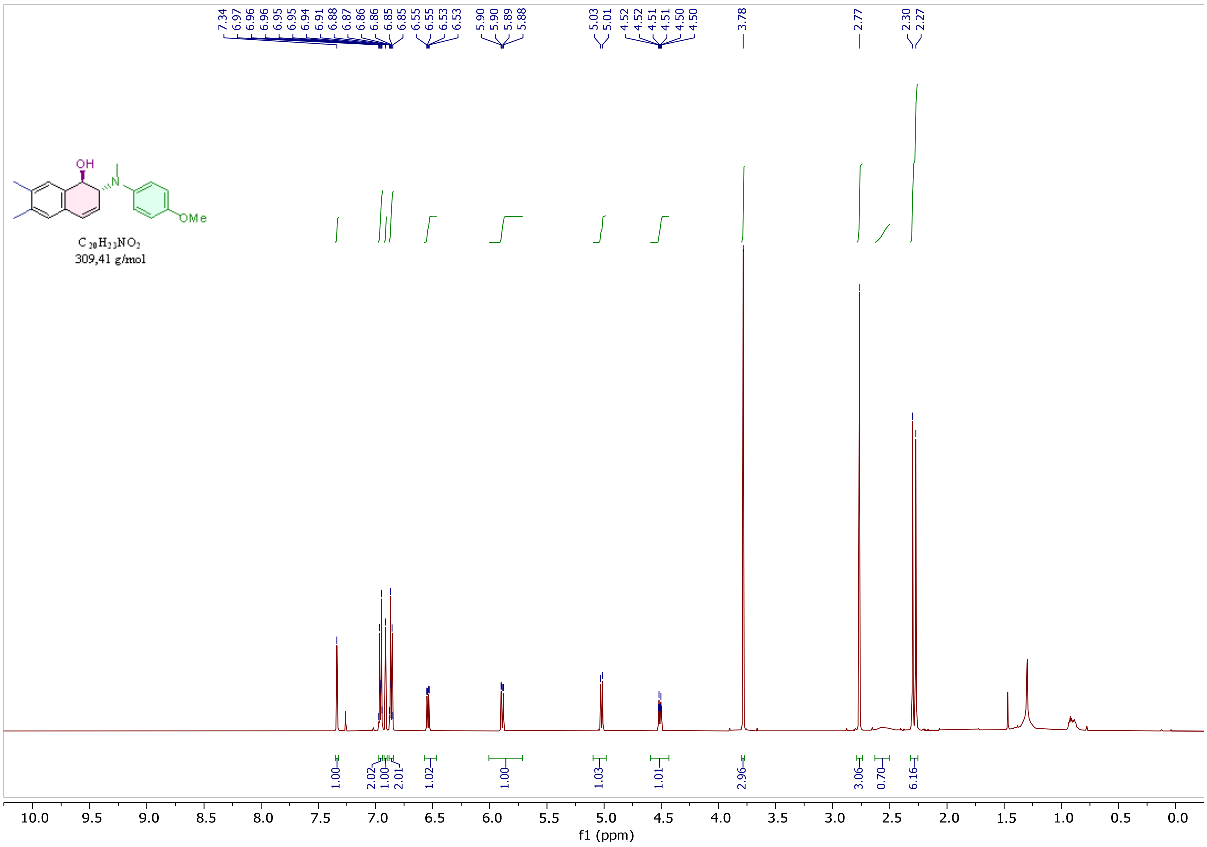

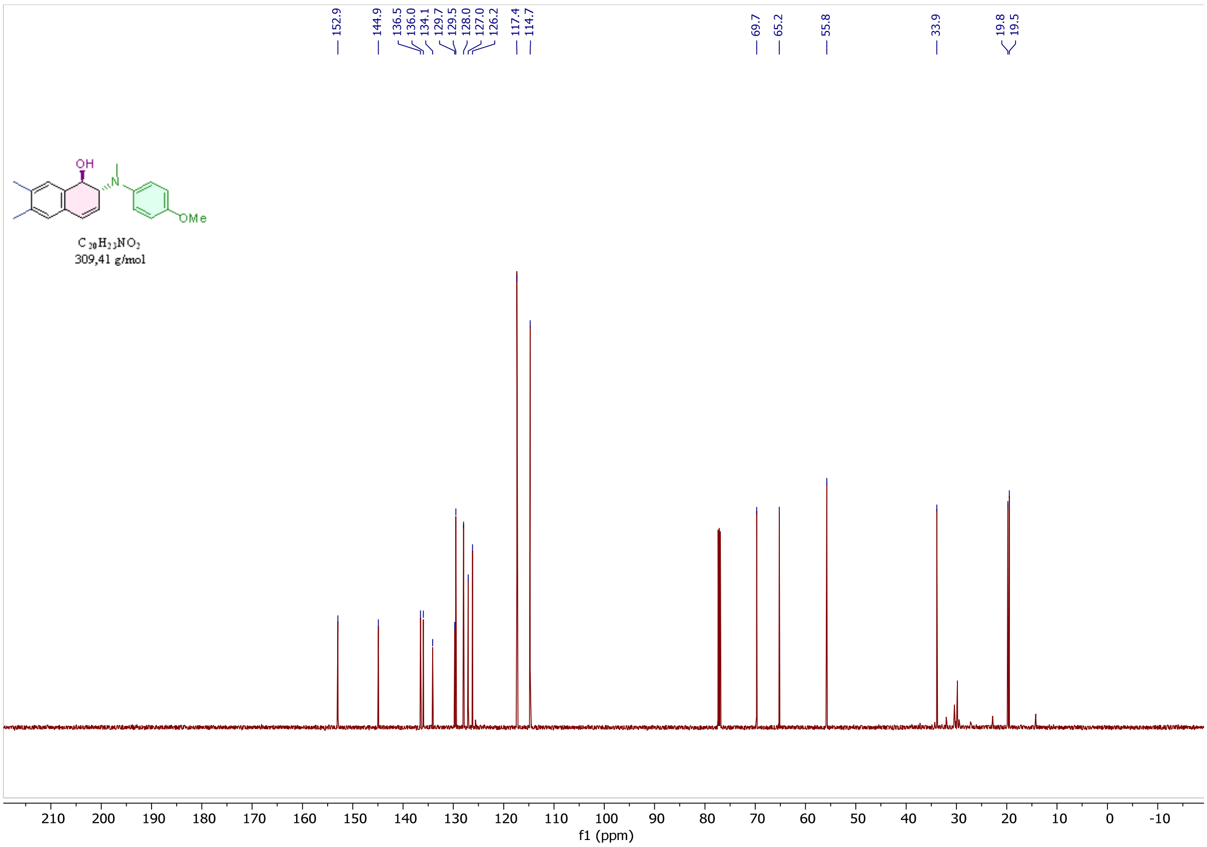

^{1}H NMR (400 MHz, $CDCl_3$) and ^{13}C{^{1}H} NMR (101 MHz, $CDCl_3$) Analysis of Compound **7ia**

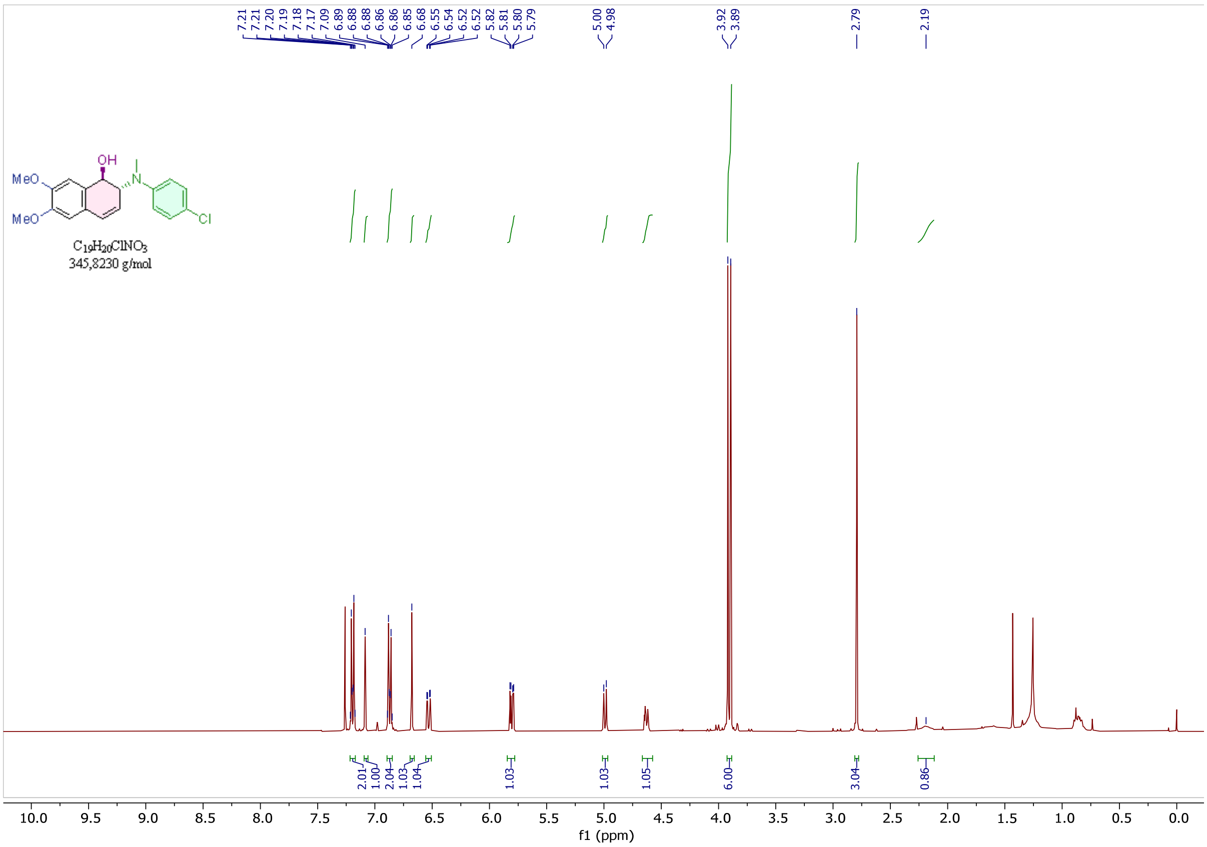

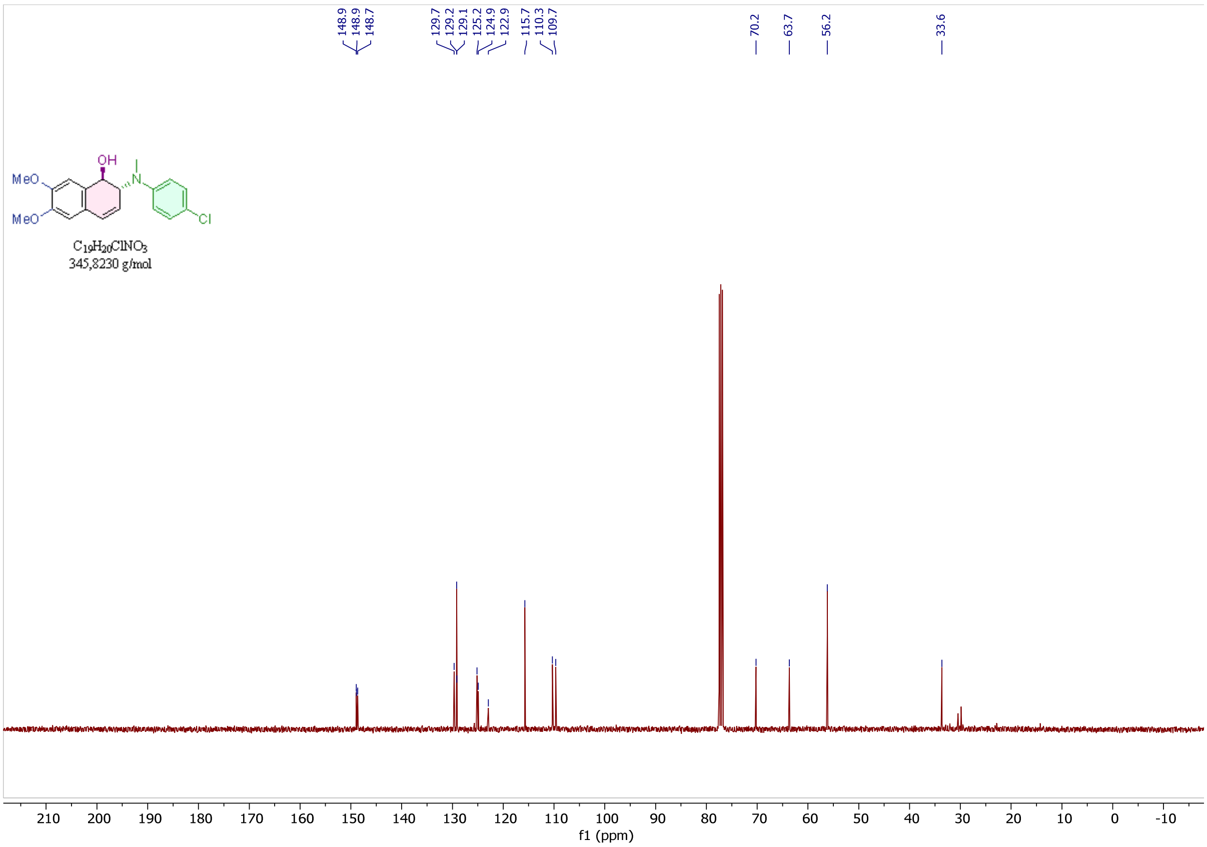

^{1}H NMR (400 MHz, $CDCl_3$) and ^{13}C{^{1}H} NMR (101 MHz, $CDCl_3$) Analysis of Compound **7ib**

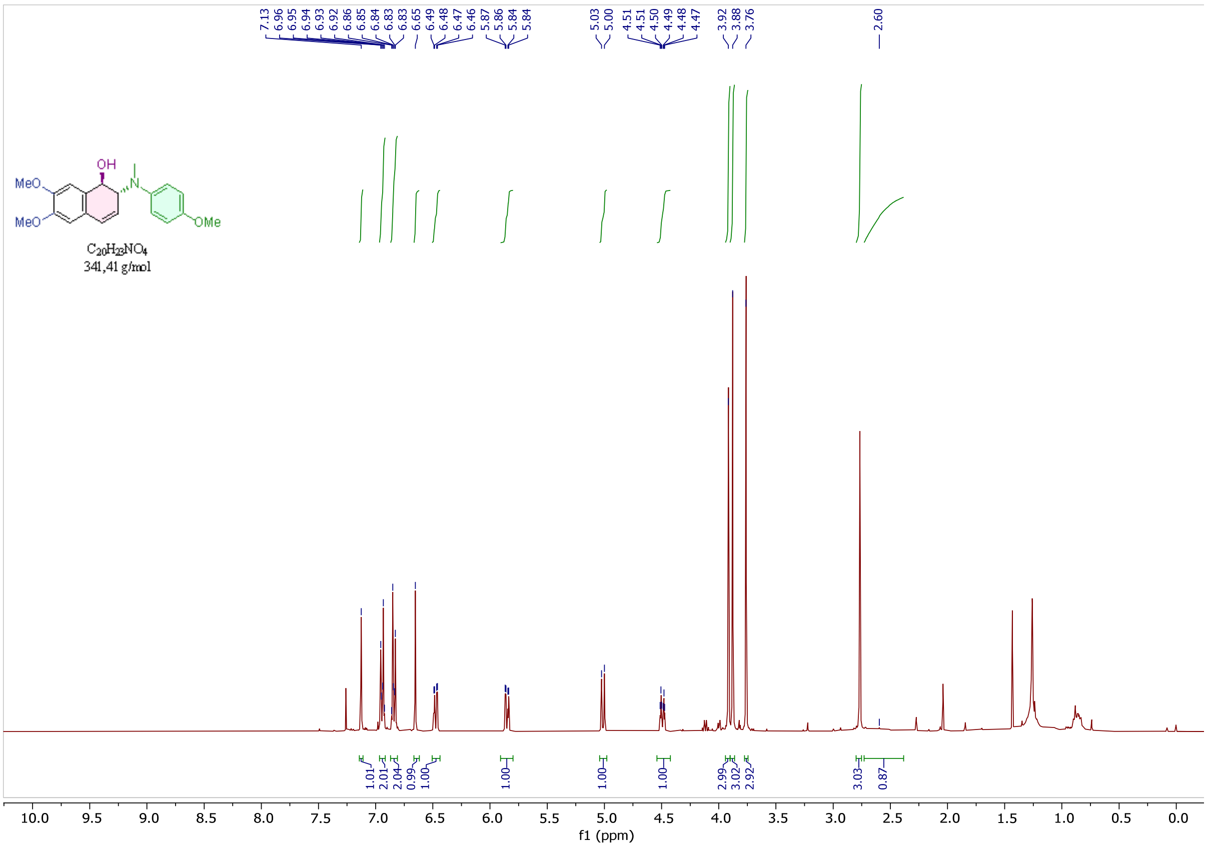

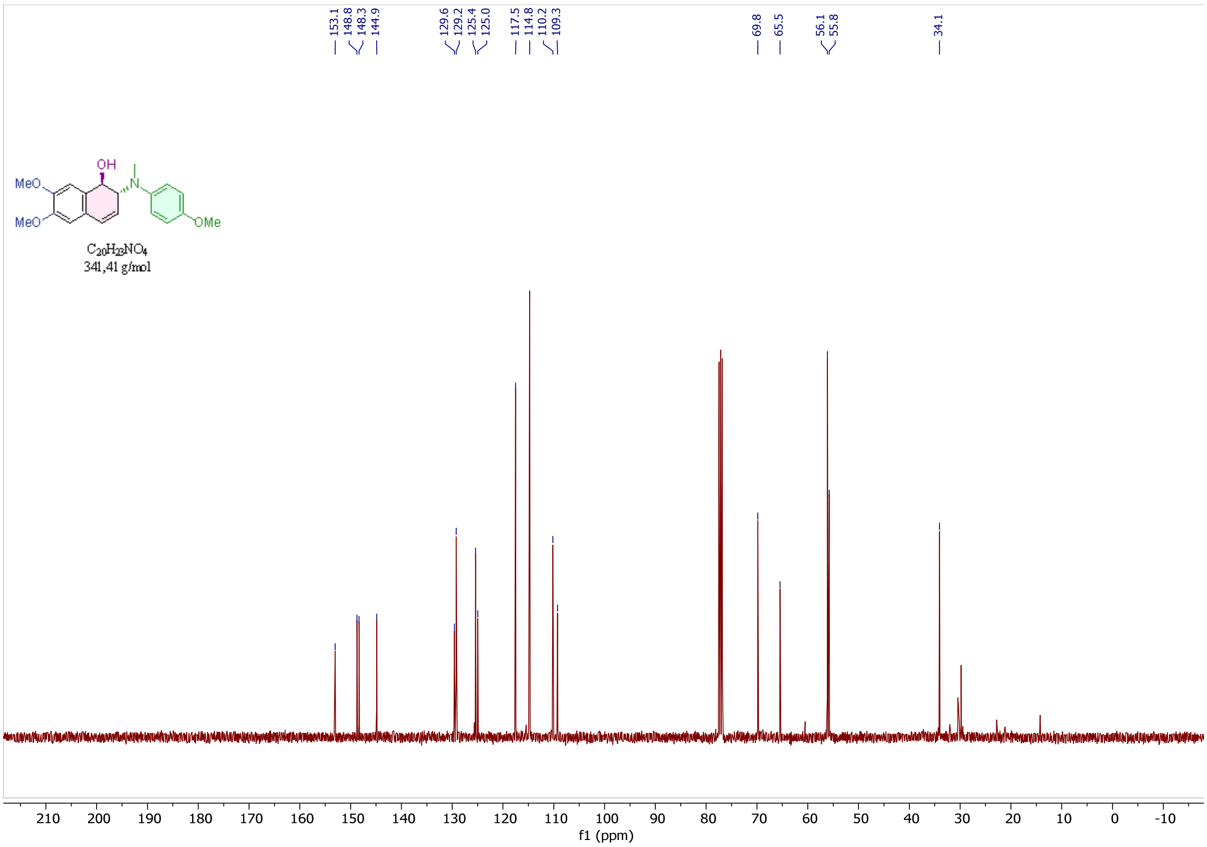

^{1}H NMR (400 MHz, $CDCl_3$), $^{13}C\{^{1}H\}$ NMR (101 MHz, $CDCl_3$) and ^{19}F NMR (337 MHz, $CDCl_3$) Analysis of Compound **7ic**

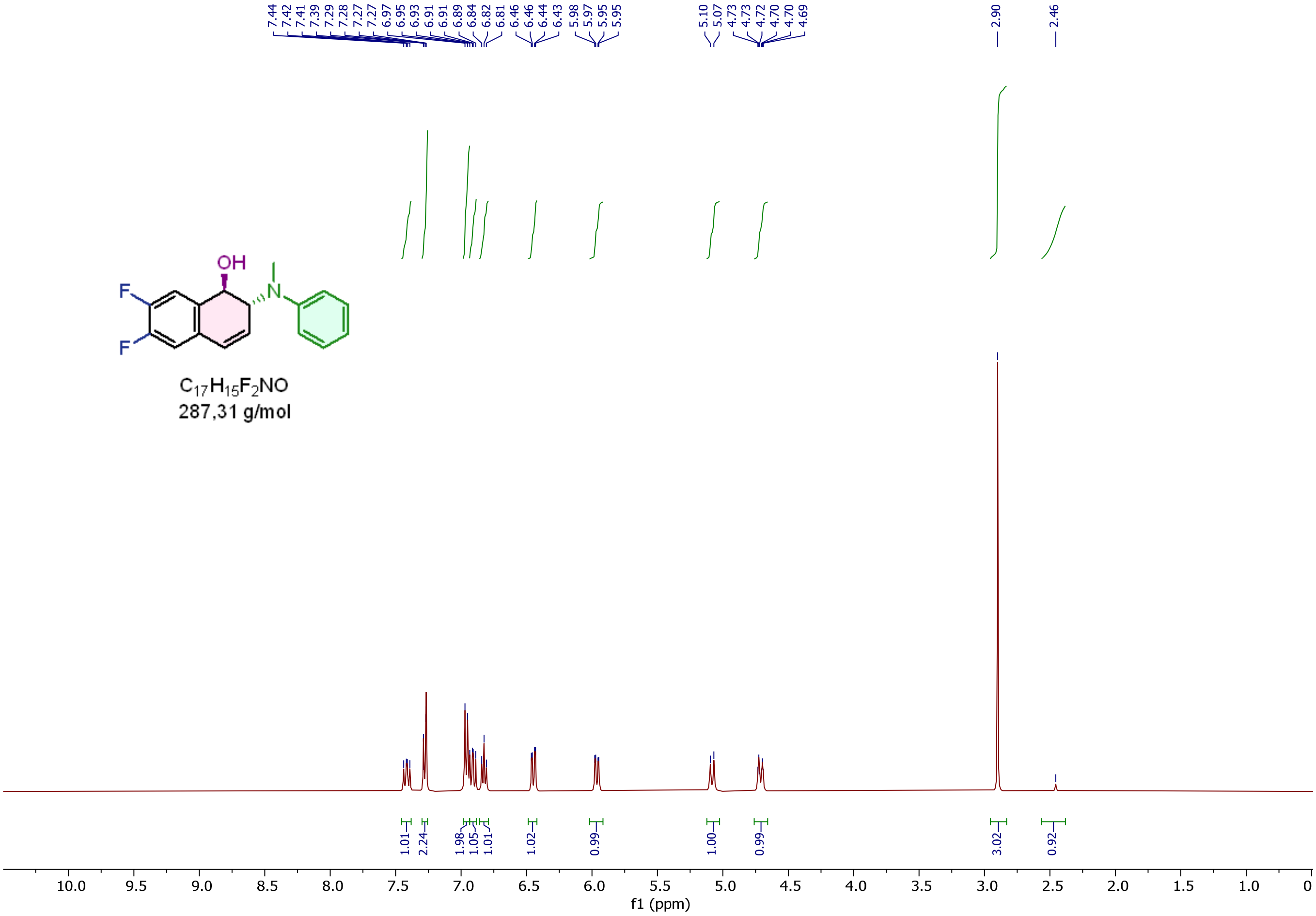

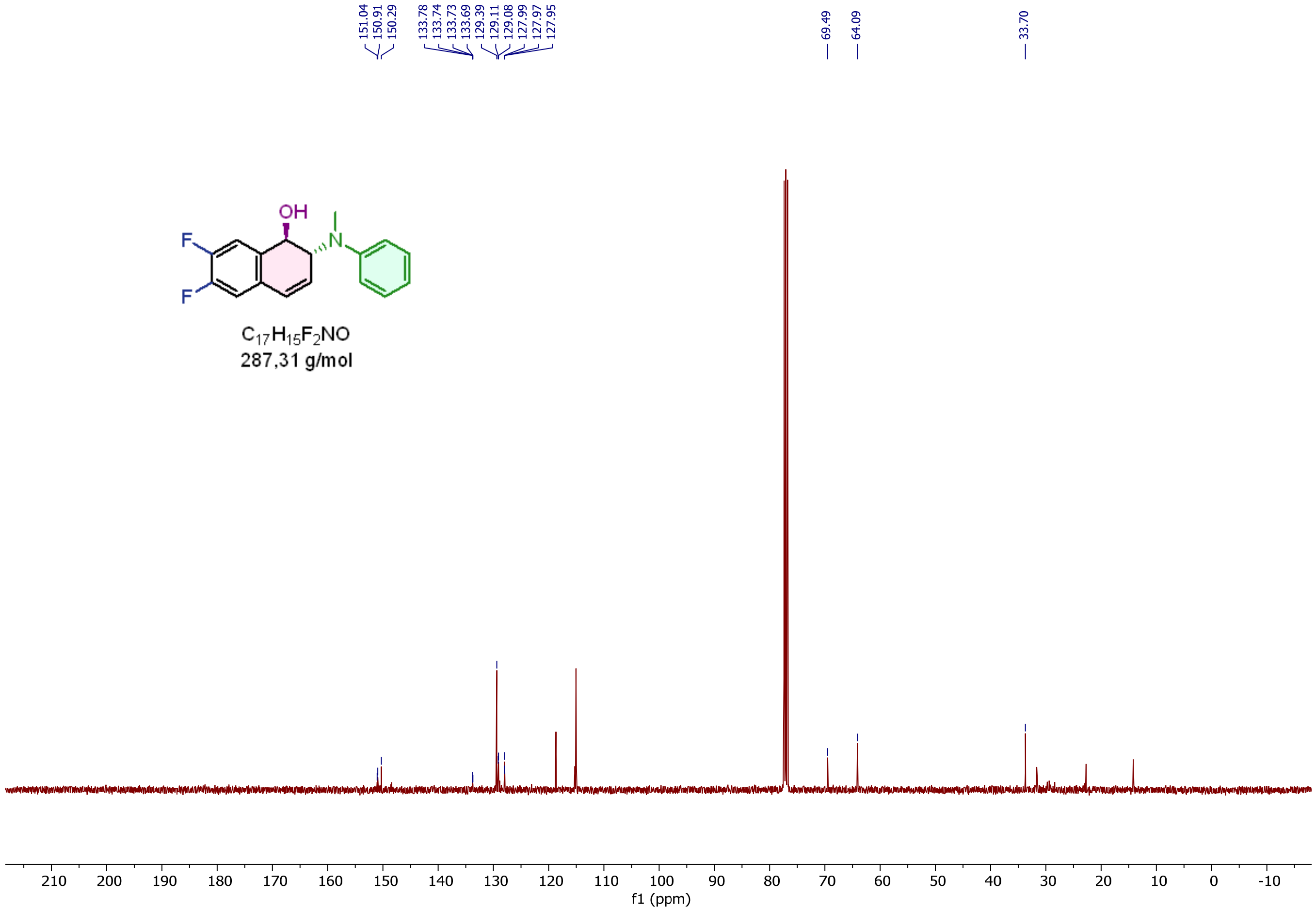

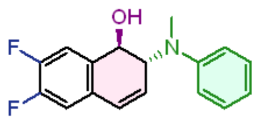

$C_{17}H_{15}F_2NO$
287,31 g/mol

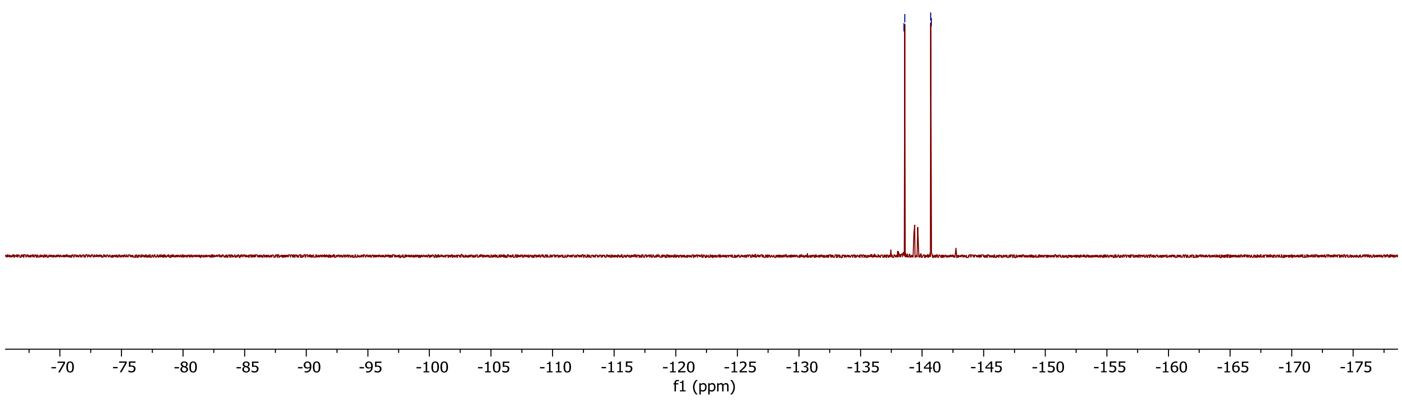

^1H NMR (400 MHz, $CDCl_3$), $^{13}C\{^1H\}$ NMR (101 MHz, $CDCl_3$) and ^{19}F NMR (337 MHz, $CDCl_3$) Analysis of Compound **7id**

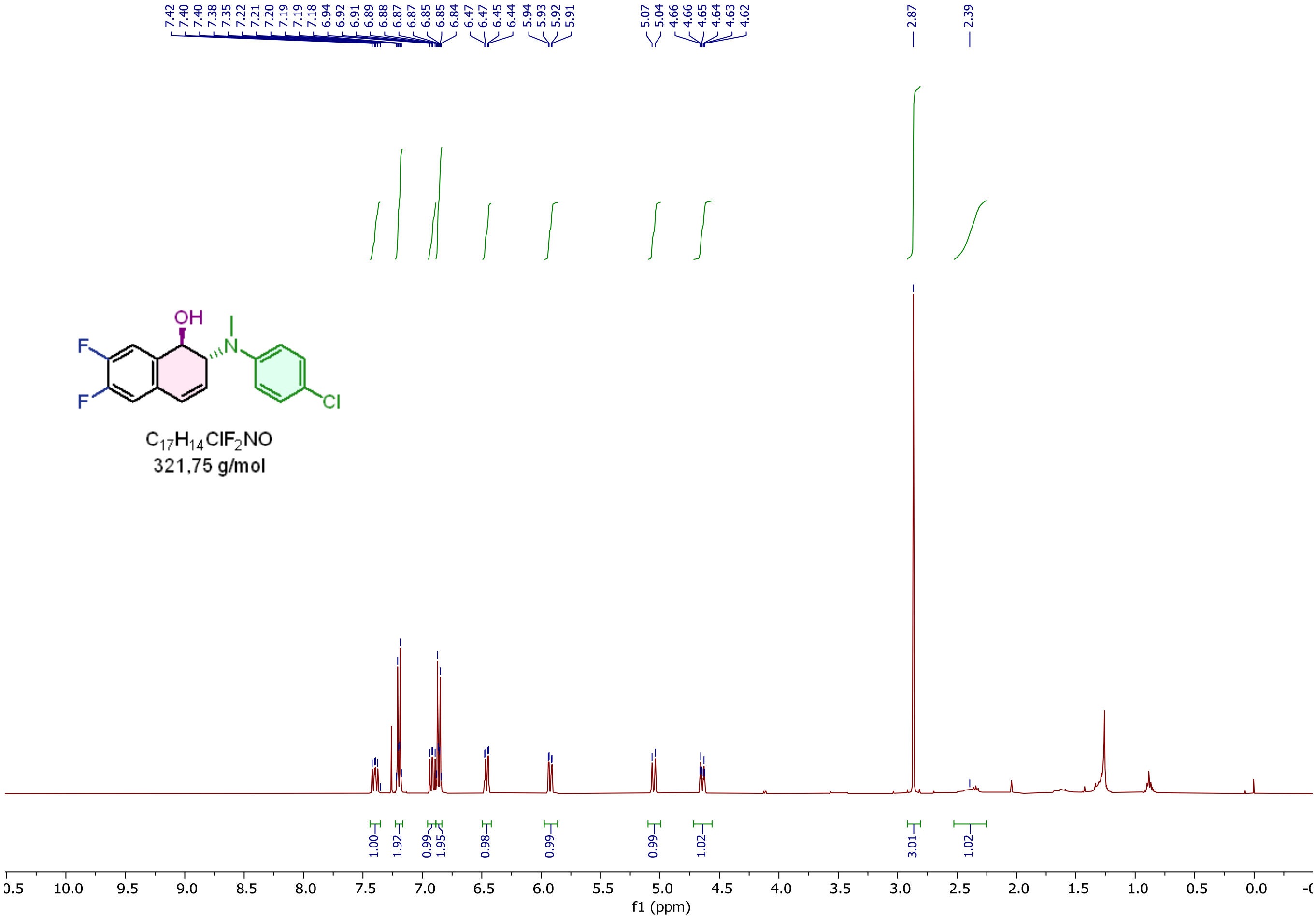

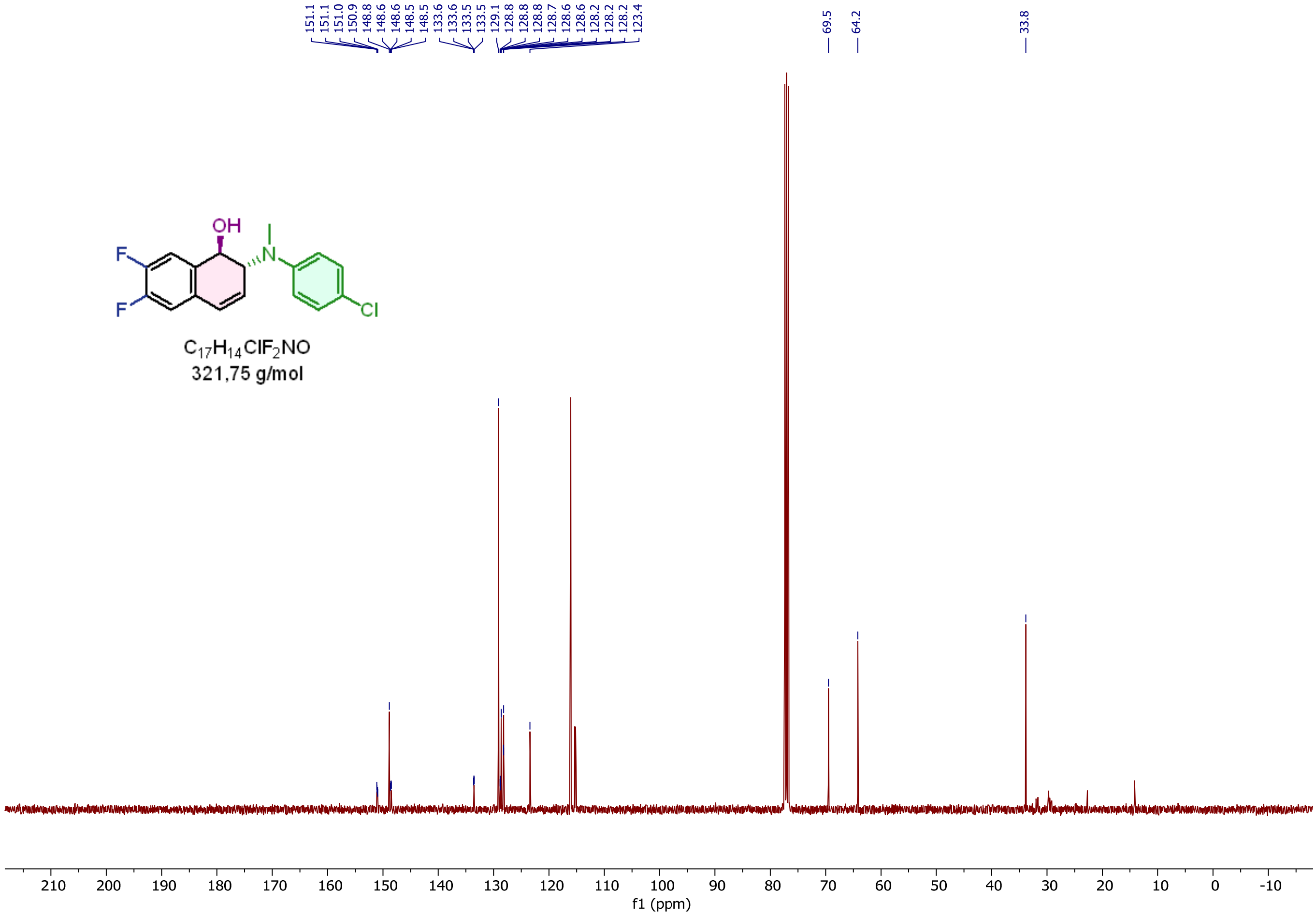

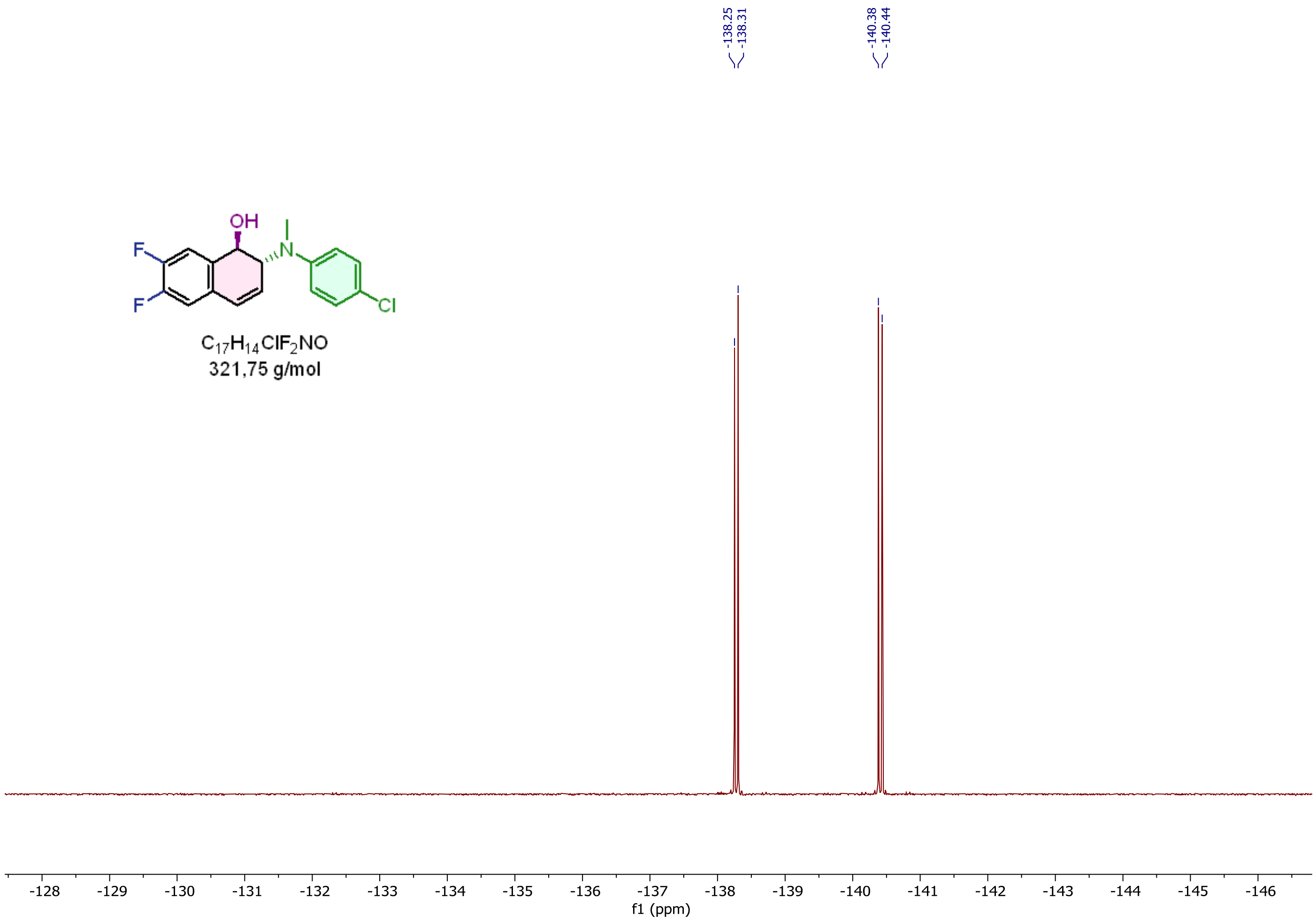
C17H14ClF2NO
321,75 g/mol
-138.25
-138.31
-140.38
-140.44
-128
-129
-130
-131
-132
-133
-134
-135
-136
-137
-138
-139
-140
-141
-142
-143
-144
-145
-146
f1 (ppm)

^{1}H NMR (400 MHz, $CDCl_3$) and ^{13}C{^{1}H} NMR (101 MHz, $CDCl_3$) Analysis of Compound **7ja**

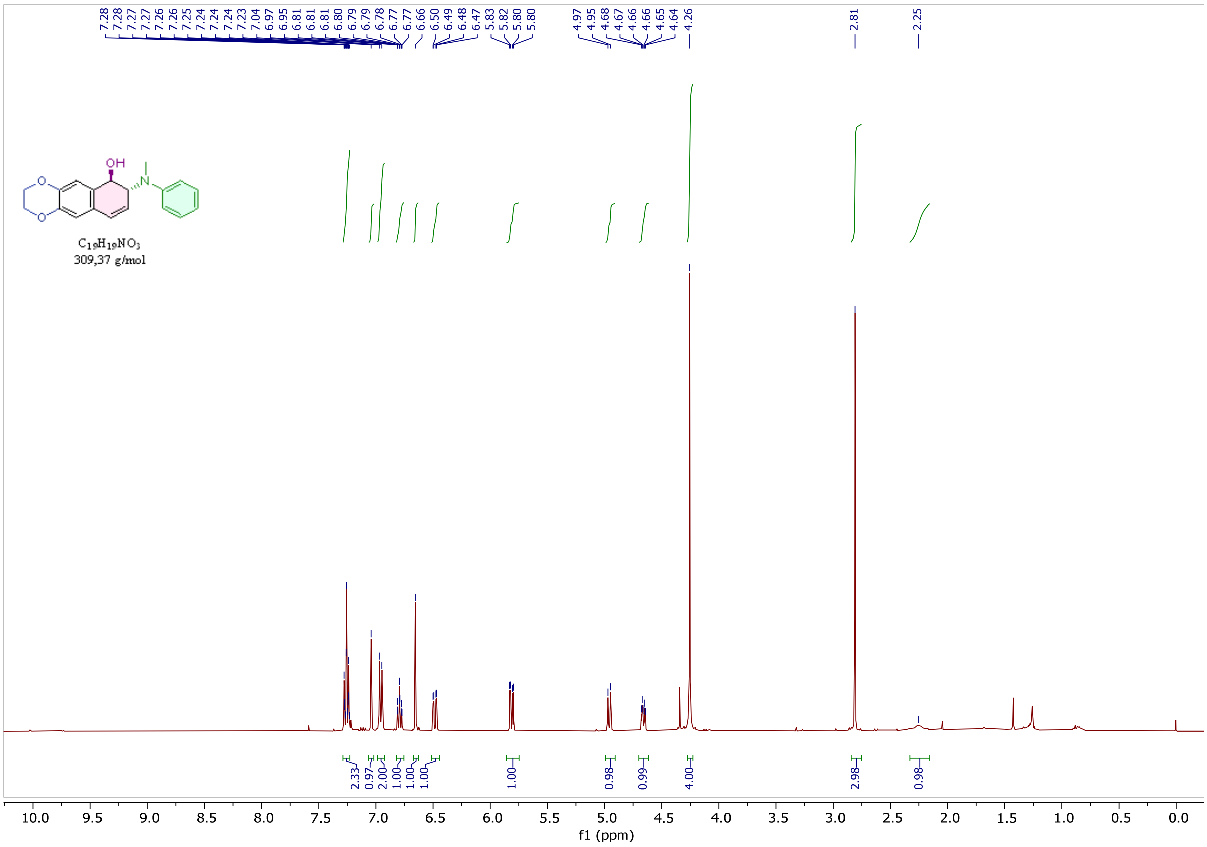

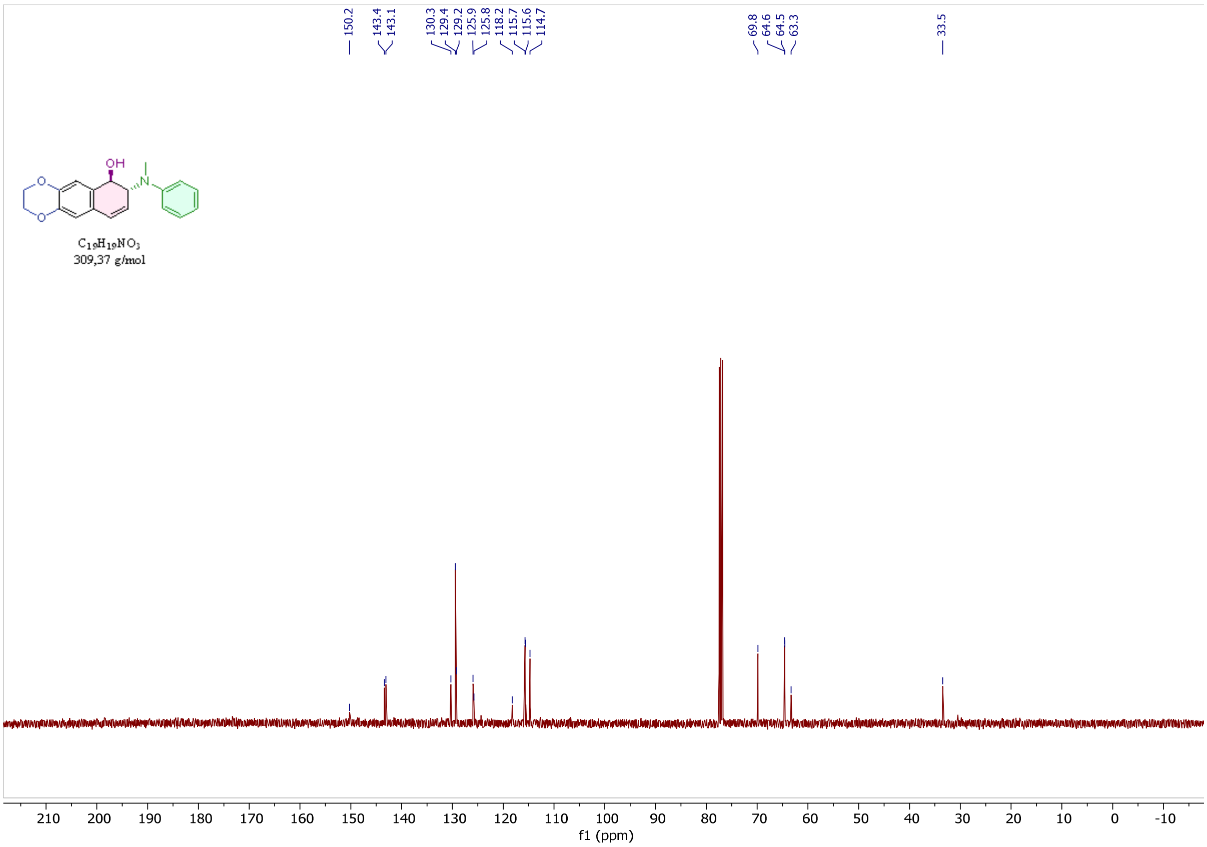

^{1}H NMR (400 MHz, $CDCl_3$) and ^{13}C{^{1}H} NMR (101 MHz, $CDCl_3$) Analysis of Compound **7jb**

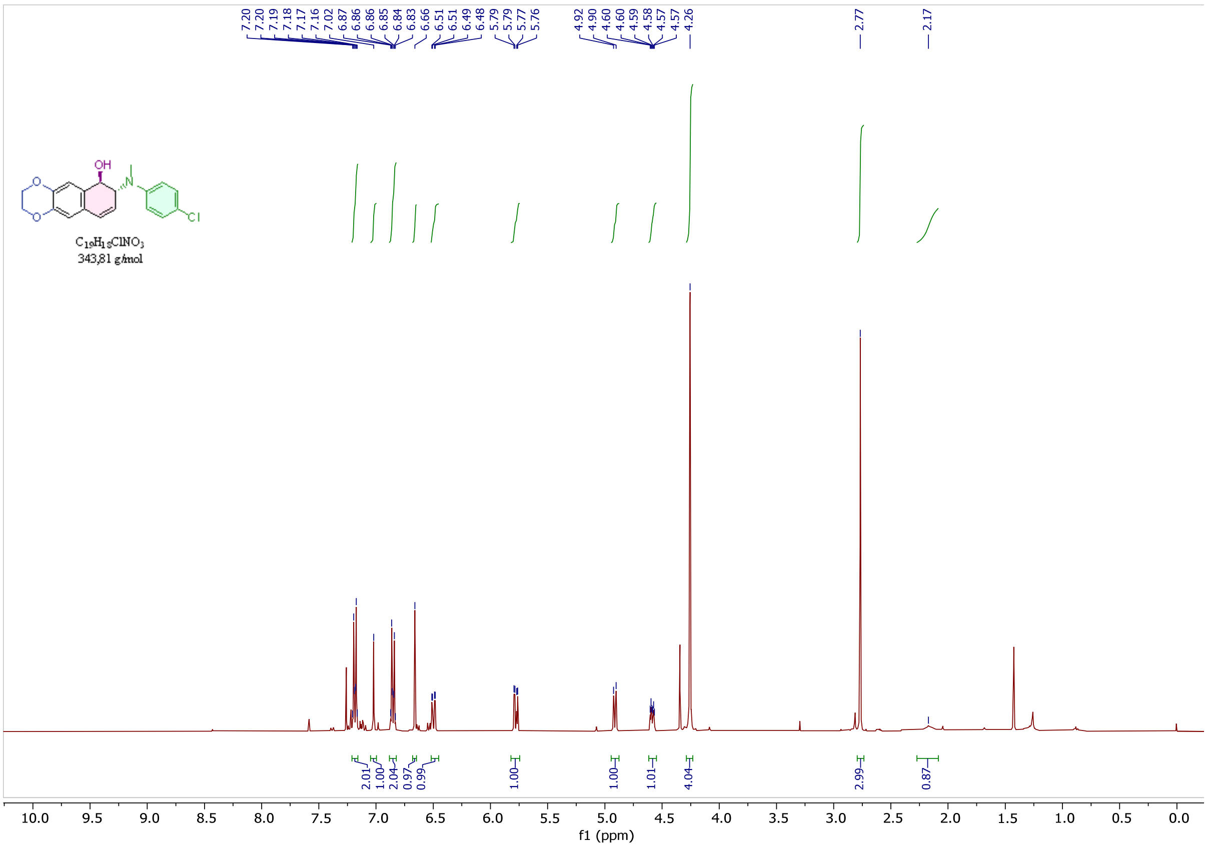

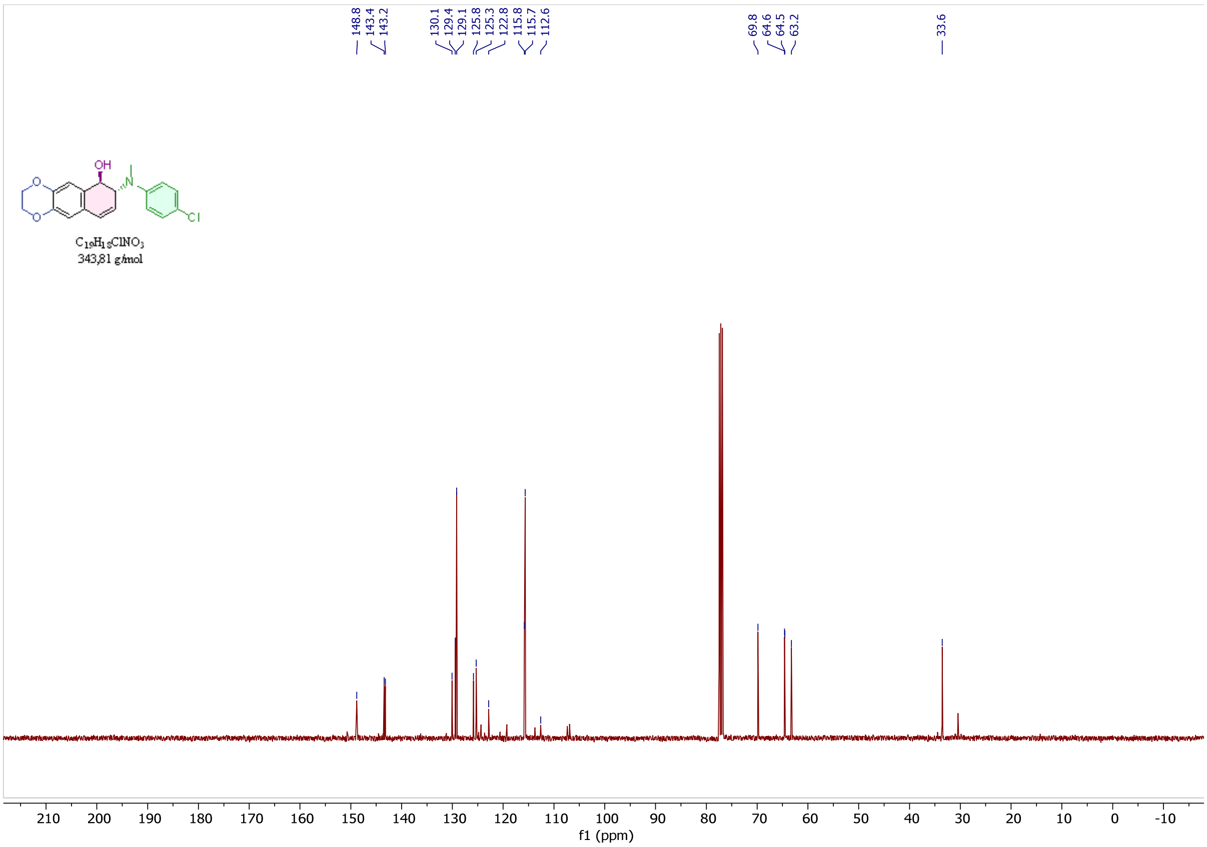

^{1}H NMR (400 MHz, $CDCl_3$) and ^{13}C{^{1}H} NMR (101 MHz, $CDCl_3$) Analysis of Compound **7jc**

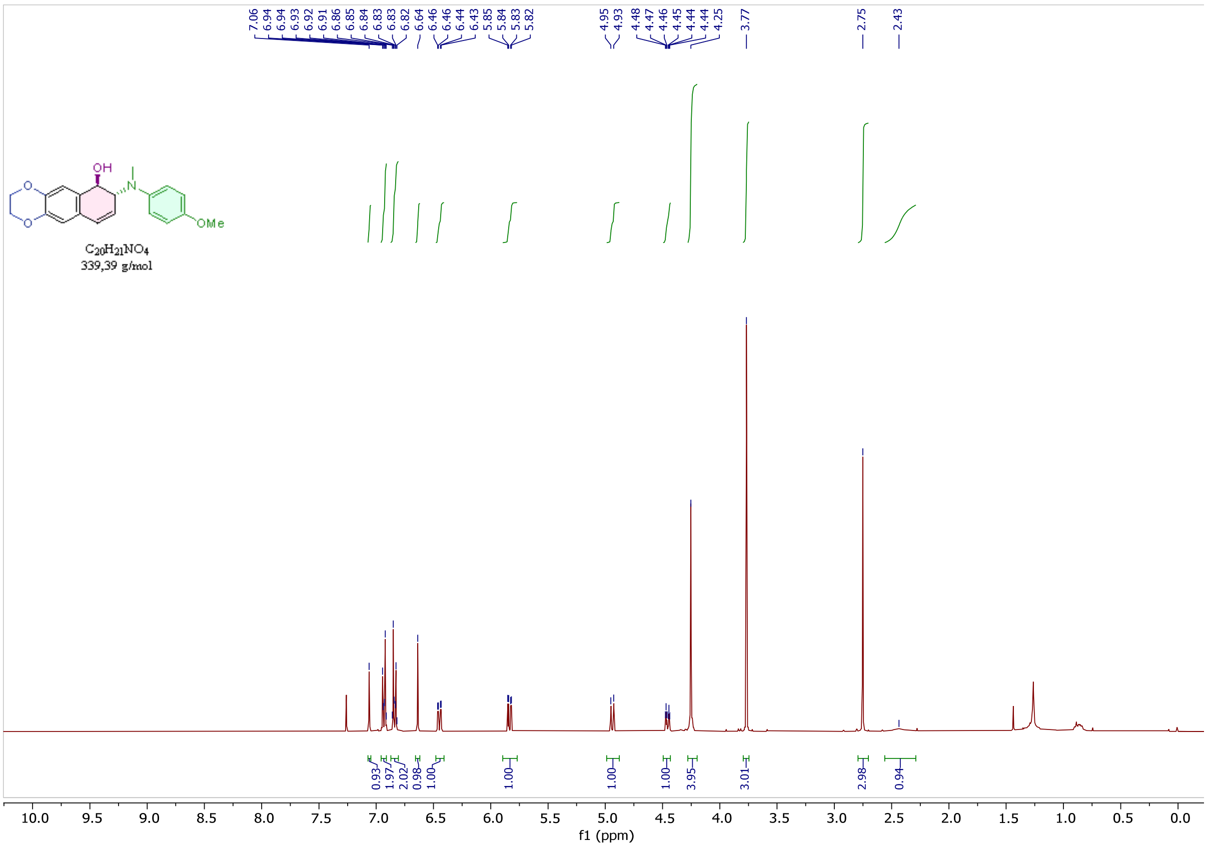

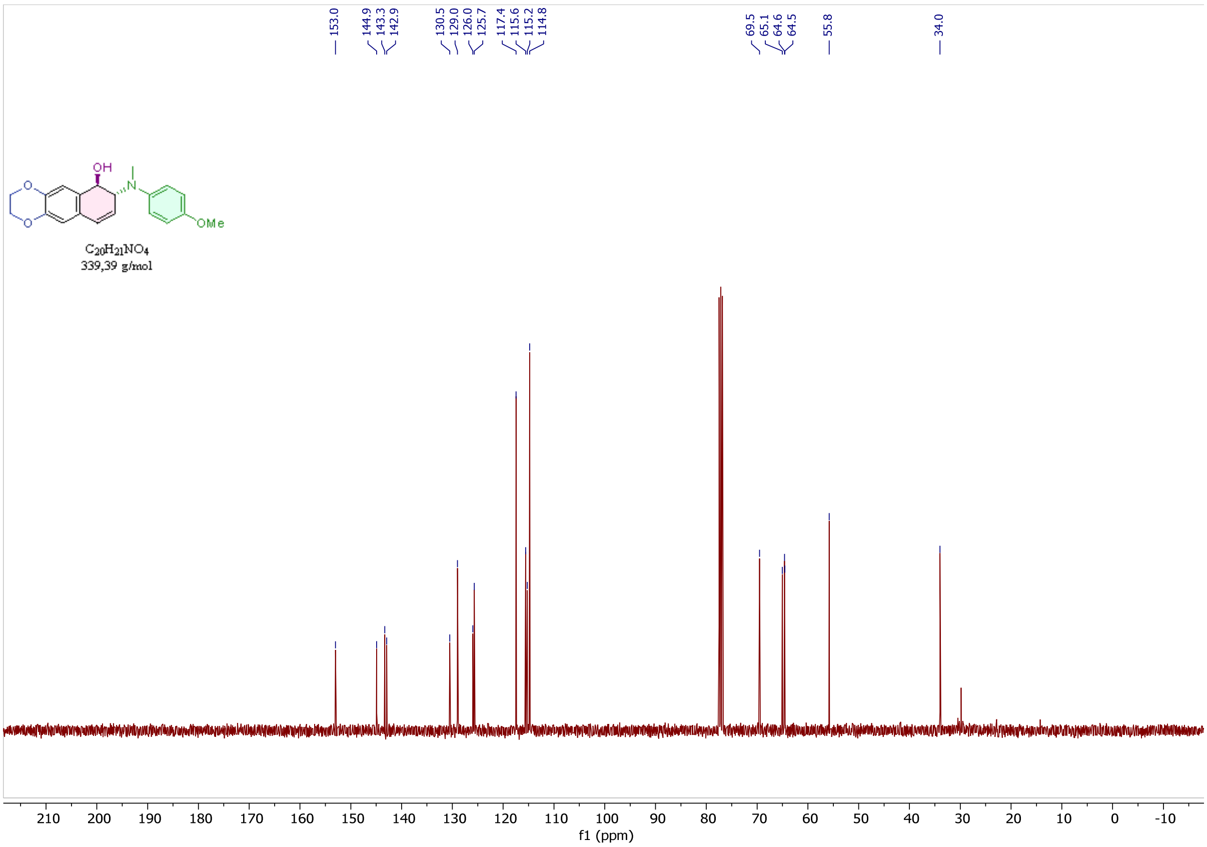

^{1}H NMR (400 MHz, $CDCl_3$) and ^{13}C{^{1}H} NMR (101 MHz, $CDCl_3$) Analysis of Compound **7ka**

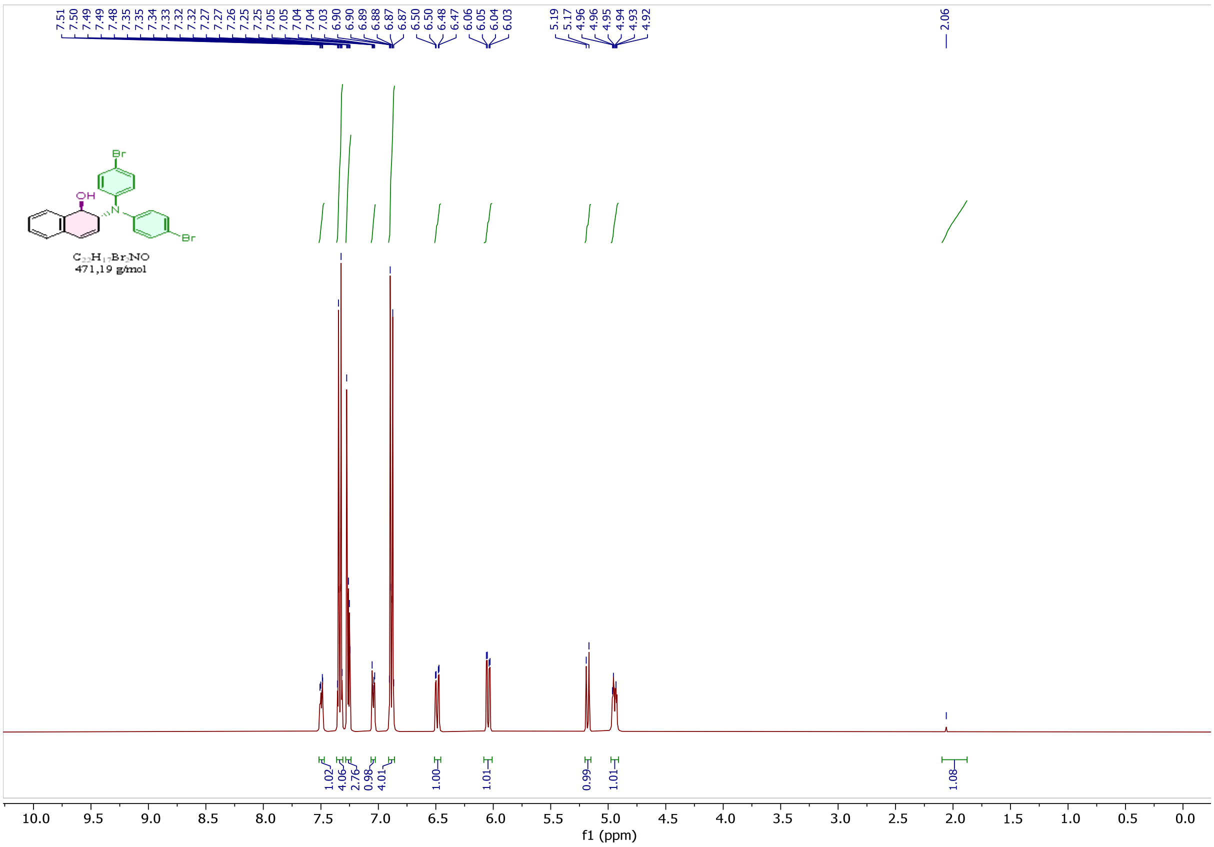

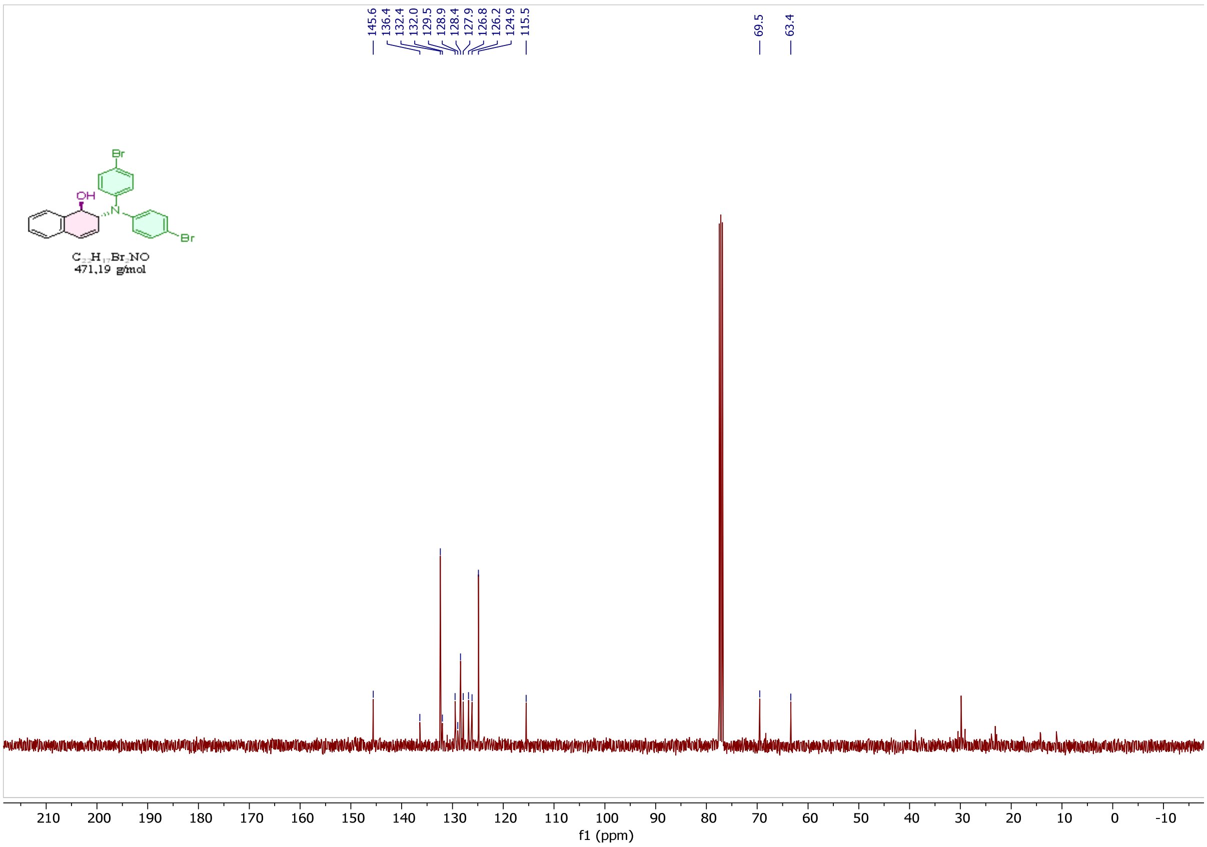

^{1}H NMR (400 MHz, $CDCl_3$) and $^{13}C\{^{1}H\}$ NMR (101 MHz, $CDCl_3$) Analysis of Compound **7kb**

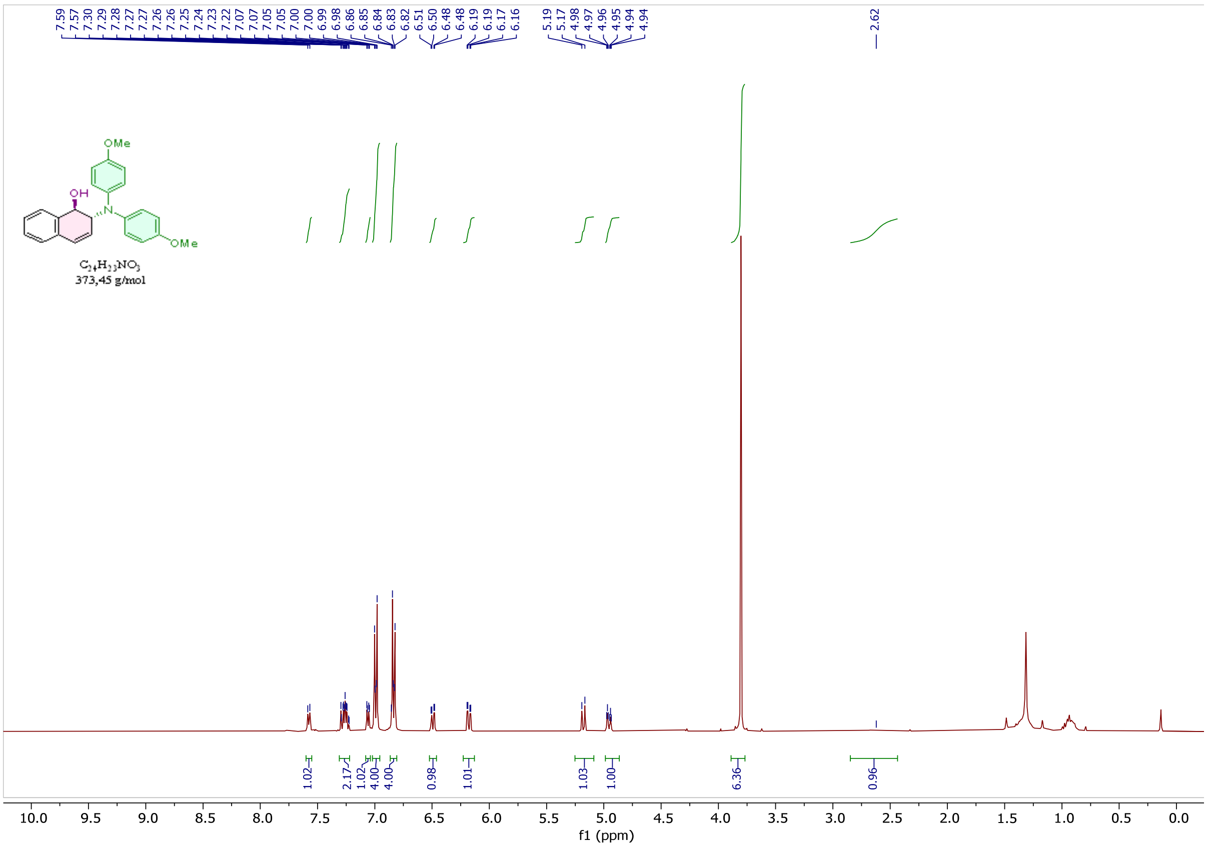

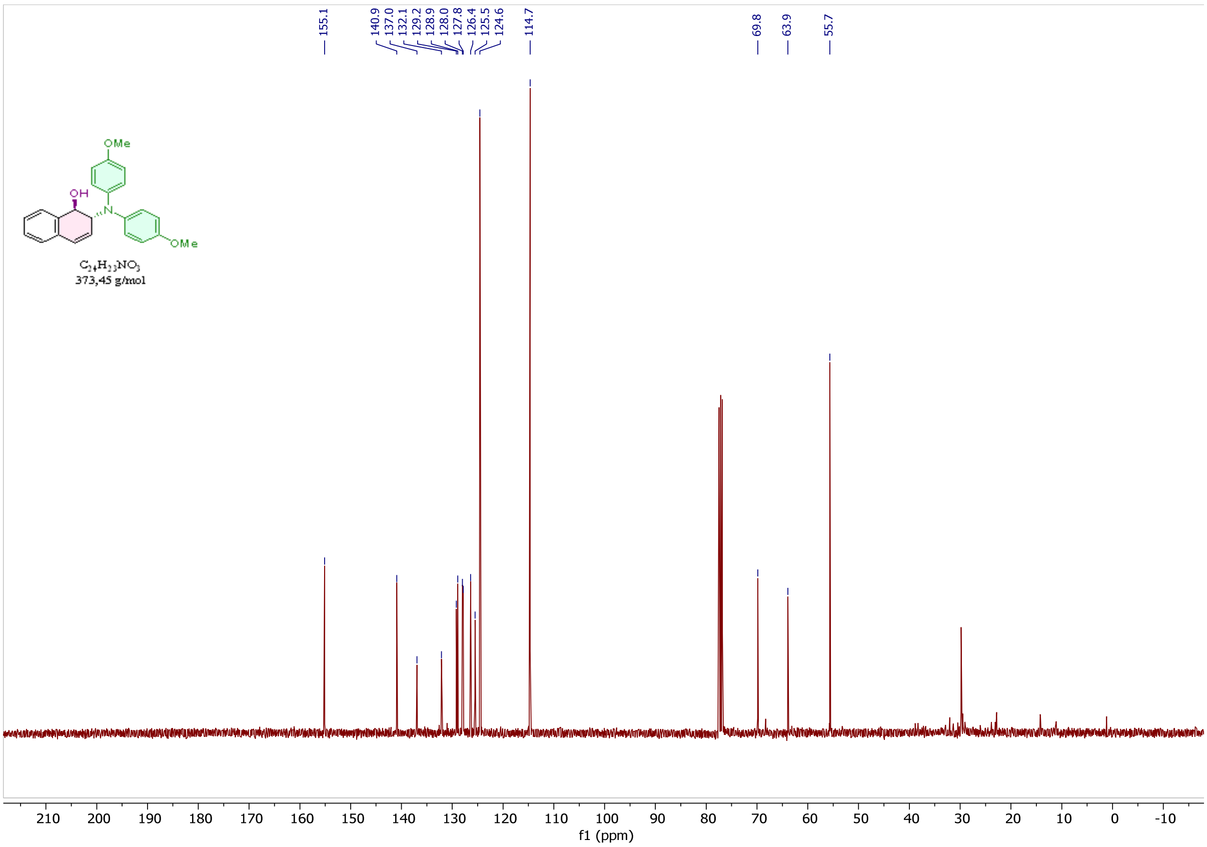

^{1}H NMR (400 MHz, $CDCl_3$) and ^{13}C{^{1}H} NMR (101 MHz, $CDCl_3$) Analysis of Compound **7la**

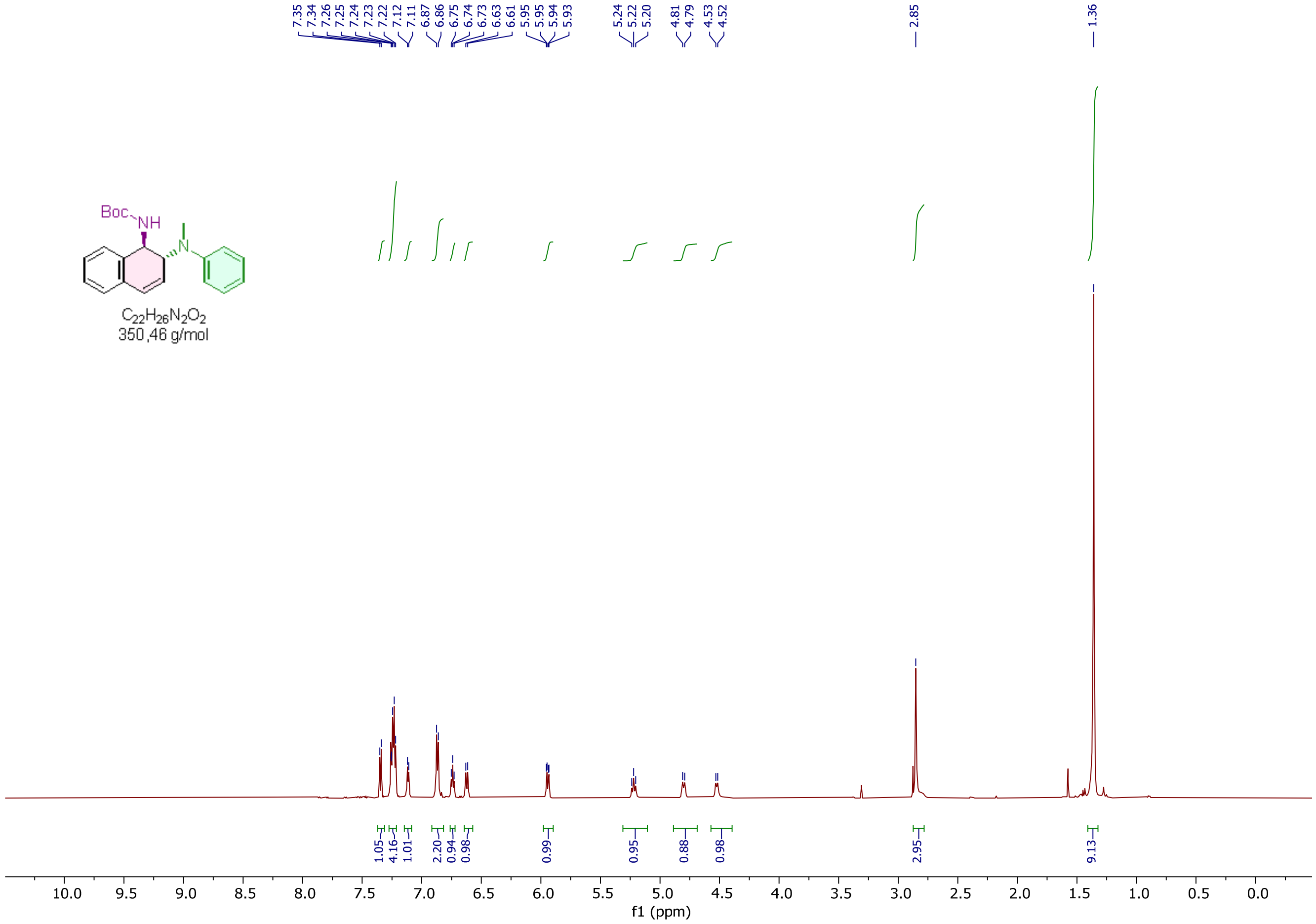

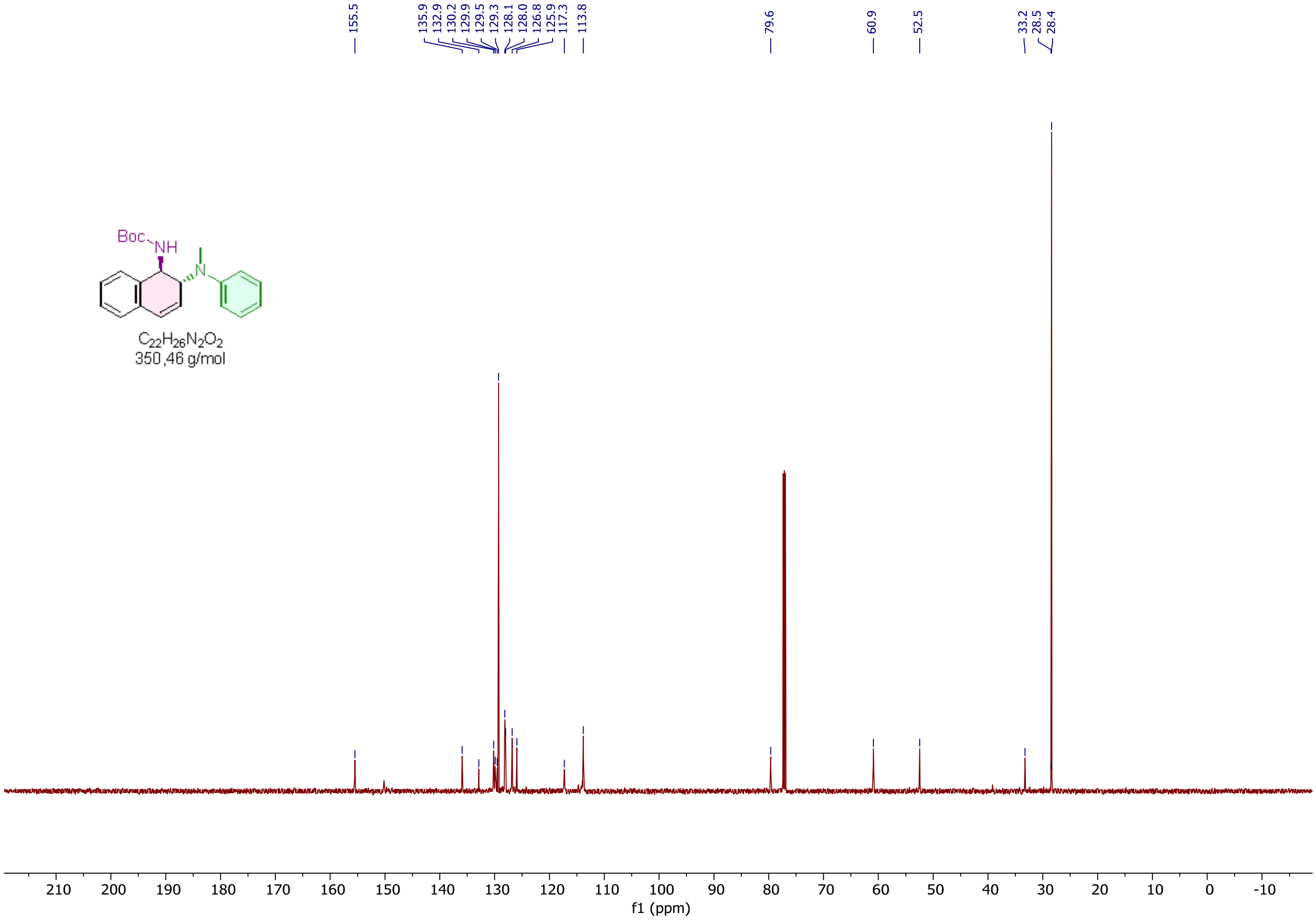

^{1}H NMR (400 MHz, $CDCl_3$) and ^{13}C{^{1}H} NMR (101 MHz, $CDCl_3$) Analysis of Compound **7lb**

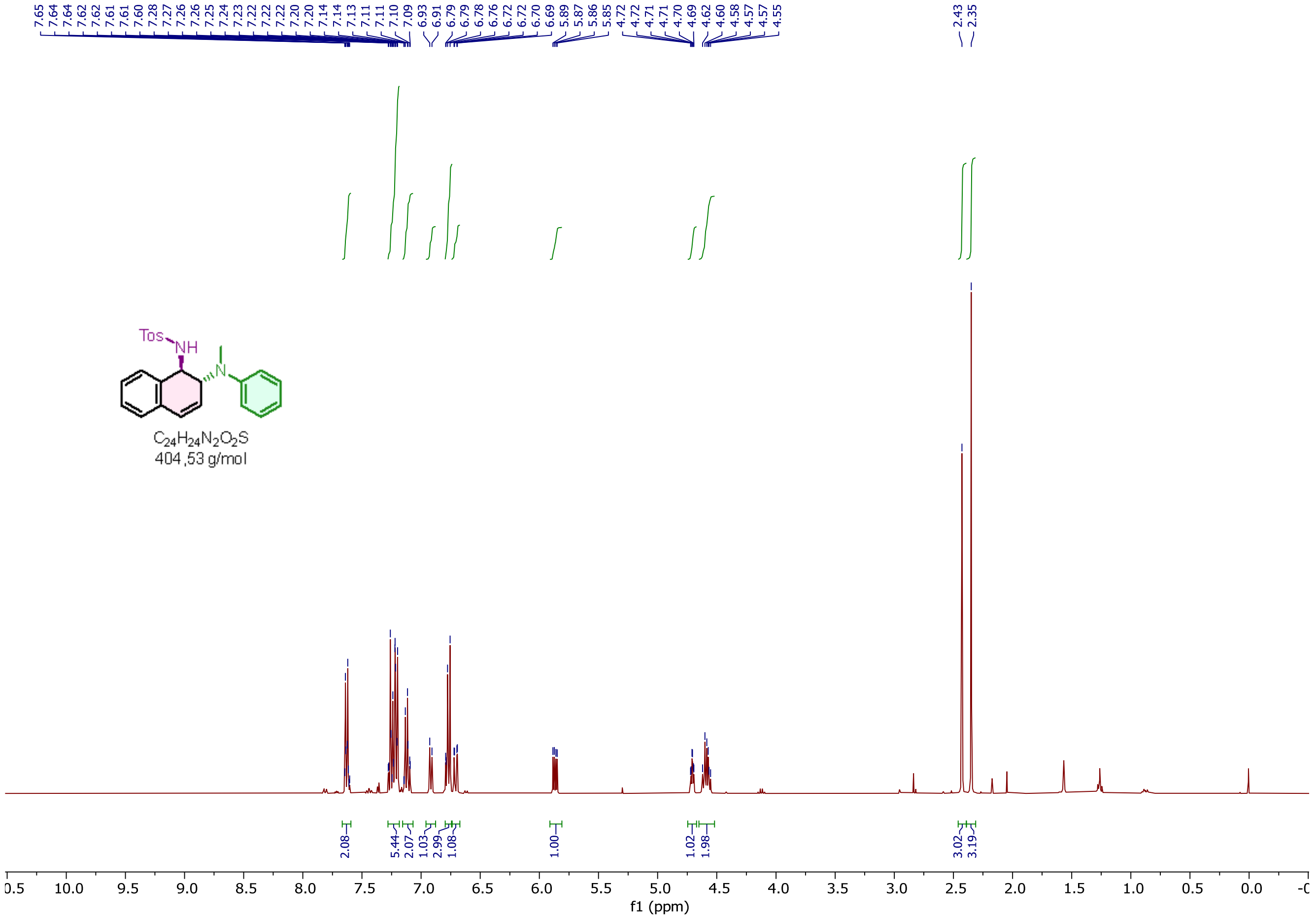

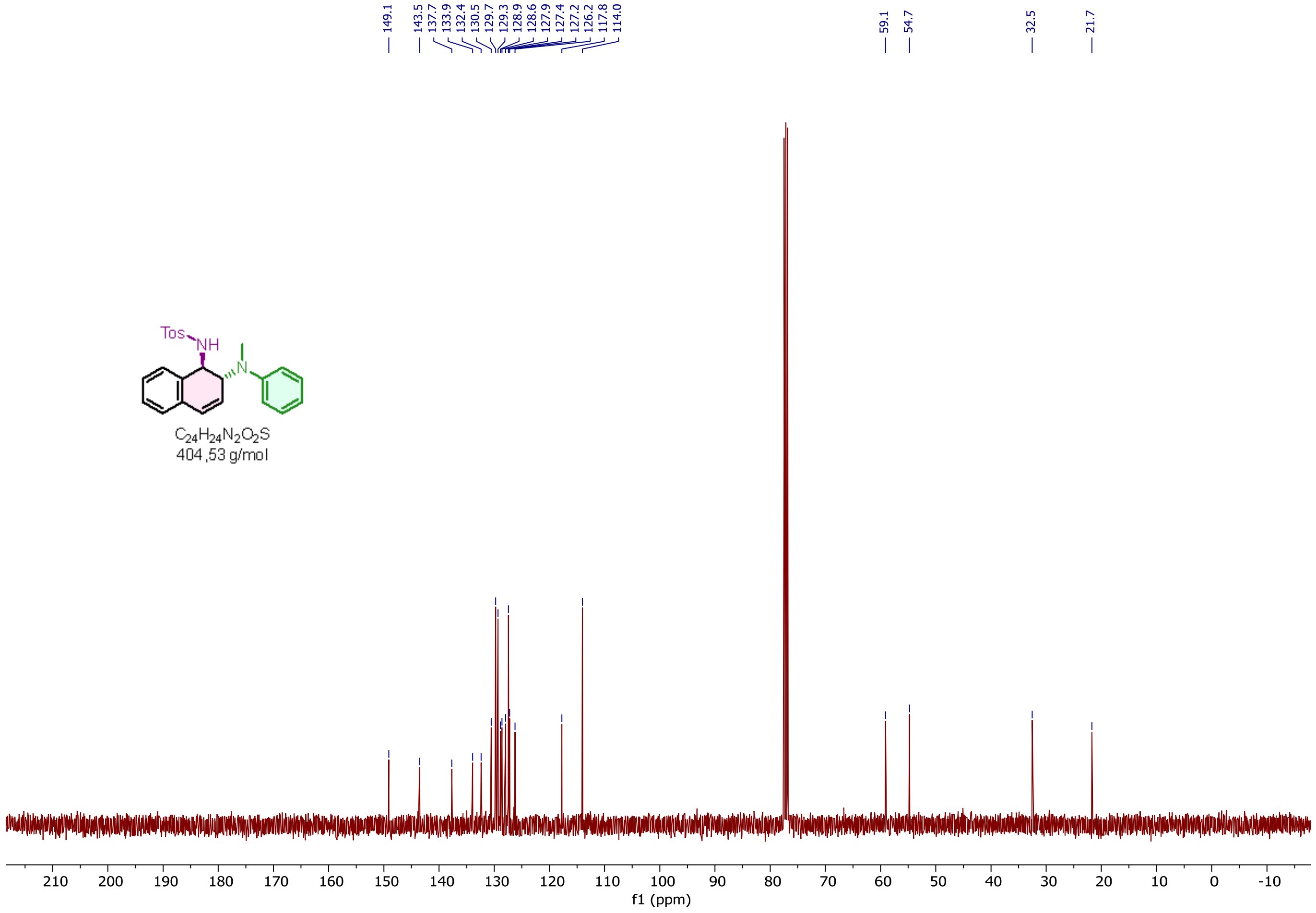

^{1}H NMR (400 MHz, $CDCl_3$) and $^{13}C\{^{1}H\}$ NMR (101 MHz, $CDCl_3$) Analysis of Compound **7ma**

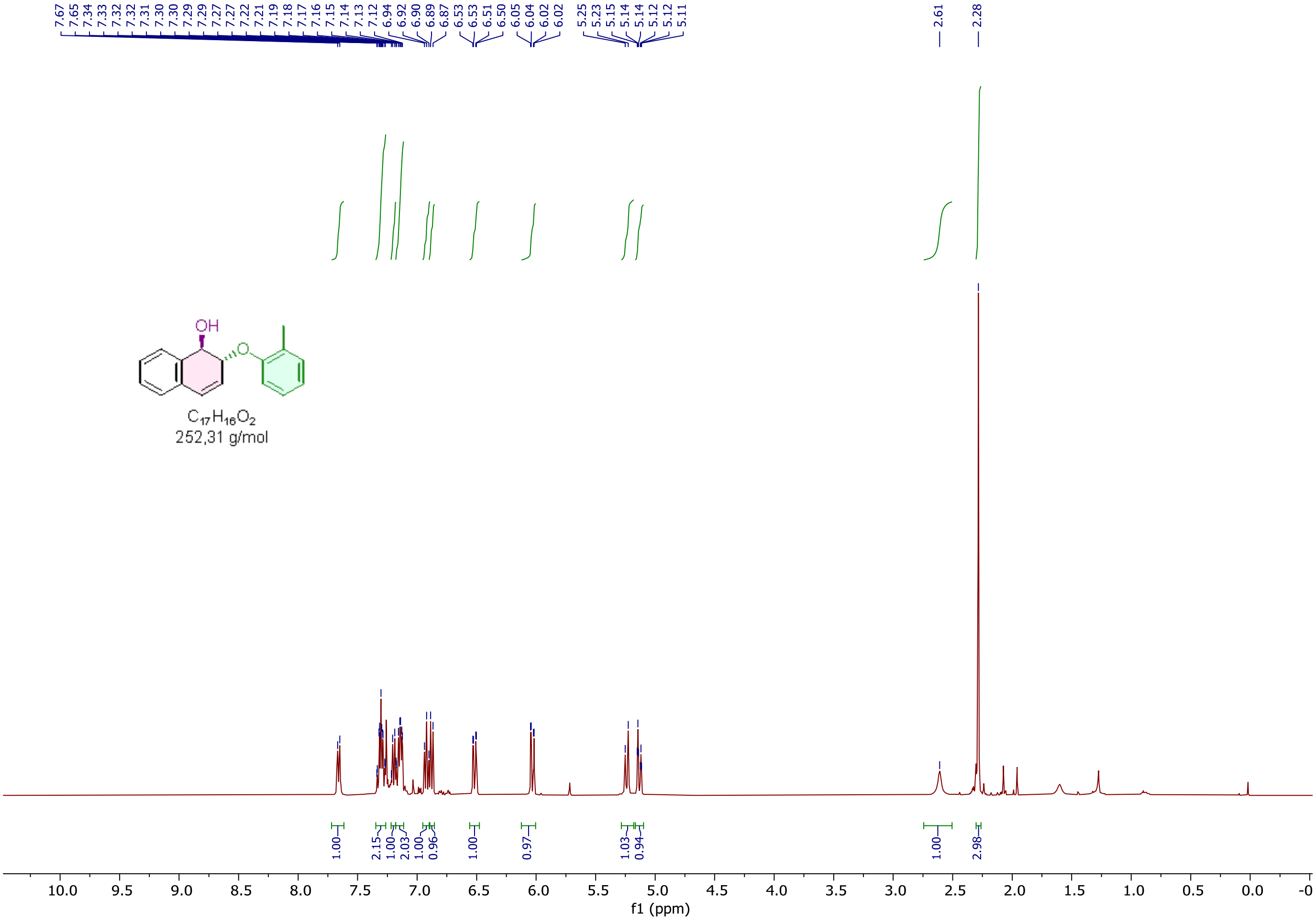

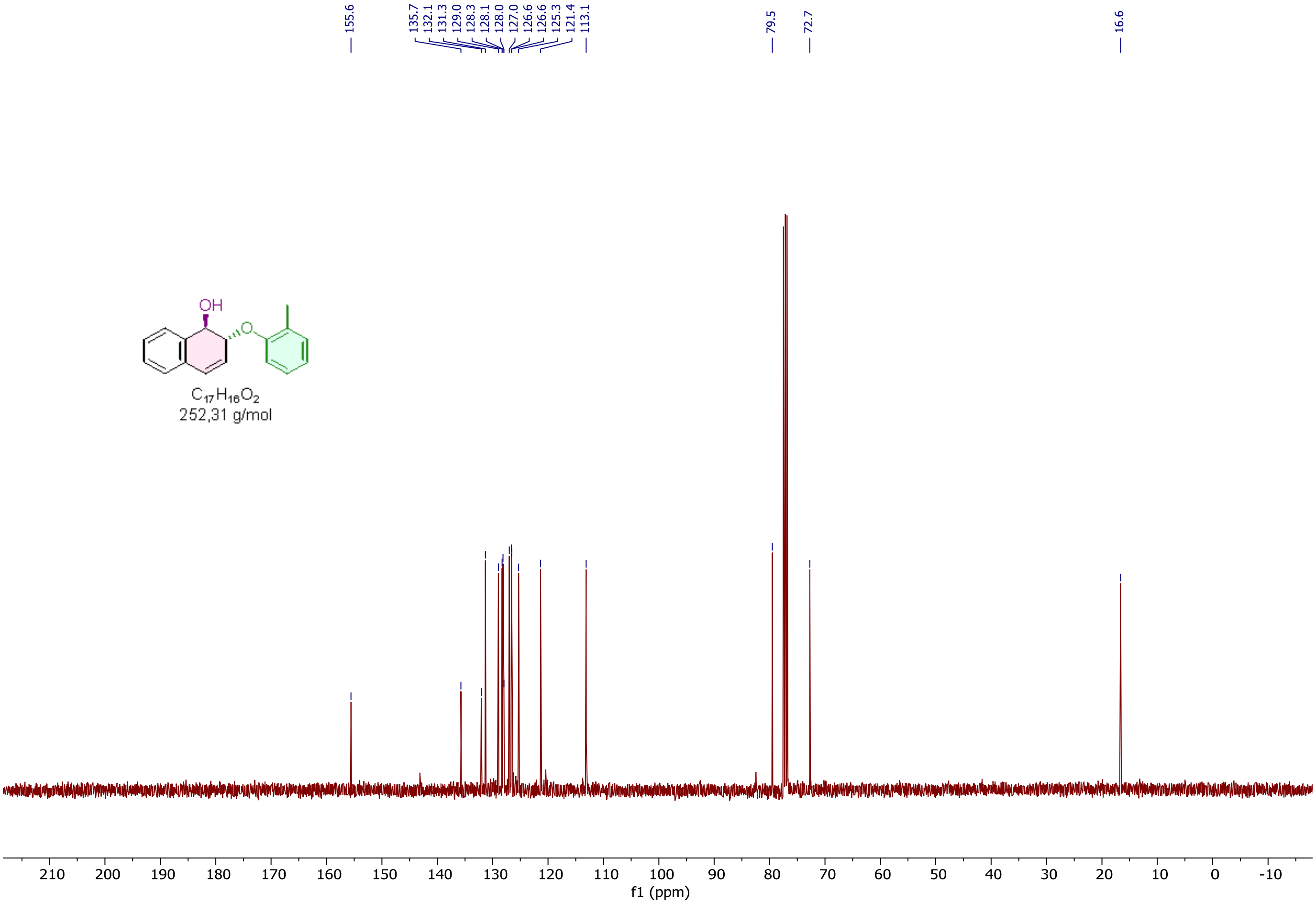

^{1}H NMR (400 MHz, $CDCl_3$) and ^{13}C{^{1}H} NMR (101 MHz, $CDCl_3$) Analysis of Compound **7mb**

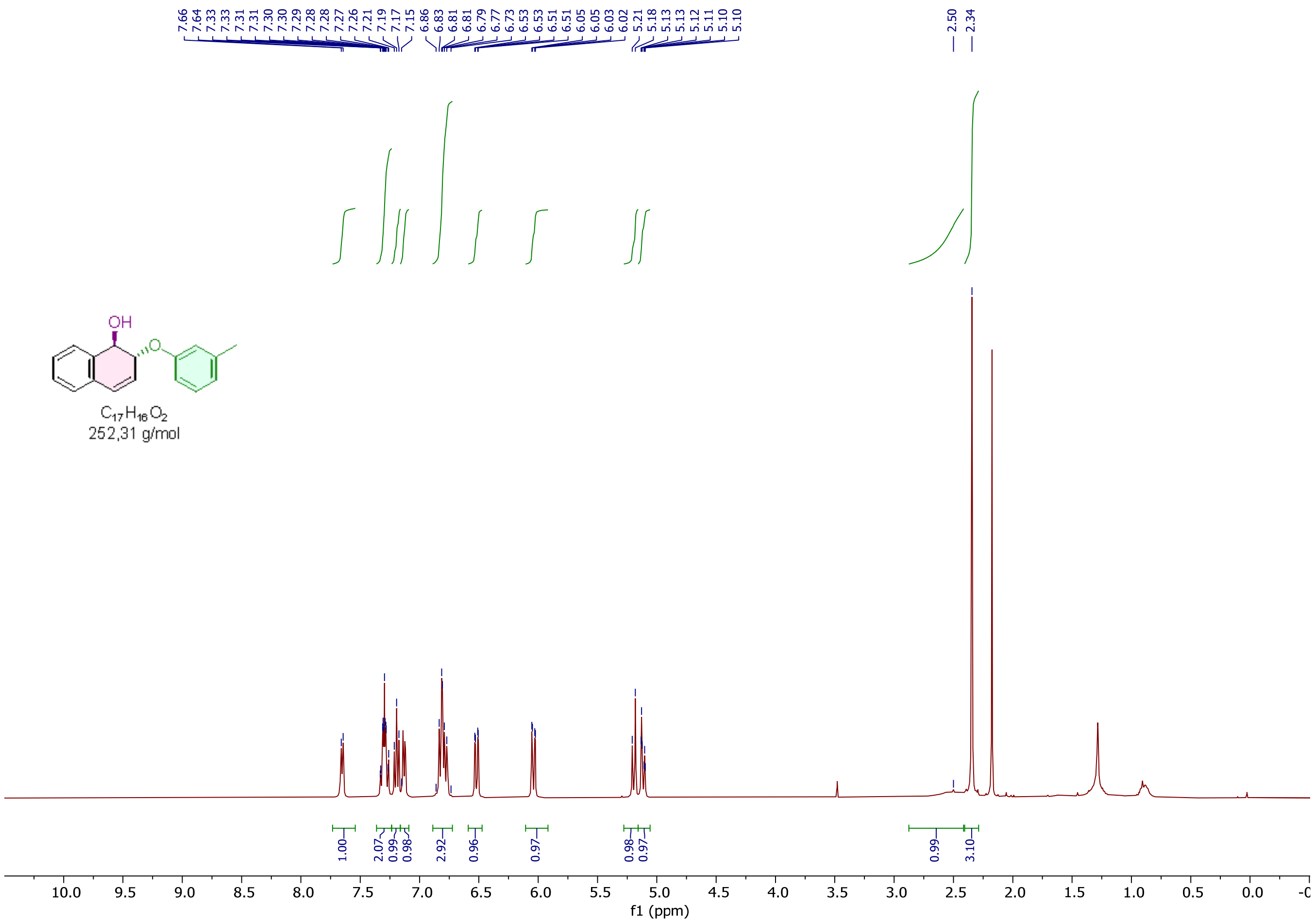

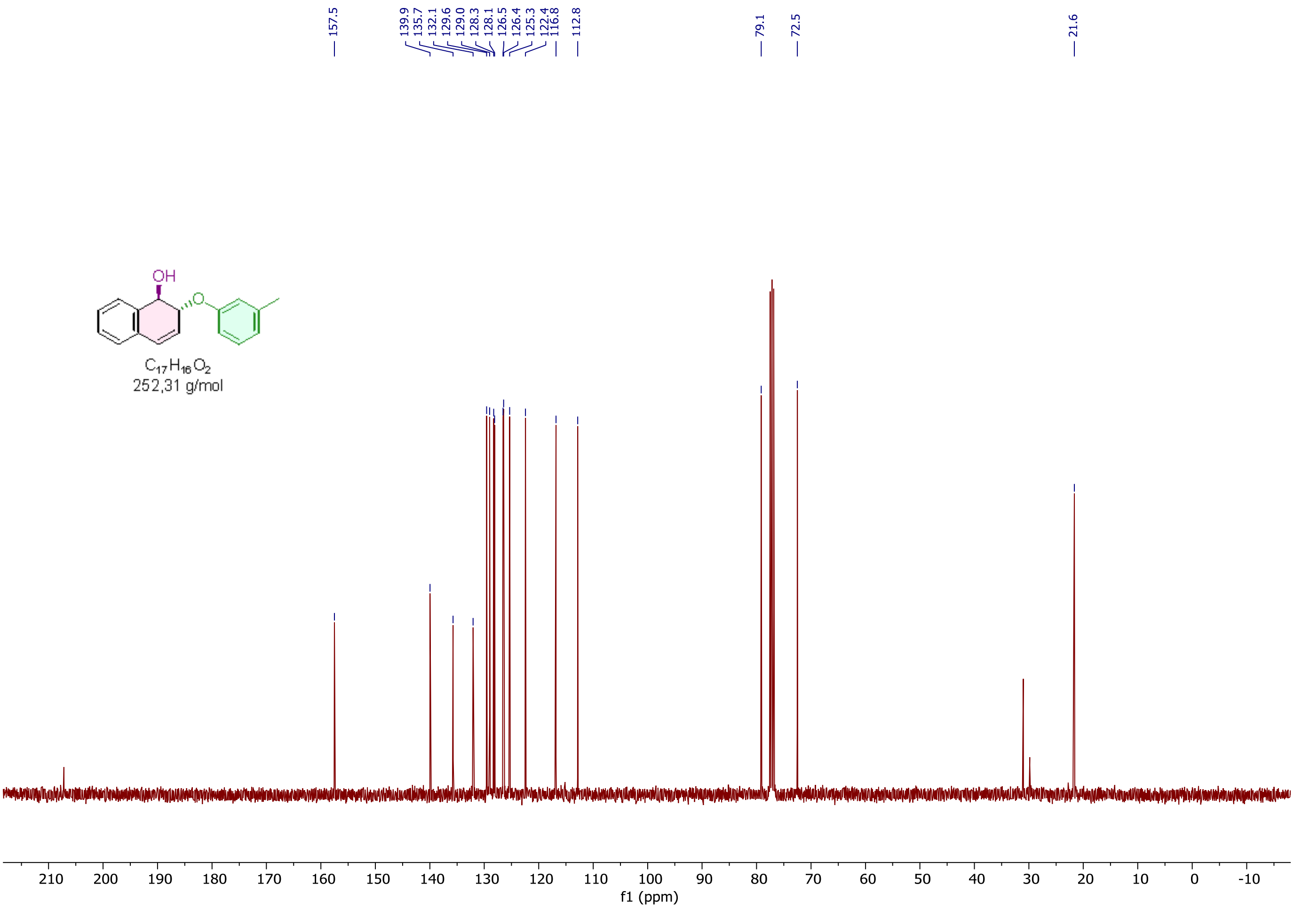

^1H NMR (400 MHz, $CDCl_3$) and $^{13}C\{^1H\}$ NMR (101 MHz, $CDCl_3$) Analysis of Compound **7mc**

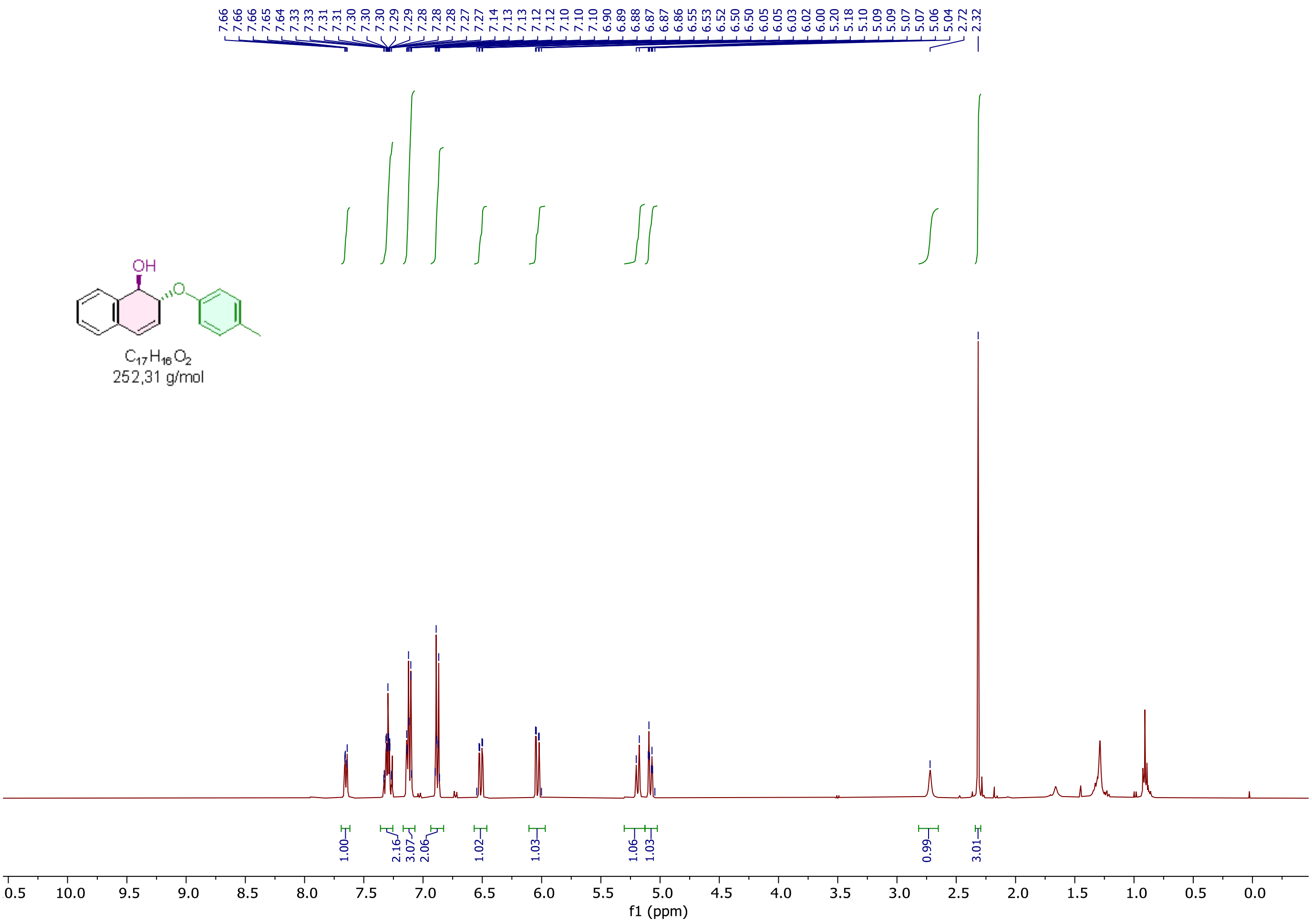

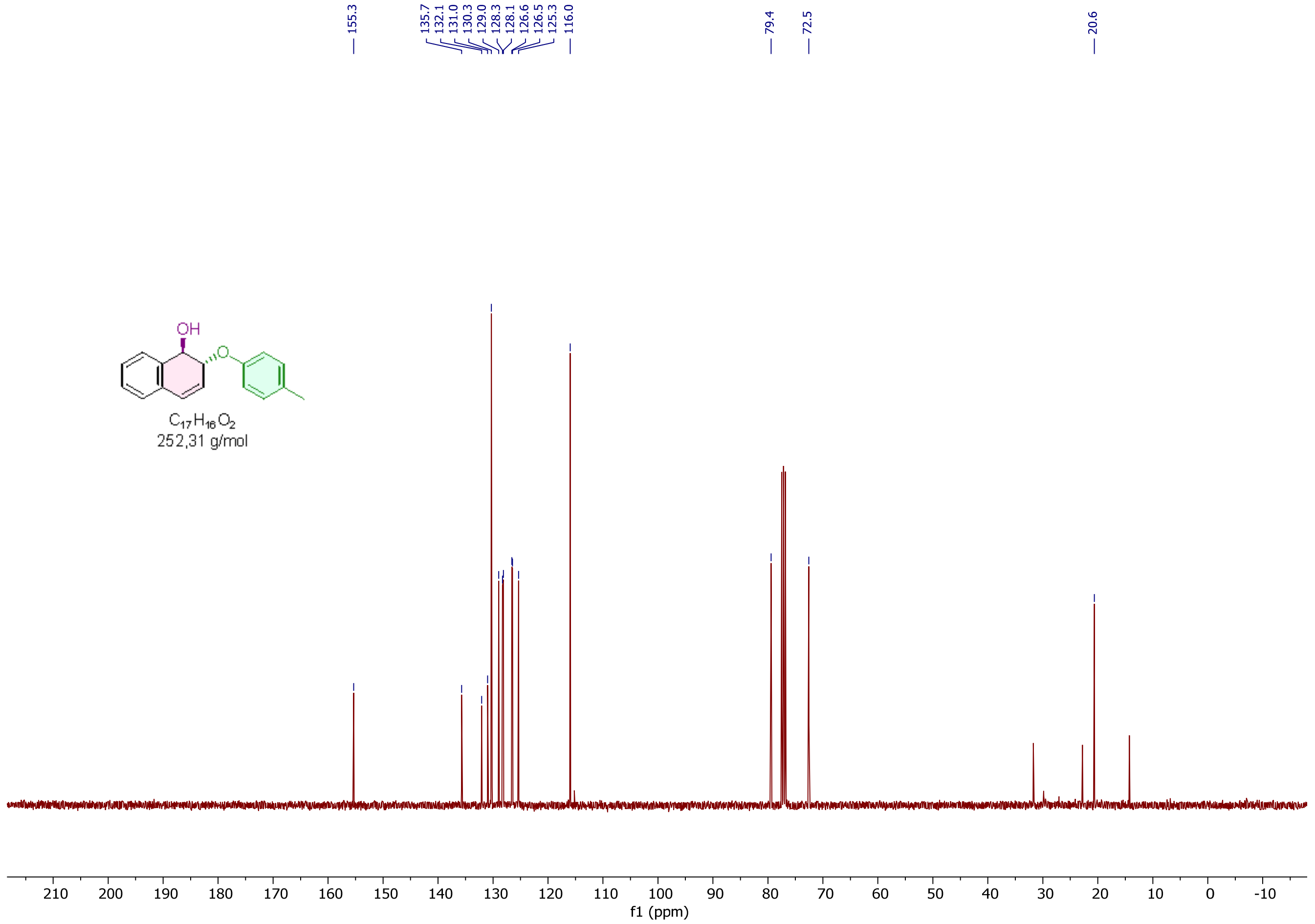

^{1}H NMR (400 MHz, $CDCl_3$) and ^{13}C{^{1}H} NMR (101 MHz, $CDCl_3$) Analysis of Compound **7na**

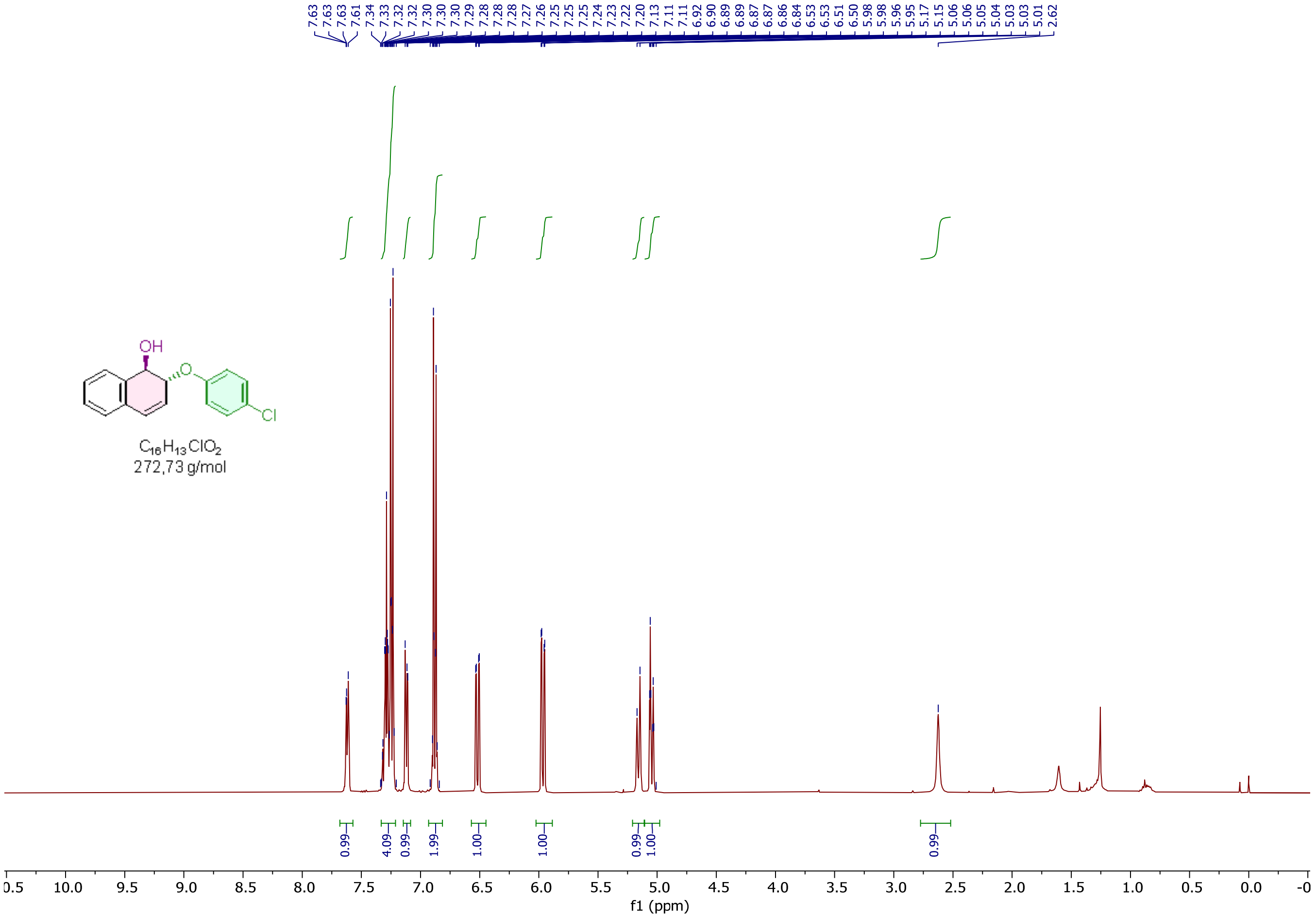

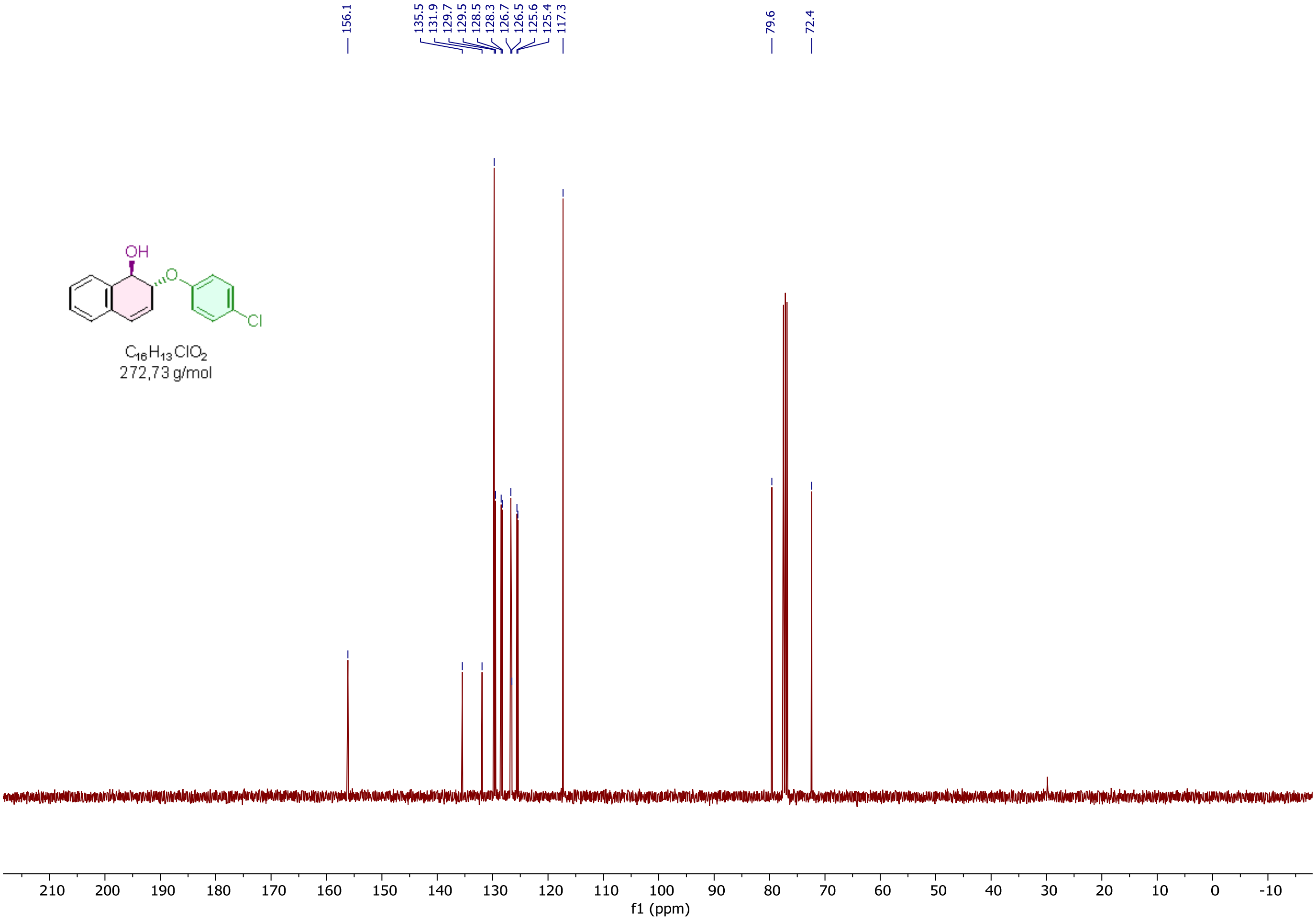

^{1}H NMR (400 MHz, $CDCl_3$) and $^{13}C\{^1H\}$ NMR (101 MHz, $CDCl_3$) Analysis of Compound **7nb**

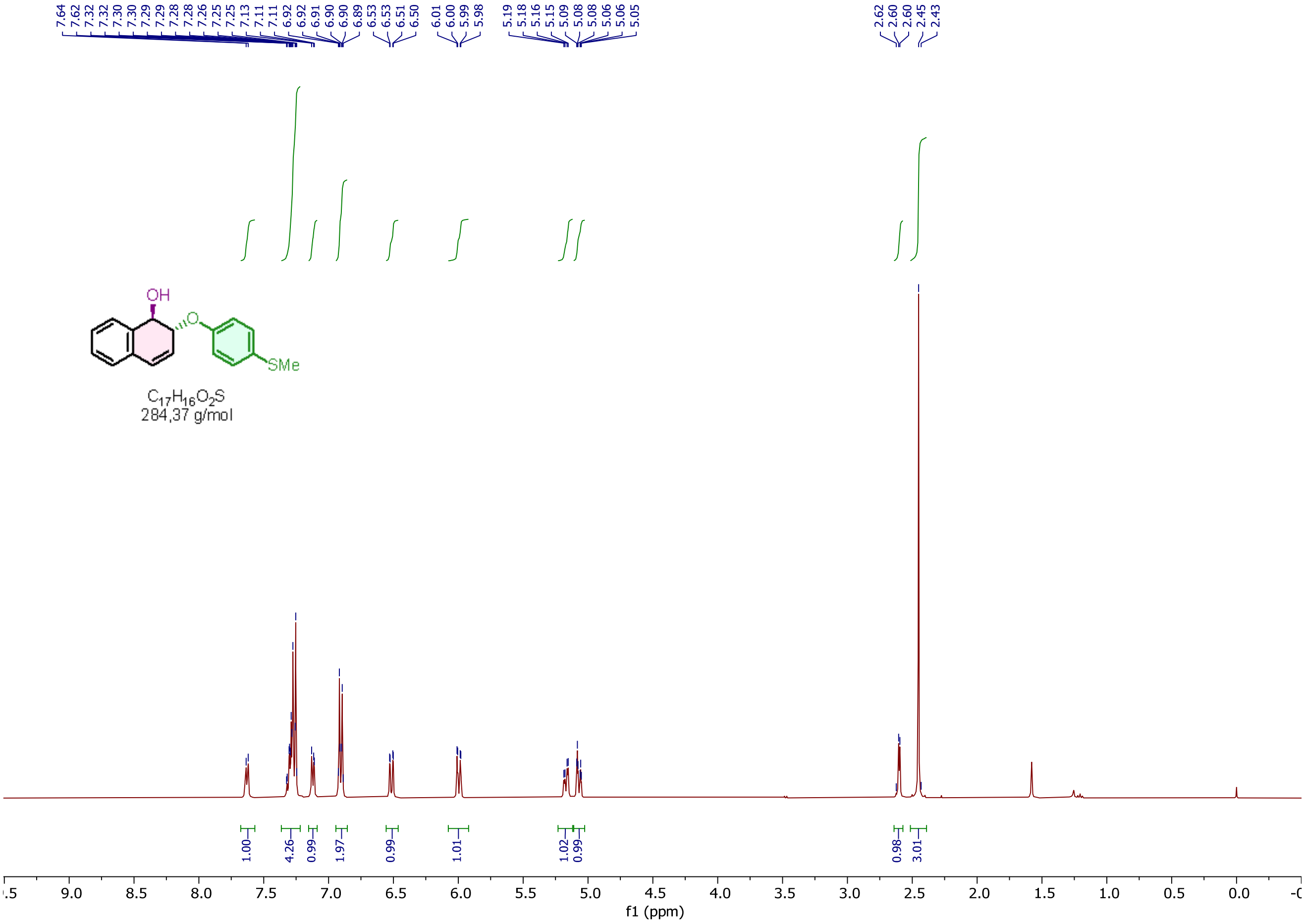

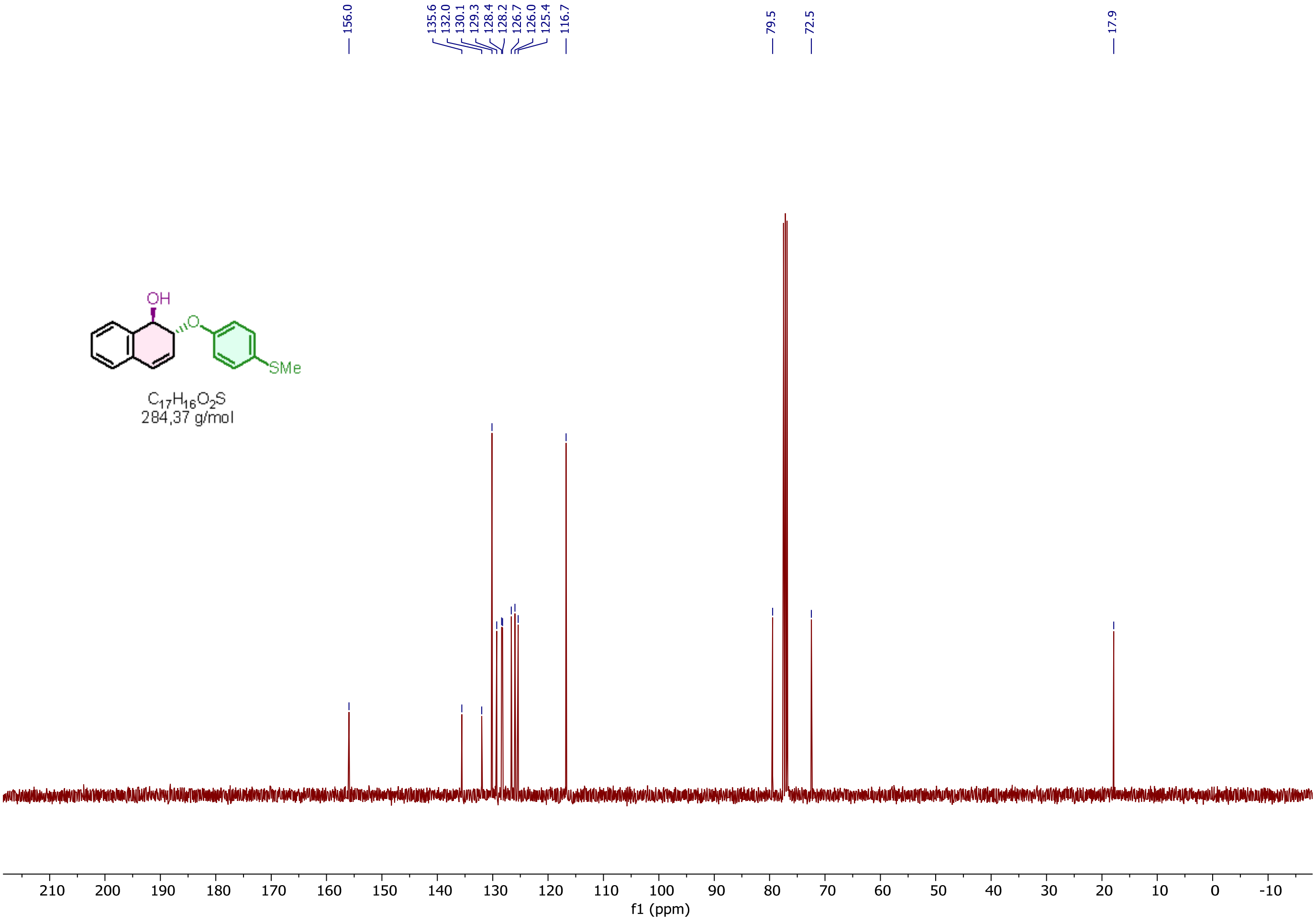

^{1}H NMR (400 MHz, $CDCl_3$) and $^{13}C\{^{1}H\}$ NMR (101 MHz, $CDCl_3$) Analysis of Compound **7nc**

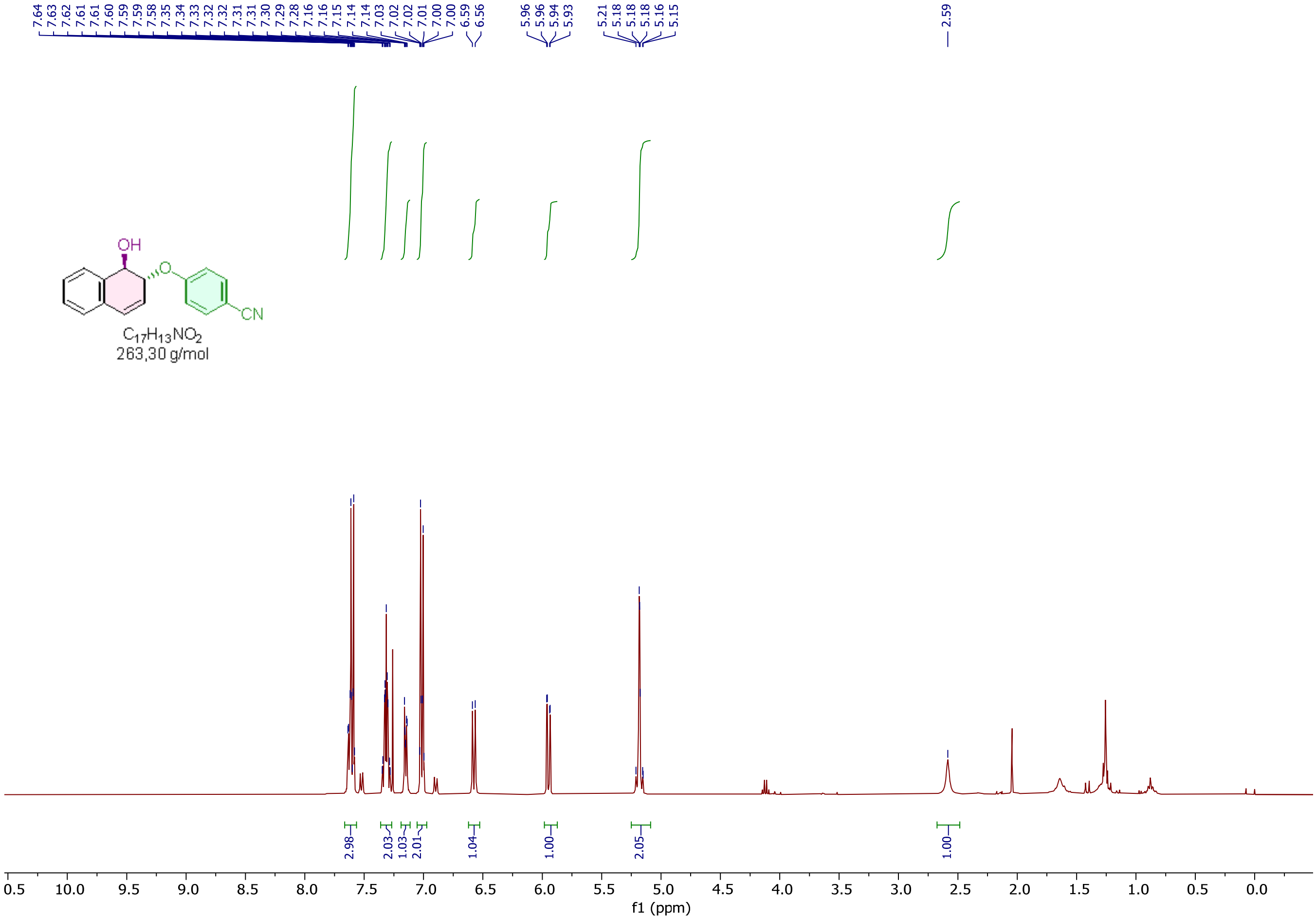

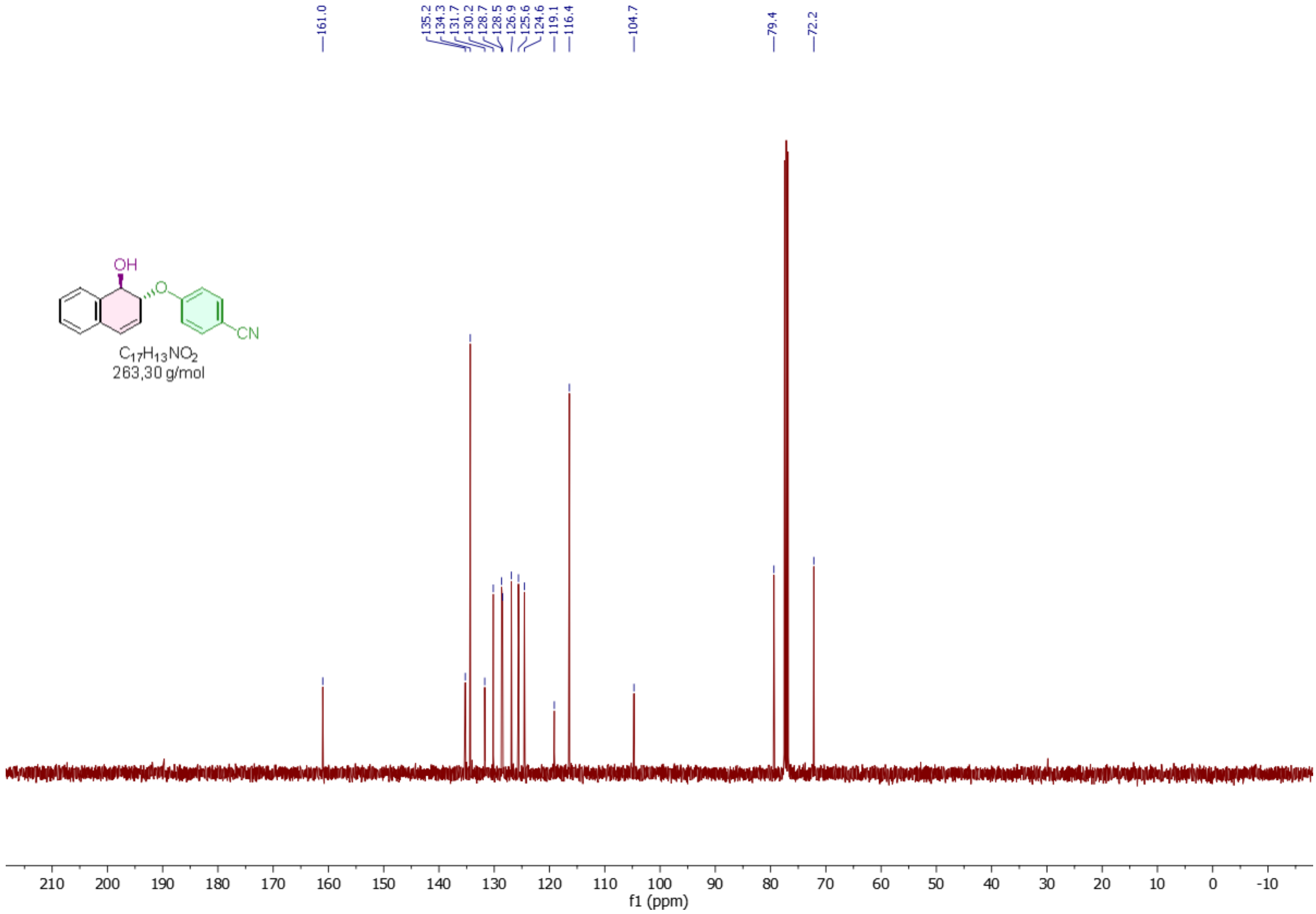

^{1}H NMR (400 MHz, $CDCl_3$) and $^{13}C\{^{1}H\}$ NMR (101 MHz, $CDCl_3$) Analysis of Compound **7nd**

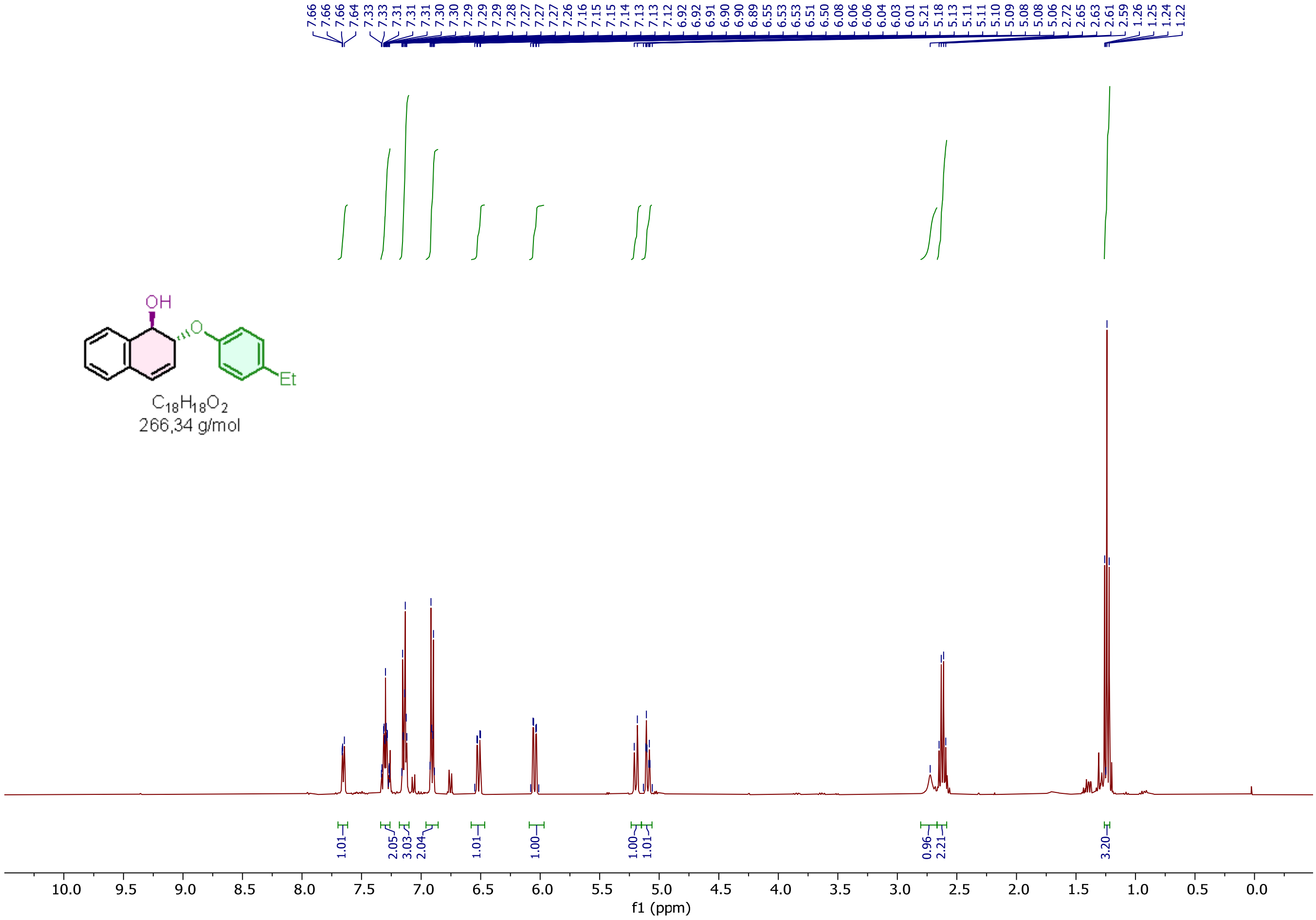

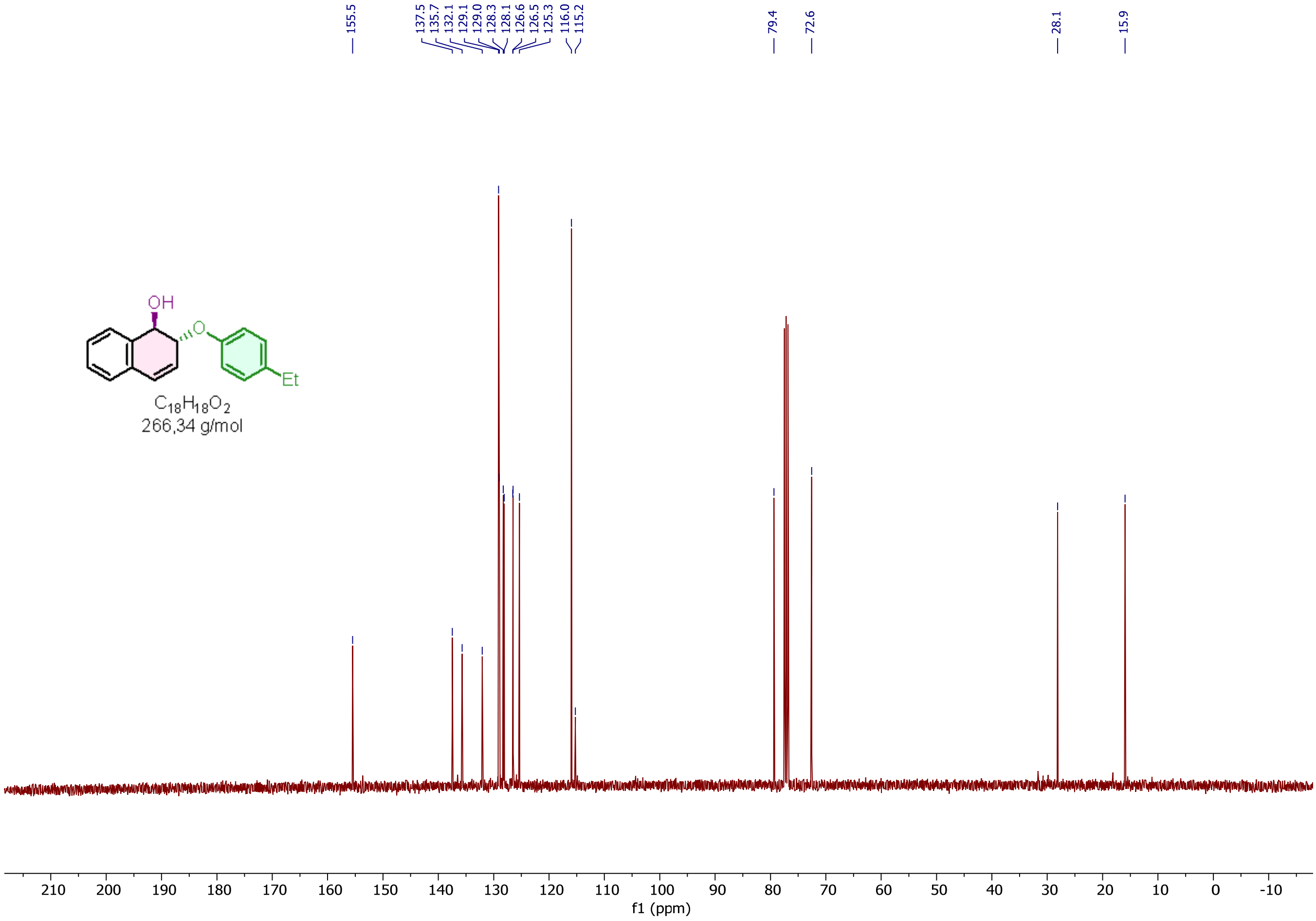

^{1}H NMR (400 MHz, $CDCl_3$) and ^{13}C{^{1}H} NMR (101 MHz, $CDCl_3$) Analysis of Compound **7ne**

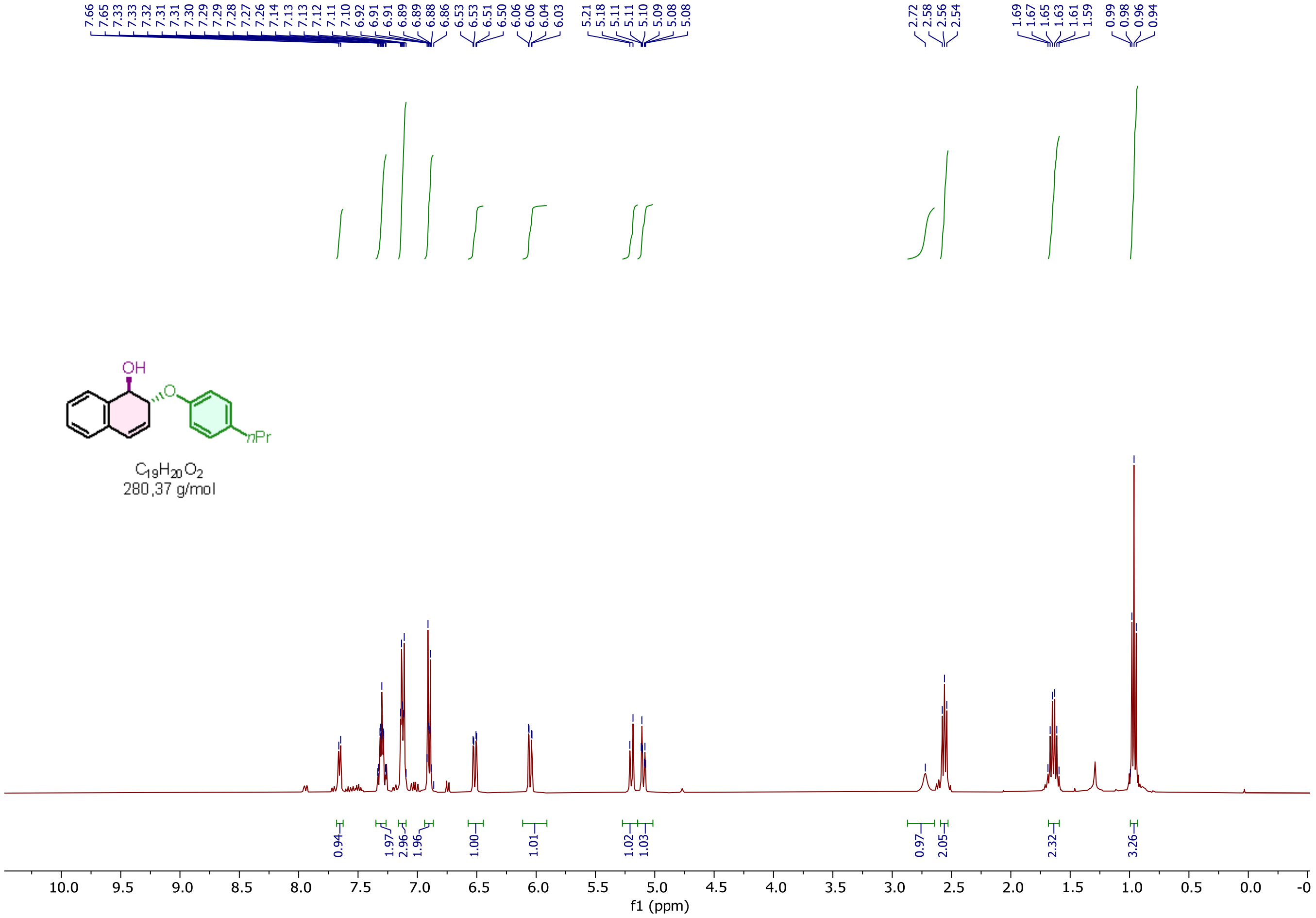

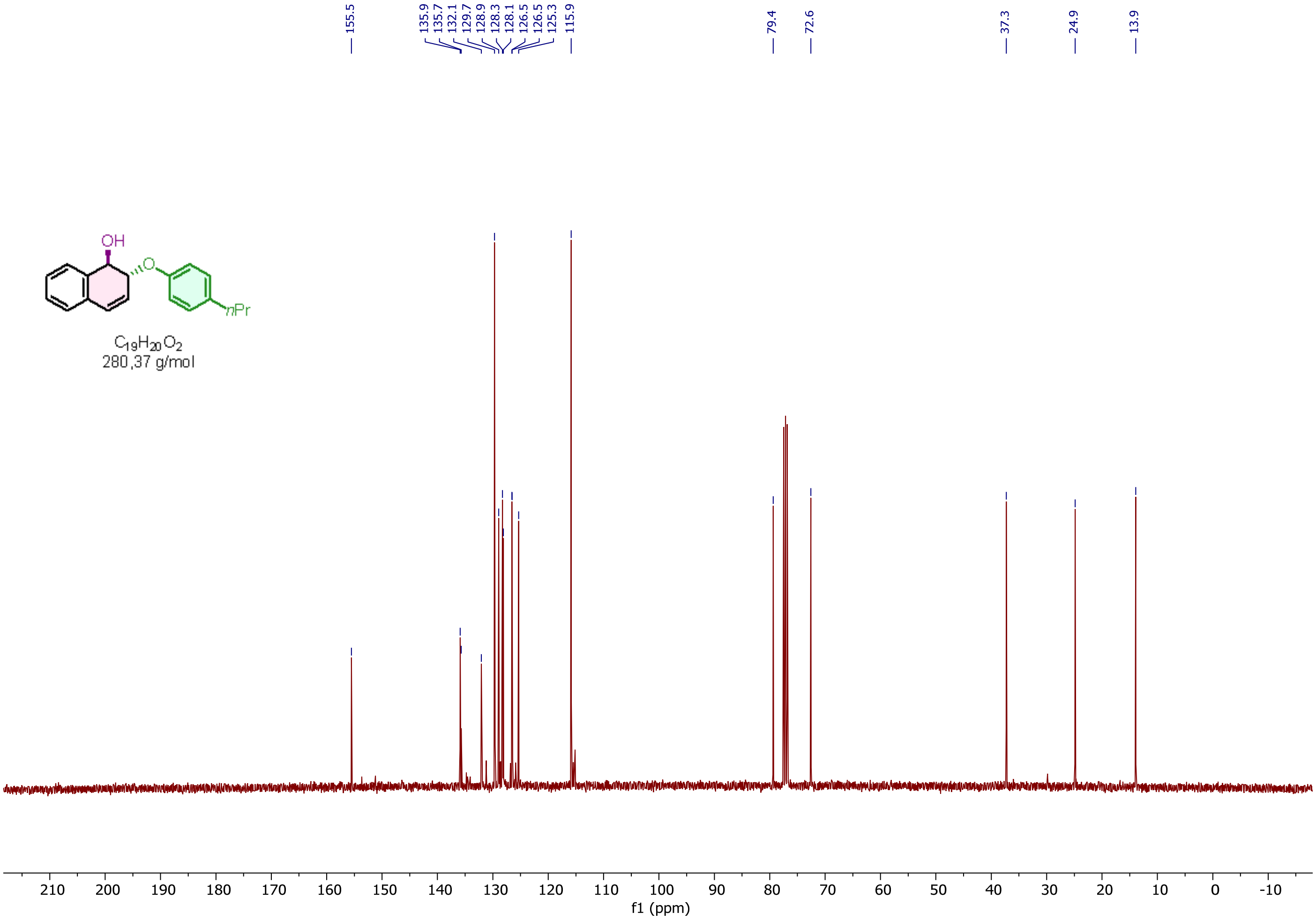

^{1}H NMR (400 MHz, $CDCl_3$) and ^{13}C{^{1}H} NMR (101 MHz, $CDCl_3$) Analysis of Compound **7nf**

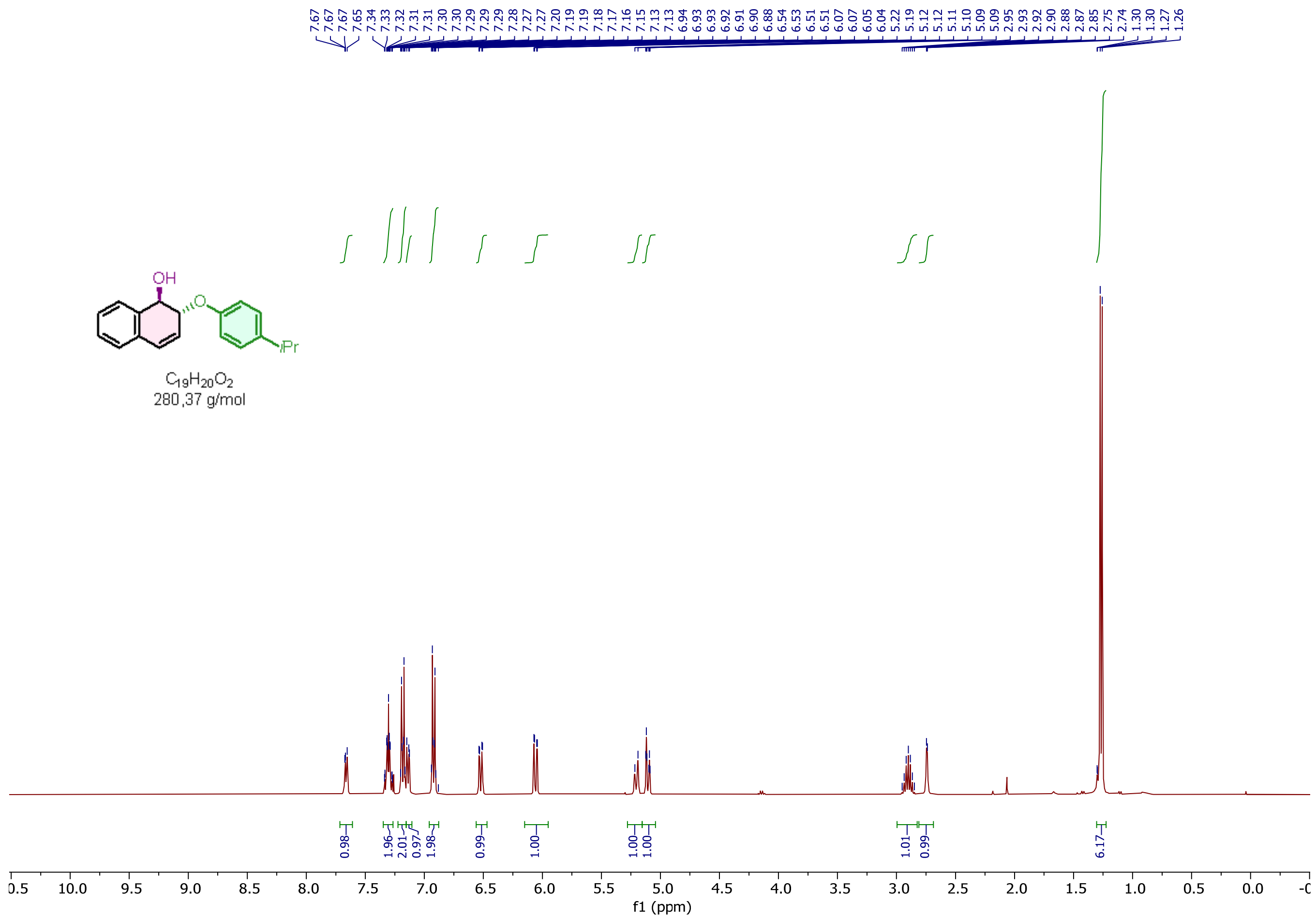

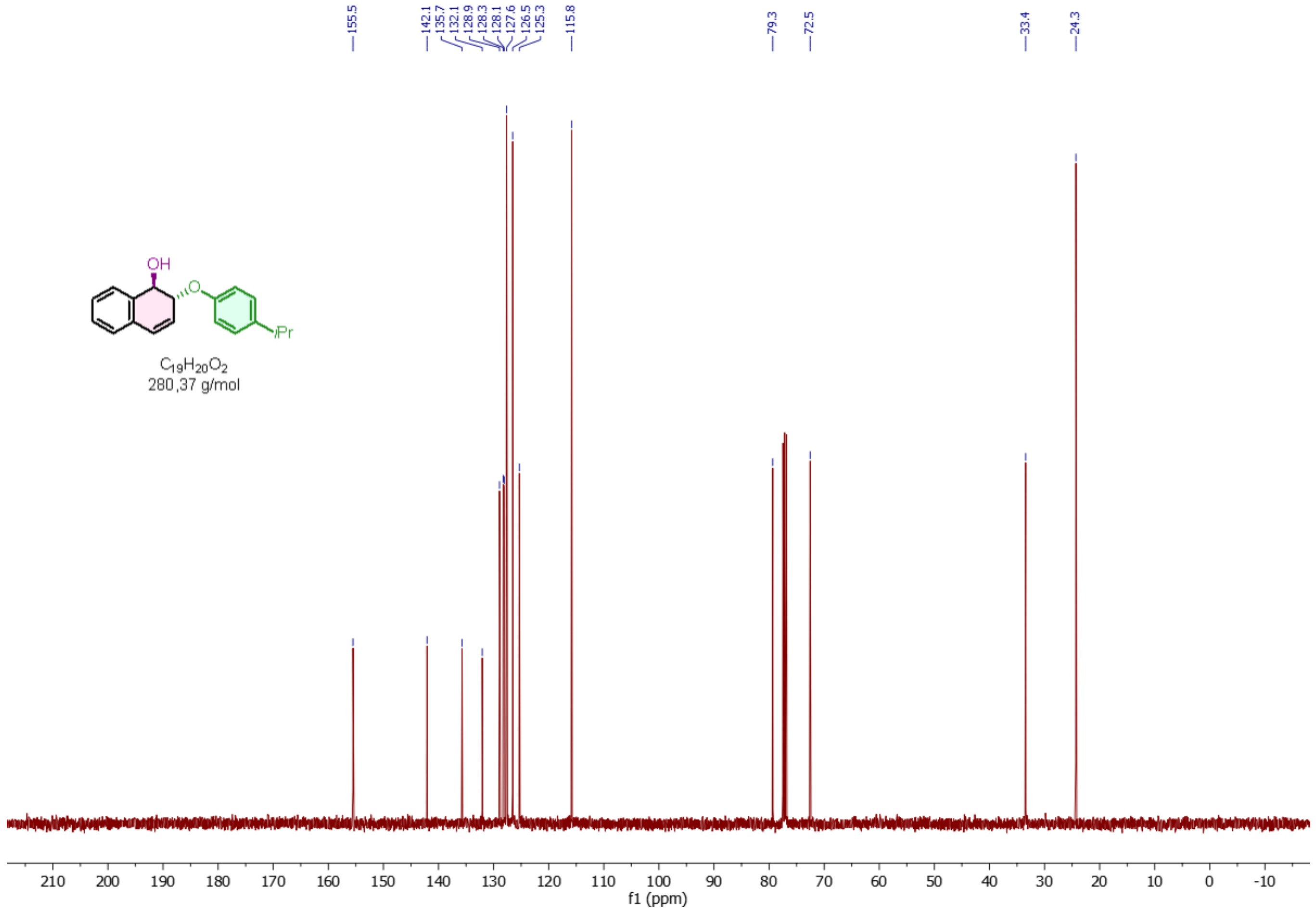

^{1}H NMR (400 MHz, $CDCl_3$) and $^{13}C\{^{1}H\}$ NMR (101 MHz, $CDCl_3$) Analysis of Compound **7ng**

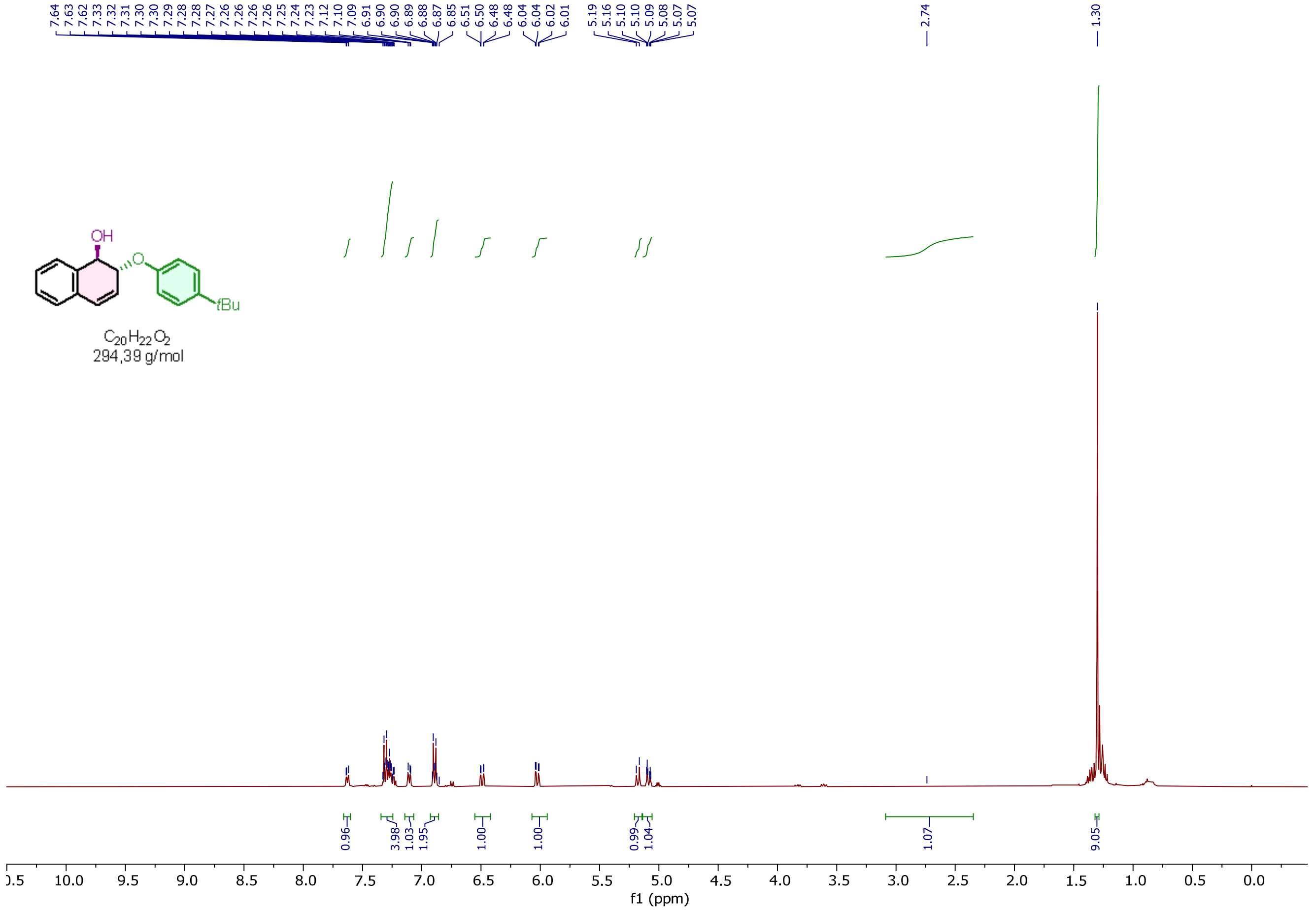

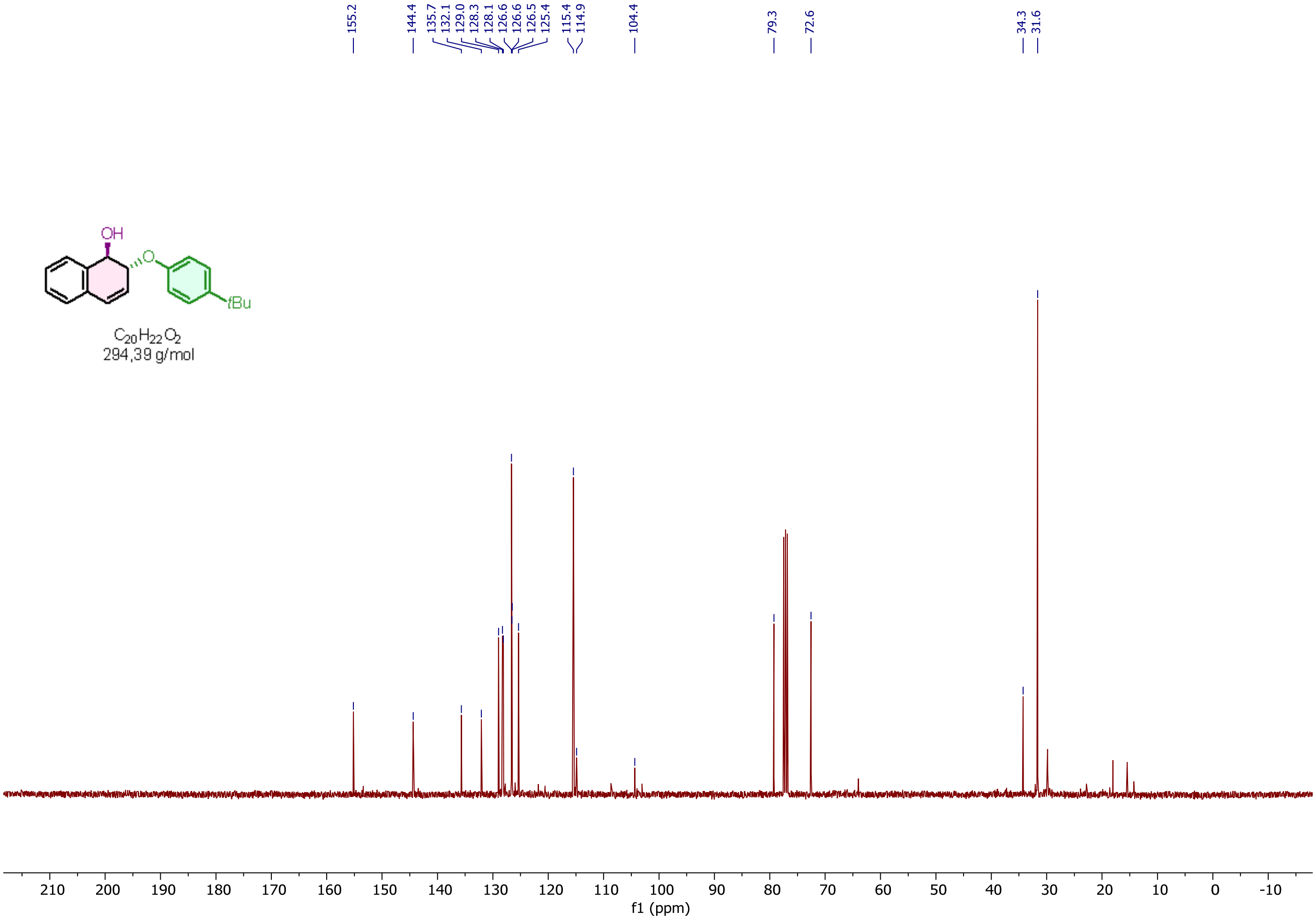

^1H NMR (400 MHz, $CDCl_3$) and $^{13}C\{^1H\}$ NMR (101 MHz, $CDCl_3$) Analysis of Compound **7nh**

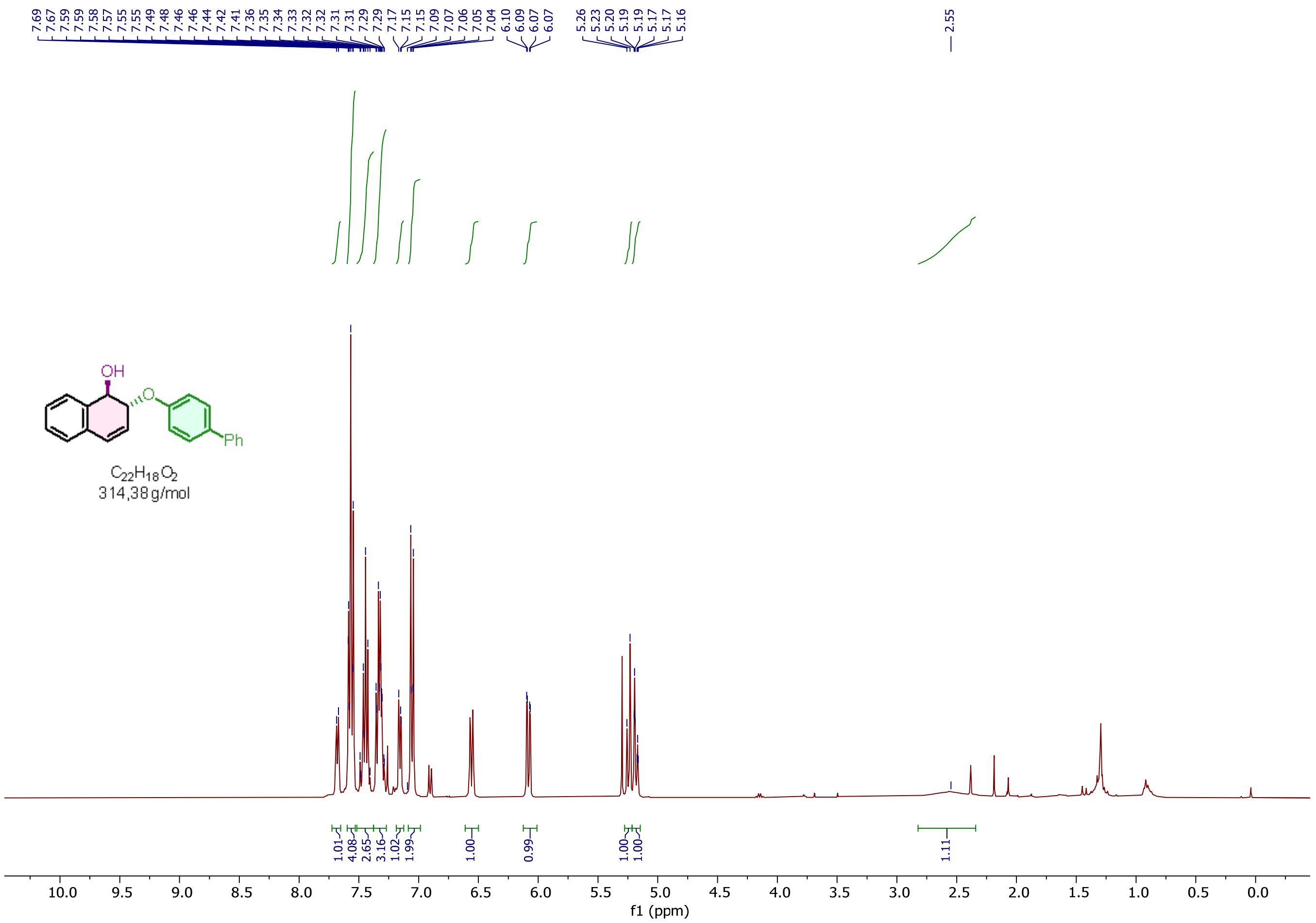

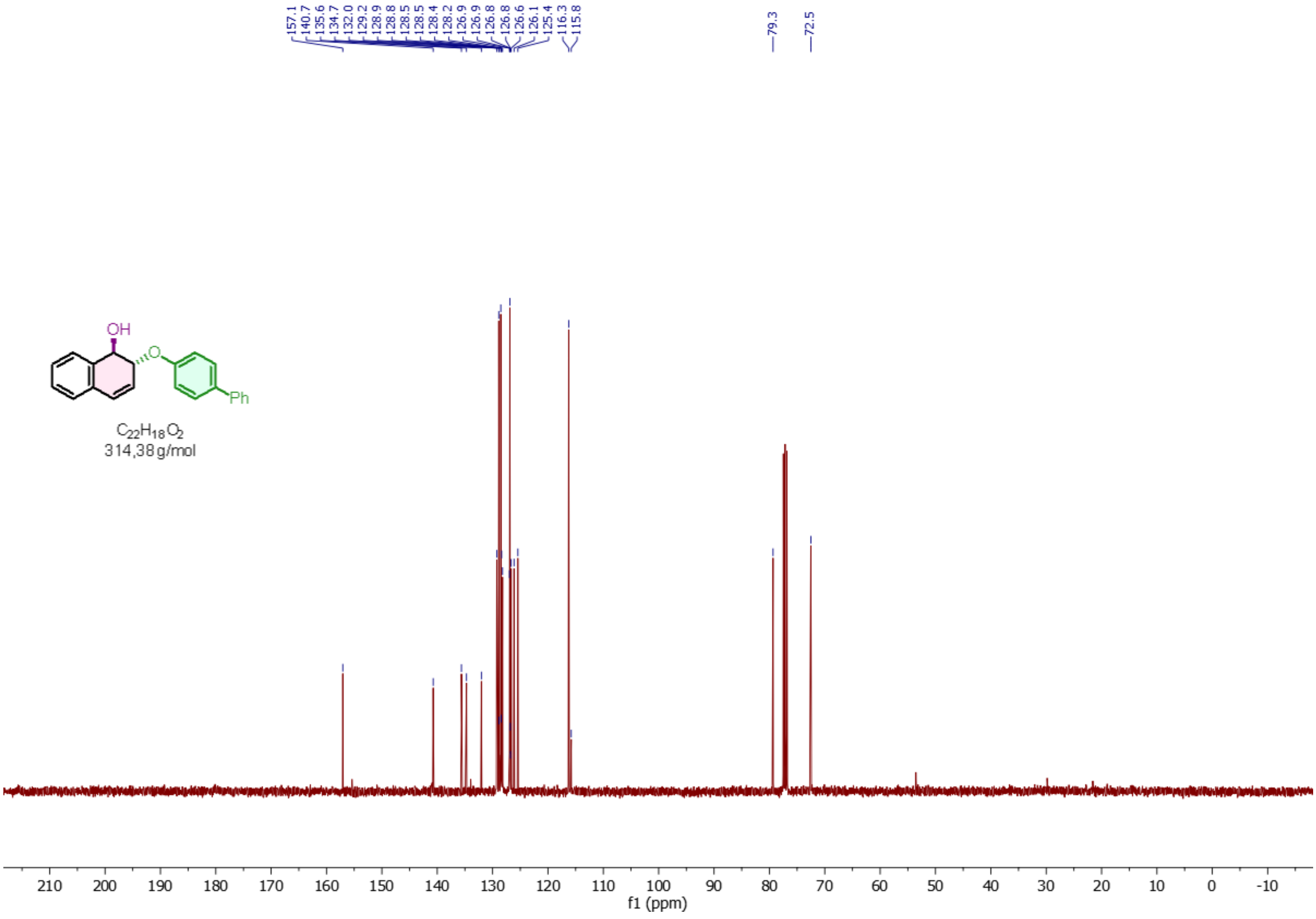

^{1}H NMR (400 MHz, $CDCl_3$) and $^{13}C\{^{1}H\}$ NMR (101 MHz, $CDCl_3$) Analysis of Compound **7ni**

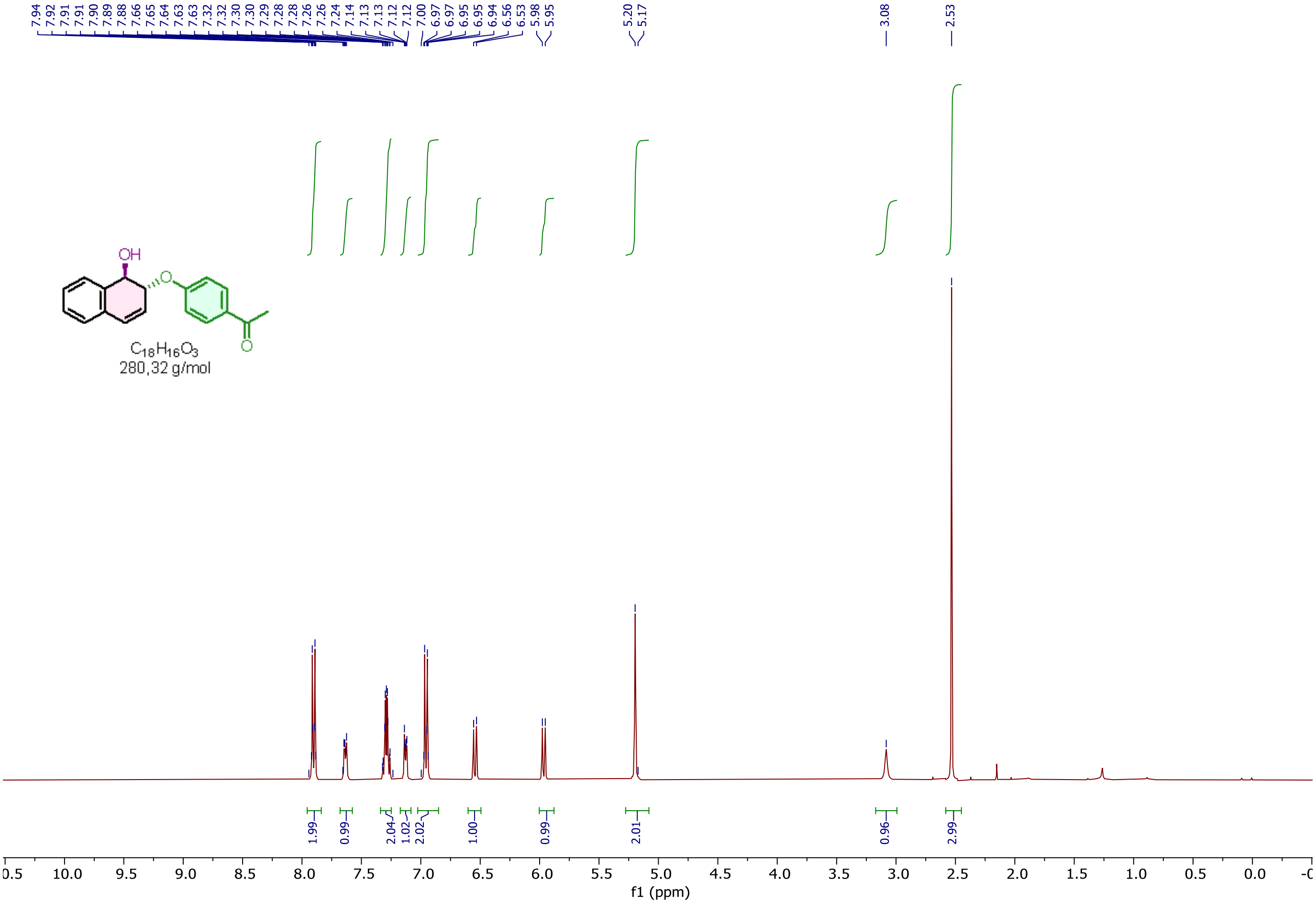

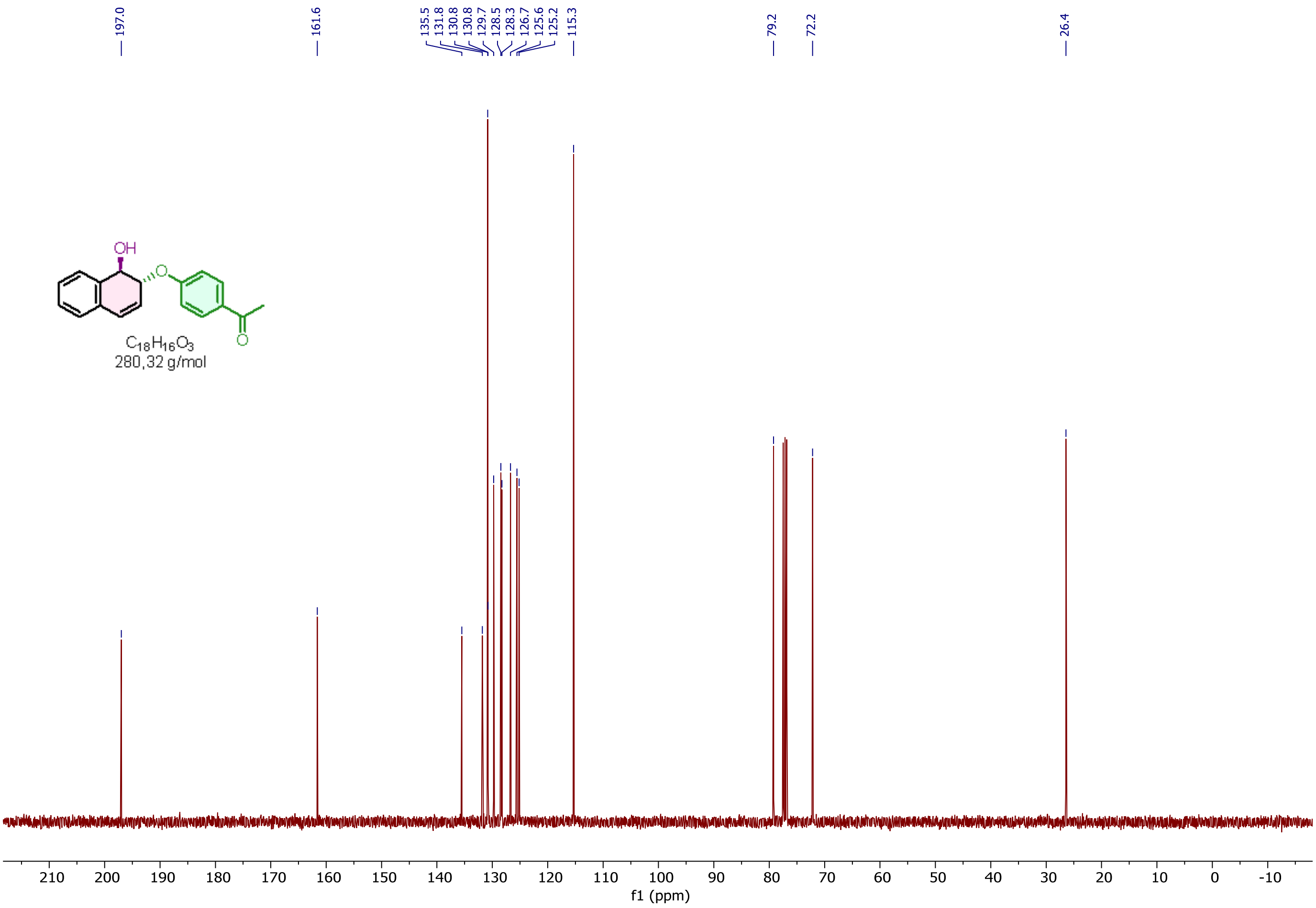

^{1}H NMR (400 MHz, MeOD) and ^{13}C{^{1}H} NMR (101 MHz, MeOD) Analysis of Compound **7nj**

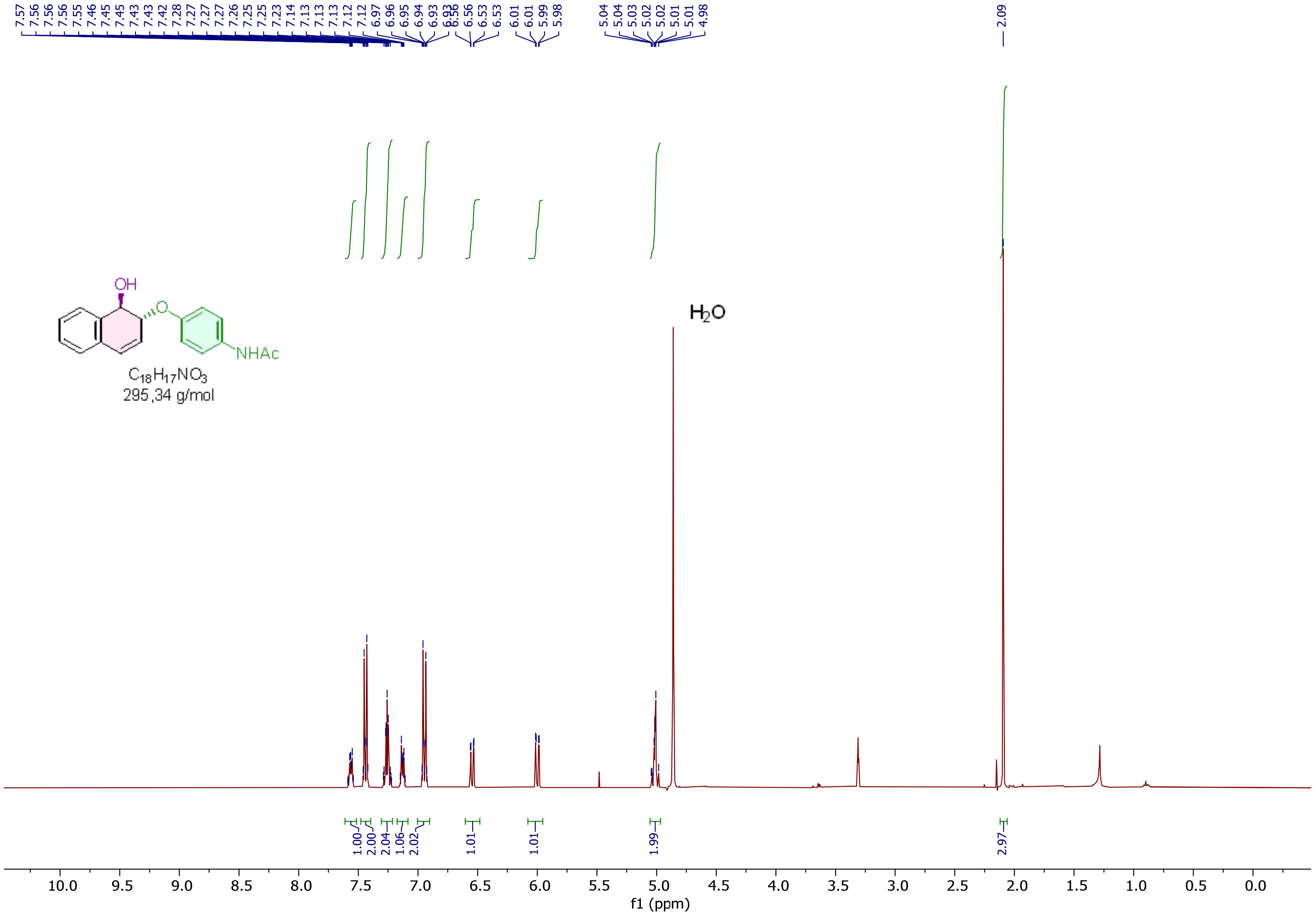

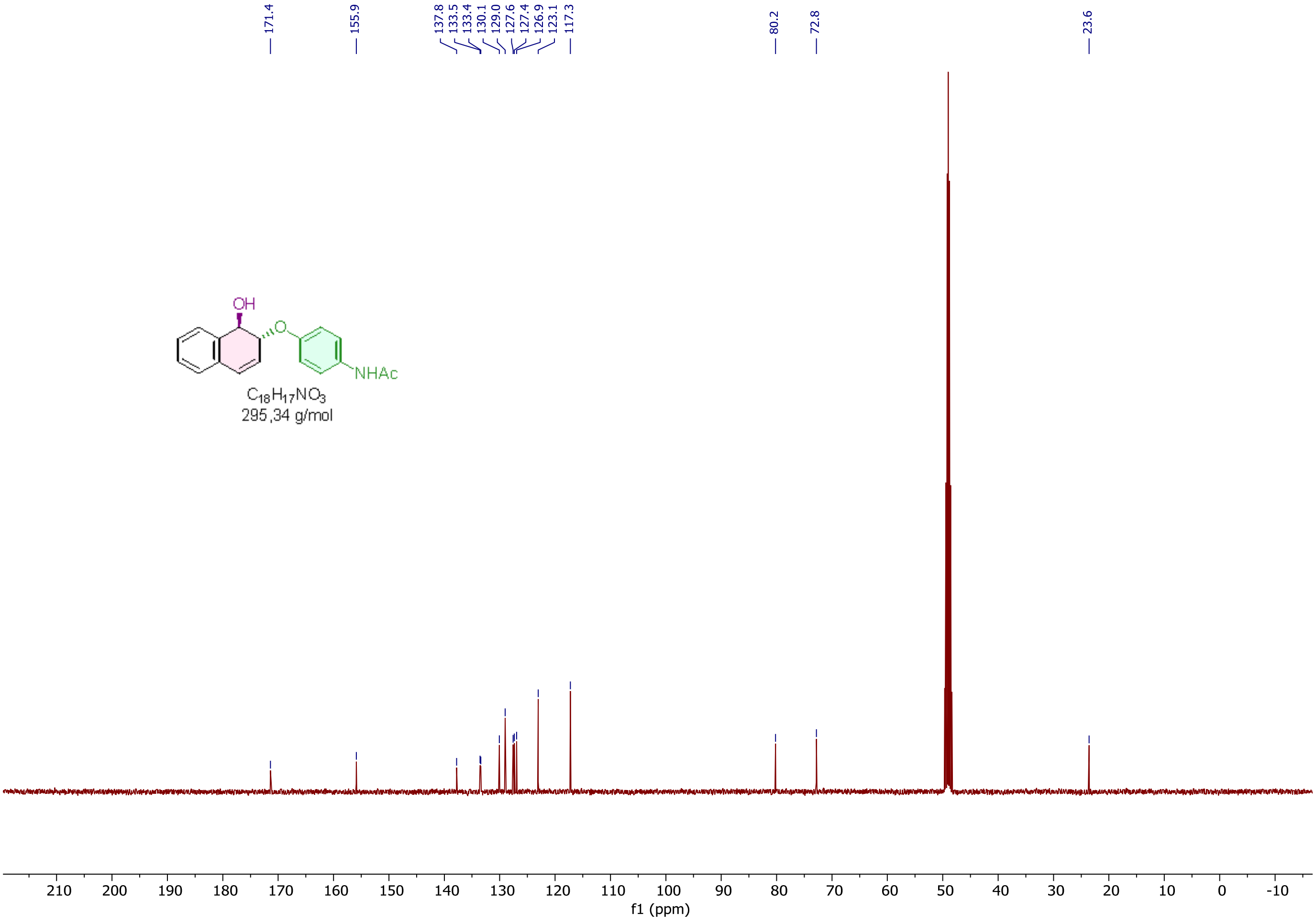

^{1}H NMR (400 MHz, $CDCl_3$) and $^{13}C\{^{1}H\}$ NMR (101 MHz, $CDCl_3$) Analysis of Compound **7nk**

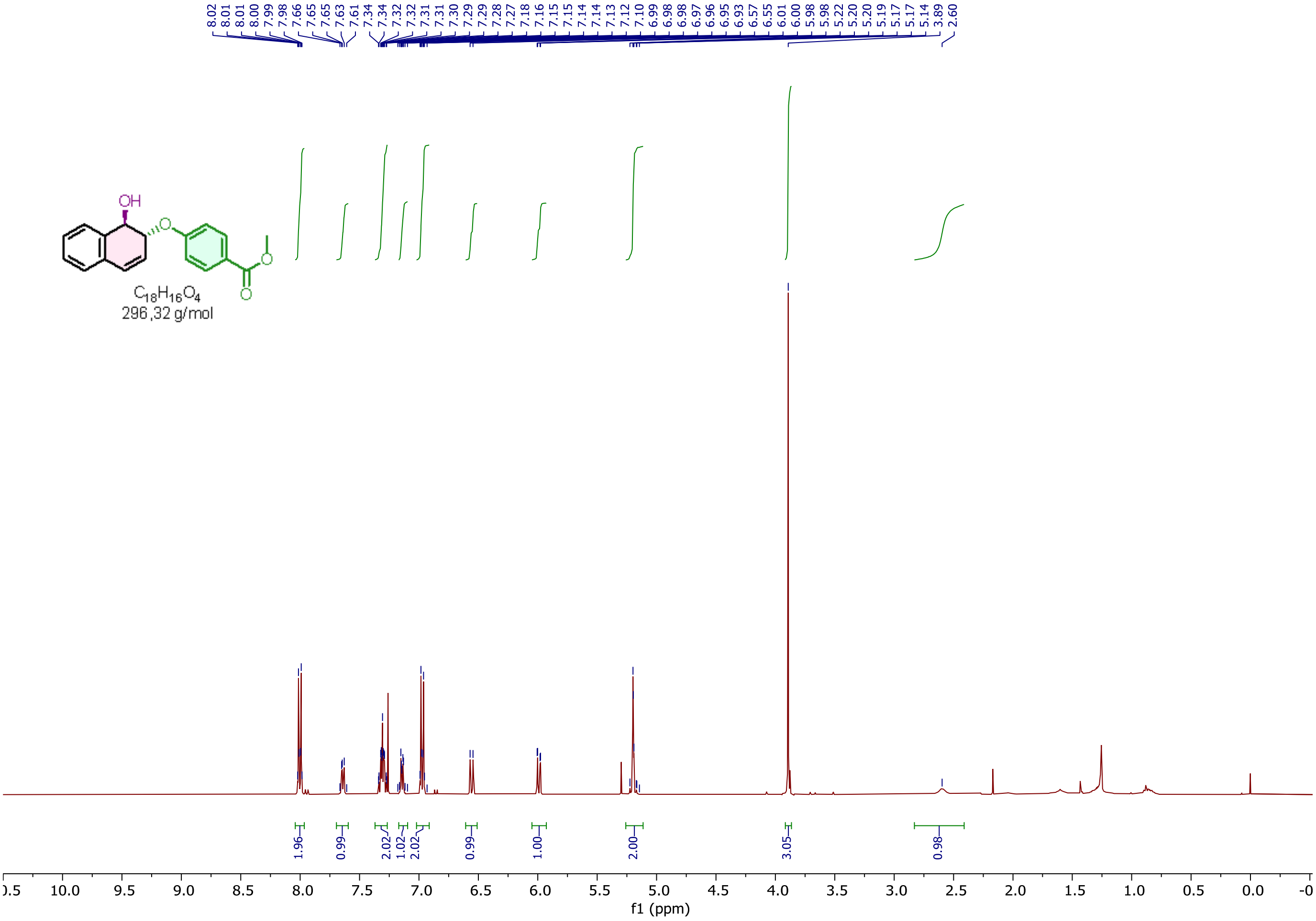

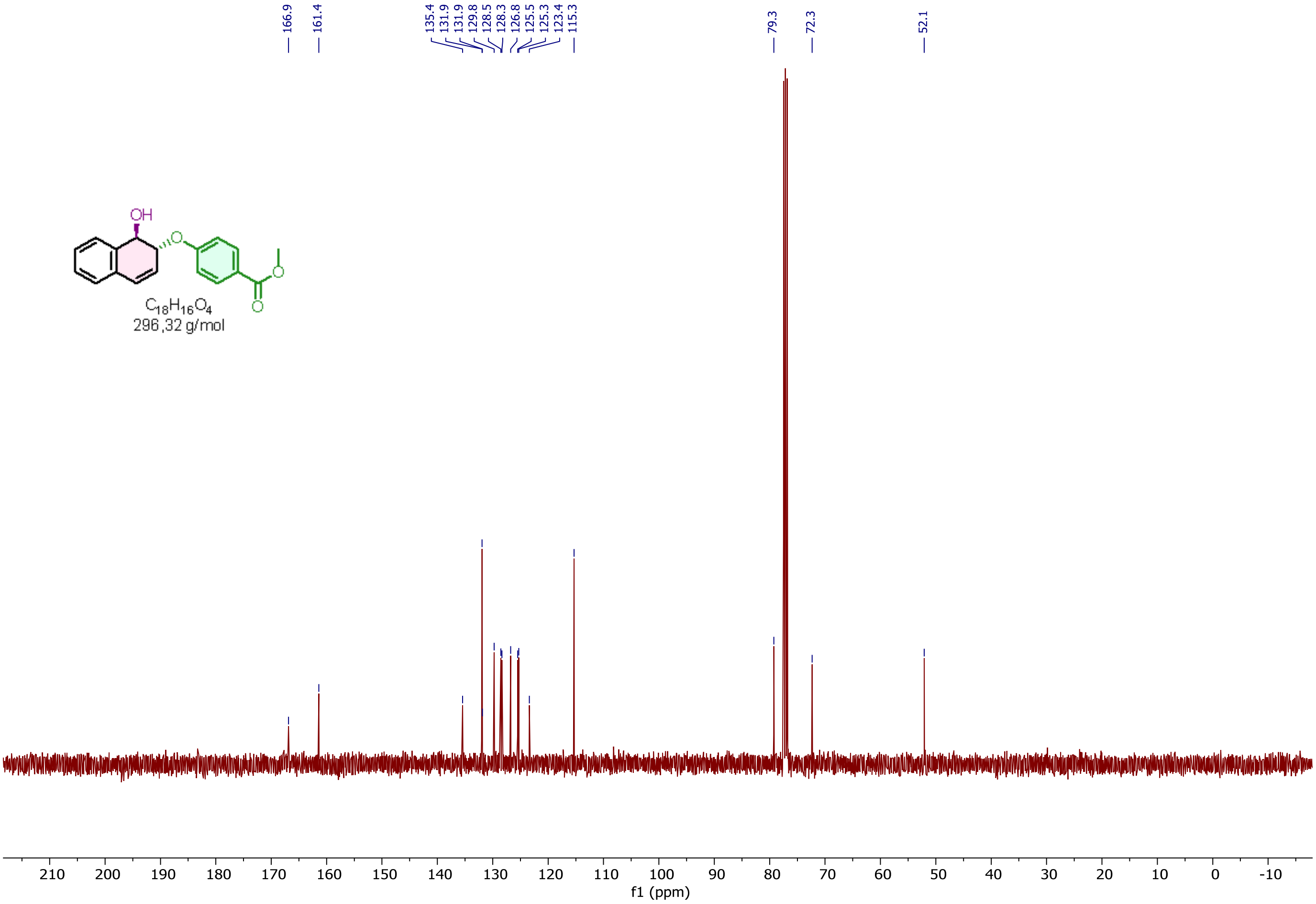

^{1}H NMR (400 MHz, $CDCl_3$) and ^{13}C{^{1}H} NMR (101 MHz, $CDCl_3$) Analysis of Compound **7nl**

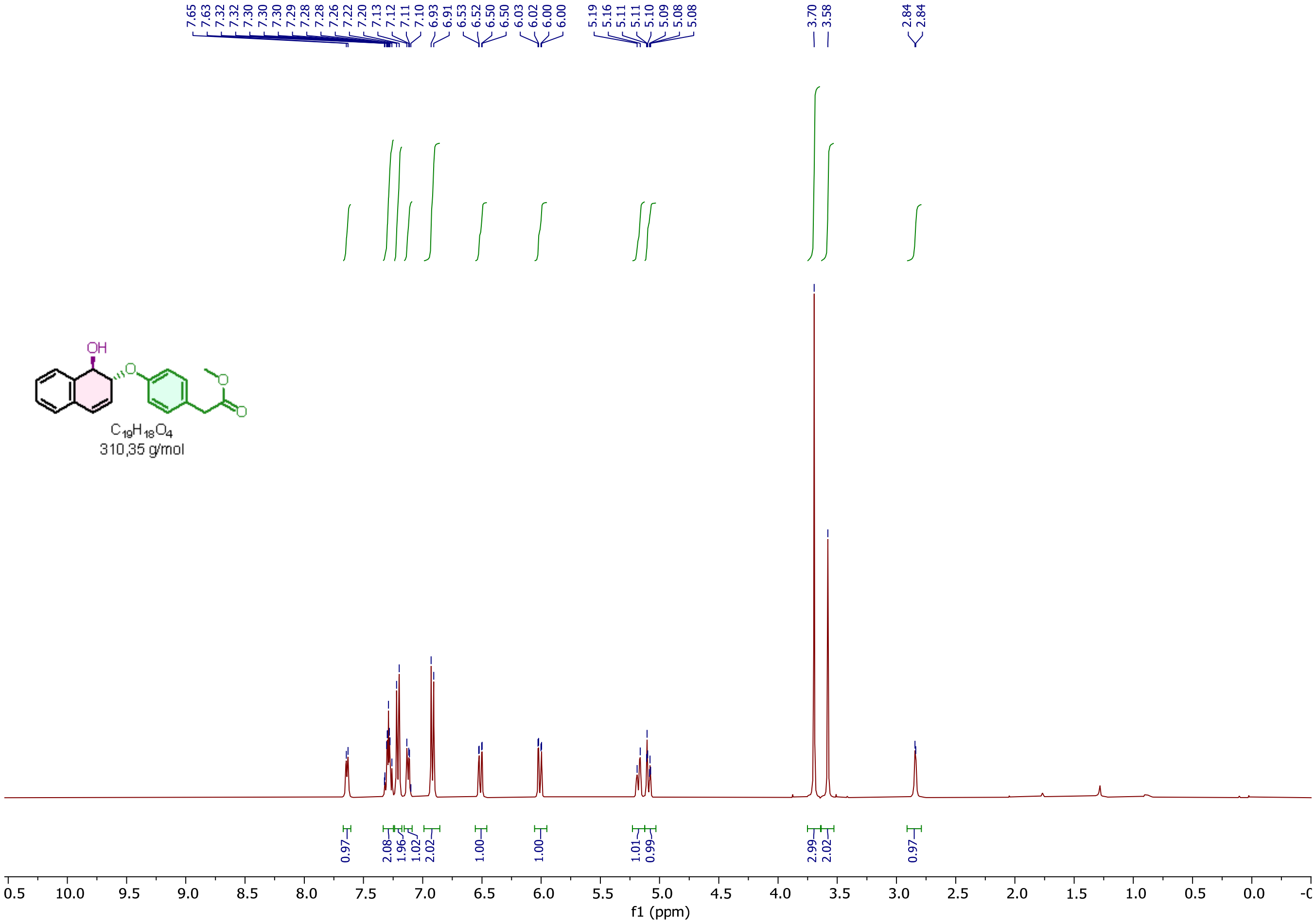

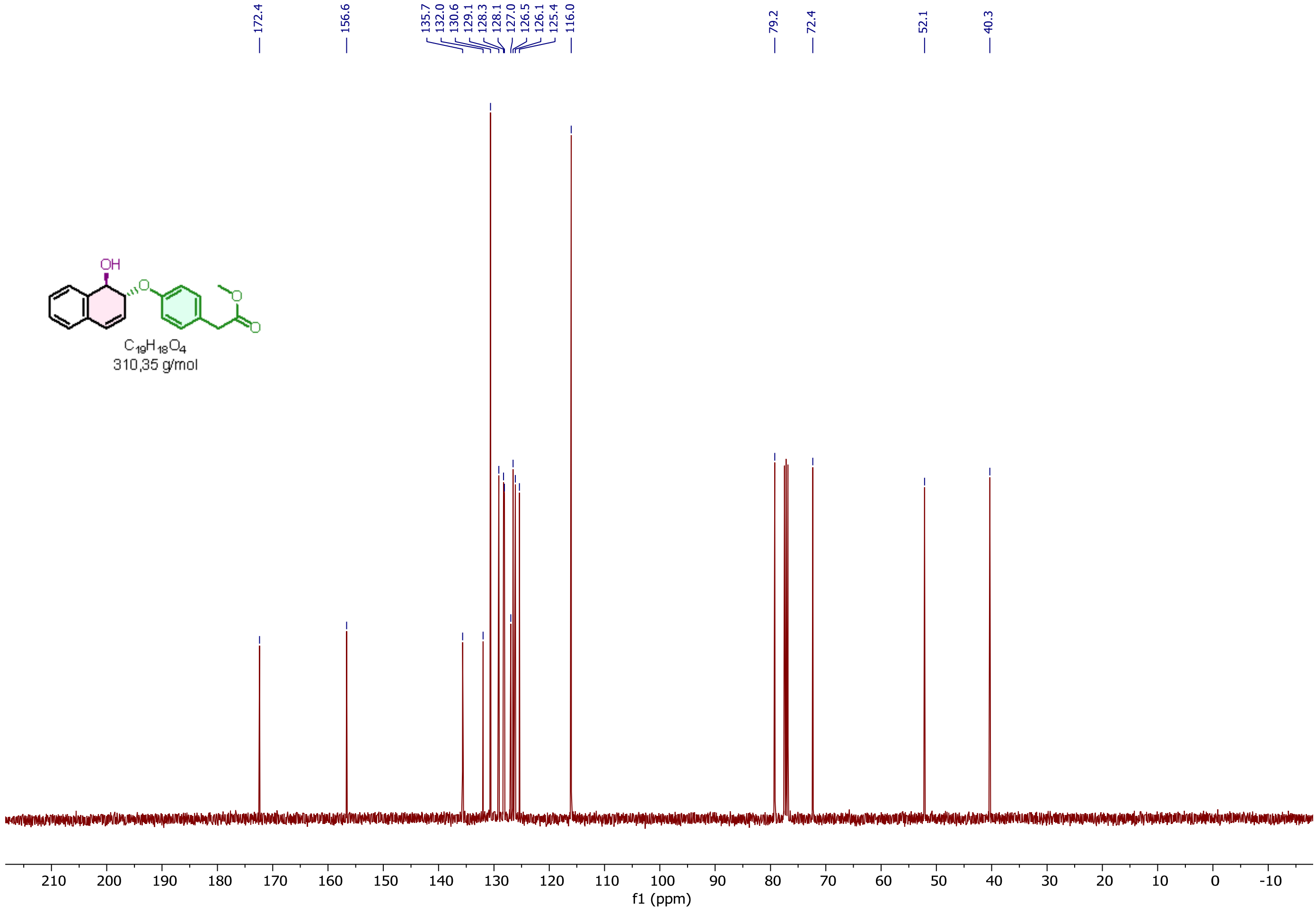

^{1}H NMR (400 MHz, $CDCl_3$) and ^{13}C{^{1}H} NMR (101 MHz, $CDCl_3$) Analysis of Compound **7o**

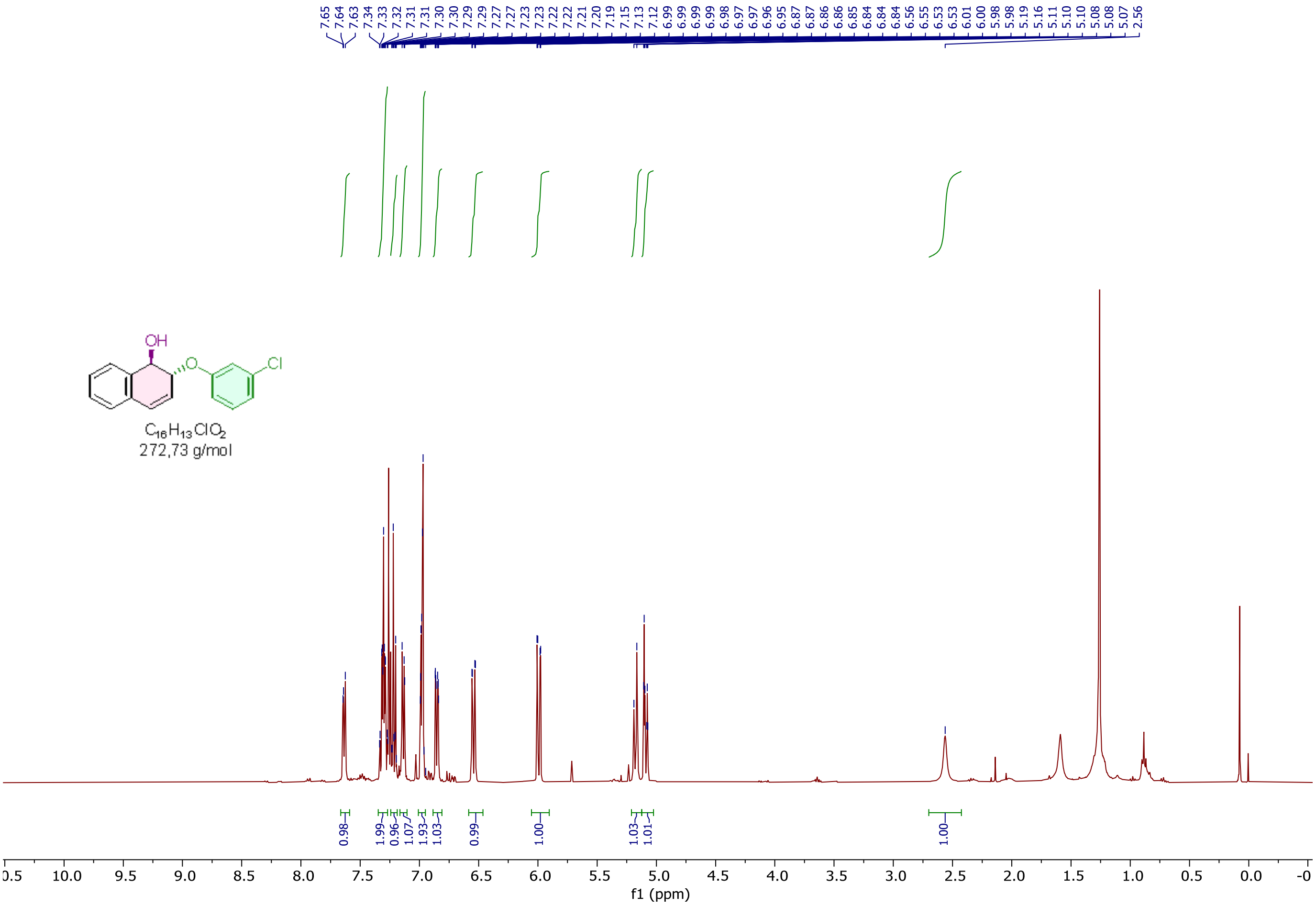

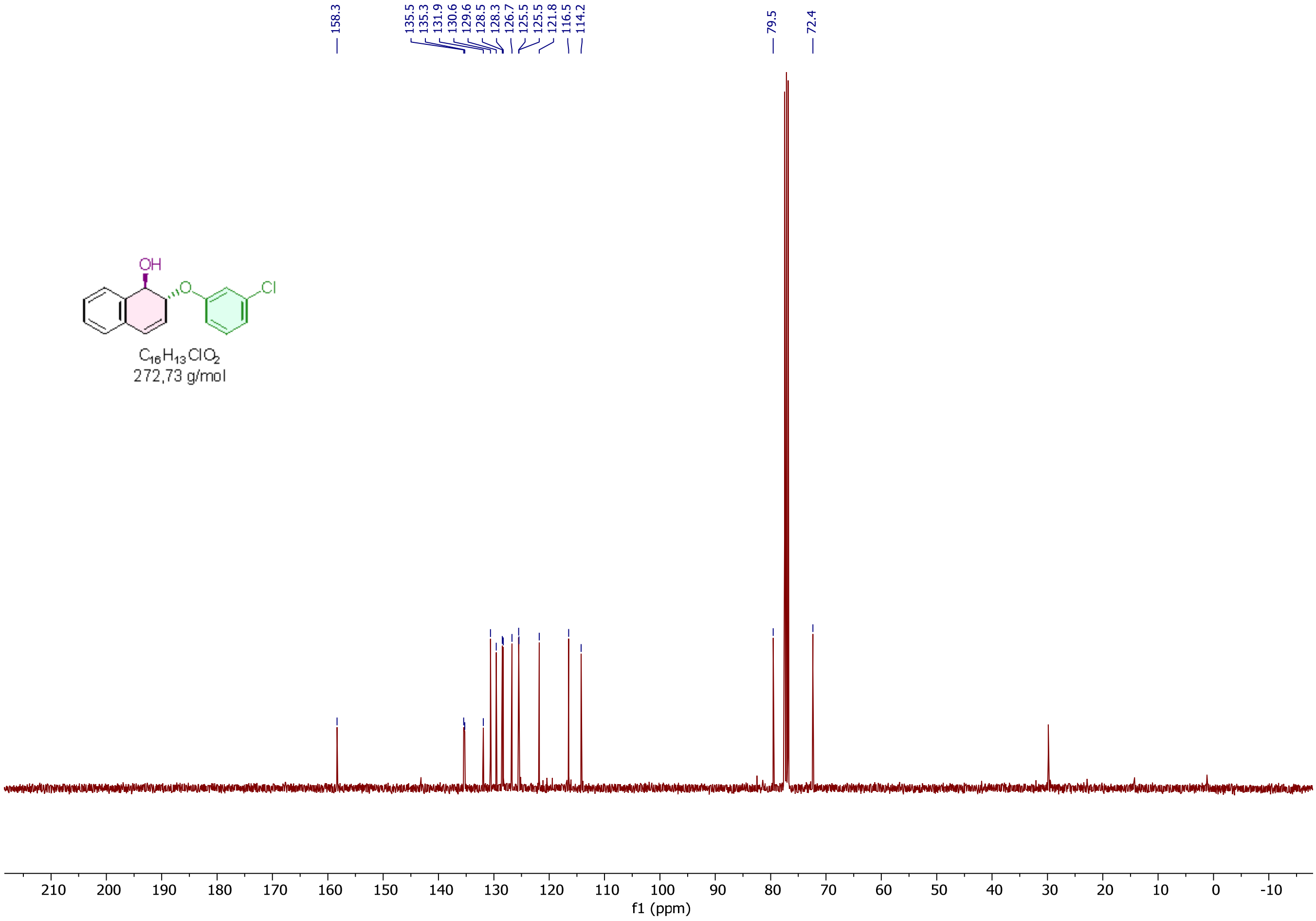

^1H NMR (400 MHz, $CDCl_3$) and $^{13}C\{^1H\}$ NMR (101 MHz, $CDCl_3$) Analysis of Compound **7p**

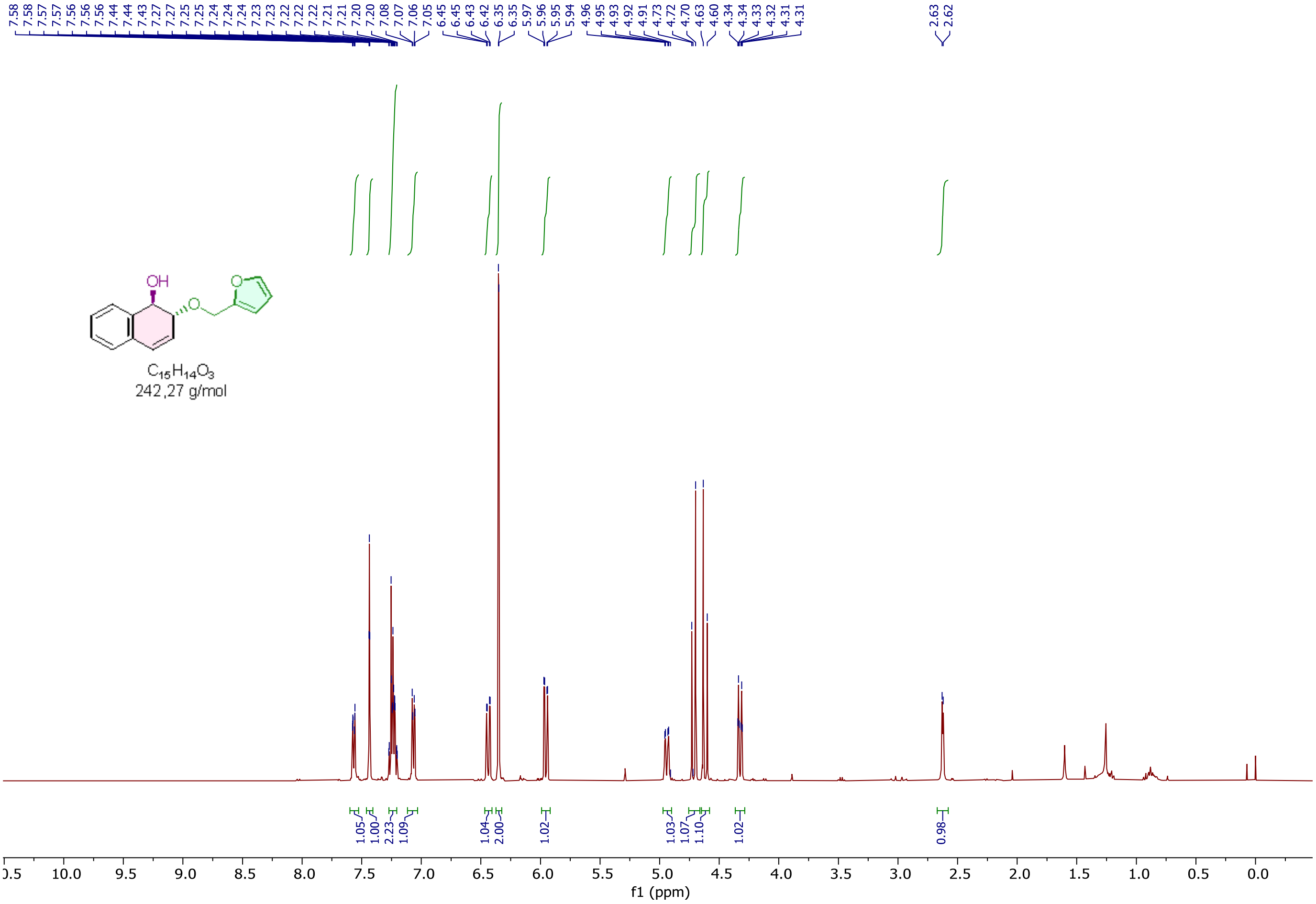

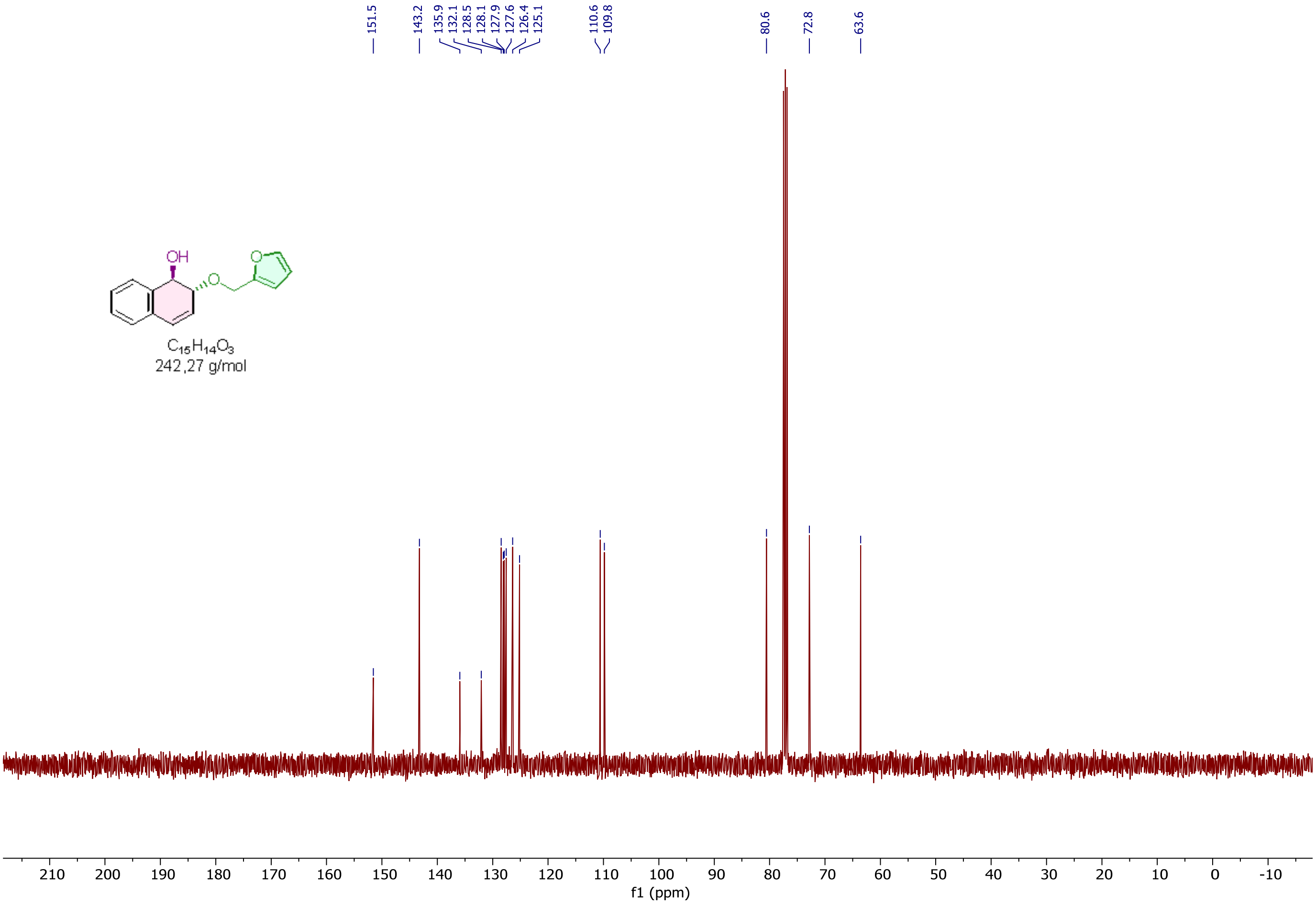

^{1}H NMR (400 MHz, $CDCl_3$) and $^{13}C\{^{1}H\}$ NMR (101 MHz, $CDCl_3$) Analysis of Compound **7r**

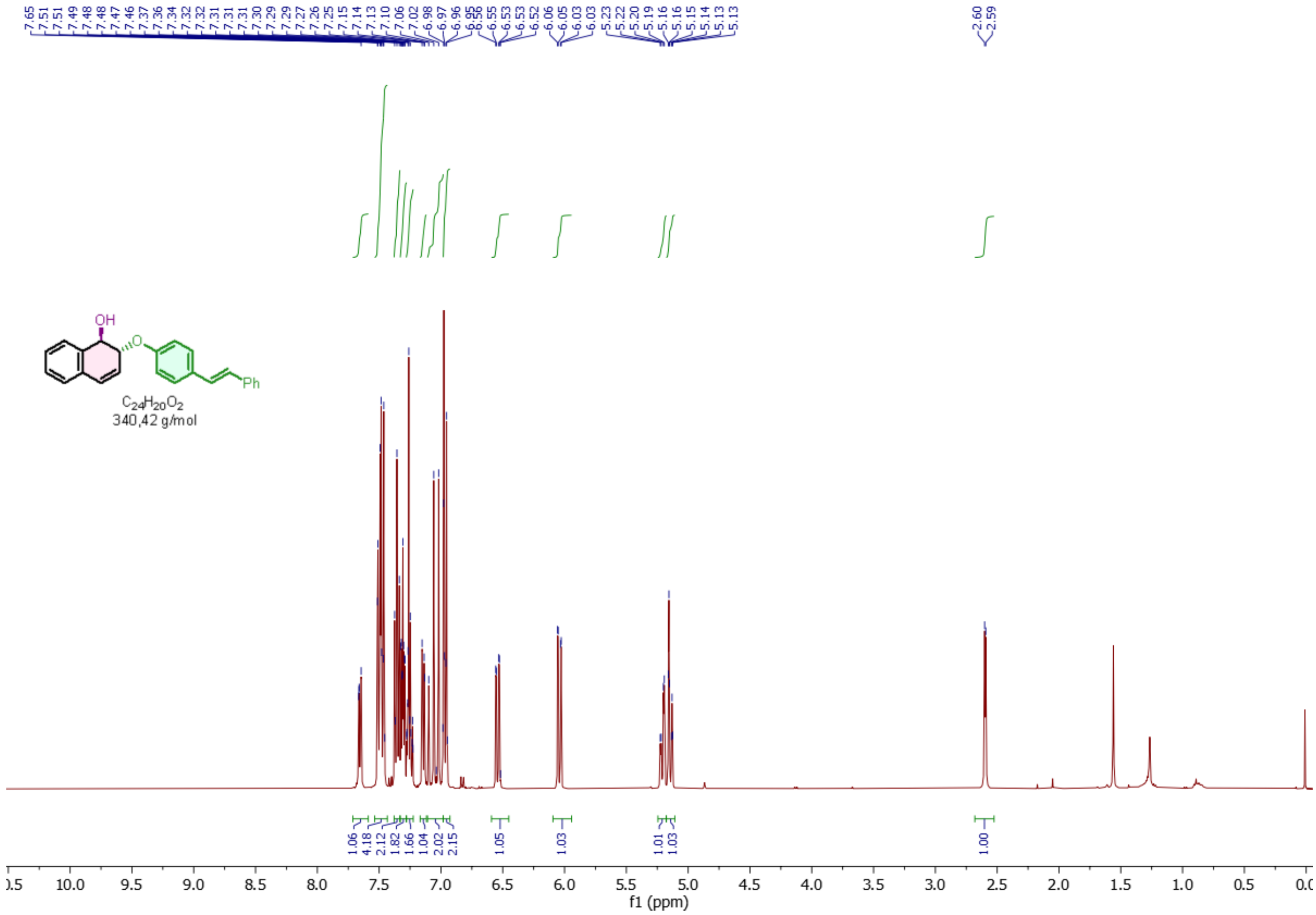

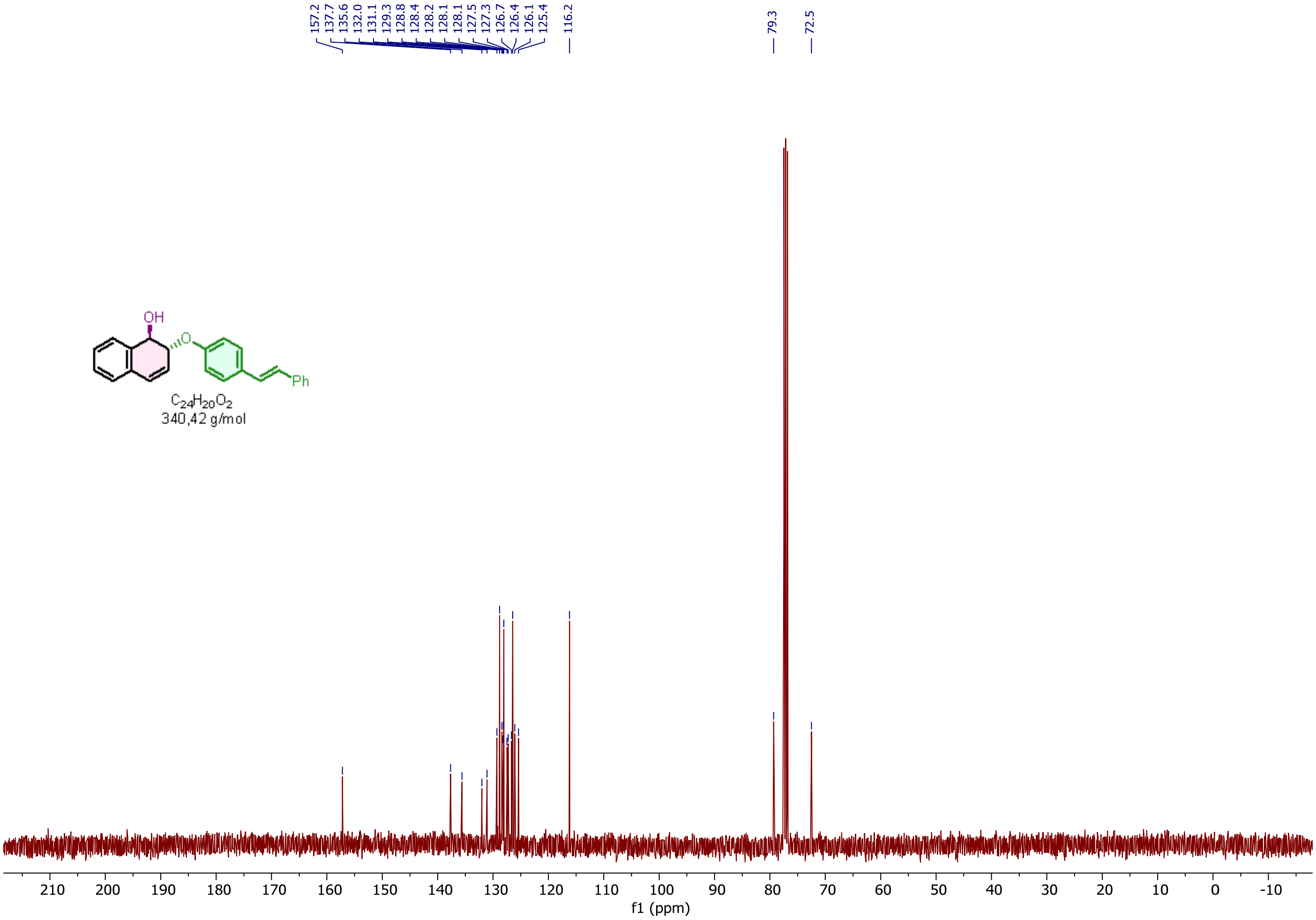

^{1}H NMR (400 MHz, $CDCl_3$) and $^{13}C\{^{1}H\}$ NMR (101 MHz, $CDCl_3$) Analysis of Compound **8a**

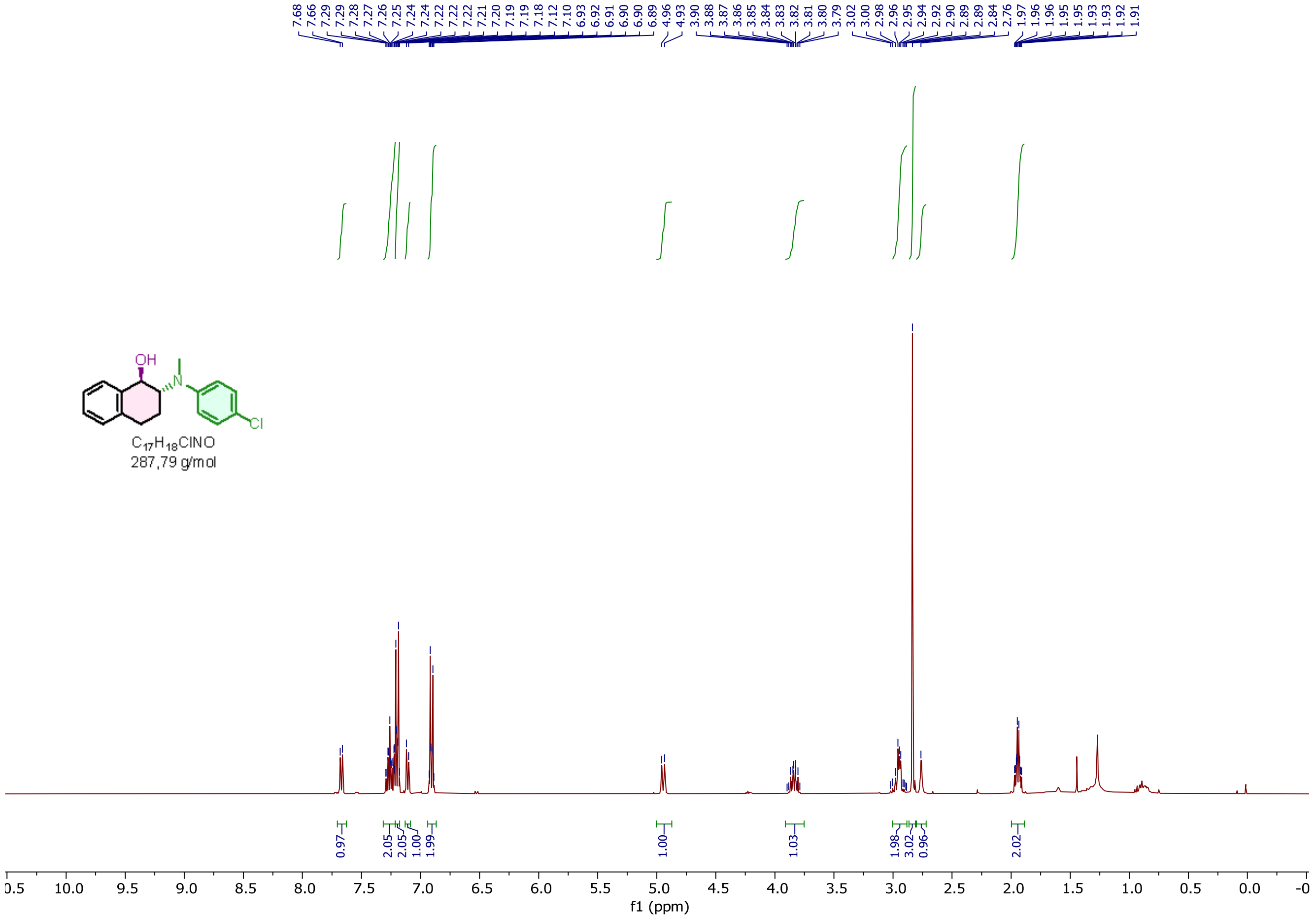

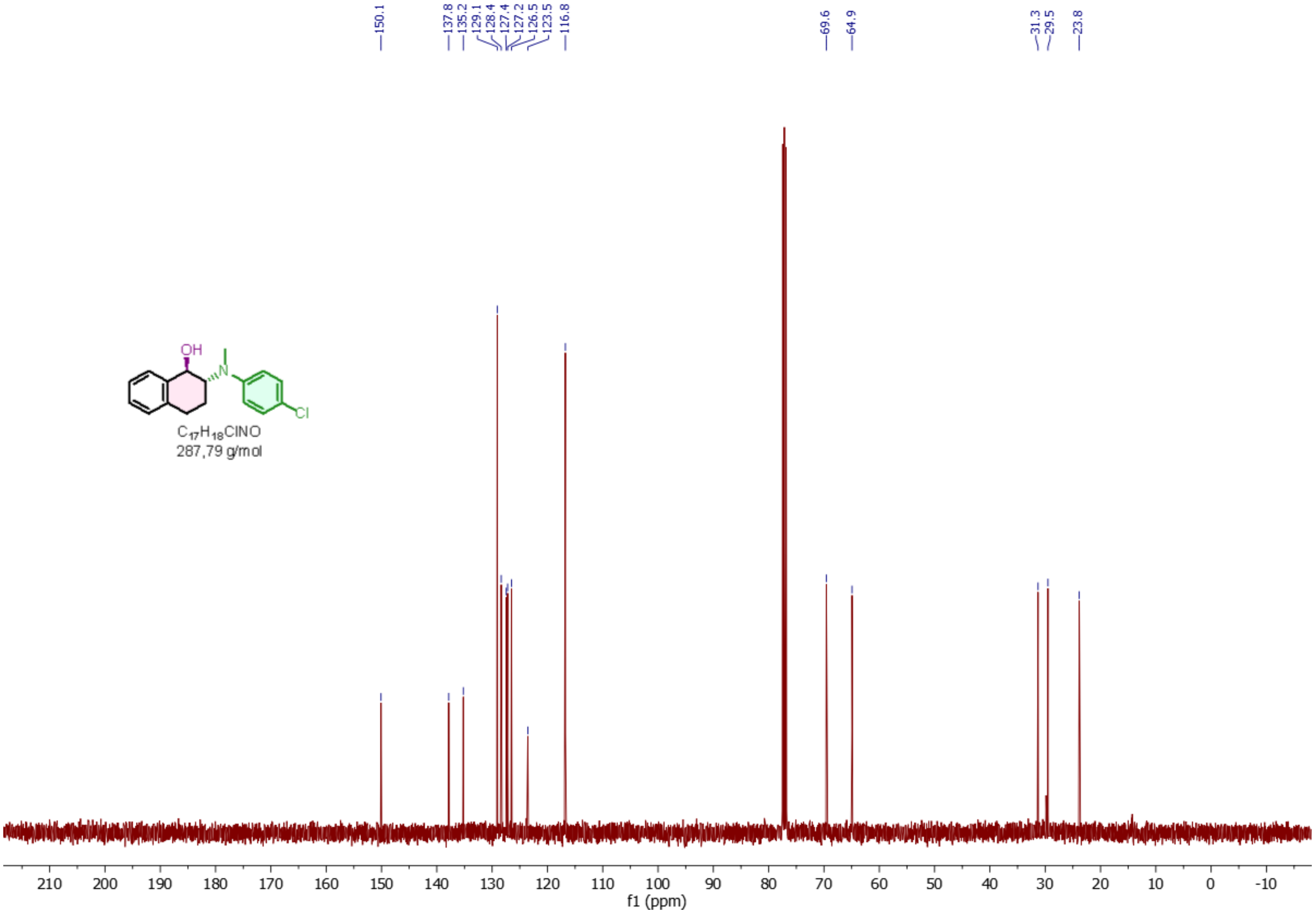

^{1}H NMR (400 MHz, $CDCl_3$) and $^{13}C\{^{1}H\}$ NMR (101 MHz, $CDCl_3$) Analysis of Compound **8b**

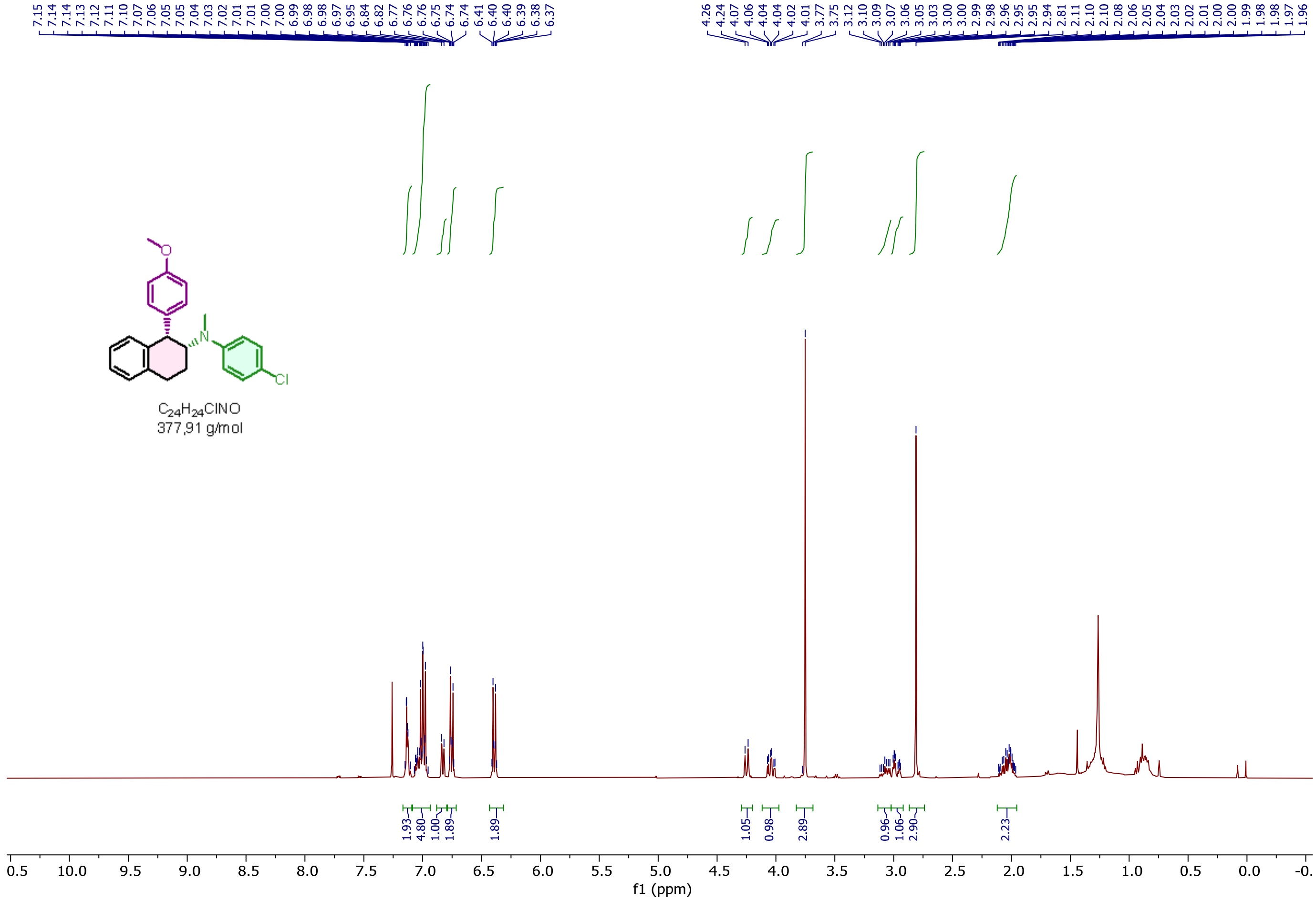

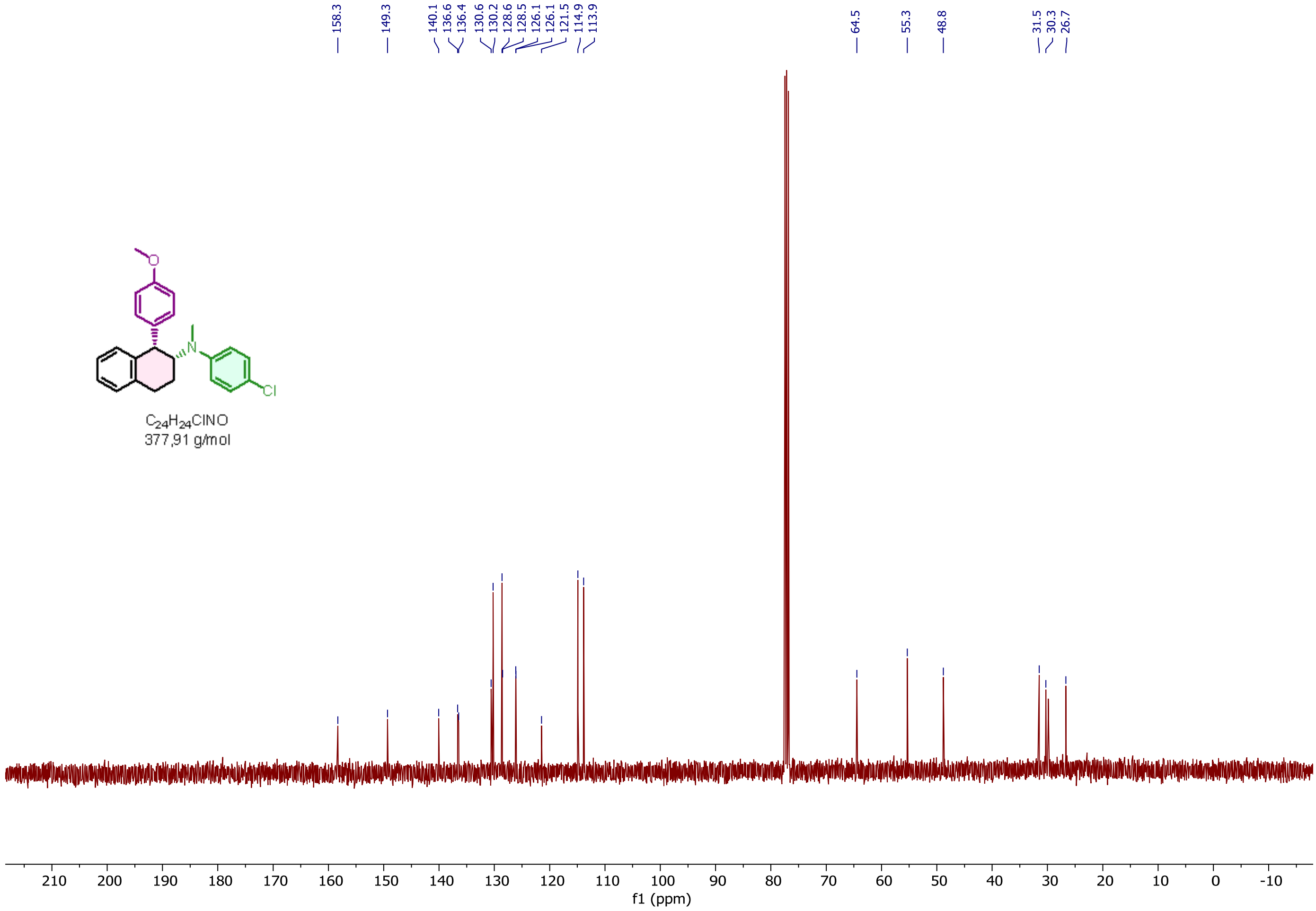

^{1}H NMR (400 MHz, $CDCl_3$) and $^{13}C\{^{1}H\}$ NMR (101 MHz, $CDCl_3$) Analysis of Compound **8c**

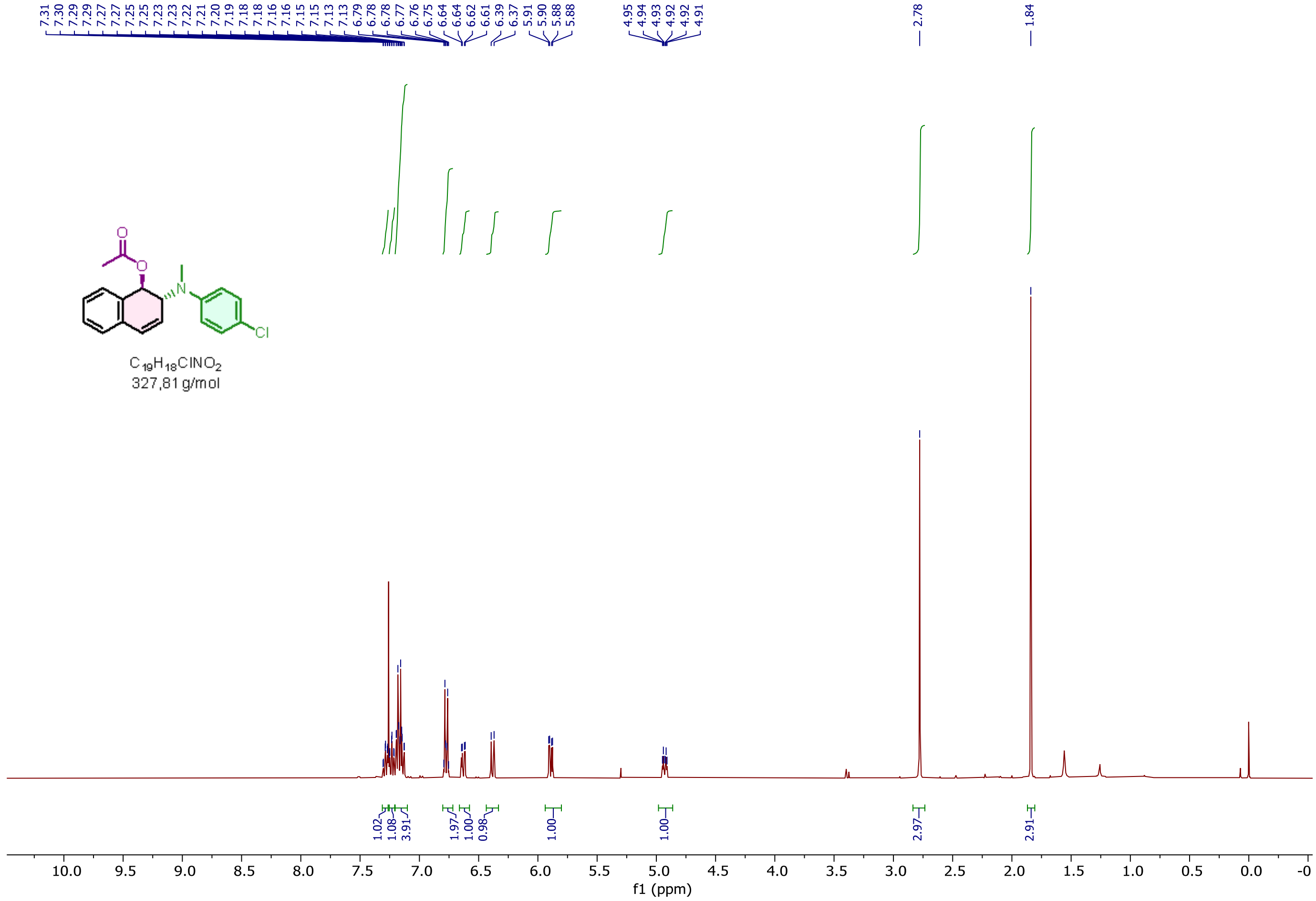

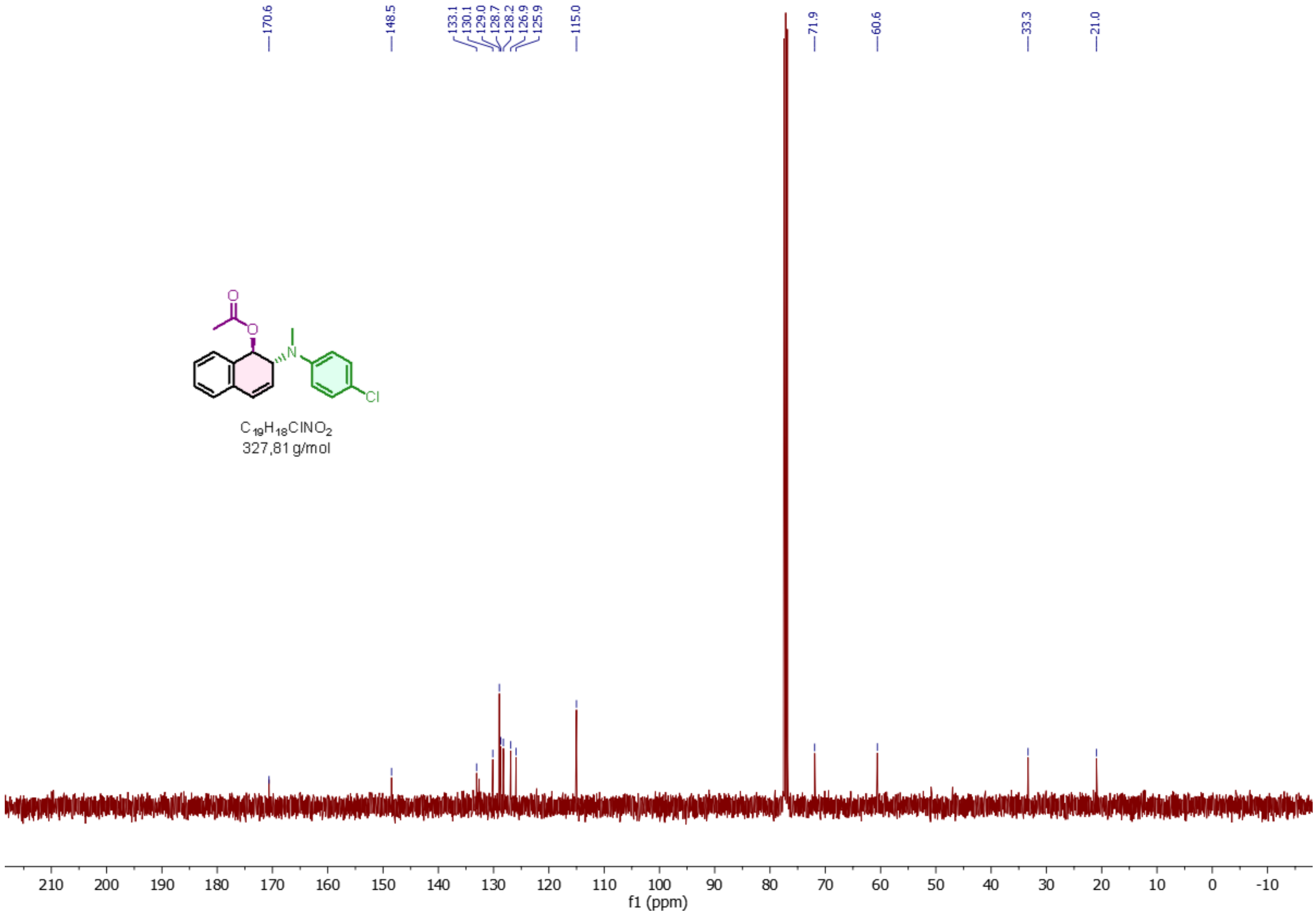

[1] H NMR (400 MHz, $CDCl_3$) and [13] C{[1] H} NMR (101 MHz, $CDCl_3$) Analysis of Compound **8d**

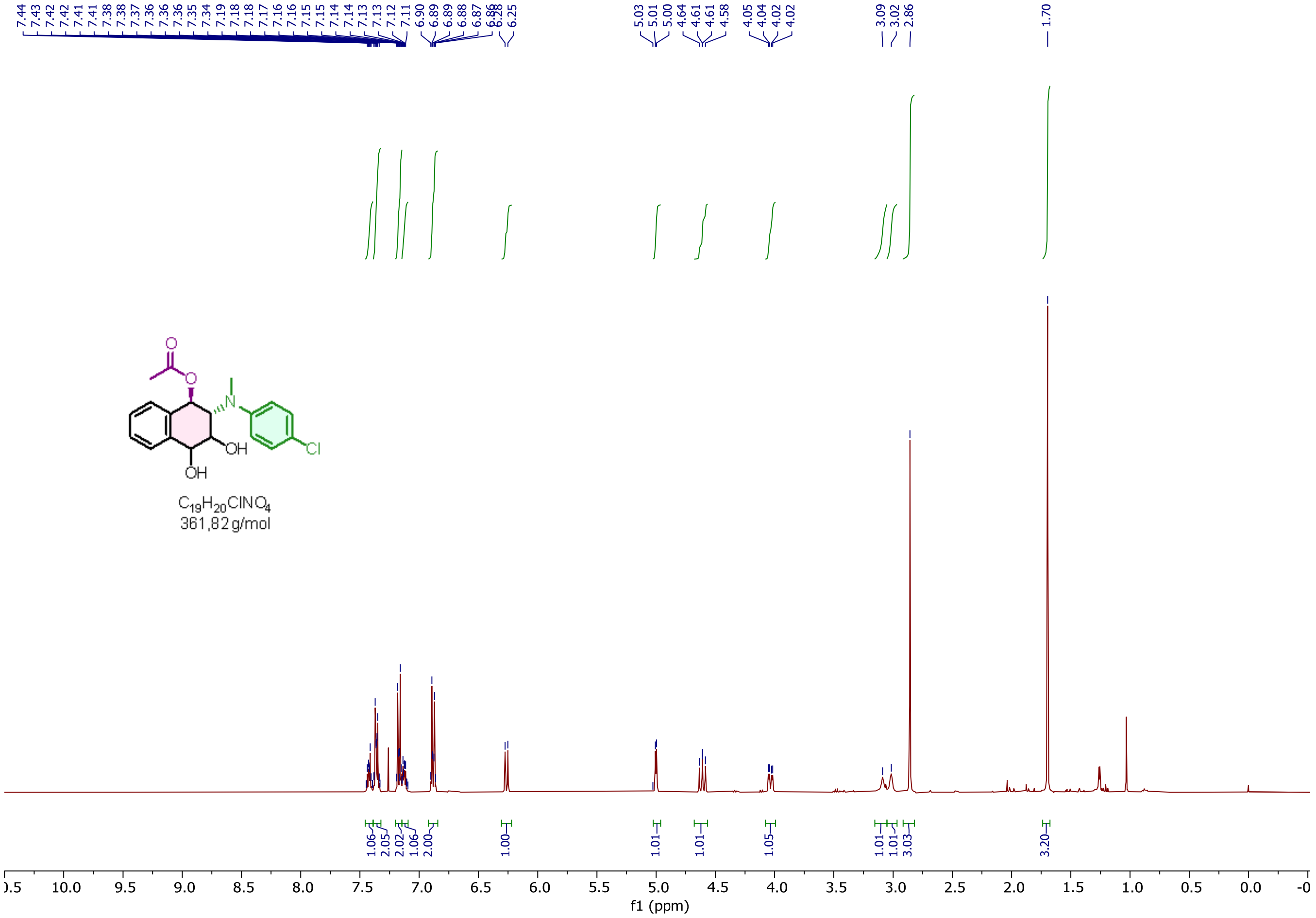

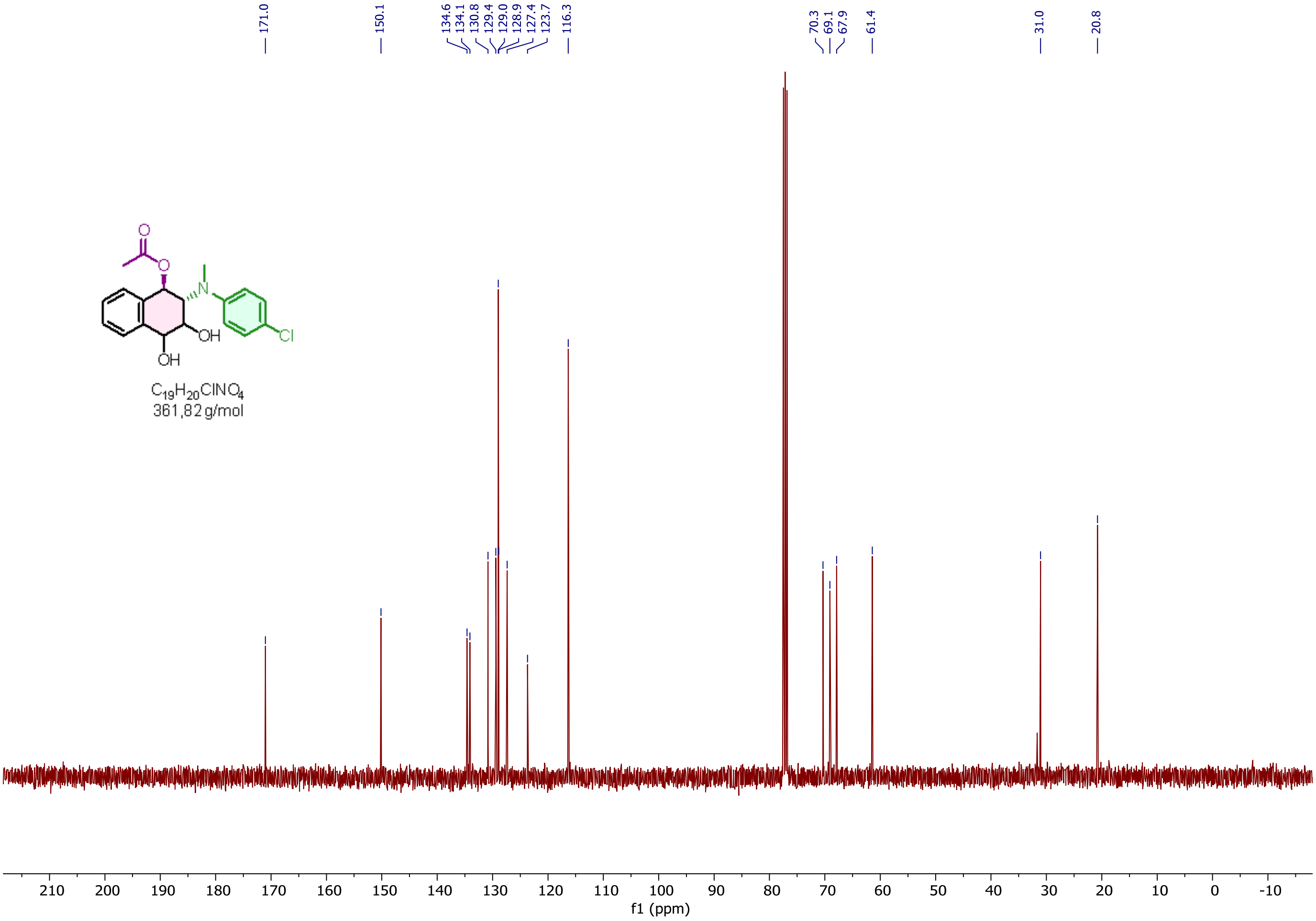

^{1}H NMR (400 MHz, $CDCl_3$) and $^{13}C\{^1H\}$ NMR (101 MHz, $CDCl_3$) Analysis of Compound **8e**

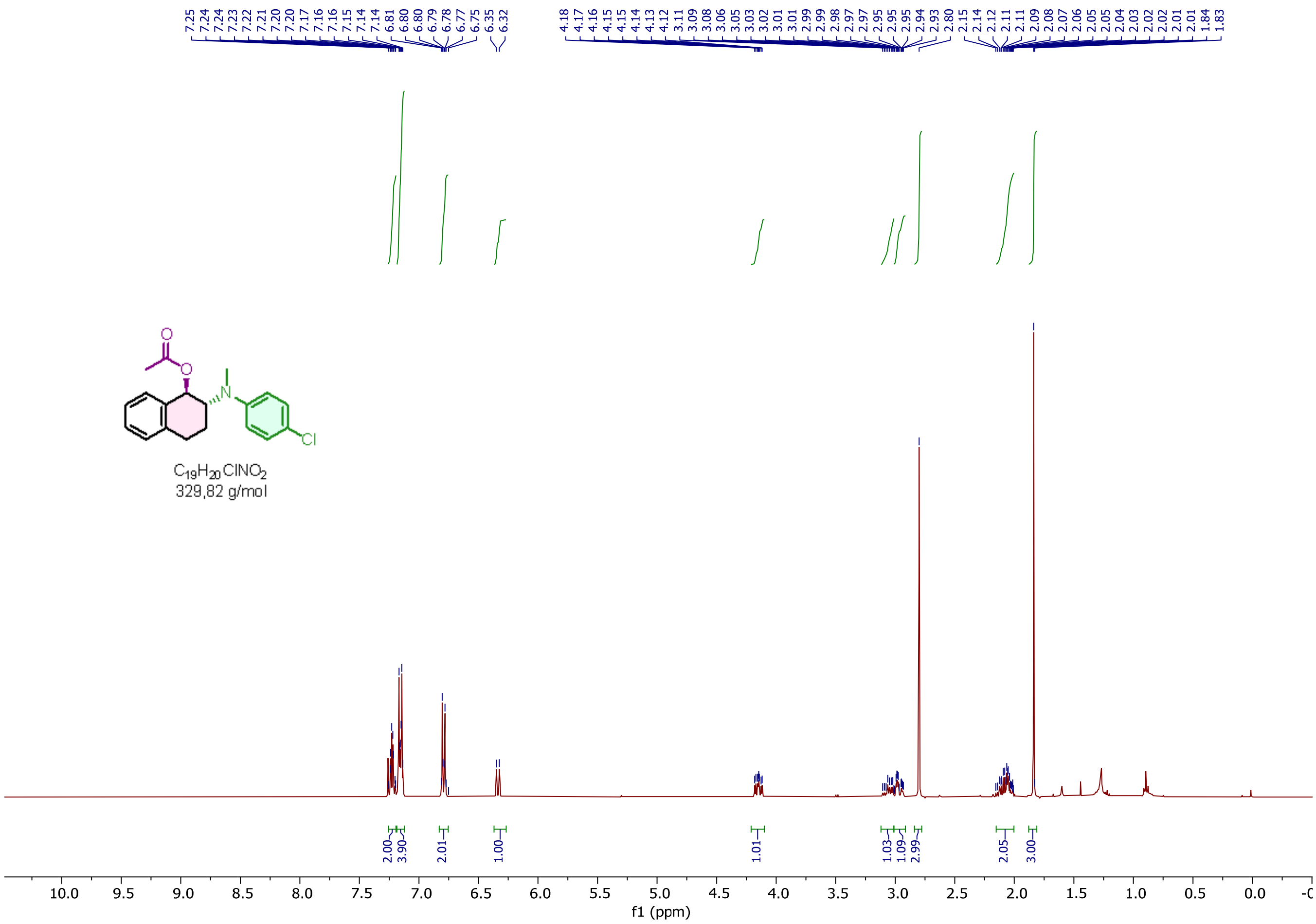

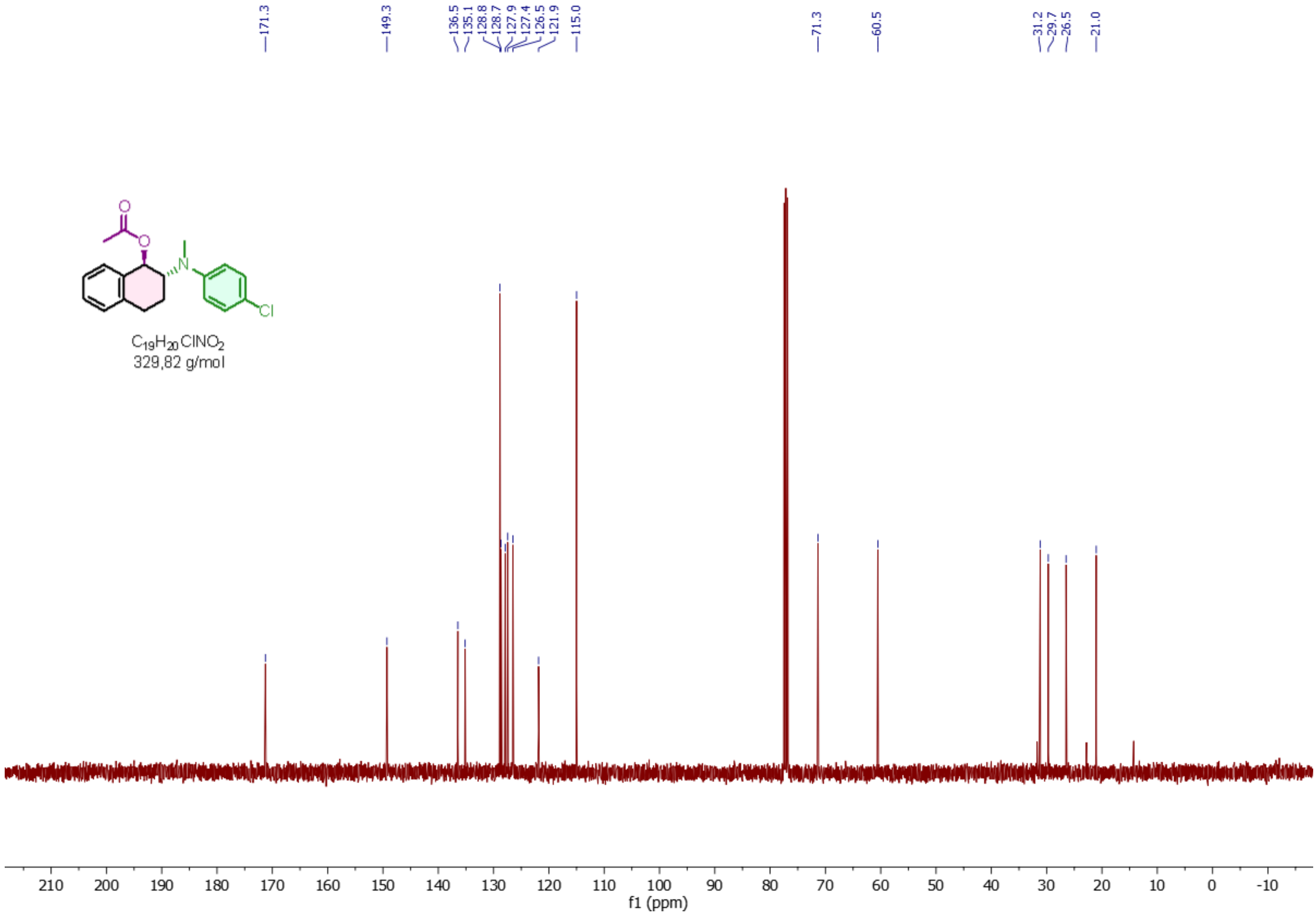

Supplement: Supplementary file 3 [file jo5c02582_si_003.pdf]
